# Supplementary material for: Insights into Molecular Mechanism of Secondary Xylem Rapid Growth in Salix psammophila
Source: Plants (Basel). 2025 Feb 5;14(3):459. doi: 10.3390/plants14030459 (PMC11819810; doi:10.3390/plants14030459)
Supplement: Supplementary file 1 [file plants-14-00459-s001.zip › Supplementary Table/Table S1 .pdf]

Table S1 Quality control of transcriptome sequencing data for secondary xylem and phloem of *Salix psammophila*.

	R1-before-total-reads	R1-before-total-bases	R1-before-Q20	R1-before-Q30	R1-after-total-reads	R1-after-total-bases	R1-after-Q20	R1-after-Q30	R2-before-total-reads	R2-before-total-bases	R2-before-Q20	R2-before-Q30	R2-after-total-reads	R2-after-total-bases	R2-after-Q20	R2-after-Q30
**1a_m_1**	25619465	3842919750	98.00%	96.25%	24533817	3655361294	98.60%	97.48%	25619465	3842919750	97.65%	95.34%	24533817	3655361294	98.60%	97.22%
**1a_m_2**	21461837	3219275550	98.27%	96.79%	20815194	3106479158	98.70%	97.65%	21461837	3219275550	97.97%	96.05%	20815194	3106479158	98.63%	97.30%
**1a_m_3**	20711540	3106731000	97.92%	95.97%	19984762	2981499544	98.43%	97.01%	20711540	3106731000	97.56%	95.19%	19984762	2981499544	98.38%	96.77%
**1a_r_1**	24299446	3644916900	98.36%	97.00%	23628646	3527295850	98.77%	97.81%	24299446	3644916900	97.97%	96.05%	23628646	3527295850	98.65%	97.35%
**1a_r_2**	19736300	2960445000	98.48%	97.20%	19216123	2868162724	98.86%	97.95%	19736300	2960445000	98.15%	96.38%	19216123	2868162724	98.75%	97.51%
**1a_r_3**	26359120	3953868000	98.22%	96.77%	25471953	3797440518	98.70%	97.74%	26359120	3953868000	97.87%	95.91%	25471953	3797440518	98.63%	97.35%
**2a_m_1**	23308423	3496263450	98.34%	96.94%	22515396	3356472090	98.80%	97.85%	23308423	3496263450	98.01%	96.12%	22515396	3356472090	98.69%	97.40%
**2a_m_2**	35410771	5311615650	97.65%	95.54%	33249931	4922974451	98.45%	97.13%	35410771	5311615650	97.68%	95.63%	33249931	4922974451	98.62%	97.37%
**2a_m_3**	33319846	4997976900	97.97%	96.33%	31896624	4729674197	98.58%	97.53%	33319846	4997976900	97.71%	95.62%	31896624	4729674197	98.69%	97.48%
**2a_r_1**	22674034	3401105100	98.26%	96.86%	21991046	3281444411	98.71%	97.75%	22674034	3401105100	97.93%	96.01%	21991046	3281444411	98.63%	97.34%
**2a_r_2**	23443513	3516526950	98.19%	96.74%	22672913	3378956825	98.68%	97.70%	23443513	3516526950	97.84%	95.82%	22672913	3378956825	98.63%	97.34%
**2a_r_3**	22524319	3378647850	98.31%	96.91%	21884269	3268139503	98.73%	97.74%	22524319	3378647850	97.89%	95.91%	21884269	3268139503	98.61%	97.27%
**3a_m_1**	20163523	3024528450	98.40%	97.07%	19595220	2925236945	98.82%	97.90%	20163523	3024528450	98.14%	96.38%	19595220	2925236945	98.77%	97.57%
**3a_m_2**	37370344	5605551600	98.08%	96.52%	35956810	5350705663	98.63%	97.61%	37370344	5605551600	97.89%	95.97%	35956810	5350705663	98.67%	97.44%
**3a_m_3**	20044489	3006673350	98.29%	96.88%	19465171	2906696628	98.73%	97.75%	20044489	3006673350	97.95%	96.01%	19465171	2906696628	98.63%	97.31%
**3a_r_1**	24399724	3659958600	98.17%	96.67%	23489417	3498129044	98.70%	97.70%	24399724	3659958600	97.81%	95.80%	23489417	3498129044	98.60%	97.28%
**3a_r_2**	21872285	3280842750	98.27%	96.87%	21218887	3168409465	98.71%	97.75%	21872285	3280842750	97.85%	95.83%	21218887	3168409465	98.64%	97.34%
**3a_r_3**	24703626	3705543900	98.31%	96.95%	24002981	3584672048	98.73%	97.79%	24703626	3705543900	98.03%	96.18%	24002981	3584672048	98.67%	97.40%
