# Supplementary material for: Insights into Molecular Mechanism of Secondary Xylem Rapid Growth in Salix psammophila
Source: Plants (Basel). 2025 Feb 5;14(3):459. doi: 10.3390/plants14030459 (PMC11819810; doi:10.3390/plants14030459)
Supplement: Supplementary file 1 [file plants-14-00459-s001.zip › Supplementary Table/Table S10.pdf]

Table S10 Genes regulating brown module-related transcription factors.

X	Description	PFAMs
**Sapur.002G045200**	Myb-like DNA-binding domain	Myb_DNA-binding
**Sapur.002G092000**	transcription factor	Myb_DNA-binding
**Sapur.002G092000**	transcription factor	Myb_DNA-binding
**Sapur.002G106500**	Transcription factor	Myb_DNA-binding
**Sapur.002G106500**	Transcription factor	Myb_DNA-binding
**Sapur.002G106500**	transcription factor KAN4	Myb_DNA-binding
**Sapur.003G022000**	Myb-related protein	Myb_DNA-binding
**Sapur.003G037600**	Plant lipoxygenase may be involved in a number of diverse aspects of plant physiology including growth and development, pest resistance, and senescence or responses to wounding	Lipoxygenase,PLAT
**Sapur.003G106800**	SANT SWI3, ADA2, N-CoR and TFIIIB'' DNA-binding domains	Myb_DNA-binding
**Sapur.004G020300**	transcription factor	Myb_DNA-binding
**Sapur.004G073900**	Transcription factor	Myb_DNA-binding
**Sapur.004G162300**	SANT SWI3, ADA2, N-CoR and TFIIIB'' DNA-binding domains	Myb_DNA-binding
**Sapur.005G057900**	Transcription factor	Myb_DNA-binding
**Sapur.005G128900**	SANT SWI3, ADA2, N-CoR and TFIIIB'' DNA-binding domains	Myb_DNA-binding
**Sapur.005G128900**	SANT SWI3, ADA2, N-CoR and TFIIIB'' DNA-binding domains	Myb_DNA-binding
**Sapur.006G101600**	SANT SWI3, ADA2, N-CoR and TFIIIB'' DNA-binding domains	Myb_DNA-binding
**Sapur.007G058100**	Myb-related protein	Myb_DNA-binding
**Sapur.007G085400**	Transcription factor	Myb_DNA-binding
**Sapur.008G056800**	Myb-like DNA-binding domain	Myb_DNA-binding
**Sapur.008G098500**	Transcription factor	Myb_DNA-binding
**Sapur.008G114700**	Myb family transcription factor	Myb_DNA-binding
**Sapur.009G003800**	SANT SWI3, ADA2, N-CoR and TFIIIB'' DNA-binding domains	Myb_DNA-binding
**Sapur.010G073800**	Myb family transcription factor	Myb_DNA-binding
**Sapur.010G116200**	Transcription factor	Myb_DNA-binding
**Sapur.010G145600**	Myb-like DNA-binding domain	Myb_DNA-binding
**Sapur.010G145600**	Myb-like DNA-binding domain	Myb_DNA-binding
**Sapur.011G091900**	SANT SWI3, ADA2, N-CoR and TFIIIB'' DNA-binding domains	Myb_DNA-binding
**Sapur.012G038400**	transcription factor	Myb_DNA-binding
**Sapur.016G088300**	SANT SWI3, ADA2, N-CoR and TFIIIB'' DNA-binding domains	Myb_DNA-binding
**Sapur.016G186600**	Trihelix transcription factor	Myb_DNA-bind_4

**Sapur.016G211100**	Myb-related protein	DUF971,Myb_DNA-binding
**Sapur.016G266600**	SANT SWI3, ADA2, N-CoR and TFIIIB'' DNA-binding domains	Myb_DNA-binding
**Sapur.019G079300**	transcription regulator recruiting activity	Myb_DNA-binding
**Sapur.019G104800**	transcription factor	Myb_DNA-binding
**Sapur.15WG045300**	transcription factor	Myb_DNA-binding
**Sapur.15ZG048700**	transcription factor	Myb_DNA-binding
**Sapur.15ZG135000**	SWI SNF complex subunit	Myb_DNA-binding,SWIRM,SWIRM-assoc_1
**Sapur.T099500**	HSA	HSA,Myb_DNA-bind_6
**Sapur.T115700**	Myb-like DNA-binding domain	Myb_DNA-binding
