# Supplementary material for: Insights into Molecular Mechanism of Secondary Xylem Rapid Growth in Salix psammophila
Source: Plants (Basel). 2025 Feb 5;14(3):459. doi: 10.3390/plants14030459 (PMC11819810; doi:10.3390/plants14030459)
Supplement: Supplementary file 1 [file plants-14-00459-s001.zip › Supplementary Table/Table S11.pdf]

Table S11 Genes regulating gray module-related transcription factors.

X	Description	PFAMs
**Sapur.001G011800**	Plant lipoxygenase may be involved in a number of diverse aspects of plant physiology including growth and development, pest resistance, and senescence or responses to wounding	Lipoxygenase,PLAT
**Sapur.001G012100**	Plant lipoxygenase may be involved in a number of diverse aspects of plant physiology including growth and development, pest resistance, and senescence or responses to wounding	Lipoxygenase,PLAT
**Sapur.001G012200**	Plant lipoxygenase may be involved in a number of diverse aspects of plant physiology including growth and development, pest resistance, and senescence or responses to wounding	Lipoxygenase,PLAT
**Sapur.001G012200**	Plant lipoxygenase may be involved in a number of diverse aspects of plant physiology including growth and development, pest resistance, and senescence or responses to wounding	Lipoxygenase,PLAT
**Sapur.001G012300**	Plant lipoxygenase may be involved in a number of diverse aspects of plant physiology including growth and development, pest resistance, and senescence or responses to wounding	Lipoxygenase,PLAT
**Sapur.001G012500**	Plant lipoxygenase may be involved in a number of diverse aspects of plant physiology including growth and development, pest resistance, and senescence or responses to wounding	Lipoxygenase,PLAT

**Sapur.001G012600**	Plant lipoxygenase may be involved in a number of diverse aspects of plant physiology including growth and development, pest resistance, and senescence or responses to wounding	Lipoxygenase,PLAT
**Sapur.001G012700**	Plant lipoxygenase may be involved in a number of diverse aspects of plant physiology including growth and development, pest resistance, and senescence or responses to wounding	Lipoxygenase,PLAT
**Sapur.001G012800**	Plant lipoxygenase may be involved in a number of diverse aspects of plant physiology including growth and development, pest resistance, and senescence or responses to wounding	Lipoxygenase,PLAT
**Sapur.001G043700**	Endoribonuclease ysh1	Beta-Casp,CPSF73-100_C,Lactamase_B,Lactamase_B_6,RMMBL
**Sapur.001G064700**	SANT SWI3, ADA2, N-CoR and TFIIIB'' DNA-binding domains	Myb_DNA-binding
**Sapur.001G070000**	SANT SWI3, ADA2, N-CoR and TFIIIB'' DNA-binding domains	Myb_DNA-binding
**Sapur.001G070200**	SANT SWI3, ADA2, N-CoR and TFIIIB'' DNA-binding domains	Myb_DNA-binding
**Sapur.001G106800**	Trihelix transcription factor	Myb_DNA-bind_4
**Sapur.001G109700**	Myb family transcription factor	Myb_CC_LHEQLE,Myb_DNA-binding
**Sapur.001G138200**	PLATZ transcription factor	PLATZ
**Sapur.001G172000**	Myb SANT-like DNA-binding domain protein	Myb_DNA-bind_3
**Sapur.002G014200**	Synaptotagmin-like mitochondrial-lipid-binding domain	C2,SMP_LBD
**Sapur.002G014200**	Synaptotagmin-like mitochondrial-lipid-binding	C2,SMP_LBD

	domain	
**Sapur.002G014200**	Synaptotagmin-like mitochondrial-lipid-binding domain	C2,SMP_LBD
**Sapur.002G017800**	Myb/SANT-like DNA-binding domain	Myb_DNA-bind_3
**Sapur.002G017800**	Myb/SANT-like DNA-binding domain	Myb_DNA-bind_3
**Sapur.002G017800**	Myb/SANT-like DNA-binding domain	Myb_DNA-bind_3
**Sapur.002G053400**	Trihelix transcription factor	Myb_DNA-bind_4
**Sapur.002G053500**	Trihelix transcription factor	Myb_DNA-bind_4
**Sapur.002G105300**	transcription factor	Myb_DNA-binding
**Sapur.002G115100**	Myb-related protein	Myb_DNA-binding
**Sapur.002G118100**	PLATZ transcription factor	PLATZ
**Sapur.002G133300**	Myb SANT-like DNA-binding domain protein	Myb_DNA-bind_3
**Sapur.002G140000**	SANT SWI3, ADA2, N-CoR and TFIIIB'' DNA-binding domains	Myb_DNA-binding
**Sapur.002G145900**	SANT SWI3, ADA2, N-CoR and TFIIIB'' DNA-binding domains	Myb_DNA-binding
**Sapur.002G161500**	transcription factor	Myb_DNA-binding,P_C
**Sapur.002G200800**	Fanconi anemia group F protein (FANCF)	FANCF,Myb_CC_LHEQLE,Myb_DNA-binding
**Sapur.002G200800**	Fanconi anemia group F protein (FANCF)	FANCF,Myb_CC_LHEQLE,Myb_DNA-binding
**Sapur.003G021600**	Myb-related protein	Myb_DNA-binding
**Sapur.003G040200**	transcription factor	Myb_DNA-bind_4
**Sapur.003G047200**	transcription factor	Myb_DNA-binding
**Sapur.003G055900**	Two-component response regulator-like	Myb_DNA-binding,Response_reg
**Sapur.003G055900**	Two-component response regulator-like	Myb_DNA-binding,Response_reg
**Sapur.003G055900**	Two-component response regulator-like	Myb_DNA-binding,Response_reg
**Sapur.003G055900**	Two-component response regulator-like	Myb_DNA-binding,Response_reg
**Sapur.003G055900**	Two-component response regulator-like	Myb_DNA-binding,Response_reg
**Sapur.003G055900**	Two-component response regulator-like	Myb_DNA-binding,Response_reg
**Sapur.003G055900**	Two-component response	Myb_DNA-binding,Response_

	regulator-like	reg
**Sapur.003G055900**	Two-component response regulator-like	Myb_DNA-binding,Response_reg
**Sapur.003G055900**	Two-component response regulator-like	Myb_DNA-binding,Response_reg
**Sapur.003G055900**	Two-component response regulator-like	Myb_DNA-binding,Response_reg
**Sapur.003G055900**	Two-component response regulator-like	Myb_DNA-binding,Response_reg
**Sapur.003G055900**	Two-component response regulator-like	Myb_DNA-binding,Response_reg
**Sapur.003G066600**	Myb family transcription factor	Myb_CC_LHEQLE,Myb_DNA-binding
**Sapur.003G088800**	SANT SWI3, ADA2, N-CoR and TFIIIB" DNA-binding domains	Myb_DNA-binding
**Sapur.003G088800**	SANT SWI3, ADA2, N-CoR and TFIIIB" DNA-binding domains	Myb_DNA-binding
**Sapur.003G161700**	Bromodomain-containing protein	Bromodomain,Myb_DNA-binding
**Sapur.003G161700**	Bromodomain-containing protein	Bromodomain,Myb_DNA-binding
**Sapur.004G006000**	transcription factor	Myb_DNA-binding
**Sapur.004G016300**	Myb-related protein	Myb_DNA-binding
**Sapur.004G044300**	Myb SANT-like DNA-binding domain protein	Myb_DNA-bind_3
**Sapur.004G070700**	transcription factor	Myb_DNA-binding
**Sapur.004G105300**	Myb-like DNA-binding domain	Myb_DNA-binding
**Sapur.004G116600**	SANT SWI3, ADA2, N-CoR and TFIIIB" DNA-binding domains	Myb_DNA-binding
**Sapur.004G117100**	RADIALIS-like	Myb_DNA-binding
**Sapur.004G145400**	Myb SANT-like DNA-binding domain protein	Myb_DNA-bind_3
**Sapur.005G095000**	Transcription factor MYB98-like	Myb_DNA-binding
**Sapur.005G097600**	atrl6,rl6,rsm3	Myb_DNA-binding
**Sapur.005G098200**	SANT SWI3, ADA2, N-CoR and TFIIIB" DNA-binding domains	Myb_DNA-binding
**Sapur.005G103600**	PLATZ transcription factor	PLATZ
**Sapur.005G123100**	nuclease activity	DDE_Tnp_4,Myb_DNA-bind_3
**Sapur.005G123500**	Myb SANT-like DNA-binding	Myb_DNA-bind_3

	domain protein	
**Sapur.005G151200**	Trihelix transcription factor	Myb_DNA-bind_4
**Sapur.005G178500**	Myb-related protein	Myb_DNA-binding
**Sapur.006G051100**	Synaptotagmin-5-like	C2,SMP_LBD
**Sapur.006G082700**	MYB-CC type transfactor, LHEQLE motif	Myb_CC_LHEQLE,Myb_DNA-binding
**Sapur.006G083100**	Myb/SANT-like DNA-binding domain	Myb_DNA-bind_4
**Sapur.006G083100**	Myb/SANT-like DNA-binding domain	Myb_DNA-bind_4
**Sapur.006G100400**	SANT SWI3, ADA2, N-CoR and TFIIIB'' DNA-binding domains	Myb_DNA-binding
**Sapur.006G104600**	Myb/SANT-like DNA-binding domain	Myb_DNA-bind_3
**Sapur.006G124400**	transcription, DNA-templated	Myb_DNA-binding
**Sapur.006G138500**	Myb SANT-like DNA-binding domain protein	Myb_DNA-bind_3
**Sapur.006G139600**	transcription factor	Myb_DNA-binding
**Sapur.006G145500**	Myb SANT-like DNA-binding domain protein	Myb_DNA-bind_3
**Sapur.006G190200**	transcription, DNA-templated	Myb_DNA-binding
**Sapur.006G225900**	RNA polymerase II transcription regulator recruiting activity	Myb_DNA-binding
**Sapur.006G226300**	RNA polymerase II transcription regulator recruiting activity	Myb_DNA-binding
**Sapur.006G226300**	RNA polymerase II transcription regulator recruiting activity	Myb_DNA-binding
**Sapur.006G226600**	RNA polymerase II transcription regulator recruiting activity	Myb_DNA-binding
**Sapur.006G227000**	RNA polymerase II transcription regulator recruiting activity	Myb_DNA-binding
**Sapur.007G006000**	transcription factor	Myb_DNA-binding
**Sapur.007G026400**	nuclease activity	DDE_Tnp_4,Myb_DNA-bind_3
**Sapur.007G031100**	PLATZ transcription factor	PLATZ
**Sapur.007G057000**	HSA	HSA,Myb_DNA-bind_6
**Sapur.007G057600**	histone H4-K5 acetylation	HSA,Myb_DNA-bind_6
**Sapur.007G057700**	HSA	HSA,Myb_DNA-bind_6
**Sapur.007G057800**	HSA	HSA,Myb_DNA-bind_6
**Sapur.007G060400**	Myb SANT-like DNA-binding domain protein	Myb_DNA-bind_3
**Sapur.007G122400**	transcription	Myb_DNA-binding
**Sapur.007G122400**	transcription	Myb_DNA-binding

**Sapur.008G018300**	Amino acid kinase family	AA_kinase,Myb_DNA-bind_4
**Sapur.008G018300**	Amino acid kinase family	AA_kinase,Myb_DNA-bind_4
**Sapur.008G056100**	Transcription factor	Myb_DNA-binding
**Sapur.008G064900**	transcription	Myb_DNA-binding
**Sapur.008G069400**	transcription factor	Myb_DNA-binding
**Sapur.008G070500**	SANT SWI3, ADA2, N-CoR and TFIIIB'' DNA-binding domains	Myb_DNA-binding
**Sapur.009G022300**	Myb SANT-like DNA-binding domain protein	Myb_DNA-bind_3
**Sapur.009G032800**	SANT SWI3, ADA2, N-CoR and TFIIIB'' DNA-binding domains	Myb_DNA-binding
**Sapur.009G045100**	Myb SANT-like DNA-binding domain protein	Myb_DNA-bind_3
**Sapur.009G058600**	Myb-like DNA-binding domain	Myb_DNA-binding
**Sapur.009G058600**	Myb-like DNA-binding domain	Myb_DNA-binding
**Sapur.009G075300**	Transcription factor	Myb_DNA-binding
**Sapur.009G092600**	RADIALIS-like	Myb_DNA-binding
**Sapur.010G003200**	Transcription factor	Myb_DNA-binding
**Sapur.010G018400**	nuclease activity	DDE_Tnp_4,Myb_DNA-bind_3
**Sapur.010G018600**	nuclease activity	DDE_Tnp_4,Myb_DNA-bind_3
**Sapur.010G041000**	RNA polymerase II transcription regulator recruiting activity	Myb_DNA-binding
**Sapur.010G051200**	Myb SANT-like DNA-binding domain protein	Myb_DNA-bind_3
**Sapur.010G110200**	transcription factor	Myb_DNA-binding
**Sapur.010G128000**	Transcription factor	Myb_DNA-binding
**Sapur.010G129100**	transcription factor	Myb_DNA-binding
**Sapur.010G129300**	Myb family transcription factor	Myb_CC_LHEQLE,Myb_DNA-binding
**Sapur.010G129300**	Myb family transcription factor	Myb_CC_LHEQLE,Myb_DNA-binding
**Sapur.010G135300**	transcription	Myb_DNA-binding
**Sapur.010G135300**	transcription	Myb_DNA-binding
**Sapur.010G179300**	Synaptotagmin-3-like	C2,SMP_LBD
**Sapur.011G061800**	Myb SANT-like DNA-binding domain protein	Myb_DNA-bind_3
**Sapur.011G087000**	Myb SANT-like DNA-binding domain protein	Myb_DNA-bind_3
**Sapur.011G123900**	transcription factor	Myb_DNA-binding
**Sapur.012G052700**	Transcription factor	Myb_DNA-binding

**Sapur.012G059600**	Transcription factor WER-like	Myb_DNA-binding
**Sapur.012G081500**	Myb/SANT-like DNA-binding domain	Myb_DNA-bind_4
**Sapur.012G102800**	response regulator	Myb_DNA-binding,Response_reg
**Sapur.012G108000**	Transcription factor	Myb_DNA-binding
**Sapur.013G037000**	Trihelix transcription factor	Myb_DNA-bind_4
**Sapur.013G070200**	Myb-related protein	Myb_DNA-binding
**Sapur.013G070200**	Myb-related protein	Myb_DNA-binding
**Sapur.013G076200**	HSA	HSA,Myb_DNA-bind_6
**Sapur.013G076500**	HSA	HSA,Myb_DNA-bind_6
**Sapur.013G076600**	histone H4-K5 acetylation	HSA,Myb_DNA-bind_6
**Sapur.013G079700**	Myb SANT-like DNA-binding domain protein	Myb_DNA-bind_3
**Sapur.013G103200**	transcription regulator recruiting activity	Myb_DNA-binding
**Sapur.013G132600**	transcription factor	Myb_DNA-binding
**Sapur.013G133000**	Myb-related protein	Myb_DNA-binding
**Sapur.014G020700**	HSA	HSA,Myb_DNA-bind_6
**Sapur.014G020800**	histone H4-K5 acetylation	HSA,Myb_DNA-bind_6
**Sapur.014G021000**	histone H4-K5 acetylation	HSA,Myb_DNA-bind_6
**Sapur.014G021800**	HSA	HSA,Myb_DNA-bind_6
**Sapur.014G026900**	Transcription factor	Myb_DNA-binding
**Sapur.014G041400**	Myb-related protein	Myb_DNA-binding
**Sapur.014G054400**	Synaptotagmin-like mitochondrial-lipid-binding domain	C2,SMP_LBD
**Sapur.014G057900**	response regulator	Myb_DNA-binding,Response_reg
**Sapur.014G061000**	SANT SWI3, ADA2, N-CoR and TFIIIB'' DNA-binding domains	Myb_DNA-binding
**Sapur.014G074900**	transcription factor	Myb_DNA-binding
**Sapur.014G100400**	Two-component response regulator	Myb_DNA-binding,Response_reg
**Sapur.014G141400**	MYB-CC type transfactor, LHEQLE motif	Myb_CC_LHEQLE,Myb_DNA-binding
**Sapur.014G143100**	Myb SANT-like DNA-binding domain protein	Myb_DNA-bind_3
**Sapur.016G102000**	MYB-CC type transfactor, LHEQLE motif	Myb_CC_LHEQLE,Myb_DNA-binding
**Sapur.016G102500**	Myb/SANT-like DNA-binding domain	Myb_DNA-bind_4
**Sapur.016G140300**	SANT SWI3, ADA2, N-CoR	Myb_DNA-binding

	and TFIIIB" DNA-binding domains	
**Sapur.016G141000**	Trihelix transcription factor	Myb_DNA-bind_4
**Sapur.016G164600**	Myb-like DNA-binding domain	Myb_DNA-binding
**Sapur.016G219200**	Myb-related protein	Myb_DNA-binding
**Sapur.016G219700**	SANT SWI3, ADA2, N-CoR and TFIIIB" DNA-binding domains	Myb_DNA-binding
**Sapur.017G008600**	transcription	Myb_DNA-binding
**Sapur.017G012300**	Myb SANT-like DNA-binding domain protein	Myb_DNA-bind_3
**Sapur.017G043100**	Myb family transcription factor APL	Myb_CC_LHEQLE,Myb_DNA-binding
**Sapur.017G060700**	transcription	Myb_DNA-binding
**Sapur.017G068500**	Myb-related protein	Myb_DNA-binding
**Sapur.017G070500**	transcription factor	Myb_DNA-binding
**Sapur.017G070500**	transcription factor	Myb_DNA-binding
**Sapur.017G071300**	transcription factor	Myb_DNA-binding
**Sapur.017G104000**	Transcription factor	Myb_DNA-binding
**Sapur.017G104200**	Transcription factor	Myb_DNA-binding
**Sapur.017G104300**	Transcription factor	Myb_DNA-binding
**Sapur.018G045600**	Myb SANT-like DNA-binding domain protein	Myb_DNA-bind_3
**Sapur.018G059300**	Myb/SANT-like DNA-binding domain	Myb_DNA-bind_3
**Sapur.018G059800**	Myb SANT-like DNA-binding domain protein	Myb_DNA-bind_3
**Sapur.018G070600**	two-component response regulator	Myb_DNA-binding,Response_reg
**Sapur.018G071300**	transcription factor	Myb_DNA-binding
**Sapur.018G085000**	two-component response regulator	Myb_DNA-binding,Response_reg
**Sapur.018G097300**	Synaptotagmin-5-like	C2,SMP_LBD
**Sapur.018G097300**	Synaptotagmin-5-like	C2,SMP_LBD
**Sapur.019G004500**	Myb-like DNA-binding domain	Myb_DNA-binding
**Sapur.019G004800**	Myb-like DNA-binding domain	Myb_DNA-binding
**Sapur.019G005300**	Myb-like DNA-binding domain	Myb_DNA-binding
**Sapur.019G006100**	Myb-like DNA-binding domain	Myb_DNA-binding
**Sapur.019G020500**	Trihelix transcription factor	Myb_DNA-bind_4
**Sapur.019G020500**	Trihelix transcription factor	Myb_DNA-bind_4
**Sapur.019G052500**	Myb SANT-like DNA-binding domain protein	Myb_DNA-bind_3
**Sapur.019G105300**	Myb-related protein	Myb_DNA-binding
**Sapur.019G105400**	Myb-related protein	Myb_DNA-binding

**Sapur.019G105500**	Myb-related protein Myb4-like	Myb_DNA-binding
**Sapur.15WG002300**	Nascent polypeptide-associated complex subunit alpha-like protein	NAC
**Sapur.15WG017100**	transcription factor	Myb_DNA-binding
**Sapur.15WG024100**	transcription, DNA-templated	Myb_DNA-binding
**Sapur.15WG025100**	Transcription repressor KAN1-like	Myb_DNA-binding
**Sapur.15WG026400**	SANT SWI3, ADA2, N-CoR and TFIIIB'' DNA-binding domains	Myb_DNA-binding
**Sapur.15WG038700**	PLATZ transcription factor family protein	PLATZ
**Sapur.15WG038800**	PLATZ transcription factor family protein	PLATZ
**Sapur.15WG038900**	PLATZ transcription factor family protein	PLATZ
**Sapur.15WG039000**	PLATZ transcription factor family protein	PLATZ
**Sapur.15WG039100**	PLATZ transcription factor family protein	PLATZ
**Sapur.15WG039200**	PLATZ transcription factor family protein	PLATZ
**Sapur.15WG039300**	PLATZ transcription factor family protein	PLATZ
**Sapur.15WG039400**	PLATZ transcription factor family protein	PLATZ
**Sapur.15WG039500**	PLATZ transcription factor family protein	PLATZ
**Sapur.15WG039600**	PLATZ transcription factor family protein	PLATZ
**Sapur.15WG039700**	PLATZ transcription factor family protein	PLATZ
**Sapur.15WG039800**	PLATZ transcription factor family protein	PLATZ
**Sapur.15WG073300**	Myb SANT-like DNA-binding domain protein	Myb_DNA-bind_3
**Sapur.15WG087800**	Transcription factor	Myb_DNA-binding
**Sapur.15WG088100**	transcription factor	Myb_DNA-binding
**Sapur.15WG08810**	transcription factor	Myb_DNA-binding

**0**		
**Sapur.15WG095000**	Transcription factor WER-like	Myb_DNA-binding
**Sapur.15WG095100**	Transcription factor WER-like	Myb_DNA-binding
**Sapur.15WG097000**	Transcription factor MYB98-like	Myb_DNA-binding
**Sapur.15WG100700**	transcription factor	Myb_DNA-binding
**Sapur.15WG111100**	Zn-dependent metallo-hydrolase RNA specificity domain	Lactamase_B,Lactamase_B_2, Myb_DNA-bind_4,RMMBL
**Sapur.15WG111100**	Zn-dependent metallo-hydrolase RNA specificity domain	Lactamase_B,Lactamase_B_2, Myb_DNA-bind_4,RMMBL
**Sapur.15WG111100**	Zn-dependent metallo-hydrolase RNA specificity domain	Lactamase_B,Lactamase_B_2, Myb_DNA-bind_4,RMMBL
**Sapur.15WG116900**	SWI SNF complex subunit	Myb_DNA-binding,SURF2,SWIRM,SWIRM-assoc_1
**Sapur.15WG116900**	SWI SNF complex subunit	Myb_DNA-binding,SURF2,SWIRM,SWIRM-assoc_1
**Sapur.15WG121400**	Myb/SANT-like DNA-binding domain	Myb_DNA-bind_4
**Sapur.15WG129800**	Myb/SANT-like DNA-binding domain	Myb_DNA-bind_4
**Sapur.15WG129800**	Myb/SANT-like DNA-binding domain	Myb_DNA-bind_4
**Sapur.15WG129800**	Myb/SANT-like DNA-binding domain	Myb_DNA-bind_4
**Sapur.15WG129800**	transcription factor	Myb_DNA-bind_4
**Sapur.15WG129800**	transcription factor	Myb_DNA-bind_4
**Sapur.15WG138100**	Myb-like DNA-binding domain	Myb_DNA-binding
**Sapur.15WG149300**	Transcription factor	Myb_DNA-binding
**Sapur.15WG152800**	SWI SNF complex subunit	Myb_DNA-binding,SWIRM,SWIRM-assoc_1
**Sapur.15ZG077600**	Transcription factor WER-like	Myb_DNA-binding
**Sapur.15ZG077700**	Transcription factor WER-like	Myb_DNA-binding
**Sapur.15ZG093500**	Zn-dependent metallo-hydrolase RNA specificity domain	Lactamase_B,Lactamase_B_2, Myb_DNA-bind_4,RMMBL
**Sapur.15ZG09350**	Zn-dependent metallo-hydrolase	Lactamase_B,Lactamase_B_2,

**0**	RNA specificity domain	Myb_DNA-bind_4,RMMBL
**Sapur.15ZG093500**	Zn-dependent metallo-hydrolase RNA specificity domain	Lactamase_B,Lactamase_B_2, Myb_DNA-bind_4,RMMBL
**Sapur.15ZG103800**	Myb/SANT-like DNA-binding domain	Myb_DNA-bind_4
**Sapur.15ZG112000**	Myb/SANT-like DNA-binding domain	Myb_DNA-bind_4
**Sapur.15ZG112000**	Myb/SANT-like DNA-binding domain	Myb_DNA-bind_4
**Sapur.15ZG112000**	Myb/SANT-like DNA-binding domain	Myb_DNA-bind_4
**Sapur.15ZG112000**	transcription factor	Myb_DNA-bind_4
**Sapur.15ZG112000**	transcription factor	Myb_DNA-bind_4
**Sapur.15ZG131500**	Transcription factor	Myb_DNA-binding
**Sapur.T023200**	Protein of unknown function (DUF3755)	DUF3755,Myb_DNA-binding
**Sapur.T033300**	PLATZ transcription factor family protein	PLATZ
**Sapur.T033400**	PLATZ transcription factor family protein	PLATZ
**Sapur.T033500**	PLATZ transcription factor family protein	PLATZ
**Sapur.T033600**	PLATZ transcription factor family protein	PLATZ
**Sapur.T033700**	PLATZ transcription factor family protein	PLATZ
**Sapur.T033800**	PLATZ transcription factor family protein	PLATZ
**Sapur.T033900**	PLATZ transcription factor family protein	PLATZ
**Sapur.T034000**	PLATZ transcription factor family protein	PLATZ
**Sapur.T074000**	Myb SANT-like DNA-binding domain protein	Myb_DNA-bind_3
**Sapur.T076700**	PLATZ transcription factor family protein	PLATZ
**Sapur.T090500**	Myb-related protein	Myb_DNA-binding
**Sapur.T093700**	HSA	HSA,Myb_DNA-bind_6
**Sapur.T093800**	histone H4-K5 acetylation	HSA,Myb_DNA-bind_6
**Sapur.T099900**	histone H4-K5 acetylation	HSA,Myb_DNA-bind_6
**Sapur.T100400**	histone H4-K5 acetylation	HSA,Myb_DNA-bind_6

**Sapur.T100600**	HSA	HSA,Myb_DNA-bind_6
**Sapur.T101000**	HSA	HSA,Myb_DNA-bind_6
**Sapur.T101200**	HSA	HSA,Myb_DNA-bind_6
**Sapur.T101900**	HSA	HSA,Myb_DNA-bind_6
**Sapur.T102800**	HSA	HSA,Myb_DNA-bind_6
**Sapur.T103200**	HSA	HSA,Myb_DNA-bind_6
**Sapur.T112300**	PLATZ transcription factor family protein	PLATZ
**Sapur.T112400**	PLATZ transcription factor family protein	PLATZ
**Sapur.T112500**	PLATZ transcription factor family protein	PLATZ
**Sapur.T112600**	PLATZ transcription factor family protein	PLATZ
**Sapur.T112700**	PLATZ transcription factor family protein	PLATZ
**Sapur.T112800**	PLATZ transcription factor family protein	PLATZ
**Sapur.T112900**	PLATZ transcription factor family protein	PLATZ
**Sapur.T113000**	PLATZ transcription factor family protein	PLATZ
**Sapur.T113100**	PLATZ transcription factor family protein	PLATZ
**Sapur.T113200**	PLATZ transcription factor family protein	PLATZ
**Sapur.T113300**	PLATZ transcription factor family protein	PLATZ
**Sapur.T113400**	PLATZ transcription factor family protein	PLATZ
**Sapur.T113500**	PLATZ transcription factor family protein	PLATZ
**Sapur.T114100**	Myb-like DNA-binding domain	Myb_DNA-binding
**Sapur.T115200**	Myb-like DNA-binding domain	Myb_DNA-binding
**Sapur.T117100**	Myb-like DNA-binding domain	Myb_DNA-binding
**Sapur.T130900**	HSA	HSA,Myb_DNA-bind_6
**Sapur.T140200**	Myb-like DNA-binding domain	Myb_DNA-binding
**Sapur.T140600**	HSA	HSA,Myb_DNA-bind_6
**Sapur.T141600**	HSA	HSA,Myb_DNA-bind_6
**Sapur.T144400**	RNA polymerase II transcription regulator recruiting activity	Myb_DNA-binding
**Sapur.T148000**	transcription	Myb_DNA-binding
**Sapur.T148600**	SANT SWI3, ADA2, N-CoR and TFIIIB" DNA-binding	Myb_DNA-binding

	domains	
**Sapur.T152400**	HSA	HSA,Myb_DNA-bind_6
**Sapur.T159200**	HSA	HSA,Myb_DNA-bind_6
**Sapur.T164300**	HSA	HSA,Myb_DNA-bind_6
**Sapur.T166500**	HSA	HSA,Myb_DNA-bind_6
**Sapur.T168300**	PLATZ transcription factor family protein	PLATZ
**Sapur.T168300**	PLATZ transcription factor family protein	PLATZ
**Sapur.T170600**	HSA	HSA,Myb_DNA-bind_6
**Sapur.T174100**	HSA	HSA,Myb_DNA-bind_6
**Sapur.T174200**	HSA	HSA,Myb_DNA-bind_6
**Sapur.T174200**	HSA	HSA,Myb_DNA-bind_6
**Sapur.T175800**	HSA	HSA,Myb_DNA-bind_6
**Sapur.T176000**	histone H4-K5 acetylation	HSA,Myb_DNA-bind_6
**Sapur.T176800**	HSA	HSA,Myb_DNA-bind_6
**Sapur.T179700**	HSA	HSA,Myb_DNA-bind_6
**Sapur.T180500**	HSA	HSA,Myb_DNA-bind_6
**Sapur.T182600**	HSA	HSA,Myb_DNA-bind_6
**Sapur.T182700**	histone H4-K5 acetylation	HSA,Myb_DNA-bind_6
**Sapur.T188300**	HSA	HSA,Myb_DNA-bind_6
**Sapur.T188400**	HSA	HSA,Myb_DNA-bind_6
**Sapur.T188500**	histone H4-K5 acetylation	HSA,Myb_DNA-bind_6
**Sapur.T188900**	HSA	HSA,Myb_DNA-bind_6
**Sapur.T192400**	Myb-like DNA-binding domain	Myb_DNA-binding
