# Supplementary material for: Insights into Molecular Mechanism of Secondary Xylem Rapid Growth in Salix psammophila
Source: Plants (Basel). 2025 Feb 5;14(3):459. doi: 10.3390/plants14030459 (PMC11819810; doi:10.3390/plants14030459)
Supplement: Supplementary file 1 [file plants-14-00459-s001.zip › Supplementary Table/Table S12.pdf]

Table S12 Genes regulating green module-related transcription factors.

X	Description	PFAMs
**Sapur.004G061200**	ODORANT1-like	Myb_DNA-binding
**Sapur.006G128000**	Trihelix transcription factor	Myb_DNA-bind_4
**Sapur.007G022600**	SANT SWI3, ADA2, N-CoR and TFIIIB'' DNA-binding domains	Myb_DNA-binding
**Sapur.007G119500**	Protein of unknown function (DUF3755)	DUF3755,Myb_DNA-binding
**Sapur.009G016800**	Homeobox-leucine zipper protein	HALZ,HD-ZIP_N,Homeobox
**Sapur.014G022100**	HSA	HSA,Myb_DNA-bind_6
**Sapur.014G022100**	histone H4-K5 acetylation	HSA,Myb_DNA-bind_6
**Sapur.016G106500**	Myb-like DNA-binding domain	Myb_DNA-binding
**Sapur.016G106500**	Myb-like DNA-binding domain	Myb_DNA-binding
**Sapur.016G123300**	Transcription factor	Myb_CC_LHEQLE,Myb_DNA-binding
**Sapur.017G103800**	Transcription factor	Myb_DNA-binding
**Sapur.017G103900**	Transcription factor	Myb_DNA-binding
**Sapur.017G104100**	Transcription factor	Myb_DNA-binding
**Sapur.15WG023500**	Nascent polypeptide-associated complex subunit beta	NAC
**Sapur.T100000**	HSA	HSA,Myb_DNA-bind_6
**Sapur.T188000**	HSA	HSA,Myb_DNA-bind_6
