# Supplementary material for: Insights into Molecular Mechanism of Secondary Xylem Rapid Growth in Salix psammophila
Source: Plants (Basel). 2025 Feb 5;14(3):459. doi: 10.3390/plants14030459 (PMC11819810; doi:10.3390/plants14030459)
Supplement: Supplementary file 1 [file plants-14-00459-s001.zip › Supplementary Table/Table S2 .pdf]

Table S2 Mapping of RNA-Seq data of secondary xylem and phloem of *Salix psammophila* with reference genome.

	Total_reads	Mapped_reads	Unmapped_reads	Mapped_ratio	Uniq_reads	Uniq_ratio	Multiple_reads	Multiple_ratio
**1a_m_1**	24533817	22341782	2192035	91.07%	13957473	56.89%	8384309	34.17%
**1a_m_2**	20815194	18255715.5	2559478.5	87.70%	16725755	80.35%	1529960.5	7.35%
**1a_m_3**	19984762	17743305	2241457	88.78%	13037550.5	65.24%	4705754.5	23.55%
**1a_r_1**	23628646	20906390	2722256	88.48%	18568648.5	78.59%	2337741.5	9.89%
**1a_r_2**	19216123	16984984	2231139	88.39%	14605045.5	76.00%	2379938.5	12.39%
**1a_r_3**	25471953	22771456	2700497	89.40%	19304511.5	75.79%	3466944.5	13.61%
**2a_m_1**	22515396	19821122.5	2694273.5	88.03%	17926539.5	79.62%	1894583	8.41%
**2a_m_2**	33249931	29948911.5	3301019.5	90.07%	17701918.5	53.24%	12246993	36.83%
**2a_m_3**	31896624	27547897	4348727	86.37%	25632030	80.36%	1915867	6.01%
**2a_r_1**	21991046	19384931	2606115	88.15%	17266955.5	78.52%	2117975.5	9.63%
**2a_r_2**	22672913	20238238.5	2434674.5	89.26%	17247395	76.07%	2990843.5	13.19%
**2a_r_3**	21884269	19270457	2613812	88.06%	17002094	77.69%	2268363	10.37%
**3a_m_1**	19595220	16961940.5	2633279.5	86.56%	15867060	80.97%	1094880.5	5.59%
**3a_m_2**	35956810	31186819.5	4769990.5	86.73%	28086981	78.11%	3099838.5	8.62%
**3a_m_3**	19465171	17212771.5	2252399.5	88.43%	14984071	76.98%	2228700.5	11.45%
**3a_r_1**	23489417	20675498.5	2813918.5	88.02%	18610028	79.23%	2065470.5	8.79%
**3a_r_2**	21218887	18742239.5	2476647.5	88.33%	16126232.5	76.00%	2616007	12.33%
**3a_r_3**	24002981	21315376	2687605	88.80%	17514852	72.97%	3800524	15.83%
