# Supplementary material for: Insights into Molecular Mechanism of Secondary Xylem Rapid Growth in Salix psammophila
Source: Plants (Basel). 2025 Feb 5;14(3):459. doi: 10.3390/plants14030459 (PMC11819810; doi:10.3390/plants14030459)
Supplement: Supplementary file 1 [file plants-14-00459-s001.zip › Supplementary Table/Table S3 .pdf]

Table S3 Comparison of xylem-specific differentially expressed genes GO annotation in 1, and 2-year-old.

	GOBPID	Count	Size	Term	gene_id
**m_down_BP**	GO:0006468	39	1578	protein phosphorylation	Sapur.002G195600 Sapur.003G018500 Sapur.003G139700 Sapur.003G160700 Sapur.004G016000 Sapur.004G032900 Sapur.004G040600 Sapur.004G067800 Sapur.004G157600 Sapur.005G124000 Sapur.005G124100 Sapur.005G188000 Sapur.006G044000 Sapur.006G096300 Sapur.006G108800 Sapur.007G029800 Sapur.007G114500 Sapur.009G005200 Sapur.009G116000 Sapur.011G002400 Sapur.011G057400 Sapur.011G065900 Sapur.011G093300 Sapur.011G103400 Sapur.011G103500 Sapur.011G109100 Sapur.012G067300 Sapur.012G098500 Sapur.013G035400 Sapur.014G039900 Sapur.016G222400 Sapur.016G263500 Sapur.017G080200 Sapur.019G013400 Sapur.15WG028200 Sapur.15ZG018000 Sapur.15ZG027900 Sapur.15ZG061200 Sapur.15ZG124400
	GO:0016310	39	1593	phosphorylation	Sapur.002G195600 Sapur.003G018500 Sapur.003G139700 Sapur.003G160700 Sapur.004G016000 Sapur.004G032900 Sapur.004G040600 Sapur.004G067800 Sapur.004G157600 Sapur.005G124000 Sapur.005G124100 Sapur.005G188000 Sapur.006G044000 Sapur.006G096300 Sapur.006G108800 Sapur.007G029800 Sapur.007G114500 Sapur.009G005200 Sapur.009G116000 Sapur.011G002400 Sapur.011G057400 Sapur.011G065900 Sapur.011G093300 Sapur.011G103400 Sapur.011G103500 Sapur.011G109100 Sapur.012G067300 Sapur.012G098500 Sapur.013G035400 Sapur.014G039900 Sapur.016G222400 Sapur.016G263500 Sapur.017G080200 Sapur.019G013400 Sapur.15WG028200 Sapur.15ZG018000 Sapur.15ZG027900 Sapur.15ZG061200 Sapur.15ZG124400
	GO:0046034	3	31	ATP metabolic process	Sapur.004G008500 Sapur.012G055400 Sapur.018G045400
	GO:0006508	15	483	proteolysis	Sapur.001G051400 Sapur.002G013000 Sapur.002G101500 Sapur.002G143900 Sapur.004G121400 Sapur.005G161200 Sapur.006G026000 Sapur.009G106200 Sapur.012G067700 Sapur.013G004300 Sapur.014G009700 Sapur.016G002200 Sapur.018G010400 Sapur.018G084700 Sapur.15ZG068300
	GO:0009199	3	37	ribonucleoside triphosphate metabolic process	Sapur.004G008500 Sapur.012G055400 Sapur.018G045400

GO:0009141	3	37	nucleoside triphosphate metabolic process	Sapur.004G008500 Sapur.012G055400 Sapur.018G045400
GO:0009144	3	37	purine nucleoside triphosphate metabolic process	Sapur.004G008500 Sapur.012G055400 Sapur.018G045400
GO:0009205	3	37	purine ribonucleoside triphosphate metabolic process	Sapur.004G008500 Sapur.012G055400 Sapur.018G045400
GO:0006796	44	1954	phosphate-containing compound metabolic process	Sapur.002G195600 Sapur.003G018500 Sapur.003G139700 Sapur.003G160700 Sapur.004G008500 Sapur.004G016000 Sapur.004G032900 Sapur.004G040600 Sapur.004G067800 Sapur.004G157600 Sapur.005G124000 Sapur.005G124100 Sapur.005G188000 Sapur.006G044000 Sapur.006G096300 Sapur.006G108800 Sapur.007G029800 Sapur.007G114500 Sapur.009G005200 Sapur.009G116000 Sapur.011G002400 Sapur.011G057400 Sapur.011G065900 Sapur.011G093300 Sapur.011G103400 Sapur.011G103500 Sapur.011G109100 Sapur.012G055400 Sapur.012G067300 Sapur.012G098500 Sapur.013G035400 Sapur.014G039900 Sapur.016G222400 Sapur.016G263500 Sapur.017G080200 Sapur.018G045400 Sapur.019G013400 Sapur.15WG028200 Sapur.15ZG015100 Sapur.15ZG018000 Sapur.15ZG027900 Sapur.15ZG061200 Sapur.15ZG124400 Sapur.001G057600
GO:0006793	44	1955	phosphorus metabolic process	Sapur.002G195600 Sapur.003G018500 Sapur.003G139700 Sapur.003G160700 Sapur.004G008500 Sapur.004G016000 Sapur.004G032900 Sapur.004G040600 Sapur.004G067800 Sapur.004G157600 Sapur.005G124000 Sapur.005G124100 Sapur.005G188000 Sapur.006G044000 Sapur.006G096300 Sapur.006G108800 Sapur.007G029800 Sapur.007G114500 Sapur.009G005200 Sapur.009G116000 Sapur.011G002400 Sapur.011G057400 Sapur.011G065900 Sapur.011G093300 Sapur.011G103400 Sapur.011G103500 Sapur.011G109100 Sapur.012G055400 Sapur.012G067300 Sapur.012G098500 Sapur.013G035400 Sapur.014G039900 Sapur.016G222400 Sapur.016G263500 Sapur.017G080200 Sapur.018G045400 Sapur.019G013400 Sapur.15WG028200 Sapur.15ZG015100 Sapur.15ZG018000 Sapur.15ZG027900 Sapur.15ZG061200 Sapur.15ZG124400 Sapur.001G057600

m_up_BP	GO:0006817	1	2	phosphate ion transport	Sapur.010G024300
	GO:0005985	2	16	sucrose metabolic process	Sapur.004G057100 Sapur.018G021500
	GO:0005984	2	19	disaccharide metabolic process	Sapur.004G057100 Sapur.018G021500
	GO:0009311	2	20	oligosaccharide metabolic process	Sapur.004G057100 Sapur.018G021500
	GO:0030259	1	5	lipid glycosylation	Sapur.005G152300
	GO:0006952	2	53	defense response	Sapur.004G012400 Sapur.011G086500
m_down_CC	GO:0016469	3	42	proton-transporting two-sector ATPase complex	Sapur.004G008500 Sapur.012G055400 Sapur.018G045400
	GO:0045259	2	22	proton-transporting ATP synthase complex	Sapur.004G008500 Sapur.012G055400
	GO:0033178	2	23	proton-transporting two-sector ATPase complex, catalytic domain	Sapur.004G008500 Sapur.018G045400
	GO:0016020	24	1182	membrane	Sapur.001G022700 Sapur.001G143100 Sapur.004G040800 Sapur.005G048900 Sapur.006G084000 Sapur.006G100000 Sapur.008G029700 Sapur.008G138300 Sapur.009G102200 Sapur.010G007300 Sapur.010G024300 Sapur.010G033800 Sapur.010G124000 Sapur.010G167900 Sapur.011G062200 Sapur.011G108900 Sapur.012G051600 Sapur.013G107200 Sapur.016G084700 Sapur.016G223600 Sapur.15ZG074200 Sapur.004G008500 Sapur.012G055400 Sapur.018G045400
m_up_CC	GO:0005884	1	2	actin filament	Sapur.15WG079100
	GO:0005674	1	3	transcription factor TFIIF complex	Sapur.010G175200
	GO:0015935	1	6	small ribosomal subunit	Sapur.013G097900
m_down_MF	GO:0017171	9	153	serine hydrolase activity	Sapur.002G013000 Sapur.002G101500 Sapur.004G121400 Sapur.009G106200 Sapur.013G004300 Sapur.014G009700 Sapur.018G084700 Sapur.001G051400 Sapur.006G026000
	GO:0008236	9	153	serine-type peptidase activity	Sapur.002G013000 Sapur.002G101500 Sapur.004G121400 Sapur.009G106200 Sapur.013G004300 Sapur.014G009700 Sapur.018G084700 Sapur.001G051400

GO:0004252	6	92	serine-type endopeptidase activity	Sapur.006G026000 Sapur.002G013000 Sapur.002G101500 Sapur.004G121400 Sapur.009G106200 Sapur.013G004300 Sapur.014G009700
GO:0016798	14	379	hydrolase activity, acting on glycosyl bonds	Sapur.001G171300 Sapur.002G192300 Sapur.003G024100 Sapur.006G101100 Sapur.008G043800 Sapur.008G107800 Sapur.010G082200 Sapur.010G124000 Sapur.010G137900 Sapur.014G071900 Sapur.014G097700 Sapur.014G113800 Sapur.019G068900 Sapur.019G109000
GO:0005215	22	729	transporter activity	Sapur.001G022700 Sapur.004G008500 Sapur.004G040800 Sapur.006G084000 Sapur.006G100000 Sapur.008G029700 Sapur.008G138300 Sapur.009G090000 Sapur.009G102200 Sapur.010G007300 Sapur.010G024300 Sapur.010G033800 Sapur.010G167900 Sapur.011G028400 Sapur.011G108900 Sapur.012G051600 Sapur.013G107200 Sapur.016G084700 Sapur.016G223600 Sapur.017G067400 Sapur.15ZG074200 Sapur.012G055400
GO:0004553	13	357	hydrolase activity, hydrolyzing O-glycosyl compounds	Sapur.001G171300 Sapur.002G192300 Sapur.003G024100 Sapur.006G101100 Sapur.008G043800 Sapur.008G107800 Sapur.010G082200 Sapur.010G137900 Sapur.014G071900 Sapur.014G097700 Sapur.014G113800 Sapur.019G068900 Sapur.019G109000
GO:0004672	39	1565	protein kinase activity	Sapur.002G195600 Sapur.003G018500 Sapur.003G139700 Sapur.003G160700 Sapur.004G016000 Sapur.004G032900 Sapur.004G040600 Sapur.004G067800 Sapur.004G157600 Sapur.005G124000 Sapur.005G124100 Sapur.005G188000 Sapur.006G044000 Sapur.006G096300 Sapur.006G108800 Sapur.007G029800 Sapur.007G114500 Sapur.009G005200 Sapur.009G116000 Sapur.011G002400 Sapur.011G057400 Sapur.011G065900 Sapur.011G093300 Sapur.011G103400 Sapur.011G103500 Sapur.011G109100 Sapur.012G067300 Sapur.012G098500 Sapur.013G035400 Sapur.014G039900 Sapur.016G222400 Sapur.016G263500 Sapur.017G080200 Sapur.019G013400 Sapur.15WG028200 Sapur.15ZG018000 Sapur.15ZG027900 Sapur.15ZG061200 Sapur.15ZG124400
GO:0005507	5	85	copper ion binding	Sapur.001G098800 Sapur.009G080300 Sapur.010G060200 Sapur.014G079000 Sapur.016G116800
GO:0016301	40	1669	kinase activity	Sapur.002G195600 Sapur.003G018500 Sapur.003G139700 Sapur.003G160700

				Sapur.004G016000 Sapur.004G032900 Sapur.004G040600 Sapur.004G067800 Sapur.004G157600 Sapur.005G124000 Sapur.005G124100 Sapur.005G188000 Sapur.006G044000 Sapur.006G096300 Sapur.006G108800 Sapur.007G029800 Sapur.007G114500 Sapur.009G005200 Sapur.009G116000 Sapur.011G002400 Sapur.011G057400 Sapur.011G065900 Sapur.011G093300 Sapur.011G103400 Sapur.011G103500 Sapur.011G109100 Sapur.012G067300 Sapur.012G098500 Sapur.013G035400 Sapur.014G039900 Sapur.016G222400 Sapur.016G263500 Sapur.017G080200 Sapur.019G013400 Sapur.15WG028200 Sapur.15ZG018000 Sapur.15ZG027900 Sapur.15ZG061200 Sapur.15ZG124400 Sapur.014G132000
GO:0016773	40	1675	phosphotransferase activity, alcohol group as acceptor	Sapur.002G195600 Sapur.003G018500 Sapur.003G139700 Sapur.003G160700 Sapur.004G016000 Sapur.004G032900 Sapur.004G040600 Sapur.004G067800 Sapur.004G157600 Sapur.005G124000 Sapur.005G124100 Sapur.005G188000 Sapur.006G044000 Sapur.006G096300 Sapur.006G108800 Sapur.007G029800 Sapur.007G114500 Sapur.009G005200 Sapur.009G116000 Sapur.011G002400 Sapur.011G057400 Sapur.011G065900 Sapur.011G093300 Sapur.011G103400 Sapur.011G103500 Sapur.011G109100 Sapur.012G067300 Sapur.012G098500 Sapur.013G035400 Sapur.014G039900 Sapur.016G222400 Sapur.016G263500 Sapur.017G080200 Sapur.019G013400 Sapur.15WG028200 Sapur.15ZG018000 Sapur.15ZG027900 Sapur.15ZG061200 Sapur.15ZG124400 Sapur.009G066500
GO:0008233	14	445	peptidase activity	Sapur.002G013000 Sapur.002G101500 Sapur.002G143900 Sapur.004G121400 Sapur.005G161200 Sapur.009G106200 Sapur.013G004300 Sapur.014G009700 Sapur.016G002200 Sapur.018G010400 Sapur.018G084700 Sapur.001G051400 Sapur.006G026000 Sapur.012G067700
GO:0140096	55	2472	catalytic activity, acting on a protein	Sapur.002G013000 Sapur.002G101500 Sapur.002G143900 Sapur.002G195600 Sapur.003G018500 Sapur.003G139700 Sapur.003G160700 Sapur.004G016000 Sapur.004G032900 Sapur.004G040600 Sapur.004G067800 Sapur.004G121400 Sapur.004G157600 Sapur.005G124000 Sapur.005G124100 Sapur.005G161200 Sapur.005G188000 Sapur.006G044000 Sapur.006G096300 Sapur.006G108800 Sapur.007G029800 Sapur.007G114500 Sapur.008G116700 Sapur.009G005200 Sapur.009G106200 Sapur.009G116000 Sapur.011G002400 Sapur.011G057400

				Sapur.011G065900 Sapur.011G093300 Sapur.011G103400 Sapur.011G103500 Sapur.011G109100 Sapur.012G067300 Sapur.012G098500 Sapur.013G004300 Sapur.013G035400 Sapur.014G009700 Sapur.014G039900 Sapur.016G002200 Sapur.016G222400 Sapur.016G263500 Sapur.017G080200 Sapur.018G010400 Sapur.018G084700 Sapur.019G013400 Sapur.15WG028200 Sapur.15ZG018000 Sapur.15ZG027900 Sapur.15ZG061200 Sapur.15ZG124400 Sapur.001G051400 Sapur.006G026000 Sapur.012G067700 Sapur.15ZG015100
GO:0004175	10	290	endopeptidase activity	Sapur.002G013000 Sapur.002G101500 Sapur.002G143900 Sapur.004G121400 Sapur.005G161200 Sapur.009G106200 Sapur.013G004300 Sapur.014G009700 Sapur.016G002200 Sapur.018G010400
GO:0004857	7	176	enzyme inhibitor activity	Sapur.001G089400 Sapur.001G135400 Sapur.003G041900 Sapur.003G078700 Sapur.005G047000 Sapur.010G082300 Sapur.15ZG119700
GO:0140678	7	176	molecular function inhibitor activity	Sapur.001G089400 Sapur.001G135400 Sapur.003G041900 Sapur.003G078700 Sapur.005G047000 Sapur.010G082300 Sapur.15ZG119700
GO:0005315	1	2	inorganic phosphate transmembrane transporter activity	Sapur.010G024300
GO:0016787	43	1881	hydrolase activity	Sapur.001G143100 Sapur.002G013000 Sapur.002G101500 Sapur.002G143900 Sapur.004G121400 Sapur.005G048900 Sapur.005G161200 Sapur.009G106200 Sapur.011G028400 Sapur.013G004300 Sapur.013G110800 Sapur.014G009700 Sapur.016G002200 Sapur.017G067400 Sapur.018G010400 Sapur.018G084700 Sapur.001G051400 Sapur.001G057600 Sapur.001G171300 Sapur.002G192300 Sapur.002G197100 Sapur.003G024100 Sapur.006G026000 Sapur.006G101100 Sapur.007G091900 Sapur.008G043800 Sapur.008G054000 Sapur.008G107800 Sapur.008G112800 Sapur.010G082200 Sapur.010G124000 Sapur.010G137900 Sapur.010G151600 Sapur.012G067700 Sapur.013G000800 Sapur.014G071900 Sapur.014G097700 Sapur.014G113800 Sapur.019G068900 Sapur.019G109000 Sapur.15WG032400 Sapur.15ZG015100 Sapur.15ZG097200
GO:0043531	11	351	ADP binding	Sapur.005G024800 Sapur.012G086700 Sapur.012G091800 Sapur.014G001700 Sapur.017G018700 Sapur.017G113400 Sapur.017G123300 Sapur.018G016600

					Sapur.019G023100 Sapur.019G036300 Sapur.019G092800
	GO:0016854	1	3	racemase and epimerase activity	Sapur.15ZG060400
	GO:0016857	1	3	racemase and epimerase activity, acting on carbohydrates and derivatives	Sapur.15ZG060400
	GO:0004719	1	3	protein-L-isoaspartate (D-aspartate) O-methyltransferase activity	Sapur.15ZG086800
	GO:0046906	7	454	tetrapyrrole binding	Sapur.001G089000 Sapur.001G093200 Sapur.001G097300 Sapur.007G001800 Sapur.007G047200 Sapur.014G018000 Sapur.019G000600
	GO:0020037	7	454	heme binding	Sapur.001G089000 Sapur.001G093200 Sapur.001G097300 Sapur.007G001800 Sapur.007G047200 Sapur.014G018000 Sapur.019G000600
**m_up_MF**	GO:0016705	6	354	oxidoreductase activity, acting on paired donors, with incorporation or reduction of molecular oxygen	Sapur.001G089000 Sapur.001G093200 Sapur.001G097300 Sapur.007G001800 Sapur.007G047200 Sapur.014G018000
	GO:0010340	1	4	carboxyl-O-methyltransferase activity	Sapur.15ZG086800
	GO:0051998	1	4	protein carboxyl O-methyltransferase activity	Sapur.15ZG086800
	GO:0008276	1	4	protein methyltransferase activity	Sapur.15ZG086800
	GO:0005506	6	373	iron ion binding	Sapur.001G089000 Sapur.001G093200 Sapur.001G097300 Sapur.007G001800 Sapur.007G047200 Sapur.014G018000
	GO:0003950	1	8	NAD+ ADP-ribosyltransferase activity	Sapur.15ZG078700
