# Supplementary material for: Insights into Molecular Mechanism of Secondary Xylem Rapid Growth in Salix psammophila
Source: Plants (Basel). 2025 Feb 5;14(3):459. doi: 10.3390/plants14030459 (PMC11819810; doi:10.3390/plants14030459)
Supplement: Supplementary file 1 [file plants-14-00459-s001.zip › Supplementary Table/Table S4 .pdf]

Table S4 Comparison of xylem-specific differentially expressed genes GO annotation in 2, and 3-year-old.

	GOBPID	Count	Size	Term	gene_id
m_down_BP	GO:0051179	329	1416	localization	Sapur.001G016200 Sapur.001G027700 Sapur.001G028900 Sapur.001G033600 Sapur.001G037600 Sapur.001G037800 Sapur.001G040600 Sapur.001G046900 Sapur.001G079000 Sapur.001G082900 Sapur.001G110400 Sapur.001G132900 Sapur.001G138400 Sapur.001G147300 Sapur.001G154800 Sapur.001G164900 Sapur.001G169300 Sapur.001G174500 Sapur.001G185600 Sapur.001G187000 Sapur.002G011100 Sapur.002G013700 Sapur.002G016500 Sapur.002G017000 Sapur.002G038200 Sapur.002G046500 Sapur.002G053800 Sapur.002G056800 Sapur.002G067400 Sapur.002G068100 Sapur.002G086000 Sapur.002G098200 Sapur.002G110800 Sapur.002G116800 Sapur.002G118400 Sapur.002G133400 Sapur.002G138300 Sapur.002G155000 Sapur.002G177900 Sapur.002G186300 Sapur.002G190100 Sapur.002G193600 Sapur.003G005100 Sapur.003G010700 Sapur.003G014800 Sapur.003G021100 Sapur.003G029600 Sapur.003G029700 Sapur.003G039800 Sapur.003G061600 Sapur.003G065900 Sapur.003G074700 Sapur.003G076900 Sapur.003G082500 Sapur.003G090300 Sapur.003G092800 Sapur.003G094300 Sapur.003G096000 Sapur.003G100300 Sapur.003G107900 Sapur.003G127800 Sapur.003G134200 Sapur.003G137200 Sapur.003G138600 Sapur.003G139000 Sapur.004G005200 Sapur.004G020800 Sapur.004G021600 Sapur.004G023500 Sapur.004G025800 Sapur.004G026500 Sapur.004G034200 Sapur.004G048700 Sapur.004G050600 Sapur.004G070600 Sapur.004G076400 Sapur.004G099600 Sapur.004G115300 Sapur.004G126600 Sapur.004G131800 Sapur.004G132700 Sapur.004G141700 Sapur.004G155400 Sapur.004G162900 Sapur.004G168100 Sapur.004G168900 Sapur.004G171500 Sapur.004G172600 Sapur.005G031200 Sapur.005G031500 Sapur.005G034100 Sapur.005G051300 Sapur.005G058800 Sapur.005G080800 Sapur.005G121300 Sapur.005G138700 Sapur.005G160500 Sapur.005G172700 Sapur.005G174600 Sapur.005G180700 Sapur.005G191500 Sapur.005G204400 Sapur.006G001200 Sapur.006G004700 Sapur.006G009900 Sapur.006G015100 Sapur.006G015300 Sapur.006G023800 Sapur.006G034400 Sapur.006G037500 Sapur.006G049300 Sapur.006G059100 Sapur.006G062900 Sapur.006G076200 Sapur.006G084000 Sapur.006G102100 Sapur.006G102500 Sapur.006G105100 Sapur.006G105200 Sapur.006G123300 Sapur.006G150200 Sapur.006G153100 Sapur.006G167000 Sapur.006G186200 Sapur.006G190100 Sapur.006G198200 Sapur.006G201300 Sapur.006G223800 Sapur.007G021700 Sapur.007G023000 Sapur.007G027700 Sapur.007G051300 Sapur.007G111000 Sapur.008G005900 Sapur.008G015200 Sapur.008G025200 Sapur.008G038400 Sapur.008G050300 Sapur.008G071600 Sapur.008G076300 Sapur.008G104200 Sapur.008G105500 Sapur.008G108600 Sapur.008G113200 Sapur.008G126700 Sapur.008G142900 Sapur.008G153800 Sapur.008G162700 Sapur.009G003300 Sapur.009G006300 Sapur.009G009400 Sapur.009G036200 Sapur.009G039900 Sapur.009G054300 Sapur.009G057100 Sapur.009G062200 Sapur.009G062300 Sapur.009G063600 Sapur.009G068000 Sapur.009G090000 Sapur.009G091000 Sapur.009G102500 Sapur.009G109000 Sapur.009G109200 Sapur.009G117600 Sapur.009G129500 Sapur.009G131800 Sapur.010G007300 Sapur.010G014600 Sapur.010G048300 Sapur.010G066100 Sapur.010G073400 Sapur.010G081400 Sapur.010G085400 Sapur.010G087800 Sapur.010G090500 Sapur.010G103300 Sapur.010G108800 Sapur.010G127200 Sapur.010G133600

				Sapur.010G141400 Sapur.010G146400 Sapur.010G149500 Sapur.010G150300 Sapur.010G151900 Sapur.010G159200 Sapur.010G165600 Sapur.010G189300 Sapur.010G195700 Sapur.011G006300 Sapur.011G034100 Sapur.011G041500 Sapur.011G050700 Sapur.011G075900 Sapur.011G101600 Sapur.011G106700 Sapur.011G107600 Sapur.011G113700 Sapur.011G115500 Sapur.011G121700 Sapur.012G007100 Sapur.012G009800 Sapur.012G025200 Sapur.012G028100 Sapur.012G030400 Sapur.012G034700 Sapur.012G040200 Sapur.012G043900 Sapur.012G071300 Sapur.012G089500 Sapur.012G100200 Sapur.013G034800 Sapur.013G066200 Sapur.013G066500 Sapur.013G068000 Sapur.013G080100 Sapur.013G090600 Sapur.013G100800 Sapur.013G101200 Sapur.013G105800 Sapur.013G109100 Sapur.013G109400 Sapur.013G128500 Sapur.014G044700 Sapur.014G048500 Sapur.014G050800 Sapur.014G060800 Sapur.014G083800 Sapur.014G089900 Sapur.014G092800 Sapur.014G099300 Sapur.014G102800 Sapur.014G104400 Sapur.014G105600 Sapur.014G113300 Sapur.014G123000 Sapur.014G123600 Sapur.014G138800 Sapur.016G040900 Sapur.016G047900 Sapur.016G065300 Sapur.016G096400 Sapur.016G097500 Sapur.016G098500 Sapur.016G102900 Sapur.016G109800 Sapur.016G113000 Sapur.016G138400 Sapur.016G147600 Sapur.016G163500 Sapur.016G175100 Sapur.016G192300 Sapur.016G202100 Sapur.016G203700 Sapur.016G204500 Sapur.016G212300 Sapur.016G220900 Sapur.016G226600 Sapur.016G231500 Sapur.016G231600 Sapur.016G234000 Sapur.016G239800 Sapur.016G242000 Sapur.016G248500 Sapur.016G258100 Sapur.016G276200 Sapur.016G285800 Sapur.016G295000 Sapur.016G295100 Sapur.016G307500 Sapur.016G310000 Sapur.017G027100 Sapur.017G027200 Sapur.017G044400 Sapur.017G044500 Sapur.017G056200 Sapur.017G067400 Sapur.017G071700 Sapur.017G073700 Sapur.017G078400 Sapur.017G095100 Sapur.017G119100 Sapur.018G006400 Sapur.018G026100 Sapur.018G031900 Sapur.018G051100 Sapur.018G060200 Sapur.018G081000 Sapur.018G084500 Sapur.018G089800 Sapur.018G097400 Sapur.018G101700 Sapur.018G118500 Sapur.018G118800 Sapur.019G002100 Sapur.019G002700 Sapur.019G054200 Sapur.019G076600 Sapur.019G082100 Sapur.019G097900 Sapur.019G099600 Sapur.15WG044900 Sapur.15WG076900 Sapur.15WG078100 Sapur.15WG081100 Sapur.15ZG012800 Sapur.15ZG029200 Sapur.15ZG029900 Sapur.15ZG031600 Sapur.15ZG032200 Sapur.15ZG049100 Sapur.15ZG059400 Sapur.15ZG063600 Sapur.15ZG071300 Sapur.15ZG073700 Sapur.15ZG093800 Sapur.15ZG123400 Sapur.15ZG132200 Sapur.T001500 Sapur.T047800 Sapur.T080400 Sapur.T114400 Sapur.010G042500 Sapur.003G108000 Sapur.006G116300 Sapur.006G180200 Sapur.007G049600 Sapur.010G079100 Sapur.15ZG006400
GO:0051234	323	1407	establishment of localization	Sapur.001G016200 Sapur.001G027700 Sapur.001G028900 Sapur.001G033600 Sapur.001G037600 Sapur.001G037800 Sapur.001G040600 Sapur.001G046900 Sapur.001G079000 Sapur.001G082900 Sapur.001G110400 Sapur.001G132900 Sapur.001G138400 Sapur.001G147300 Sapur.001G154800 Sapur.001G164900 Sapur.001G169300 Sapur.001G174500 Sapur.001G185600 Sapur.001G187000 Sapur.002G011100 Sapur.002G013700 Sapur.002G017000 Sapur.002G038200 Sapur.002G046500 Sapur.002G053800 Sapur.002G056800 Sapur.002G067400 Sapur.002G068100 Sapur.002G086000 Sapur.002G098200 Sapur.002G110800 Sapur.002G116800 Sapur.002G118400 Sapur.002G133400 Sapur.002G138300 Sapur.002G155000 Sapur.002G177900 Sapur.002G186300 Sapur.002G190100 Sapur.002G193600 Sapur.003G005100 Sapur.003G010700 Sapur.003G014800 Sapur.003G021100 Sapur.003G029600 Sapur.003G029700

	Sapur.003G039800 Sapur.003G061600 Sapur.003G065900 Sapur.003G074700 Sapur.003G076900 Sapur.003G082500 Sapur.003G090300 Sapur.003G092800 Sapur.003G094300 Sapur.003G096000 Sapur.003G100300 Sapur.003G107900 Sapur.003G127800 Sapur.003G134200 Sapur.003G137200 Sapur.003G138600 Sapur.003G139000 Sapur.004G005200 Sapur.004G020800 Sapur.004G021600 Sapur.004G023500 Sapur.004G025800 Sapur.004G026500 Sapur.004G034200 Sapur.004G048700 Sapur.004G050600 Sapur.004G070600 Sapur.004G076400 Sapur.004G099600 Sapur.004G115300 Sapur.004G131800 Sapur.004G132700 Sapur.004G141700 Sapur.004G155400 Sapur.004G162900 Sapur.004G168100 Sapur.004G168900 Sapur.004G171500 Sapur.004G172600 Sapur.005G031200 Sapur.005G031500 Sapur.005G034100 Sapur.005G051300 Sapur.005G058800 Sapur.005G080800 Sapur.005G121300 Sapur.005G138700 Sapur.005G160500 Sapur.005G172700 Sapur.005G174600 Sapur.005G180700 Sapur.005G191500 Sapur.005G204400 Sapur.006G001200 Sapur.006G004700 Sapur.006G009900 Sapur.006G015100 Sapur.006G015300 Sapur.006G023800 Sapur.006G034400 Sapur.006G037500 Sapur.006G049300 Sapur.006G059100 Sapur.006G062900 Sapur.006G076200 Sapur.006G084000 Sapur.006G102100 Sapur.006G102500 Sapur.006G105100 Sapur.006G105200 Sapur.006G123300 Sapur.006G150200 Sapur.006G153100 Sapur.006G167000 Sapur.006G186200 Sapur.006G190100 Sapur.006G198200 Sapur.006G201300 Sapur.006G223800 Sapur.007G021700 Sapur.007G023000 Sapur.007G027700 Sapur.007G051300 Sapur.007G111000 Sapur.008G005900 Sapur.008G015200 Sapur.008G025200 Sapur.008G038400 Sapur.008G050300 Sapur.008G071600 Sapur.008G076300 Sapur.008G104200 Sapur.008G105500 Sapur.008G108600 Sapur.008G113200 Sapur.008G126700 Sapur.008G142900 Sapur.008G153800 Sapur.008G162700 Sapur.009G003300 Sapur.009G006300 Sapur.009G009400 Sapur.009G036200 Sapur.009G039900 Sapur.009G054300 Sapur.009G057100 Sapur.009G062200 Sapur.009G062300 Sapur.009G063600 Sapur.009G068000 Sapur.009G090000 Sapur.009G091000 Sapur.009G109000 Sapur.009G109200 Sapur.009G117600 Sapur.009G129500 Sapur.009G131800 Sapur.010G007300 Sapur.010G014600 Sapur.010G048300 Sapur.010G066100 Sapur.010G073400 Sapur.010G081400 Sapur.010G085400 Sapur.010G087800 Sapur.010G090500 Sapur.010G103300 Sapur.010G108800 Sapur.010G127200 Sapur.010G133600 Sapur.010G141400 Sapur.010G146400 Sapur.010G149500 Sapur.010G150300 Sapur.010G151900 Sapur.010G159200 Sapur.010G165600 Sapur.010G189300 Sapur.010G195700 Sapur.011G006300 Sapur.011G034100 Sapur.011G041500 Sapur.011G050700 Sapur.011G075900 Sapur.011G101600 Sapur.011G106700 Sapur.011G107600 Sapur.011G113700 Sapur.011G115500 Sapur.011G121700 Sapur.012G007100 Sapur.012G009800 Sapur.012G025200 Sapur.012G028100 Sapur.012G030400 Sapur.012G034700 Sapur.012G040200 Sapur.012G043900 Sapur.012G071300 Sapur.012G089500 Sapur.012G100200 Sapur.013G034800 Sapur.013G066200 Sapur.013G066500 Sapur.013G068000 Sapur.013G080100 Sapur.013G090600 Sapur.013G100800 Sapur.013G101200 Sapur.013G105800 Sapur.013G109100 Sapur.013G109400 Sapur.013G128500 Sapur.014G044700 Sapur.014G048500 Sapur.014G050800 Sapur.014G060800 Sapur.014G083800 Sapur.014G089900 Sapur.014G092800 Sapur.014G099300 Sapur.014G102800 Sapur.014G104400 Sapur.014G105600 Sapur.014G113300 Sapur.014G123000 Sapur.014G123600 Sapur.014G138800 Sapur.016G040900 Sapur.016G047900 Sapur.016G065300 Sapur.016G096400 Sapur.016G097500 Sapur.016G098500 Sapur.016G102900 Sapur.016G109800 Sapur.016G113000 Sapur.016G138400

GO:0006810	321	1398	transport	Sapur.016G147600 Sapur.016G163500 Sapur.016G175100 Sapur.016G202100 Sapur.016G203700 Sapur.016G204500 Sapur.016G212300 Sapur.016G220900 Sapur.016G226600 Sapur.016G231500 Sapur.016G231600 Sapur.016G234000 Sapur.016G239800 Sapur.016G242000 Sapur.016G248500 Sapur.016G258100 Sapur.016G276200 Sapur.016G285800 Sapur.016G295000 Sapur.016G295100 Sapur.016G307500 Sapur.016G310000 Sapur.017G027100 Sapur.017G027200 Sapur.017G056200 Sapur.017G067400 Sapur.017G071700 Sapur.017G073700 Sapur.017G078400 Sapur.017G095100 Sapur.017G119100 Sapur.018G006400 Sapur.018G026100 Sapur.018G031900 Sapur.018G051100 Sapur.018G060200 Sapur.018G081000 Sapur.018G084500 Sapur.018G089800 Sapur.018G097400 Sapur.018G101700 Sapur.018G118500 Sapur.018G118800 Sapur.019G002100 Sapur.019G002700 Sapur.019G054200 Sapur.019G076600 Sapur.019G082100 Sapur.019G097900 Sapur.019G099600 Sapur.15WG044900 Sapur.15WG076900 Sapur.15WG078100 Sapur.15WG081100 Sapur.15ZG012800 Sapur.15ZG029200 Sapur.15ZG029900 Sapur.15ZG031600 Sapur.15ZG032200 Sapur.15ZG049100 Sapur.15ZG059400 Sapur.15ZG063600 Sapur.15ZG071300 Sapur.15ZG073700 Sapur.15ZG093800 Sapur.15ZG123400 Sapur.15ZG132200 Sapur.T001500 Sapur.T047800 Sapur.T080400 Sapur.T114400 Sapur.010G042500 Sapur.003G108000 Sapur.006G116300 Sapur.006G180200 Sapur.007G049600 Sapur.010G079100 Sapur.15ZG006400 Sapur.001G016200 Sapur.001G027700 Sapur.001G028900 Sapur.001G033600 Sapur.001G037600 Sapur.001G037800 Sapur.001G040600 Sapur.001G046900 Sapur.001G079000 Sapur.001G082900 Sapur.001G110400 Sapur.001G132900 Sapur.001G138400 Sapur.001G147300 Sapur.001G154800 Sapur.001G164900 Sapur.001G169300 Sapur.001G174500 Sapur.001G185600 Sapur.001G187000 Sapur.002G011100 Sapur.002G013700 Sapur.002G017000 Sapur.002G038200 Sapur.002G046500 Sapur.002G053800 Sapur.002G056800 Sapur.002G067400 Sapur.002G068100 Sapur.002G086000 Sapur.002G098200 Sapur.002G110800 Sapur.002G116800 Sapur.002G118400 Sapur.002G133400 Sapur.002G138300 Sapur.002G155000 Sapur.002G177900 Sapur.002G186300 Sapur.002G190100 Sapur.002G193600 Sapur.003G005100 Sapur.003G010700 Sapur.003G014800 Sapur.003G021100 Sapur.003G029600 Sapur.003G029700 Sapur.003G039800 Sapur.003G061600 Sapur.003G065900 Sapur.003G074700 Sapur.003G076900 Sapur.003G082500 Sapur.003G090300 Sapur.003G092800 Sapur.003G094300 Sapur.003G096000 Sapur.003G100300 Sapur.003G107900 Sapur.003G127800 Sapur.003G134200 Sapur.003G137200 Sapur.003G138600 Sapur.003G139000 Sapur.004G005200 Sapur.004G020800 Sapur.004G021600 Sapur.004G023500 Sapur.004G025800 Sapur.004G026500 Sapur.004G034200 Sapur.004G048700 Sapur.004G050600 Sapur.004G070600 Sapur.004G076400 Sapur.004G099600 Sapur.004G115300 Sapur.004G131800 Sapur.004G132700 Sapur.004G141700 Sapur.004G155400 Sapur.004G162900 Sapur.004G168100 Sapur.004G168900 Sapur.004G171500 Sapur.004G172600 Sapur.005G031200 Sapur.005G031500 Sapur.005G034100 Sapur.005G051300 Sapur.005G058800 Sapur.005G121300 Sapur.005G138700 Sapur.005G160500 Sapur.005G172700 Sapur.005G174600 Sapur.005G180700 Sapur.005G191500 Sapur.005G204400 Sapur.006G001200 Sapur.006G004700 Sapur.006G009900 Sapur.006G015100 Sapur.006G015300 Sapur.006G023800 Sapur.006G034400 Sapur.006G037500 Sapur.006G049300 Sapur.006G062900 Sapur.006G076200 Sapur.006G084000 Sapur.006G102100 Sapur.006G102500 Sapur.006G105100 Sapur.006G105200 Sapur.006G123300 Sapur.006G150200 Sapur.006G153100 Sapur.006G167000

Sapur.006G186200	Sapur.006G190100	Sapur.006G198200	Sapur.006G201300	Sapur.006G223800	Sapur.007G021700
Sapur.007G023000	Sapur.007G027700	Sapur.007G051300	Sapur.007G111000	Sapur.008G005900	Sapur.008G015200
Sapur.008G025200	Sapur.008G038400	Sapur.008G050300	Sapur.008G071600	Sapur.008G076300	Sapur.008G104200
Sapur.008G105500	Sapur.008G108600	Sapur.008G113200	Sapur.008G126700	Sapur.008G142900	Sapur.008G153800
Sapur.008G162700	Sapur.009G003300	Sapur.009G006300	Sapur.009G009400	Sapur.009G036200	Sapur.009G039900
Sapur.009G054300	Sapur.009G057100	Sapur.009G062200	Sapur.009G062300	Sapur.009G063600	Sapur.009G068000
Sapur.009G090000	Sapur.009G091000	Sapur.009G109000	Sapur.009G109200	Sapur.009G117600	Sapur.009G129500
Sapur.009G131800	Sapur.010G007300	Sapur.010G014600	Sapur.010G048300	Sapur.010G066100	Sapur.010G073400
Sapur.010G081400	Sapur.010G085400	Sapur.010G087800	Sapur.010G090500	Sapur.010G103300	Sapur.010G108800
Sapur.010G127200	Sapur.010G133600	Sapur.010G141400	Sapur.010G146400	Sapur.010G149500	Sapur.010G150300
Sapur.010G151900	Sapur.010G159200	Sapur.010G165600	Sapur.010G189300	Sapur.010G195700	Sapur.011G006300
Sapur.011G034100	Sapur.011G041500	Sapur.011G050700	Sapur.011G075900	Sapur.011G101600	Sapur.011G106700
Sapur.011G107600	Sapur.011G113700	Sapur.011G115500	Sapur.011G121700	Sapur.012G007100	Sapur.012G009800
Sapur.012G025200	Sapur.012G028100	Sapur.012G030400	Sapur.012G034700	Sapur.012G040200	Sapur.012G043900
Sapur.012G071300	Sapur.012G089500	Sapur.012G100200	Sapur.013G034800	Sapur.013G066200	Sapur.013G066500
Sapur.013G068000	Sapur.013G080100	Sapur.013G090600	Sapur.013G100800	Sapur.013G101200	Sapur.013G105800
Sapur.013G109100	Sapur.013G109400	Sapur.013G128500	Sapur.014G044700	Sapur.014G048500	Sapur.014G050800
Sapur.014G060800	Sapur.014G083800	Sapur.014G089900	Sapur.014G092800	Sapur.014G099300	Sapur.014G102800
Sapur.014G104400	Sapur.014G105600	Sapur.014G113300	Sapur.014G123000	Sapur.014G123600	Sapur.014G138800
Sapur.016G040900	Sapur.016G047900	Sapur.016G065300	Sapur.016G096400	Sapur.016G097500	Sapur.016G098500
Sapur.016G102900	Sapur.016G109800	Sapur.016G113000	Sapur.016G138400	Sapur.016G147600	Sapur.016G163500
Sapur.016G175100	Sapur.016G202100	Sapur.016G203700	Sapur.016G204500	Sapur.016G212300	Sapur.016G220900
Sapur.016G226600	Sapur.016G231500	Sapur.016G231600	Sapur.016G234000	Sapur.016G239800	Sapur.016G242000
Sapur.016G248500	Sapur.016G258100	Sapur.016G276200	Sapur.016G285800	Sapur.016G295000	Sapur.016G295100
Sapur.016G307500	Sapur.016G310000	Sapur.017G027100	Sapur.017G027200	Sapur.017G056200	Sapur.017G067400
Sapur.017G071700	Sapur.017G073700	Sapur.017G078400	Sapur.017G095100	Sapur.017G119100	Sapur.018G006400
Sapur.018G026100	Sapur.018G031900	Sapur.018G051100	Sapur.018G060200	Sapur.018G081000	Sapur.018G084500
Sapur.018G089800	Sapur.018G097400	Sapur.018G101700	Sapur.018G118500	Sapur.018G118800	Sapur.019G002100
Sapur.019G002700	Sapur.019G054200	Sapur.019G076600	Sapur.019G082100	Sapur.019G097900	Sapur.019G099600
Sapur.15WG044900	Sapur.15WG076900	Sapur.15WG078100	Sapur.15WG081100	Sapur.15ZG012800	Sapur.15ZG029200
Sapur.15ZG029900	Sapur.15ZG031600	Sapur.15ZG032200	Sapur.15ZG049100	Sapur.15ZG059400	Sapur.15ZG063600
Sapur.15ZG071300	Sapur.15ZG073700	Sapur.15ZG093800	Sapur.15ZG123400	Sapur.15ZG132200	Sapur.T001500
Sapur.T047800	Sapur.T080400	Sapur.T114400	Sapur.010G042500	Sapur.003G108000	Sapur.006G116300

				Sapur.006G180200 Sapur.007G049600 Sapur.010G079100 Sapur.15ZG006400
GO:0016192	67	164	vesicle-mediated transport	Sapur.001G037800 Sapur.001G040600 Sapur.001G082900 Sapur.001G132900 Sapur.001G154800 Sapur.002G013700 Sapur.002G046500 Sapur.002G053800 Sapur.002G118400 Sapur.002G138300 Sapur.002G186300 Sapur.003G029700 Sapur.003G039800 Sapur.003G094300 Sapur.003G134200 Sapur.003G137200 Sapur.003G139000 Sapur.004G026500 Sapur.004G034200 Sapur.004G099600 Sapur.004G115300 Sapur.005G160500 Sapur.005G180700 Sapur.006G076200 Sapur.006G153100 Sapur.006G167000 Sapur.006G201300 Sapur.006G223800 Sapur.007G111000 Sapur.008G025200 Sapur.009G054300 Sapur.009G057100 Sapur.009G091000 Sapur.009G109200 Sapur.009G129500 Sapur.010G146400 Sapur.010G149500 Sapur.010G159200 Sapur.010G195700 Sapur.011G034100 Sapur.011G041500 Sapur.011G113700 Sapur.011G115500 Sapur.011G121700 Sapur.012G043900 Sapur.012G089500 Sapur.013G109100 Sapur.013G109400 Sapur.014G048500 Sapur.014G050800 Sapur.014G123000 Sapur.014G138800 Sapur.016G065300 Sapur.016G102900 Sapur.016G163500 Sapur.016G276200 Sapur.017G027200 Sapur.018G006400 Sapur.018G084500 Sapur.019G097900 Sapur.15WG044900 Sapur.15WG081100 Sapur.15ZG012800 Sapur.15ZG049100 Sapur.15ZG059400 Sapur.15ZG071300 Sapur.T080400
GO:0009100	36	67	glycoprotein metabolic process	Sapur.002G004300 Sapur.002G028200 Sapur.002G072000 Sapur.002G176800 Sapur.003G010200 Sapur.003G099600 Sapur.004G138500 Sapur.004G155200 Sapur.005G101600 Sapur.005G180500 Sapur.005G204000 Sapur.006G008700 Sapur.006G054000 Sapur.006G212700 Sapur.007G028100 Sapur.008G028500 Sapur.008G052000 Sapur.008G149700 Sapur.009G079300 Sapur.010G023600 Sapur.010G118100 Sapur.010G129800 Sapur.010G178800 Sapur.011G112000 Sapur.012G052600 Sapur.013G056100 Sapur.014G118100 Sapur.014G125100 Sapur.016G010900 Sapur.016G252400 Sapur.016G290200 Sapur.018G062800 Sapur.019G079900 Sapur.15ZG018200 Sapur.15ZG021300 Sapur.15ZG101800
GO:0043413	34	61	macromolecule glycosylation	Sapur.002G004300 Sapur.002G028200 Sapur.002G072000 Sapur.002G176800 Sapur.003G010200 Sapur.003G099600 Sapur.004G138500 Sapur.004G155200 Sapur.005G101600 Sapur.005G180500 Sapur.005G204000 Sapur.006G008700 Sapur.006G054000 Sapur.006G212700 Sapur.007G028100 Sapur.008G028500 Sapur.008G149700 Sapur.009G079300 Sapur.010G023600 Sapur.010G118100 Sapur.010G129800 Sapur.010G178800 Sapur.011G112000 Sapur.012G052600 Sapur.014G118100 Sapur.014G125100 Sapur.016G010900 Sapur.016G252400 Sapur.016G290200 Sapur.018G062800 Sapur.019G079900 Sapur.15ZG018200 Sapur.15ZG021300 Sapur.15ZG101800
GO:0006486	34	61	protein glycosylation	Sapur.002G004300 Sapur.002G028200 Sapur.002G072000 Sapur.002G176800 Sapur.003G010200 Sapur.003G099600 Sapur.004G138500 Sapur.004G155200 Sapur.005G101600 Sapur.005G180500 Sapur.005G204000 Sapur.006G008700 Sapur.006G054000 Sapur.006G212700 Sapur.007G028100 Sapur.008G028500 Sapur.008G149700 Sapur.009G079300 Sapur.010G023600 Sapur.010G118100 Sapur.010G129800 Sapur.010G178800 Sapur.011G112000 Sapur.012G052600 Sapur.014G118100 Sapur.014G125100 Sapur.016G010900 Sapur.016G252400 Sapur.016G290200 Sapur.018G062800 Sapur.019G079900 Sapur.15ZG018200 Sapur.15ZG021300 Sapur.15ZG101800
GO:005	97	314	cellular	Sapur.001G027700 Sapur.001G028900 Sapur.001G037800 Sapur.001G082900 Sapur.001G110400

1641			localization	Sapur.001G147300 Sapur.001G154800 Sapur.002G013700 Sapur.002G016500 Sapur.002G017000 Sapur.002G053800 Sapur.002G116800 Sapur.002G118400 Sapur.002G190100 Sapur.003G029700 Sapur.003G065900 Sapur.003G074700 Sapur.003G094300 Sapur.003G137200 Sapur.003G139000 Sapur.004G005200 Sapur.004G023500 Sapur.004G026500 Sapur.004G050600 Sapur.004G070600 Sapur.004G115300 Sapur.004G126600 Sapur.005G051300 Sapur.005G080800 Sapur.005G174600 Sapur.005G191500 Sapur.006G059100 Sapur.006G062900 Sapur.006G076200 Sapur.006G123300 Sapur.006G150200 Sapur.006G201300 Sapur.006G223800 Sapur.007G023000 Sapur.008G108600 Sapur.008G142900 Sapur.009G009400 Sapur.009G039900 Sapur.009G054300 Sapur.009G057100 Sapur.009G091000 Sapur.009G102500 Sapur.009G109200 Sapur.010G081400 Sapur.010G108800 Sapur.010G141400 Sapur.010G146400 Sapur.010G149500 Sapur.010G159200 Sapur.011G006300 Sapur.011G034100 Sapur.011G075900 Sapur.011G106700 Sapur.011G113700 Sapur.012G028100 Sapur.012G030400 Sapur.012G034700 Sapur.013G034800 Sapur.013G066200 Sapur.013G066500 Sapur.013G128500 Sapur.014G044700 Sapur.014G048500 Sapur.014G060800 Sapur.014G099300 Sapur.014G123600 Sapur.016G109800 Sapur.016G113000 Sapur.016G163500 Sapur.016G192300 Sapur.016G203700 Sapur.016G204500 Sapur.016G212300 Sapur.016G248500 Sapur.016G285800 Sapur.017G044400 Sapur.017G044500 Sapur.017G119100 Sapur.018G006400 Sapur.018G031900 Sapur.018G051100 Sapur.018G081000 Sapur.019G097900 Sapur.019G099600 Sapur.15WG044900 Sapur.15ZG029200 Sapur.15ZG029900 Sapur.15ZG031600 Sapur.15ZG032200 Sapur.15ZG049100 Sapur.15ZG071300 Sapur.15ZG093800
GO:0009101	35	65	glycoprotein biosynthetic process	Sapur.002G004300 Sapur.002G028200 Sapur.002G072000 Sapur.002G176800 Sapur.003G010200 Sapur.003G099600 Sapur.004G138500 Sapur.004G155200 Sapur.005G101600 Sapur.005G180500 Sapur.005G204000 Sapur.006G008700 Sapur.006G054000 Sapur.006G212700 Sapur.007G028100 Sapur.008G028500 Sapur.008G052000 Sapur.008G149700 Sapur.009G079300 Sapur.010G023600 Sapur.010G118100 Sapur.010G129800 Sapur.010G178800 Sapur.011G112000 Sapur.012G052600 Sapur.014G118100 Sapur.014G125100 Sapur.016G010900 Sapur.016G252400 Sapur.016G290200 Sapur.018G062800 Sapur.019G079900 Sapur.15ZG018200 Sapur.15ZG021300 Sapur.15ZG101800
GO:0070085	34	66	glycosylation	Sapur.002G004300 Sapur.002G028200 Sapur.002G072000 Sapur.002G176800 Sapur.003G010200 Sapur.003G099600 Sapur.004G138500 Sapur.004G155200 Sapur.005G101600 Sapur.005G180500 Sapur.005G204000 Sapur.006G008700 Sapur.006G054000 Sapur.006G212700 Sapur.007G028100 Sapur.008G028500 Sapur.008G149700 Sapur.009G079300 Sapur.010G023600 Sapur.010G118100 Sapur.010G129800 Sapur.010G178800 Sapur.011G112000 Sapur.012G052600 Sapur.014G118100 Sapur.014G125100 Sapur.016G010900 Sapur.016G252400 Sapur.016G290200 Sapur.018G062800 Sapur.019G079900 Sapur.15ZG018200 Sapur.15ZG021300 Sapur.15ZG101800
GO:0070727	85	272	cellular macromolecule localization	Sapur.001G027700 Sapur.001G028900 Sapur.001G082900 Sapur.001G110400 Sapur.001G154800 Sapur.002G013700 Sapur.002G016500 Sapur.002G017000 Sapur.002G116800 Sapur.002G190100 Sapur.003G029700 Sapur.003G065900 Sapur.003G074700 Sapur.003G094300 Sapur.004G005200 Sapur.004G023500 Sapur.004G026500 Sapur.004G050600 Sapur.004G070600 Sapur.004G115300 Sapur.004G126600 Sapur.005G080800 Sapur.005G174600 Sapur.005G191500 Sapur.006G059100 Sapur.006G062900 Sapur.006G076200 Sapur.006G123300 Sapur.006G150200

				Sapur.006G201300 Sapur.006G223800 Sapur.007G023000 Sapur.008G108600 Sapur.008G142900 Sapur.009G009400 Sapur.009G039900 Sapur.009G057100 Sapur.009G091000 Sapur.009G102500 Sapur.010G081400 Sapur.010G108800 Sapur.010G141400 Sapur.010G146400 Sapur.010G149500 Sapur.010G159200 Sapur.011G006300 Sapur.011G034100 Sapur.011G075900 Sapur.011G106700 Sapur.012G028100 Sapur.012G030400 Sapur.012G034700 Sapur.013G034800 Sapur.013G066200 Sapur.013G066500 Sapur.013G128500 Sapur.014G044700 Sapur.014G060800 Sapur.014G099300 Sapur.014G123600 Sapur.016G109800 Sapur.016G113000 Sapur.016G163500 Sapur.016G192300 Sapur.016G203700 Sapur.016G204500 Sapur.016G212300 Sapur.016G248500 Sapur.016G285800 Sapur.017G044400 Sapur.017G044500 Sapur.017G119100 Sapur.018G006400 Sapur.018G031900 Sapur.018G081000 Sapur.019G097900 Sapur.019G099600 Sapur.15WG044900 Sapur.15ZG029200 Sapur.15ZG029900 Sapur.15ZG031600 Sapur.15ZG032200 Sapur.15ZG049100 Sapur.15ZG071300 Sapur.15ZG093800
GO:0008104	85	272	protein localization	Sapur.001G027700 Sapur.001G028900 Sapur.001G082900 Sapur.001G110400 Sapur.001G154800 Sapur.002G013700 Sapur.002G016500 Sapur.002G017000 Sapur.002G116800 Sapur.002G190100 Sapur.003G029700 Sapur.003G065900 Sapur.003G074700 Sapur.003G094300 Sapur.004G005200 Sapur.004G023500 Sapur.004G026500 Sapur.004G050600 Sapur.004G070600 Sapur.004G115300 Sapur.004G126600 Sapur.005G080800 Sapur.005G174600 Sapur.005G191500 Sapur.006G059100 Sapur.006G062900 Sapur.006G076200 Sapur.006G123300 Sapur.006G150200 Sapur.006G201300 Sapur.006G223800 Sapur.007G023000 Sapur.008G108600 Sapur.008G142900 Sapur.009G009400 Sapur.009G039900 Sapur.009G057100 Sapur.009G091000 Sapur.009G102500 Sapur.010G081400 Sapur.010G108800 Sapur.010G141400 Sapur.010G146400 Sapur.010G149500 Sapur.010G159200 Sapur.011G006300 Sapur.011G034100 Sapur.011G075900 Sapur.011G106700 Sapur.012G028100 Sapur.012G030400 Sapur.012G034700 Sapur.013G034800 Sapur.013G066200 Sapur.013G066500 Sapur.013G128500 Sapur.014G044700 Sapur.014G060800 Sapur.014G099300 Sapur.014G123600 Sapur.016G109800 Sapur.016G113000 Sapur.016G163500 Sapur.016G192300 Sapur.016G203700 Sapur.016G204500 Sapur.016G212300 Sapur.016G248500 Sapur.016G285800 Sapur.017G044400 Sapur.017G044500 Sapur.017G119100 Sapur.018G006400 Sapur.018G031900 Sapur.018G081000 Sapur.019G097900 Sapur.019G099600 Sapur.15WG044900 Sapur.15ZG029200 Sapur.15ZG029900 Sapur.15ZG031600 Sapur.15ZG032200 Sapur.15ZG049100 Sapur.15ZG071300 Sapur.15ZG093800
GO:0033036	90	298	macromolecule localization	Sapur.001G027700 Sapur.001G028900 Sapur.001G082900 Sapur.001G110400 Sapur.001G154800 Sapur.002G013700 Sapur.002G016500 Sapur.002G017000 Sapur.002G116800 Sapur.002G190100 Sapur.003G021100 Sapur.003G029700 Sapur.003G065900 Sapur.003G074700 Sapur.003G094300 Sapur.004G005200 Sapur.004G023500 Sapur.004G026500 Sapur.004G050600 Sapur.004G070600 Sapur.004G115300 Sapur.004G126600 Sapur.004G171500 Sapur.005G080800 Sapur.005G174600 Sapur.005G191500 Sapur.006G059100 Sapur.006G062900 Sapur.006G076200 Sapur.006G123300 Sapur.006G150200 Sapur.006G201300 Sapur.006G223800 Sapur.007G023000 Sapur.008G108600 Sapur.008G142900 Sapur.008G153800 Sapur.009G009400 Sapur.009G039900 Sapur.009G057100 Sapur.009G091000 Sapur.009G102500 Sapur.010G081400 Sapur.010G103300 Sapur.010G108800 Sapur.010G141400 Sapur.010G146400

				Sapur.010G149500 Sapur.010G159200 Sapur.011G006300 Sapur.011G034100 Sapur.011G075900 Sapur.011G106700 Sapur.012G028100 Sapur.012G030400 Sapur.012G034700 Sapur.013G034800 Sapur.013G066200 Sapur.013G066500 Sapur.013G128500 Sapur.014G044700 Sapur.014G060800 Sapur.014G099300 Sapur.014G123600 Sapur.016G109800 Sapur.016G113000 Sapur.016G163500 Sapur.016G192300 Sapur.016G203700 Sapur.016G204500 Sapur.016G212300 Sapur.016G248500 Sapur.016G285800 Sapur.017G044400 Sapur.017G044500 Sapur.017G119100 Sapur.018G006400 Sapur.018G031900 Sapur.018G081000 Sapur.019G097900 Sapur.019G099600 Sapur.15WG044900 Sapur.15ZG029200 Sapur.15ZG029900 Sapur.15ZG031600 Sapur.15ZG032200 Sapur.15ZG049100 Sapur.15ZG071300 Sapur.15ZG093800 Sapur.010G042500
GO:0006508	127	483	proteolysis	Sapur.001G001400 Sapur.001G031800 Sapur.001G051400 Sapur.001G088500 Sapur.001G098400 Sapur.001G135000 Sapur.001G139800 Sapur.001G146600 Sapur.002G003700 Sapur.002G009400 Sapur.002G022300 Sapur.002G056400 Sapur.002G063400 Sapur.002G072700 Sapur.002G084000 Sapur.002G097300 Sapur.002G101500 Sapur.002G108400 Sapur.002G124000 Sapur.002G134700 Sapur.002G143900 Sapur.002G150900 Sapur.003G034800 Sapur.003G037900 Sapur.003G045000 Sapur.003G087600 Sapur.003G087900 Sapur.003G088100 Sapur.003G105400 Sapur.003G151400 Sapur.004G005000 Sapur.004G058500 Sapur.004G060000 Sapur.004G120300 Sapur.004G130500 Sapur.004G149500 Sapur.004G162500 Sapur.004G164900 Sapur.004G165000 Sapur.004G173100 Sapur.005G001600 Sapur.005G028800 Sapur.005G047600 Sapur.005G053000 Sapur.005G062300 Sapur.005G072700 Sapur.005G086400 Sapur.005G086600 Sapur.005G087800 Sapur.005G122400 Sapur.005G123400 Sapur.005G125000 Sapur.005G147300 Sapur.005G194900 Sapur.005G204800 Sapur.006G026100 Sapur.006G061500 Sapur.006G063300 Sapur.006G070700 Sapur.006G115800 Sapur.006G169900 Sapur.006G192500 Sapur.006G194300 Sapur.007G042300 Sapur.007G060300 Sapur.007G095100 Sapur.007G113300 Sapur.008G024600 Sapur.008G031500 Sapur.008G045500 Sapur.008G051200 Sapur.008G051300 Sapur.008G061000 Sapur.008G093300 Sapur.008G142600 Sapur.009G000500 Sapur.009G001100 Sapur.009G023600 Sapur.009G024900 Sapur.009G042700 Sapur.009G096300 Sapur.009G106500 Sapur.009G125500 Sapur.009G126900 Sapur.010G051000 Sapur.010G098600 Sapur.010G115800 Sapur.011G006500 Sapur.012G013000 Sapur.012G013500 Sapur.012G018300 Sapur.012G080500 Sapur.012G086400 Sapur.012G101000 Sapur.012G107200 Sapur.014G010900 Sapur.014G013900 Sapur.014G030900 Sapur.014G039700 Sapur.014G043900 Sapur.014G077700 Sapur.014G096400 Sapur.016G030700 Sapur.016G066000 Sapur.016G133500 Sapur.016G181700 Sapur.016G183900 Sapur.016G228400 Sapur.016G294800 Sapur.017G109200 Sapur.018G005200 Sapur.018G010500 Sapur.018G010800 Sapur.018G010900 Sapur.018G011100 Sapur.018G011200 Sapur.018G058400 Sapur.018G078800 Sapur.019G016700 Sapur.019G022400 Sapur.019G052800 Sapur.019G053400 Sapur.15ZG010600 Sapur.15ZG102400 Sapur.15ZG130400 Sapur.T026500 Sapur.T045900
GO:0045184	79	263	establishment of protein localization	Sapur.001G027700 Sapur.001G028900 Sapur.001G082900 Sapur.001G110400 Sapur.001G154800 Sapur.002G013700 Sapur.002G017000 Sapur.002G116800 Sapur.002G190100 Sapur.003G029700 Sapur.003G065900 Sapur.003G074700 Sapur.003G094300 Sapur.004G005200 Sapur.004G023500 Sapur.004G026500 Sapur.004G050600

				Sapur.004G070600 Sapur.004G115300 Sapur.005G080800 Sapur.005G174600 Sapur.005G191500 Sapur.006G059100 Sapur.006G062900 Sapur.006G076200 Sapur.006G123300 Sapur.006G150200 Sapur.006G201300 Sapur.006G223800 Sapur.007G023000 Sapur.008G108600 Sapur.008G142900 Sapur.009G009400 Sapur.009G039900 Sapur.009G057100 Sapur.009G091000 Sapur.010G081400 Sapur.010G108800 Sapur.010G141400 Sapur.010G146400 Sapur.010G149500 Sapur.010G159200 Sapur.011G006300 Sapur.011G034100 Sapur.011G075900 Sapur.011G106700 Sapur.012G028100 Sapur.012G030400 Sapur.012G034700 Sapur.013G034800 Sapur.013G066200 Sapur.013G066500 Sapur.013G128500 Sapur.014G044700 Sapur.014G060800 Sapur.014G099300 Sapur.014G123600 Sapur.016G109800 Sapur.016G113000 Sapur.016G163500 Sapur.016G203700 Sapur.016G204500 Sapur.016G212300 Sapur.016G248500 Sapur.016G285800 Sapur.017G119100 Sapur.018G006400 Sapur.018G031900 Sapur.018G081000 Sapur.019G097900 Sapur.019G099600 Sapur.15WG044900 Sapur.15ZG029200 Sapur.15ZG029900 Sapur.15ZG031600 Sapur.15ZG032200 Sapur.15ZG049100 Sapur.15ZG071300 Sapur.15ZG093800
GO:0071702	95	339	organic substance transport	Sapur.001G027700 Sapur.001G028900 Sapur.001G082900 Sapur.001G110400 Sapur.001G154800 Sapur.002G013700 Sapur.002G017000 Sapur.002G110800 Sapur.002G116800 Sapur.002G190100 Sapur.003G014800 Sapur.003G021100 Sapur.003G029700 Sapur.003G065900 Sapur.003G074700 Sapur.003G094300 Sapur.003G100300 Sapur.004G005200 Sapur.004G023500 Sapur.004G026500 Sapur.004G050600 Sapur.004G070600 Sapur.004G076400 Sapur.004G115300 Sapur.004G171500 Sapur.005G058800 Sapur.005G174600 Sapur.005G191500 Sapur.006G062900 Sapur.006G076200 Sapur.006G123300 Sapur.006G150200 Sapur.006G201300 Sapur.006G223800 Sapur.007G023000 Sapur.008G108600 Sapur.008G142900 Sapur.008G153800 Sapur.009G009400 Sapur.009G039900 Sapur.009G057100 Sapur.009G091000 Sapur.010G081400 Sapur.010G090500 Sapur.010G103300 Sapur.010G108800 Sapur.010G141400 Sapur.010G146400 Sapur.010G149500 Sapur.010G159200 Sapur.011G006300 Sapur.011G034100 Sapur.011G075900 Sapur.011G106700 Sapur.012G028100 Sapur.012G030400 Sapur.012G034700 Sapur.012G071300 Sapur.013G034800 Sapur.013G066200 Sapur.013G066500 Sapur.013G128500 Sapur.014G044700 Sapur.014G060800 Sapur.014G099300 Sapur.014G123600 Sapur.016G098500 Sapur.016G109800 Sapur.016G113000 Sapur.016G163500 Sapur.016G203700 Sapur.016G204500 Sapur.016G212300 Sapur.016G242000 Sapur.016G248500 Sapur.016G285800 Sapur.017G027100 Sapur.017G119100 Sapur.018G006400 Sapur.018G031900 Sapur.018G081000 Sapur.019G097900 Sapur.019G099600 Sapur.15WG044900 Sapur.15ZG029200 Sapur.15ZG029900 Sapur.15ZG031600 Sapur.15ZG032200 Sapur.15ZG049100 Sapur.15ZG071300 Sapur.15ZG093800 Sapur.T001500 Sapur.010G042500 Sapur.010G079100 Sapur.004G168100
GO:0015031	77	254	protein transport	Sapur.001G027700 Sapur.001G028900 Sapur.001G082900 Sapur.001G110400 Sapur.001G154800 Sapur.002G013700 Sapur.002G017000 Sapur.002G116800 Sapur.002G190100 Sapur.003G029700 Sapur.003G065900 Sapur.003G074700 Sapur.003G094300 Sapur.004G005200 Sapur.004G023500 Sapur.004G026500 Sapur.004G050600 Sapur.004G070600 Sapur.004G115300 Sapur.005G174600 Sapur.005G191500 Sapur.006G062900 Sapur.006G076200 Sapur.006G123300 Sapur.006G150200 Sapur.006G201300 Sapur.006G223800 Sapur.007G023000 Sapur.008G108600 Sapur.008G142900 Sapur.009G009400 Sapur.009G039900 Sapur.009G057100 Sapur.009G091000 Sapur.010G081400

				Sapur.010G108800 Sapur.010G141400 Sapur.010G146400 Sapur.010G149500 Sapur.010G159200 Sapur.011G006300 Sapur.011G034100 Sapur.011G075900 Sapur.011G106700 Sapur.012G028100 Sapur.012G030400 Sapur.012G034700 Sapur.013G034800 Sapur.013G066200 Sapur.013G066500 Sapur.013G128500 Sapur.014G044700 Sapur.014G060800 Sapur.014G099300 Sapur.014G123600 Sapur.016G109800 Sapur.016G113000 Sapur.016G163500 Sapur.016G203700 Sapur.016G204500 Sapur.016G212300 Sapur.016G248500 Sapur.016G285800 Sapur.017G119100 Sapur.018G006400 Sapur.018G031900 Sapur.018G081000 Sapur.019G097900 Sapur.019G099600 Sapur.15WG044900 Sapur.15ZG029200 Sapur.15ZG029900 Sapur.15ZG031600 Sapur.15ZG032200 Sapur.15ZG049100 Sapur.15ZG071300 Sapur.15ZG093800
GO:1901137	50	139	carbohydrate derivative biosynthetic process	Sapur.001G026000 Sapur.001G050000 Sapur.002G004300 Sapur.002G028200 Sapur.002G072000 Sapur.002G176800 Sapur.002G189800 Sapur.003G010200 Sapur.003G038600 Sapur.003G054000 Sapur.003G060700 Sapur.003G062100 Sapur.003G099600 Sapur.003G123600 Sapur.004G138500 Sapur.004G155200 Sapur.005G101600 Sapur.005G180500 Sapur.005G204000 Sapur.006G008700 Sapur.006G054000 Sapur.006G212700 Sapur.007G028100 Sapur.008G028500 Sapur.008G052000 Sapur.008G149700 Sapur.009G047600 Sapur.009G079300 Sapur.010G023600 Sapur.010G118100 Sapur.010G129800 Sapur.010G173300 Sapur.010G178800 Sapur.010G202000 Sapur.011G112000 Sapur.012G052600 Sapur.014G062000 Sapur.014G118100 Sapur.014G125100 Sapur.016G010900 Sapur.016G252400 Sapur.016G290200 Sapur.018G041100 Sapur.018G062800 Sapur.019G022400 Sapur.019G079900 Sapur.15ZG018200 Sapur.15ZG021300 Sapur.15ZG101800 Sapur.T049800
GO:0071705	93	336	nitrogen compound transport	Sapur.001G027700 Sapur.001G028900 Sapur.001G082900 Sapur.001G110400 Sapur.001G154800 Sapur.002G013700 Sapur.002G017000 Sapur.002G038200 Sapur.002G110800 Sapur.002G116800 Sapur.002G190100 Sapur.003G014800 Sapur.003G029600 Sapur.003G029700 Sapur.003G065900 Sapur.003G074700 Sapur.003G094300 Sapur.004G005200 Sapur.004G023500 Sapur.004G026500 Sapur.004G050600 Sapur.004G070600 Sapur.004G076400 Sapur.004G115300 Sapur.005G174600 Sapur.005G191500 Sapur.006G062900 Sapur.006G076200 Sapur.006G084000 Sapur.006G123300 Sapur.006G150200 Sapur.006G201300 Sapur.006G223800 Sapur.007G023000 Sapur.008G105500 Sapur.008G108600 Sapur.008G126700 Sapur.008G142900 Sapur.009G009400 Sapur.009G039900 Sapur.009G057100 Sapur.009G091000 Sapur.010G048300 Sapur.010G081400 Sapur.010G090500 Sapur.010G108800 Sapur.010G141400 Sapur.010G146400 Sapur.010G149500 Sapur.010G159200 Sapur.011G006300 Sapur.011G034100 Sapur.011G075900 Sapur.011G106700 Sapur.012G028100 Sapur.012G030400 Sapur.012G034700 Sapur.013G034800 Sapur.013G066200 Sapur.013G066500 Sapur.013G128500 Sapur.014G044700 Sapur.014G060800 Sapur.014G099300 Sapur.014G123600 Sapur.016G109800 Sapur.016G113000 Sapur.016G163500 Sapur.016G203700 Sapur.016G204500 Sapur.016G212300 Sapur.016G242000 Sapur.016G248500 Sapur.016G285800 Sapur.017G119100 Sapur.018G006400 Sapur.018G031900 Sapur.018G081000 Sapur.019G097900 Sapur.019G099600 Sapur.15WG044900 Sapur.15ZG029200 Sapur.15ZG029900 Sapur.15ZG031600 Sapur.15ZG032200 Sapur.15ZG049100 Sapur.15ZG071300 Sapur.15ZG093800 Sapur.T001500 Sapur.003G108000 Sapur.007G049600 Sapur.15ZG006400 Sapur.004G168100
GO:001	14	19	protein	Sapur.001G146600 Sapur.002G150900 Sapur.003G034800 Sapur.004G058500 Sapur.004G149500

6485			processing	Sapur.005G028800 Sapur.006G194300 Sapur.007G113300 Sapur.008G051200 Sapur.008G051300 Sapur.008G142600 Sapur.009G125500 Sapur.010G051000 Sapur.018G005200
GO:0051649	71	257	establishment of localization in cell	Sapur.001G028900 Sapur.001G037800 Sapur.001G082900 Sapur.001G147300 Sapur.001G154800 Sapur.002G013700 Sapur.002G116800 Sapur.002G118400 Sapur.002G190100 Sapur.003G029700 Sapur.003G074700 Sapur.003G094300 Sapur.003G137200 Sapur.003G139000 Sapur.004G005200 Sapur.004G026500 Sapur.004G050600 Sapur.004G070600 Sapur.004G115300 Sapur.005G051300 Sapur.005G174600 Sapur.006G062900 Sapur.006G076200 Sapur.006G150200 Sapur.006G201300 Sapur.006G223800 Sapur.008G108600 Sapur.008G142900 Sapur.009G009400 Sapur.009G054300 Sapur.009G057100 Sapur.009G091000 Sapur.009G109200 Sapur.010G081400 Sapur.010G108800 Sapur.010G141400 Sapur.010G146400 Sapur.010G149500 Sapur.010G159200 Sapur.011G006300 Sapur.011G034100 Sapur.011G106700 Sapur.011G113700 Sapur.012G028100 Sapur.012G030400 Sapur.013G066200 Sapur.013G066500 Sapur.014G044700 Sapur.014G048500 Sapur.014G099300 Sapur.014G123600 Sapur.016G109800 Sapur.016G113000 Sapur.016G163500 Sapur.016G203700 Sapur.016G204500 Sapur.016G212300 Sapur.016G285800 Sapur.017G119100 Sapur.018G006400 Sapur.018G031900 Sapur.018G051100 Sapur.018G081000 Sapur.019G097900 Sapur.15WG044900 Sapur.15ZG029200 Sapur.15ZG029900 Sapur.15ZG031600 Sapur.15ZG032200 Sapur.15ZG049100 Sapur.15ZG071300
GO:0046907	71	257	intracellular transport	Sapur.001G028900 Sapur.001G037800 Sapur.001G082900 Sapur.001G147300 Sapur.001G154800 Sapur.002G013700 Sapur.002G116800 Sapur.002G118400 Sapur.002G190100 Sapur.003G029700 Sapur.003G074700 Sapur.003G094300 Sapur.003G137200 Sapur.003G139000 Sapur.004G005200 Sapur.004G026500 Sapur.004G050600 Sapur.004G070600 Sapur.004G115300 Sapur.005G051300 Sapur.005G174600 Sapur.006G062900 Sapur.006G076200 Sapur.006G150200 Sapur.006G201300 Sapur.006G223800 Sapur.008G108600 Sapur.008G142900 Sapur.009G009400 Sapur.009G054300 Sapur.009G057100 Sapur.009G091000 Sapur.009G109200 Sapur.010G081400 Sapur.010G108800 Sapur.010G141400 Sapur.010G146400 Sapur.010G149500 Sapur.010G159200 Sapur.011G006300 Sapur.011G034100 Sapur.011G106700 Sapur.011G113700 Sapur.012G028100 Sapur.012G030400 Sapur.013G066200 Sapur.013G066500 Sapur.014G044700 Sapur.014G048500 Sapur.014G099300 Sapur.014G123600 Sapur.016G109800 Sapur.016G113000 Sapur.016G163500 Sapur.016G203700 Sapur.016G204500 Sapur.016G212300 Sapur.016G285800 Sapur.017G119100 Sapur.018G006400 Sapur.018G031900 Sapur.018G051100 Sapur.018G081000 Sapur.019G097900 Sapur.15WG044900 Sapur.15ZG029200 Sapur.15ZG029900 Sapur.15ZG031600 Sapur.15ZG032200 Sapur.15ZG049100 Sapur.15ZG071300
GO:0007010	34	93	cytoskeleton organization	Sapur.001G044900 Sapur.002G146800 Sapur.002G174200 Sapur.003G130500 Sapur.004G039300 Sapur.004G130100 Sapur.005G156700 Sapur.006G028600 Sapur.006G038100 Sapur.006G086600 Sapur.006G135400 Sapur.008G109800 Sapur.008G113100 Sapur.008G139000 Sapur.008G147700 Sapur.009G033900 Sapur.010G044700 Sapur.010G164300 Sapur.010G206600 Sapur.011G046000 Sapur.013G017100 Sapur.014G034600 Sapur.014G114900 Sapur.014G128900 Sapur.016G112500 Sapur.016G129900 Sapur.016G130000 Sapur.016G201500 Sapur.017G045900 Sapur.017G082000 Sapur.017G125500 Sapur.018G096500 Sapur.T001400 Sapur.15WG079100
GO:000	55	187	microtubule-base	Sapur.001G000500 Sapur.001G044900 Sapur.001G086500 Sapur.002G015900 Sapur.002G090500

7017			d process	Sapur.002G122000 Sapur.002G146800 Sapur.002G159800 Sapur.002G174200 Sapur.002G182100 Sapur.003G028400 Sapur.003G090100 Sapur.003G091100 Sapur.003G130500 Sapur.003G166900 Sapur.003G169700 Sapur.004G120900 Sapur.004G122400 Sapur.005G015200 Sapur.005G192400 Sapur.006G025400 Sapur.006G159000 Sapur.007G012500 Sapur.007G043900 Sapur.008G113100 Sapur.008G161000 Sapur.009G018700 Sapur.009G052100 Sapur.009G052200 Sapur.009G066800 Sapur.009G097000 Sapur.010G008800 Sapur.010G206600 Sapur.011G097800 Sapur.011G108500 Sapur.011G119600 Sapur.013G010400 Sapur.014G014400 Sapur.014G128900 Sapur.016G030100 Sapur.016G054000 Sapur.016G056900 Sapur.016G077400 Sapur.016G094800 Sapur.016G127100 Sapur.016G158100 Sapur.016G158200 Sapur.016G172500 Sapur.016G275100 Sapur.016G302200 Sapur.016G302700 Sapur.017G082000 Sapur.017G125500 Sapur.019G035200 Sapur.T001400
GO:0030029	20	41	actin filament-based process	Sapur.004G039300 Sapur.004G130100 Sapur.005G156700 Sapur.006G086600 Sapur.008G139000 Sapur.008G147700 Sapur.009G033900 Sapur.010G044700 Sapur.010G164300 Sapur.011G046000 Sapur.013G017100 Sapur.014G034600 Sapur.014G114900 Sapur.016G112500 Sapur.016G129900 Sapur.016G130000 Sapur.016G201500 Sapur.017G045900 Sapur.018G096500 Sapur.15WG079100
GO:0030036	20	41	actin cytoskeleton organization	Sapur.004G039300 Sapur.004G130100 Sapur.005G156700 Sapur.006G086600 Sapur.008G139000 Sapur.008G147700 Sapur.009G033900 Sapur.010G044700 Sapur.010G164300 Sapur.011G046000 Sapur.013G017100 Sapur.014G034600 Sapur.014G114900 Sapur.016G112500 Sapur.016G129900 Sapur.016G130000 Sapur.016G201500 Sapur.017G045900 Sapur.018G096500 Sapur.15WG079100
GO:0044272	17	32	sulfur compound biosynthetic process	Sapur.001G050000 Sapur.002G168400 Sapur.003G123600 Sapur.004G144000 Sapur.005G067900 Sapur.006G101400 Sapur.007G074100 Sapur.008G052000 Sapur.008G078200 Sapur.009G120400 Sapur.010G119600 Sapur.013G056900 Sapur.014G091500 Sapur.019G038000 Sapur.019G038200 Sapur.019G098500 Sapur.T140300
GO:0006886	59	213	intracellular protein transport	Sapur.001G028900 Sapur.001G082900 Sapur.001G154800 Sapur.002G116800 Sapur.002G190100 Sapur.003G029700 Sapur.003G074700 Sapur.003G094300 Sapur.004G005200 Sapur.004G026500 Sapur.004G050600 Sapur.004G070600 Sapur.004G115300 Sapur.005G174600 Sapur.006G062900 Sapur.006G076200 Sapur.006G150200 Sapur.006G201300 Sapur.006G223800 Sapur.008G108600 Sapur.008G142900 Sapur.009G009400 Sapur.009G057100 Sapur.009G091000 Sapur.010G081400 Sapur.010G108800 Sapur.010G141400 Sapur.010G146400 Sapur.010G149500 Sapur.010G159200 Sapur.011G006300 Sapur.011G034100 Sapur.011G106700 Sapur.012G028100 Sapur.012G030400 Sapur.013G066200 Sapur.013G066500 Sapur.014G044700 Sapur.014G099300 Sapur.014G123600 Sapur.016G109800 Sapur.016G113000 Sapur.016G163500 Sapur.016G203700 Sapur.016G204500 Sapur.016G212300 Sapur.016G285800 Sapur.017G119100 Sapur.018G006400 Sapur.018G031900 Sapur.018G081000 Sapur.019G097900 Sapur.15WG044900 Sapur.15ZG029200 Sapur.15ZG029900 Sapur.15ZG031600 Sapur.15ZG032200 Sapur.15ZG049100 Sapur.15ZG071300
GO:1901135	61	232	carbohydrate derivative metabolic process	Sapur.001G026000 Sapur.001G050000 Sapur.002G004300 Sapur.002G028200 Sapur.002G072000 Sapur.002G176800 Sapur.002G189800 Sapur.003G010200 Sapur.003G038600 Sapur.003G054000 Sapur.003G060700 Sapur.003G062100 Sapur.003G099600 Sapur.003G123600 Sapur.004G138500 Sapur.004G155200 Sapur.005G040300

				Sapur.005G101600 Sapur.005G180500 Sapur.005G204000 Sapur.006G008700 Sapur.006G054000 Sapur.006G124700 Sapur.006G177400 Sapur.006G212700 Sapur.007G028100 Sapur.008G028500 Sapur.008G052000 Sapur.008G149700 Sapur.009G047600 Sapur.009G079300 Sapur.010G023600 Sapur.010G110700 Sapur.010G118100 Sapur.010G129800 Sapur.010G173300 Sapur.010G178800 Sapur.010G202000 Sapur.010G205000 Sapur.011G112000 Sapur.012G052600 Sapur.013G037300 Sapur.013G056100 Sapur.013G113400 Sapur.014G062000 Sapur.014G118100 Sapur.014G118900 Sapur.014G125100 Sapur.016G010900 Sapur.016G212300 Sapur.016G252400 Sapur.016G290200 Sapur.018G041100 Sapur.018G062800 Sapur.019G022400 Sapur.019G079900 Sapur.15ZG018200 Sapur.15ZG021300 Sapur.15ZG101800 Sapur.T049800 Sapur.004G077000
GO:0006555	7	8	methionine metabolic process	Sapur.004G144000 Sapur.009G120400 Sapur.013G056900 Sapur.017G000100 Sapur.019G038000 Sapur.019G038200 Sapur.T140300
GO:0006465	9	13	signal peptide processing	Sapur.001G146600 Sapur.003G034800 Sapur.005G028800 Sapur.006G194300 Sapur.008G051200 Sapur.008G051300 Sapur.008G142600 Sapur.010G051000 Sapur.018G005200
GO:0030041	8	11	actin filament polymerization	Sapur.005G156700 Sapur.008G139000 Sapur.010G044700 Sapur.013G017100 Sapur.014G034600 Sapur.014G114900 Sapur.017G045900 Sapur.018G096500
GO:0007015	16	37	actin filament organization	Sapur.004G130100 Sapur.005G156700 Sapur.008G139000 Sapur.008G147700 Sapur.009G033900 Sapur.010G044700 Sapur.010G164300 Sapur.013G017100 Sapur.014G034600 Sapur.014G114900 Sapur.016G129900 Sapur.016G130000 Sapur.016G201500 Sapur.017G045900 Sapur.018G096500 Sapur.15WG079100
GO:0045010	9	14	actin nucleation	Sapur.008G139000 Sapur.008G147700 Sapur.010G044700 Sapur.014G034600 Sapur.014G114900 Sapur.016G201500 Sapur.017G045900 Sapur.018G096500 Sapur.15WG079100
GO:0097435	17	41	supramolecular fiber organization	Sapur.002G146800 Sapur.004G130100 Sapur.005G156700 Sapur.008G139000 Sapur.008G147700 Sapur.009G033900 Sapur.010G044700 Sapur.010G164300 Sapur.013G017100 Sapur.014G034600 Sapur.014G114900 Sapur.016G129900 Sapur.016G130000 Sapur.016G201500 Sapur.017G045900 Sapur.018G096500 Sapur.15WG079100
GO:0006694	17	41	steroid biosynthetic process	Sapur.002G002500 Sapur.002G011200 Sapur.002G103600 Sapur.002G119700 Sapur.002G157000 Sapur.003G079300 Sapur.006G175000 Sapur.008G041000 Sapur.009G044900 Sapur.010G163100 Sapur.014G049800 Sapur.014G103300 Sapur.016G074400 Sapur.016G130500 Sapur.016G240600 Sapur.016G246100 Sapur.017G049800
GO:0009086	6	7	methionine biosynthetic process	Sapur.004G144000 Sapur.009G120400 Sapur.013G056900 Sapur.019G038000 Sapur.019G038200 Sapur.T140300
GO:0034314	8	12	Arp2/3 complex-mediated actin nucleation	Sapur.008G139000 Sapur.008G147700 Sapur.010G044700 Sapur.014G034600 Sapur.014G114900 Sapur.016G201500 Sapur.017G045900 Sapur.018G096500
GO:0008202	17	43	steroid metabolic process	Sapur.002G002500 Sapur.002G011200 Sapur.002G103600 Sapur.002G119700 Sapur.002G157000 Sapur.003G079300 Sapur.006G175000 Sapur.008G041000 Sapur.009G044900 Sapur.010G163100 Sapur.014G049800

				Sapur.014G103300 Sapur.016G074400 Sapur.016G130500 Sapur.016G240600 Sapur.016G246100 Sapur.017G049800
GO:0048193	18	47	Golgi vesicle transport	Sapur.001G037800 Sapur.002G046500 Sapur.002G053800 Sapur.002G118400 Sapur.002G138300 Sapur.003G137200 Sapur.003G139000 Sapur.004G034200 Sapur.005G160500 Sapur.009G054300 Sapur.009G109200 Sapur.011G041500 Sapur.011G115500 Sapur.012G043900 Sapur.014G048500 Sapur.15WG081100 Sapur.15ZG059400 Sapur.T080400
GO:0051258	9	15	protein polymerization	Sapur.002G146800 Sapur.005G156700 Sapur.008G139000 Sapur.010G044700 Sapur.013G017100 Sapur.014G034600 Sapur.014G114900 Sapur.017G045900 Sapur.018G096500
GO:0044087	7	10	regulation of cellular component biogenesis	Sapur.005G156700 Sapur.008G139000 Sapur.010G044700 Sapur.013G017100 Sapur.014G034600 Sapur.014G114900 Sapur.018G096500
GO:0032271	7	10	regulation of protein polymerization	Sapur.005G156700 Sapur.008G139000 Sapur.010G044700 Sapur.013G017100 Sapur.014G034600 Sapur.014G114900 Sapur.018G096500
GO:0110053	7	10	regulation of actin filament organization	Sapur.005G156700 Sapur.008G139000 Sapur.010G044700 Sapur.013G017100 Sapur.014G034600 Sapur.014G114900 Sapur.018G096500
GO:0008064	7	10	regulation of actin polymerization or depolymerization	Sapur.005G156700 Sapur.008G139000 Sapur.010G044700 Sapur.013G017100 Sapur.014G034600 Sapur.014G114900 Sapur.018G096500
GO:0032956	7	10	regulation of actin cytoskeleton organization	Sapur.005G156700 Sapur.008G139000 Sapur.010G044700 Sapur.013G017100 Sapur.014G034600 Sapur.014G114900 Sapur.018G096500
GO:0032970	7	10	regulation of actin filament-based process	Sapur.005G156700 Sapur.008G139000 Sapur.010G044700 Sapur.013G017100 Sapur.014G034600 Sapur.014G114900 Sapur.018G096500
GO:0043254	7	10	regulation of protein-containing complex assembly	Sapur.005G156700 Sapur.008G139000 Sapur.010G044700 Sapur.013G017100 Sapur.014G034600 Sapur.014G114900 Sapur.018G096500

GO:1902903	7	10	regulation of supramolecular fiber organization	Sapur.005G156700 Sapur.008G139000 Sapur.010G044700 Sapur.013G017100 Sapur.014G034600 Sapur.014G114900 Sapur.018G096500
GO:0030832	7	10	regulation of actin filament length	Sapur.005G156700 Sapur.008G139000 Sapur.010G044700 Sapur.013G017100 Sapur.014G034600 Sapur.014G114900 Sapur.018G096500
GO:0030833	7	10	regulation of actin filament polymerization	Sapur.005G156700 Sapur.008G139000 Sapur.010G044700 Sapur.013G017100 Sapur.014G034600 Sapur.014G114900 Sapur.018G096500
GO:0006487	7	10	protein N-linked glycosylation	Sapur.002G028200 Sapur.005G101600 Sapur.005G180500 Sapur.006G054000 Sapur.007G028100 Sapur.008G149700 Sapur.010G023600
GO:0070972	16	41	protein localization to endoplasmic reticulum	Sapur.002G016500 Sapur.004G050600 Sapur.004G126600 Sapur.005G174600 Sapur.006G150200 Sapur.009G102500 Sapur.012G030400 Sapur.016G192300 Sapur.017G044400 Sapur.017G044500 Sapur.017G119100 Sapur.018G081000 Sapur.15ZG029200 Sapur.15ZG029900 Sapur.15ZG031600 Sapur.15ZG032200
GO:0008154	12	26	actin polymerization or depolymerization	Sapur.004G130100 Sapur.005G156700 Sapur.008G139000 Sapur.010G044700 Sapur.010G164300 Sapur.013G017100 Sapur.014G034600 Sapur.014G114900 Sapur.016G129900 Sapur.016G130000 Sapur.017G045900 Sapur.018G096500
GO:0045454	34	122	cell redox homeostasis	Sapur.001G150900 Sapur.002G065500 Sapur.002G161300 Sapur.002G197600 Sapur.003G153700 Sapur.004G006900 Sapur.005G127900 Sapur.005G185800 Sapur.006G030600 Sapur.006G124800 Sapur.006G164600 Sapur.007G015200 Sapur.007G121300 Sapur.008G079500 Sapur.008G102400 Sapur.008G116700 Sapur.009G001900 Sapur.009G008200 Sapur.009G059000 Sapur.009G078700 Sapur.010G088200 Sapur.011G073300 Sapur.011G100000 Sapur.013G053100 Sapur.013G104000 Sapur.014G098300 Sapur.014G130300 Sapur.016G034600 Sapur.016G165000 Sapur.016G277000 Sapur.017G067800 Sapur.018G104600 Sapur.019G035100 Sapur.019G080100
GO:0006790	18	50	sulfur compound metabolic process	Sapur.001G050000 Sapur.002G168400 Sapur.003G123600 Sapur.004G144000 Sapur.005G067900 Sapur.006G101400 Sapur.007G074100 Sapur.008G052000 Sapur.008G078200 Sapur.009G120400 Sapur.010G119600 Sapur.013G056900 Sapur.014G091500 Sapur.017G000100 Sapur.019G038000 Sapur.019G038200 Sapur.019G098500 Sapur.T140300
GO:0046500	5	6	S-adenosylmethionine metabolic process	Sapur.002G168400 Sapur.006G101400 Sapur.008G078200 Sapur.010G119600 Sapur.014G091500
GO:000	5	6	S-adenosylmethio	Sapur.002G168400 Sapur.006G101400 Sapur.008G078200 Sapur.010G119600 Sapur.014G091500

6556			nine biosynthetic process	
GO:0019236	4	4	response to pheromone	Sapur.001G067000 Sapur.003G109500 Sapur.006G176100 Sapur.016G076200
GO:0006891	4	4	intra-Golgi vesicle-mediated transport	Sapur.002G046500 Sapur.005G160500 Sapur.012G043900 Sapur.T080400
GO:0045185	6	9	maintenance of protein location	Sapur.002G016500 Sapur.004G126600 Sapur.009G102500 Sapur.016G192300 Sapur.017G044400 Sapur.017G044500
GO:0051235	6	9	maintenance of location	Sapur.002G016500 Sapur.004G126600 Sapur.009G102500 Sapur.016G192300 Sapur.017G044400 Sapur.017G044500
GO:0072595	6	9	maintenance of protein localization in organelle	Sapur.002G016500 Sapur.004G126600 Sapur.009G102500 Sapur.016G192300 Sapur.017G044400 Sapur.017G044500
GO:0035437	6	9	maintenance of protein localization in endoplasmic reticulum	Sapur.002G016500 Sapur.004G126600 Sapur.009G102500 Sapur.016G192300 Sapur.017G044400 Sapur.017G044500
GO:0051651	6	9	maintenance of location in cell	Sapur.002G016500 Sapur.004G126600 Sapur.009G102500 Sapur.016G192300 Sapur.017G044400 Sapur.017G044500
GO:0032507	6	9	maintenance of protein location in cell	Sapur.002G016500 Sapur.004G126600 Sapur.009G102500 Sapur.016G192300 Sapur.017G044400 Sapur.017G044500
GO:0006621	6	9	protein retention in ER lumen	Sapur.002G016500 Sapur.004G126600 Sapur.009G102500 Sapur.016G192300 Sapur.017G044400 Sapur.017G044500
GO:0042592	35	140	homeostatic process	Sapur.001G150900 Sapur.001G161400 Sapur.002G065500 Sapur.002G161300 Sapur.002G197600 Sapur.003G153700 Sapur.004G006900 Sapur.005G127900 Sapur.005G185800 Sapur.006G030600 Sapur.006G124800 Sapur.006G164600 Sapur.007G015200 Sapur.007G121300 Sapur.008G079500 Sapur.008G102400 Sapur.008G116700 Sapur.009G001900 Sapur.009G008200 Sapur.009G059000 Sapur.009G078700 Sapur.010G088200 Sapur.011G073300 Sapur.011G100000 Sapur.013G053100 Sapur.013G104000 Sapur.014G098300 Sapur.014G130300 Sapur.016G034600 Sapur.016G165000 Sapur.016G277000 Sapur.017G067800 Sapur.018G104600 Sapur.019G035100 Sapur.019G080100

GO:0019725	35	140	cellular homeostasis	Sapur.001G150900 Sapur.001G161400 Sapur.002G065500 Sapur.002G161300 Sapur.002G197600 Sapur.003G153700 Sapur.004G006900 Sapur.005G127900 Sapur.005G185800 Sapur.006G030600 Sapur.006G124800 Sapur.006G164600 Sapur.007G015200 Sapur.007G121300 Sapur.008G079500 Sapur.008G102400 Sapur.008G116700 Sapur.009G001900 Sapur.009G008200 Sapur.009G059000 Sapur.009G078700 Sapur.010G088200 Sapur.011G073300 Sapur.011G100000 Sapur.013G053100 Sapur.013G104000 Sapur.014G098300 Sapur.014G130300 Sapur.016G034600 Sapur.016G165000 Sapur.016G277000 Sapur.017G067800 Sapur.018G104600 Sapur.019G035100 Sapur.019G080100
GO:0008643	5	7	carbohydrate transport	Sapur.003G100300 Sapur.005G058800 Sapur.016G098500 Sapur.017G027100 Sapur.010G079100
GO:0090066	7	13	regulation of anatomical structure size	Sapur.005G156700 Sapur.008G139000 Sapur.010G044700 Sapur.013G017100 Sapur.014G034600 Sapur.014G114900 Sapur.018G096500
GO:0009066	7	13	aspartate family amino acid metabolic process	Sapur.004G144000 Sapur.009G120400 Sapur.013G056900 Sapur.017G000100 Sapur.019G038000 Sapur.019G038200 Sapur.T140300
GO:0032535	7	13	regulation of cellular component size	Sapur.005G156700 Sapur.008G139000 Sapur.010G044700 Sapur.013G017100 Sapur.014G034600 Sapur.014G114900 Sapur.018G096500
GO:0022900	9	20	electron transport chain	Sapur.001G187900 Sapur.002G054000 Sapur.003G020000 Sapur.004G050300 Sapur.004G142200 Sapur.005G150200 Sapur.005G150300 Sapur.007G003500 Sapur.009G118300
GO:0008610	43	186	lipid biosynthetic process	Sapur.001G026000 Sapur.001G095200 Sapur.002G002500 Sapur.002G011200 Sapur.002G103600 Sapur.002G119700 Sapur.002G157000 Sapur.002G189800 Sapur.003G060700 Sapur.003G062100 Sapur.003G079300 Sapur.004G098500 Sapur.004G114100 Sapur.004G114400 Sapur.004G116800 Sapur.005G067900 Sapur.005G157000 Sapur.006G131700 Sapur.006G175000 Sapur.006G206200 Sapur.007G074100 Sapur.008G041000 Sapur.009G019400 Sapur.009G044900 Sapur.010G163100 Sapur.011G119400 Sapur.013G013900 Sapur.013G024200 Sapur.014G049800 Sapur.014G103300 Sapur.016G074400 Sapur.016G093100 Sapur.016G127900 Sapur.016G130500 Sapur.016G240600 Sapur.016G246100 Sapur.017G049800 Sapur.017G096500 Sapur.018G026500 Sapur.018G053800 Sapur.019G022400 Sapur.019G098500 Sapur.T049800
GO:0070925	9	21	organelle assembly	Sapur.002G174200 Sapur.002G176700 Sapur.003G076300 Sapur.004G007800 Sapur.011G003200 Sapur.012G067400 Sapur.014G128900 Sapur.017G082000 Sapur.T001400
GO:0065008	7	14	regulation of biological quality	Sapur.005G156700 Sapur.008G139000 Sapur.010G044700 Sapur.013G017100 Sapur.014G034600 Sapur.014G114900 Sapur.018G096500
GO:000	7	14	sulfur amino acid	Sapur.004G144000 Sapur.009G120400 Sapur.013G056900 Sapur.017G000100 Sapur.019G038000

0096			metabolic process	Sapur.019G038200 Sapur.T140300
GO:0006013	4	5	mannose metabolic process	Sapur.002G184100 Sapur.007G051800 Sapur.014G116700 Sapur.016G222900
GO:0006629	82	408	lipid metabolic process	Sapur.001G026000 Sapur.001G074000 Sapur.001G095200 Sapur.001G175000 Sapur.002G002500 Sapur.002G011200 Sapur.002G103600 Sapur.002G119700 Sapur.002G148800 Sapur.002G157000 Sapur.002G161700 Sapur.002G189800 Sapur.003G060700 Sapur.003G062100 Sapur.003G079300 Sapur.003G104000 Sapur.004G005000 Sapur.004G011400 Sapur.004G061700 Sapur.004G098500 Sapur.004G114100 Sapur.004G114400 Sapur.004G116800 Sapur.004G167700 Sapur.005G001600 Sapur.005G067900 Sapur.005G157000 Sapur.006G056800 Sapur.006G087900 Sapur.006G123700 Sapur.006G131700 Sapur.006G175000 Sapur.006G188100 Sapur.006G206200 Sapur.007G074100 Sapur.008G007500 Sapur.008G041000 Sapur.008G043100 Sapur.008G160200 Sapur.009G019400 Sapur.009G044900 Sapur.009G074400 Sapur.009G099500 Sapur.010G011100 Sapur.010G160600 Sapur.010G163100 Sapur.011G006500 Sapur.011G034900 Sapur.011G044300 Sapur.011G112300 Sapur.011G119400 Sapur.012G093300 Sapur.013G013900 Sapur.013G024200 Sapur.013G091800 Sapur.014G016700 Sapur.014G049800 Sapur.014G103300 Sapur.016G041700 Sapur.016G074400 Sapur.016G093100 Sapur.016G096000 Sapur.016G127900 Sapur.016G130500 Sapur.016G193400 Sapur.016G212300 Sapur.016G228400 Sapur.016G240600 Sapur.016G246100 Sapur.017G046000 Sapur.017G049800 Sapur.017G096500 Sapur.018G026500 Sapur.018G053800 Sapur.018G103200 Sapur.019G022400 Sapur.019G098500 Sapur.15WG071900 Sapur.15ZG115800 Sapur.T004200 Sapur.T049800 Sapur.T052100
GO:0022904	6	11	respiratory electron transport chain	Sapur.002G054000 Sapur.004G050300 Sapur.004G142200 Sapur.005G150200 Sapur.005G150300 Sapur.009G118300
GO:0042775	5	8	mitochondrial ATP synthesis coupled electron transport	Sapur.002G054000 Sapur.004G142200 Sapur.005G150200 Sapur.005G150300 Sapur.009G118300
GO:0006122	5	8	mitochondrial electron transport, ubiquinol to cytochrome c	Sapur.002G054000 Sapur.004G142200 Sapur.005G150200 Sapur.005G150300 Sapur.009G118300
GO:0019646	5	8	aerobic electron transport chain	Sapur.002G054000 Sapur.004G142200 Sapur.005G150200 Sapur.005G150300 Sapur.009G118300
GO:0009071	3	3	serine family amino acid	Sapur.004G005600 Sapur.006G189300 Sapur.011G005900

			catabolic process	
GO:0006546	3	3	glycine catabolic process	Sapur.004G005600 Sapur.006G189300 Sapur.011G005900
GO:0016255	3	3	attachment of GPI anchor to protein	Sapur.002G189800 Sapur.019G022400 Sapur.T049800
GO:0009067	6	12	aspartate family amino acid biosynthetic process	Sapur.004G144000 Sapur.009G120400 Sapur.013G056900 Sapur.019G038000 Sapur.019G038200 Sapur.T140300
GO:0006505	7	16	GPI anchor metabolic process	Sapur.001G026000 Sapur.002G189800 Sapur.003G060700 Sapur.003G062100 Sapur.016G212300 Sapur.019G022400 Sapur.T049800
GO:0042773	5	9	ATP synthesis coupled electron transport	Sapur.002G054000 Sapur.004G142200 Sapur.005G150200 Sapur.005G150300 Sapur.009G118300
GO:0006119	5	9	oxidative phosphorylation	Sapur.002G054000 Sapur.004G142200 Sapur.005G150200 Sapur.005G150300 Sapur.009G118300
GO:0005975	101	538	carbohydrate metabolic process	Sapur.001G053200 Sapur.001G072900 Sapur.001G112100 Sapur.001G161400 Sapur.001G167900 Sapur.001G171300 Sapur.002G001800 Sapur.002G017900 Sapur.002G064200 Sapur.002G075000 Sapur.002G125200 Sapur.002G147500 Sapur.002G162300 Sapur.002G182500 Sapur.002G184100 Sapur.003G013400 Sapur.003G022500 Sapur.003G024100 Sapur.003G063600 Sapur.003G112400 Sapur.003G118000 Sapur.003G119700 Sapur.003G165600 Sapur.004G011800 Sapur.004G012100 Sapur.004G129600 Sapur.004G152100 Sapur.005G052600 Sapur.005G118500 Sapur.005G190700 Sapur.005G206700 Sapur.006G001400 Sapur.006G090000 Sapur.006G119400 Sapur.006G126300 Sapur.006G135500 Sapur.006G180700 Sapur.006G194700 Sapur.007G051800 Sapur.007G064600 Sapur.007G089900 Sapur.007G090100 Sapur.008G038800 Sapur.008G043700 Sapur.008G043800 Sapur.008G062900 Sapur.008G076700 Sapur.008G112200 Sapur.008G152200 Sapur.009G003700 Sapur.009G009100 Sapur.009G014300 Sapur.009G065500 Sapur.009G068900 Sapur.009G120900 Sapur.009G128100 Sapur.010G021300 Sapur.010G028000 Sapur.010G071500 Sapur.010G082200 Sapur.010G082500 Sapur.010G110600 Sapur.010G160100 Sapur.010G167500 Sapur.011G002500 Sapur.011G009700 Sapur.011G072100 Sapur.011G112600 Sapur.011G118500 Sapur.012G030800 Sapur.012G033500 Sapur.013G005200 Sapur.013G117800 Sapur.013G119000 Sapur.014G072000 Sapur.014G091800 Sapur.014G097700 Sapur.014G116700 Sapur.014G136300 Sapur.016G000600 Sapur.016G045800 Sapur.016G054400 Sapur.016G054600 Sapur.016G061400 Sapur.016G206300 Sapur.016G222900 Sapur.016G287300 Sapur.016G289400 Sapur.016G301100 Sapur.017G046300 Sapur.018G061200 Sapur.018G066500 Sapur.018G086200 Sapur.018G101200 Sapur.019G037200 Sapur.019G109000 Sapur.15ZG118900 Sapur.T191600 Sapur.006G112700 Sapur.018G045900 Sapur.016G023600

GO:0000097	6	13	sulfur amino acid biosynthetic process	Sapur.004G144000 Sapur.009G120400 Sapur.013G056900 Sapur.019G038000 Sapur.019G038200 Sapur.T140300
GO:0006506	6	13	GPI anchor biosynthetic process	Sapur.001G026000 Sapur.002G189800 Sapur.003G060700 Sapur.003G062100 Sapur.019G022400 Sapur.T049800
GO:0015693	6	13	magnesium ion transport	Sapur.003G005100 Sapur.006G186200 Sapur.009G036200 Sapur.013G105800 Sapur.014G113300 Sapur.019G082100
GO:0051493	8	21	regulation of cytoskeleton organization	Sapur.005G156700 Sapur.008G139000 Sapur.010G044700 Sapur.013G017100 Sapur.014G034600 Sapur.014G114900 Sapur.017G125500 Sapur.018G096500
GO:0051668	14	48	localization within membrane	Sapur.002G053800 Sapur.004G050600 Sapur.005G080800 Sapur.005G174600 Sapur.006G059100 Sapur.006G150200 Sapur.012G030400 Sapur.016G204500 Sapur.017G119100 Sapur.018G081000 Sapur.15ZG029200 Sapur.15ZG029900 Sapur.15ZG031600 Sapur.15ZG032200
GO:0055085	124	681	transmembrane transport	Sapur.001G033600 Sapur.001G079000 Sapur.001G164900 Sapur.001G185600 Sapur.002G011100 Sapur.002G038200 Sapur.002G056800 Sapur.002G067400 Sapur.002G086000 Sapur.002G110800 Sapur.002G133400 Sapur.003G014800 Sapur.003G029600 Sapur.003G061600 Sapur.003G082500 Sapur.003G100300 Sapur.003G107900 Sapur.003G138600 Sapur.004G020800 Sapur.004G021600 Sapur.004G025800 Sapur.004G048700 Sapur.004G050600 Sapur.004G076400 Sapur.004G141700 Sapur.004G155400 Sapur.004G162900 Sapur.004G168100 Sapur.004G168900 Sapur.004G172600 Sapur.005G031200 Sapur.005G031500 Sapur.005G034100 Sapur.005G121300 Sapur.005G138700 Sapur.005G172700 Sapur.005G204400 Sapur.006G004700 Sapur.006G009900 Sapur.006G034400 Sapur.006G049300 Sapur.006G062900 Sapur.006G084000 Sapur.006G102100 Sapur.006G190100 Sapur.007G021700 Sapur.007G027700 Sapur.007G051300 Sapur.008G005900 Sapur.008G015200 Sapur.008G050300 Sapur.008G104200 Sapur.008G105500 Sapur.008G113200 Sapur.008G126700 Sapur.008G162700 Sapur.009G003300 Sapur.009G006300 Sapur.009G062200 Sapur.009G062300 Sapur.009G068000 Sapur.009G090000 Sapur.009G117600 Sapur.009G131800 Sapur.010G007300 Sapur.010G014600 Sapur.010G048300 Sapur.010G085400 Sapur.010G087800 Sapur.010G090500 Sapur.010G133600 Sapur.010G151900 Sapur.011G107600 Sapur.012G007100 Sapur.012G009800 Sapur.012G025200 Sapur.012G071300 Sapur.012G100200 Sapur.013G068000 Sapur.013G080100 Sapur.013G090600 Sapur.013G100800 Sapur.014G083800 Sapur.014G089900 Sapur.014G102800 Sapur.014G104400 Sapur.014G123600 Sapur.016G040900 Sapur.016G047900 Sapur.016G096400 Sapur.016G097500 Sapur.016G098500 Sapur.016G138400 Sapur.016G147600 Sapur.016G175100 Sapur.016G202100 Sapur.016G220900 Sapur.016G226600 Sapur.016G231500 Sapur.016G231600 Sapur.016G239800 Sapur.016G242000 Sapur.016G295000 Sapur.016G295100 Sapur.016G307500 Sapur.016G310000 Sapur.017G056200 Sapur.017G067400 Sapur.017G073700 Sapur.017G095100 Sapur.017G119100 Sapur.018G060200 Sapur.018G089800

GO:0051604	36	163	protein maturation	Sapur.018G097400 Sapur.018G118800 Sapur.019G054200 Sapur.019G076600 Sapur.15WG078100 Sapur.15ZG073700 Sapur.15ZG123400 Sapur.T001500 Sapur.T047800 Sapur.006G116300 Sapur.006G180200 Sapur.001G124800 Sapur.001G146600 Sapur.002G150900 Sapur.002G164400 Sapur.002G192700 Sapur.003G034800 Sapur.004G058500 Sapur.004G129800 Sapur.004G149500 Sapur.005G013100 Sapur.005G028800 Sapur.005G042300 Sapur.005G058100 Sapur.005G193300 Sapur.006G194300 Sapur.007G113300 Sapur.008G051200 Sapur.008G051300 Sapur.008G142600 Sapur.009G035100 Sapur.009G125500 Sapur.010G008100 Sapur.010G051000 Sapur.010G112900 Sapur.010G148200 Sapur.012G083500 Sapur.013G008900 Sapur.014G095000 Sapur.016G002900 Sapur.016G093100 Sapur.017G016100 Sapur.018G005200 Sapur.15ZG109000 Sapur.15ZG121900 Sapur.T017000 Sapur.T064000
GO:0051225	4	7	spindle assembly	Sapur.002G174200 Sapur.014G128900 Sapur.017G082000 Sapur.T001400
GO:0006643	10	31	membrane lipid metabolic process	Sapur.001G026000 Sapur.001G175000 Sapur.002G189800 Sapur.003G060700 Sapur.003G062100 Sapur.016G193400 Sapur.016G212300 Sapur.017G046000 Sapur.019G022400 Sapur.T049800
GO:0090150	13	46	establishment of protein localization to membrane	Sapur.004G050600 Sapur.005G080800 Sapur.005G174600 Sapur.006G059100 Sapur.006G150200 Sapur.012G030400 Sapur.016G204500 Sapur.017G119100 Sapur.018G081000 Sapur.15ZG029200 Sapur.15ZG029900 Sapur.15ZG031600 Sapur.15ZG032200
GO:0072657	13	46	protein localization to membrane	Sapur.004G050600 Sapur.005G080800 Sapur.005G174600 Sapur.006G059100 Sapur.006G150200 Sapur.012G030400 Sapur.016G204500 Sapur.017G119100 Sapur.018G081000 Sapur.15ZG029200 Sapur.15ZG029900 Sapur.15ZG031600 Sapur.15ZG032200
GO:0072599	10	32	establishment of protein localization to endoplasmic reticulum	Sapur.004G050600 Sapur.005G174600 Sapur.006G150200 Sapur.012G030400 Sapur.017G119100 Sapur.018G081000 Sapur.15ZG029200 Sapur.15ZG029900 Sapur.15ZG031600 Sapur.15ZG032200
GO:0045047	10	32	protein targeting to ER	Sapur.004G050600 Sapur.005G174600 Sapur.006G150200 Sapur.012G030400 Sapur.017G119100 Sapur.018G081000 Sapur.15ZG029200 Sapur.15ZG029900 Sapur.15ZG031600 Sapur.15ZG032200
GO:0006612	10	32	protein targeting to membrane	Sapur.004G050600 Sapur.005G174600 Sapur.006G150200 Sapur.012G030400 Sapur.017G119100 Sapur.018G081000 Sapur.15ZG029200 Sapur.15ZG029900 Sapur.15ZG031600 Sapur.15ZG032200
GO:0036211	333	2018	protein modification process	Sapur.001G015800 Sapur.001G026000 Sapur.001G033500 Sapur.001G042400 Sapur.001G043100 Sapur.001G066800 Sapur.001G078000 Sapur.001G086800 Sapur.001G093700 Sapur.001G103900 Sapur.001G124800 Sapur.001G137500 Sapur.001G148700 Sapur.001G171200 Sapur.001G175900 Sapur.001G190300 Sapur.001G191000 Sapur.002G004300 Sapur.002G004900 Sapur.002G010500 Sapur.002G028000 Sapur.002G028200 Sapur.002G046900

	Sapur.002G055700 Sapur.002G072000 Sapur.002G106900 Sapur.002G120400 Sapur.002G164000 Sapur.002G176000
	Sapur.002G176800 Sapur.002G180800 Sapur.002G188100 Sapur.002G189800 Sapur.002G192000 Sapur.002G198100
	Sapur.002G199800 Sapur.002G201200 Sapur.002G201300 Sapur.003G010200 Sapur.003G013500 Sapur.003G037300
	Sapur.003G042700 Sapur.003G051100 Sapur.003G052500 Sapur.003G060700 Sapur.003G061800 Sapur.003G062100
	Sapur.003G067600 Sapur.003G073200 Sapur.003G075500 Sapur.003G082200 Sapur.003G085300 Sapur.003G089700
	Sapur.003G095700 Sapur.003G099600 Sapur.003G099700 Sapur.003G131900 Sapur.003G133000 Sapur.003G138700
	Sapur.004G009000 Sapur.004G017100 Sapur.004G023900 Sapur.004G029200 Sapur.004G032900 Sapur.004G033000
	Sapur.004G044400 Sapur.004G046500 Sapur.004G057300 Sapur.004G061000 Sapur.004G067800 Sapur.004G068600
	Sapur.004G080700 Sapur.004G087900 Sapur.004G115800 Sapur.004G129800 Sapur.004G135100 Sapur.004G138500
	Sapur.004G155200 Sapur.004G168300 Sapur.004G170800 Sapur.004G172200 Sapur.004G177200 Sapur.005G001800
	Sapur.005G011700 Sapur.005G015300 Sapur.005G021200 Sapur.005G031100 Sapur.005G039800 Sapur.005G050800
	Sapur.005G057200 Sapur.005G065000 Sapur.005G065300 Sapur.005G081600 Sapur.005G101600 Sapur.005G109200
	Sapur.005G143900 Sapur.005G148400 Sapur.005G160000 Sapur.005G169900 Sapur.005G180500 Sapur.005G202000
	Sapur.005G203300 Sapur.005G203500 Sapur.005G204000 Sapur.005G205600 Sapur.005G208100 Sapur.006G008700
	Sapur.006G039400 Sapur.006G043700 Sapur.006G044000 Sapur.006G054000 Sapur.006G059800 Sapur.006G063800
	Sapur.006G064600 Sapur.006G069300 Sapur.006G077000 Sapur.006G085900 Sapur.006G088300 Sapur.006G090100
	Sapur.006G093700 Sapur.006G096300 Sapur.006G107000 Sapur.006G117000 Sapur.006G151400 Sapur.006G185100
	Sapur.006G195000 Sapur.006G196200 Sapur.006G210500 Sapur.006G212700 Sapur.006G215000 Sapur.006G217500
	Sapur.007G003100 Sapur.007G012300 Sapur.007G028100 Sapur.007G037800 Sapur.007G044000 Sapur.007G045200
	Sapur.007G054000 Sapur.007G077400 Sapur.007G085900 Sapur.007G094200 Sapur.007G099400 Sapur.007G124000
	Sapur.007G124100 Sapur.008G008800 Sapur.008G016100 Sapur.008G028500 Sapur.008G054200 Sapur.008G054500
	Sapur.008G076100 Sapur.008G089200 Sapur.008G103500 Sapur.008G107400 Sapur.008G111800 Sapur.008G113700
	Sapur.008G116800 Sapur.008G120900 Sapur.008G136200 Sapur.008G136400 Sapur.008G143200 Sapur.008G149700
	Sapur.008G165900 Sapur.008G166200 Sapur.009G030800 Sapur.009G035100 Sapur.009G058900 Sapur.009G059300
	Sapur.009G079300 Sapur.009G091500 Sapur.009G092000 Sapur.009G107300 Sapur.010G000800 Sapur.010G008100
	Sapur.010G023600 Sapur.010G028600 Sapur.010G041500 Sapur.010G052000 Sapur.010G064500 Sapur.010G070900
	Sapur.010G075700 Sapur.010G076000 Sapur.010G076900 Sapur.010G112900 Sapur.010G118000 Sapur.010G118100
	Sapur.010G127100 Sapur.010G129800 Sapur.010G145100 Sapur.010G147600 Sapur.010G147800 Sapur.010G148200
	Sapur.010G169400 Sapur.010G170600 Sapur.010G178800 Sapur.010G189000 Sapur.011G021000 Sapur.011G030700
	Sapur.011G036900 Sapur.011G040500 Sapur.011G040600 Sapur.011G046900 Sapur.011G047800 Sapur.011G050200
	Sapur.011G050900 Sapur.011G080000 Sapur.011G080400 Sapur.011G080600 Sapur.011G089900 Sapur.011G095700
	Sapur.011G097200 Sapur.011G101300 Sapur.011G108300 Sapur.011G112000 Sapur.011G121500 Sapur.012G020400
	Sapur.012G029700 Sapur.012G032600 Sapur.012G041400 Sapur.012G048500 Sapur.012G051500 Sapur.012G052000

				Sapur.012G052600 Sapur.012G063200 Sapur.012G064400 Sapur.012G064900 Sapur.012G093400 Sapur.012G097700 Sapur.012G106400 Sapur.012G106800 Sapur.013G003400 Sapur.013G010500 Sapur.013G034500 Sapur.013G055800 Sapur.013G108700 Sapur.013G141900 Sapur.014G014200 Sapur.014G017800 Sapur.014G034800 Sapur.014G050200 Sapur.014G054500 Sapur.014G065600 Sapur.014G067100 Sapur.014G067400 Sapur.014G069000 Sapur.014G084200 Sapur.014G091100 Sapur.014G095200 Sapur.014G110500 Sapur.014G118100 Sapur.014G119600 Sapur.014G125100 Sapur.014G126200 Sapur.016G010900 Sapur.016G045300 Sapur.016G046200 Sapur.016G048600 Sapur.016G049500 Sapur.016G064900 Sapur.016G090700 Sapur.016G113500 Sapur.016G115900 Sapur.016G122900 Sapur.016G132100 Sapur.016G133200 Sapur.016G164800 Sapur.016G165400 Sapur.016G222400 Sapur.016G252400 Sapur.016G256600 Sapur.016G263500 Sapur.016G267500 Sapur.016G267900 Sapur.016G269800 Sapur.016G272100 Sapur.016G274100 Sapur.016G290200 Sapur.016G293800 Sapur.016G294500 Sapur.016G304300 Sapur.017G001800 Sapur.017G002000 Sapur.017G019000 Sapur.017G019900 Sapur.017G072700 Sapur.017G096900 Sapur.017G097600 Sapur.017G107900 Sapur.017G111400 Sapur.017G114100 Sapur.017G125500 Sapur.018G013300 Sapur.018G018700 Sapur.018G035500 Sapur.018G048200 Sapur.018G053200 Sapur.018G058300 Sapur.018G060300 Sapur.018G062800 Sapur.018G067300 Sapur.018G072700 Sapur.018G082400 Sapur.018G086200 Sapur.018G107900 Sapur.018G114200 Sapur.019G008900 Sapur.019G022400 Sapur.019G061700 Sapur.019G062400 Sapur.019G079900 Sapur.019G083400 Sapur.019G099500 Sapur.019G113000 Sapur.15WG028200 Sapur.15WG031300 Sapur.15WG066000 Sapur.15WG079300 Sapur.15ZG018000 Sapur.15ZG018200 Sapur.15ZG021300 Sapur.15ZG027900 Sapur.15ZG035300 Sapur.15ZG054200 Sapur.15ZG082200 Sapur.15ZG085600 Sapur.15ZG093000 Sapur.15ZG101800 Sapur.15ZG102900 Sapur.15ZG103100 Sapur.15ZG124400 Sapur.15ZG134100 Sapur.T044700 Sapur.T049800 Sapur.T051500
GO:0051128	11	37	regulation of cellular component organization	Sapur.005G156700 Sapur.007G124000 Sapur.007G124100 Sapur.008G139000 Sapur.010G044700 Sapur.010G125800 Sapur.013G017100 Sapur.014G034600 Sapur.014G114900 Sapur.017G125500 Sapur.018G096500
GO:0042157	6	15	lipoprotein metabolic process	Sapur.001G026000 Sapur.002G189800 Sapur.003G060700 Sapur.003G062100 Sapur.019G022400 Sapur.T049800
GO:0042158	6	15	lipoprotein biosynthetic process	Sapur.001G026000 Sapur.002G189800 Sapur.003G060700 Sapur.003G062100 Sapur.019G022400 Sapur.T049800
GO:0006497	6	15	protein lipidation	Sapur.001G026000 Sapur.002G189800 Sapur.003G060700 Sapur.003G062100 Sapur.019G022400 Sapur.T049800
GO:0046394	28	126	carboxylic acid biosynthetic process	Sapur.001G095200 Sapur.004G098500 Sapur.004G116800 Sapur.004G144000 Sapur.005G067900 Sapur.005G073900 Sapur.005G157000 Sapur.006G131700 Sapur.006G206200 Sapur.007G074100 Sapur.009G019400 Sapur.009G120400 Sapur.011G119400 Sapur.012G016400 Sapur.013G013900 Sapur.013G056900 Sapur.016G032800

				Sapur.016G093100 Sapur.016G127900 Sapur.017G096500 Sapur.018G026500 Sapur.018G053800 Sapur.019G038000 Sapur.019G038200 Sapur.019G098500 Sapur.15WG027600 Sapur.15ZG027200 Sapur.T140300
GO:0016053	28	126	organic acid biosynthetic process	Sapur.001G095200 Sapur.004G098500 Sapur.004G116800 Sapur.004G144000 Sapur.005G067900 Sapur.005G073900 Sapur.005G157000 Sapur.006G131700 Sapur.006G206200 Sapur.007G074100 Sapur.009G019400 Sapur.009G120400 Sapur.011G119400 Sapur.012G016400 Sapur.013G013900 Sapur.013G056900 Sapur.016G032800 Sapur.016G093100 Sapur.016G127900 Sapur.017G096500 Sapur.018G026500 Sapur.018G053800 Sapur.019G038000 Sapur.019G038200 Sapur.019G098500 Sapur.15WG027600 Sapur.15ZG027200 Sapur.T140300
GO:0033043	10	33	regulation of organelle organization	Sapur.005G156700 Sapur.007G124000 Sapur.007G124100 Sapur.008G139000 Sapur.010G044700 Sapur.013G017100 Sapur.014G034600 Sapur.014G114900 Sapur.017G125500 Sapur.018G096500
GO:1901605	16	63	alpha-amino acid metabolic process	Sapur.004G144000 Sapur.005G073900 Sapur.009G120400 Sapur.012G016400 Sapur.013G056900 Sapur.016G032800 Sapur.016G248100 Sapur.017G000100 Sapur.019G038000 Sapur.019G038200 Sapur.15WG027600 Sapur.15ZG027200 Sapur.T140300 Sapur.004G005600 Sapur.006G189300 Sapur.011G005900
GO:1903509	7	20	liposaccharide metabolic process	Sapur.001G026000 Sapur.002G189800 Sapur.003G060700 Sapur.003G062100 Sapur.016G212300 Sapur.019G022400 Sapur.T049800
GO:0006664	7	20	glycolipid metabolic process	Sapur.001G026000 Sapur.002G189800 Sapur.003G060700 Sapur.003G062100 Sapur.016G212300 Sapur.019G022400 Sapur.T049800
GO:0007018	24	106	microtubule-based movement	Sapur.001G000500 Sapur.002G122000 Sapur.002G159800 Sapur.002G182100 Sapur.003G028400 Sapur.003G169700 Sapur.004G120900 Sapur.004G122400 Sapur.005G015200 Sapur.006G159000 Sapur.007G012500 Sapur.007G043900 Sapur.009G018700 Sapur.009G097000 Sapur.010G008800 Sapur.011G097800 Sapur.011G108500 Sapur.013G010400 Sapur.014G014400 Sapur.016G054000 Sapur.016G056900 Sapur.016G077400 Sapur.016G127100 Sapur.016G275100
GO:0035384	2	2	thioester biosynthetic process	Sapur.001G050000 Sapur.003G123600
GO:0051016	2	2	barbed-end actin filament capping	Sapur.005G156700 Sapur.013G017100
GO:1901880	2	2	negative regulation of protein depolymerization	Sapur.005G156700 Sapur.013G017100
GO:1901879	2	2	regulation of protein	Sapur.005G156700 Sapur.013G017100

			depolymerization	
GO:0032272	2	2	negative regulation of protein polymerization	Sapur.005G156700 Sapur.013G017100
GO:0031333	2	2	negative regulation of protein-containing complex assembly	Sapur.005G156700 Sapur.013G017100
GO:0006084	2	2	acetyl-CoA metabolic process	Sapur.001G050000 Sapur.003G123600
GO:0006085	2	2	acetyl-CoA biosynthetic process	Sapur.001G050000 Sapur.003G123600
GO:0006086	2	2	acetyl-CoA biosynthetic process from pyruvate	Sapur.001G050000 Sapur.003G123600
GO:0006730	2	2	one-carbon metabolic process	Sapur.016G195500 Sapur.017G050100
GO:0043242	2	2	negative regulation of protein-containing complex disassembly	Sapur.005G156700 Sapur.013G017100
GO:1902904	2	2	negative regulation of supramolecular fiber organization	Sapur.005G156700 Sapur.013G017100
GO:0051693	2	2	actin filament capping	Sapur.005G156700 Sapur.013G017100

GO:0007155	2	2	cell adhesion	Sapur.002G108400 Sapur.014G030900
GO:0051494	2	2	negative regulation of cytoskeleton organization	Sapur.005G156700 Sapur.013G017100
GO:0031023	2	2	microtubule organizing center organization	Sapur.002G174200 Sapur.014G128900
GO:0007029	2	2	endoplasmic reticulum organization	Sapur.005G080800 Sapur.012G098100
GO:0030834	2	2	regulation of actin filament depolymerization	Sapur.005G156700 Sapur.013G017100
GO:0030835	2	2	negative regulation of actin filament depolymerization	Sapur.005G156700 Sapur.013G017100
GO:0030837	2	2	negative regulation of actin filament polymerization	Sapur.005G156700 Sapur.013G017100
GO:0018279	2	2	protein N-linked glycosylation via asparagine	Sapur.005G101600 Sapur.007G028100
GO:0071616	2	2	acyl-CoA biosynthetic process	Sapur.001G050000 Sapur.003G123600
GO:0033365	21	90	protein localization to organelle	Sapur.002G016500 Sapur.004G050600 Sapur.004G126600 Sapur.005G080800 Sapur.005G174600 Sapur.006G062900 Sapur.006G150200 Sapur.009G102500 Sapur.012G030400 Sapur.014G099300 Sapur.014G123600 Sapur.016G192300 Sapur.016G204500 Sapur.017G044400 Sapur.017G044500 Sapur.017G119100 Sapur.018G081000 Sapur.15ZG029200 Sapur.15ZG029900 Sapur.15ZG031600 Sapur.15ZG032200

GO:0006996	51	260	organelle organization	Sapur.001G044900 Sapur.002G146800 Sapur.002G174200 Sapur.002G176700 Sapur.003G036900 Sapur.003G076300 Sapur.003G130500 Sapur.004G007800 Sapur.004G039300 Sapur.004G130100 Sapur.005G080800 Sapur.005G156700 Sapur.006G028600 Sapur.006G038100 Sapur.006G062900 Sapur.006G086600 Sapur.006G135400 Sapur.007G124000 Sapur.007G124100 Sapur.008G109800 Sapur.008G113100 Sapur.008G139000 Sapur.008G147700 Sapur.009G033900 Sapur.009G036100 Sapur.010G044700 Sapur.010G073200 Sapur.010G164300 Sapur.010G206600 Sapur.011G003200 Sapur.011G046000 Sapur.012G067400 Sapur.012G098100 Sapur.013G017100 Sapur.014G034600 Sapur.014G114900 Sapur.014G123600 Sapur.014G128900 Sapur.016G112500 Sapur.016G129900 Sapur.016G130000 Sapur.016G201500 Sapur.016G204500 Sapur.017G045900 Sapur.017G082000 Sapur.017G125500 Sapur.018G051900 Sapur.018G096500 Sapur.15ZG093800 Sapur.T001400 Sapur.15WG079100
GO:0140694	5	12	non-membrane-bounded organelle assembly	Sapur.002G174200 Sapur.012G067400 Sapur.014G128900 Sapur.017G082000 Sapur.T001400
GO:0072330	17	70	monocarboxylic acid biosynthetic process	Sapur.001G095200 Sapur.004G098500 Sapur.004G116800 Sapur.005G067900 Sapur.005G157000 Sapur.006G131700 Sapur.006G206200 Sapur.007G074100 Sapur.009G019400 Sapur.011G119400 Sapur.013G013900 Sapur.016G093100 Sapur.016G127900 Sapur.017G096500 Sapur.018G026500 Sapur.018G053800 Sapur.019G098500
GO:0006633	17	70	fatty acid biosynthetic process	Sapur.001G095200 Sapur.004G098500 Sapur.004G116800 Sapur.005G067900 Sapur.005G157000 Sapur.006G131700 Sapur.006G206200 Sapur.007G074100 Sapur.009G019400 Sapur.011G119400 Sapur.013G013900 Sapur.016G093100 Sapur.016G127900 Sapur.017G096500 Sapur.018G026500 Sapur.018G053800 Sapur.019G098500
GO:0009106	3	5	lipoate metabolic process	Sapur.005G067900 Sapur.007G074100 Sapur.019G098500
GO:0009107	3	5	lipoate biosynthetic process	Sapur.005G067900 Sapur.007G074100 Sapur.019G098500
GO:0015689	3	5	molybdate ion transport	Sapur.003G108000 Sapur.007G049600 Sapur.15ZG006400
GO:0006468	261	1578	protein phosphorylation	Sapur.001G015800 Sapur.001G033500 Sapur.001G042400 Sapur.001G043100 Sapur.001G066800 Sapur.001G078000 Sapur.001G086800 Sapur.001G093700 Sapur.001G103900 Sapur.001G137500 Sapur.001G148700 Sapur.001G171200 Sapur.001G175900 Sapur.001G190300 Sapur.001G191000 Sapur.002G010500 Sapur.002G028000 Sapur.002G046900 Sapur.002G055700 Sapur.002G106900 Sapur.002G120400 Sapur.002G164000 Sapur.002G176000 Sapur.002G180800 Sapur.002G188100 Sapur.002G192000 Sapur.002G198100 Sapur.002G199800 Sapur.002G201200 Sapur.002G201300 Sapur.003G013500 Sapur.003G037300 Sapur.003G042700 Sapur.003G051100 Sapur.003G052500 Sapur.003G061800 Sapur.003G067600 Sapur.003G073200 Sapur.003G075500 Sapur.003G082200 Sapur.003G085300 Sapur.003G089700 Sapur.003G095700 Sapur.003G099700 Sapur.003G131900 Sapur.003G133000 Sapur.003G138700

Sapur.004G009000	Sapur.004G017100	Sapur.004G023900	Sapur.004G029200	Sapur.004G032900	Sapur.004G033000
Sapur.004G044400	Sapur.004G046500	Sapur.004G057300	Sapur.004G061000	Sapur.004G067800	Sapur.004G068600
Sapur.004G080700	Sapur.004G087900	Sapur.004G115800	Sapur.004G135100	Sapur.004G170800	Sapur.004G172200
Sapur.004G177200	Sapur.005G001800	Sapur.005G011700	Sapur.005G021200	Sapur.005G031100	Sapur.005G039800
Sapur.005G050800	Sapur.005G057200	Sapur.005G065000	Sapur.005G065300	Sapur.005G081600	Sapur.005G109200
Sapur.005G143900	Sapur.005G148400	Sapur.005G160000	Sapur.005G202000	Sapur.005G203500	Sapur.005G205600
Sapur.005G208100	Sapur.006G039400	Sapur.006G043700	Sapur.006G044000	Sapur.006G059800	Sapur.006G063800
Sapur.006G064600	Sapur.006G077000	Sapur.006G090100	Sapur.006G093700	Sapur.006G096300	Sapur.006G107000
Sapur.006G117000	Sapur.006G151400	Sapur.006G185100	Sapur.006G195000	Sapur.006G196200	Sapur.006G210500
Sapur.006G215000	Sapur.006G217500	Sapur.007G003100	Sapur.007G012300	Sapur.007G037800	Sapur.007G044000
Sapur.007G054000	Sapur.007G077400	Sapur.007G085900	Sapur.007G124000	Sapur.007G124100	Sapur.008G008800
Sapur.008G016100	Sapur.008G054200	Sapur.008G054500	Sapur.008G076100	Sapur.008G089200	Sapur.008G107400
Sapur.008G111800	Sapur.008G113700	Sapur.008G116800	Sapur.008G136200	Sapur.008G136400	Sapur.008G143200
Sapur.008G165900	Sapur.009G030800	Sapur.009G058900	Sapur.009G059300	Sapur.009G091500	Sapur.009G092000
Sapur.009G107300	Sapur.010G000800	Sapur.010G028600	Sapur.010G041500	Sapur.010G052000	Sapur.010G064500
Sapur.010G070900	Sapur.010G075700	Sapur.010G076000	Sapur.010G076900	Sapur.010G127100	Sapur.010G145100
Sapur.010G147600	Sapur.010G147800	Sapur.010G169400	Sapur.010G189000	Sapur.011G021000	Sapur.011G030700
Sapur.011G036900	Sapur.011G040500	Sapur.011G040600	Sapur.011G046900	Sapur.011G047800	Sapur.011G050900
Sapur.011G080000	Sapur.011G080600	Sapur.011G089900	Sapur.011G095700	Sapur.011G097200	Sapur.011G101300
Sapur.011G108300	Sapur.011G121500	Sapur.012G020400	Sapur.012G029700	Sapur.012G032600	Sapur.012G041400
Sapur.012G048500	Sapur.012G051500	Sapur.012G052000	Sapur.012G063200	Sapur.012G064400	Sapur.012G064900
Sapur.012G093400	Sapur.012G097700	Sapur.012G106800	Sapur.013G003400	Sapur.013G034500	Sapur.013G055800
Sapur.013G108700	Sapur.013G141900	Sapur.014G014200	Sapur.014G017800	Sapur.014G050200	Sapur.014G054500
Sapur.014G065600	Sapur.014G067100	Sapur.014G067400	Sapur.014G069000	Sapur.014G084200	Sapur.014G091100
Sapur.014G095200	Sapur.014G110500	Sapur.014G119600	Sapur.014G126200	Sapur.016G045300	Sapur.016G046200
Sapur.016G048600	Sapur.016G049500	Sapur.016G064900	Sapur.016G090700	Sapur.016G113500	Sapur.016G115900
Sapur.016G122900	Sapur.016G133200	Sapur.016G164800	Sapur.016G165400	Sapur.016G222400	Sapur.016G256600
Sapur.016G263500	Sapur.016G267500	Sapur.016G267900	Sapur.016G269800	Sapur.016G272100	Sapur.016G274100
Sapur.016G293800	Sapur.016G294500	Sapur.016G304300	Sapur.017G001800	Sapur.017G002000	Sapur.017G019000
Sapur.017G072700	Sapur.017G096900	Sapur.017G097600	Sapur.017G107900	Sapur.017G111400	Sapur.017G114100
Sapur.017G125500	Sapur.018G018700	Sapur.018G035500	Sapur.018G048200	Sapur.018G053200	Sapur.018G058300
Sapur.018G060300	Sapur.018G067300	Sapur.018G072700	Sapur.018G082400	Sapur.018G086200	Sapur.018G107900
Sapur.018G114200	Sapur.019G008900	Sapur.019G061700	Sapur.019G062400	Sapur.019G083400	Sapur.019G099500

				Sapur.019G113000 Sapur.15WG028200 Sapur.15WG031300 Sapur.15WG066000 Sapur.15WG079300 Sapur.15ZG018000 Sapur.15ZG027900 Sapur.15ZG035300 Sapur.15ZG054200 Sapur.15ZG082200 Sapur.15ZG085600 Sapur.15ZG093000 Sapur.15ZG124400 Sapur.15ZG134100 Sapur.T044700 Sapur.T051500
GO:0019538	497	3116	protein metabolic process	Sapur.001G001400 Sapur.001G015800 Sapur.001G026000 Sapur.001G031800 Sapur.001G033500 Sapur.001G042400 Sapur.001G043100 Sapur.001G051400 Sapur.001G066800 Sapur.001G078000 Sapur.001G086800 Sapur.001G088500 Sapur.001G093700 Sapur.001G098400 Sapur.001G103900 Sapur.001G124800 Sapur.001G135000 Sapur.001G137500 Sapur.001G139800 Sapur.001G146600 Sapur.001G148700 Sapur.001G171200 Sapur.001G175900 Sapur.001G190300 Sapur.001G191000 Sapur.002G003700 Sapur.002G004300 Sapur.002G004900 Sapur.002G009400 Sapur.002G010500 Sapur.002G022300 Sapur.002G028000 Sapur.002G028200 Sapur.002G044500 Sapur.002G046900 Sapur.002G055700 Sapur.002G056400 Sapur.002G063400 Sapur.002G072000 Sapur.002G072700 Sapur.002G084000 Sapur.002G096700 Sapur.002G097300 Sapur.002G101500 Sapur.002G106900 Sapur.002G108400 Sapur.002G120400 Sapur.002G124000 Sapur.002G134700 Sapur.002G143900 Sapur.002G150900 Sapur.002G164000 Sapur.002G164400 Sapur.002G176000 Sapur.002G176800 Sapur.002G180800 Sapur.002G188000 Sapur.002G188100 Sapur.002G189800 Sapur.002G192000 Sapur.002G192700 Sapur.002G198100 Sapur.002G199800 Sapur.002G201200 Sapur.002G201300 Sapur.003G010200 Sapur.003G013500 Sapur.003G034800 Sapur.003G037300 Sapur.003G037900 Sapur.003G042700 Sapur.003G045000 Sapur.003G051100 Sapur.003G052500 Sapur.003G060700 Sapur.003G061800 Sapur.003G062100 Sapur.003G067600 Sapur.003G068500 Sapur.003G073200 Sapur.003G075500 Sapur.003G082200 Sapur.003G085300 Sapur.003G087600 Sapur.003G087900 Sapur.003G088100 Sapur.003G089700 Sapur.003G095700 Sapur.003G099600 Sapur.003G099700 Sapur.003G105400 Sapur.003G131900 Sapur.003G133000 Sapur.003G138700 Sapur.003G151400 Sapur.004G005000 Sapur.004G009000 Sapur.004G017100 Sapur.004G023900 Sapur.004G029200 Sapur.004G032900 Sapur.004G033000 Sapur.004G044400 Sapur.004G046500 Sapur.004G057300 Sapur.004G058500 Sapur.004G060000 Sapur.004G061000 Sapur.004G067800 Sapur.004G068600 Sapur.004G080700 Sapur.004G087900 Sapur.004G104700 Sapur.004G115800 Sapur.004G120300 Sapur.004G129800 Sapur.004G130500 Sapur.004G135100 Sapur.004G138500 Sapur.004G149500 Sapur.004G155200 Sapur.004G162500 Sapur.004G164900 Sapur.004G165000 Sapur.004G168300 Sapur.004G170800 Sapur.004G172200 Sapur.004G173100 Sapur.004G177200 Sapur.005G001600 Sapur.005G001800 Sapur.005G011700 Sapur.005G013100 Sapur.005G015300 Sapur.005G021200 Sapur.005G028800 Sapur.005G031100 Sapur.005G039800 Sapur.005G042300 Sapur.005G047600 Sapur.005G050800 Sapur.005G053000 Sapur.005G057200 Sapur.005G058100 Sapur.005G062300 Sapur.005G065000 Sapur.005G065300 Sapur.005G072700 Sapur.005G081600 Sapur.005G086400 Sapur.005G086600 Sapur.005G087800 Sapur.005G101600 Sapur.005G109200 Sapur.005G122400 Sapur.005G123400 Sapur.005G125000 Sapur.005G143900 Sapur.005G147300 Sapur.005G148400 Sapur.005G160000 Sapur.005G169900 Sapur.005G180500 Sapur.005G193300 Sapur.005G194900 Sapur.005G202000 Sapur.005G203300 Sapur.005G203500 Sapur.005G204000 Sapur.005G204800 Sapur.005G205600 Sapur.005G208100 Sapur.006G008700 Sapur.006G026100 Sapur.006G039400 Sapur.006G043700 Sapur.006G044000 Sapur.006G054000 Sapur.006G059800

	Sapur.006G061500 Sapur.006G063300 Sapur.006G063800 Sapur.006G064600 Sapur.006G069300 Sapur.006G070700
	Sapur.006G077000 Sapur.006G085900 Sapur.006G088300 Sapur.006G090100 Sapur.006G093700 Sapur.006G096300
	Sapur.006G107000 Sapur.006G115800 Sapur.006G117000 Sapur.006G151400 Sapur.006G169900 Sapur.006G185100
	Sapur.006G192500 Sapur.006G194300 Sapur.006G195000 Sapur.006G196200 Sapur.006G210500 Sapur.006G212700
	Sapur.006G215000 Sapur.006G217500 Sapur.007G003100 Sapur.007G010500 Sapur.007G012300 Sapur.007G028100
	Sapur.007G037800 Sapur.007G042300 Sapur.007G044000 Sapur.007G045200 Sapur.007G054000 Sapur.007G060300
	Sapur.007G066700 Sapur.007G077400 Sapur.007G085900 Sapur.007G094200 Sapur.007G095100 Sapur.007G099400
	Sapur.007G113300 Sapur.007G124000 Sapur.007G124100 Sapur.008G008800 Sapur.008G016100 Sapur.008G024600
	Sapur.008G028500 Sapur.008G031500 Sapur.008G045500 Sapur.008G051200 Sapur.008G051300 Sapur.008G052000
	Sapur.008G054200 Sapur.008G054500 Sapur.008G061000 Sapur.008G076100 Sapur.008G089200 Sapur.008G093300
	Sapur.008G103500 Sapur.008G107400 Sapur.008G111800 Sapur.008G113700 Sapur.008G116800 Sapur.008G120900
	Sapur.008G136200 Sapur.008G136400 Sapur.008G142600 Sapur.008G143200 Sapur.008G149700 Sapur.008G165900
	Sapur.008G166200 Sapur.009G000500 Sapur.009G001100 Sapur.009G023600 Sapur.009G024900 Sapur.009G030800
	Sapur.009G035100 Sapur.009G042700 Sapur.009G058900 Sapur.009G059300 Sapur.009G079300 Sapur.009G091500
	Sapur.009G092000 Sapur.009G096300 Sapur.009G106500 Sapur.009G107300 Sapur.009G125500 Sapur.009G126900
	Sapur.010G000800 Sapur.010G008100 Sapur.010G013800 Sapur.010G023600 Sapur.010G028600 Sapur.010G041500
	Sapur.010G045000 Sapur.010G051000 Sapur.010G052000 Sapur.010G064500 Sapur.010G070900 Sapur.010G075700
	Sapur.010G076000 Sapur.010G076900 Sapur.010G098600 Sapur.010G112900 Sapur.010G115800 Sapur.010G118000
	Sapur.010G118100 Sapur.010G125800 Sapur.010G127100 Sapur.010G129800 Sapur.010G145100 Sapur.010G147600
	Sapur.010G147800 Sapur.010G148200 Sapur.010G169400 Sapur.010G170600 Sapur.010G178800 Sapur.010G189000
	Sapur.011G006500 Sapur.011G021000 Sapur.011G030700 Sapur.011G036900 Sapur.011G040500 Sapur.011G040600
	Sapur.011G046900 Sapur.011G047800 Sapur.011G050200 Sapur.011G050900 Sapur.011G080000 Sapur.011G080400
	Sapur.011G080600 Sapur.011G089900 Sapur.011G095700 Sapur.011G097200 Sapur.011G101300 Sapur.011G108300
	Sapur.011G112000 Sapur.011G121500 Sapur.012G013000 Sapur.012G013500 Sapur.012G018300 Sapur.012G020400
	Sapur.012G029700 Sapur.012G032600 Sapur.012G041400 Sapur.012G048500 Sapur.012G051500 Sapur.012G052000
	Sapur.012G052600 Sapur.012G063200 Sapur.012G064400 Sapur.012G064900 Sapur.012G065200 Sapur.012G067800
	Sapur.012G080500 Sapur.012G083500 Sapur.012G086400 Sapur.012G093400 Sapur.012G097700 Sapur.012G101000
	Sapur.012G106400 Sapur.012G106800 Sapur.012G107200 Sapur.013G003400 Sapur.013G008900 Sapur.013G010500
	Sapur.013G034500 Sapur.013G055800 Sapur.013G056100 Sapur.013G091200 Sapur.013G108700 Sapur.013G141900
	Sapur.014G009000 Sapur.014G010900 Sapur.014G013900 Sapur.014G014200 Sapur.014G017800 Sapur.014G030900
	Sapur.014G034800 Sapur.014G039700 Sapur.014G043900 Sapur.014G050200 Sapur.014G054500 Sapur.014G065600
	Sapur.014G067100 Sapur.014G067400 Sapur.014G069000 Sapur.014G077700 Sapur.014G084200 Sapur.014G091100
	Sapur.014G095000 Sapur.014G095200 Sapur.014G096400 Sapur.014G110500 Sapur.014G118100 Sapur.014G119600

				Sapur.014G125100 Sapur.014G126200 Sapur.016G002900 Sapur.016G010900 Sapur.016G030700 Sapur.016G045300 Sapur.016G046200 Sapur.016G048600 Sapur.016G049500 Sapur.016G064900 Sapur.016G066000 Sapur.016G090700 Sapur.016G093100 Sapur.016G113500 Sapur.016G115900 Sapur.016G122900 Sapur.016G132100 Sapur.016G133200 Sapur.016G133500 Sapur.016G164800 Sapur.016G165400 Sapur.016G181700 Sapur.016G183900 Sapur.016G222400 Sapur.016G227000 Sapur.016G228400 Sapur.016G252400 Sapur.016G256600 Sapur.016G263500 Sapur.016G267500 Sapur.016G267900 Sapur.016G269800 Sapur.016G272100 Sapur.016G274100 Sapur.016G290200 Sapur.016G293800 Sapur.016G294500 Sapur.016G294800 Sapur.016G304300 Sapur.017G001800 Sapur.017G002000 Sapur.017G016100 Sapur.017G019000 Sapur.017G019900 Sapur.017G069700 Sapur.017G072700 Sapur.017G096900 Sapur.017G097600 Sapur.017G107900 Sapur.017G109200 Sapur.017G111400 Sapur.017G114100 Sapur.017G125500 Sapur.018G005200 Sapur.018G010500 Sapur.018G010800 Sapur.018G010900 Sapur.018G011100 Sapur.018G011200 Sapur.018G013300 Sapur.018G018700 Sapur.018G035500 Sapur.018G048200 Sapur.018G053200 Sapur.018G058300 Sapur.018G058400 Sapur.018G060300 Sapur.018G062800 Sapur.018G067300 Sapur.018G072700 Sapur.018G078800 Sapur.018G079700 Sapur.018G082400 Sapur.018G086200 Sapur.018G107900 Sapur.018G114200 Sapur.019G008900 Sapur.019G016700 Sapur.019G022400 Sapur.019G052800 Sapur.019G053400 Sapur.019G061700 Sapur.019G062400 Sapur.019G079900 Sapur.019G083400 Sapur.019G099500 Sapur.019G113000 Sapur.15WG028200 Sapur.15WG031300 Sapur.15WG066000 Sapur.15WG079300 Sapur.15ZG010600 Sapur.15ZG018000 Sapur.15ZG018200 Sapur.15ZG021300 Sapur.15ZG027900 Sapur.15ZG035300 Sapur.15ZG054200 Sapur.15ZG082200 Sapur.15ZG085600 Sapur.15ZG093000 Sapur.15ZG101800 Sapur.15ZG102400 Sapur.15ZG102900 Sapur.15ZG103100 Sapur.15ZG109000 Sapur.15ZG111000 Sapur.15ZG121900 Sapur.15ZG124400 Sapur.15ZG130400 Sapur.15ZG134100 Sapur.T017000 Sapur.T026500 Sapur.T044700 Sapur.T045900 Sapur.T049800 Sapur.T051500 Sapur.T064000 Sapur.006G141200 Sapur.005G174600
GO:0051261	6	17	protein depolymerization	Sapur.004G130100 Sapur.005G156700 Sapur.010G164300 Sapur.013G017100 Sapur.016G129900 Sapur.016G130000
GO:0046467	6	17	membrane lipid biosynthetic process	Sapur.001G026000 Sapur.002G189800 Sapur.003G060700 Sapur.003G062100 Sapur.019G022400 Sapur.T049800
GO:0030042	6	17	actin filament depolymerization	Sapur.004G130100 Sapur.005G156700 Sapur.010G164300 Sapur.013G017100 Sapur.016G129900 Sapur.016G130000
GO:0009247	6	17	glycolipid biosynthetic process	Sapur.001G026000 Sapur.002G189800 Sapur.003G060700 Sapur.003G062100 Sapur.019G022400 Sapur.T049800
GO:0006613	8	26	cotranslational protein targeting to membrane	Sapur.005G174600 Sapur.006G150200 Sapur.012G030400 Sapur.018G081000 Sapur.15ZG029200 Sapur.15ZG029900 Sapur.15ZG031600 Sapur.15ZG032200

GO:0006614	8	26	SRP-dependent cotranslational protein targeting to membrane	Sapur.005G174600 Sapur.006G150200 Sapur.012G030400 Sapur.018G081000 Sapur.15ZG029200 Sapur.15ZG029900 Sapur.15ZG031600 Sapur.15ZG032200
GO:0000045	4	9	autophagosome assembly	Sapur.002G176700 Sapur.003G076300 Sapur.004G007800 Sapur.011G003200
GO:1905037	4	9	autophagosome organization	Sapur.002G176700 Sapur.003G076300 Sapur.004G007800 Sapur.011G003200
GO:0016236	4	9	macroautophagy	Sapur.002G176700 Sapur.003G076300 Sapur.004G007800 Sapur.011G003200
GO:1901607	11	41	alpha-amino acid biosynthetic process	Sapur.004G144000 Sapur.005G073900 Sapur.009G120400 Sapur.012G016400 Sapur.013G056900 Sapur.016G032800 Sapur.019G038000 Sapur.019G038200 Sapur.15WG027600 Sapur.15ZG027200 Sapur.T140300
GO:0016310	262	1593	phosphorylation	Sapur.001G015800 Sapur.001G033500 Sapur.001G042400 Sapur.001G043100 Sapur.001G066800 Sapur.001G078000 Sapur.001G086800 Sapur.001G093700 Sapur.001G103900 Sapur.001G137500 Sapur.001G148700 Sapur.001G161400 Sapur.001G171200 Sapur.001G175900 Sapur.001G190300 Sapur.001G191000 Sapur.002G010500 Sapur.002G028000 Sapur.002G046900 Sapur.002G055700 Sapur.002G106900 Sapur.002G120400 Sapur.002G164000 Sapur.002G176000 Sapur.002G180800 Sapur.002G188100 Sapur.002G192000 Sapur.002G198100 Sapur.002G199800 Sapur.002G201200 Sapur.002G201300 Sapur.003G013500 Sapur.003G037300 Sapur.003G042700 Sapur.003G051100 Sapur.003G052500 Sapur.003G061800 Sapur.003G067600 Sapur.003G073200 Sapur.003G075500 Sapur.003G082200 Sapur.003G085300 Sapur.003G089700 Sapur.003G095700 Sapur.003G099700 Sapur.003G131900 Sapur.003G133000 Sapur.003G138700 Sapur.004G009000 Sapur.004G017100 Sapur.004G023900 Sapur.004G029200 Sapur.004G032900 Sapur.004G033000 Sapur.004G044400 Sapur.004G046500 Sapur.004G057300 Sapur.004G061000 Sapur.004G067800 Sapur.004G068600 Sapur.004G080700 Sapur.004G087900 Sapur.004G115800 Sapur.004G135100 Sapur.004G170800 Sapur.004G172200 Sapur.004G177200 Sapur.005G001800 Sapur.005G011700 Sapur.005G021200 Sapur.005G031100 Sapur.005G039800 Sapur.005G050800 Sapur.005G057200 Sapur.005G065000 Sapur.005G065300 Sapur.005G081600 Sapur.005G109200 Sapur.005G143900 Sapur.005G148400 Sapur.005G160000 Sapur.005G202000 Sapur.005G203500 Sapur.005G205600 Sapur.005G208100 Sapur.006G039400 Sapur.006G043700 Sapur.006G044000 Sapur.006G059800 Sapur.006G063800 Sapur.006G064600 Sapur.006G077000 Sapur.006G090100 Sapur.006G093700 Sapur.006G096300 Sapur.006G107000 Sapur.006G117000 Sapur.006G151400 Sapur.006G185100 Sapur.006G195000 Sapur.006G196200 Sapur.006G210500 Sapur.006G215000 Sapur.006G217500 Sapur.007G003100 Sapur.007G012300 Sapur.007G037800 Sapur.007G044000 Sapur.007G054000 Sapur.007G077400 Sapur.007G085900 Sapur.007G124000 Sapur.007G124100 Sapur.008G008800 Sapur.008G016100 Sapur.008G054200 Sapur.008G054500 Sapur.008G076100 Sapur.008G089200

				Sapur.008G107400 Sapur.008G111800 Sapur.008G113700 Sapur.008G116800 Sapur.008G136200 Sapur.008G136400 Sapur.008G143200 Sapur.008G165900 Sapur.009G030800 Sapur.009G058900 Sapur.009G059300 Sapur.009G091500 Sapur.009G092000 Sapur.009G107300 Sapur.010G000800 Sapur.010G028600 Sapur.010G041500 Sapur.010G052000 Sapur.010G064500 Sapur.010G070900 Sapur.010G075700 Sapur.010G076000 Sapur.010G076900 Sapur.010G127100 Sapur.010G145100 Sapur.010G147600 Sapur.010G147800 Sapur.010G169400 Sapur.010G189000 Sapur.011G021000 Sapur.011G030700 Sapur.011G036900 Sapur.011G040500 Sapur.011G040600 Sapur.011G046900 Sapur.011G047800 Sapur.011G050900 Sapur.011G080000 Sapur.011G080600 Sapur.011G089900 Sapur.011G095700 Sapur.011G097200 Sapur.011G101300 Sapur.011G108300 Sapur.011G121500 Sapur.012G020400 Sapur.012G029700 Sapur.012G032600 Sapur.012G041400 Sapur.012G048500 Sapur.012G051500 Sapur.012G052000 Sapur.012G063200 Sapur.012G064400 Sapur.012G064900 Sapur.012G093400 Sapur.012G097700 Sapur.012G106800 Sapur.013G003400 Sapur.013G034500 Sapur.013G055800 Sapur.013G108700 Sapur.013G141900 Sapur.014G014200 Sapur.014G017800 Sapur.014G050200 Sapur.014G054500 Sapur.014G065600 Sapur.014G067100 Sapur.014G067400 Sapur.014G069000 Sapur.014G084200 Sapur.014G091100 Sapur.014G095200 Sapur.014G110500 Sapur.014G119600 Sapur.014G126200 Sapur.016G045300 Sapur.016G046200 Sapur.016G048600 Sapur.016G049500 Sapur.016G064900 Sapur.016G090700 Sapur.016G113500 Sapur.016G115900 Sapur.016G122900 Sapur.016G133200 Sapur.016G164800 Sapur.016G165400 Sapur.016G222400 Sapur.016G256600 Sapur.016G263500 Sapur.016G267500 Sapur.016G267900 Sapur.016G269800 Sapur.016G272100 Sapur.016G274100 Sapur.016G293800 Sapur.016G294500 Sapur.016G304300 Sapur.017G001800 Sapur.017G002000 Sapur.017G019000 Sapur.017G072700 Sapur.017G096900 Sapur.017G097600 Sapur.017G107900 Sapur.017G111400 Sapur.017G114100 Sapur.017G125500 Sapur.018G018700 Sapur.018G035500 Sapur.018G048200 Sapur.018G053200 Sapur.018G058300 Sapur.018G060300 Sapur.018G067300 Sapur.018G072700 Sapur.018G082400 Sapur.018G086200 Sapur.018G107900 Sapur.018G114200 Sapur.019G008900 Sapur.019G061700 Sapur.019G062400 Sapur.019G083400 Sapur.019G099500 Sapur.019G113000 Sapur.15WG028200 Sapur.15WG031300 Sapur.15WG066000 Sapur.15WG079300 Sapur.15ZG018000 Sapur.15ZG027900 Sapur.15ZG035300 Sapur.15ZG054200 Sapur.15ZG082200 Sapur.15ZG085600 Sapur.15ZG093000 Sapur.15ZG124400 Sapur.15ZG134100 Sapur.T044700 Sapur.T051500
GO:0015698	10	37	inorganic anion transport	Sapur.001G185600 Sapur.002G038200 Sapur.003G029600 Sapur.008G105500 Sapur.008G126700 Sapur.016G310000 Sapur.018G097400 Sapur.003G108000 Sapur.007G049600 Sapur.15ZG006400
GO:0015980	6	18	energy derivation by oxidation of organic compounds	Sapur.002G054000 Sapur.004G050300 Sapur.004G142200 Sapur.005G150200 Sapur.005G150300 Sapur.009G118300
GO:0045333	6	18	cellular respiration	Sapur.002G054000 Sapur.004G050300 Sapur.004G142200 Sapur.005G150200 Sapur.005G150300 Sapur.009G118300
GO:0010256	3	6	endomembrane system	Sapur.005G080800 Sapur.012G098100 Sapur.15ZG093800

				organization	
	GO:0008215	3	6	spermine metabolic process	Sapur.008G171500 Sapur.010G013500 Sapur.010G104400
	GO:0043244	3	6	regulation of protein-containing complex disassembly	Sapur.005G156700 Sapur.010G125800 Sapur.013G017100
	GO:0006597	3	6	spermine biosynthetic process	Sapur.008G171500 Sapur.010G013500 Sapur.010G104400
	GO:0006544	3	6	glycine metabolic process	Sapur.004G005600 Sapur.006G189300 Sapur.011G005900
	GO:0006672	3	6	ceramide metabolic process	Sapur.001G175000 Sapur.016G193400 Sapur.017G046000
					Sapur.004G111400 Sapur.009G086200 Sapur.013G002400 Sapur.013G003000 Sapur.013G003100
m_up_BP	GO:0050794	395	1754	regulation of cellular process	Sapur.001G017300 Sapur.001G027900 Sapur.001G028100 Sapur.001G028400 Sapur.001G034200 Sapur.001G064300 Sapur.001G067700 Sapur.001G069800 Sapur.001G072500 Sapur.001G075700 Sapur.001G108900 Sapur.001G119600 Sapur.001G129000 Sapur.001G130000 Sapur.001G163400 Sapur.001G164600 Sapur.001G179800 Sapur.001G191500 Sapur.002G001600 Sapur.002G012900 Sapur.002G023100 Sapur.002G024200 Sapur.002G028500 Sapur.002G030000 Sapur.002G030100 Sapur.002G033200 Sapur.002G043300 Sapur.002G064600 Sapur.002G069500 Sapur.002G070300 Sapur.002G073000 Sapur.002G080700 Sapur.002G091700 Sapur.002G110600 Sapur.002G115400 Sapur.002G125900 Sapur.002G126000 Sapur.002G136900 Sapur.002G142300 Sapur.002G143500 Sapur.002G146000 Sapur.002G151100 Sapur.002G163600 Sapur.002G175800 Sapur.002G190900 Sapur.003G017600 Sapur.003G026500 Sapur.003G047900 Sapur.003G049200 Sapur.003G057500 Sapur.003G067100 Sapur.003G099900 Sapur.003G102000 Sapur.003G111600 Sapur.003G123800 Sapur.003G125900 Sapur.003G138200 Sapur.003G142400 Sapur.003G145700 Sapur.003G150800 Sapur.004G007000 Sapur.004G027800 Sapur.004G030500 Sapur.004G030800 Sapur.004G031600 Sapur.004G054800 Sapur.004G056900 Sapur.004G071100 Sapur.004G100100 Sapur.004G118800 Sapur.004G121500 Sapur.004G123100 Sapur.004G131600 Sapur.004G137000 Sapur.004G171400 Sapur.005G002500 Sapur.005G002800 Sapur.005G022300 Sapur.005G023100 Sapur.005G024800 Sapur.005G025000 Sapur.005G025400 Sapur.005G031700 Sapur.005G042000 Sapur.005G052900 Sapur.005G054000 Sapur.005G054800 Sapur.005G067000 Sapur.005G087400 Sapur.005G098100 Sapur.005G105500 Sapur.005G108500 Sapur.005G133200 Sapur.005G135200 Sapur.005G141300 Sapur.005G150000 Sapur.005G163900 Sapur.006G041100 Sapur.006G063200 Sapur.006G065500 Sapur.006G067500 Sapur.006G068600 Sapur.006G089400 Sapur.006G103400 Sapur.006G104700 Sapur.006G110100 Sapur.006G114200 Sapur.006G120000

Sapur.006G120300 Sapur.006G122700 Sapur.006G137200 Sapur.006G139900 Sapur.006G166000 Sapur.006G168000
Sapur.006G194100 Sapur.006G195200 Sapur.006G197300 Sapur.006G214400 Sapur.006G218000 Sapur.006G228700
Sapur.006G228900 Sapur.007G001300 Sapur.007G005500 Sapur.007G021900 Sapur.007G034000 Sapur.007G035800
Sapur.007G044600 Sapur.007G050900 Sapur.007G051700 Sapur.007G058700 Sapur.007G062200 Sapur.007G082500
Sapur.007G087800 Sapur.007G089800 Sapur.007G094000 Sapur.007G114800 Sapur.007G123400 Sapur.007G123700
Sapur.008G034200 Sapur.008G042700 Sapur.008G053100 Sapur.008G057000 Sapur.008G064800 Sapur.008G069200
Sapur.008G076200 Sapur.008G081900 Sapur.008G093800 Sapur.008G094300 Sapur.008G096700 Sapur.008G104300
Sapur.008G111900 Sapur.008G119700 Sapur.008G121600 Sapur.008G134800 Sapur.008G145300 Sapur.008G145600
Sapur.008G160400 Sapur.009G002400 Sapur.009G005300 Sapur.009G009700 Sapur.009G011600 Sapur.009G021700
Sapur.009G035200 Sapur.009G040000 Sapur.009G094800 Sapur.009G097900 Sapur.009G104900 Sapur.009G112800
Sapur.009G115000 Sapur.009G126000 Sapur.009G126600 Sapur.010G003100 Sapur.010G005200 Sapur.010G013000
Sapur.010G040000 Sapur.010G040100 Sapur.010G046500 Sapur.010G056400 Sapur.010G065900 Sapur.010G068700
Sapur.010G096300 Sapur.010G099700 Sapur.010G100200 Sapur.010G113700 Sapur.010G114700 Sapur.010G121400
Sapur.010G123400 Sapur.010G128300 Sapur.010G144500 Sapur.010G145400 Sapur.010G149100 Sapur.010G154300
Sapur.010G161100 Sapur.011G006600 Sapur.011G032100 Sapur.011G035500 Sapur.011G037900 Sapur.011G038200
Sapur.011G039300 Sapur.011G041100 Sapur.011G042400 Sapur.011G045800 Sapur.011G049300 Sapur.011G054600
Sapur.011G061100 Sapur.011G066500 Sapur.011G079300 Sapur.011G088400 Sapur.011G088500 Sapur.012G004000
Sapur.012G010400 Sapur.012G017700 Sapur.012G018600 Sapur.012G020900 Sapur.012G024500 Sapur.012G031400
Sapur.012G051400 Sapur.012G060100 Sapur.012G067900 Sapur.012G078700 Sapur.012G099900 Sapur.013G000400
Sapur.013G008000 Sapur.013G038800 Sapur.013G039500 Sapur.013G041800 Sapur.013G050700 Sapur.013G050900
Sapur.013G055500 Sapur.013G059000 Sapur.013G067900 Sapur.013G068800 Sapur.013G087600 Sapur.013G137400
Sapur.013G141800 Sapur.014G003700 Sapur.014G004000 Sapur.014G005200 Sapur.014G021100 Sapur.014G034500
Sapur.014G053000 Sapur.014G059000 Sapur.014G059100 Sapur.014G059200 Sapur.014G065300 Sapur.014G078300
Sapur.014G078500 Sapur.014G079300 Sapur.014G082100 Sapur.014G085400 Sapur.014G085500 Sapur.014G094500
Sapur.014G095700 Sapur.014G099500 Sapur.014G101000 Sapur.016G018700 Sapur.016G035600 Sapur.016G051900
Sapur.016G052700 Sapur.016G064600 Sapur.016G070400 Sapur.016G079900 Sapur.016G080200 Sapur.016G085100
Sapur.016G087700 Sapur.016G111900 Sapur.016G114700 Sapur.016G131300 Sapur.016G142200 Sapur.016G142300
Sapur.016G153200 Sapur.016G159000 Sapur.016G167800 Sapur.016G199700 Sapur.016G216900 Sapur.016G224700
Sapur.016G229800 Sapur.016G233500 Sapur.016G255700 Sapur.016G288500 Sapur.016G298800 Sapur.017G004800
Sapur.017G006400 Sapur.017G006500 Sapur.017G007100 Sapur.017G053300 Sapur.017G057100 Sapur.017G069900
Sapur.017G078300 Sapur.017G114800 Sapur.017G115700 Sapur.018G007100 Sapur.018G015000 Sapur.018G022700
Sapur.018G025000 Sapur.018G034800 Sapur.018G035300 Sapur.018G057700 Sapur.018G060400 Sapur.018G067800
Sapur.018G071800 Sapur.019G007100 Sapur.019G007800 Sapur.019G007900 Sapur.019G009300 Sapur.019G009600

				Sapur.019G010100 Sapur.019G010500 Sapur.019G010700 Sapur.019G011000 Sapur.019G011200 Sapur.019G011500 Sapur.019G011600 Sapur.019G011700 Sapur.019G011900 Sapur.019G012100 Sapur.019G012500 Sapur.019G012700 Sapur.019G012800 Sapur.019G016400 Sapur.019G017000 Sapur.019G025000 Sapur.019G033200 Sapur.019G034300 Sapur.019G039400 Sapur.019G039900 Sapur.019G042100 Sapur.019G042400 Sapur.019G042500 Sapur.019G043100 Sapur.019G043200 Sapur.019G044100 Sapur.019G053500 Sapur.019G060100 Sapur.019G074500 Sapur.019G091100 Sapur.019G091300 Sapur.019G091700 Sapur.019G092000 Sapur.019G092300 Sapur.019G092600 Sapur.019G092700 Sapur.019G092800 Sapur.019G093000 Sapur.019G093400 Sapur.019G093600 Sapur.019G093900 Sapur.019G094300 Sapur.019G098000 Sapur.019G108100 Sapur.019G110900 Sapur.15WG043400 Sapur.15WG060500 Sapur.15WG062900 Sapur.15WG065100 Sapur.15ZG007300 Sapur.15ZG009400 Sapur.15ZG016900 Sapur.15ZG050400 Sapur.15ZG050500 Sapur.15ZG053200 Sapur.15ZG067200 Sapur.15ZG071900 Sapur.15ZG079000 Sapur.15ZG080900 Sapur.15ZG103400 Sapur.T006300 Sapur.T012000 Sapur.T116500 Sapur.T150000 Sapur.T170000 Sapur.T175100 Sapur.006G141000 Sapur.001G041100 Sapur.001G121200 Sapur.001G162000 Sapur.006G030100 Sapur.006G216200 Sapur.008G146500 Sapur.008G155300 Sapur.008G158900 Sapur.010G018800 Sapur.012G002400 Sapur.013G028600 Sapur.014G083600 Sapur.016G034100 Sapur.018G017300 Sapur.018G017400 Sapur.018G036400 Sapur.15WG079800 Sapur.004G111400 Sapur.009G086200 Sapur.013G002400 Sapur.013G003000 Sapur.013G003100
GO:0050789	396	1760	regulation of biological process	Sapur.001G017300 Sapur.001G027900 Sapur.001G028100 Sapur.001G028400 Sapur.001G034200 Sapur.001G064300 Sapur.001G067700 Sapur.001G069800 Sapur.001G072500 Sapur.001G075700 Sapur.001G108900 Sapur.001G119600 Sapur.001G129000 Sapur.001G130000 Sapur.001G163400 Sapur.001G164600 Sapur.001G179800 Sapur.001G191500 Sapur.002G001600 Sapur.002G012900 Sapur.002G023100 Sapur.002G024200 Sapur.002G028500 Sapur.002G030000 Sapur.002G030100 Sapur.002G033200 Sapur.002G043300 Sapur.002G064600 Sapur.002G069500 Sapur.002G070300 Sapur.002G073000 Sapur.002G080700 Sapur.002G091700 Sapur.002G110600 Sapur.002G115400 Sapur.002G125900 Sapur.002G126000 Sapur.002G136900 Sapur.002G142300 Sapur.002G143500 Sapur.002G146000 Sapur.002G151100 Sapur.002G163600 Sapur.002G175800 Sapur.002G190900 Sapur.003G017600 Sapur.003G026500 Sapur.003G047900 Sapur.003G049200 Sapur.003G057500 Sapur.003G067100 Sapur.003G099900 Sapur.003G102000 Sapur.003G111600 Sapur.003G123800 Sapur.003G125900 Sapur.003G138200 Sapur.003G142400 Sapur.003G145700 Sapur.003G150800 Sapur.004G007000 Sapur.004G027800 Sapur.004G030500 Sapur.004G030800 Sapur.004G031600 Sapur.004G054800 Sapur.004G056900 Sapur.004G071100 Sapur.004G100100 Sapur.004G118800 Sapur.004G121500 Sapur.004G123100 Sapur.004G131600 Sapur.004G137000 Sapur.004G171400 Sapur.005G002500 Sapur.005G002800 Sapur.005G022300 Sapur.005G023100 Sapur.005G024800 Sapur.005G025000 Sapur.005G025400 Sapur.005G031700 Sapur.005G042000 Sapur.005G052900 Sapur.005G054000 Sapur.005G054800 Sapur.005G067000 Sapur.005G087400 Sapur.005G098100 Sapur.005G105500 Sapur.005G108500 Sapur.005G133200 Sapur.005G135200 Sapur.005G141300 Sapur.005G150000 Sapur.005G163900 Sapur.006G041100 Sapur.006G063200 Sapur.006G065500 Sapur.006G067500 Sapur.006G068600 Sapur.006G089400 Sapur.006G103400 Sapur.006G104700 Sapur.006G110100 Sapur.006G114200 Sapur.006G120000

	Sapur.006G120300 Sapur.006G122700 Sapur.006G137200 Sapur.006G139900 Sapur.006G166000 Sapur.006G168000 Sapur.006G194100 Sapur.006G195200 Sapur.006G197300 Sapur.006G214400 Sapur.006G218000 Sapur.006G228700 Sapur.006G228900 Sapur.007G001300 Sapur.007G005500 Sapur.007G021900 Sapur.007G034000 Sapur.007G035800 Sapur.007G044600 Sapur.007G050900 Sapur.007G051700 Sapur.007G058700 Sapur.007G062200 Sapur.007G082500 Sapur.007G087800 Sapur.007G089800 Sapur.007G094000 Sapur.007G114800 Sapur.007G123400 Sapur.007G123700 Sapur.008G034200 Sapur.008G042700 Sapur.008G053100 Sapur.008G057000 Sapur.008G064800 Sapur.008G069200 Sapur.008G076200 Sapur.008G081900 Sapur.008G093800 Sapur.008G094300 Sapur.008G096700 Sapur.008G104300 Sapur.008G110000 Sapur.008G111900 Sapur.008G119700 Sapur.008G121600 Sapur.008G134800 Sapur.008G145300 Sapur.008G145600 Sapur.008G160400 Sapur.009G002400 Sapur.009G005300 Sapur.009G009700 Sapur.009G011600 Sapur.009G021700 Sapur.009G035200 Sapur.009G040000 Sapur.009G094800 Sapur.009G097900 Sapur.009G104900 Sapur.009G112800 Sapur.009G115000 Sapur.009G126000 Sapur.009G126600 Sapur.010G003100 Sapur.010G005200 Sapur.010G013000 Sapur.010G040000 Sapur.010G040100 Sapur.010G046500 Sapur.010G056400 Sapur.010G065900 Sapur.010G068700 Sapur.010G096300 Sapur.010G099700 Sapur.010G100200 Sapur.010G113700 Sapur.010G114700 Sapur.010G121400 Sapur.010G123400 Sapur.010G128300 Sapur.010G144500 Sapur.010G145400 Sapur.010G149100 Sapur.010G154300 Sapur.010G161100 Sapur.011G006600 Sapur.011G032100 Sapur.011G035500 Sapur.011G037900 Sapur.011G038200 Sapur.011G039300 Sapur.011G041100 Sapur.011G042400 Sapur.011G045800 Sapur.011G049300 Sapur.011G054600 Sapur.011G061100 Sapur.011G066500 Sapur.011G079300 Sapur.011G088400 Sapur.011G088500 Sapur.012G004000 Sapur.012G010400 Sapur.012G017700 Sapur.012G018600 Sapur.012G020900 Sapur.012G024500 Sapur.012G031400 Sapur.012G051400 Sapur.012G060100 Sapur.012G067900 Sapur.012G078700 Sapur.012G099900 Sapur.013G000400 Sapur.013G008000 Sapur.013G038800 Sapur.013G039500 Sapur.013G041800 Sapur.013G050700 Sapur.013G050900 Sapur.013G055500 Sapur.013G059000 Sapur.013G067900 Sapur.013G068800 Sapur.013G087600 Sapur.013G137400 Sapur.013G141800 Sapur.014G003700 Sapur.014G004000 Sapur.014G005200 Sapur.014G021100 Sapur.014G034500 Sapur.014G053000 Sapur.014G059000 Sapur.014G059100 Sapur.014G059200 Sapur.014G065300 Sapur.014G078300 Sapur.014G078500 Sapur.014G079300 Sapur.014G082100 Sapur.014G085400 Sapur.014G085500 Sapur.014G094500 Sapur.014G095700 Sapur.014G099500 Sapur.014G101000 Sapur.016G018700 Sapur.016G035600 Sapur.016G051900 Sapur.016G052700 Sapur.016G064600 Sapur.016G070400 Sapur.016G079900 Sapur.016G080200 Sapur.016G085100 Sapur.016G087700 Sapur.016G111900 Sapur.016G114700 Sapur.016G131300 Sapur.016G142200 Sapur.016G142300 Sapur.016G153200 Sapur.016G159000 Sapur.016G167800 Sapur.016G199700 Sapur.016G216900 Sapur.016G224700 Sapur.016G229800 Sapur.016G233500 Sapur.016G255700 Sapur.016G288500 Sapur.016G298800 Sapur.017G004800 Sapur.017G006400 Sapur.017G006500 Sapur.017G007100 Sapur.017G053300 Sapur.017G057100 Sapur.017G069900 Sapur.017G078300 Sapur.017G114800 Sapur.017G115700 Sapur.018G007100 Sapur.018G015000 Sapur.018G022700 Sapur.018G025000 Sapur.018G034800 Sapur.018G035300 Sapur.018G057700 Sapur.018G060400 Sapur.018G067800 Sapur.018G071800 Sapur.019G007100 Sapur.019G007800 Sapur.019G007900 Sapur.019G009300

				Sapur.019G009600 Sapur.019G010100 Sapur.019G010500 Sapur.019G010700 Sapur.019G011000 Sapur.019G011200 Sapur.019G011500 Sapur.019G011600 Sapur.019G011700 Sapur.019G011900 Sapur.019G012100 Sapur.019G012500 Sapur.019G012700 Sapur.019G012800 Sapur.019G016400 Sapur.019G017000 Sapur.019G025000 Sapur.019G033200 Sapur.019G034300 Sapur.019G039400 Sapur.019G039900 Sapur.019G042100 Sapur.019G042400 Sapur.019G042500 Sapur.019G043100 Sapur.019G043200 Sapur.019G044100 Sapur.019G053500 Sapur.019G060100 Sapur.019G074500 Sapur.019G091100 Sapur.019G091300 Sapur.019G091700 Sapur.019G092000 Sapur.019G092300 Sapur.019G092600 Sapur.019G092700 Sapur.019G092800 Sapur.019G093000 Sapur.019G093400 Sapur.019G093600 Sapur.019G093900 Sapur.019G094300 Sapur.019G098000 Sapur.019G108100 Sapur.019G110900 Sapur.15WG043400 Sapur.15WG060500 Sapur.15WG062900 Sapur.15WG065100 Sapur.15ZG007300 Sapur.15ZG009400 Sapur.15ZG016900 Sapur.15ZG050400 Sapur.15ZG050500 Sapur.15ZG053200 Sapur.15ZG067200 Sapur.15ZG071900 Sapur.15ZG079000 Sapur.15ZG080900 Sapur.15ZG103400 Sapur.T006300 Sapur.T012000 Sapur.T116500 Sapur.T150000 Sapur.T170000 Sapur.T175100 Sapur.006G141000 Sapur.001G041100 Sapur.001G121200 Sapur.001G162000 Sapur.006G030100 Sapur.006G216200 Sapur.008G146500 Sapur.008G155300 Sapur.008G158900 Sapur.010G018800 Sapur.012G002400 Sapur.013G028600 Sapur.014G083600 Sapur.016G034100 Sapur.018G017300 Sapur.018G017400 Sapur.018G036400 Sapur.15WG079800 Sapur.004G111400 Sapur.009G086200 Sapur.013G002400 Sapur.013G003000 Sapur.013G003100
GO:0065007	396	1767	biological regulation	Sapur.001G017300 Sapur.001G027900 Sapur.001G028100 Sapur.001G028400 Sapur.001G034200 Sapur.001G064300 Sapur.001G067700 Sapur.001G069800 Sapur.001G072500 Sapur.001G075700 Sapur.001G108900 Sapur.001G119600 Sapur.001G129000 Sapur.001G130000 Sapur.001G163400 Sapur.001G164600 Sapur.001G179800 Sapur.001G191500 Sapur.002G001600 Sapur.002G012900 Sapur.002G023100 Sapur.002G024200 Sapur.002G028500 Sapur.002G030000 Sapur.002G030100 Sapur.002G033200 Sapur.002G043300 Sapur.002G064600 Sapur.002G069500 Sapur.002G070300 Sapur.002G073000 Sapur.002G080700 Sapur.002G091700 Sapur.002G110600 Sapur.002G115400 Sapur.002G125900 Sapur.002G126000 Sapur.002G136900 Sapur.002G142300 Sapur.002G143500 Sapur.002G146000 Sapur.002G151100 Sapur.002G163600 Sapur.002G175800 Sapur.002G190900 Sapur.003G017600 Sapur.003G026500 Sapur.003G047900 Sapur.003G049200 Sapur.003G057500 Sapur.003G067100 Sapur.003G099900 Sapur.003G102000 Sapur.003G111600 Sapur.003G123800 Sapur.003G125900 Sapur.003G138200 Sapur.003G142400 Sapur.003G145700 Sapur.003G150800 Sapur.004G007000 Sapur.004G027800 Sapur.004G030500 Sapur.004G030800 Sapur.004G031600 Sapur.004G054800 Sapur.004G056900 Sapur.004G071100 Sapur.004G100100 Sapur.004G118800 Sapur.004G121500 Sapur.004G123100 Sapur.004G131600 Sapur.004G137000 Sapur.004G171400 Sapur.005G002500 Sapur.005G002800 Sapur.005G022300 Sapur.005G023100 Sapur.005G024800 Sapur.005G025000 Sapur.005G025400 Sapur.005G031700 Sapur.005G042000 Sapur.005G052900 Sapur.005G054000 Sapur.005G054800 Sapur.005G067000 Sapur.005G087400 Sapur.005G098100 Sapur.005G105500 Sapur.005G108500 Sapur.005G133200 Sapur.005G135200 Sapur.005G141300 Sapur.005G150000 Sapur.005G163900 Sapur.006G041100 Sapur.006G063200 Sapur.006G065500 Sapur.006G067500 Sapur.006G068600 Sapur.006G089400 Sapur.006G103400 Sapur.006G104700 Sapur.006G110100 Sapur.006G114200 Sapur.006G120000

Sapur.006G120300 Sapur.006G122700 Sapur.006G137200 Sapur.006G139900 Sapur.006G166000 Sapur.006G168000
Sapur.006G194100 Sapur.006G195200 Sapur.006G197300 Sapur.006G214400 Sapur.006G218000 Sapur.006G228700
Sapur.006G228900 Sapur.007G001300 Sapur.007G005500 Sapur.007G021900 Sapur.007G034000 Sapur.007G035800
Sapur.007G044600 Sapur.007G050900 Sapur.007G051700 Sapur.007G058700 Sapur.007G062200 Sapur.007G082500
Sapur.007G087800 Sapur.007G089800 Sapur.007G094000 Sapur.007G114800 Sapur.007G123400 Sapur.007G123700
Sapur.008G034200 Sapur.008G042700 Sapur.008G053100 Sapur.008G057000 Sapur.008G064800 Sapur.008G069200
Sapur.008G076200 Sapur.008G081900 Sapur.008G093800 Sapur.008G094300 Sapur.008G096700 Sapur.008G104300
Sapur.008G110000 Sapur.008G111900 Sapur.008G119700 Sapur.008G121600 Sapur.008G134800 Sapur.008G145300
Sapur.008G145600 Sapur.008G160400 Sapur.009G002400 Sapur.009G005300 Sapur.009G009700 Sapur.009G011600
Sapur.009G021700 Sapur.009G035200 Sapur.009G040000 Sapur.009G094800 Sapur.009G097900 Sapur.009G104900
Sapur.009G112800 Sapur.009G115000 Sapur.009G126000 Sapur.009G126600 Sapur.010G003100 Sapur.010G005200
Sapur.010G013000 Sapur.010G040000 Sapur.010G040100 Sapur.010G046500 Sapur.010G056400 Sapur.010G065900
Sapur.010G068700 Sapur.010G096300 Sapur.010G099700 Sapur.010G100200 Sapur.010G113700 Sapur.010G114700
Sapur.010G121400 Sapur.010G123400 Sapur.010G128300 Sapur.010G144500 Sapur.010G145400 Sapur.010G149100
Sapur.010G154300 Sapur.010G161100 Sapur.011G006600 Sapur.011G032100 Sapur.011G035500 Sapur.011G037900
Sapur.011G038200 Sapur.011G039300 Sapur.011G041100 Sapur.011G042400 Sapur.011G045800 Sapur.011G049300
Sapur.011G054600 Sapur.011G061100 Sapur.011G066500 Sapur.011G079300 Sapur.011G088400 Sapur.011G088500
Sapur.012G004000 Sapur.012G010400 Sapur.012G017700 Sapur.012G018600 Sapur.012G020900 Sapur.012G024500
Sapur.012G031400 Sapur.012G051400 Sapur.012G060100 Sapur.012G067900 Sapur.012G078700 Sapur.012G099900
Sapur.013G000400 Sapur.013G008000 Sapur.013G038800 Sapur.013G039500 Sapur.013G041800 Sapur.013G050700
Sapur.013G050900 Sapur.013G055500 Sapur.013G059000 Sapur.013G067900 Sapur.013G068800 Sapur.013G087600
Sapur.013G137400 Sapur.013G141800 Sapur.014G003700 Sapur.014G004000 Sapur.014G005200 Sapur.014G021100
Sapur.014G034500 Sapur.014G053000 Sapur.014G059000 Sapur.014G059100 Sapur.014G059200 Sapur.014G065300
Sapur.014G078300 Sapur.014G078500 Sapur.014G079300 Sapur.014G082100 Sapur.014G085400 Sapur.014G085500
Sapur.014G094500 Sapur.014G095700 Sapur.014G099500 Sapur.014G101000 Sapur.016G018700 Sapur.016G035600
Sapur.016G051900 Sapur.016G052700 Sapur.016G064600 Sapur.016G070400 Sapur.016G079900 Sapur.016G080200
Sapur.016G085100 Sapur.016G087700 Sapur.016G111900 Sapur.016G114700 Sapur.016G131300 Sapur.016G142200
Sapur.016G142300 Sapur.016G153200 Sapur.016G159000 Sapur.016G167800 Sapur.016G199700 Sapur.016G216900
Sapur.016G224700 Sapur.016G229800 Sapur.016G233500 Sapur.016G255700 Sapur.016G288500 Sapur.016G298800
Sapur.017G004800 Sapur.017G006400 Sapur.017G006500 Sapur.017G007100 Sapur.017G053300 Sapur.017G057100
Sapur.017G069900 Sapur.017G078300 Sapur.017G114800 Sapur.017G115700 Sapur.018G007100 Sapur.018G015000
Sapur.018G022700 Sapur.018G025000 Sapur.018G034800 Sapur.018G035300 Sapur.018G057700 Sapur.018G060400
Sapur.018G067800 Sapur.018G071800 Sapur.019G007100 Sapur.019G007800 Sapur.019G007900 Sapur.019G009300

				Sapur.019G009600 Sapur.019G010100 Sapur.019G010500 Sapur.019G010700 Sapur.019G011000 Sapur.019G011200 Sapur.019G011500 Sapur.019G011600 Sapur.019G011700 Sapur.019G011900 Sapur.019G012100 Sapur.019G012500 Sapur.019G012700 Sapur.019G012800 Sapur.019G016400 Sapur.019G017000 Sapur.019G025000 Sapur.019G033200 Sapur.019G034300 Sapur.019G039400 Sapur.019G039900 Sapur.019G042100 Sapur.019G042400 Sapur.019G042500 Sapur.019G043100 Sapur.019G043200 Sapur.019G044100 Sapur.019G053500 Sapur.019G060100 Sapur.019G074500 Sapur.019G091100 Sapur.019G091300 Sapur.019G091700 Sapur.019G092000 Sapur.019G092300 Sapur.019G092600 Sapur.019G092700 Sapur.019G092800 Sapur.019G093000 Sapur.019G093400 Sapur.019G093600 Sapur.019G093900 Sapur.019G094300 Sapur.019G098000 Sapur.019G108100 Sapur.019G110900 Sapur.15WG043400 Sapur.15WG060500 Sapur.15WG062900 Sapur.15WG065100 Sapur.15ZG007300 Sapur.15ZG009400 Sapur.15ZG016900 Sapur.15ZG050400 Sapur.15ZG050500 Sapur.15ZG053200 Sapur.15ZG067200 Sapur.15ZG071900 Sapur.15ZG079000 Sapur.15ZG080900 Sapur.15ZG103400 Sapur.T006300 Sapur.T012000 Sapur.T116500 Sapur.T150000 Sapur.T170000 Sapur.T175100 Sapur.006G141000 Sapur.001G041100 Sapur.001G121200 Sapur.001G162000 Sapur.006G030100 Sapur.006G216200 Sapur.008G146500 Sapur.008G155300 Sapur.008G158900 Sapur.010G018800 Sapur.012G002400 Sapur.013G028600 Sapur.014G083600 Sapur.016G034100 Sapur.018G017300 Sapur.018G017400 Sapur.018G036400 Sapur.15WG079800 Sapur.013G002400 Sapur.013G003000 Sapur.013G003100 Sapur.001G017300 Sapur.001G027900
GO:0031323	287	1210	regulation of cellular metabolic process	Sapur.001G028100 Sapur.001G028400 Sapur.001G034200 Sapur.001G064300 Sapur.001G067700 Sapur.001G069800 Sapur.001G072500 Sapur.001G075700 Sapur.001G108900 Sapur.001G119600 Sapur.001G129000 Sapur.001G130000 Sapur.001G163400 Sapur.001G164600 Sapur.001G179800 Sapur.002G012900 Sapur.002G023100 Sapur.002G024200 Sapur.002G028500 Sapur.002G030000 Sapur.002G030100 Sapur.002G033200 Sapur.002G043300 Sapur.002G064600 Sapur.002G069500 Sapur.002G073000 Sapur.002G080700 Sapur.002G091700 Sapur.002G110600 Sapur.002G115400 Sapur.002G125900 Sapur.002G126000 Sapur.002G136900 Sapur.002G142300 Sapur.002G143500 Sapur.002G146000 Sapur.002G151100 Sapur.002G163600 Sapur.002G175800 Sapur.002G190900 Sapur.003G017600 Sapur.003G026500 Sapur.003G047900 Sapur.003G049200 Sapur.003G057500 Sapur.003G067100 Sapur.003G099900 Sapur.003G102000 Sapur.003G111600 Sapur.003G123800 Sapur.003G125900 Sapur.003G138200 Sapur.003G142400 Sapur.003G145700 Sapur.003G150800 Sapur.004G007000 Sapur.004G027800 Sapur.004G030500 Sapur.004G030800 Sapur.004G031600 Sapur.004G054800 Sapur.004G056900 Sapur.004G071100 Sapur.004G100100 Sapur.004G118800 Sapur.004G121500 Sapur.004G123100 Sapur.004G131600 Sapur.004G137000 Sapur.004G171400 Sapur.005G042000 Sapur.005G052900 Sapur.005G054800 Sapur.005G067000 Sapur.005G087400 Sapur.005G098100 Sapur.005G105500 Sapur.005G108500 Sapur.005G133200 Sapur.005G141300 Sapur.005G150000 Sapur.005G163900 Sapur.006G041100 Sapur.006G063200 Sapur.006G067500 Sapur.006G068600 Sapur.006G089400 Sapur.006G103400 Sapur.006G104700 Sapur.006G110100 Sapur.006G114200 Sapur.006G120000 Sapur.006G120300 Sapur.006G122700 Sapur.006G137200 Sapur.006G166000 Sapur.006G168000 Sapur.006G194100 Sapur.006G195200 Sapur.006G197300 Sapur.006G214400 Sapur.006G218000 Sapur.006G228700 Sapur.006G228900 Sapur.007G001300 Sapur.007G005500 Sapur.007G021900 Sapur.007G034000

				Sapur.007G035800 Sapur.007G044600 Sapur.007G050900 Sapur.007G058700 Sapur.007G062200 Sapur.007G082500 Sapur.007G087800 Sapur.007G089800 Sapur.007G094000 Sapur.007G114800 Sapur.007G123400 Sapur.007G123700 Sapur.008G034200 Sapur.008G042700 Sapur.008G053100 Sapur.008G064800 Sapur.008G069200 Sapur.008G081900 Sapur.008G093800 Sapur.008G094300 Sapur.008G096700 Sapur.008G104300 Sapur.008G119700 Sapur.008G121600 Sapur.008G134800 Sapur.008G160400 Sapur.009G002400 Sapur.009G005300 Sapur.009G009700 Sapur.009G011600 Sapur.009G021700 Sapur.009G040000 Sapur.009G094800 Sapur.009G097900 Sapur.009G104900 Sapur.009G112800 Sapur.009G115000 Sapur.009G126000 Sapur.009G126600 Sapur.010G003100 Sapur.010G005200 Sapur.010G013000 Sapur.010G040000 Sapur.010G040100 Sapur.010G046500 Sapur.010G065900 Sapur.010G068700 Sapur.010G096300 Sapur.010G099700 Sapur.010G100200 Sapur.010G113700 Sapur.010G114700 Sapur.010G123400 Sapur.010G128300 Sapur.010G144500 Sapur.010G149100 Sapur.010G154300 Sapur.010G161100 Sapur.011G006600 Sapur.011G032100 Sapur.011G035500 Sapur.011G037900 Sapur.011G038200 Sapur.011G039300 Sapur.011G041100 Sapur.011G042400 Sapur.011G045800 Sapur.011G049300 Sapur.011G054600 Sapur.011G061100 Sapur.011G066500 Sapur.011G079300 Sapur.011G088400 Sapur.011G088500 Sapur.012G004000 Sapur.012G010400 Sapur.012G017700 Sapur.012G018600 Sapur.012G024500 Sapur.012G031400 Sapur.012G051400 Sapur.012G060100 Sapur.012G067900 Sapur.012G078700 Sapur.012G099900 Sapur.013G000400 Sapur.013G008000 Sapur.013G038800 Sapur.013G039500 Sapur.013G041800 Sapur.013G050700 Sapur.013G050900 Sapur.013G055500 Sapur.013G059000 Sapur.013G067900 Sapur.013G068800 Sapur.013G087600 Sapur.013G137400 Sapur.013G141800 Sapur.014G003700 Sapur.014G004000 Sapur.014G005200 Sapur.014G034500 Sapur.014G053000 Sapur.014G059000 Sapur.014G059100 Sapur.014G059200 Sapur.014G065300 Sapur.014G078300 Sapur.014G078500 Sapur.014G079300 Sapur.014G085400 Sapur.014G085500 Sapur.014G094500 Sapur.014G095700 Sapur.014G099500 Sapur.014G101000 Sapur.016G018700 Sapur.016G064600 Sapur.016G070400 Sapur.016G079900 Sapur.016G080200 Sapur.016G085100 Sapur.016G087700 Sapur.016G111900 Sapur.016G114700 Sapur.016G131300 Sapur.016G153200 Sapur.016G159000 Sapur.016G167800 Sapur.016G199700 Sapur.016G216900 Sapur.016G224700 Sapur.016G229800 Sapur.016G233500 Sapur.016G255700 Sapur.016G298800 Sapur.017G004800 Sapur.017G006400 Sapur.017G006500 Sapur.017G007100 Sapur.017G053300 Sapur.017G057100 Sapur.017G069900 Sapur.017G114800 Sapur.017G115700 Sapur.018G007100 Sapur.018G022700 Sapur.018G025000 Sapur.018G034800 Sapur.018G035300 Sapur.018G057700 Sapur.018G067800 Sapur.019G025000 Sapur.019G033200 Sapur.019G039400 Sapur.019G039900 Sapur.019G060100 Sapur.019G074500 Sapur.019G098000 Sapur.019G108100 Sapur.15WG060500 Sapur.15WG062900 Sapur.15WG065100 Sapur.15ZG007300 Sapur.15ZG016900 Sapur.15ZG053200 Sapur.15ZG067200 Sapur.15ZG071900 Sapur.15ZG079000 Sapur.15ZG080900 Sapur.15ZG103400 Sapur.T006300 Sapur.T012000
GO:0060255	287	1211	regulation of macromolecule metabolic process	Sapur.013G002400 Sapur.013G003000 Sapur.013G003100 Sapur.001G017300 Sapur.001G027900 Sapur.001G028100 Sapur.001G028400 Sapur.001G034200 Sapur.001G064300 Sapur.001G067700 Sapur.001G069800 Sapur.001G072500 Sapur.001G075700 Sapur.001G108900 Sapur.001G119600 Sapur.001G129000 Sapur.001G130000 Sapur.001G163400 Sapur.001G164600 Sapur.001G179800 Sapur.002G012900 Sapur.002G023100 Sapur.002G024200

Sapur.002G028500	Sapur.002G030000	Sapur.002G030100	Sapur.002G033200	Sapur.002G043300	Sapur.002G064600
Sapur.002G069500	Sapur.002G073000	Sapur.002G080700	Sapur.002G091700	Sapur.002G110600	Sapur.002G115400
Sapur.002G125900	Sapur.002G126000	Sapur.002G136900	Sapur.002G142300	Sapur.002G143500	Sapur.002G146000
Sapur.002G151100	Sapur.002G163600	Sapur.002G175800	Sapur.002G190900	Sapur.003G017600	Sapur.003G026500
Sapur.003G047900	Sapur.003G049200	Sapur.003G057500	Sapur.003G067100	Sapur.003G099900	Sapur.003G102000
Sapur.003G111600	Sapur.003G123800	Sapur.003G125900	Sapur.003G138200	Sapur.003G142400	Sapur.003G145700
Sapur.003G150800	Sapur.004G007000	Sapur.004G027800	Sapur.004G030500	Sapur.004G030800	Sapur.004G031600
Sapur.004G054800	Sapur.004G056900	Sapur.004G071100	Sapur.004G100100	Sapur.004G118800	Sapur.004G121500
Sapur.004G123100	Sapur.004G131600	Sapur.004G137000	Sapur.004G171400	Sapur.005G042000	Sapur.005G052900
Sapur.005G054800	Sapur.005G067000	Sapur.005G087400	Sapur.005G098100	Sapur.005G105500	Sapur.005G108500
Sapur.005G133200	Sapur.005G141300	Sapur.005G150000	Sapur.005G163900	Sapur.006G041100	Sapur.006G063200
Sapur.006G067500	Sapur.006G068600	Sapur.006G089400	Sapur.006G103400	Sapur.006G104700	Sapur.006G110100
Sapur.006G114200	Sapur.006G120000	Sapur.006G120300	Sapur.006G122700	Sapur.006G137200	Sapur.006G166000
Sapur.006G168000	Sapur.006G194100	Sapur.006G195200	Sapur.006G197300	Sapur.006G214400	Sapur.006G218000
Sapur.006G228700	Sapur.006G228900	Sapur.007G001300	Sapur.007G005500	Sapur.007G021900	Sapur.007G034000
Sapur.007G035800	Sapur.007G044600	Sapur.007G050900	Sapur.007G058700	Sapur.007G062200	Sapur.007G082500
Sapur.007G087800	Sapur.007G089800	Sapur.007G094000	Sapur.007G114800	Sapur.007G123400	Sapur.007G123700
Sapur.008G034200	Sapur.008G042700	Sapur.008G053100	Sapur.008G064800	Sapur.008G069200	Sapur.008G081900
Sapur.008G093800	Sapur.008G094300	Sapur.008G096700	Sapur.008G104300	Sapur.008G119700	Sapur.008G121600
Sapur.008G134800	Sapur.008G160400	Sapur.009G002400	Sapur.009G005300	Sapur.009G009700	Sapur.009G011600
Sapur.009G021700	Sapur.009G040000	Sapur.009G094800	Sapur.009G097900	Sapur.009G104900	Sapur.009G112800
Sapur.009G115000	Sapur.009G126000	Sapur.009G126600	Sapur.010G003100	Sapur.010G005200	Sapur.010G013000
Sapur.010G040000	Sapur.010G040100	Sapur.010G046500	Sapur.010G065900	Sapur.010G068700	Sapur.010G096300
Sapur.010G099700	Sapur.010G100200	Sapur.010G113700	Sapur.010G114700	Sapur.010G123400	Sapur.010G128300
Sapur.010G144500	Sapur.010G149100	Sapur.010G154300	Sapur.010G161100	Sapur.011G006600	Sapur.011G032100
Sapur.011G035500	Sapur.011G037900	Sapur.011G038200	Sapur.011G039300	Sapur.011G041100	Sapur.011G042400
Sapur.011G045800	Sapur.011G049300	Sapur.011G054600	Sapur.011G061100	Sapur.011G066500	Sapur.011G079300
Sapur.011G088400	Sapur.011G088500	Sapur.012G004000	Sapur.012G010400	Sapur.012G017700	Sapur.012G018600
Sapur.012G024500	Sapur.012G031400	Sapur.012G051400	Sapur.012G060100	Sapur.012G067900	Sapur.012G078700
Sapur.012G099900	Sapur.013G000400	Sapur.013G008000	Sapur.013G038800	Sapur.013G039500	Sapur.013G041800
Sapur.013G050700	Sapur.013G050900	Sapur.013G055500	Sapur.013G059000	Sapur.013G067900	Sapur.013G068800
Sapur.013G087600	Sapur.013G137400	Sapur.013G141800	Sapur.014G003700	Sapur.014G004000	Sapur.014G005200
Sapur.014G034500	Sapur.014G053000	Sapur.014G059000	Sapur.014G059100	Sapur.014G059200	Sapur.014G065300

				Sapur.014G078300 Sapur.014G078500 Sapur.014G079300 Sapur.014G085400 Sapur.014G085500 Sapur.014G094500 Sapur.014G095700 Sapur.014G099500 Sapur.014G101000 Sapur.016G018700 Sapur.016G064600 Sapur.016G070400 Sapur.016G079900 Sapur.016G080200 Sapur.016G085100 Sapur.016G087700 Sapur.016G111900 Sapur.016G114700 Sapur.016G131300 Sapur.016G153200 Sapur.016G159000 Sapur.016G167800 Sapur.016G199700 Sapur.016G216900 Sapur.016G224700 Sapur.016G229800 Sapur.016G233500 Sapur.016G255700 Sapur.016G298800 Sapur.017G004800 Sapur.017G006400 Sapur.017G006500 Sapur.017G007100 Sapur.017G053300 Sapur.017G057100 Sapur.017G069900 Sapur.017G114800 Sapur.017G115700 Sapur.018G007100 Sapur.018G022700 Sapur.018G025000 Sapur.018G034800 Sapur.018G035300 Sapur.018G057700 Sapur.018G067800 Sapur.019G025000 Sapur.019G033200 Sapur.019G039400 Sapur.019G039900 Sapur.019G060100 Sapur.019G074500 Sapur.019G098000 Sapur.019G108100 Sapur.15WG060500 Sapur.15WG062900 Sapur.15WG065100 Sapur.15ZG007300 Sapur.15ZG016900 Sapur.15ZG053200 Sapur.15ZG067200 Sapur.15ZG071900 Sapur.15ZG079000 Sapur.15ZG080900 Sapur.15ZG103400 Sapur.T006300 Sapur.T012000
GO:0019222	287	1214	regulation of metabolic process	Sapur.013G002400 Sapur.013G003000 Sapur.013G003100 Sapur.001G017300 Sapur.001G027900 Sapur.001G028100 Sapur.001G028400 Sapur.001G034200 Sapur.001G064300 Sapur.001G067700 Sapur.001G069800 Sapur.001G072500 Sapur.001G075700 Sapur.001G108900 Sapur.001G119600 Sapur.001G129000 Sapur.001G130000 Sapur.001G163400 Sapur.001G164600 Sapur.001G179800 Sapur.002G012900 Sapur.002G023100 Sapur.002G024200 Sapur.002G028500 Sapur.002G030000 Sapur.002G030100 Sapur.002G033200 Sapur.002G043300 Sapur.002G064600 Sapur.002G069500 Sapur.002G073000 Sapur.002G080700 Sapur.002G091700 Sapur.002G110600 Sapur.002G115400 Sapur.002G125900 Sapur.002G126000 Sapur.002G136900 Sapur.002G142300 Sapur.002G143500 Sapur.002G146000 Sapur.002G151100 Sapur.002G163600 Sapur.002G175800 Sapur.002G190900 Sapur.003G017600 Sapur.003G026500 Sapur.003G047900 Sapur.003G049200 Sapur.003G057500 Sapur.003G067100 Sapur.003G099900 Sapur.003G102000 Sapur.003G111600 Sapur.003G123800 Sapur.003G125900 Sapur.003G138200 Sapur.003G142400 Sapur.003G145700 Sapur.003G150800 Sapur.004G007000 Sapur.004G027800 Sapur.004G030500 Sapur.004G030800 Sapur.004G031600 Sapur.004G054800 Sapur.004G056900 Sapur.004G071100 Sapur.004G100100 Sapur.004G118800 Sapur.004G121500 Sapur.004G123100 Sapur.004G131600 Sapur.004G137000 Sapur.004G171400 Sapur.005G042000 Sapur.005G052900 Sapur.005G054800 Sapur.005G067000 Sapur.005G087400 Sapur.005G098100 Sapur.005G105500 Sapur.005G108500 Sapur.005G133200 Sapur.005G141300 Sapur.005G150000 Sapur.005G163900 Sapur.006G041100 Sapur.006G063200 Sapur.006G067500 Sapur.006G068600 Sapur.006G089400 Sapur.006G103400 Sapur.006G104700 Sapur.006G110100 Sapur.006G114200 Sapur.006G120000 Sapur.006G120300 Sapur.006G122700 Sapur.006G137200 Sapur.006G166000 Sapur.006G168000 Sapur.006G194100 Sapur.006G195200 Sapur.006G197300 Sapur.006G214400 Sapur.006G218000 Sapur.006G228700 Sapur.006G228900 Sapur.007G001300 Sapur.007G005500 Sapur.007G021900 Sapur.007G034000 Sapur.007G035800 Sapur.007G044600 Sapur.007G050900 Sapur.007G058700 Sapur.007G062200 Sapur.007G082500 Sapur.007G087800 Sapur.007G089800 Sapur.007G094000 Sapur.007G114800 Sapur.007G123400 Sapur.007G123700 Sapur.008G034200 Sapur.008G042700 Sapur.008G053100 Sapur.008G064800 Sapur.008G069200 Sapur.008G081900

				Sapur.008G093800 Sapur.008G094300 Sapur.008G096700 Sapur.008G104300 Sapur.008G119700 Sapur.008G121600 Sapur.008G134800 Sapur.008G160400 Sapur.009G002400 Sapur.009G005300 Sapur.009G009700 Sapur.009G011600 Sapur.009G021700 Sapur.009G040000 Sapur.009G094800 Sapur.009G097900 Sapur.009G104900 Sapur.009G112800 Sapur.009G115000 Sapur.009G126000 Sapur.009G126600 Sapur.010G003100 Sapur.010G005200 Sapur.010G013000 Sapur.010G040000 Sapur.010G040100 Sapur.010G046500 Sapur.010G065900 Sapur.010G068700 Sapur.010G096300 Sapur.010G099700 Sapur.010G100200 Sapur.010G113700 Sapur.010G114700 Sapur.010G123400 Sapur.010G128300 Sapur.010G144500 Sapur.010G149100 Sapur.010G154300 Sapur.010G161100 Sapur.011G006600 Sapur.011G032100 Sapur.011G035500 Sapur.011G037900 Sapur.011G038200 Sapur.011G039300 Sapur.011G041100 Sapur.011G042400 Sapur.011G045800 Sapur.011G049300 Sapur.011G054600 Sapur.011G061100 Sapur.011G066500 Sapur.011G079300 Sapur.011G088400 Sapur.011G088500 Sapur.012G004000 Sapur.012G010400 Sapur.012G017700 Sapur.012G018600 Sapur.012G024500 Sapur.012G031400 Sapur.012G051400 Sapur.012G060100 Sapur.012G067900 Sapur.012G078700 Sapur.012G099900 Sapur.013G000400 Sapur.013G008000 Sapur.013G038800 Sapur.013G039500 Sapur.013G041800 Sapur.013G050700 Sapur.013G050900 Sapur.013G055500 Sapur.013G059000 Sapur.013G067900 Sapur.013G068800 Sapur.013G087600 Sapur.013G137400 Sapur.013G141800 Sapur.014G003700 Sapur.014G004000 Sapur.014G005200 Sapur.014G034500 Sapur.014G053000 Sapur.014G059000 Sapur.014G059100 Sapur.014G059200 Sapur.014G065300 Sapur.014G078300 Sapur.014G078500 Sapur.014G079300 Sapur.014G085400 Sapur.014G085500 Sapur.014G094500 Sapur.014G095700 Sapur.014G099500 Sapur.014G101000 Sapur.016G018700 Sapur.016G064600 Sapur.016G070400 Sapur.016G079900 Sapur.016G080200 Sapur.016G085100 Sapur.016G087700 Sapur.016G111900 Sapur.016G114700 Sapur.016G131300 Sapur.016G153200 Sapur.016G159000 Sapur.016G167800 Sapur.016G199700 Sapur.016G216900 Sapur.016G224700 Sapur.016G229800 Sapur.016G233500 Sapur.016G255700 Sapur.016G298800 Sapur.017G004800 Sapur.017G006400 Sapur.017G006500 Sapur.017G007100 Sapur.017G053300 Sapur.017G057100 Sapur.017G069900 Sapur.017G114800 Sapur.017G115700 Sapur.018G007100 Sapur.018G022700 Sapur.018G025000 Sapur.018G034800 Sapur.018G035300 Sapur.018G057700 Sapur.018G067800 Sapur.019G025000 Sapur.019G033200 Sapur.019G039400 Sapur.019G039900 Sapur.019G060100 Sapur.019G074500 Sapur.019G098000 Sapur.019G108100 Sapur.15WG060500 Sapur.15WG062900 Sapur.15WG065100 Sapur.15ZG007300 Sapur.15ZG016900 Sapur.15ZG053200 Sapur.15ZG067200 Sapur.15ZG071900 Sapur.15ZG079000 Sapur.15ZG080900 Sapur.15ZG103400 Sapur.T006300 Sapur.T012000
GO:0080090	278	1172	regulation of primary metabolic process	Sapur.001G017300 Sapur.001G027900 Sapur.001G028100 Sapur.001G028400 Sapur.001G034200 Sapur.001G064300 Sapur.001G067700 Sapur.001G069800 Sapur.001G072500 Sapur.001G075700 Sapur.001G108900 Sapur.001G119600 Sapur.001G129000 Sapur.001G130000 Sapur.001G163400 Sapur.001G164600 Sapur.001G179800 Sapur.002G012900 Sapur.002G023100 Sapur.002G024200 Sapur.002G028500 Sapur.002G030000 Sapur.002G030100 Sapur.002G033200 Sapur.002G043300 Sapur.002G064600 Sapur.002G069500 Sapur.002G073000 Sapur.002G080700 Sapur.002G091700 Sapur.002G110600 Sapur.002G115400 Sapur.002G125900 Sapur.002G126000 Sapur.002G136900 Sapur.002G142300 Sapur.002G143500 Sapur.002G146000 Sapur.002G151100 Sapur.002G163600 Sapur.002G175800

	Sapur.002G190900	Sapur.003G017600	Sapur.003G026500	Sapur.003G047900	Sapur.003G049200	Sapur.003G057500
	Sapur.003G067100	Sapur.003G099900	Sapur.003G102000	Sapur.003G111600	Sapur.003G123800	Sapur.003G125900
	Sapur.003G138200	Sapur.003G145700	Sapur.003G150800	Sapur.004G007000	Sapur.004G027800	Sapur.004G030500
	Sapur.004G030800	Sapur.004G031600	Sapur.004G054800	Sapur.004G056900	Sapur.004G071100	Sapur.004G118800
	Sapur.004G121500	Sapur.004G123100	Sapur.004G131600	Sapur.004G137000	Sapur.004G171400	Sapur.005G042000
	Sapur.005G052900	Sapur.005G054800	Sapur.005G067000	Sapur.005G087400	Sapur.005G098100	Sapur.005G105500
	Sapur.005G108500	Sapur.005G133200	Sapur.005G141300	Sapur.005G150000	Sapur.005G163900	Sapur.006G041100
	Sapur.006G063200	Sapur.006G067500	Sapur.006G068600	Sapur.006G089400	Sapur.006G103400	Sapur.006G104700
	Sapur.006G110100	Sapur.006G114200	Sapur.006G120000	Sapur.006G120300	Sapur.006G122700	Sapur.006G137200
	Sapur.006G166000	Sapur.006G168000	Sapur.006G194100	Sapur.006G195200	Sapur.006G197300	Sapur.006G214400
	Sapur.006G218000	Sapur.006G228700	Sapur.006G228900	Sapur.007G001300	Sapur.007G005500	Sapur.007G021900
	Sapur.007G034000	Sapur.007G035800	Sapur.007G044600	Sapur.007G050900	Sapur.007G058700	Sapur.007G062200
	Sapur.007G082500	Sapur.007G087800	Sapur.007G089800	Sapur.007G094000	Sapur.007G114800	Sapur.007G123400
	Sapur.007G123700	Sapur.008G034200	Sapur.008G042700	Sapur.008G053100	Sapur.008G064800	Sapur.008G069200
	Sapur.008G081900	Sapur.008G093800	Sapur.008G094300	Sapur.008G096700	Sapur.008G104300	Sapur.008G119700
	Sapur.008G121600	Sapur.008G134800	Sapur.008G160400	Sapur.009G005300	Sapur.009G009700	Sapur.009G011600
	Sapur.009G021700	Sapur.009G040000	Sapur.009G094800	Sapur.009G097900	Sapur.009G104900	Sapur.009G112800
	Sapur.009G115000	Sapur.009G126000	Sapur.009G126600	Sapur.010G003100	Sapur.010G005200	Sapur.010G013000
	Sapur.010G040000	Sapur.010G040100	Sapur.010G046500	Sapur.010G065900	Sapur.010G068700	Sapur.010G096300
	Sapur.010G099700	Sapur.010G100200	Sapur.010G113700	Sapur.010G114700	Sapur.010G123400	Sapur.010G128300
	Sapur.010G149100	Sapur.010G154300	Sapur.010G161100	Sapur.011G006600	Sapur.011G032100	Sapur.011G035500
	Sapur.011G037900	Sapur.011G038200	Sapur.011G039300	Sapur.011G041100	Sapur.011G042400	Sapur.011G045800
	Sapur.011G049300	Sapur.011G054600	Sapur.011G061100	Sapur.011G066500	Sapur.011G079300	Sapur.011G088400
	Sapur.011G088500	Sapur.012G004000	Sapur.012G010400	Sapur.012G017700	Sapur.012G018600	Sapur.012G024500
	Sapur.012G031400	Sapur.012G051400	Sapur.012G060100	Sapur.012G067900	Sapur.012G078700	Sapur.012G099900
	Sapur.013G000400	Sapur.013G008000	Sapur.013G038800	Sapur.013G039500	Sapur.013G041800	Sapur.013G050700
	Sapur.013G050900	Sapur.013G055500	Sapur.013G059000	Sapur.013G068800	Sapur.013G087600	Sapur.013G137400
	Sapur.013G141800	Sapur.014G003700	Sapur.014G004000	Sapur.014G005200	Sapur.014G034500	Sapur.014G053000
	Sapur.014G059000	Sapur.014G059100	Sapur.014G059200	Sapur.014G065300	Sapur.014G078300	Sapur.014G078500
	Sapur.014G079300	Sapur.014G085400	Sapur.014G085500	Sapur.014G094500	Sapur.014G095700	Sapur.014G099500
	Sapur.014G101000	Sapur.016G018700	Sapur.016G064600	Sapur.016G070400	Sapur.016G079900	Sapur.016G080200
	Sapur.016G085100	Sapur.016G087700	Sapur.016G111900	Sapur.016G114700	Sapur.016G131300	Sapur.016G153200
	Sapur.016G159000	Sapur.016G167800	Sapur.016G199700	Sapur.016G216900	Sapur.016G224700	Sapur.016G229800

				Sapur.016G233500 Sapur.016G255700 Sapur.016G298800 Sapur.017G004800 Sapur.017G006400 Sapur.017G006500 Sapur.017G007100 Sapur.017G053300 Sapur.017G057100 Sapur.017G069900 Sapur.017G114800 Sapur.017G115700 Sapur.018G007100 Sapur.018G022700 Sapur.018G025000 Sapur.018G034800 Sapur.018G035300 Sapur.018G057700 Sapur.018G067800 Sapur.019G025000 Sapur.019G033200 Sapur.019G039900 Sapur.019G060100 Sapur.019G074500 Sapur.019G098000 Sapur.019G108100 Sapur.15WG060500 Sapur.15WG062900 Sapur.15WG065100 Sapur.15ZG007300 Sapur.15ZG016900 Sapur.15ZG053200 Sapur.15ZG067200 Sapur.15ZG071900 Sapur.15ZG079000 Sapur.15ZG080900 Sapur.15ZG103400 Sapur.T006300 Sapur.T012000
GO:0051171	278	1172	regulation of nitrogen compound metabolic process	Sapur.001G017300 Sapur.001G027900 Sapur.001G028100 Sapur.001G028400 Sapur.001G034200 Sapur.001G064300 Sapur.001G067700 Sapur.001G069800 Sapur.001G072500 Sapur.001G075700 Sapur.001G108900 Sapur.001G119600 Sapur.001G129000 Sapur.001G130000 Sapur.001G163400 Sapur.001G164600 Sapur.001G179800 Sapur.002G012900 Sapur.002G023100 Sapur.002G024200 Sapur.002G028500 Sapur.002G030000 Sapur.002G030100 Sapur.002G033200 Sapur.002G043300 Sapur.002G064600 Sapur.002G069500 Sapur.002G073000 Sapur.002G080700 Sapur.002G091700 Sapur.002G110600 Sapur.002G115400 Sapur.002G125900 Sapur.002G126000 Sapur.002G136900 Sapur.002G142300 Sapur.002G143500 Sapur.002G146000 Sapur.002G151100 Sapur.002G163600 Sapur.002G175800 Sapur.002G190900 Sapur.003G017600 Sapur.003G026500 Sapur.003G047900 Sapur.003G049200 Sapur.003G057500 Sapur.003G067100 Sapur.003G099900 Sapur.003G102000 Sapur.003G111600 Sapur.003G123800 Sapur.003G125900 Sapur.003G138200 Sapur.003G145700 Sapur.003G150800 Sapur.004G007000 Sapur.004G027800 Sapur.004G030500 Sapur.004G030800 Sapur.004G031600 Sapur.004G054800 Sapur.004G056900 Sapur.004G071100 Sapur.004G118800 Sapur.004G121500 Sapur.004G123100 Sapur.004G131600 Sapur.004G137000 Sapur.004G171400 Sapur.005G042000 Sapur.005G052900 Sapur.005G054800 Sapur.005G067000 Sapur.005G087400 Sapur.005G098100 Sapur.005G105500 Sapur.005G108500 Sapur.005G133200 Sapur.005G141300 Sapur.005G150000 Sapur.005G163900 Sapur.006G041100 Sapur.006G063200 Sapur.006G067500 Sapur.006G068600 Sapur.006G089400 Sapur.006G103400 Sapur.006G104700 Sapur.006G110100 Sapur.006G114200 Sapur.006G120000 Sapur.006G120300 Sapur.006G122700 Sapur.006G137200 Sapur.006G166000 Sapur.006G168000 Sapur.006G194100 Sapur.006G195200 Sapur.006G197300 Sapur.006G214400 Sapur.006G218000 Sapur.006G228700 Sapur.006G228900 Sapur.007G001300 Sapur.007G005500 Sapur.007G021900 Sapur.007G034000 Sapur.007G035800 Sapur.007G044600 Sapur.007G050900 Sapur.007G058700 Sapur.007G062200 Sapur.007G082500 Sapur.007G087800 Sapur.007G089800 Sapur.007G094000 Sapur.007G114800 Sapur.007G123400 Sapur.007G123700 Sapur.008G034200 Sapur.008G042700 Sapur.008G053100 Sapur.008G064800 Sapur.008G069200 Sapur.008G081900 Sapur.008G093800 Sapur.008G094300 Sapur.008G096700 Sapur.008G104300 Sapur.008G119700 Sapur.008G121600 Sapur.008G134800 Sapur.008G160400 Sapur.009G005300 Sapur.009G009700 Sapur.009G011600 Sapur.009G021700 Sapur.009G040000 Sapur.009G094800 Sapur.009G097900 Sapur.009G104900 Sapur.009G112800 Sapur.009G115000 Sapur.009G126000 Sapur.009G126600 Sapur.010G003100 Sapur.010G005200 Sapur.010G013000 Sapur.010G040000 Sapur.010G040100 Sapur.010G046500 Sapur.010G065900 Sapur.010G068700 Sapur.010G096300

				Sapur.010G099700 Sapur.010G100200 Sapur.010G113700 Sapur.010G114700 Sapur.010G123400 Sapur.010G128300 Sapur.010G149100 Sapur.010G154300 Sapur.010G161100 Sapur.011G006600 Sapur.011G032100 Sapur.011G035500 Sapur.011G037900 Sapur.011G038200 Sapur.011G039300 Sapur.011G041100 Sapur.011G042400 Sapur.011G045800 Sapur.011G049300 Sapur.011G054600 Sapur.011G061100 Sapur.011G066500 Sapur.011G079300 Sapur.011G088400 Sapur.011G088500 Sapur.012G004000 Sapur.012G010400 Sapur.012G017700 Sapur.012G018600 Sapur.012G024500 Sapur.012G031400 Sapur.012G051400 Sapur.012G060100 Sapur.012G067900 Sapur.012G078700 Sapur.012G099900 Sapur.013G000400 Sapur.013G008000 Sapur.013G038800 Sapur.013G039500 Sapur.013G041800 Sapur.013G050700 Sapur.013G050900 Sapur.013G055500 Sapur.013G059000 Sapur.013G068800 Sapur.013G087600 Sapur.013G137400 Sapur.013G141800 Sapur.014G003700 Sapur.014G004000 Sapur.014G005200 Sapur.014G034500 Sapur.014G053000 Sapur.014G059000 Sapur.014G059100 Sapur.014G059200 Sapur.014G065300 Sapur.014G078300 Sapur.014G078500 Sapur.014G079300 Sapur.014G085400 Sapur.014G085500 Sapur.014G094500 Sapur.014G095700 Sapur.014G099500 Sapur.014G101000 Sapur.016G018700 Sapur.016G064600 Sapur.016G070400 Sapur.016G079900 Sapur.016G080200 Sapur.016G085100 Sapur.016G087700 Sapur.016G111900 Sapur.016G114700 Sapur.016G131300 Sapur.016G153200 Sapur.016G159000 Sapur.016G167800 Sapur.016G199700 Sapur.016G216900 Sapur.016G224700 Sapur.016G229800 Sapur.016G233500 Sapur.016G255700 Sapur.016G298800 Sapur.017G004800 Sapur.017G006400 Sapur.017G006500 Sapur.017G007100 Sapur.017G053300 Sapur.017G057100 Sapur.017G069900 Sapur.017G114800 Sapur.017G115700 Sapur.018G007100 Sapur.018G022700 Sapur.018G025000 Sapur.018G034800 Sapur.018G035300 Sapur.018G057700 Sapur.018G067800 Sapur.019G025000 Sapur.019G033200 Sapur.019G039900 Sapur.019G060100 Sapur.019G074500 Sapur.019G098000 Sapur.019G108100 Sapur.15WG060500 Sapur.15WG062900 Sapur.15WG065100 Sapur.15ZG007300 Sapur.15ZG016900 Sapur.15ZG053200 Sapur.15ZG067200 Sapur.15ZG071900 Sapur.15ZG079000 Sapur.15ZG080900 Sapur.15ZG103400 Sapur.T006300 Sapur.T012000
GO:0031326	280	1187	regulation of cellular biosynthetic process	Sapur.013G002400 Sapur.013G003000 Sapur.013G003100 Sapur.001G017300 Sapur.001G027900 Sapur.001G028100 Sapur.001G028400 Sapur.001G034200 Sapur.001G064300 Sapur.001G067700 Sapur.001G069800 Sapur.001G072500 Sapur.001G075700 Sapur.001G108900 Sapur.001G119600 Sapur.001G129000 Sapur.001G130000 Sapur.001G163400 Sapur.001G164600 Sapur.001G179800 Sapur.002G012900 Sapur.002G023100 Sapur.002G024200 Sapur.002G028500 Sapur.002G030000 Sapur.002G030100 Sapur.002G033200 Sapur.002G043300 Sapur.002G064600 Sapur.002G069500 Sapur.002G073000 Sapur.002G080700 Sapur.002G091700 Sapur.002G110600 Sapur.002G115400 Sapur.002G125900 Sapur.002G126000 Sapur.002G136900 Sapur.002G142300 Sapur.002G143500 Sapur.002G146000 Sapur.002G151100 Sapur.002G163600 Sapur.002G175800 Sapur.002G190900 Sapur.003G017600 Sapur.003G026500 Sapur.003G047900 Sapur.003G049200 Sapur.003G057500 Sapur.003G067100 Sapur.003G099900 Sapur.003G102000 Sapur.003G111600 Sapur.003G123800 Sapur.003G125900 Sapur.003G138200 Sapur.003G142400 Sapur.003G145700 Sapur.003G150800 Sapur.004G007000 Sapur.004G030500 Sapur.004G030800 Sapur.004G031600 Sapur.004G054800 Sapur.004G056900 Sapur.004G100100 Sapur.004G118800 Sapur.004G121500 Sapur.004G123100 Sapur.004G131600

Sapur.004G137000	Sapur.004G171400	Sapur.005G042000	Sapur.005G052900	Sapur.005G054800	Sapur.005G067000
Sapur.005G087400	Sapur.005G098100	Sapur.005G105500	Sapur.005G108500	Sapur.005G133200	Sapur.005G141300
Sapur.005G150000	Sapur.005G163900	Sapur.006G041100	Sapur.006G063200	Sapur.006G067500	Sapur.006G068600
Sapur.006G089400	Sapur.006G103400	Sapur.006G104700	Sapur.006G110100	Sapur.006G114200	Sapur.006G120000
Sapur.006G120300	Sapur.006G122700	Sapur.006G137200	Sapur.006G166000	Sapur.006G168000	Sapur.006G194100
Sapur.006G195200	Sapur.006G197300	Sapur.006G214400	Sapur.006G218000	Sapur.006G228700	Sapur.006G228900
Sapur.007G001300	Sapur.007G005500	Sapur.007G021900	Sapur.007G034000	Sapur.007G035800	Sapur.007G044600
Sapur.007G050900	Sapur.007G058700	Sapur.007G062200	Sapur.007G082500	Sapur.007G087800	Sapur.007G089800
Sapur.007G094000	Sapur.007G114800	Sapur.007G123400	Sapur.008G034200	Sapur.008G042700	Sapur.008G053100
Sapur.008G064800	Sapur.008G069200	Sapur.008G081900	Sapur.008G093800	Sapur.008G094300	Sapur.008G096700
Sapur.008G104300	Sapur.008G119700	Sapur.008G121600	Sapur.008G134800	Sapur.008G160400	Sapur.009G002400
Sapur.009G005300	Sapur.009G009700	Sapur.009G011600	Sapur.009G021700	Sapur.009G040000	Sapur.009G094800
Sapur.009G097900	Sapur.009G104900	Sapur.009G112800	Sapur.009G115000	Sapur.009G126000	Sapur.009G126600
Sapur.010G003100	Sapur.010G005200	Sapur.010G013000	Sapur.010G040000	Sapur.010G040100	Sapur.010G046500
Sapur.010G065900	Sapur.010G068700	Sapur.010G096300	Sapur.010G099700	Sapur.010G100200	Sapur.010G113700
Sapur.010G114700	Sapur.010G123400	Sapur.010G128300	Sapur.010G144500	Sapur.010G149100	Sapur.010G154300
Sapur.010G161100	Sapur.011G006600	Sapur.011G032100	Sapur.011G035500	Sapur.011G037900	Sapur.011G038200
Sapur.011G039300	Sapur.011G041100	Sapur.011G042400	Sapur.011G045800	Sapur.011G049300	Sapur.011G054600
Sapur.011G061100	Sapur.011G066500	Sapur.011G079300	Sapur.011G088400	Sapur.011G088500	Sapur.012G004000
Sapur.012G010400	Sapur.012G017700	Sapur.012G018600	Sapur.012G024500	Sapur.012G031400	Sapur.012G051400
Sapur.012G060100	Sapur.012G067900	Sapur.012G078700	Sapur.013G000400	Sapur.013G008000	Sapur.013G038800
Sapur.013G039500	Sapur.013G041800	Sapur.013G050700	Sapur.013G050900	Sapur.013G055500	Sapur.013G059000
Sapur.013G067900	Sapur.013G068800	Sapur.013G087600	Sapur.013G137400	Sapur.013G141800	Sapur.014G003700
Sapur.014G004000	Sapur.014G005200	Sapur.014G034500	Sapur.014G053000	Sapur.014G059000	Sapur.014G059100
Sapur.014G059200	Sapur.014G065300	Sapur.014G078300	Sapur.014G078500	Sapur.014G079300	Sapur.014G085400
Sapur.014G085500	Sapur.014G094500	Sapur.014G095700	Sapur.014G099500	Sapur.014G101000	Sapur.016G018700
Sapur.016G064600	Sapur.016G070400	Sapur.016G079900	Sapur.016G080200	Sapur.016G085100	Sapur.016G087700
Sapur.016G111900	Sapur.016G114700	Sapur.016G131300	Sapur.016G153200	Sapur.016G159000	Sapur.016G167800
Sapur.016G199700	Sapur.016G216900	Sapur.016G224700	Sapur.016G229800	Sapur.016G233500	Sapur.016G255700
Sapur.016G298800	Sapur.017G004800	Sapur.017G007100	Sapur.017G053300	Sapur.017G057100	Sapur.017G069900
Sapur.017G114800	Sapur.017G115700	Sapur.018G007100	Sapur.018G022700	Sapur.018G025000	Sapur.018G034800
Sapur.018G035300	Sapur.018G057700	Sapur.018G067800	Sapur.019G025000	Sapur.019G033200	Sapur.019G039400
Sapur.019G039900	Sapur.019G060100	Sapur.019G074500	Sapur.019G098000	Sapur.019G108100	Sapur.15WG060500

GO:0010556	280	1187	regulation of macromolecule biosynthetic process	Sapur.15WG065100 Sapur.15ZG007300 Sapur.15ZG016900 Sapur.15ZG053200 Sapur.15ZG067200 Sapur.15ZG071900 Sapur.15ZG079000 Sapur.15ZG080900 Sapur.15ZG103400 Sapur.T006300 Sapur.T012000 Sapur.013G002400 Sapur.013G003000 Sapur.013G003100 Sapur.001G017300 Sapur.001G027900 Sapur.001G028100 Sapur.001G028400 Sapur.001G034200 Sapur.001G064300 Sapur.001G067700 Sapur.001G069800 Sapur.001G072500 Sapur.001G075700 Sapur.001G108900 Sapur.001G119600 Sapur.001G129000 Sapur.001G130000 Sapur.001G163400 Sapur.001G164600 Sapur.001G179800 Sapur.002G012900 Sapur.002G023100 Sapur.002G024200 Sapur.002G028500 Sapur.002G030000 Sapur.002G030100 Sapur.002G033200 Sapur.002G043300 Sapur.002G064600 Sapur.002G069500 Sapur.002G073000 Sapur.002G080700 Sapur.002G091700 Sapur.002G110600 Sapur.002G115400 Sapur.002G125900 Sapur.002G126000 Sapur.002G136900 Sapur.002G142300 Sapur.002G143500 Sapur.002G146000 Sapur.002G151100 Sapur.002G163600 Sapur.002G175800 Sapur.002G190900 Sapur.003G017600 Sapur.003G026500 Sapur.003G047900 Sapur.003G049200 Sapur.003G057500 Sapur.003G067100 Sapur.003G099900 Sapur.003G102000 Sapur.003G111600 Sapur.003G123800 Sapur.003G125900 Sapur.003G138200 Sapur.003G142400 Sapur.003G145700 Sapur.003G150800 Sapur.004G007000 Sapur.004G030500 Sapur.004G030800 Sapur.004G031600 Sapur.004G054800 Sapur.004G056900 Sapur.004G100100 Sapur.004G118800 Sapur.004G121500 Sapur.004G123100 Sapur.004G131600 Sapur.004G137000 Sapur.004G171400 Sapur.005G042000 Sapur.005G052900 Sapur.005G054800 Sapur.005G067000 Sapur.005G087400 Sapur.005G098100 Sapur.005G105500 Sapur.005G108500 Sapur.005G133200 Sapur.005G141300 Sapur.005G150000 Sapur.005G163900 Sapur.006G041100 Sapur.006G063200 Sapur.006G067500 Sapur.006G068600 Sapur.006G089400 Sapur.006G103400 Sapur.006G104700 Sapur.006G110100 Sapur.006G114200 Sapur.006G120000 Sapur.006G120300 Sapur.006G122700 Sapur.006G137200 Sapur.006G166000 Sapur.006G168000 Sapur.006G194100 Sapur.006G195200 Sapur.006G197300 Sapur.006G214400 Sapur.006G218000 Sapur.006G228700 Sapur.006G228900 Sapur.007G001300 Sapur.007G005500 Sapur.007G021900 Sapur.007G034000 Sapur.007G035800 Sapur.007G044600 Sapur.007G050900 Sapur.007G058700 Sapur.007G062200 Sapur.007G082500 Sapur.007G087800 Sapur.007G089800 Sapur.007G094000 Sapur.007G114800 Sapur.007G123400 Sapur.008G034200 Sapur.008G042700 Sapur.008G053100 Sapur.008G064800 Sapur.008G069200 Sapur.008G081900 Sapur.008G093800 Sapur.008G094300 Sapur.008G096700 Sapur.008G104300 Sapur.008G119700 Sapur.008G121600 Sapur.008G134800 Sapur.008G160400 Sapur.009G002400 Sapur.009G005300 Sapur.009G009700 Sapur.009G011600 Sapur.009G021700 Sapur.009G040000 Sapur.009G094800 Sapur.009G097900 Sapur.009G104900 Sapur.009G112800 Sapur.009G115000 Sapur.009G126000 Sapur.009G126600 Sapur.010G003100 Sapur.010G005200 Sapur.010G013000 Sapur.010G040000 Sapur.010G040100 Sapur.010G046500 Sapur.010G065900 Sapur.010G068700 Sapur.010G096300 Sapur.010G099700 Sapur.010G100200 Sapur.010G113700 Sapur.010G114700 Sapur.010G123400 Sapur.010G128300 Sapur.010G144500 Sapur.010G149100 Sapur.010G154300 Sapur.010G161100 Sapur.011G006600 Sapur.011G032100 Sapur.011G035500 Sapur.011G037900 Sapur.011G038200 Sapur.011G039300 Sapur.011G041100 Sapur.011G042400 Sapur.011G045800 Sapur.011G049300 Sapur.011G054600 Sapur.011G061100 Sapur.011G066500 Sapur.011G079300 Sapur.011G088400 Sapur.011G088500 Sapur.012G004000

				Sapur.012G010400 Sapur.012G017700 Sapur.012G018600 Sapur.012G024500 Sapur.012G031400 Sapur.012G051400 Sapur.012G060100 Sapur.012G067900 Sapur.012G078700 Sapur.013G000400 Sapur.013G008000 Sapur.013G038800 Sapur.013G039500 Sapur.013G041800 Sapur.013G050700 Sapur.013G050900 Sapur.013G055500 Sapur.013G059000 Sapur.013G067900 Sapur.013G068800 Sapur.013G087600 Sapur.013G137400 Sapur.013G141800 Sapur.014G003700 Sapur.014G004000 Sapur.014G005200 Sapur.014G034500 Sapur.014G053000 Sapur.014G059000 Sapur.014G059100 Sapur.014G059200 Sapur.014G065300 Sapur.014G078300 Sapur.014G078500 Sapur.014G079300 Sapur.014G085400 Sapur.014G085500 Sapur.014G094500 Sapur.014G095700 Sapur.014G099500 Sapur.014G101000 Sapur.016G018700 Sapur.016G064600 Sapur.016G070400 Sapur.016G079900 Sapur.016G080200 Sapur.016G085100 Sapur.016G087700 Sapur.016G111900 Sapur.016G114700 Sapur.016G131300 Sapur.016G153200 Sapur.016G159000 Sapur.016G167800 Sapur.016G199700 Sapur.016G216900 Sapur.016G224700 Sapur.016G229800 Sapur.016G233500 Sapur.016G255700 Sapur.016G298800 Sapur.017G004800 Sapur.017G007100 Sapur.017G053300 Sapur.017G057100 Sapur.017G069900 Sapur.017G114800 Sapur.017G115700 Sapur.018G007100 Sapur.018G022700 Sapur.018G025000 Sapur.018G034800 Sapur.018G035300 Sapur.018G057700 Sapur.018G067800 Sapur.019G025000 Sapur.019G033200 Sapur.019G039400 Sapur.019G039900 Sapur.019G060100 Sapur.019G074500 Sapur.019G098000 Sapur.019G108100 Sapur.15WG060500 Sapur.15WG065100 Sapur.15ZG007300 Sapur.15ZG016900 Sapur.15ZG053200 Sapur.15ZG067200 Sapur.15ZG071900 Sapur.15ZG079000 Sapur.15ZG080900 Sapur.15ZG103400 Sapur.T006300 Sapur.T012000
GO:0009889	280	1187	regulation of biosynthetic process	Sapur.013G002400 Sapur.013G003000 Sapur.013G003100 Sapur.001G017300 Sapur.001G027900 Sapur.001G028100 Sapur.001G028400 Sapur.001G034200 Sapur.001G064300 Sapur.001G067700 Sapur.001G069800 Sapur.001G072500 Sapur.001G075700 Sapur.001G108900 Sapur.001G119600 Sapur.001G129000 Sapur.001G130000 Sapur.001G163400 Sapur.001G164600 Sapur.001G179800 Sapur.002G012900 Sapur.002G023100 Sapur.002G024200 Sapur.002G028500 Sapur.002G030000 Sapur.002G030100 Sapur.002G033200 Sapur.002G043300 Sapur.002G064600 Sapur.002G069500 Sapur.002G073000 Sapur.002G080700 Sapur.002G091700 Sapur.002G110600 Sapur.002G115400 Sapur.002G125900 Sapur.002G126000 Sapur.002G136900 Sapur.002G142300 Sapur.002G143500 Sapur.002G146000 Sapur.002G151100 Sapur.002G163600 Sapur.002G175800 Sapur.002G190900 Sapur.003G017600 Sapur.003G026500 Sapur.003G047900 Sapur.003G049200 Sapur.003G057500 Sapur.003G067100 Sapur.003G099900 Sapur.003G102000 Sapur.003G111600 Sapur.003G123800 Sapur.003G125900 Sapur.003G138200 Sapur.003G142400 Sapur.003G145700 Sapur.003G150800 Sapur.004G007000 Sapur.004G030500 Sapur.004G030800 Sapur.004G031600 Sapur.004G054800 Sapur.004G056900 Sapur.004G100100 Sapur.004G118800 Sapur.004G121500 Sapur.004G123100 Sapur.004G131600 Sapur.004G137000 Sapur.004G171400 Sapur.005G042000 Sapur.005G052900 Sapur.005G054800 Sapur.005G067000 Sapur.005G087400 Sapur.005G098100 Sapur.005G105500 Sapur.005G108500 Sapur.005G133200 Sapur.005G141300 Sapur.005G150000 Sapur.005G163900 Sapur.006G041100 Sapur.006G063200 Sapur.006G067500 Sapur.006G068600 Sapur.006G089400 Sapur.006G103400 Sapur.006G104700 Sapur.006G110100 Sapur.006G114200 Sapur.006G120000 Sapur.006G120300 Sapur.006G122700 Sapur.006G137200 Sapur.006G166000 Sapur.006G168000 Sapur.006G194100

				Sapur.006G195200 Sapur.006G197300 Sapur.006G214400 Sapur.006G218000 Sapur.006G228700 Sapur.006G228900 Sapur.007G001300 Sapur.007G005500 Sapur.007G021900 Sapur.007G034000 Sapur.007G035800 Sapur.007G044600 Sapur.007G050900 Sapur.007G058700 Sapur.007G062200 Sapur.007G082500 Sapur.007G087800 Sapur.007G089800 Sapur.007G094000 Sapur.007G114800 Sapur.007G123400 Sapur.008G034200 Sapur.008G042700 Sapur.008G053100 Sapur.008G064800 Sapur.008G069200 Sapur.008G081900 Sapur.008G093800 Sapur.008G094300 Sapur.008G096700 Sapur.008G104300 Sapur.008G119700 Sapur.008G121600 Sapur.008G134800 Sapur.008G160400 Sapur.009G002400 Sapur.009G005300 Sapur.009G009700 Sapur.009G011600 Sapur.009G021700 Sapur.009G040000 Sapur.009G094800 Sapur.009G097900 Sapur.009G104900 Sapur.009G112800 Sapur.009G115000 Sapur.009G126000 Sapur.009G126600 Sapur.010G003100 Sapur.010G005200 Sapur.010G013000 Sapur.010G040000 Sapur.010G040100 Sapur.010G046500 Sapur.010G065900 Sapur.010G068700 Sapur.010G096300 Sapur.010G099700 Sapur.010G100200 Sapur.010G113700 Sapur.010G114700 Sapur.010G123400 Sapur.010G128300 Sapur.010G144500 Sapur.010G149100 Sapur.010G154300 Sapur.010G161100 Sapur.011G006600 Sapur.011G032100 Sapur.011G035500 Sapur.011G037900 Sapur.011G038200 Sapur.011G039300 Sapur.011G041100 Sapur.011G042400 Sapur.011G045800 Sapur.011G049300 Sapur.011G054600 Sapur.011G061100 Sapur.011G066500 Sapur.011G079300 Sapur.011G088400 Sapur.011G088500 Sapur.012G004000 Sapur.012G010400 Sapur.012G017700 Sapur.012G018600 Sapur.012G024500 Sapur.012G031400 Sapur.012G051400 Sapur.012G060100 Sapur.012G067900 Sapur.012G078700 Sapur.013G000400 Sapur.013G008000 Sapur.013G038800 Sapur.013G039500 Sapur.013G041800 Sapur.013G050700 Sapur.013G050900 Sapur.013G055500 Sapur.013G059000 Sapur.013G067900 Sapur.013G068800 Sapur.013G087600 Sapur.013G137400 Sapur.013G141800 Sapur.014G003700 Sapur.014G004000 Sapur.014G005200 Sapur.014G034500 Sapur.014G053000 Sapur.014G059000 Sapur.014G059100 Sapur.014G059200 Sapur.014G065300 Sapur.014G078300 Sapur.014G078500 Sapur.014G079300 Sapur.014G085400 Sapur.014G085500 Sapur.014G094500 Sapur.014G095700 Sapur.014G099500 Sapur.014G101000 Sapur.016G018700 Sapur.016G064600 Sapur.016G070400 Sapur.016G079900 Sapur.016G080200 Sapur.016G085100 Sapur.016G087700 Sapur.016G111900 Sapur.016G114700 Sapur.016G131300 Sapur.016G153200 Sapur.016G159000 Sapur.016G167800 Sapur.016G199700 Sapur.016G216900 Sapur.016G224700 Sapur.016G229800 Sapur.016G233500 Sapur.016G255700 Sapur.016G298800 Sapur.017G004800 Sapur.017G007100 Sapur.017G053300 Sapur.017G057100 Sapur.017G069900 Sapur.017G114800 Sapur.017G115700 Sapur.018G007100 Sapur.018G022700 Sapur.018G025000 Sapur.018G034800 Sapur.018G035300 Sapur.018G057700 Sapur.018G067800 Sapur.019G025000 Sapur.019G033200 Sapur.019G039400 Sapur.019G039900 Sapur.019G060100 Sapur.019G074500 Sapur.019G098000 Sapur.019G108100 Sapur.15WG060500 Sapur.15WG065100 Sapur.15ZG007300 Sapur.15ZG016900 Sapur.15ZG053200 Sapur.15ZG067200 Sapur.15ZG071900 Sapur.15ZG079000 Sapur.15ZG080900 Sapur.15ZG103400 Sapur.T006300 Sapur.T012000
GO:0010468	280	1187	regulation of gene expression	Sapur.013G002400 Sapur.013G003000 Sapur.013G003100 Sapur.001G017300 Sapur.001G027900 Sapur.001G028100 Sapur.001G028400 Sapur.001G034200 Sapur.001G064300 Sapur.001G067700 Sapur.001G069800 Sapur.001G072500 Sapur.001G075700 Sapur.001G108900 Sapur.001G119600 Sapur.001G129000 Sapur.001G130000

Sapur.001G163400 Sapur.001G164600 Sapur.001G179800 Sapur.002G012900 Sapur.002G023100 Sapur.002G024200
Sapur.002G028500 Sapur.002G030000 Sapur.002G030100 Sapur.002G033200 Sapur.002G043300 Sapur.002G064600
Sapur.002G069500 Sapur.002G073000 Sapur.002G080700 Sapur.002G091700 Sapur.002G110600 Sapur.002G115400
Sapur.002G125900 Sapur.002G126000 Sapur.002G136900 Sapur.002G142300 Sapur.002G143500 Sapur.002G146000
Sapur.002G151100 Sapur.002G163600 Sapur.002G175800 Sapur.002G190900 Sapur.003G017600 Sapur.003G026500
Sapur.003G047900 Sapur.003G049200 Sapur.003G057500 Sapur.003G067100 Sapur.003G099900 Sapur.003G102000
Sapur.003G111600 Sapur.003G123800 Sapur.003G125900 Sapur.003G138200 Sapur.003G142400 Sapur.003G145700
Sapur.003G150800 Sapur.004G007000 Sapur.004G030500 Sapur.004G030800 Sapur.004G031600 Sapur.004G054800
Sapur.004G056900 Sapur.004G100100 Sapur.004G118800 Sapur.004G121500 Sapur.004G123100 Sapur.004G131600
Sapur.004G137000 Sapur.004G171400 Sapur.005G042000 Sapur.005G052900 Sapur.005G054800 Sapur.005G067000
Sapur.005G087400 Sapur.005G098100 Sapur.005G105500 Sapur.005G108500 Sapur.005G133200 Sapur.005G141300
Sapur.005G150000 Sapur.005G163900 Sapur.006G041100 Sapur.006G063200 Sapur.006G067500 Sapur.006G068600
Sapur.006G089400 Sapur.006G103400 Sapur.006G104700 Sapur.006G110100 Sapur.006G114200 Sapur.006G120000
Sapur.006G120300 Sapur.006G122700 Sapur.006G137200 Sapur.006G166000 Sapur.006G168000 Sapur.006G194100
Sapur.006G195200 Sapur.006G197300 Sapur.006G214400 Sapur.006G218000 Sapur.006G228700 Sapur.006G228900
Sapur.007G001300 Sapur.007G005500 Sapur.007G021900 Sapur.007G034000 Sapur.007G035800 Sapur.007G044600
Sapur.007G050900 Sapur.007G058700 Sapur.007G062200 Sapur.007G082500 Sapur.007G087800 Sapur.007G089800
Sapur.007G094000 Sapur.007G114800 Sapur.007G123400 Sapur.008G034200 Sapur.008G042700 Sapur.008G053100
Sapur.008G064800 Sapur.008G069200 Sapur.008G081900 Sapur.008G093800 Sapur.008G094300 Sapur.008G096700
Sapur.008G104300 Sapur.008G119700 Sapur.008G121600 Sapur.008G134800 Sapur.008G160400 Sapur.009G002400
Sapur.009G005300 Sapur.009G009700 Sapur.009G011600 Sapur.009G021700 Sapur.009G040000 Sapur.009G094800
Sapur.009G097900 Sapur.009G104900 Sapur.009G112800 Sapur.009G115000 Sapur.009G126000 Sapur.009G126600
Sapur.010G003100 Sapur.010G005200 Sapur.010G013000 Sapur.010G040000 Sapur.010G040100 Sapur.010G046500
Sapur.010G065900 Sapur.010G068700 Sapur.010G096300 Sapur.010G099700 Sapur.010G100200 Sapur.010G113700
Sapur.010G114700 Sapur.010G123400 Sapur.010G128300 Sapur.010G144500 Sapur.010G149100 Sapur.010G154300
Sapur.010G161100 Sapur.011G006600 Sapur.011G032100 Sapur.011G035500 Sapur.011G037900 Sapur.011G038200
Sapur.011G039300 Sapur.011G041100 Sapur.011G042400 Sapur.011G045800 Sapur.011G049300 Sapur.011G054600
Sapur.011G061100 Sapur.011G066500 Sapur.011G079300 Sapur.011G088400 Sapur.011G088500 Sapur.012G004000
Sapur.012G010400 Sapur.012G017700 Sapur.012G018600 Sapur.012G024500 Sapur.012G031400 Sapur.012G051400
Sapur.012G060100 Sapur.012G067900 Sapur.012G078700 Sapur.013G000400 Sapur.013G008000 Sapur.013G038800
Sapur.013G039500 Sapur.013G041800 Sapur.013G050700 Sapur.013G050900 Sapur.013G055500 Sapur.013G059000
Sapur.013G067900 Sapur.013G068800 Sapur.013G087600 Sapur.013G137400 Sapur.013G141800 Sapur.014G003700
Sapur.014G004000 Sapur.014G005200 Sapur.014G034500 Sapur.014G053000 Sapur.014G059000 Sapur.014G059100

				Sapur.014G059200 Sapur.014G065300 Sapur.014G078300 Sapur.014G078500 Sapur.014G079300 Sapur.014G085400 Sapur.014G085500 Sapur.014G094500 Sapur.014G095700 Sapur.014G099500 Sapur.014G101000 Sapur.016G018700 Sapur.016G064600 Sapur.016G070400 Sapur.016G079900 Sapur.016G080200 Sapur.016G085100 Sapur.016G087700 Sapur.016G111900 Sapur.016G114700 Sapur.016G131300 Sapur.016G153200 Sapur.016G159000 Sapur.016G167800 Sapur.016G199700 Sapur.016G216900 Sapur.016G224700 Sapur.016G229800 Sapur.016G233500 Sapur.016G255700 Sapur.016G298800 Sapur.017G004800 Sapur.017G007100 Sapur.017G053300 Sapur.017G057100 Sapur.017G069900 Sapur.017G114800 Sapur.017G115700 Sapur.018G007100 Sapur.018G022700 Sapur.018G025000 Sapur.018G034800 Sapur.018G035300 Sapur.018G057700 Sapur.018G067800 Sapur.019G025000 Sapur.019G033200 Sapur.019G039400 Sapur.019G039900 Sapur.019G060100 Sapur.019G074500 Sapur.019G098000 Sapur.019G108100 Sapur.15WG060500 Sapur.15WG065100 Sapur.15ZG007300 Sapur.15ZG016900 Sapur.15ZG053200 Sapur.15ZG067200 Sapur.15ZG071900 Sapur.15ZG079000 Sapur.15ZG080900 Sapur.15ZG103400 Sapur.T006300 Sapur.T012000
GO:0051252	271	1141	regulation of RNA metabolic process	Sapur.001G017300 Sapur.001G027900 Sapur.001G028100 Sapur.001G028400 Sapur.001G034200 Sapur.001G064300 Sapur.001G067700 Sapur.001G069800 Sapur.001G072500 Sapur.001G075700 Sapur.001G108900 Sapur.001G119600 Sapur.001G129000 Sapur.001G130000 Sapur.001G163400 Sapur.001G164600 Sapur.001G179800 Sapur.002G012900 Sapur.002G023100 Sapur.002G024200 Sapur.002G028500 Sapur.002G030000 Sapur.002G030100 Sapur.002G033200 Sapur.002G043300 Sapur.002G064600 Sapur.002G069500 Sapur.002G073000 Sapur.002G080700 Sapur.002G091700 Sapur.002G110600 Sapur.002G115400 Sapur.002G125900 Sapur.002G126000 Sapur.002G136900 Sapur.002G142300 Sapur.002G143500 Sapur.002G146000 Sapur.002G151100 Sapur.002G163600 Sapur.002G175800 Sapur.002G190900 Sapur.003G017600 Sapur.003G026500 Sapur.003G047900 Sapur.003G049200 Sapur.003G057500 Sapur.003G067100 Sapur.003G099900 Sapur.003G102000 Sapur.003G111600 Sapur.003G123800 Sapur.003G125900 Sapur.003G138200 Sapur.003G145700 Sapur.003G150800 Sapur.004G007000 Sapur.004G030500 Sapur.004G030800 Sapur.004G031600 Sapur.004G054800 Sapur.004G056900 Sapur.004G118800 Sapur.004G121500 Sapur.004G123100 Sapur.004G131600 Sapur.004G137000 Sapur.004G171400 Sapur.005G042000 Sapur.005G052900 Sapur.005G054800 Sapur.005G067000 Sapur.005G087400 Sapur.005G098100 Sapur.005G105500 Sapur.005G108500 Sapur.005G133200 Sapur.005G141300 Sapur.005G150000 Sapur.005G163900 Sapur.006G041100 Sapur.006G063200 Sapur.006G067500 Sapur.006G068600 Sapur.006G089400 Sapur.006G103400 Sapur.006G104700 Sapur.006G110100 Sapur.006G114200 Sapur.006G120000 Sapur.006G120300 Sapur.006G122700 Sapur.006G137200 Sapur.006G166000 Sapur.006G168000 Sapur.006G194100 Sapur.006G195200 Sapur.006G197300 Sapur.006G214400 Sapur.006G218000 Sapur.006G228700 Sapur.006G228900 Sapur.007G001300 Sapur.007G005500 Sapur.007G021900 Sapur.007G034000 Sapur.007G035800 Sapur.007G044600 Sapur.007G050900 Sapur.007G058700 Sapur.007G062200 Sapur.007G082500 Sapur.007G087800 Sapur.007G089800 Sapur.007G094000 Sapur.007G114800 Sapur.007G123400 Sapur.008G034200 Sapur.008G042700 Sapur.008G053100 Sapur.008G064800 Sapur.008G069200 Sapur.008G081900 Sapur.008G093800 Sapur.008G094300 Sapur.008G096700 Sapur.008G104300 Sapur.008G119700 Sapur.008G121600 Sapur.008G134800 Sapur.008G160400

				Sapur.009G005300 Sapur.009G009700 Sapur.009G011600 Sapur.009G021700 Sapur.009G040000 Sapur.009G094800 Sapur.009G097900 Sapur.009G104900 Sapur.009G112800 Sapur.009G115000 Sapur.009G126000 Sapur.009G126600 Sapur.010G003100 Sapur.010G005200 Sapur.010G013000 Sapur.010G040000 Sapur.010G040100 Sapur.010G046500 Sapur.010G065900 Sapur.010G068700 Sapur.010G096300 Sapur.010G099700 Sapur.010G100200 Sapur.010G113700 Sapur.010G114700 Sapur.010G123400 Sapur.010G128300 Sapur.010G149100 Sapur.010G154300 Sapur.010G161100 Sapur.011G006600 Sapur.011G032100 Sapur.011G035500 Sapur.011G037900 Sapur.011G038200 Sapur.011G039300 Sapur.011G041100 Sapur.011G042400 Sapur.011G045800 Sapur.011G049300 Sapur.011G054600 Sapur.011G061100 Sapur.011G066500 Sapur.011G079300 Sapur.011G088400 Sapur.011G088500 Sapur.012G004000 Sapur.012G010400 Sapur.012G017700 Sapur.012G018600 Sapur.012G024500 Sapur.012G031400 Sapur.012G051400 Sapur.012G060100 Sapur.012G067900 Sapur.012G078700 Sapur.013G000400 Sapur.013G008000 Sapur.013G038800 Sapur.013G039500 Sapur.013G041800 Sapur.013G050700 Sapur.013G050900 Sapur.013G055500 Sapur.013G059000 Sapur.013G068800 Sapur.013G087600 Sapur.013G137400 Sapur.013G141800 Sapur.014G003700 Sapur.014G004000 Sapur.014G005200 Sapur.014G034500 Sapur.014G053000 Sapur.014G059000 Sapur.014G059100 Sapur.014G059200 Sapur.014G065300 Sapur.014G078300 Sapur.014G078500 Sapur.014G079300 Sapur.014G085400 Sapur.014G085500 Sapur.014G094500 Sapur.014G095700 Sapur.014G099500 Sapur.014G101000 Sapur.016G018700 Sapur.016G064600 Sapur.016G070400 Sapur.016G079900 Sapur.016G080200 Sapur.016G085100 Sapur.016G087700 Sapur.016G111900 Sapur.016G114700 Sapur.016G131300 Sapur.016G153200 Sapur.016G159000 Sapur.016G167800 Sapur.016G199700 Sapur.016G216900 Sapur.016G224700 Sapur.016G229800 Sapur.016G233500 Sapur.016G255700 Sapur.016G298800 Sapur.017G004800 Sapur.017G007100 Sapur.017G053300 Sapur.017G057100 Sapur.017G069900 Sapur.017G114800 Sapur.017G115700 Sapur.018G007100 Sapur.018G022700 Sapur.018G025000 Sapur.018G034800 Sapur.018G035300 Sapur.018G057700 Sapur.018G067800 Sapur.019G025000 Sapur.019G033200 Sapur.019G039900 Sapur.019G060100 Sapur.019G074500 Sapur.019G098000 Sapur.019G108100 Sapur.15WG060500 Sapur.15WG065100 Sapur.15ZG007300 Sapur.15ZG016900 Sapur.15ZG053200 Sapur.15ZG067200 Sapur.15ZG071900 Sapur.15ZG079000 Sapur.15ZG080900 Sapur.15ZG103400 Sapur.T006300 Sapur.T012000
GO:2001141	271	1141	regulation of RNA biosynthetic process	Sapur.001G017300 Sapur.001G027900 Sapur.001G028100 Sapur.001G028400 Sapur.001G034200 Sapur.001G064300 Sapur.001G067700 Sapur.001G069800 Sapur.001G072500 Sapur.001G075700 Sapur.001G108900 Sapur.001G119600 Sapur.001G129000 Sapur.001G130000 Sapur.001G163400 Sapur.001G164600 Sapur.001G179800 Sapur.002G012900 Sapur.002G023100 Sapur.002G024200 Sapur.002G028500 Sapur.002G030000 Sapur.002G030100 Sapur.002G033200 Sapur.002G043300 Sapur.002G064600 Sapur.002G069500 Sapur.002G073000 Sapur.002G080700 Sapur.002G091700 Sapur.002G110600 Sapur.002G115400 Sapur.002G125900 Sapur.002G126000 Sapur.002G136900 Sapur.002G142300 Sapur.002G143500 Sapur.002G146000 Sapur.002G151100 Sapur.002G163600 Sapur.002G175800 Sapur.002G190900 Sapur.003G017600 Sapur.003G026500 Sapur.003G047900 Sapur.003G049200 Sapur.003G057500 Sapur.003G067100 Sapur.003G099900 Sapur.003G102000 Sapur.003G111600 Sapur.003G123800 Sapur.003G125900

	Sapur.003G138200	Sapur.003G145700	Sapur.003G150800	Sapur.004G007000	Sapur.004G030500	Sapur.004G030800
	Sapur.004G031600	Sapur.004G054800	Sapur.004G056900	Sapur.004G118800	Sapur.004G121500	Sapur.004G123100
	Sapur.004G131600	Sapur.004G137000	Sapur.004G171400	Sapur.005G042000	Sapur.005G052900	Sapur.005G054800
	Sapur.005G067000	Sapur.005G087400	Sapur.005G098100	Sapur.005G105500	Sapur.005G108500	Sapur.005G133200
	Sapur.005G141300	Sapur.005G150000	Sapur.005G163900	Sapur.006G041100	Sapur.006G063200	Sapur.006G067500
	Sapur.006G068600	Sapur.006G089400	Sapur.006G103400	Sapur.006G104700	Sapur.006G110100	Sapur.006G114200
	Sapur.006G120000	Sapur.006G120300	Sapur.006G122700	Sapur.006G137200	Sapur.006G166000	Sapur.006G168000
	Sapur.006G194100	Sapur.006G195200	Sapur.006G197300	Sapur.006G214400	Sapur.006G218000	Sapur.006G228700
	Sapur.006G228900	Sapur.007G001300	Sapur.007G005500	Sapur.007G021900	Sapur.007G034000	Sapur.007G035800
	Sapur.007G044600	Sapur.007G050900	Sapur.007G058700	Sapur.007G062200	Sapur.007G082500	Sapur.007G087800
	Sapur.007G089800	Sapur.007G094000	Sapur.007G114800	Sapur.007G123400	Sapur.008G034200	Sapur.008G042700
	Sapur.008G053100	Sapur.008G064800	Sapur.008G069200	Sapur.008G081900	Sapur.008G093800	Sapur.008G094300
	Sapur.008G096700	Sapur.008G104300	Sapur.008G119700	Sapur.008G121600	Sapur.008G134800	Sapur.008G160400
	Sapur.009G005300	Sapur.009G009700	Sapur.009G011600	Sapur.009G021700	Sapur.009G040000	Sapur.009G094800
	Sapur.009G097900	Sapur.009G104900	Sapur.009G112800	Sapur.009G115000	Sapur.009G126000	Sapur.009G126600
	Sapur.010G003100	Sapur.010G005200	Sapur.010G013000	Sapur.010G040000	Sapur.010G040100	Sapur.010G046500
	Sapur.010G065900	Sapur.010G068700	Sapur.010G096300	Sapur.010G099700	Sapur.010G100200	Sapur.010G113700
	Sapur.010G114700	Sapur.010G123400	Sapur.010G128300	Sapur.010G149100	Sapur.010G154300	Sapur.010G161100
	Sapur.011G006600	Sapur.011G032100	Sapur.011G035500	Sapur.011G037900	Sapur.011G038200	Sapur.011G039300
	Sapur.011G041100	Sapur.011G042400	Sapur.011G045800	Sapur.011G049300	Sapur.011G054600	Sapur.011G061100
	Sapur.011G066500	Sapur.011G079300	Sapur.011G088400	Sapur.011G088500	Sapur.012G004000	Sapur.012G010400
	Sapur.012G017700	Sapur.012G018600	Sapur.012G024500	Sapur.012G031400	Sapur.012G051400	Sapur.012G060100
	Sapur.012G067900	Sapur.012G078700	Sapur.013G000400	Sapur.013G008000	Sapur.013G038800	Sapur.013G039500
	Sapur.013G041800	Sapur.013G050700	Sapur.013G050900	Sapur.013G055500	Sapur.013G059000	Sapur.013G068800
	Sapur.013G087600	Sapur.013G137400	Sapur.013G141800	Sapur.014G003700	Sapur.014G004000	Sapur.014G005200
	Sapur.014G034500	Sapur.014G053000	Sapur.014G059000	Sapur.014G059100	Sapur.014G059200	Sapur.014G065300
	Sapur.014G078300	Sapur.014G078500	Sapur.014G079300	Sapur.014G085400	Sapur.014G085500	Sapur.014G094500
	Sapur.014G095700	Sapur.014G099500	Sapur.014G101000	Sapur.016G018700	Sapur.016G064600	Sapur.016G070400
	Sapur.016G079900	Sapur.016G080200	Sapur.016G085100	Sapur.016G087700	Sapur.016G111900	Sapur.016G114700
	Sapur.016G131300	Sapur.016G153200	Sapur.016G159000	Sapur.016G167800	Sapur.016G199700	Sapur.016G216900
	Sapur.016G224700	Sapur.016G229800	Sapur.016G233500	Sapur.016G255700	Sapur.016G298800	Sapur.017G004800
	Sapur.017G007100	Sapur.017G053300	Sapur.017G057100	Sapur.017G069900	Sapur.017G114800	Sapur.017G115700
	Sapur.018G007100	Sapur.018G022700	Sapur.018G025000	Sapur.018G034800	Sapur.018G035300	Sapur.018G057700

				Sapur.018G067800 Sapur.019G025000 Sapur.019G033200 Sapur.019G039900 Sapur.019G060100 Sapur.019G074500 Sapur.019G098000 Sapur.019G108100 Sapur.15WG060500 Sapur.15WG065100 Sapur.15ZG007300 Sapur.15ZG016900 Sapur.15ZG053200 Sapur.15ZG067200 Sapur.15ZG071900 Sapur.15ZG079000 Sapur.15ZG080900 Sapur.15ZG103400 Sapur.T006300 Sapur.T012000
GO:0006355	271	1141	regulation of DNA-templated transcription	Sapur.001G017300 Sapur.001G027900 Sapur.001G028100 Sapur.001G028400 Sapur.001G034200 Sapur.001G064300 Sapur.001G067700 Sapur.001G069800 Sapur.001G072500 Sapur.001G075700 Sapur.001G108900 Sapur.001G119600 Sapur.001G129000 Sapur.001G130000 Sapur.001G163400 Sapur.001G164600 Sapur.001G179800 Sapur.002G012900 Sapur.002G023100 Sapur.002G024200 Sapur.002G028500 Sapur.002G030000 Sapur.002G030100 Sapur.002G033200 Sapur.002G043300 Sapur.002G064600 Sapur.002G069500 Sapur.002G073000 Sapur.002G080700 Sapur.002G091700 Sapur.002G110600 Sapur.002G115400 Sapur.002G125900 Sapur.002G126000 Sapur.002G136900 Sapur.002G142300 Sapur.002G143500 Sapur.002G146000 Sapur.002G151100 Sapur.002G163600 Sapur.002G175800 Sapur.002G190900 Sapur.003G017600 Sapur.003G026500 Sapur.003G047900 Sapur.003G049200 Sapur.003G057500 Sapur.003G067100 Sapur.003G099900 Sapur.003G102000 Sapur.003G111600 Sapur.003G123800 Sapur.003G125900 Sapur.003G138200 Sapur.003G145700 Sapur.003G150800 Sapur.004G007000 Sapur.004G030500 Sapur.004G030800 Sapur.004G031600 Sapur.004G054800 Sapur.004G056900 Sapur.004G118800 Sapur.004G121500 Sapur.004G123100 Sapur.004G131600 Sapur.004G137000 Sapur.004G171400 Sapur.005G042000 Sapur.005G052900 Sapur.005G054800 Sapur.005G067000 Sapur.005G087400 Sapur.005G098100 Sapur.005G105500 Sapur.005G108500 Sapur.005G133200 Sapur.005G141300 Sapur.005G150000 Sapur.005G163900 Sapur.006G041100 Sapur.006G063200 Sapur.006G067500 Sapur.006G068600 Sapur.006G089400 Sapur.006G103400 Sapur.006G104700 Sapur.006G110100 Sapur.006G114200 Sapur.006G120000 Sapur.006G120300 Sapur.006G122700 Sapur.006G137200 Sapur.006G166000 Sapur.006G168000 Sapur.006G194100 Sapur.006G195200 Sapur.006G197300 Sapur.006G214400 Sapur.006G218000 Sapur.006G228700 Sapur.006G228900 Sapur.007G001300 Sapur.007G005500 Sapur.007G021900 Sapur.007G034000 Sapur.007G035800 Sapur.007G044600 Sapur.007G050900 Sapur.007G058700 Sapur.007G062200 Sapur.007G082500 Sapur.007G087800 Sapur.007G089800 Sapur.007G094000 Sapur.007G114800 Sapur.007G123400 Sapur.008G034200 Sapur.008G042700 Sapur.008G053100 Sapur.008G064800 Sapur.008G069200 Sapur.008G081900 Sapur.008G093800 Sapur.008G094300 Sapur.008G096700 Sapur.008G104300 Sapur.008G119700 Sapur.008G121600 Sapur.008G134800 Sapur.008G160400 Sapur.009G005300 Sapur.009G009700 Sapur.009G011600 Sapur.009G021700 Sapur.009G040000 Sapur.009G094800 Sapur.009G097900 Sapur.009G104900 Sapur.009G112800 Sapur.009G115000 Sapur.009G126000 Sapur.009G126600 Sapur.010G003100 Sapur.010G005200 Sapur.010G013000 Sapur.010G040000 Sapur.010G040100 Sapur.010G046500 Sapur.010G065900 Sapur.010G068700 Sapur.010G096300 Sapur.010G099700 Sapur.010G100200 Sapur.010G113700 Sapur.010G114700 Sapur.010G123400 Sapur.010G128300 Sapur.010G149100 Sapur.010G154300 Sapur.010G161100 Sapur.011G006600 Sapur.011G032100 Sapur.011G035500 Sapur.011G037900 Sapur.011G038200 Sapur.011G039300 Sapur.011G041100 Sapur.011G042400 Sapur.011G045800 Sapur.011G049300 Sapur.011G054600 Sapur.011G061100

				Sapur.011G066500 Sapur.011G079300 Sapur.011G088400 Sapur.011G088500 Sapur.012G004000 Sapur.012G010400 Sapur.012G017700 Sapur.012G018600 Sapur.012G024500 Sapur.012G031400 Sapur.012G051400 Sapur.012G060100 Sapur.012G067900 Sapur.012G078700 Sapur.013G000400 Sapur.013G008000 Sapur.013G038800 Sapur.013G039500 Sapur.013G041800 Sapur.013G050700 Sapur.013G050900 Sapur.013G055500 Sapur.013G059000 Sapur.013G068800 Sapur.013G087600 Sapur.013G137400 Sapur.013G141800 Sapur.014G003700 Sapur.014G004000 Sapur.014G005200 Sapur.014G034500 Sapur.014G053000 Sapur.014G059000 Sapur.014G059100 Sapur.014G059200 Sapur.014G065300 Sapur.014G078300 Sapur.014G078500 Sapur.014G079300 Sapur.014G085400 Sapur.014G085500 Sapur.014G094500 Sapur.014G095700 Sapur.014G099500 Sapur.014G101000 Sapur.016G018700 Sapur.016G064600 Sapur.016G070400 Sapur.016G079900 Sapur.016G080200 Sapur.016G085100 Sapur.016G087700 Sapur.016G111900 Sapur.016G114700 Sapur.016G131300 Sapur.016G153200 Sapur.016G159000 Sapur.016G167800 Sapur.016G199700 Sapur.016G216900 Sapur.016G224700 Sapur.016G229800 Sapur.016G233500 Sapur.016G255700 Sapur.016G298800 Sapur.017G004800 Sapur.017G007100 Sapur.017G053300 Sapur.017G057100 Sapur.017G069900 Sapur.017G114800 Sapur.017G115700 Sapur.018G007100 Sapur.018G022700 Sapur.018G025000 Sapur.018G034800 Sapur.018G035300 Sapur.018G057700 Sapur.018G067800 Sapur.019G025000 Sapur.019G033200 Sapur.019G039900 Sapur.019G060100 Sapur.019G074500 Sapur.019G098000 Sapur.019G108100 Sapur.15WG060500 Sapur.15WG065100 Sapur.15ZG007300 Sapur.15ZG016900 Sapur.15ZG053200 Sapur.15ZG067200 Sapur.15ZG071900 Sapur.15ZG079000 Sapur.15ZG080900 Sapur.15ZG103400 Sapur.T006300 Sapur.T012000
GO:0019219	272	1148	regulation of nucleobase-containing compound metabolic process	Sapur.001G017300 Sapur.001G027900 Sapur.001G028100 Sapur.001G028400 Sapur.001G034200 Sapur.001G064300 Sapur.001G067700 Sapur.001G069800 Sapur.001G072500 Sapur.001G075700 Sapur.001G108900 Sapur.001G119600 Sapur.001G129000 Sapur.001G130000 Sapur.001G163400 Sapur.001G164600 Sapur.001G179800 Sapur.002G012900 Sapur.002G023100 Sapur.002G024200 Sapur.002G028500 Sapur.002G030000 Sapur.002G030100 Sapur.002G033200 Sapur.002G043300 Sapur.002G064600 Sapur.002G069500 Sapur.002G073000 Sapur.002G080700 Sapur.002G091700 Sapur.002G110600 Sapur.002G115400 Sapur.002G125900 Sapur.002G126000 Sapur.002G136900 Sapur.002G142300 Sapur.002G143500 Sapur.002G146000 Sapur.002G151100 Sapur.002G163600 Sapur.002G175800 Sapur.002G190900 Sapur.003G017600 Sapur.003G026500 Sapur.003G047900 Sapur.003G049200 Sapur.003G057500 Sapur.003G067100 Sapur.003G099900 Sapur.003G102000 Sapur.003G111600 Sapur.003G123800 Sapur.003G125900 Sapur.003G138200 Sapur.003G145700 Sapur.003G150800 Sapur.004G007000 Sapur.004G027800 Sapur.004G030500 Sapur.004G030800 Sapur.004G031600 Sapur.004G054800 Sapur.004G056900 Sapur.004G118800 Sapur.004G121500 Sapur.004G123100 Sapur.004G131600 Sapur.004G137000 Sapur.004G171400 Sapur.005G042000 Sapur.005G052900 Sapur.005G054800 Sapur.005G067000 Sapur.005G087400 Sapur.005G098100 Sapur.005G105500 Sapur.005G108500 Sapur.005G133200 Sapur.005G141300 Sapur.005G150000 Sapur.005G163900 Sapur.006G041100 Sapur.006G063200 Sapur.006G067500 Sapur.006G068600 Sapur.006G089400 Sapur.006G103400 Sapur.006G104700 Sapur.006G110100 Sapur.006G114200 Sapur.006G120000 Sapur.006G120300 Sapur.006G122700 Sapur.006G137200 Sapur.006G166000

				Sapur.006G168000 Sapur.006G194100 Sapur.006G195200 Sapur.006G197300 Sapur.006G214400 Sapur.006G218000 Sapur.006G228700 Sapur.006G228900 Sapur.007G001300 Sapur.007G005500 Sapur.007G021900 Sapur.007G034000 Sapur.007G035800 Sapur.007G044600 Sapur.007G050900 Sapur.007G058700 Sapur.007G062200 Sapur.007G082500 Sapur.007G087800 Sapur.007G089800 Sapur.007G094000 Sapur.007G114800 Sapur.007G123400 Sapur.008G034200 Sapur.008G042700 Sapur.008G053100 Sapur.008G064800 Sapur.008G069200 Sapur.008G081900 Sapur.008G093800 Sapur.008G094300 Sapur.008G096700 Sapur.008G104300 Sapur.008G119700 Sapur.008G121600 Sapur.008G134800 Sapur.008G160400 Sapur.009G005300 Sapur.009G009700 Sapur.009G011600 Sapur.009G021700 Sapur.009G040000 Sapur.009G094800 Sapur.009G097900 Sapur.009G104900 Sapur.009G112800 Sapur.009G115000 Sapur.009G126000 Sapur.009G126600 Sapur.010G003100 Sapur.010G005200 Sapur.010G013000 Sapur.010G040000 Sapur.010G040100 Sapur.010G046500 Sapur.010G065900 Sapur.010G068700 Sapur.010G096300 Sapur.010G099700 Sapur.010G100200 Sapur.010G113700 Sapur.010G114700 Sapur.010G123400 Sapur.010G128300 Sapur.010G149100 Sapur.010G154300 Sapur.010G161100 Sapur.011G006600 Sapur.011G032100 Sapur.011G035500 Sapur.011G037900 Sapur.011G038200 Sapur.011G039300 Sapur.011G041100 Sapur.011G042400 Sapur.011G045800 Sapur.011G049300 Sapur.011G054600 Sapur.011G061100 Sapur.011G066500 Sapur.011G079300 Sapur.011G088400 Sapur.011G088500 Sapur.012G004000 Sapur.012G010400 Sapur.012G017700 Sapur.012G018600 Sapur.012G024500 Sapur.012G031400 Sapur.012G051400 Sapur.012G060100 Sapur.012G067900 Sapur.012G078700 Sapur.013G000400 Sapur.013G008000 Sapur.013G038800 Sapur.013G039500 Sapur.013G041800 Sapur.013G050700 Sapur.013G050900 Sapur.013G055500 Sapur.013G059000 Sapur.013G068800 Sapur.013G087600 Sapur.013G137400 Sapur.013G141800 Sapur.014G003700 Sapur.014G004000 Sapur.014G005200 Sapur.014G034500 Sapur.014G053000 Sapur.014G059000 Sapur.014G059100 Sapur.014G059200 Sapur.014G065300 Sapur.014G078300 Sapur.014G078500 Sapur.014G079300 Sapur.014G085400 Sapur.014G085500 Sapur.014G094500 Sapur.014G095700 Sapur.014G099500 Sapur.014G101000 Sapur.016G018700 Sapur.016G064600 Sapur.016G070400 Sapur.016G079900 Sapur.016G080200 Sapur.016G085100 Sapur.016G087700 Sapur.016G111900 Sapur.016G114700 Sapur.016G131300 Sapur.016G153200 Sapur.016G159000 Sapur.016G167800 Sapur.016G199700 Sapur.016G216900 Sapur.016G224700 Sapur.016G229800 Sapur.016G233500 Sapur.016G255700 Sapur.016G298800 Sapur.017G004800 Sapur.017G007100 Sapur.017G053300 Sapur.017G057100 Sapur.017G069900 Sapur.017G114800 Sapur.017G115700 Sapur.018G007100 Sapur.018G022700 Sapur.018G025000 Sapur.018G034800 Sapur.018G035300 Sapur.018G057700 Sapur.018G067800 Sapur.019G025000 Sapur.019G033200 Sapur.019G039900 Sapur.019G060100 Sapur.019G074500 Sapur.019G098000 Sapur.019G108100 Sapur.15WG060500 Sapur.15WG065100 Sapur.15ZG007300 Sapur.15ZG016900 Sapur.15ZG053200 Sapur.15ZG067200 Sapur.15ZG071900 Sapur.15ZG079000 Sapur.15ZG080900 Sapur.15ZG103400 Sapur.T006300 Sapur.T012000
GO:0019438	315	1389	aromatic compound biosynthetic process	Sapur.001G017300 Sapur.001G025400 Sapur.001G027900 Sapur.001G028100 Sapur.001G028400 Sapur.001G034200 Sapur.001G064300 Sapur.001G067700 Sapur.001G069800 Sapur.001G072500 Sapur.001G075700 Sapur.001G090900 Sapur.001G108900 Sapur.001G119600 Sapur.001G129000 Sapur.001G130000 Sapur.001G163400

Sapur.001G164600 Sapur.001G179800 Sapur.001G183500 Sapur.002G012900 Sapur.002G023100 Sapur.002G024200
Sapur.002G028500 Sapur.002G030000 Sapur.002G030100 Sapur.002G033200 Sapur.002G043300 Sapur.002G064600
Sapur.002G069500 Sapur.002G073000 Sapur.002G080700 Sapur.002G091700 Sapur.002G110600 Sapur.002G112100
Sapur.002G115400 Sapur.002G125900 Sapur.002G126000 Sapur.002G128100 Sapur.002G136900 Sapur.002G142300
Sapur.002G143500 Sapur.002G146000 Sapur.002G151100 Sapur.002G163600 Sapur.002G175800 Sapur.002G190900
Sapur.003G017600 Sapur.003G026500 Sapur.003G047900 Sapur.003G049200 Sapur.003G057500 Sapur.003G067100
Sapur.003G070500 Sapur.003G091600 Sapur.003G092400 Sapur.003G099900 Sapur.003G102000 Sapur.003G111600
Sapur.003G123800 Sapur.003G125900 Sapur.003G138200 Sapur.003G145700 Sapur.003G150800 Sapur.003G152800
Sapur.004G007000 Sapur.004G008500 Sapur.004G030500 Sapur.004G030800 Sapur.004G031600 Sapur.004G054800
Sapur.004G056900 Sapur.004G118800 Sapur.004G121500 Sapur.004G123100 Sapur.004G131600 Sapur.004G137000
Sapur.004G171400 Sapur.005G042000 Sapur.005G052900 Sapur.005G054800 Sapur.005G055100 Sapur.005G067000
Sapur.005G087400 Sapur.005G098100 Sapur.005G105500 Sapur.005G108500 Sapur.005G133200 Sapur.005G141300
Sapur.005G150000 Sapur.005G163900 Sapur.006G041100 Sapur.006G043000 Sapur.006G049700 Sapur.006G063200
Sapur.006G067500 Sapur.006G068600 Sapur.006G070000 Sapur.006G083400 Sapur.006G089400 Sapur.006G103400
Sapur.006G104700 Sapur.006G110100 Sapur.006G114200 Sapur.006G118700 Sapur.006G120000 Sapur.006G120300
Sapur.006G122700 Sapur.006G137200 Sapur.006G166000 Sapur.006G168000 Sapur.006G194100 Sapur.006G195200
Sapur.006G197300 Sapur.006G214400 Sapur.006G218000 Sapur.006G228700 Sapur.006G228900 Sapur.007G001300
Sapur.007G005500 Sapur.007G021900 Sapur.007G034000 Sapur.007G035800 Sapur.007G044600 Sapur.007G050900
Sapur.007G058700 Sapur.007G062200 Sapur.007G082500 Sapur.007G087500 Sapur.007G087800 Sapur.007G089800
Sapur.007G094000 Sapur.007G114800 Sapur.007G123400 Sapur.008G034200 Sapur.008G042700 Sapur.008G053100
Sapur.008G064800 Sapur.008G069200 Sapur.008G081900 Sapur.008G090400 Sapur.008G093800 Sapur.008G094300
Sapur.008G096700 Sapur.008G104300 Sapur.008G119700 Sapur.008G121600 Sapur.008G134800 Sapur.008G160400
Sapur.009G005300 Sapur.009G009700 Sapur.009G011600 Sapur.009G021700 Sapur.009G040000 Sapur.009G094800
Sapur.009G097900 Sapur.009G104900 Sapur.009G112800 Sapur.009G115000 Sapur.009G126000 Sapur.009G126600
Sapur.010G003100 Sapur.010G005200 Sapur.010G013000 Sapur.010G040000 Sapur.010G040100 Sapur.010G046500
Sapur.010G065900 Sapur.010G068700 Sapur.010G086900 Sapur.010G096300 Sapur.010G099700 Sapur.010G100200
Sapur.010G113700 Sapur.010G114700 Sapur.010G123400 Sapur.010G128300 Sapur.010G134600 Sapur.010G149100
Sapur.010G151500 Sapur.010G154300 Sapur.010G161100 Sapur.011G006600 Sapur.011G014000 Sapur.011G032100
Sapur.011G035500 Sapur.011G037900 Sapur.011G038200 Sapur.011G039300 Sapur.011G041100 Sapur.011G042400
Sapur.011G045800 Sapur.011G049300 Sapur.011G054600 Sapur.011G061100 Sapur.011G064300 Sapur.011G066500
Sapur.011G079300 Sapur.011G088400 Sapur.011G088500 Sapur.012G004000 Sapur.012G010400 Sapur.012G017700
Sapur.012G018600 Sapur.012G024500 Sapur.012G031400 Sapur.012G051400 Sapur.012G055400 Sapur.012G060100
Sapur.012G067900 Sapur.012G078700 Sapur.012G084400 Sapur.012G105100 Sapur.013G000400 Sapur.013G008000

				Sapur.013G038800 Sapur.013G039500 Sapur.013G041800 Sapur.013G050700 Sapur.013G050900 Sapur.013G055500 Sapur.013G059000 Sapur.013G068800 Sapur.013G087600 Sapur.013G108500 Sapur.013G137400 Sapur.013G141800 Sapur.014G003700 Sapur.014G004000 Sapur.014G005200 Sapur.014G034500 Sapur.014G053000 Sapur.014G059000 Sapur.014G059100 Sapur.014G059200 Sapur.014G065300 Sapur.014G078300 Sapur.014G078500 Sapur.014G079300 Sapur.014G085400 Sapur.014G085500 Sapur.014G094500 Sapur.014G095700 Sapur.014G099500 Sapur.014G101000 Sapur.016G018700 Sapur.016G030200 Sapur.016G064600 Sapur.016G070400 Sapur.016G079900 Sapur.016G080200 Sapur.016G085100 Sapur.016G087700 Sapur.016G101200 Sapur.016G104000 Sapur.016G111900 Sapur.016G114700 Sapur.016G131300 Sapur.016G153200 Sapur.016G159000 Sapur.016G167800 Sapur.016G199700 Sapur.016G216900 Sapur.016G224700 Sapur.016G229800 Sapur.016G233500 Sapur.016G255700 Sapur.016G298800 Sapur.017G004800 Sapur.017G007100 Sapur.017G053300 Sapur.017G057100 Sapur.017G069900 Sapur.017G085900 Sapur.017G106300 Sapur.017G114800 Sapur.017G115700 Sapur.018G007100 Sapur.018G022500 Sapur.018G022700 Sapur.018G025000 Sapur.018G034800 Sapur.018G035300 Sapur.018G057700 Sapur.018G067800 Sapur.019G025000 Sapur.019G033200 Sapur.019G039900 Sapur.019G060100 Sapur.019G074500 Sapur.019G098000 Sapur.019G101700 Sapur.019G108100 Sapur.15WG060500 Sapur.15WG063000 Sapur.15WG065100 Sapur.15ZG002000 Sapur.15ZG007300 Sapur.15ZG016900 Sapur.15ZG053200 Sapur.15ZG067200 Sapur.15ZG071900 Sapur.15ZG079000 Sapur.15ZG080900 Sapur.15ZG085300 Sapur.15ZG103400 Sapur.T006300 Sapur.T012000 Sapur.003G056300 Sapur.002G029000 Sapur.001G057600 Sapur.003G116600 Sapur.008G063700 Sapur.010G137300 Sapur.014G098600
GO:0034654	308	1354	nucleobase-containing compound biosynthetic process	Sapur.001G017300 Sapur.001G025400 Sapur.001G027900 Sapur.001G028100 Sapur.001G028400 Sapur.001G034200 Sapur.001G064300 Sapur.001G067700 Sapur.001G069800 Sapur.001G072500 Sapur.001G075700 Sapur.001G090900 Sapur.001G108900 Sapur.001G119600 Sapur.001G129000 Sapur.001G130000 Sapur.001G163400 Sapur.001G164600 Sapur.001G179800 Sapur.002G012900 Sapur.002G023100 Sapur.002G024200 Sapur.002G028500 Sapur.002G030000 Sapur.002G030100 Sapur.002G033200 Sapur.002G043300 Sapur.002G064600 Sapur.002G069500 Sapur.002G073000 Sapur.002G080700 Sapur.002G091700 Sapur.002G110600 Sapur.002G112100 Sapur.002G115400 Sapur.002G125900 Sapur.002G126000 Sapur.002G136900 Sapur.002G142300 Sapur.002G143500 Sapur.002G146000 Sapur.002G151100 Sapur.002G163600 Sapur.002G175800 Sapur.002G190900 Sapur.003G017600 Sapur.003G026500 Sapur.003G047900 Sapur.003G049200 Sapur.003G057500 Sapur.003G067100 Sapur.003G070500 Sapur.003G091600 Sapur.003G092400 Sapur.003G099900 Sapur.003G102000 Sapur.003G111600 Sapur.003G123800 Sapur.003G125900 Sapur.003G138200 Sapur.003G145700 Sapur.003G150800 Sapur.003G152800 Sapur.004G007000 Sapur.004G008500 Sapur.004G030500 Sapur.004G030800 Sapur.004G031600 Sapur.004G054800 Sapur.004G056900 Sapur.004G118800 Sapur.004G121500 Sapur.004G123100 Sapur.004G131600 Sapur.004G137000 Sapur.004G171400 Sapur.005G042000 Sapur.005G052900 Sapur.005G054800 Sapur.005G055100 Sapur.005G067000 Sapur.005G087400 Sapur.005G098100 Sapur.005G105500 Sapur.005G108500 Sapur.005G133200 Sapur.005G141300 Sapur.005G150000 Sapur.005G163900 Sapur.006G041100 Sapur.006G043000 Sapur.006G063200 Sapur.006G067500 Sapur.006G068600 Sapur.006G070000

Sapur.006G083400	Sapur.006G089400	Sapur.006G103400	Sapur.006G104700	Sapur.006G110100	Sapur.006G114200
Sapur.006G118700	Sapur.006G120000	Sapur.006G120300	Sapur.006G122700	Sapur.006G137200	Sapur.006G166000
Sapur.006G168000	Sapur.006G194100	Sapur.006G195200	Sapur.006G197300	Sapur.006G214400	Sapur.006G218000
Sapur.006G228700	Sapur.006G228900	Sapur.007G001300	Sapur.007G005500	Sapur.007G021900	Sapur.007G034000
Sapur.007G035800	Sapur.007G044600	Sapur.007G050900	Sapur.007G058700	Sapur.007G062200	Sapur.007G082500
Sapur.007G087500	Sapur.007G087800	Sapur.007G089800	Sapur.007G094000	Sapur.007G114800	Sapur.007G123400
Sapur.008G034200	Sapur.008G042700	Sapur.008G053100	Sapur.008G064800	Sapur.008G069200	Sapur.008G081900
Sapur.008G090400	Sapur.008G093800	Sapur.008G094300	Sapur.008G096700	Sapur.008G104300	Sapur.008G119700
Sapur.008G121600	Sapur.008G134800	Sapur.008G160400	Sapur.009G005300	Sapur.009G009700	Sapur.009G011600
Sapur.009G021700	Sapur.009G040000	Sapur.009G094800	Sapur.009G097900	Sapur.009G104900	Sapur.009G112800
Sapur.009G115000	Sapur.009G126000	Sapur.009G126600	Sapur.010G003100	Sapur.010G005200	Sapur.010G013000
Sapur.010G040000	Sapur.010G040100	Sapur.010G046500	Sapur.010G065900	Sapur.010G068700	Sapur.010G086900
Sapur.010G096300	Sapur.010G099700	Sapur.010G100200	Sapur.010G113700	Sapur.010G114700	Sapur.010G123400
Sapur.010G128300	Sapur.010G134600	Sapur.010G149100	Sapur.010G151500	Sapur.010G154300	Sapur.010G161100
Sapur.011G006600	Sapur.011G014000	Sapur.011G032100	Sapur.011G035500	Sapur.011G037900	Sapur.011G038200
Sapur.011G039300	Sapur.011G041100	Sapur.011G042400	Sapur.011G045800	Sapur.011G049300	Sapur.011G054600
Sapur.011G061100	Sapur.011G064300	Sapur.011G066500	Sapur.011G079300	Sapur.011G088400	Sapur.011G088500
Sapur.012G004000	Sapur.012G010400	Sapur.012G017700	Sapur.012G018600	Sapur.012G024500	Sapur.012G031400
Sapur.012G051400	Sapur.012G055400	Sapur.012G060100	Sapur.012G067900	Sapur.012G078700	Sapur.012G105100
Sapur.013G000400	Sapur.013G008000	Sapur.013G038800	Sapur.013G039500	Sapur.013G041800	Sapur.013G050700
Sapur.013G050900	Sapur.013G055500	Sapur.013G059000	Sapur.013G068800	Sapur.013G087600	Sapur.013G108500
Sapur.013G137400	Sapur.013G141800	Sapur.014G003700	Sapur.014G004000	Sapur.014G005200	Sapur.014G034500
Sapur.014G053000	Sapur.014G059000	Sapur.014G059100	Sapur.014G059200	Sapur.014G065300	Sapur.014G078300
Sapur.014G078500	Sapur.014G079300	Sapur.014G085400	Sapur.014G085500	Sapur.014G094500	Sapur.014G095700
Sapur.014G099500	Sapur.014G101000	Sapur.016G018700	Sapur.016G030200	Sapur.016G064600	Sapur.016G070400
Sapur.016G079900	Sapur.016G080200	Sapur.016G085100	Sapur.016G087700	Sapur.016G104000	Sapur.016G111900
Sapur.016G114700	Sapur.016G131300	Sapur.016G153200	Sapur.016G159000	Sapur.016G167800	Sapur.016G199700
Sapur.016G216900	Sapur.016G224700	Sapur.016G229800	Sapur.016G233500	Sapur.016G255700	Sapur.016G298800
Sapur.017G004800	Sapur.017G007100	Sapur.017G053300	Sapur.017G057100	Sapur.017G069900	Sapur.017G085900
Sapur.017G114800	Sapur.017G115700	Sapur.018G007100	Sapur.018G022500	Sapur.018G022700	Sapur.018G025000
Sapur.018G034800	Sapur.018G035300	Sapur.018G057700	Sapur.018G067800	Sapur.019G025000	Sapur.019G033200
Sapur.019G039900	Sapur.019G060100	Sapur.019G074500	Sapur.019G098000	Sapur.019G101700	Sapur.019G108100
Sapur.15WG060500	Sapur.15WG065100	Sapur.15ZG002000	Sapur.15ZG007300	Sapur.15ZG016900	Sapur.15ZG053200

				Sapur.15ZG067200 Sapur.15ZG071900 Sapur.15ZG079000 Sapur.15ZG080900 Sapur.15ZG085300 Sapur.15ZG103400 Sapur.T006300 Sapur.T012000 Sapur.003G056300 Sapur.002G029000 Sapur.001G057600 Sapur.003G116600 Sapur.008G063700 Sapur.010G137300 Sapur.014G098600
GO:0032774	288	1268	RNA biosynthetic process	Sapur.001G017300 Sapur.001G025400 Sapur.001G027900 Sapur.001G028100 Sapur.001G028400 Sapur.001G034200 Sapur.001G064300 Sapur.001G067700 Sapur.001G069800 Sapur.001G072500 Sapur.001G075700 Sapur.001G090900 Sapur.001G108900 Sapur.001G119600 Sapur.001G129000 Sapur.001G130000 Sapur.001G163400 Sapur.001G164600 Sapur.001G179800 Sapur.002G012900 Sapur.002G023100 Sapur.002G024200 Sapur.002G028500 Sapur.002G030000 Sapur.002G030100 Sapur.002G033200 Sapur.002G043300 Sapur.002G064600 Sapur.002G069500 Sapur.002G073000 Sapur.002G080700 Sapur.002G091700 Sapur.002G110600 Sapur.002G115400 Sapur.002G125900 Sapur.002G126000 Sapur.002G136900 Sapur.002G142300 Sapur.002G143500 Sapur.002G146000 Sapur.002G151100 Sapur.002G163600 Sapur.002G175800 Sapur.002G190900 Sapur.003G017600 Sapur.003G026500 Sapur.003G047900 Sapur.003G049200 Sapur.003G057500 Sapur.003G067100 Sapur.003G070500 Sapur.003G091600 Sapur.003G099900 Sapur.003G102000 Sapur.003G111600 Sapur.003G123800 Sapur.003G125900 Sapur.003G138200 Sapur.003G145700 Sapur.003G150800 Sapur.003G152800 Sapur.004G007000 Sapur.004G030500 Sapur.004G030800 Sapur.004G031600 Sapur.004G054800 Sapur.004G056900 Sapur.004G118800 Sapur.004G121500 Sapur.004G123100 Sapur.004G131600 Sapur.004G137000 Sapur.004G171400 Sapur.005G042000 Sapur.005G052900 Sapur.005G054800 Sapur.005G055100 Sapur.005G067000 Sapur.005G087400 Sapur.005G098100 Sapur.005G105500 Sapur.005G108500 Sapur.005G133200 Sapur.005G141300 Sapur.005G150000 Sapur.005G163900 Sapur.006G041100 Sapur.006G043000 Sapur.006G063200 Sapur.006G067500 Sapur.006G068600 Sapur.006G070000 Sapur.006G089400 Sapur.006G103400 Sapur.006G104700 Sapur.006G110100 Sapur.006G114200 Sapur.006G118700 Sapur.006G120000 Sapur.006G120300 Sapur.006G122700 Sapur.006G137200 Sapur.006G166000 Sapur.006G168000 Sapur.006G194100 Sapur.006G195200 Sapur.006G197300 Sapur.006G214400 Sapur.006G218000 Sapur.006G228700 Sapur.006G228900 Sapur.007G001300 Sapur.007G005500 Sapur.007G021900 Sapur.007G034000 Sapur.007G035800 Sapur.007G044600 Sapur.007G050900 Sapur.007G058700 Sapur.007G062200 Sapur.007G082500 Sapur.007G087500 Sapur.007G087800 Sapur.007G089800 Sapur.007G094000 Sapur.007G114800 Sapur.007G123400 Sapur.008G034200 Sapur.008G042700 Sapur.008G053100 Sapur.008G064800 Sapur.008G069200 Sapur.008G081900 Sapur.008G090400 Sapur.008G093800 Sapur.008G094300 Sapur.008G096700 Sapur.008G104300 Sapur.008G119700 Sapur.008G121600 Sapur.008G134800 Sapur.008G160400 Sapur.009G005300 Sapur.009G009700 Sapur.009G011600 Sapur.009G021700 Sapur.009G040000 Sapur.009G094800 Sapur.009G097900 Sapur.009G104900 Sapur.009G112800 Sapur.009G115000 Sapur.009G126000 Sapur.009G126600 Sapur.010G003100 Sapur.010G005200 Sapur.010G013000 Sapur.010G040000 Sapur.010G040100 Sapur.010G046500 Sapur.010G065900 Sapur.010G068700 Sapur.010G096300 Sapur.010G099700 Sapur.010G100200 Sapur.010G113700 Sapur.010G114700 Sapur.010G123400 Sapur.010G128300 Sapur.010G149100 Sapur.010G154300 Sapur.010G161100 Sapur.011G006600 Sapur.011G032100 Sapur.011G035500 Sapur.011G037900 Sapur.011G038200 Sapur.011G039300 Sapur.011G041100

				Sapur.011G042400 Sapur.011G045800 Sapur.011G049300 Sapur.011G054600 Sapur.011G061100 Sapur.011G066500 Sapur.011G079300 Sapur.011G088400 Sapur.011G088500 Sapur.012G004000 Sapur.012G010400 Sapur.012G017700 Sapur.012G018600 Sapur.012G024500 Sapur.012G031400 Sapur.012G051400 Sapur.012G060100 Sapur.012G067900 Sapur.012G078700 Sapur.012G105100 Sapur.013G000400 Sapur.013G008000 Sapur.013G038800 Sapur.013G039500 Sapur.013G041800 Sapur.013G050700 Sapur.013G050900 Sapur.013G055500 Sapur.013G059000 Sapur.013G068800 Sapur.013G087600 Sapur.013G137400 Sapur.013G141800 Sapur.014G003700 Sapur.014G004000 Sapur.014G005200 Sapur.014G034500 Sapur.014G053000 Sapur.014G059000 Sapur.014G059100 Sapur.014G059200 Sapur.014G065300 Sapur.014G078300 Sapur.014G078500 Sapur.014G079300 Sapur.014G085400 Sapur.014G085500 Sapur.014G094500 Sapur.014G095700 Sapur.014G099500 Sapur.014G101000 Sapur.016G018700 Sapur.016G030200 Sapur.016G064600 Sapur.016G070400 Sapur.016G079900 Sapur.016G080200 Sapur.016G085100 Sapur.016G087700 Sapur.016G111900 Sapur.016G114700 Sapur.016G131300 Sapur.016G153200 Sapur.016G159000 Sapur.016G167800 Sapur.016G199700 Sapur.016G216900 Sapur.016G224700 Sapur.016G229800 Sapur.016G233500 Sapur.016G255700 Sapur.016G298800 Sapur.017G004800 Sapur.017G007100 Sapur.017G053300 Sapur.017G057100 Sapur.017G069900 Sapur.017G085900 Sapur.017G114800 Sapur.017G115700 Sapur.018G007100 Sapur.018G022700 Sapur.018G025000 Sapur.018G034800 Sapur.018G035300 Sapur.018G057700 Sapur.018G067800 Sapur.019G025000 Sapur.019G033200 Sapur.019G039900 Sapur.019G060100 Sapur.019G074500 Sapur.019G098000 Sapur.019G101700 Sapur.019G108100 Sapur.15WG060500 Sapur.15WG065100 Sapur.15ZG002000 Sapur.15ZG007300 Sapur.15ZG016900 Sapur.15ZG053200 Sapur.15ZG067200 Sapur.15ZG071900 Sapur.15ZG079000 Sapur.15ZG080900 Sapur.15ZG103400 Sapur.T006300 Sapur.T012000 Sapur.002G029000
GO:0018130	314	1411	heterocycle biosynthetic process	Sapur.001G017300 Sapur.001G025400 Sapur.001G027900 Sapur.001G028100 Sapur.001G028400 Sapur.001G034200 Sapur.001G064300 Sapur.001G067700 Sapur.001G069800 Sapur.001G072500 Sapur.001G075700 Sapur.001G090900 Sapur.001G108900 Sapur.001G119600 Sapur.001G129000 Sapur.001G130000 Sapur.001G163400 Sapur.001G164600 Sapur.001G179800 Sapur.001G183500 Sapur.002G012900 Sapur.002G023100 Sapur.002G024200 Sapur.002G028500 Sapur.002G030000 Sapur.002G030100 Sapur.002G033200 Sapur.002G043300 Sapur.002G064600 Sapur.002G069500 Sapur.002G073000 Sapur.002G080700 Sapur.002G091700 Sapur.002G110600 Sapur.002G112100 Sapur.002G115400 Sapur.002G125900 Sapur.002G126000 Sapur.002G128100 Sapur.002G136900 Sapur.002G142300 Sapur.002G143500 Sapur.002G146000 Sapur.002G151100 Sapur.002G163600 Sapur.002G175800 Sapur.002G190900 Sapur.003G017600 Sapur.003G026500 Sapur.003G047900 Sapur.003G049200 Sapur.003G057500 Sapur.003G067100 Sapur.003G070500 Sapur.003G091600 Sapur.003G092400 Sapur.003G099900 Sapur.003G102000 Sapur.003G111600 Sapur.003G123800 Sapur.003G125900 Sapur.003G138200 Sapur.003G145700 Sapur.003G150800 Sapur.003G152800 Sapur.004G007000 Sapur.004G008500 Sapur.004G030500 Sapur.004G030800 Sapur.004G031600 Sapur.004G054800 Sapur.004G056900 Sapur.004G118800 Sapur.004G121500 Sapur.004G123100 Sapur.004G131600 Sapur.004G137000 Sapur.004G171400 Sapur.005G042000 Sapur.005G052900 Sapur.005G054800 Sapur.005G055100 Sapur.005G067000

Sapur.005G087400 Sapur.005G098100 Sapur.005G105500 Sapur.005G108500 Sapur.005G133200 Sapur.005G141300
Sapur.005G150000 Sapur.005G163900 Sapur.006G041100 Sapur.006G043000 Sapur.006G063200 Sapur.006G067500
Sapur.006G068600 Sapur.006G070000 Sapur.006G083400 Sapur.006G089400 Sapur.006G103400 Sapur.006G104700
Sapur.006G110100 Sapur.006G114200 Sapur.006G118700 Sapur.006G120000 Sapur.006G120300 Sapur.006G122700
Sapur.006G137200 Sapur.006G166000 Sapur.006G168000 Sapur.006G194100 Sapur.006G195200 Sapur.006G197300
Sapur.006G214400 Sapur.006G218000 Sapur.006G228700 Sapur.006G228900 Sapur.007G001300 Sapur.007G005500
Sapur.007G021900 Sapur.007G034000 Sapur.007G035800 Sapur.007G044600 Sapur.007G050900 Sapur.007G058700
Sapur.007G062200 Sapur.007G082500 Sapur.007G087500 Sapur.007G087800 Sapur.007G089800 Sapur.007G094000
Sapur.007G114800 Sapur.007G123400 Sapur.008G034200 Sapur.008G042700 Sapur.008G053100 Sapur.008G064800
Sapur.008G069200 Sapur.008G081900 Sapur.008G090400 Sapur.008G093800 Sapur.008G094300 Sapur.008G096700
Sapur.008G104300 Sapur.008G119700 Sapur.008G121600 Sapur.008G134800 Sapur.008G160400 Sapur.009G005300
Sapur.009G009700 Sapur.009G011600 Sapur.009G021700 Sapur.009G040000 Sapur.009G094800 Sapur.009G097900
Sapur.009G104900 Sapur.009G112800 Sapur.009G115000 Sapur.009G126000 Sapur.009G126600 Sapur.010G003100
Sapur.010G005200 Sapur.010G013000 Sapur.010G040000 Sapur.010G040100 Sapur.010G046500 Sapur.010G065900
Sapur.010G068700 Sapur.010G086900 Sapur.010G096300 Sapur.010G099700 Sapur.010G100200 Sapur.010G113700
Sapur.010G114700 Sapur.010G123400 Sapur.010G128300 Sapur.010G134600 Sapur.010G149100 Sapur.010G151500
Sapur.010G154300 Sapur.010G161100 Sapur.011G006600 Sapur.011G014000 Sapur.011G032100 Sapur.011G035500
Sapur.011G037900 Sapur.011G038200 Sapur.011G039300 Sapur.011G041100 Sapur.011G042400 Sapur.011G045800
Sapur.011G049300 Sapur.011G054600 Sapur.011G061100 Sapur.011G064300 Sapur.011G066500 Sapur.011G079300
Sapur.011G088400 Sapur.011G088500 Sapur.012G004000 Sapur.012G010400 Sapur.012G017700 Sapur.012G018600
Sapur.012G024500 Sapur.012G031400 Sapur.012G051400 Sapur.012G055400 Sapur.012G060100 Sapur.012G067900
Sapur.012G078700 Sapur.012G084400 Sapur.012G105100 Sapur.013G000400 Sapur.013G008000 Sapur.013G038800
Sapur.013G039500 Sapur.013G041800 Sapur.013G050700 Sapur.013G050900 Sapur.013G055500 Sapur.013G059000
Sapur.013G068800 Sapur.013G087600 Sapur.013G108500 Sapur.013G137400 Sapur.013G141800 Sapur.014G003700
Sapur.014G004000 Sapur.014G005200 Sapur.014G034500 Sapur.014G053000 Sapur.014G059000 Sapur.014G059100
Sapur.014G059200 Sapur.014G065300 Sapur.014G078300 Sapur.014G078500 Sapur.014G079300 Sapur.014G085400
Sapur.014G085500 Sapur.014G094500 Sapur.014G095700 Sapur.014G099500 Sapur.014G101000 Sapur.016G018700
Sapur.016G030200 Sapur.016G064600 Sapur.016G070400 Sapur.016G079900 Sapur.016G080200 Sapur.016G085100
Sapur.016G087700 Sapur.016G101200 Sapur.016G104000 Sapur.016G111900 Sapur.016G114700 Sapur.016G131300
Sapur.016G153200 Sapur.016G159000 Sapur.016G167800 Sapur.016G199700 Sapur.016G216900 Sapur.016G224700
Sapur.016G229800 Sapur.016G233500 Sapur.016G255700 Sapur.016G298800 Sapur.017G004800 Sapur.017G007100
Sapur.017G053300 Sapur.017G057100 Sapur.017G069900 Sapur.017G085900 Sapur.017G106300 Sapur.017G114800
Sapur.017G115700 Sapur.018G007100 Sapur.018G022500 Sapur.018G022700 Sapur.018G025000 Sapur.018G034800

				Sapur.018G035300 Sapur.018G057700 Sapur.018G067800 Sapur.019G025000 Sapur.019G033200 Sapur.019G039900 Sapur.019G060100 Sapur.019G074500 Sapur.019G098000 Sapur.019G101700 Sapur.019G108100 Sapur.15WG060500 Sapur.15WG063000 Sapur.15WG065100 Sapur.15ZG002000 Sapur.15ZG007300 Sapur.15ZG016900 Sapur.15ZG053200 Sapur.15ZG067200 Sapur.15ZG071900 Sapur.15ZG079000 Sapur.15ZG080900 Sapur.15ZG085300 Sapur.15ZG103400 Sapur.T006300 Sapur.T012000 Sapur.003G056300 Sapur.002G029000 Sapur.001G057600 Sapur.003G116600 Sapur.008G063700 Sapur.010G137300 Sapur.014G098600
GO:0006351	287	1266	DNA-templated transcription	Sapur.001G017300 Sapur.001G025400 Sapur.001G027900 Sapur.001G028100 Sapur.001G028400 Sapur.001G034200 Sapur.001G064300 Sapur.001G067700 Sapur.001G069800 Sapur.001G072500 Sapur.001G075700 Sapur.001G090900 Sapur.001G108900 Sapur.001G119600 Sapur.001G129000 Sapur.001G130000 Sapur.001G163400 Sapur.001G164600 Sapur.001G179800 Sapur.002G012900 Sapur.002G023100 Sapur.002G024200 Sapur.002G028500 Sapur.002G030000 Sapur.002G030100 Sapur.002G033200 Sapur.002G043300 Sapur.002G064600 Sapur.002G069500 Sapur.002G073000 Sapur.002G080700 Sapur.002G091700 Sapur.002G110600 Sapur.002G115400 Sapur.002G125900 Sapur.002G126000 Sapur.002G136900 Sapur.002G142300 Sapur.002G143500 Sapur.002G146000 Sapur.002G151100 Sapur.002G163600 Sapur.002G175800 Sapur.002G190900 Sapur.003G017600 Sapur.003G026500 Sapur.003G047900 Sapur.003G049200 Sapur.003G057500 Sapur.003G067100 Sapur.003G091600 Sapur.003G099900 Sapur.003G102000 Sapur.003G111600 Sapur.003G123800 Sapur.003G125900 Sapur.003G138200 Sapur.003G145700 Sapur.003G150800 Sapur.003G152800 Sapur.004G007000 Sapur.004G030500 Sapur.004G030800 Sapur.004G031600 Sapur.004G054800 Sapur.004G056900 Sapur.004G118800 Sapur.004G121500 Sapur.004G123100 Sapur.004G131600 Sapur.004G137000 Sapur.004G171400 Sapur.005G042000 Sapur.005G052900 Sapur.005G054800 Sapur.005G055100 Sapur.005G067000 Sapur.005G087400 Sapur.005G098100 Sapur.005G105500 Sapur.005G108500 Sapur.005G133200 Sapur.005G141300 Sapur.005G150000 Sapur.005G163900 Sapur.006G041100 Sapur.006G043000 Sapur.006G063200 Sapur.006G067500 Sapur.006G068600 Sapur.006G070000 Sapur.006G089400 Sapur.006G103400 Sapur.006G104700 Sapur.006G110100 Sapur.006G114200 Sapur.006G118700 Sapur.006G120000 Sapur.006G120300 Sapur.006G122700 Sapur.006G137200 Sapur.006G166000 Sapur.006G168000 Sapur.006G194100 Sapur.006G195200 Sapur.006G197300 Sapur.006G214400 Sapur.006G218000 Sapur.006G228700 Sapur.006G228900 Sapur.007G001300 Sapur.007G005500 Sapur.007G021900 Sapur.007G034000 Sapur.007G035800 Sapur.007G044600 Sapur.007G050900 Sapur.007G058700 Sapur.007G062200 Sapur.007G082500 Sapur.007G087500 Sapur.007G087800 Sapur.007G089800 Sapur.007G094000 Sapur.007G114800 Sapur.007G123400 Sapur.008G034200 Sapur.008G042700 Sapur.008G053100 Sapur.008G064800 Sapur.008G069200 Sapur.008G081900 Sapur.008G090400 Sapur.008G093800 Sapur.008G094300 Sapur.008G096700 Sapur.008G104300 Sapur.008G119700 Sapur.008G121600 Sapur.008G134800 Sapur.008G160400 Sapur.009G005300 Sapur.009G009700 Sapur.009G011600 Sapur.009G021700 Sapur.009G040000 Sapur.009G094800 Sapur.009G097900 Sapur.009G104900 Sapur.009G112800 Sapur.009G115000 Sapur.009G126000 Sapur.009G126600 Sapur.010G003100 Sapur.010G005200 Sapur.010G013000 Sapur.010G040000 Sapur.010G040100 Sapur.010G046500 Sapur.010G065900 Sapur.010G068700

				Sapur.010G096300 Sapur.010G099700 Sapur.010G100200 Sapur.010G113700 Sapur.010G114700 Sapur.010G123400 Sapur.010G128300 Sapur.010G149100 Sapur.010G154300 Sapur.010G161100 Sapur.011G006600 Sapur.011G032100 Sapur.011G035500 Sapur.011G037900 Sapur.011G038200 Sapur.011G039300 Sapur.011G041100 Sapur.011G042400 Sapur.011G045800 Sapur.011G049300 Sapur.011G054600 Sapur.011G061100 Sapur.011G066500 Sapur.011G079300 Sapur.011G088400 Sapur.011G088500 Sapur.012G004000 Sapur.012G010400 Sapur.012G017700 Sapur.012G018600 Sapur.012G024500 Sapur.012G031400 Sapur.012G051400 Sapur.012G060100 Sapur.012G067900 Sapur.012G078700 Sapur.012G105100 Sapur.013G000400 Sapur.013G008000 Sapur.013G038800 Sapur.013G039500 Sapur.013G041800 Sapur.013G050700 Sapur.013G050900 Sapur.013G055500 Sapur.013G059000 Sapur.013G068800 Sapur.013G087600 Sapur.013G137400 Sapur.013G141800 Sapur.014G003700 Sapur.014G004000 Sapur.014G005200 Sapur.014G034500 Sapur.014G053000 Sapur.014G059000 Sapur.014G059100 Sapur.014G059200 Sapur.014G065300 Sapur.014G078300 Sapur.014G078500 Sapur.014G079300 Sapur.014G085400 Sapur.014G085500 Sapur.014G094500 Sapur.014G095700 Sapur.014G099500 Sapur.014G101000 Sapur.016G018700 Sapur.016G030200 Sapur.016G064600 Sapur.016G070400 Sapur.016G079900 Sapur.016G080200 Sapur.016G085100 Sapur.016G087700 Sapur.016G111900 Sapur.016G114700 Sapur.016G131300 Sapur.016G153200 Sapur.016G159000 Sapur.016G167800 Sapur.016G199700 Sapur.016G216900 Sapur.016G224700 Sapur.016G229800 Sapur.016G233500 Sapur.016G255700 Sapur.016G298800 Sapur.017G004800 Sapur.017G007100 Sapur.017G053300 Sapur.017G057100 Sapur.017G069900 Sapur.017G085900 Sapur.017G114800 Sapur.017G115700 Sapur.018G007100 Sapur.018G022700 Sapur.018G025000 Sapur.018G034800 Sapur.018G035300 Sapur.018G057700 Sapur.018G067800 Sapur.019G025000 Sapur.019G033200 Sapur.019G039900 Sapur.019G060100 Sapur.019G074500 Sapur.019G098000 Sapur.019G101700 Sapur.019G108100 Sapur.15WG060500 Sapur.15WG065100 Sapur.15ZG002000 Sapur.15ZG007300 Sapur.15ZG016900 Sapur.15ZG053200 Sapur.15ZG067200 Sapur.15ZG071900 Sapur.15ZG079000 Sapur.15ZG080900 Sapur.15ZG103400 Sapur.T006300 Sapur.T012000 Sapur.002G029000
GO:1901362	318	1454	organic cyclic compound biosynthetic process	Sapur.001G017300 Sapur.001G025400 Sapur.001G027900 Sapur.001G028100 Sapur.001G028400 Sapur.001G034200 Sapur.001G064300 Sapur.001G067700 Sapur.001G069800 Sapur.001G072500 Sapur.001G075700 Sapur.001G090900 Sapur.001G108900 Sapur.001G119600 Sapur.001G129000 Sapur.001G130000 Sapur.001G163400 Sapur.001G164600 Sapur.001G179800 Sapur.001G183500 Sapur.002G012900 Sapur.002G023100 Sapur.002G024200 Sapur.002G028500 Sapur.002G030000 Sapur.002G030100 Sapur.002G033200 Sapur.002G043300 Sapur.002G064600 Sapur.002G069500 Sapur.002G073000 Sapur.002G080700 Sapur.002G091700 Sapur.002G110600 Sapur.002G112100 Sapur.002G115400 Sapur.002G125900 Sapur.002G126000 Sapur.002G128100 Sapur.002G136900 Sapur.002G142300 Sapur.002G143500 Sapur.002G146000 Sapur.002G151100 Sapur.002G163600 Sapur.002G175800 Sapur.002G190900 Sapur.003G017600 Sapur.003G026500 Sapur.003G047900 Sapur.003G049200 Sapur.003G057500 Sapur.003G060900 Sapur.003G067100 Sapur.003G070500 Sapur.003G091600 Sapur.003G092400 Sapur.003G099900 Sapur.003G102000 Sapur.003G111600 Sapur.003G123800 Sapur.003G125900 Sapur.003G138200 Sapur.003G145700 Sapur.003G150800 Sapur.003G152800 Sapur.004G007000 Sapur.004G008500 Sapur.004G030500 Sapur.004G030800 Sapur.004G031600

	Sapur.004G054800	Sapur.004G056900	Sapur.004G118800	Sapur.004G121500	Sapur.004G123100	Sapur.004G131600
	Sapur.004G137000	Sapur.004G171400	Sapur.005G042000	Sapur.005G052900	Sapur.005G054800	Sapur.005G055100
	Sapur.005G067000	Sapur.005G087400	Sapur.005G098100	Sapur.005G105500	Sapur.005G108500	Sapur.005G133200
	Sapur.005G141300	Sapur.005G150000	Sapur.005G163900	Sapur.006G041100	Sapur.006G043000	Sapur.006G049700
	Sapur.006G063200	Sapur.006G067500	Sapur.006G068600	Sapur.006G070000	Sapur.006G083400	Sapur.006G089400
	Sapur.006G103400	Sapur.006G104700	Sapur.006G110100	Sapur.006G114200	Sapur.006G118700	Sapur.006G120000
	Sapur.006G120300	Sapur.006G122700	Sapur.006G137200	Sapur.006G166000	Sapur.006G168000	Sapur.006G194100
	Sapur.006G195200	Sapur.006G197300	Sapur.006G214400	Sapur.006G218000	Sapur.006G228700	Sapur.006G228900
	Sapur.007G001300	Sapur.007G005500	Sapur.007G021900	Sapur.007G034000	Sapur.007G035800	Sapur.007G044600
	Sapur.007G050900	Sapur.007G058700	Sapur.007G062200	Sapur.007G082500	Sapur.007G087500	Sapur.007G087800
	Sapur.007G089800	Sapur.007G094000	Sapur.007G114800	Sapur.007G123400	Sapur.008G034200	Sapur.008G042700
	Sapur.008G053100	Sapur.008G064800	Sapur.008G069200	Sapur.008G081900	Sapur.008G090400	Sapur.008G093800
	Sapur.008G094300	Sapur.008G096700	Sapur.008G104300	Sapur.008G119700	Sapur.008G121600	Sapur.008G134800
	Sapur.008G160400	Sapur.009G005300	Sapur.009G009700	Sapur.009G011600	Sapur.009G021700	Sapur.009G040000
	Sapur.009G094800	Sapur.009G097900	Sapur.009G104900	Sapur.009G112800	Sapur.009G115000	Sapur.009G126000
	Sapur.009G126600	Sapur.010G003100	Sapur.010G005200	Sapur.010G013000	Sapur.010G040000	Sapur.010G040100
	Sapur.010G046500	Sapur.010G065900	Sapur.010G068700	Sapur.010G086900	Sapur.010G096300	Sapur.010G099700
	Sapur.010G100200	Sapur.010G113700	Sapur.010G114700	Sapur.010G123400	Sapur.010G128300	Sapur.010G134600
	Sapur.010G149100	Sapur.010G151500	Sapur.010G154300	Sapur.010G161100	Sapur.011G006600	Sapur.011G014000
	Sapur.011G032100	Sapur.011G035500	Sapur.011G037900	Sapur.011G038200	Sapur.011G039300	Sapur.011G041100
	Sapur.011G042400	Sapur.011G045800	Sapur.011G049300	Sapur.011G054600	Sapur.011G061100	Sapur.011G064300
	Sapur.011G066500	Sapur.011G079300	Sapur.011G088400	Sapur.011G088500	Sapur.012G004000	Sapur.012G010400
	Sapur.012G017700	Sapur.012G018600	Sapur.012G024500	Sapur.012G031400	Sapur.012G051400	Sapur.012G055400
	Sapur.012G060100	Sapur.012G067900	Sapur.012G078700	Sapur.012G084400	Sapur.012G105100	Sapur.013G000400
	Sapur.013G008000	Sapur.013G038800	Sapur.013G039500	Sapur.013G041800	Sapur.013G050700	Sapur.013G050900
	Sapur.013G055500	Sapur.013G059000	Sapur.013G068800	Sapur.013G087600	Sapur.013G108500	Sapur.013G137400
	Sapur.013G141800	Sapur.014G003700	Sapur.014G004000	Sapur.014G005200	Sapur.014G034500	Sapur.014G053000
	Sapur.014G059000	Sapur.014G059100	Sapur.014G059200	Sapur.014G065300	Sapur.014G078300	Sapur.014G078500
	Sapur.014G079300	Sapur.014G085400	Sapur.014G085500	Sapur.014G094500	Sapur.014G095700	Sapur.014G099500
	Sapur.014G101000	Sapur.016G016700	Sapur.016G018700	Sapur.016G030200	Sapur.016G064600	Sapur.016G070400
	Sapur.016G079900	Sapur.016G080200	Sapur.016G085100	Sapur.016G087700	Sapur.016G101200	Sapur.016G104000
	Sapur.016G111900	Sapur.016G114700	Sapur.016G131300	Sapur.016G153200	Sapur.016G159000	Sapur.016G167800
	Sapur.016G199700	Sapur.016G216900	Sapur.016G224700	Sapur.016G229800	Sapur.016G233500	Sapur.016G255700

				Sapur.016G298800 Sapur.017G004800 Sapur.017G007100 Sapur.017G053300 Sapur.017G057100 Sapur.017G069900 Sapur.017G085900 Sapur.017G089700 Sapur.017G106300 Sapur.017G114800 Sapur.017G115700 Sapur.018G007100 Sapur.018G022500 Sapur.018G022700 Sapur.018G025000 Sapur.018G034800 Sapur.018G035300 Sapur.018G057700 Sapur.018G067800 Sapur.019G025000 Sapur.019G033200 Sapur.019G039900 Sapur.019G060100 Sapur.019G074500 Sapur.019G098000 Sapur.019G101700 Sapur.019G108100 Sapur.15WG060500 Sapur.15WG063000 Sapur.15WG065100 Sapur.15ZG002000 Sapur.15ZG007300 Sapur.15ZG016900 Sapur.15ZG053200 Sapur.15ZG067200 Sapur.15ZG071900 Sapur.15ZG079000 Sapur.15ZG080900 Sapur.15ZG085300 Sapur.15ZG103400 Sapur.T006300 Sapur.T012000 Sapur.003G056300 Sapur.002G029000 Sapur.001G057600 Sapur.003G116600 Sapur.008G063700 Sapur.010G137300 Sapur.014G098600
GO:0006139	432	2108	nucleobase-containing compound metabolic process	Sapur.007G110800 Sapur.013G054700 Sapur.017G027400 Sapur.001G017300 Sapur.001G025400 Sapur.001G027900 Sapur.001G028100 Sapur.001G028400 Sapur.001G034200 Sapur.001G055600 Sapur.001G058800 Sapur.001G064300 Sapur.001G067700 Sapur.001G069800 Sapur.001G072500 Sapur.001G075700 Sapur.001G090900 Sapur.001G108900 Sapur.001G119600 Sapur.001G129000 Sapur.001G130000 Sapur.001G149200 Sapur.001G163400 Sapur.001G163900 Sapur.001G164600 Sapur.001G167800 Sapur.001G179800 Sapur.001G183500 Sapur.001G183600 Sapur.002G012900 Sapur.002G023100 Sapur.002G024200 Sapur.002G028500 Sapur.002G030000 Sapur.002G030100 Sapur.002G033200 Sapur.002G043300 Sapur.002G048900 Sapur.002G064600 Sapur.002G069500 Sapur.002G073000 Sapur.002G080700 Sapur.002G091700 Sapur.002G110600 Sapur.002G112100 Sapur.002G115400 Sapur.002G125900 Sapur.002G126000 Sapur.002G136900 Sapur.002G142300 Sapur.002G143500 Sapur.002G146000 Sapur.002G151100 Sapur.002G161200 Sapur.002G163600 Sapur.002G175800 Sapur.002G190900 Sapur.003G016800 Sapur.003G017600 Sapur.003G026500 Sapur.003G047900 Sapur.003G049200 Sapur.003G054700 Sapur.003G057500 Sapur.003G067100 Sapur.003G068400 Sapur.003G070500 Sapur.003G091600 Sapur.003G092400 Sapur.003G099900 Sapur.003G102000 Sapur.003G111600 Sapur.003G123800 Sapur.003G125900 Sapur.003G138200 Sapur.003G145700 Sapur.003G150800 Sapur.003G152800 Sapur.003G155000 Sapur.004G007000 Sapur.004G008500 Sapur.004G027800 Sapur.004G030500 Sapur.004G030800 Sapur.004G031600 Sapur.004G054800 Sapur.004G056900 Sapur.004G102300 Sapur.004G103000 Sapur.004G118800 Sapur.004G121500 Sapur.004G123100 Sapur.004G131600 Sapur.004G137000 Sapur.004G171400 Sapur.005G005600 Sapur.005G042000 Sapur.005G045400 Sapur.005G046200 Sapur.005G048400 Sapur.005G050200 Sapur.005G052900 Sapur.005G054800 Sapur.005G055100 Sapur.005G067000 Sapur.005G069000 Sapur.005G087400 Sapur.005G098100 Sapur.005G105500 Sapur.005G108500 Sapur.005G119200 Sapur.005G133200 Sapur.005G141300 Sapur.005G150000 Sapur.005G163900 Sapur.006G025100 Sapur.006G041100 Sapur.006G043000 Sapur.006G063200 Sapur.006G067500 Sapur.006G068600 Sapur.006G070000 Sapur.006G083400 Sapur.006G089400 Sapur.006G103400 Sapur.006G104700 Sapur.006G110100 Sapur.006G114200 Sapur.006G118700 Sapur.006G120000 Sapur.006G120300 Sapur.006G121100 Sapur.006G122700 Sapur.006G137200 Sapur.006G154200 Sapur.006G166000 Sapur.006G168000 Sapur.006G194100 Sapur.006G195200 Sapur.006G197300 Sapur.006G214400 Sapur.006G218000 Sapur.006G228700

Sapur.006G228900 Sapur.007G001300 Sapur.007G002900 Sapur.007G005500 Sapur.007G021900 Sapur.007G034000
Sapur.007G035800 Sapur.007G044500 Sapur.007G044600 Sapur.007G050900 Sapur.007G058700 Sapur.007G062200
Sapur.007G071200 Sapur.007G082500 Sapur.007G087500 Sapur.007G087800 Sapur.007G089800 Sapur.007G094000
Sapur.007G114800 Sapur.007G115600 Sapur.007G123400 Sapur.008G034200 Sapur.008G042700 Sapur.008G044200
Sapur.008G053100 Sapur.008G060600 Sapur.008G064800 Sapur.008G069200 Sapur.008G081900 Sapur.008G090400
Sapur.008G093800 Sapur.008G094300 Sapur.008G096700 Sapur.008G098100 Sapur.008G102800 Sapur.008G104300
Sapur.008G119700 Sapur.008G121600 Sapur.008G128300 Sapur.008G134800 Sapur.008G160400 Sapur.008G162200
Sapur.008G168800 Sapur.009G002400 Sapur.009G002900 Sapur.009G005300 Sapur.009G009700 Sapur.009G011600
Sapur.009G021700 Sapur.009G040000 Sapur.009G045200 Sapur.009G094800 Sapur.009G097900 Sapur.009G099800
Sapur.009G104900 Sapur.009G112500 Sapur.009G112800 Sapur.009G115000 Sapur.009G126000 Sapur.009G126600
Sapur.010G003100 Sapur.010G005200 Sapur.010G011800 Sapur.010G013000 Sapur.010G040000 Sapur.010G040100
Sapur.010G046500 Sapur.010G046700 Sapur.010G053800 Sapur.010G065900 Sapur.010G068700 Sapur.010G086900
Sapur.010G087900 Sapur.010G093900 Sapur.010G096300 Sapur.010G099700 Sapur.010G100200 Sapur.010G113700
Sapur.010G114700 Sapur.010G123400 Sapur.010G128300 Sapur.010G134600 Sapur.010G136100 Sapur.010G141100
Sapur.010G149100 Sapur.010G151500 Sapur.010G154300 Sapur.010G161100 Sapur.010G169600 Sapur.010G193700
Sapur.011G006600 Sapur.011G008400 Sapur.011G014000 Sapur.011G032100 Sapur.011G035500 Sapur.011G037900
Sapur.011G038200 Sapur.011G039300 Sapur.011G041100 Sapur.011G042400 Sapur.011G045800 Sapur.011G049300
Sapur.011G054600 Sapur.011G056100 Sapur.011G061100 Sapur.011G064300 Sapur.011G066500 Sapur.011G079300
Sapur.011G088400 Sapur.011G088500 Sapur.011G101200 Sapur.012G000700 Sapur.012G004000 Sapur.012G010400
Sapur.012G017700 Sapur.012G018600 Sapur.012G020900 Sapur.012G024200 Sapur.012G024500 Sapur.012G031400
Sapur.012G051400 Sapur.012G055400 Sapur.012G060100 Sapur.012G067900 Sapur.012G078700 Sapur.012G105100
Sapur.013G000400 Sapur.013G008000 Sapur.013G010700 Sapur.013G038800 Sapur.013G039500 Sapur.013G041800
Sapur.013G050700 Sapur.013G050900 Sapur.013G055500 Sapur.013G059000 Sapur.013G067900 Sapur.013G068800
Sapur.013G082200 Sapur.013G087600 Sapur.013G103900 Sapur.013G108500 Sapur.013G126600 Sapur.013G128900
Sapur.013G135300 Sapur.013G137400 Sapur.013G138200 Sapur.013G141800 Sapur.014G003700 Sapur.014G004000
Sapur.014G005200 Sapur.014G034500 Sapur.014G053000 Sapur.014G059000 Sapur.014G059100 Sapur.014G059200
Sapur.014G065300 Sapur.014G066300 Sapur.014G078300 Sapur.014G078500 Sapur.014G079300 Sapur.014G085400
Sapur.014G085500 Sapur.014G094500 Sapur.014G095700 Sapur.014G099500 Sapur.014G101000 Sapur.014G104700
Sapur.014G106600 Sapur.014G112900 Sapur.014G117700 Sapur.014G138900 Sapur.014G142200 Sapur.016G018700
Sapur.016G030200 Sapur.016G039500 Sapur.016G058600 Sapur.016G064600 Sapur.016G070400 Sapur.016G079900
Sapur.016G080200 Sapur.016G085100 Sapur.016G087700 Sapur.016G104000 Sapur.016G111900 Sapur.016G114700
Sapur.016G131300 Sapur.016G151000 Sapur.016G152700 Sapur.016G153200 Sapur.016G159000 Sapur.016G167800
Sapur.016G195300 Sapur.016G199700 Sapur.016G205800 Sapur.016G216900 Sapur.016G224700 Sapur.016G225800

				Sapur.016G229800 Sapur.016G233500 Sapur.016G255700 Sapur.016G298800 Sapur.016G310400 Sapur.017G004800 Sapur.017G007100 Sapur.017G053300 Sapur.017G057100 Sapur.017G069900 Sapur.017G085900 Sapur.017G114800 Sapur.017G115700 Sapur.017G119700 Sapur.018G007100 Sapur.018G022500 Sapur.018G022700 Sapur.018G025000 Sapur.018G034800 Sapur.018G035300 Sapur.018G045400 Sapur.018G047900 Sapur.018G057700 Sapur.018G064600 Sapur.018G067800 Sapur.018G087600 Sapur.018G090600 Sapur.018G114600 Sapur.018G115000 Sapur.019G025000 Sapur.019G033200 Sapur.019G039400 Sapur.019G039900 Sapur.019G060100 Sapur.019G074500 Sapur.019G075900 Sapur.019G098000 Sapur.019G101700 Sapur.019G108100 Sapur.15WG032400 Sapur.15WG060500 Sapur.15WG065100 Sapur.15WG072800 Sapur.15WG074800 Sapur.15ZG002000 Sapur.15ZG007300 Sapur.15ZG016900 Sapur.15ZG020500 Sapur.15ZG036200 Sapur.15ZG043500 Sapur.15ZG053200 Sapur.15ZG067200 Sapur.15ZG071900 Sapur.15ZG079000 Sapur.15ZG080300 Sapur.15ZG080900 Sapur.15ZG085300 Sapur.15ZG103400 Sapur.T006300 Sapur.T012000<br>Sapur.003G056300 Sapur.001G151600 Sapur.002G029000 Sapur.004G156500 Sapur.006G022000 Sapur.016G026400 Sapur.018G088200 Sapur.15ZG007500 Sapur.15ZG120200 Sapur.T047900 Sapur.002G047600 Sapur.004G123200<br>Sapur.008G019800 Sapur.009G099900 Sapur.013G094800 Sapur.013G094900 Sapur.013G095000 Sapur.013G095100 Sapur.001G057600 Sapur.002G194100 Sapur.003G002000 Sapur.003G116600 Sapur.003G168000 Sapur.006G136200 Sapur.007G112200 Sapur.008G063700 Sapur.010G009500 Sapur.010G137300 Sapur.010G163200 Sapur.014G098600 Sapur.017G020600<br>Sapur.007G110800 Sapur.013G054700 Sapur.017G027400 Sapur.001G017300 Sapur.001G025400
GO:0006725	440	2155	cellular aromatic compound metabolic process	Sapur.001G027900 Sapur.001G028100 Sapur.001G028400 Sapur.001G034200 Sapur.001G055600 Sapur.001G058800 Sapur.001G064300 Sapur.001G067700 Sapur.001G069800 Sapur.001G072500 Sapur.001G075700 Sapur.001G090900 Sapur.001G108900 Sapur.001G119600 Sapur.001G129000 Sapur.001G130000 Sapur.001G149200 Sapur.001G163400 Sapur.001G163900 Sapur.001G164600 Sapur.001G167800 Sapur.001G179800 Sapur.001G183500 Sapur.001G183600 Sapur.002G012900 Sapur.002G023100 Sapur.002G024200 Sapur.002G028500 Sapur.002G030000 Sapur.002G030100 Sapur.002G033200 Sapur.002G043300 Sapur.002G048900 Sapur.002G064600 Sapur.002G069500 Sapur.002G073000 Sapur.002G080700 Sapur.002G091700 Sapur.002G110600 Sapur.002G112100 Sapur.002G115400 Sapur.002G125900 Sapur.002G126000 Sapur.002G128100 Sapur.002G136900 Sapur.002G142300 Sapur.002G143500 Sapur.002G146000 Sapur.002G151100 Sapur.002G161200 Sapur.002G163600 Sapur.002G175800 Sapur.002G190900 Sapur.003G016800 Sapur.003G017600 Sapur.003G026500 Sapur.003G047900 Sapur.003G049200 Sapur.003G054700 Sapur.003G057500 Sapur.003G067100 Sapur.003G068400 Sapur.003G070500 Sapur.003G091600 Sapur.003G092400 Sapur.003G099900 Sapur.003G102000 Sapur.003G111600 Sapur.003G123800 Sapur.003G125900 Sapur.003G138200 Sapur.003G145700 Sapur.003G150800 Sapur.003G152800 Sapur.003G155000 Sapur.004G007000 Sapur.004G008500 Sapur.004G027800 Sapur.004G030500 Sapur.004G030800 Sapur.004G031600 Sapur.004G054800 Sapur.004G056900 Sapur.004G102300 Sapur.004G103000 Sapur.004G118800 Sapur.004G121500 Sapur.004G123100 Sapur.004G131600 Sapur.004G137000 Sapur.004G171400 Sapur.005G005600 Sapur.005G042000 Sapur.005G045400 Sapur.005G046200 Sapur.005G048400

Sapur.005G050200 Sapur.005G052900 Sapur.005G054800 Sapur.005G055100 Sapur.005G067000 Sapur.005G069000
Sapur.005G087400 Sapur.005G098100 Sapur.005G105500 Sapur.005G108500 Sapur.005G119200 Sapur.005G133200
Sapur.005G141300 Sapur.005G150000 Sapur.005G163900 Sapur.006G025100 Sapur.006G041100 Sapur.006G043000
Sapur.006G049700 Sapur.006G063200 Sapur.006G067500 Sapur.006G068600 Sapur.006G070000 Sapur.006G083400
Sapur.006G089400 Sapur.006G103400 Sapur.006G104700 Sapur.006G110100 Sapur.006G114200 Sapur.006G118700
Sapur.006G120000 Sapur.006G120300 Sapur.006G121100 Sapur.006G122700 Sapur.006G137200 Sapur.006G154200
Sapur.006G166000 Sapur.006G168000 Sapur.006G194100 Sapur.006G195200 Sapur.006G197300 Sapur.006G214400
Sapur.006G218000 Sapur.006G228700 Sapur.006G228900 Sapur.007G001300 Sapur.007G002900 Sapur.007G005500
Sapur.007G021900 Sapur.007G034000 Sapur.007G035800 Sapur.007G044500 Sapur.007G044600 Sapur.007G050900
Sapur.007G058700 Sapur.007G062200 Sapur.007G071200 Sapur.007G082500 Sapur.007G087500 Sapur.007G087800
Sapur.007G089800 Sapur.007G094000 Sapur.007G114800 Sapur.007G115600 Sapur.007G123400 Sapur.008G034200
Sapur.008G042700 Sapur.008G044200 Sapur.008G053100 Sapur.008G060600 Sapur.008G064800 Sapur.008G069200
Sapur.008G081900 Sapur.008G090400 Sapur.008G093800 Sapur.008G094300 Sapur.008G096700 Sapur.008G098100
Sapur.008G102800 Sapur.008G104300 Sapur.008G119700 Sapur.008G121600 Sapur.008G128300 Sapur.008G134800
Sapur.008G160400 Sapur.008G162200 Sapur.008G168800 Sapur.009G002400 Sapur.009G002900 Sapur.009G005300
Sapur.009G009700 Sapur.009G011600 Sapur.009G021700 Sapur.009G040000 Sapur.009G045200 Sapur.009G094800
Sapur.009G097900 Sapur.009G099800 Sapur.009G104900 Sapur.009G112500 Sapur.009G112800 Sapur.009G115000
Sapur.009G126000 Sapur.009G126600 Sapur.010G003100 Sapur.010G005200 Sapur.010G011800 Sapur.010G013000
Sapur.010G040000 Sapur.010G040100 Sapur.010G046500 Sapur.010G046700 Sapur.010G053800 Sapur.010G065900
Sapur.010G068700 Sapur.010G086900 Sapur.010G087900 Sapur.010G093900 Sapur.010G096300 Sapur.010G099700
Sapur.010G100200 Sapur.010G113700 Sapur.010G114700 Sapur.010G123400 Sapur.010G128300 Sapur.010G134600
Sapur.010G136100 Sapur.010G141100 Sapur.010G149100 Sapur.010G151500 Sapur.010G154300 Sapur.010G161100
Sapur.010G169600 Sapur.010G193700 Sapur.011G006600 Sapur.011G008400 Sapur.011G014000 Sapur.011G032100
Sapur.011G035500 Sapur.011G037900 Sapur.011G038200 Sapur.011G039300 Sapur.011G041100 Sapur.011G042400
Sapur.011G045800 Sapur.011G049300 Sapur.011G054600 Sapur.011G056100 Sapur.011G061100 Sapur.011G064300
Sapur.011G066500 Sapur.011G079300 Sapur.011G088400 Sapur.011G088500 Sapur.011G101200 Sapur.012G000700
Sapur.012G004000 Sapur.012G010400 Sapur.012G017700 Sapur.012G018600 Sapur.012G020900 Sapur.012G024200
Sapur.012G024500 Sapur.012G031400 Sapur.012G051400 Sapur.012G055400 Sapur.012G060100 Sapur.012G067900
Sapur.012G078700 Sapur.012G084400 Sapur.012G105100 Sapur.013G000400 Sapur.013G008000 Sapur.013G010700
Sapur.013G038800 Sapur.013G039500 Sapur.013G041800 Sapur.013G050700 Sapur.013G050900 Sapur.013G055500
Sapur.013G059000 Sapur.013G067900 Sapur.013G068800 Sapur.013G082200 Sapur.013G087600 Sapur.013G103900
Sapur.013G108500 Sapur.013G126600 Sapur.013G128900 Sapur.013G135300 Sapur.013G137400 Sapur.013G138200
Sapur.013G141800 Sapur.014G003700 Sapur.014G004000 Sapur.014G005200 Sapur.014G034500 Sapur.014G053000

				Sapur.014G059000 Sapur.014G059100 Sapur.014G059200 Sapur.014G065300 Sapur.014G066300 Sapur.014G078300 Sapur.014G078500 Sapur.014G079300 Sapur.014G085400 Sapur.014G085500 Sapur.014G094500 Sapur.014G095700 Sapur.014G099500 Sapur.014G101000 Sapur.014G104700 Sapur.014G106600 Sapur.014G112900 Sapur.014G117700 Sapur.014G138900 Sapur.014G142200 Sapur.016G018700 Sapur.016G030200 Sapur.016G039500 Sapur.016G058600 Sapur.016G064600 Sapur.016G070400 Sapur.016G079900 Sapur.016G080200 Sapur.016G085100 Sapur.016G087700 Sapur.016G101200 Sapur.016G104000 Sapur.016G111900 Sapur.016G114700 Sapur.016G131300 Sapur.016G151000 Sapur.016G152700 Sapur.016G153200 Sapur.016G159000 Sapur.016G167800 Sapur.016G195300 Sapur.016G199700 Sapur.016G205800 Sapur.016G216900 Sapur.016G224700 Sapur.016G225800 Sapur.016G229800 Sapur.016G233500 Sapur.016G255700 Sapur.016G298800 Sapur.016G310400 Sapur.017G004800 Sapur.017G007100 Sapur.017G053300 Sapur.017G057100 Sapur.017G069900 Sapur.017G085900 Sapur.017G106300 Sapur.017G114800 Sapur.017G115700 Sapur.017G119700 Sapur.018G007100 Sapur.018G022500 Sapur.018G022700 Sapur.018G025000 Sapur.018G034800 Sapur.018G035300 Sapur.018G045400 Sapur.018G047900 Sapur.018G057700 Sapur.018G064600 Sapur.018G067800 Sapur.018G087600 Sapur.018G090600 Sapur.018G103000 Sapur.018G114600 Sapur.018G115000 Sapur.019G025000 Sapur.019G033200 Sapur.019G039400 Sapur.019G039900 Sapur.019G060100 Sapur.019G074500 Sapur.019G075900 Sapur.019G098000 Sapur.019G101700 Sapur.019G108100 Sapur.15WG032400 Sapur.15WG060500 Sapur.15WG063000 Sapur.15WG065100 Sapur.15WG072800 Sapur.15WG074800 Sapur.15ZG002000 Sapur.15ZG007300 Sapur.15ZG016900 Sapur.15ZG020500 Sapur.15ZG036200 Sapur.15ZG043500 Sapur.15ZG053200 Sapur.15ZG067200 Sapur.15ZG071900 Sapur.15ZG079000 Sapur.15ZG080300 Sapur.15ZG080900 Sapur.15ZG085300 Sapur.15ZG103400 Sapur.T006300 Sapur.T012000 Sapur.003G056300 Sapur.001G151600 Sapur.002G029000 Sapur.004G156500 Sapur.006G022000 Sapur.016G026400 Sapur.018G088200 Sapur.15ZG007500 Sapur.15ZG120200 Sapur.T047900 Sapur.002G047600 Sapur.004G123200 Sapur.008G019800 Sapur.008G054900 Sapur.009G099900 Sapur.013G094800 Sapur.013G094900 Sapur.013G095000 Sapur.013G095100 Sapur.001G057600 Sapur.002G194100 Sapur.003G002000 Sapur.003G116600 Sapur.003G168000 Sapur.006G136200 Sapur.007G112200 Sapur.008G063700 Sapur.010G009500 Sapur.010G137300 Sapur.010G163200 Sapur.014G098600 Sapur.017G020600
GO:0016070	348	1647	RNA metabolic process	Sapur.007G110800 Sapur.013G054700 Sapur.017G027400 Sapur.001G017300 Sapur.001G025400 Sapur.001G027900 Sapur.001G028100 Sapur.001G028400 Sapur.001G034200 Sapur.001G064300 Sapur.001G067700 Sapur.001G069800 Sapur.001G072500 Sapur.001G075700 Sapur.001G090900 Sapur.001G108900 Sapur.001G119600 Sapur.001G129000 Sapur.001G130000 Sapur.001G149200 Sapur.001G163400 Sapur.001G163900 Sapur.001G164600 Sapur.001G167800 Sapur.001G179800 Sapur.002G012900 Sapur.002G023100 Sapur.002G024200 Sapur.002G028500 Sapur.002G030000 Sapur.002G030100 Sapur.002G033200 Sapur.002G043300 Sapur.002G048900 Sapur.002G064600 Sapur.002G069500 Sapur.002G073000 Sapur.002G080700 Sapur.002G091700 Sapur.002G110600 Sapur.002G115400 Sapur.002G125900 Sapur.002G126000 Sapur.002G136900 Sapur.002G142300 Sapur.002G143500 Sapur.002G146000 Sapur.002G151100 Sapur.002G161200 Sapur.002G163600 Sapur.002G175800 Sapur.002G190900 Sapur.003G016800

Sapur.003G017600 Sapur.003G026500 Sapur.003G047900 Sapur.003G049200 Sapur.003G054700 Sapur.003G057500
Sapur.003G067100 Sapur.003G070500 Sapur.003G091600 Sapur.003G099900 Sapur.003G102000 Sapur.003G111600
Sapur.003G123800 Sapur.003G125900 Sapur.003G138200 Sapur.003G145700 Sapur.003G150800 Sapur.003G152800
Sapur.003G155000 Sapur.004G007000 Sapur.004G030500 Sapur.004G030800 Sapur.004G031600 Sapur.004G054800
Sapur.004G056900 Sapur.004G102300 Sapur.004G118800 Sapur.004G121500 Sapur.004G123100 Sapur.004G131600
Sapur.004G137000 Sapur.004G171400 Sapur.005G042000 Sapur.005G046200 Sapur.005G048400 Sapur.005G050200
Sapur.005G052900 Sapur.005G054800 Sapur.005G055100 Sapur.005G067000 Sapur.005G087400 Sapur.005G098100
Sapur.005G105500 Sapur.005G108500 Sapur.005G119200 Sapur.005G133200 Sapur.005G141300 Sapur.005G150000
Sapur.005G163900 Sapur.006G041100 Sapur.006G043000 Sapur.006G063200 Sapur.006G067500 Sapur.006G068600
Sapur.006G070000 Sapur.006G089400 Sapur.006G103400 Sapur.006G104700 Sapur.006G110100 Sapur.006G114200
Sapur.006G118700 Sapur.006G120000 Sapur.006G120300 Sapur.006G121100 Sapur.006G122700 Sapur.006G137200
Sapur.006G154200 Sapur.006G166000 Sapur.006G168000 Sapur.006G194100 Sapur.006G195200 Sapur.006G197300
Sapur.006G214400 Sapur.006G218000 Sapur.006G228700 Sapur.006G228900 Sapur.007G001300 Sapur.007G002900
Sapur.007G005500 Sapur.007G021900 Sapur.007G034000 Sapur.007G035800 Sapur.007G044500 Sapur.007G044600
Sapur.007G050900 Sapur.007G058700 Sapur.007G062200 Sapur.007G082500 Sapur.007G087500 Sapur.007G087800
Sapur.007G089800 Sapur.007G094000 Sapur.007G114800 Sapur.007G115600 Sapur.007G123400 Sapur.008G034200
Sapur.008G042700 Sapur.008G044200 Sapur.008G053100 Sapur.008G060600 Sapur.008G064800 Sapur.008G069200
Sapur.008G081900 Sapur.008G090400 Sapur.008G093800 Sapur.008G094300 Sapur.008G096700 Sapur.008G098100
Sapur.008G104300 Sapur.008G119700 Sapur.008G121600 Sapur.008G134800 Sapur.008G160400 Sapur.008G162200
Sapur.008G168800 Sapur.009G002400 Sapur.009G005300 Sapur.009G009700 Sapur.009G011600 Sapur.009G021700
Sapur.009G040000 Sapur.009G094800 Sapur.009G097900 Sapur.009G099800 Sapur.009G104900 Sapur.009G112500
Sapur.009G112800 Sapur.009G115000 Sapur.009G126000 Sapur.009G126600 Sapur.010G003100 Sapur.010G005200
Sapur.010G011800 Sapur.010G013000 Sapur.010G040000 Sapur.010G040100 Sapur.010G046500 Sapur.010G065900
Sapur.010G068700 Sapur.010G093900 Sapur.010G096300 Sapur.010G099700 Sapur.010G100200 Sapur.010G113700
Sapur.010G114700 Sapur.010G123400 Sapur.010G128300 Sapur.010G141100 Sapur.010G149100 Sapur.010G154300
Sapur.010G161100 Sapur.010G169600 Sapur.011G006600 Sapur.011G008400 Sapur.011G032100 Sapur.011G035500
Sapur.011G037900 Sapur.011G038200 Sapur.011G039300 Sapur.011G041100 Sapur.011G042400 Sapur.011G045800
Sapur.011G049300 Sapur.011G054600 Sapur.011G061100 Sapur.011G066500 Sapur.011G079300 Sapur.011G088400
Sapur.011G088500 Sapur.012G004000 Sapur.012G010400 Sapur.012G017700 Sapur.012G018600 Sapur.012G024500
Sapur.012G031400 Sapur.012G051400 Sapur.012G060100 Sapur.012G067900 Sapur.012G078700 Sapur.012G105100
Sapur.013G000400 Sapur.013G008000 Sapur.013G038800 Sapur.013G039500 Sapur.013G041800 Sapur.013G050700
Sapur.013G050900 Sapur.013G055500 Sapur.013G059000 Sapur.013G067900 Sapur.013G068800 Sapur.013G082200
Sapur.013G087600 Sapur.013G103900 Sapur.013G126600 Sapur.013G128900 Sapur.013G137400 Sapur.013G138200

				Sapur.013G141800 Sapur.014G003700 Sapur.014G004000 Sapur.014G005200 Sapur.014G034500 Sapur.014G053000 Sapur.014G059000 Sapur.014G059100 Sapur.014G059200 Sapur.014G065300 Sapur.014G078300 Sapur.014G078500 Sapur.014G079300 Sapur.014G085400 Sapur.014G085500 Sapur.014G094500 Sapur.014G095700 Sapur.014G099500 Sapur.014G101000 Sapur.014G112900 Sapur.016G018700 Sapur.016G030200 Sapur.016G058600 Sapur.016G064600 Sapur.016G070400 Sapur.016G079900 Sapur.016G080200 Sapur.016G085100 Sapur.016G087700 Sapur.016G111900 Sapur.016G114700 Sapur.016G131300 Sapur.016G152700 Sapur.016G153200 Sapur.016G159000 Sapur.016G167800 Sapur.016G199700 Sapur.016G205800 Sapur.016G216900 Sapur.016G224700 Sapur.016G225800 Sapur.016G229800 Sapur.016G233500 Sapur.016G255700 Sapur.016G298800 Sapur.016G310400 Sapur.017G004800 Sapur.017G007100 Sapur.017G053300 Sapur.017G057100 Sapur.017G069900 Sapur.017G085900 Sapur.017G114800 Sapur.017G115700 Sapur.018G007100 Sapur.018G022700 Sapur.018G025000 Sapur.018G034800 Sapur.018G035300 Sapur.018G057700 Sapur.018G064600 Sapur.018G067800 Sapur.018G090600 Sapur.018G114600 Sapur.018G115000 Sapur.019G025000 Sapur.019G033200 Sapur.019G039400 Sapur.019G039900 Sapur.019G060100 Sapur.019G074500 Sapur.019G098000 Sapur.019G101700 Sapur.019G108100 Sapur.15WG060500 Sapur.15WG065100 Sapur.15ZG002000 Sapur.15ZG007300 Sapur.15ZG016900 Sapur.15ZG053200 Sapur.15ZG067200 Sapur.15ZG071900 Sapur.15ZG079000 Sapur.15ZG080300 Sapur.15ZG080900 Sapur.15ZG103400 Sapur.T006300 Sapur.T012000 Sapur.002G029000 Sapur.002G194100 Sapur.003G002000 Sapur.003G168000 Sapur.006G136200 Sapur.007G112200 Sapur.010G009500 Sapur.010G163200 Sapur.017G020600
GO:0046483	439	2170	heterocycle metabolic process	Sapur.007G110800 Sapur.013G054700 Sapur.017G027400 Sapur.001G017300 Sapur.001G025400 Sapur.001G027900 Sapur.001G028100 Sapur.001G028400 Sapur.001G034200 Sapur.001G055600 Sapur.001G058800 Sapur.001G064300 Sapur.001G067700 Sapur.001G069800 Sapur.001G072500 Sapur.001G075700 Sapur.001G090900 Sapur.001G108900 Sapur.001G119600 Sapur.001G129000 Sapur.001G130000 Sapur.001G149200 Sapur.001G163400 Sapur.001G163900 Sapur.001G164600 Sapur.001G167800 Sapur.001G179800 Sapur.001G183500 Sapur.001G183600 Sapur.002G012900 Sapur.002G023100 Sapur.002G024200 Sapur.002G028500 Sapur.002G030000 Sapur.002G030100 Sapur.002G033200 Sapur.002G043300 Sapur.002G048900 Sapur.002G064600 Sapur.002G069500 Sapur.002G073000 Sapur.002G080700 Sapur.002G091700 Sapur.002G110600 Sapur.002G112100 Sapur.002G115400 Sapur.002G125900 Sapur.002G126000 Sapur.002G128100 Sapur.002G136900 Sapur.002G142300 Sapur.002G143500 Sapur.002G146000 Sapur.002G151100 Sapur.002G161200 Sapur.002G163600 Sapur.002G175800 Sapur.002G190900 Sapur.003G016800 Sapur.003G017600 Sapur.003G026500 Sapur.003G047900 Sapur.003G049200 Sapur.003G054700 Sapur.003G057500 Sapur.003G067100 Sapur.003G068400 Sapur.003G070500 Sapur.003G091600 Sapur.003G092400 Sapur.003G099900 Sapur.003G102000 Sapur.003G111600 Sapur.003G123800 Sapur.003G125900 Sapur.003G138200 Sapur.003G145700 Sapur.003G150800 Sapur.003G152800 Sapur.003G155000 Sapur.004G007000 Sapur.004G008500 Sapur.004G027800 Sapur.004G030500 Sapur.004G030800 Sapur.004G031600 Sapur.004G054800 Sapur.004G056900 Sapur.004G102300 Sapur.004G103000 Sapur.004G118800 Sapur.004G121500 Sapur.004G123100 Sapur.004G131600 Sapur.004G137000

Sapur.004G171400 Sapur.005G005600 Sapur.005G042000 Sapur.005G045400 Sapur.005G046200 Sapur.005G048400
Sapur.005G050200 Sapur.005G052900 Sapur.005G054800 Sapur.005G055100 Sapur.005G067000 Sapur.005G069000
Sapur.005G087400 Sapur.005G098100 Sapur.005G105500 Sapur.005G108500 Sapur.005G119200 Sapur.005G133200
Sapur.005G141300 Sapur.005G150000 Sapur.005G163900 Sapur.006G025100 Sapur.006G041100 Sapur.006G043000
Sapur.006G063200 Sapur.006G067500 Sapur.006G068600 Sapur.006G070000 Sapur.006G083400 Sapur.006G089400
Sapur.006G103400 Sapur.006G104700 Sapur.006G110100 Sapur.006G114200 Sapur.006G118700 Sapur.006G120000
Sapur.006G120300 Sapur.006G121100 Sapur.006G122700 Sapur.006G137200 Sapur.006G154200 Sapur.006G166000
Sapur.006G168000 Sapur.006G194100 Sapur.006G195200 Sapur.006G197300 Sapur.006G214400 Sapur.006G218000
Sapur.006G228700 Sapur.006G228900 Sapur.007G001300 Sapur.007G002900 Sapur.007G005500 Sapur.007G021900
Sapur.007G034000 Sapur.007G035800 Sapur.007G044500 Sapur.007G044600 Sapur.007G050900 Sapur.007G058700
Sapur.007G062200 Sapur.007G071200 Sapur.007G082500 Sapur.007G087500 Sapur.007G087800 Sapur.007G089800
Sapur.007G094000 Sapur.007G114800 Sapur.007G115600 Sapur.007G123400 Sapur.008G034200 Sapur.008G042700
Sapur.008G044200 Sapur.008G053100 Sapur.008G060600 Sapur.008G064800 Sapur.008G069200 Sapur.008G081900
Sapur.008G090400 Sapur.008G093800 Sapur.008G094300 Sapur.008G096700 Sapur.008G098100 Sapur.008G102800
Sapur.008G104300 Sapur.008G119700 Sapur.008G121600 Sapur.008G128300 Sapur.008G134800 Sapur.008G160400
Sapur.008G162200 Sapur.008G168800 Sapur.009G002400 Sapur.009G002900 Sapur.009G005300 Sapur.009G009700
Sapur.009G011600 Sapur.009G021700 Sapur.009G040000 Sapur.009G045200 Sapur.009G094800 Sapur.009G097900
Sapur.009G099800 Sapur.009G104900 Sapur.009G112500 Sapur.009G112800 Sapur.009G115000 Sapur.009G126000
Sapur.009G126600 Sapur.010G003100 Sapur.010G005200 Sapur.010G011800 Sapur.010G013000 Sapur.010G040000
Sapur.010G040100 Sapur.010G046500 Sapur.010G046700 Sapur.010G053800 Sapur.010G065900 Sapur.010G068700
Sapur.010G086900 Sapur.010G087900 Sapur.010G093900 Sapur.010G096300 Sapur.010G099700 Sapur.010G100200
Sapur.010G113700 Sapur.010G114700 Sapur.010G123400 Sapur.010G128300 Sapur.010G134600 Sapur.010G136100
Sapur.010G141100 Sapur.010G149100 Sapur.010G151500 Sapur.010G154300 Sapur.010G161100 Sapur.010G169600
Sapur.010G193700 Sapur.011G006600 Sapur.011G008400 Sapur.011G014000 Sapur.011G032100 Sapur.011G035500
Sapur.011G037900 Sapur.011G038200 Sapur.011G039300 Sapur.011G041100 Sapur.011G042400 Sapur.011G045800
Sapur.011G049300 Sapur.011G054600 Sapur.011G056100 Sapur.011G061100 Sapur.011G064300 Sapur.011G066500
Sapur.011G079300 Sapur.011G088400 Sapur.011G088500 Sapur.011G101200 Sapur.012G000700 Sapur.012G004000
Sapur.012G010400 Sapur.012G017700 Sapur.012G018600 Sapur.012G020900 Sapur.012G024200 Sapur.012G024500
Sapur.012G031400 Sapur.012G051400 Sapur.012G055400 Sapur.012G060100 Sapur.012G067900 Sapur.012G078700
Sapur.012G084400 Sapur.012G105100 Sapur.013G000400 Sapur.013G008000 Sapur.013G010700 Sapur.013G038800
Sapur.013G039500 Sapur.013G041800 Sapur.013G050700 Sapur.013G050900 Sapur.013G055500 Sapur.013G059000
Sapur.013G067900 Sapur.013G068800 Sapur.013G082200 Sapur.013G087600 Sapur.013G103900 Sapur.013G108500
Sapur.013G126600 Sapur.013G128900 Sapur.013G135300 Sapur.013G137400 Sapur.013G138200 Sapur.013G141800

				Sapur.014G003700 Sapur.014G004000 Sapur.014G005200 Sapur.014G034500 Sapur.014G053000 Sapur.014G059000 Sapur.014G059100 Sapur.014G059200 Sapur.014G065300 Sapur.014G066300 Sapur.014G078300 Sapur.014G078500 Sapur.014G079300 Sapur.014G085400 Sapur.014G085500 Sapur.014G094500 Sapur.014G095700 Sapur.014G099500 Sapur.014G101000 Sapur.014G104700 Sapur.014G106600 Sapur.014G112900 Sapur.014G117700 Sapur.014G138900 Sapur.014G142200 Sapur.016G018700 Sapur.016G030200 Sapur.016G039500 Sapur.016G058600 Sapur.016G064600 Sapur.016G070400 Sapur.016G079900 Sapur.016G080200 Sapur.016G085100 Sapur.016G087700 Sapur.016G101200 Sapur.016G104000 Sapur.016G111900 Sapur.016G114700 Sapur.016G131300 Sapur.016G151000 Sapur.016G152700 Sapur.016G153200 Sapur.016G159000 Sapur.016G167800 Sapur.016G195300 Sapur.016G199700 Sapur.016G205800 Sapur.016G216900 Sapur.016G224700 Sapur.016G225800 Sapur.016G229800 Sapur.016G233500 Sapur.016G255700 Sapur.016G298800 Sapur.016G310400 Sapur.017G004800 Sapur.017G007100 Sapur.017G053300 Sapur.017G057100 Sapur.017G069900 Sapur.017G085900 Sapur.017G106300 Sapur.017G114800 Sapur.017G115700 Sapur.017G119700 Sapur.018G007100 Sapur.018G022500 Sapur.018G022700 Sapur.018G025000 Sapur.018G034800 Sapur.018G035300 Sapur.018G045400 Sapur.018G047900 Sapur.018G057700 Sapur.018G064600 Sapur.018G067800 Sapur.018G087600 Sapur.018G090600 Sapur.018G103000 Sapur.018G114600 Sapur.018G115000 Sapur.019G025000 Sapur.019G033200 Sapur.019G039400 Sapur.019G039900 Sapur.019G060100 Sapur.019G074500 Sapur.019G075900 Sapur.019G098000 Sapur.019G101700 Sapur.019G108100 Sapur.15WG032400 Sapur.15WG060500 Sapur.15WG063000 Sapur.15WG065100 Sapur.15WG072800 Sapur.15WG074800 Sapur.15ZG002000 Sapur.15ZG007300 Sapur.15ZG016900 Sapur.15ZG020500 Sapur.15ZG036200 Sapur.15ZG043500 Sapur.15ZG053200 Sapur.15ZG067200 Sapur.15ZG071900 Sapur.15ZG079000 Sapur.15ZG080300 Sapur.15ZG080900 Sapur.15ZG085300 Sapur.15ZG103400 Sapur.T006300 Sapur.T012000 Sapur.003G056300 Sapur.001G151600 Sapur.002G029000 Sapur.004G156500 Sapur.006G022000 Sapur.016G026400 Sapur.018G088200 Sapur.15ZG007500 Sapur.15ZG120200 Sapur.T047900 Sapur.002G047600 Sapur.004G123200 Sapur.008G019800 Sapur.008G054900 Sapur.009G099900 Sapur.013G094800 Sapur.013G094900 Sapur.013G095000 Sapur.013G095100 Sapur.001G057600 Sapur.002G194100 Sapur.003G002000 Sapur.003G116600 Sapur.003G168000 Sapur.006G136200 Sapur.007G112200 Sapur.008G063700 Sapur.010G009500 Sapur.010G137300 Sapur.010G163200 Sapur.014G098600 Sapur.017G020600
GO:0090304	392	1921	nucleic acid metabolic process	Sapur.007G110800 Sapur.013G054700 Sapur.017G027400 Sapur.001G017300 Sapur.001G025400 Sapur.001G027900 Sapur.001G028100 Sapur.001G028400 Sapur.001G034200 Sapur.001G055600 Sapur.001G058800 Sapur.001G064300 Sapur.001G067700 Sapur.001G069800 Sapur.001G072500 Sapur.001G075700 Sapur.001G090900 Sapur.001G108900 Sapur.001G119600 Sapur.001G129000 Sapur.001G130000 Sapur.001G149200 Sapur.001G163400 Sapur.001G163900 Sapur.001G164600 Sapur.001G167800 Sapur.001G179800 Sapur.001G183600 Sapur.002G012900 Sapur.002G023100 Sapur.002G024200 Sapur.002G028500 Sapur.002G030000 Sapur.002G030100 Sapur.002G033200 Sapur.002G043300 Sapur.002G048900 Sapur.002G064600 Sapur.002G069500 Sapur.002G073000 Sapur.002G080700 Sapur.002G091700 Sapur.002G110600 Sapur.002G115400 Sapur.002G125900 Sapur.002G126000 Sapur.002G136900

Sapur.002G142300	Sapur.002G143500	Sapur.002G146000	Sapur.002G151100	Sapur.002G161200	Sapur.002G163600
Sapur.002G175800	Sapur.002G190900	Sapur.003G016800	Sapur.003G017600	Sapur.003G026500	Sapur.003G047900
Sapur.003G049200	Sapur.003G054700	Sapur.003G057500	Sapur.003G067100	Sapur.003G068400	Sapur.003G070500
Sapur.003G091600	Sapur.003G099900	Sapur.003G102000	Sapur.003G111600	Sapur.003G123800	Sapur.003G125900
Sapur.003G138200	Sapur.003G145700	Sapur.003G150800	Sapur.003G152800	Sapur.003G155000	Sapur.004G007000
Sapur.004G027800	Sapur.004G030500	Sapur.004G030800	Sapur.004G031600	Sapur.004G054800	Sapur.004G056900
Sapur.004G102300	Sapur.004G103000	Sapur.004G118800	Sapur.004G121500	Sapur.004G123100	Sapur.004G131600
Sapur.004G137000	Sapur.004G171400	Sapur.005G005600	Sapur.005G042000	Sapur.005G045400	Sapur.005G046200
Sapur.005G048400	Sapur.005G050200	Sapur.005G052900	Sapur.005G054800	Sapur.005G055100	Sapur.005G067000
Sapur.005G069000	Sapur.005G087400	Sapur.005G098100	Sapur.005G105500	Sapur.005G108500	Sapur.005G119200
Sapur.005G133200	Sapur.005G141300	Sapur.005G150000	Sapur.005G163900	Sapur.006G025100	Sapur.006G041100
Sapur.006G043000	Sapur.006G063200	Sapur.006G067500	Sapur.006G068600	Sapur.006G070000	Sapur.006G089400
Sapur.006G103400	Sapur.006G104700	Sapur.006G110100	Sapur.006G114200	Sapur.006G118700	Sapur.006G120000
Sapur.006G120300	Sapur.006G121100	Sapur.006G122700	Sapur.006G137200	Sapur.006G154200	Sapur.006G166000
Sapur.006G168000	Sapur.006G194100	Sapur.006G195200	Sapur.006G197300	Sapur.006G214400	Sapur.006G218000
Sapur.006G228700	Sapur.006G228900	Sapur.007G001300	Sapur.007G002900	Sapur.007G005500	Sapur.007G021900
Sapur.007G034000	Sapur.007G035800	Sapur.007G044500	Sapur.007G044600	Sapur.007G050900	Sapur.007G058700
Sapur.007G062200	Sapur.007G071200	Sapur.007G082500	Sapur.007G087500	Sapur.007G087800	Sapur.007G089800
Sapur.007G094000	Sapur.007G114800	Sapur.007G115600	Sapur.007G123400	Sapur.008G034200	Sapur.008G042700
Sapur.008G044200	Sapur.008G053100	Sapur.008G060600	Sapur.008G064800	Sapur.008G069200	Sapur.008G081900
Sapur.008G090400	Sapur.008G093800	Sapur.008G094300	Sapur.008G096700	Sapur.008G098100	Sapur.008G104300
Sapur.008G119700	Sapur.008G121600	Sapur.008G128300	Sapur.008G134800	Sapur.008G160400	Sapur.008G162200
Sapur.008G168800	Sapur.009G002400	Sapur.009G002900	Sapur.009G005300	Sapur.009G009700	Sapur.009G011600
Sapur.009G021700	Sapur.009G040000	Sapur.009G045200	Sapur.009G094800	Sapur.009G097900	Sapur.009G099800
Sapur.009G104900	Sapur.009G112500	Sapur.009G112800	Sapur.009G115000	Sapur.009G126000	Sapur.009G126600
Sapur.010G003100	Sapur.010G005200	Sapur.010G011800	Sapur.010G013000	Sapur.010G040000	Sapur.010G040100
Sapur.010G046500	Sapur.010G046700	Sapur.010G053800	Sapur.010G065900	Sapur.010G068700	Sapur.010G093900
Sapur.010G096300	Sapur.010G099700	Sapur.010G100200	Sapur.010G113700	Sapur.010G114700	Sapur.010G123400
Sapur.010G128300	Sapur.010G136100	Sapur.010G141100	Sapur.010G149100	Sapur.010G154300	Sapur.010G161100
Sapur.010G169600	Sapur.010G193700	Sapur.011G006600	Sapur.011G008400	Sapur.011G032100	Sapur.011G035500
Sapur.011G037900	Sapur.011G038200	Sapur.011G039300	Sapur.011G041100	Sapur.011G042400	Sapur.011G045800
Sapur.011G049300	Sapur.011G054600	Sapur.011G056100	Sapur.011G061100	Sapur.011G066500	Sapur.011G079300
Sapur.011G088400	Sapur.011G088500	Sapur.011G101200	Sapur.012G000700	Sapur.012G004000	Sapur.012G010400

				Sapur.012G017700 Sapur.012G018600 Sapur.012G020900 Sapur.012G024200 Sapur.012G024500 Sapur.012G031400 Sapur.012G051400 Sapur.012G060100 Sapur.012G067900 Sapur.012G078700 Sapur.012G105100 Sapur.013G000400 Sapur.013G008000 Sapur.013G010700 Sapur.013G038800 Sapur.013G039500 Sapur.013G041800 Sapur.013G050700 Sapur.013G050900 Sapur.013G055500 Sapur.013G059000 Sapur.013G067900 Sapur.013G068800 Sapur.013G082200 Sapur.013G087600 Sapur.013G103900 Sapur.013G126600 Sapur.013G128900 Sapur.013G135300 Sapur.013G137400 Sapur.013G138200 Sapur.013G141800 Sapur.014G003700 Sapur.014G004000 Sapur.014G005200 Sapur.014G034500 Sapur.014G053000 Sapur.014G059000 Sapur.014G059100 Sapur.014G059200 Sapur.014G065300 Sapur.014G066300 Sapur.014G078300 Sapur.014G078500 Sapur.014G079300 Sapur.014G085400 Sapur.014G085500 Sapur.014G094500 Sapur.014G095700 Sapur.014G099500 Sapur.014G101000 Sapur.014G104700 Sapur.014G106600 Sapur.014G112900 Sapur.014G117700 Sapur.014G138900 Sapur.014G142200 Sapur.016G018700 Sapur.016G030200 Sapur.016G039500 Sapur.016G058600 Sapur.016G064600 Sapur.016G070400 Sapur.016G079900 Sapur.016G080200 Sapur.016G085100 Sapur.016G087700 Sapur.016G111900 Sapur.016G114700 Sapur.016G131300 Sapur.016G151000 Sapur.016G152700 Sapur.016G153200 Sapur.016G159000 Sapur.016G167800 Sapur.016G195300 Sapur.016G199700 Sapur.016G205800 Sapur.016G216900 Sapur.016G224700 Sapur.016G225800 Sapur.016G229800 Sapur.016G233500 Sapur.016G255700 Sapur.016G298800 Sapur.016G310400 Sapur.017G004800 Sapur.017G007100 Sapur.017G053300 Sapur.017G057100 Sapur.017G069900 Sapur.017G085900 Sapur.017G114800 Sapur.017G115700 Sapur.017G119700 Sapur.018G007100 Sapur.018G022700 Sapur.018G025000 Sapur.018G034800 Sapur.018G035300 Sapur.018G057700 Sapur.018G064600 Sapur.018G067800 Sapur.018G087600 Sapur.018G090600 Sapur.018G114600 Sapur.018G115000 Sapur.019G025000 Sapur.019G033200 Sapur.019G039400 Sapur.019G039900 Sapur.019G060100 Sapur.019G074500 Sapur.019G075900 Sapur.019G098000 Sapur.019G101700 Sapur.019G108100 Sapur.15WG032400 Sapur.15WG060500 Sapur.15WG065100 Sapur.15WG072800 Sapur.15WG074800 Sapur.15ZG002000 Sapur.15ZG007300 Sapur.15ZG016900 Sapur.15ZG020500 Sapur.15ZG036200 Sapur.15ZG043500 Sapur.15ZG053200 Sapur.15ZG067200 Sapur.15ZG071900 Sapur.15ZG079000 Sapur.15ZG080300 Sapur.15ZG080900 Sapur.15ZG103400 Sapur.T006300 Sapur.T012000 Sapur.002G029000 Sapur.T047900 Sapur.002G194100 Sapur.003G002000 Sapur.003G168000 Sapur.006G136200 Sapur.007G112200 Sapur.010G009500 Sapur.010G163200 Sapur.017G020600
GO:0000278	19	31	mitotic cell cycle	Sapur.001G103000 Sapur.002G001600 Sapur.002G070300 Sapur.004G071100 Sapur.005G135200 Sapur.006G030400 Sapur.006G065500 Sapur.007G123700 Sapur.008G057000 Sapur.008G145300 Sapur.010G008600 Sapur.010G056400 Sapur.010G145400 Sapur.012G020900 Sapur.012G099900 Sapur.014G127000 Sapur.017G006400 Sapur.017G006500 Sapur.15WG062900
GO:1901360	443	2218	organic cyclic compound metabolic process	Sapur.007G110800 Sapur.013G054700 Sapur.017G027400 Sapur.001G017300 Sapur.001G025400 Sapur.001G027900 Sapur.001G028100 Sapur.001G028400 Sapur.001G034200 Sapur.001G055600 Sapur.001G058800 Sapur.001G064300 Sapur.001G067700 Sapur.001G069800 Sapur.001G072500 Sapur.001G075700 Sapur.001G090900 Sapur.001G108900 Sapur.001G119600 Sapur.001G129000 Sapur.001G130000 Sapur.001G149200 Sapur.001G163400

	Sapur.001G163900	Sapur.001G164600	Sapur.001G167800	Sapur.001G179800	Sapur.001G183500	Sapur.001G183600
	Sapur.002G012900	Sapur.002G023100	Sapur.002G024200	Sapur.002G028500	Sapur.002G030000	Sapur.002G030100
	Sapur.002G033200	Sapur.002G043300	Sapur.002G048900	Sapur.002G064600	Sapur.002G069500	Sapur.002G073000
	Sapur.002G080700	Sapur.002G091700	Sapur.002G110600	Sapur.002G112100	Sapur.002G115400	Sapur.002G125900
	Sapur.002G126000	Sapur.002G128100	Sapur.002G136900	Sapur.002G142300	Sapur.002G143500	Sapur.002G146000
	Sapur.002G151100	Sapur.002G161200	Sapur.002G163600	Sapur.002G175800	Sapur.002G190900	Sapur.003G016800
	Sapur.003G017600	Sapur.003G026500	Sapur.003G047900	Sapur.003G049200	Sapur.003G054700	Sapur.003G057500
	Sapur.003G060900	Sapur.003G067100	Sapur.003G068400	Sapur.003G070500	Sapur.003G091600	Sapur.003G092400
	Sapur.003G099900	Sapur.003G102000	Sapur.003G111600	Sapur.003G123800	Sapur.003G125900	Sapur.003G138200
	Sapur.003G145700	Sapur.003G150800	Sapur.003G152800	Sapur.003G155000	Sapur.004G007000	Sapur.004G008500
	Sapur.004G027800	Sapur.004G030500	Sapur.004G030800	Sapur.004G031600	Sapur.004G054800	Sapur.004G056900
	Sapur.004G102300	Sapur.004G103000	Sapur.004G118800	Sapur.004G121500	Sapur.004G123100	Sapur.004G131600
	Sapur.004G137000	Sapur.004G171400	Sapur.005G005600	Sapur.005G042000	Sapur.005G045400	Sapur.005G046200
	Sapur.005G048400	Sapur.005G050200	Sapur.005G052900	Sapur.005G054800	Sapur.005G055100	Sapur.005G067000
	Sapur.005G069000	Sapur.005G087400	Sapur.005G098100	Sapur.005G105500	Sapur.005G108500	Sapur.005G119200
	Sapur.005G133200	Sapur.005G141300	Sapur.005G150000	Sapur.005G163900	Sapur.006G025100	Sapur.006G041100
	Sapur.006G043000	Sapur.006G049700	Sapur.006G063200	Sapur.006G067500	Sapur.006G068600	Sapur.006G070000
	Sapur.006G083400	Sapur.006G089400	Sapur.006G103400	Sapur.006G104700	Sapur.006G110100	Sapur.006G114200
	Sapur.006G118700	Sapur.006G120000	Sapur.006G120300	Sapur.006G121100	Sapur.006G122700	Sapur.006G137200
	Sapur.006G154200	Sapur.006G166000	Sapur.006G168000	Sapur.006G194100	Sapur.006G195200	Sapur.006G197300
	Sapur.006G214400	Sapur.006G218000	Sapur.006G228700	Sapur.006G228900	Sapur.007G001300	Sapur.007G002900
	Sapur.007G005500	Sapur.007G021900	Sapur.007G034000	Sapur.007G035800	Sapur.007G044500	Sapur.007G044600
	Sapur.007G050900	Sapur.007G058700	Sapur.007G062200	Sapur.007G071200	Sapur.007G082500	Sapur.007G087500
	Sapur.007G087800	Sapur.007G089800	Sapur.007G094000	Sapur.007G114800	Sapur.007G115600	Sapur.007G123400
	Sapur.008G034200	Sapur.008G042700	Sapur.008G044200	Sapur.008G053100	Sapur.008G060600	Sapur.008G064800
	Sapur.008G069200	Sapur.008G081900	Sapur.008G090400	Sapur.008G093800	Sapur.008G094300	Sapur.008G096700
	Sapur.008G098100	Sapur.008G102800	Sapur.008G104300	Sapur.008G119700	Sapur.008G121600	Sapur.008G128300
	Sapur.008G134800	Sapur.008G160400	Sapur.008G162200	Sapur.008G168800	Sapur.009G002400	Sapur.009G002900
	Sapur.009G005300	Sapur.009G009700	Sapur.009G011600	Sapur.009G021700	Sapur.009G040000	Sapur.009G045200
	Sapur.009G094800	Sapur.009G097900	Sapur.009G099800	Sapur.009G104900	Sapur.009G112500	Sapur.009G112800
	Sapur.009G115000	Sapur.009G126000	Sapur.009G126600	Sapur.010G003100	Sapur.010G005200	Sapur.010G011800
	Sapur.010G013000	Sapur.010G040000	Sapur.010G040100	Sapur.010G046500	Sapur.010G046700	Sapur.010G053800
	Sapur.010G065900	Sapur.010G068700	Sapur.010G086900	Sapur.010G087900	Sapur.010G093900	Sapur.010G096300

Sapur.010G099700 Sapur.010G100200 Sapur.010G113700 Sapur.010G114700 Sapur.010G123400 Sapur.010G128300
Sapur.010G134600 Sapur.010G136100 Sapur.010G141100 Sapur.010G149100 Sapur.010G151500 Sapur.010G154300
Sapur.010G161100 Sapur.010G169600 Sapur.010G193700 Sapur.011G006600 Sapur.011G008400 Sapur.011G014000
Sapur.011G032100 Sapur.011G035500 Sapur.011G037900 Sapur.011G038200 Sapur.011G039300 Sapur.011G041100
Sapur.011G042400 Sapur.011G045800 Sapur.011G049300 Sapur.011G054600 Sapur.011G056100 Sapur.011G061100
Sapur.011G064300 Sapur.011G066500 Sapur.011G079300 Sapur.011G088400 Sapur.011G088500 Sapur.011G101200
Sapur.012G000700 Sapur.012G004000 Sapur.012G010400 Sapur.012G017700 Sapur.012G018600 Sapur.012G020900
Sapur.012G024200 Sapur.012G024500 Sapur.012G031400 Sapur.012G051400 Sapur.012G055400 Sapur.012G060100
Sapur.012G067900 Sapur.012G078700 Sapur.012G084400 Sapur.012G105100 Sapur.013G000400 Sapur.013G008000
Sapur.013G010700 Sapur.013G038800 Sapur.013G039500 Sapur.013G041800 Sapur.013G050700 Sapur.013G050900
Sapur.013G055500 Sapur.013G059000 Sapur.013G067900 Sapur.013G068800 Sapur.013G082200 Sapur.013G087600
Sapur.013G103900 Sapur.013G108500 Sapur.013G126600 Sapur.013G128900 Sapur.013G135300 Sapur.013G137400
Sapur.013G138200 Sapur.013G141800 Sapur.014G003700 Sapur.014G004000 Sapur.014G005200 Sapur.014G034500
Sapur.014G053000 Sapur.014G059000 Sapur.014G059100 Sapur.014G059200 Sapur.014G065300 Sapur.014G066300
Sapur.014G078300 Sapur.014G078500 Sapur.014G079300 Sapur.014G085400 Sapur.014G085500 Sapur.014G094500
Sapur.014G095700 Sapur.014G099500 Sapur.014G101000 Sapur.014G104700 Sapur.014G106600 Sapur.014G112900
Sapur.014G117700 Sapur.014G138900 Sapur.014G142200 Sapur.016G016700 Sapur.016G018700 Sapur.016G030200
Sapur.016G039500 Sapur.016G058600 Sapur.016G064600 Sapur.016G070400 Sapur.016G079900 Sapur.016G080200
Sapur.016G085100 Sapur.016G087700 Sapur.016G101200 Sapur.016G104000 Sapur.016G111900 Sapur.016G114700
Sapur.016G131300 Sapur.016G151000 Sapur.016G152700 Sapur.016G153200 Sapur.016G159000 Sapur.016G167800
Sapur.016G195300 Sapur.016G199700 Sapur.016G205800 Sapur.016G216900 Sapur.016G224700 Sapur.016G225800
Sapur.016G229800 Sapur.016G233500 Sapur.016G255700 Sapur.016G298800 Sapur.016G310400 Sapur.017G004800
Sapur.017G007100 Sapur.017G053300 Sapur.017G057100 Sapur.017G069900 Sapur.017G085900 Sapur.017G089700
Sapur.017G106300 Sapur.017G114800 Sapur.017G115700 Sapur.017G119700 Sapur.018G007100 Sapur.018G022500
Sapur.018G022700 Sapur.018G025000 Sapur.018G034800 Sapur.018G035300 Sapur.018G045400 Sapur.018G047900
Sapur.018G057700 Sapur.018G064600 Sapur.018G067800 Sapur.018G087600 Sapur.018G090600 Sapur.018G103000
Sapur.018G114600 Sapur.018G115000 Sapur.019G025000 Sapur.019G033200 Sapur.019G039400 Sapur.019G039900
Sapur.019G060100 Sapur.019G074500 Sapur.019G075900 Sapur.019G098000 Sapur.019G101700 Sapur.019G108100
Sapur.15WG032400 Sapur.15WG060500 Sapur.15WG063000 Sapur.15WG065100 Sapur.15WG072800 Sapur.15WG074800
Sapur.15ZG002000 Sapur.15ZG007300 Sapur.15ZG016900 Sapur.15ZG020500 Sapur.15ZG036200 Sapur.15ZG043500
Sapur.15ZG053200 Sapur.15ZG067200 Sapur.15ZG071900 Sapur.15ZG079000 Sapur.15ZG080300 Sapur.15ZG080900
Sapur.15ZG085300 Sapur.15ZG103400 Sapur.T006300 Sapur.T012000 Sapur.003G056300 Sapur.001G151600
Sapur.002G029000 Sapur.004G156500 Sapur.006G022000 Sapur.016G026400 Sapur.018G088200 Sapur.15ZG007500

				Sapur.15ZG120200 Sapur.T047900 Sapur.002G047600 Sapur.004G123200 Sapur.008G019800 Sapur.008G054900 Sapur.009G099900 Sapur.013G094800 Sapur.013G094900 Sapur.013G095000 Sapur.013G095100 Sapur.001G057600 Sapur.002G194100 Sapur.003G002000 Sapur.003G116600 Sapur.003G168000 Sapur.006G136200 Sapur.007G112200 Sapur.008G063700 Sapur.010G009500 Sapur.010G137300 Sapur.010G163200 Sapur.014G098600 Sapur.017G020600
GO:1903047	18	30	mitotic cell cycle process	Sapur.001G103000 Sapur.002G001600 Sapur.002G070300 Sapur.004G071100 Sapur.005G135200 Sapur.006G030400 Sapur.007G123700 Sapur.008G057000 Sapur.008G145300 Sapur.010G008600 Sapur.010G056400 Sapur.010G145400 Sapur.012G020900 Sapur.012G099900 Sapur.014G127000 Sapur.017G006400 Sapur.017G006500 Sapur.15WG062900
GO:0042908	29	68	xenobiotic transport	Sapur.001G094500 Sapur.002G043400 Sapur.002G086100 Sapur.003G081500 Sapur.004G009600 Sapur.004G009700 Sapur.004G138100 Sapur.005G082300 Sapur.005G114300 Sapur.005G163600 Sapur.005G163800 Sapur.008G101500 Sapur.008G101600 Sapur.008G101700 Sapur.009G040500 Sapur.009G097300 Sapur.010G089300 Sapur.011G001000 Sapur.011G080900 Sapur.012G102500 Sapur.012G111400 Sapur.013G071900 Sapur.013G072500 Sapur.013G072600 Sapur.013G072700 Sapur.016G153700 Sapur.019G046000 Sapur.019G064700 Sapur.019G083100
GO:0006855	29	68	xenobiotic transmembrane transport	Sapur.001G094500 Sapur.002G043400 Sapur.002G086100 Sapur.003G081500 Sapur.004G009600 Sapur.004G009700 Sapur.004G138100 Sapur.005G082300 Sapur.005G114300 Sapur.005G163600 Sapur.005G163800 Sapur.008G101500 Sapur.008G101600 Sapur.008G101700 Sapur.009G040500 Sapur.009G097300 Sapur.010G089300 Sapur.011G001000 Sapur.011G080900 Sapur.012G102500 Sapur.012G111400 Sapur.013G071900 Sapur.013G072500 Sapur.013G072600 Sapur.013G072700 Sapur.016G153700 Sapur.019G046000 Sapur.019G064700 Sapur.019G083100
GO:0022402	21	48	cell cycle process	Sapur.001G103000 Sapur.002G001600 Sapur.002G070300 Sapur.004G071100 Sapur.005G135200 Sapur.006G030400 Sapur.006G065500 Sapur.007G123700 Sapur.008G057000 Sapur.008G145300 Sapur.009G003100 Sapur.010G008600 Sapur.010G056400 Sapur.010G145400 Sapur.010G183500 Sapur.012G020900 Sapur.012G099900 Sapur.014G127000 Sapur.017G006400 Sapur.017G006500 Sapur.15WG062900
GO:0010564	15	29	regulation of cell cycle process	Sapur.002G001600 Sapur.002G070300 Sapur.004G071100 Sapur.005G135200 Sapur.006G065500 Sapur.007G123700 Sapur.008G057000 Sapur.008G145300 Sapur.010G056400 Sapur.010G145400 Sapur.012G020900 Sapur.012G099900 Sapur.017G006400 Sapur.017G006500 Sapur.15WG062900
GO:0000280	11	20	nuclear division	Sapur.001G103000 Sapur.002G001600 Sapur.006G030400 Sapur.008G057000 Sapur.008G145300 Sapur.009G003100 Sapur.010G008600 Sapur.010G056400 Sapur.010G145400 Sapur.010G183500 Sapur.014G127000
GO:0009725	15	34	response to hormone	Sapur.001G072500 Sapur.002G043300 Sapur.004G054800 Sapur.005G163900 Sapur.006G063200 Sapur.006G104700 Sapur.006G114200 Sapur.009G009700 Sapur.011G041100 Sapur.014G078500 Sapur.016G085100 Sapur.016G229800 Sapur.017G115700 Sapur.15ZG103400 Sapur.T012000
GO:0009719	15	34	response to endogenous stimulus	Sapur.001G072500 Sapur.002G043300 Sapur.004G054800 Sapur.005G163900 Sapur.006G063200 Sapur.006G104700 Sapur.006G114200 Sapur.009G009700 Sapur.011G041100 Sapur.014G078500 Sapur.016G085100 Sapur.016G229800 Sapur.017G115700 Sapur.15ZG103400 Sapur.T012000

GO:0098813	11	21	nuclear chromosome segregation	Sapur.001G103000 Sapur.002G001600 Sapur.006G030400 Sapur.008G057000 Sapur.008G145300 Sapur.009G003100 Sapur.010G008600 Sapur.010G056400 Sapur.010G145400 Sapur.010G183500 Sapur.014G127000
GO:0140014	9	15	mitotic nuclear division	Sapur.001G103000 Sapur.002G001600 Sapur.006G030400 Sapur.008G057000 Sapur.008G145300 Sapur.010G008600 Sapur.010G056400 Sapur.010G145400 Sapur.014G127000
GO:0000070	9	15	mitotic sister chromatid segregation	Sapur.001G103000 Sapur.002G001600 Sapur.006G030400 Sapur.008G057000 Sapur.008G145300 Sapur.010G008600 Sapur.010G056400 Sapur.010G145400 Sapur.014G127000
GO:0007059	14	32	chromosome segregation	Sapur.001G103000 Sapur.002G001600 Sapur.006G030400 Sapur.006G065500 Sapur.008G057000 Sapur.008G145300 Sapur.009G003100 Sapur.010G008600 Sapur.010G056400 Sapur.010G145400 Sapur.010G183500 Sapur.014G127000 Sapur.002G070300 Sapur.005G135200
GO:0007018	32	106	microtubule-based movement	Sapur.001G094200 Sapur.002G064800 Sapur.002G085200 Sapur.002G089300 Sapur.004G019100 Sapur.004G109700 Sapur.004G147000 Sapur.005G022300 Sapur.006G036800 Sapur.006G067400 Sapur.006G069500 Sapur.006G112500 Sapur.006G172300 Sapur.008G137200 Sapur.009G122200 Sapur.010G042700 Sapur.010G119100 Sapur.011G018500 Sapur.011G103700 Sapur.012G037400 Sapur.012G040700 Sapur.014G005100 Sapur.014G052300 Sapur.016G071200 Sapur.018G057600 Sapur.15WG040400 Sapur.15WG044400 Sapur.15ZG044100 Sapur.15ZG044300 Sapur.15ZG044900 Sapur.15ZG049500 Sapur.T003900
GO:0009628	8	13	response to abiotic stimulus	Sapur.009G112000 Sapur.010G065900 Sapur.012G020900 Sapur.002G008900 Sapur.005G198700 Sapur.010G113700 Sapur.013G000400 Sapur.15WG060500
GO:0045930	8	13	negative regulation of mitotic cell cycle	Sapur.002G001600 Sapur.002G070300 Sapur.005G135200 Sapur.008G057000 Sapur.008G145300 Sapur.010G056400 Sapur.010G145400 Sapur.012G020900
GO:0007093	8	13	mitotic cell cycle checkpoint signaling	Sapur.002G001600 Sapur.002G070300 Sapur.005G135200 Sapur.008G057000 Sapur.008G145300 Sapur.010G056400 Sapur.010G145400 Sapur.012G020900
GO:0010033	18	48	response to organic substance	Sapur.001G072500 Sapur.002G043300 Sapur.004G054800 Sapur.005G163900 Sapur.006G063200 Sapur.006G104700 Sapur.006G114200 Sapur.009G009700 Sapur.011G041100 Sapur.014G078500 Sapur.016G085100 Sapur.016G118000 Sapur.016G229800 Sapur.017G115700 Sapur.15ZG103400 Sapur.T012000 Sapur.002G008900 Sapur.005G198700
GO:0044271	358	1913	cellular nitrogen compound biosynthetic	Sapur.001G017300 Sapur.001G025400 Sapur.001G027900 Sapur.001G028100 Sapur.001G028400 Sapur.001G034200 Sapur.001G064300 Sapur.001G067700 Sapur.001G069800 Sapur.001G072500 Sapur.001G075700 Sapur.001G090900 Sapur.001G108900 Sapur.001G119600 Sapur.001G129000 Sapur.001G130000 Sapur.001G163400

process	Sapur.001G164600 Sapur.001G179800 Sapur.001G183500 Sapur.002G012900 Sapur.002G023100 Sapur.002G024200<br>Sapur.002G028500 Sapur.002G030000 Sapur.002G030100 Sapur.002G033200 Sapur.002G041000 Sapur.002G043300<br>Sapur.002G064600 Sapur.002G069500 Sapur.002G073000 Sapur.002G080700 Sapur.002G091700 Sapur.002G110600<br>Sapur.002G112100 Sapur.002G115400 Sapur.002G125900 Sapur.002G126000 Sapur.002G128100 Sapur.002G136900<br>Sapur.002G142300 Sapur.002G143500 Sapur.002G146000 Sapur.002G151100 Sapur.002G161200 Sapur.002G163600<br>Sapur.002G175800 Sapur.002G190900 Sapur.003G017600 Sapur.003G026500 Sapur.003G047900 Sapur.003G049200<br>Sapur.003G057500 Sapur.003G067100 Sapur.003G070500 Sapur.003G091600 Sapur.003G092400 Sapur.003G099900<br>Sapur.003G102000 Sapur.003G111600 Sapur.003G117300 Sapur.003G123800 Sapur.003G125900 Sapur.003G138200<br>Sapur.003G145700 Sapur.003G150800 Sapur.003G152800 Sapur.004G002900 Sapur.004G007000 Sapur.004G008500<br>Sapur.004G030500 Sapur.004G030800 Sapur.004G031600 Sapur.004G054800 Sapur.004G056900 Sapur.004G078500<br>Sapur.004G118800 Sapur.004G121500 Sapur.004G122800 Sapur.004G123100 Sapur.004G131600 Sapur.004G137000<br>Sapur.004G171400 Sapur.004G176300 Sapur.005G042000 Sapur.005G052900 Sapur.005G054800 Sapur.005G055100<br>Sapur.005G067000 Sapur.005G082000 Sapur.005G087400 Sapur.005G098100 Sapur.005G105500 Sapur.005G108500<br>Sapur.005G133200 Sapur.005G141300 Sapur.005G150000 Sapur.005G152800 Sapur.005G163900 Sapur.006G027800<br>Sapur.006G033400 Sapur.006G041100 Sapur.006G043000 Sapur.006G063200 Sapur.006G067500 Sapur.006G068600<br>Sapur.006G070000 Sapur.006G083400 Sapur.006G089400 Sapur.006G092800 Sapur.006G103400 Sapur.006G104700<br>Sapur.006G110100 Sapur.006G114200 Sapur.006G118700 Sapur.006G120000 Sapur.006G120300 Sapur.006G122700<br>Sapur.006G137200 Sapur.006G166000 Sapur.006G168000 Sapur.006G194100 Sapur.006G195200 Sapur.006G197300<br>Sapur.006G214400 Sapur.006G218000 Sapur.006G228700 Sapur.006G228900 Sapur.007G001300 Sapur.007G002900<br>Sapur.007G005500 Sapur.007G021900 Sapur.007G034000 Sapur.007G035800 Sapur.007G044600 Sapur.007G050900<br>Sapur.007G058700 Sapur.007G062200 Sapur.007G082500 Sapur.007G087500 Sapur.007G087800 Sapur.007G089800<br>Sapur.007G094000 Sapur.007G114800 Sapur.007G115600 Sapur.007G123400 Sapur.008G034200 Sapur.008G042700<br>Sapur.008G053100 Sapur.008G060200 Sapur.008G064800 Sapur.008G069200 Sapur.008G080600 Sapur.008G081900<br>Sapur.008G090400 Sapur.008G093800 Sapur.008G094300 Sapur.008G096700 Sapur.008G104300 Sapur.008G119700<br>Sapur.008G121600 Sapur.008G124200 Sapur.008G134800 Sapur.008G160400 Sapur.009G005300 Sapur.009G009700<br>Sapur.009G011600 Sapur.009G021700 Sapur.009G040000 Sapur.009G094800 Sapur.009G097900 Sapur.009G104900<br>Sapur.009G112500 Sapur.009G112800 Sapur.009G115000 Sapur.009G126000 Sapur.009G126600 Sapur.010G003100<br>Sapur.010G005200 Sapur.010G013000 Sapur.010G040000 Sapur.010G040100 Sapur.010G046500 Sapur.010G065900<br>Sapur.010G068700 Sapur.010G086900 Sapur.010G093900 Sapur.010G096300 Sapur.010G099700 Sapur.010G100200<br>Sapur.010G113700 Sapur.010G114700 Sapur.010G123400 Sapur.010G128300 Sapur.010G134600 Sapur.010G149100<br>Sapur.010G151500 Sapur.010G154300 Sapur.010G161100 Sapur.011G006600 Sapur.011G008400 Sapur.011G014000<br>Sapur.011G032100 Sapur.011G035500 Sapur.011G037900 Sapur.011G038200 Sapur.011G039300 Sapur.011G041100<br>Sapur.011G042400 Sapur.011G045800 Sapur.011G049300 Sapur.011G054600 Sapur.011G061100 Sapur.011G064300

				Sapur.011G066500 Sapur.011G079300 Sapur.011G088400 Sapur.011G088500 Sapur.011G099500 Sapur.011G109600 Sapur.012G004000 Sapur.012G010400 Sapur.012G017700 Sapur.012G018600 Sapur.012G024500 Sapur.012G031400 Sapur.012G039100 Sapur.012G051400 Sapur.012G055400 Sapur.012G060100 Sapur.012G067900 Sapur.012G078700 Sapur.012G084400 Sapur.012G105100 Sapur.013G000400 Sapur.013G008000 Sapur.013G008600 Sapur.013G029500 Sapur.013G038800 Sapur.013G039500 Sapur.013G041800 Sapur.013G050700 Sapur.013G050900 Sapur.013G055500 Sapur.013G059000 Sapur.013G068800 Sapur.013G087600 Sapur.013G108500 Sapur.013G137400 Sapur.013G141800 Sapur.014G003700 Sapur.014G004000 Sapur.014G005200 Sapur.014G034500 Sapur.014G053000 Sapur.014G059000 Sapur.014G059100 Sapur.014G059200 Sapur.014G065300 Sapur.014G078300 Sapur.014G078500 Sapur.014G079300 Sapur.014G085400 Sapur.014G085500 Sapur.014G094500 Sapur.014G095700 Sapur.014G099500 Sapur.014G100000 Sapur.014G101000 Sapur.016G018700 Sapur.016G030200 Sapur.016G064600 Sapur.016G070400 Sapur.016G079900 Sapur.016G080200 Sapur.016G085100 Sapur.016G087700 Sapur.016G101200 Sapur.016G104000 Sapur.016G111900 Sapur.016G114700 Sapur.016G131300 Sapur.016G144900 Sapur.016G153200 Sapur.016G159000 Sapur.016G167800 Sapur.016G199700 Sapur.016G216900 Sapur.016G224700 Sapur.016G229800 Sapur.016G233500 Sapur.016G246900 Sapur.016G255700 Sapur.016G298800 Sapur.017G004800 Sapur.017G007100 Sapur.017G053300 Sapur.017G057100 Sapur.017G069900 Sapur.017G085900 Sapur.017G106300 Sapur.017G114800 Sapur.017G115700 Sapur.018G007100 Sapur.018G022500 Sapur.018G022700 Sapur.018G025000 Sapur.018G034800 Sapur.018G035300 Sapur.018G039600 Sapur.018G057700 Sapur.018G060900 Sapur.018G067800 Sapur.018G077400 Sapur.018G078200 Sapur.019G025000 Sapur.019G033200 Sapur.019G039900 Sapur.019G060100 Sapur.019G065000 Sapur.019G074500 Sapur.019G077800 Sapur.019G094000 Sapur.019G098000 Sapur.019G101700 Sapur.019G108100 Sapur.15WG060500 Sapur.15WG063000 Sapur.15WG065100 Sapur.15ZG002000 Sapur.15ZG007300 Sapur.15ZG016900 Sapur.15ZG053200 Sapur.15ZG067200 Sapur.15ZG071900 Sapur.15ZG079000 Sapur.15ZG079100 Sapur.15ZG080900 Sapur.15ZG085300 Sapur.15ZG103400 Sapur.T006300 Sapur.T012000 Sapur.003G056300 Sapur.002G029000 Sapur.001G057600 Sapur.002G194100 Sapur.003G002000 Sapur.003G116600 Sapur.003G168000 Sapur.006G136200 Sapur.007G112200 Sapur.008G063700 Sapur.010G009500 Sapur.010G137300 Sapur.010G163200 Sapur.014G098600 Sapur.017G020600
GO:0000819	9	17	sister chromatid segregation	Sapur.001G103000 Sapur.002G001600 Sapur.006G030400 Sapur.008G057000 Sapur.008G145300 Sapur.010G008600 Sapur.010G056400 Sapur.010G145400 Sapur.014G127000
GO:0007049	27	89	cell cycle	Sapur.001G103000 Sapur.002G001600 Sapur.002G070300 Sapur.004G071100 Sapur.005G135200 Sapur.006G004100 Sapur.006G030400 Sapur.006G065500 Sapur.006G194600 Sapur.007G123700 Sapur.008G057000 Sapur.008G121600 Sapur.008G145300 Sapur.009G003100 Sapur.010G008600 Sapur.010G056400 Sapur.010G075900 Sapur.010G145400 Sapur.010G183500 Sapur.012G020900 Sapur.012G099900 Sapur.014G127000 Sapur.016G087700 Sapur.017G006400 Sapur.017G006500 Sapur.15WG062900 Sapur.15ZG040500
GO:0006260	20	59	DNA replication	Sapur.001G055600 Sapur.001G058800 Sapur.003G068400 Sapur.003G070500 Sapur.004G103000 Sapur.005G069000 Sapur.007G071200 Sapur.010G053800 Sapur.010G193700 Sapur.011G056100 Sapur.011G101200

				Sapur.012G020900 Sapur.013G010700 Sapur.014G066300 Sapur.017G119700 Sapur.018G087600 Sapur.15WG072800 Sapur.15WG074800 Sapur.15ZG043500 Sapur.T047900
GO:0051726	16	44	regulation of cell cycle	Sapur.002G001600 Sapur.002G070300 Sapur.004G071100 Sapur.005G135200 Sapur.006G065500 Sapur.007G123700 Sapur.008G057000 Sapur.008G121600 Sapur.008G145300 Sapur.010G056400 Sapur.010G145400 Sapur.012G020900 Sapur.012G099900 Sapur.017G006400 Sapur.017G006500 Sapur.15WG062900
GO:0007346	8	15	regulation of mitotic cell cycle	Sapur.002G001600 Sapur.002G070300 Sapur.005G135200 Sapur.008G057000 Sapur.008G145300 Sapur.010G056400 Sapur.010G145400 Sapur.012G020900
GO:0051304	7	12	chromosome separation	Sapur.002G001600 Sapur.008G057000 Sapur.008G145300 Sapur.010G056400 Sapur.010G145400 Sapur.002G070300 Sapur.005G135200
GO:0042221	18	53	response to chemical	Sapur.001G072500 Sapur.002G043300 Sapur.004G054800 Sapur.005G163900 Sapur.006G063200 Sapur.006G104700 Sapur.006G114200 Sapur.009G009700 Sapur.011G041100 Sapur.014G078500 Sapur.016G085100 Sapur.016G118000 Sapur.016G229800 Sapur.017G115700 Sapur.15ZG103400 Sapur.T012000 Sapur.002G008900 Sapur.005G198700
GO:0007154	112	534	cell communication	Sapur.004G111400 Sapur.009G086200 Sapur.001G191500 Sapur.002G001600 Sapur.002G070300 Sapur.005G002500 Sapur.005G002800 Sapur.005G022300 Sapur.005G023100 Sapur.005G024800 Sapur.005G025000 Sapur.005G025400 Sapur.005G031700 Sapur.005G054000 Sapur.005G135200 Sapur.006G139900 Sapur.007G051700 Sapur.008G057000 Sapur.008G076200 Sapur.008G111900 Sapur.008G145300 Sapur.008G145600 Sapur.009G035200 Sapur.010G056400 Sapur.010G113700 Sapur.010G121400 Sapur.010G145400 Sapur.011G066500 Sapur.012G020900 Sapur.014G021100 Sapur.014G082100 Sapur.016G035600 Sapur.016G051900 Sapur.016G052700 Sapur.016G142200 Sapur.016G142300 Sapur.016G288500 Sapur.017G078300 Sapur.018G015000 Sapur.018G060400 Sapur.018G071800 Sapur.019G007100 Sapur.019G007800 Sapur.019G007900 Sapur.019G009300 Sapur.019G009600 Sapur.019G010100 Sapur.019G010500 Sapur.019G010700 Sapur.019G011000 Sapur.019G011200 Sapur.019G011500 Sapur.019G011600 Sapur.019G011700 Sapur.019G011900 Sapur.019G012100 Sapur.019G012500 Sapur.019G012700 Sapur.019G012800 Sapur.019G016400 Sapur.019G017000 Sapur.019G034300 Sapur.019G042100 Sapur.019G042400 Sapur.019G042500 Sapur.019G043100 Sapur.019G043200 Sapur.019G044100 Sapur.019G053500 Sapur.019G091100 Sapur.019G091300 Sapur.019G091700 Sapur.019G092000 Sapur.019G092300 Sapur.019G092600 Sapur.019G092700 Sapur.019G092800 Sapur.019G093000 Sapur.019G093400 Sapur.019G093600 Sapur.019G093900 Sapur.019G094300 Sapur.019G110900 Sapur.15WG043400 Sapur.15WG060500 Sapur.15ZG009400 Sapur.15ZG050400 Sapur.15ZG050500 Sapur.T116500 Sapur.T150000 Sapur.T170000 Sapur.T175100 Sapur.006G141000 Sapur.009G010000 Sapur.010G175000 Sapur.001G041100 Sapur.001G121200 Sapur.001G162000 Sapur.006G030100 Sapur.006G216200 Sapur.008G146500 Sapur.008G155300 Sapur.008G158900 Sapur.010G018800 Sapur.012G002400 Sapur.013G028600 Sapur.014G083600 Sapur.016G034100 Sapur.018G017300 Sapur.018G017400 Sapur.018G036400 Sapur.15WG079800

GO:0005984	9	19	disaccharide metabolic process	Sapur.002G158700 Sapur.004G057100 Sapur.013G092100 Sapur.017G046200 Sapur.017G114500 Sapur.018G021500 Sapur.15ZG022300 Sapur.T013300 Sapur.003G106500
GO:0045786	8	16	negative regulation of cell cycle	Sapur.002G001600 Sapur.002G070300 Sapur.005G135200 Sapur.008G057000 Sapur.008G145300 Sapur.010G056400 Sapur.010G145400 Sapur.012G020900
GO:0010948	8	16	negative regulation of cell cycle process	Sapur.002G001600 Sapur.002G070300 Sapur.005G135200 Sapur.008G057000 Sapur.008G145300 Sapur.010G056400 Sapur.010G145400 Sapur.012G020900
GO:0000075	8	16	cell cycle checkpoint signaling	Sapur.002G001600 Sapur.002G070300 Sapur.005G135200 Sapur.008G057000 Sapur.008G145300 Sapur.010G056400 Sapur.010G145400 Sapur.012G020900
GO:0005985	8	16	sucrose metabolic process	Sapur.002G158700 Sapur.004G057100 Sapur.013G092100 Sapur.017G046200 Sapur.017G114500 Sapur.018G021500 Sapur.15ZG022300 Sapur.T013300
GO:1901988	8	16	negative regulation of cell cycle phase transition	Sapur.002G001600 Sapur.002G070300 Sapur.005G135200 Sapur.008G057000 Sapur.008G145300 Sapur.010G056400 Sapur.010G145400 Sapur.012G020900
GO:0055085	138	681	transmembrane transport	Sapur.001G006200 Sapur.001G008300 Sapur.001G054900 Sapur.001G078400 Sapur.001G090700 Sapur.001G094500 Sapur.001G102400 Sapur.001G120100 Sapur.001G126500 Sapur.001G137400 Sapur.002G004100 Sapur.002G043400 Sapur.002G086100 Sapur.002G132500 Sapur.002G156100 Sapur.003G025000 Sapur.003G069300 Sapur.003G073700 Sapur.003G081500 Sapur.003G098800 Sapur.003G099500 Sapur.003G138500 Sapur.003G152900 Sapur.004G009600 Sapur.004G009700 Sapur.004G134700 Sapur.004G138100 Sapur.004G157800 Sapur.005G009200 Sapur.005G061600 Sapur.005G082300 Sapur.005G114300 Sapur.005G122600 Sapur.005G131700 Sapur.005G163600 Sapur.005G163800 Sapur.005G197500 Sapur.005G204900 Sapur.006G010900 Sapur.006G026500 Sapur.006G028100 Sapur.006G042200 Sapur.006G042300 Sapur.006G079600 Sapur.006G091800 Sapur.006G154500 Sapur.006G206900 Sapur.007G081600 Sapur.007G094100 Sapur.008G065700 Sapur.008G066200 Sapur.008G101500 Sapur.008G101600 Sapur.008G101700 Sapur.008G118300 Sapur.008G141100 Sapur.008G163400 Sapur.009G034100 Sapur.009G034500 Sapur.009G040500 Sapur.009G045700 Sapur.009G057300 Sapur.009G057800 Sapur.009G063500 Sapur.009G089800 Sapur.009G097300 Sapur.010G001900 Sapur.010G024600 Sapur.010G040800 Sapur.010G077300 Sapur.010G089300 Sapur.010G133500 Sapur.010G194900 Sapur.011G001000 Sapur.011G028400 Sapur.011G047400 Sapur.011G080900 Sapur.011G096900 Sapur.012G000800 Sapur.012G005500 Sapur.012G006100 Sapur.012G007900 Sapur.012G017000 Sapur.012G025300 Sapur.012G028700 Sapur.012G035400 Sapur.012G051100 Sapur.012G073300 Sapur.012G100800

				Sapur.012G102500 Sapur.012G111400 Sapur.013G028200 Sapur.013G059700 Sapur.013G064500 Sapur.013G071900 Sapur.013G072500 Sapur.013G072600 Sapur.013G072700 Sapur.014G057600 Sapur.014G090200 Sapur.014G090300 Sapur.014G108300 Sapur.014G128800 Sapur.016G004300 Sapur.016G005900 Sapur.016G031200 Sapur.016G092200 Sapur.016G106100 Sapur.016G153700 Sapur.016G225300 Sapur.016G230700 Sapur.016G230800 Sapur.016G266000 Sapur.017G057900 Sapur.017G059600 Sapur.017G064600 Sapur.017G069400 Sapur.017G101500 Sapur.018G026000 Sapur.018G063900 Sapur.018G088100 Sapur.018G094900 Sapur.018G103300 Sapur.019G039500 Sapur.019G046000 Sapur.019G064700 Sapur.019G076400 Sapur.019G083100 Sapur.019G113800 Sapur.15ZG018400 Sapur.15ZG026500 Sapur.15ZG078400 Sapur.15ZG126400 Sapur.T005400 Sapur.T005500 Sapur.006G076500 Sapur.009G028800 Sapur.009G028900
GO:0006520	39	153	amino acid metabolic process	Sapur.001G057600 Sapur.002G085400 Sapur.002G161200 Sapur.003G092400 Sapur.004G122800 Sapur.005G058900 Sapur.006G027800 Sapur.006G049700 Sapur.007G002900 Sapur.007G115600 Sapur.008G002300 Sapur.009G056800 Sapur.009G112500 Sapur.010G093900 Sapur.010G121200 Sapur.010G193600 Sapur.011G008400 Sapur.013G054200 Sapur.014G098600 Sapur.016G228700 Sapur.016G231000 Sapur.017G108800 Sapur.019G070000 Sapur.15ZG101500 Sapur.001G180200 Sapur.008G054900 Sapur.011G120500 Sapur.016G153900 Sapur.002G194100 Sapur.003G002000 Sapur.003G168000 Sapur.006G136200 Sapur.007G112200 Sapur.010G009500 Sapur.010G163200 Sapur.017G020600 Sapur.004G028700 Sapur.004G108600 Sapur.011G036500
GO:0023052	110	528	signaling	Sapur.004G111400 Sapur.009G086200 Sapur.001G191500 Sapur.002G001600 Sapur.002G070300 Sapur.005G002500 Sapur.005G002800 Sapur.005G022300 Sapur.005G023100 Sapur.005G024800 Sapur.005G025000 Sapur.005G025400 Sapur.005G031700 Sapur.005G054000 Sapur.005G135200 Sapur.006G139900 Sapur.007G051700 Sapur.008G057000 Sapur.008G076200 Sapur.008G111900 Sapur.008G145300 Sapur.008G145600 Sapur.009G035200 Sapur.010G056400 Sapur.010G113700 Sapur.010G121400 Sapur.010G145400 Sapur.011G066500 Sapur.012G020900 Sapur.014G021100 Sapur.014G082100 Sapur.016G035600 Sapur.016G051900 Sapur.016G052700 Sapur.016G142200 Sapur.016G142300 Sapur.016G288500 Sapur.017G078300 Sapur.018G015000 Sapur.018G060400 Sapur.018G071800 Sapur.019G007100 Sapur.019G007800 Sapur.019G007900 Sapur.019G009300 Sapur.019G009600 Sapur.019G010100 Sapur.019G010500 Sapur.019G010700 Sapur.019G011000 Sapur.019G011200 Sapur.019G011500 Sapur.019G011600 Sapur.019G011700 Sapur.019G011900 Sapur.019G012100 Sapur.019G012500 Sapur.019G012700 Sapur.019G012800 Sapur.019G016400 Sapur.019G017000 Sapur.019G034300 Sapur.019G042100 Sapur.019G042400 Sapur.019G042500 Sapur.019G043100 Sapur.019G043200 Sapur.019G044100 Sapur.019G053500 Sapur.019G091100 Sapur.019G091300 Sapur.019G091700 Sapur.019G092000 Sapur.019G092300 Sapur.019G092600 Sapur.019G092700 Sapur.019G092800 Sapur.019G093000 Sapur.019G093400 Sapur.019G093600 Sapur.019G093900 Sapur.019G094300 Sapur.019G110900 Sapur.15WG043400 Sapur.15WG060500 Sapur.15ZG009400 Sapur.15ZG050400 Sapur.15ZG050500 Sapur.T116500 Sapur.T150000 Sapur.T170000 Sapur.T175100 Sapur.006G141000 Sapur.001G041100 Sapur.001G121200 Sapur.001G162000 Sapur.006G030100 Sapur.006G216200 Sapur.008G146500 Sapur.008G155300 Sapur.008G158900

				Sapur.010G018800 Sapur.012G002400 Sapur.013G028600 Sapur.014G083600 Sapur.016G034100 Sapur.018G017300 Sapur.018G017400 Sapur.018G036400 Sapur.15WG079800
GO:0007165	110	528	signal transduction	Sapur.004G111400 Sapur.009G086200 Sapur.001G191500 Sapur.002G001600 Sapur.002G070300 Sapur.005G002500 Sapur.005G002800 Sapur.005G022300 Sapur.005G023100 Sapur.005G024800 Sapur.005G025000 Sapur.005G025400 Sapur.005G031700 Sapur.005G054000 Sapur.005G135200 Sapur.006G139900 Sapur.007G051700 Sapur.008G057000 Sapur.008G076200 Sapur.008G111900 Sapur.008G145300 Sapur.008G145600 Sapur.009G035200 Sapur.010G056400 Sapur.010G113700 Sapur.010G121400 Sapur.010G145400 Sapur.011G066500 Sapur.012G020900 Sapur.014G021100 Sapur.014G082100 Sapur.016G035600 Sapur.016G051900 Sapur.016G052700 Sapur.016G142200 Sapur.016G142300 Sapur.016G288500 Sapur.017G078300 Sapur.018G015000 Sapur.018G060400 Sapur.018G071800 Sapur.019G007100 Sapur.019G007800 Sapur.019G007900 Sapur.019G009300 Sapur.019G009600 Sapur.019G010100 Sapur.019G010500 Sapur.019G010700 Sapur.019G011000 Sapur.019G011200 Sapur.019G011500 Sapur.019G011600 Sapur.019G011700 Sapur.019G011900 Sapur.019G012100 Sapur.019G012500 Sapur.019G012700 Sapur.019G012800 Sapur.019G016400 Sapur.019G017000 Sapur.019G034300 Sapur.019G042100 Sapur.019G042400 Sapur.019G042500 Sapur.019G043100 Sapur.019G043200 Sapur.019G044100 Sapur.019G053500 Sapur.019G091100 Sapur.019G091300 Sapur.019G091700 Sapur.019G092000 Sapur.019G092300 Sapur.019G092600 Sapur.019G092700 Sapur.019G092800 Sapur.019G093000 Sapur.019G093400 Sapur.019G093600 Sapur.019G093900 Sapur.019G094300 Sapur.019G110900 Sapur.15WG043400 Sapur.15WG060500 Sapur.15ZG009400 Sapur.15ZG050400 Sapur.15ZG050500 Sapur.T116500 Sapur.T150000 Sapur.T170000 Sapur.T175100 Sapur.006G141000 Sapur.001G041100 Sapur.001G121200 Sapur.001G162000 Sapur.006G030100 Sapur.006G216200 Sapur.008G146500 Sapur.008G155300 Sapur.008G158900 Sapur.010G018800 Sapur.012G002400 Sapur.013G028600 Sapur.014G083600 Sapur.016G034100 Sapur.018G017300 Sapur.018G017400 Sapur.018G036400 Sapur.15WG079800
GO:0001934	6	10	positive regulation of protein phosphorylation	Sapur.004G071100 Sapur.007G123700 Sapur.012G099900 Sapur.017G006400 Sapur.017G006500 Sapur.15WG062900
GO:0032147	6	10	activation of protein kinase activity	Sapur.004G071100 Sapur.007G123700 Sapur.012G099900 Sapur.017G006400 Sapur.017G006500 Sapur.15WG062900
GO:0090224	6	10	regulation of spindle organization	Sapur.004G071100 Sapur.007G123700 Sapur.012G099900 Sapur.017G006400 Sapur.017G006500 Sapur.15WG062900
GO:0045937	6	10	positive regulation of	Sapur.004G071100 Sapur.007G123700 Sapur.012G099900 Sapur.017G006400 Sapur.017G006500 Sapur.15WG062900

			phosphate metabolic process	
GO:0042327	6	10	positive regulation of phosphorylation	Sapur.004G071100 Sapur.007G123700 Sapur.012G099900 Sapur.017G006400 Sapur.017G006500 Sapur.15WG062900
GO:0033674	6	10	positive regulation of kinase activity	Sapur.004G071100 Sapur.007G123700 Sapur.012G099900 Sapur.017G006400 Sapur.017G006500 Sapur.15WG062900
GO:0060236	6	10	regulation of mitotic spindle organization	Sapur.004G071100 Sapur.007G123700 Sapur.012G099900 Sapur.017G006400 Sapur.017G006500 Sapur.15WG062900
GO:0031401	6	10	positive regulation of protein modification process	Sapur.004G071100 Sapur.007G123700 Sapur.012G099900 Sapur.017G006400 Sapur.017G006500 Sapur.15WG062900
GO:0045860	6	10	positive regulation of protein kinase activity	Sapur.004G071100 Sapur.007G123700 Sapur.012G099900 Sapur.017G006400 Sapur.017G006500 Sapur.15WG062900
GO:0051347	6	10	positive regulation of transferase activity	Sapur.004G071100 Sapur.007G123700 Sapur.012G099900 Sapur.017G006400 Sapur.017G006500 Sapur.15WG062900
GO:1902850	6	10	microtubule cytoskeleton organization involved in mitosis	Sapur.004G071100 Sapur.007G123700 Sapur.012G099900 Sapur.017G006400 Sapur.017G006500 Sapur.15WG062900
GO:0010562	6	10	positive regulation of phosphorus metabolic process	Sapur.004G071100 Sapur.007G123700 Sapur.012G099900 Sapur.017G006400 Sapur.017G006500 Sapur.15WG062900

GO:0007052	6	10	mitotic spindle organization	Sapur.004G071100 Sapur.007G123700 Sapur.012G099900 Sapur.017G006400 Sapur.017G006500 Sapur.15WG062900
GO:0009311	9	20	oligosaccharide metabolic process	Sapur.002G158700 Sapur.004G057100 Sapur.013G092100 Sapur.017G046200 Sapur.017G114500 Sapur.018G021500 Sapur.15ZG022300 Sapur.T013300 Sapur.003G106500
GO:0034641	469	2625	cellular nitrogen compound metabolic process	Sapur.007G110800 Sapur.013G054700 Sapur.017G027400 Sapur.001G017300 Sapur.001G025400 Sapur.001G027900 Sapur.001G028100 Sapur.001G028400 Sapur.001G034200 Sapur.001G055600 Sapur.001G058800 Sapur.001G064300 Sapur.001G067700 Sapur.001G069800 Sapur.001G072500 Sapur.001G075700 Sapur.001G090900 Sapur.001G108900 Sapur.001G119600 Sapur.001G129000 Sapur.001G130000 Sapur.001G149200 Sapur.001G163400 Sapur.001G163900 Sapur.001G164600 Sapur.001G167800 Sapur.001G179800 Sapur.001G183500 Sapur.001G183600 Sapur.002G012900 Sapur.002G023100 Sapur.002G024200 Sapur.002G028500 Sapur.002G030000 Sapur.002G030100 Sapur.002G033200 Sapur.002G041000 Sapur.002G043300 Sapur.002G048900 Sapur.002G064600 Sapur.002G069500 Sapur.002G073000 Sapur.002G080700 Sapur.002G091700 Sapur.002G110600 Sapur.002G112100 Sapur.002G115400 Sapur.002G125900 Sapur.002G126000 Sapur.002G128100 Sapur.002G136900 Sapur.002G142300 Sapur.002G143500 Sapur.002G146000 Sapur.002G151100 Sapur.002G161200 Sapur.002G163600 Sapur.002G175800 Sapur.002G190900 Sapur.003G016800 Sapur.003G017600 Sapur.003G026500 Sapur.003G047900 Sapur.003G049200 Sapur.003G054700 Sapur.003G057500 Sapur.003G067100 Sapur.003G068400 Sapur.003G070500 Sapur.003G091600 Sapur.003G092400 Sapur.003G099900 Sapur.003G102000 Sapur.003G111600 Sapur.003G117300 Sapur.003G123800 Sapur.003G125900 Sapur.003G138200 Sapur.003G145700 Sapur.003G150800 Sapur.003G152800 Sapur.003G155000 Sapur.004G002900 Sapur.004G007000 Sapur.004G008500 Sapur.004G027800 Sapur.004G030500 Sapur.004G030800 Sapur.004G031600 Sapur.004G054800 Sapur.004G056900 Sapur.004G078500 Sapur.004G102300 Sapur.004G103000 Sapur.004G118800 Sapur.004G121500 Sapur.004G122800 Sapur.004G123100 Sapur.004G131600 Sapur.004G137000 Sapur.004G171400 Sapur.004G176300 Sapur.005G005600 Sapur.005G042000 Sapur.005G045400 Sapur.005G046200 Sapur.005G048400 Sapur.005G050200 Sapur.005G052900 Sapur.005G054800 Sapur.005G055100 Sapur.005G067000 Sapur.005G069000 Sapur.005G082000 Sapur.005G087400 Sapur.005G098100 Sapur.005G105500 Sapur.005G108500 Sapur.005G119200 Sapur.005G133200 Sapur.005G141300 Sapur.005G150000 Sapur.005G152800 Sapur.005G163900 Sapur.006G025100 Sapur.006G027800 Sapur.006G033400 Sapur.006G041100 Sapur.006G043000 Sapur.006G063200 Sapur.006G067500 Sapur.006G068600 Sapur.006G070000 Sapur.006G083400 Sapur.006G089400 Sapur.006G092800 Sapur.006G103400 Sapur.006G104700 Sapur.006G110100 Sapur.006G114200 Sapur.006G118700 Sapur.006G120000 Sapur.006G120300 Sapur.006G121100 Sapur.006G122700 Sapur.006G137200 Sapur.006G154200 Sapur.006G166000 Sapur.006G168000 Sapur.006G194100 Sapur.006G195200 Sapur.006G197300 Sapur.006G214400 Sapur.006G218000 Sapur.006G228700 Sapur.006G228900 Sapur.007G001300 Sapur.007G002900 Sapur.007G005500 Sapur.007G021900 Sapur.007G034000 Sapur.007G035800 Sapur.007G044500 Sapur.007G044600 Sapur.007G050900 Sapur.007G058700 Sapur.007G062200 Sapur.007G071200 Sapur.007G082500 Sapur.007G087500 Sapur.007G087800 Sapur.007G089800 Sapur.007G094000

Sapur.007G114800 Sapur.007G115600 Sapur.007G123400 Sapur.008G034200 Sapur.008G042700 Sapur.008G044200
Sapur.008G053100 Sapur.008G060200 Sapur.008G060600 Sapur.008G064800 Sapur.008G069200 Sapur.008G080600
Sapur.008G081900 Sapur.008G090400 Sapur.008G093800 Sapur.008G094300 Sapur.008G096700 Sapur.008G098100
Sapur.008G102800 Sapur.008G104300 Sapur.008G119700 Sapur.008G121600 Sapur.008G124200 Sapur.008G128300
Sapur.008G134800 Sapur.008G160400 Sapur.008G162200 Sapur.008G168800 Sapur.009G002400 Sapur.009G002900
Sapur.009G005300 Sapur.009G009700 Sapur.009G011600 Sapur.009G021700 Sapur.009G040000 Sapur.009G045200
Sapur.009G094800 Sapur.009G097900 Sapur.009G099800 Sapur.009G104900 Sapur.009G112500 Sapur.009G112800
Sapur.009G115000 Sapur.009G126000 Sapur.009G126600 Sapur.010G003100 Sapur.010G005200 Sapur.010G011800
Sapur.010G013000 Sapur.010G040000 Sapur.010G040100 Sapur.010G046500 Sapur.010G046700 Sapur.010G053800
Sapur.010G065900 Sapur.010G068700 Sapur.010G086900 Sapur.010G087900 Sapur.010G093900 Sapur.010G096300
Sapur.010G099700 Sapur.010G100200 Sapur.010G113700 Sapur.010G114700 Sapur.010G123400 Sapur.010G128300
Sapur.010G134600 Sapur.010G136100 Sapur.010G141100 Sapur.010G149100 Sapur.010G151500 Sapur.010G154300
Sapur.010G161100 Sapur.010G169600 Sapur.010G193700 Sapur.011G006600 Sapur.011G008400 Sapur.011G014000
Sapur.011G032100 Sapur.011G035500 Sapur.011G037900 Sapur.011G038200 Sapur.011G039300 Sapur.011G041100
Sapur.011G042400 Sapur.011G045800 Sapur.011G049300 Sapur.011G054600 Sapur.011G056100 Sapur.011G061100
Sapur.011G064300 Sapur.011G066500 Sapur.011G079300 Sapur.011G088400 Sapur.011G088500 Sapur.011G099500
Sapur.011G101200 Sapur.011G109600 Sapur.012G000700 Sapur.012G004000 Sapur.012G010400 Sapur.012G017700
Sapur.012G018600 Sapur.012G020900 Sapur.012G024200 Sapur.012G024500 Sapur.012G031400 Sapur.012G039100
Sapur.012G051400 Sapur.012G055400 Sapur.012G060100 Sapur.012G067900 Sapur.012G078700 Sapur.012G084400
Sapur.012G105100 Sapur.013G000400 Sapur.013G008000 Sapur.013G008600 Sapur.013G010700 Sapur.013G029500
Sapur.013G038800 Sapur.013G039500 Sapur.013G041800 Sapur.013G050700 Sapur.013G050900 Sapur.013G055500
Sapur.013G059000 Sapur.013G067900 Sapur.013G068800 Sapur.013G082200 Sapur.013G087600 Sapur.013G103900
Sapur.013G108500 Sapur.013G126600 Sapur.013G128900 Sapur.013G135300 Sapur.013G137400 Sapur.013G138200
Sapur.013G141800 Sapur.014G003700 Sapur.014G004000 Sapur.014G005200 Sapur.014G034500 Sapur.014G053000
Sapur.014G059000 Sapur.014G059100 Sapur.014G059200 Sapur.014G065300 Sapur.014G066300 Sapur.014G078300
Sapur.014G078500 Sapur.014G079300 Sapur.014G085400 Sapur.014G085500 Sapur.014G094500 Sapur.014G095700
Sapur.014G099500 Sapur.014G100000 Sapur.014G101000 Sapur.014G104700 Sapur.014G106600 Sapur.014G112900
Sapur.014G117700 Sapur.014G138900 Sapur.014G142200 Sapur.016G018700 Sapur.016G030200 Sapur.016G039500
Sapur.016G058600 Sapur.016G064600 Sapur.016G070400 Sapur.016G079900 Sapur.016G080200 Sapur.016G085100
Sapur.016G087700 Sapur.016G101200 Sapur.016G104000 Sapur.016G111900 Sapur.016G114700 Sapur.016G131300
Sapur.016G144900 Sapur.016G151000 Sapur.016G152700 Sapur.016G153200 Sapur.016G159000 Sapur.016G167800
Sapur.016G195300 Sapur.016G199700 Sapur.016G205800 Sapur.016G216900 Sapur.016G224700 Sapur.016G225800
Sapur.016G229800 Sapur.016G233500 Sapur.016G246900 Sapur.016G255700 Sapur.016G298800 Sapur.016G310400

				Sapur.017G004800 Sapur.017G007100 Sapur.017G053300 Sapur.017G057100 Sapur.017G069900 Sapur.017G085900 Sapur.017G106300 Sapur.017G114800 Sapur.017G115700 Sapur.017G119700 Sapur.018G007100 Sapur.018G022500 Sapur.018G022700 Sapur.018G025000 Sapur.018G034800 Sapur.018G035300 Sapur.018G039600 Sapur.018G045400 Sapur.018G047900 Sapur.018G057700 Sapur.018G060900 Sapur.018G064600 Sapur.018G067800 Sapur.018G077400 Sapur.018G078200 Sapur.018G087600 Sapur.018G090600 Sapur.018G103000 Sapur.018G114600 Sapur.018G115000 Sapur.019G025000 Sapur.019G033200 Sapur.019G039400 Sapur.019G039900 Sapur.019G060100 Sapur.019G065000 Sapur.019G074500 Sapur.019G075900 Sapur.019G077800 Sapur.019G094000 Sapur.019G098000 Sapur.019G101700 Sapur.019G108100 Sapur.15WG032400 Sapur.15WG060500 Sapur.15WG063000 Sapur.15WG065100 Sapur.15WG072800 Sapur.15WG074800 Sapur.15ZG002000 Sapur.15ZG007300 Sapur.15ZG016900 Sapur.15ZG020500 Sapur.15ZG036200 Sapur.15ZG043500 Sapur.15ZG053200 Sapur.15ZG067200 Sapur.15ZG071900 Sapur.15ZG079000 Sapur.15ZG079100 Sapur.15ZG080300 Sapur.15ZG080900 Sapur.15ZG085300 Sapur.15ZG103400 Sapur.T006300 Sapur.T012000 Sapur.003G056300 Sapur.001G151600 Sapur.002G029000 Sapur.004G156500 Sapur.006G022000 Sapur.016G026400 Sapur.018G088200 Sapur.15ZG007500 Sapur.15ZG120200 Sapur.T047900 Sapur.002G047600 Sapur.004G123200 Sapur.008G019800 Sapur.008G054900 Sapur.009G099900 Sapur.013G094800 Sapur.013G094900 Sapur.013G095000 Sapur.013G095100 Sapur.001G057600 Sapur.002G194100 Sapur.003G002000 Sapur.003G116600 Sapur.003G168000 Sapur.006G136200 Sapur.007G112200 Sapur.008G063700 Sapur.010G009500 Sapur.010G137300 Sapur.010G163200 Sapur.014G098600 Sapur.017G020600
GO:0006020	4	5	inositol metabolic process	Sapur.005G061300 Sapur.007G081700 Sapur.008G116400 Sapur.018G050900
GO:0030261	4	5	chromosome condensation	Sapur.001G103000 Sapur.006G030400 Sapur.010G008600 Sapur.014G127000
GO:0007076	4	5	mitotic chromosome condensation	Sapur.001G103000 Sapur.006G030400 Sapur.010G008600 Sapur.014G127000
GO:0007017	45	187	microtubule-based process	Sapur.001G094200 Sapur.002G064800 Sapur.002G085200 Sapur.002G089300 Sapur.004G019100 Sapur.004G071100 Sapur.004G109700 Sapur.004G147000 Sapur.005G022300 Sapur.006G036800 Sapur.006G065500 Sapur.006G067400 Sapur.006G069500 Sapur.006G112500 Sapur.006G172300 Sapur.007G123700 Sapur.008G137200 Sapur.009G029500 Sapur.009G122200 Sapur.010G042700 Sapur.010G081900 Sapur.010G119100 Sapur.011G018500 Sapur.011G103700 Sapur.012G037400 Sapur.012G040700 Sapur.012G099300 Sapur.012G099900 Sapur.014G005100 Sapur.014G051500 Sapur.014G052300 Sapur.016G071200 Sapur.016G259400 Sapur.017G006400 Sapur.017G006500 Sapur.017G067300 Sapur.018G057600 Sapur.15WG040400 Sapur.15WG044400 Sapur.15WG062900 Sapur.15ZG044100 Sapur.15ZG044300 Sapur.15ZG044900 Sapur.15ZG049500 Sapur.T003900
GO:004	6	11	positive	Sapur.004G071100 Sapur.007G123700 Sapur.012G099900 Sapur.017G006400 Sapur.017G006500

3085			regulation of catalytic activity	Sapur.15WG062900
GO:0032886	6	11	regulation of microtubule-based process	Sapur.004G071100 Sapur.007G123700 Sapur.012G099900 Sapur.017G006400 Sapur.017G006500 Sapur.15WG062900
GO:0070507	6	11	regulation of microtubule cytoskeleton organization	Sapur.004G071100 Sapur.007G123700 Sapur.012G099900 Sapur.017G006400 Sapur.017G006500 Sapur.15WG062900
GO:1901991	6	11	negative regulation of mitotic cell cycle phase transition	Sapur.002G001600 Sapur.008G057000 Sapur.008G145300 Sapur.010G056400 Sapur.010G145400 Sapur.012G020900
GO:0044093	6	11	positive regulation of molecular function	Sapur.004G071100 Sapur.007G123700 Sapur.012G099900 Sapur.017G006400 Sapur.017G006500 Sapur.15WG062900
GO:0009314	5	8	response to radiation	Sapur.009G112000 Sapur.012G020900 Sapur.010G113700 Sapur.013G000400 Sapur.15WG060500
GO:0044770	8	18	cell cycle phase transition	Sapur.002G001600 Sapur.002G070300 Sapur.005G135200 Sapur.008G057000 Sapur.008G145300 Sapur.010G056400 Sapur.010G145400 Sapur.012G020900
GO:1901987	8	18	regulation of cell cycle phase transition	Sapur.002G001600 Sapur.002G070300 Sapur.005G135200 Sapur.008G057000 Sapur.008G145300 Sapur.010G056400 Sapur.010G145400 Sapur.012G020900
GO:0030001	47	202	metal ion transport	Sapur.001G054900 Sapur.001G081300 Sapur.001G137400 Sapur.002G003300 Sapur.002G150200 Sapur.003G090400 Sapur.004G051000 Sapur.004G162100 Sapur.005G009200 Sapur.005G019400 Sapur.005G028900 Sapur.005G119500 Sapur.005G122600 Sapur.006G004800 Sapur.006G014400 Sapur.006G084800 Sapur.006G174900 Sapur.006G181200 Sapur.007G080400 Sapur.008G065700 Sapur.008G163400 Sapur.009G057300 Sapur.009G057800 Sapur.009G107600 Sapur.010G004000 Sapur.010G015700 Sapur.010G086800 Sapur.010G142500 Sapur.012G003700 Sapur.013G018100 Sapur.016G074300 Sapur.016G087600 Sapur.016G107800 Sapur.016G194200 Sapur.016G225300 Sapur.016G291800 Sapur.017G059600 Sapur.017G120700 Sapur.017G121300 Sapur.017G121600 Sapur.017G121700

GO:0048285	11	31	organelle fission	Sapur.017G122100 Sapur.15ZG002800 Sapur.009G063500 Sapur.006G076500 Sapur.009G028800 Sapur.009G028900 Sapur.001G103000 Sapur.002G001600 Sapur.006G030400 Sapur.008G057000 Sapur.008G145300 Sapur.009G003100 Sapur.010G008600 Sapur.010G056400 Sapur.010G145400 Sapur.010G183500 Sapur.014G127000 Sapur.004G111400 Sapur.009G086200 Sapur.010G075900 Sapur.001G063400 Sapur.001G072500
GO:0050896	189	1000	response to stimulus	Sapur.001G191500 Sapur.002G001600 Sapur.002G043300 Sapur.002G070300 Sapur.002G146500 Sapur.003G090600 Sapur.003G090700 Sapur.004G012300 Sapur.004G012400 Sapur.004G027800 Sapur.004G034700 Sapur.004G034800 Sapur.004G054800 Sapur.005G002500 Sapur.005G002800 Sapur.005G022300 Sapur.005G023100 Sapur.005G024800 Sapur.005G025000 Sapur.005G025400 Sapur.005G031700 Sapur.005G045400 Sapur.005G054000 Sapur.005G055700 Sapur.005G089700 Sapur.005G095300 Sapur.005G115200 Sapur.005G135200 Sapur.005G163900 Sapur.006G025100 Sapur.006G063200 Sapur.006G104700 Sapur.006G114200 Sapur.006G139900 Sapur.006G219200 Sapur.007G049700 Sapur.007G051700 Sapur.007G108700 Sapur.008G057000 Sapur.008G076200 Sapur.008G111900 Sapur.008G128300 Sapur.008G145300 Sapur.008G145600 Sapur.009G002900 Sapur.009G009600 Sapur.009G009700 Sapur.009G035200 Sapur.009G112000 Sapur.010G046700 Sapur.010G056400 Sapur.010G065900 Sapur.010G084000 Sapur.010G113700 Sapur.010G121400 Sapur.010G136100 Sapur.010G145400 Sapur.010G150000 Sapur.010G193700 Sapur.011G015200 Sapur.011G041100 Sapur.011G066500 Sapur.011G077900 Sapur.011G086400 Sapur.011G086500 Sapur.012G000700 Sapur.012G020900 Sapur.012G024200 Sapur.013G121200 Sapur.013G135300 Sapur.013G140300 Sapur.014G021100 Sapur.014G074100 Sapur.014G078500 Sapur.014G082100 Sapur.014G104700 Sapur.014G106600 Sapur.014G117700 Sapur.014G142200 Sapur.016G035600 Sapur.016G039500 Sapur.016G051900 Sapur.016G052700 Sapur.016G055400 Sapur.016G085100 Sapur.016G118000 Sapur.016G142200 Sapur.016G142300 Sapur.016G151000 Sapur.016G195300 Sapur.016G229800 Sapur.016G263600 Sapur.016G288500 Sapur.017G028400 Sapur.017G028500 Sapur.017G078300 Sapur.017G115700 Sapur.018G015000 Sapur.018G060400 Sapur.018G071800 Sapur.019G007100 Sapur.019G007800 Sapur.019G007900 Sapur.019G009300 Sapur.019G009600 Sapur.019G010100 Sapur.019G010500 Sapur.019G010700 Sapur.019G011000 Sapur.019G011200 Sapur.019G011500 Sapur.019G011600 Sapur.019G011700 Sapur.019G011900 Sapur.019G012100 Sapur.019G012500 Sapur.019G012700 Sapur.019G012800 Sapur.019G016400 Sapur.019G017000 Sapur.019G034300 Sapur.019G042100 Sapur.019G042400 Sapur.019G042500 Sapur.019G043100 Sapur.019G043200 Sapur.019G044100 Sapur.019G053500 Sapur.019G075900 Sapur.019G091100 Sapur.019G091300 Sapur.019G091700 Sapur.019G092000 Sapur.019G092300 Sapur.019G092600 Sapur.019G092700 Sapur.019G092800 Sapur.019G093000 Sapur.019G093400 Sapur.019G093600 Sapur.019G093900 Sapur.019G094300 Sapur.019G110900 Sapur.15WG032400 Sapur.15WG043400 Sapur.15WG060500 Sapur.15ZG002400 Sapur.15ZG002500 Sapur.15ZG009400 Sapur.15ZG020500 Sapur.15ZG036200 Sapur.15ZG050400 Sapur.15ZG050500 Sapur.15ZG103400 Sapur.15ZG111600 Sapur.15ZG127500 Sapur.T012000 Sapur.T116500 Sapur.T150000 Sapur.T170000 Sapur.T175100 Sapur.006G141000 Sapur.009G010000 Sapur.010G175000 Sapur.002G008900 Sapur.005G198700 Sapur.013G000400 Sapur.001G041100 Sapur.001G121200 Sapur.001G162000 Sapur.006G030100 Sapur.006G216200 Sapur.008G146500 Sapur.008G155300

				Sapur.008G158900 Sapur.010G018800 Sapur.012G002400 Sapur.013G028600 Sapur.014G083600 Sapur.016G034100 Sapur.018G017300 Sapur.018G017400 Sapur.018G036400 Sapur.15WG079800
GO:0006399	26	101	tRNA metabolic process	Sapur.001G163900 Sapur.002G161200 Sapur.005G046200 Sapur.005G050200 Sapur.007G002900 Sapur.007G044500 Sapur.007G115600 Sapur.008G098100 Sapur.008G168800 Sapur.009G099800 Sapur.009G112500 Sapur.010G011800 Sapur.010G093900 Sapur.011G008400 Sapur.014G112900 Sapur.016G152700 Sapur.016G310400 Sapur.018G090600 Sapur.002G194100 Sapur.003G002000 Sapur.003G168000 Sapur.006G136200 Sapur.007G112200 Sapur.010G009500 Sapur.010G163200 Sapur.017G020600
GO:0044772	6	13	mitotic cell cycle phase transition	Sapur.002G001600 Sapur.008G057000 Sapur.008G145300 Sapur.010G056400 Sapur.010G145400 Sapur.012G020900
GO:1901990	6	13	regulation of mitotic cell cycle phase transition	Sapur.002G001600 Sapur.008G057000 Sapur.008G145300 Sapur.010G056400 Sapur.010G145400 Sapur.012G020900
GO:0006418	14	46	tRNA aminoacylation for protein translation	Sapur.002G161200 Sapur.007G002900 Sapur.007G115600 Sapur.009G112500 Sapur.010G093900 Sapur.011G008400 Sapur.002G194100 Sapur.003G002000 Sapur.003G168000 Sapur.006G136200 Sapur.007G112200 Sapur.010G009500 Sapur.010G163200 Sapur.017G020600
GO:0033043	11	33	regulation of organelle organization	Sapur.002G001600 Sapur.004G071100 Sapur.007G123700 Sapur.008G057000 Sapur.008G145300 Sapur.010G056400 Sapur.010G145400 Sapur.012G099900 Sapur.017G006400 Sapur.017G006500 Sapur.15WG062900
GO:0006812	57	265	monoatomic cation transport	Sapur.001G008300 Sapur.001G054900 Sapur.001G081300 Sapur.001G137400 Sapur.002G003300 Sapur.002G150200 Sapur.003G090400 Sapur.003G098800 Sapur.004G051000 Sapur.004G162100 Sapur.005G009200 Sapur.005G019400 Sapur.005G028900 Sapur.005G119500 Sapur.005G122600 Sapur.006G004800 Sapur.006G014400 Sapur.006G042200 Sapur.006G042300 Sapur.006G084800 Sapur.006G174900 Sapur.006G181200 Sapur.007G080400 Sapur.007G094100 Sapur.008G065700 Sapur.008G066200 Sapur.008G163400 Sapur.009G045700 Sapur.009G057300 Sapur.009G057800 Sapur.009G063500 Sapur.009G107600 Sapur.010G004000 Sapur.010G015700 Sapur.010G086800 Sapur.010G133500 Sapur.010G142500 Sapur.012G003700 Sapur.013G018100 Sapur.014G108300 Sapur.016G074300 Sapur.016G087600 Sapur.016G107800 Sapur.016G194200 Sapur.016G225300 Sapur.016G291800 Sapur.017G059600 Sapur.017G120700 Sapur.017G121300 Sapur.017G121600 Sapur.017G121700 Sapur.017G122100 Sapur.018G103300 Sapur.15ZG002800 Sapur.006G076500 Sapur.009G028800 Sapur.009G028900
GO:1905818	5	10	regulation of chromosome	Sapur.002G001600 Sapur.008G057000 Sapur.008G145300 Sapur.010G056400 Sapur.010G145400

			separation	
GO:1905819	5	10	negative regulation of chromosome separation	Sapur.002G001600 Sapur.008G057000 Sapur.008G145300 Sapur.010G056400 Sapur.010G145400
GO:0071173	5	10	spindle assembly checkpoint signaling	Sapur.002G001600 Sapur.008G057000 Sapur.008G145300 Sapur.010G056400 Sapur.010G145400
GO:0071174	5	10	mitotic spindle checkpoint signaling	Sapur.002G001600 Sapur.008G057000 Sapur.008G145300 Sapur.010G056400 Sapur.010G145400
GO:0051985	5	10	negative regulation of chromosome segregation	Sapur.002G001600 Sapur.008G057000 Sapur.008G145300 Sapur.010G056400 Sapur.010G145400
GO:0051783	5	10	regulation of nuclear division	Sapur.002G001600 Sapur.008G057000 Sapur.008G145300 Sapur.010G056400 Sapur.010G145400
GO:0051784	5	10	negative regulation of nuclear division	Sapur.002G001600 Sapur.008G057000 Sapur.008G145300 Sapur.010G056400 Sapur.010G145400
GO:0031577	5	10	spindle checkpoint signaling	Sapur.002G001600 Sapur.008G057000 Sapur.008G145300 Sapur.010G056400 Sapur.010G145400
GO:1902100	5	10	negative regulation of metaphase/anaphase transition of cell cycle	Sapur.002G001600 Sapur.008G057000 Sapur.008G145300 Sapur.010G056400 Sapur.010G145400
GO:0010965	5	10	regulation of mitotic sister chromatid separation	Sapur.002G001600 Sapur.008G057000 Sapur.008G145300 Sapur.010G056400 Sapur.010G145400
GO:200	5	10	negative	Sapur.002G001600 Sapur.008G057000 Sapur.008G145300 Sapur.010G056400 Sapur.010G145400

1251			regulation of chromosome organization	
GO:2000816	5	10	negative regulation of mitotic sister chromatid separation	Sapur.002G001600 Sapur.008G057000 Sapur.008G145300 Sapur.010G056400 Sapur.010G145400
GO:0045841	5	10	negative regulation of mitotic metaphase/anaphase transition	Sapur.002G001600 Sapur.008G057000 Sapur.008G145300 Sapur.010G056400 Sapur.010G145400
GO:0045839	5	10	negative regulation of mitotic nuclear division	Sapur.002G001600 Sapur.008G057000 Sapur.008G145300 Sapur.010G056400 Sapur.010G145400
GO:0051306	5	10	mitotic sister chromatid separation	Sapur.002G001600 Sapur.008G057000 Sapur.008G145300 Sapur.010G056400 Sapur.010G145400
GO:0007094	5	10	mitotic spindle assembly checkpoint signaling	Sapur.002G001600 Sapur.008G057000 Sapur.008G145300 Sapur.010G056400 Sapur.010G145400
GO:0007088	5	10	regulation of mitotic nuclear division	Sapur.002G001600 Sapur.008G057000 Sapur.008G145300 Sapur.010G056400 Sapur.010G145400
GO:0033046	5	10	negative regulation of sister chromatid segregation	Sapur.002G001600 Sapur.008G057000 Sapur.008G145300 Sapur.010G056400 Sapur.010G145400
GO:0033047	5	10	regulation of mitotic sister	Sapur.002G001600 Sapur.008G057000 Sapur.008G145300 Sapur.010G056400 Sapur.010G145400

GO:0033048	5	10	chromatid segregation negative regulation of mitotic sister chromatid segregation	Sapur.002G001600 Sapur.008G057000 Sapur.008G145300 Sapur.010G056400 Sapur.010G145400
GO:0009581	3	4	detection of external stimulus	Sapur.010G113700 Sapur.013G000400 Sapur.15WG060500
GO:0009582	3	4	detection of abiotic stimulus	Sapur.010G113700 Sapur.013G000400 Sapur.15WG060500
GO:0009583	3	4	detection of light stimulus	Sapur.010G113700 Sapur.013G000400 Sapur.15WG060500
GO:0009584	3	4	detection of visible light	Sapur.010G113700 Sapur.013G000400 Sapur.15WG060500
GO:0051606	3	4	detection of stimulus	Sapur.010G113700 Sapur.013G000400 Sapur.15WG060500
GO:0009416	4	7	response to light stimulus	Sapur.009G112000 Sapur.010G113700 Sapur.013G000400 Sapur.15WG060500
GO:0051247	6	14	positive regulation of protein metabolic process	Sapur.004G071100 Sapur.007G123700 Sapur.012G099900 Sapur.017G006400 Sapur.017G006500 Sapur.15WG062900
GO:0055086	34	147	nucleobase-containing small molecule metabolic process	Sapur.001G183500 Sapur.002G112100 Sapur.003G092400 Sapur.004G008500 Sapur.006G083400 Sapur.008G102800 Sapur.010G086900 Sapur.010G087900 Sapur.010G134600 Sapur.010G151500 Sapur.011G014000 Sapur.011G064300 Sapur.012G055400 Sapur.013G108500 Sapur.016G104000 Sapur.018G022500 Sapur.018G045400 Sapur.018G047900 Sapur.15ZG085300 Sapur.003G056300 Sapur.004G156500 Sapur.002G047600 Sapur.004G123200 Sapur.008G019800 Sapur.009G099900 Sapur.013G094800 Sapur.013G094900 Sapur.013G095000 Sapur.013G095100 Sapur.001G057600 Sapur.003G116600 Sapur.008G063700 Sapur.010G137300 Sapur.014G098600
GO:0043038	14	48	amino acid activation	Sapur.002G161200 Sapur.007G002900 Sapur.007G115600 Sapur.009G112500 Sapur.010G093900 Sapur.011G008400 Sapur.002G194100 Sapur.003G002000 Sapur.003G168000 Sapur.006G136200 Sapur.007G112200 Sapur.010G009500 Sapur.010G163200 Sapur.017G020600
GO:004	14	48	tRNA	Sapur.002G161200 Sapur.007G002900 Sapur.007G115600 Sapur.009G112500 Sapur.010G093900

3039			aminoacylation	Sapur.011G008400 Sapur.002G194100 Sapur.003G002000 Sapur.003G168000 Sapur.006G136200 Sapur.007G112200 Sapur.010G009500 Sapur.010G163200 Sapur.017G020600
GO:0031325	7	18	positive regulation of cellular metabolic process	Sapur.004G071100 Sapur.007G123700 Sapur.012G099900 Sapur.017G006400 Sapur.017G006500 Sapur.019G060100 Sapur.15WG062900
GO:0009893	7	18	positive regulation of metabolic process	Sapur.004G071100 Sapur.007G123700 Sapur.012G099900 Sapur.017G006400 Sapur.017G006500 Sapur.019G060100 Sapur.15WG062900
GO:0051173	7	18	positive regulation of nitrogen compound metabolic process	Sapur.004G071100 Sapur.007G123700 Sapur.012G099900 Sapur.017G006400 Sapur.017G006500 Sapur.019G060100 Sapur.15WG062900
GO:0010604	7	18	positive regulation of macromolecule metabolic process	Sapur.004G071100 Sapur.007G123700 Sapur.012G099900 Sapur.017G006400 Sapur.017G006500 Sapur.019G060100 Sapur.15WG062900
GO:0034660	30	127	ncRNA metabolic process	Sapur.001G163900 Sapur.002G161200 Sapur.005G046200 Sapur.005G050200 Sapur.007G002900 Sapur.007G044500 Sapur.007G115600 Sapur.008G044200 Sapur.008G098100 Sapur.008G168800 Sapur.009G099800 Sapur.009G112500 Sapur.010G011800 Sapur.010G093900 Sapur.011G008400 Sapur.014G112900 Sapur.016G152700 Sapur.016G205800 Sapur.016G225800 Sapur.016G310400 Sapur.018G090600 Sapur.15ZG080300 Sapur.002G194100 Sapur.003G002000 Sapur.003G168000 Sapur.006G136200 Sapur.007G112200 Sapur.010G009500 Sapur.010G163200 Sapur.017G020600
GO:0048523	15	54	negative regulation of cellular process	Sapur.002G001600 Sapur.002G070300 Sapur.003G142400 Sapur.004G027800 Sapur.004G100100 Sapur.005G135200 Sapur.008G057000 Sapur.008G145300 Sapur.009G002400 Sapur.010G056400 Sapur.010G144500 Sapur.010G145400 Sapur.012G020900 Sapur.013G067900 Sapur.019G039400
GO:0048519	15	54	negative regulation of biological process	Sapur.002G001600 Sapur.002G070300 Sapur.003G142400 Sapur.004G027800 Sapur.004G100100 Sapur.005G135200 Sapur.008G057000 Sapur.008G145300 Sapur.009G002400 Sapur.010G056400 Sapur.010G144500 Sapur.010G145400 Sapur.012G020900 Sapur.013G067900 Sapur.019G039400
GO:1901617	5	11	organic hydroxy compound biosynthetic	Sapur.005G061300 Sapur.007G081700 Sapur.012G084400 Sapur.016G101200 Sapur.017G106300

			process	
GO:0000041	6	15	transition metal ion transport	Sapur.005G009200 Sapur.006G084800 Sapur.016G107800 Sapur.006G076500 Sapur.009G028800 Sapur.009G028900
GO:0019751	7	19	polyol metabolic process	Sapur.005G061300 Sapur.007G081700 Sapur.008G116400 Sapur.012G084400 Sapur.018G050900 Sapur.009G066300 Sapur.017G003500
GO:0006021	2	2	inositol biosynthetic process	Sapur.005G061300 Sapur.007G081700
GO:0006261	4	8	DNA-templated DNA replication	Sapur.001G055600 Sapur.001G058800 Sapur.003G070500 Sapur.T047900
GO:0051128	11	37	regulation of cellular component organization	Sapur.002G001600 Sapur.004G071100 Sapur.007G123700 Sapur.008G057000 Sapur.008G145300 Sapur.010G056400 Sapur.010G145400 Sapur.012G099900 Sapur.017G006400 Sapur.017G006500 Sapur.15WG062900
GO:0009165	20	81	nucleotide biosynthetic process	Sapur.002G112100 Sapur.003G092400 Sapur.004G008500 Sapur.006G083400 Sapur.010G086900 Sapur.010G134600 Sapur.010G151500 Sapur.011G014000 Sapur.011G064300 Sapur.012G055400 Sapur.013G108500 Sapur.016G104000 Sapur.018G022500 Sapur.15ZG085300 Sapur.003G056300 Sapur.001G057600 Sapur.003G116600 Sapur.008G063700 Sapur.010G137300 Sapur.014G098600
GO:1901293	20	81	nucleoside phosphate biosynthetic process	Sapur.002G112100 Sapur.003G092400 Sapur.004G008500 Sapur.006G083400 Sapur.010G086900 Sapur.010G134600 Sapur.010G151500 Sapur.011G014000 Sapur.011G064300 Sapur.012G055400 Sapur.013G108500 Sapur.016G104000 Sapur.018G022500 Sapur.15ZG085300 Sapur.003G056300 Sapur.001G057600 Sapur.003G116600 Sapur.008G063700 Sapur.010G137300 Sapur.014G098600
GO:0048522	7	20	positive regulation of cellular process	Sapur.004G071100 Sapur.007G123700 Sapur.012G099900 Sapur.017G006400 Sapur.017G006500 Sapur.019G060100 Sapur.15WG062900
GO:0048518	7	20	positive regulation of biological process	Sapur.004G071100 Sapur.007G123700 Sapur.012G099900 Sapur.017G006400 Sapur.017G006500 Sapur.019G060100 Sapur.15WG062900
GO:0044784	5	12	metaphase/anaphase transition of cell cycle	Sapur.002G001600 Sapur.008G057000 Sapur.008G145300 Sapur.010G056400 Sapur.010G145400
GO:005	5	12	regulation of	Sapur.002G001600 Sapur.008G057000 Sapur.008G145300 Sapur.010G056400 Sapur.010G145400

1983			chromosome segregation	
GO:1902099	5	12	regulation of metaphase/anaphase transition of cell cycle	Sapur.002G001600 Sapur.008G057000 Sapur.008G145300 Sapur.010G056400 Sapur.010G145400
GO:0030071	5	12	regulation of mitotic metaphase/anaphase transition	Sapur.002G001600 Sapur.008G057000 Sapur.008G145300 Sapur.010G056400 Sapur.010G145400
GO:0007091	5	12	metaphase/anaphase transition of mitotic cell cycle	Sapur.002G001600 Sapur.008G057000 Sapur.008G145300 Sapur.010G056400 Sapur.010G145400
GO:0051129	5	12	negative regulation of cellular component organization	Sapur.002G001600 Sapur.008G057000 Sapur.008G145300 Sapur.010G056400 Sapur.010G145400
GO:0033044	5	12	regulation of chromosome organization	Sapur.002G001600 Sapur.008G057000 Sapur.008G145300 Sapur.010G056400 Sapur.010G145400
GO:0033045	5	12	regulation of sister chromatid segregation	Sapur.002G001600 Sapur.008G057000 Sapur.008G145300 Sapur.010G056400 Sapur.010G145400
GO:0010639	5	12	negative regulation of organelle organization	Sapur.002G001600 Sapur.008G057000 Sapur.008G145300 Sapur.010G056400 Sapur.010G145400
GO:0006811	65	324	monoatomic ion transport	Sapur.001G008300 Sapur.001G054900 Sapur.001G081300 Sapur.001G137400 Sapur.002G003300 Sapur.002G150200 Sapur.003G090400 Sapur.003G098800 Sapur.004G051000 Sapur.004G157800 Sapur.004G162100 Sapur.005G009200 Sapur.005G019400 Sapur.005G028900 Sapur.005G119500 Sapur.005G122600 Sapur.006G004800 Sapur.006G014400 Sapur.006G042200 Sapur.006G042300 Sapur.006G084800 Sapur.006G174900 Sapur.006G181200

				Sapur.007G080400 Sapur.007G094100 Sapur.008G065700 Sapur.008G066200 Sapur.008G163400 Sapur.009G045700 Sapur.009G057300 Sapur.009G057800 Sapur.009G063500 Sapur.009G107600 Sapur.010G004000 Sapur.010G015700 Sapur.010G086800 Sapur.010G133500 Sapur.010G142500 Sapur.012G003700 Sapur.013G018100 Sapur.014G108300 Sapur.016G074300 Sapur.016G087600 Sapur.016G107800 Sapur.016G194200 Sapur.016G225300 Sapur.016G291800 Sapur.017G059600 Sapur.017G120700 Sapur.017G121300 Sapur.017G121600 Sapur.017G121700 Sapur.017G122100 Sapur.018G103300 Sapur.15ZG002800 Sapur.006G206900 Sapur.012G017000 Sapur.012G025300 Sapur.012G028700 Sapur.016G266000 Sapur.018G026000 Sapur.15ZG026500 Sapur.006G076500 Sapur.009G028800 Sapur.009G028900
GO:0052646	3	5	alditol phosphate metabolic process	Sapur.001G017800 Sapur.013G085300 Sapur.016G040000
GO:0006072	3	5	glycerol-3-phosphate metabolic process	Sapur.001G017800 Sapur.013G085300 Sapur.016G040000
GO:0043094	3	5	cellular metabolic compound salvage	Sapur.006G083400 Sapur.016G104000 Sapur.017G106300
GO:0006525	3	5	arginine metabolic process	Sapur.002G085400 Sapur.004G122800 Sapur.010G193600
GO:0006528	3	5	asparagine metabolic process	Sapur.005G058900 Sapur.009G056800 Sapur.010G121200
GO:0006529	3	5	asparagine biosynthetic process	Sapur.005G058900 Sapur.009G056800 Sapur.010G121200
GO:0001932	7	21	regulation of protein phosphorylation	Sapur.004G071100 Sapur.007G123700 Sapur.008G121600 Sapur.012G099900 Sapur.017G006400 Sapur.017G006500 Sapur.15WG062900
GO:0045859	7	21	regulation of protein kinase activity	Sapur.004G071100 Sapur.007G123700 Sapur.008G121600 Sapur.012G099900 Sapur.017G006400 Sapur.017G006500 Sapur.15WG062900
GO:0009116	9	30	nucleoside metabolic process	Sapur.018G047900 Sapur.002G047600 Sapur.004G123200 Sapur.008G019800 Sapur.009G099900 Sapur.013G094800 Sapur.013G094900 Sapur.013G095000 Sapur.013G095100
GO:1901657	9	30	glycosyl compound metabolic process	Sapur.018G047900 Sapur.002G047600 Sapur.004G123200 Sapur.008G019800 Sapur.009G099900 Sapur.013G094800 Sapur.013G094900 Sapur.013G095000 Sapur.013G095100
GO:000	6	17	pyrimidine	Sapur.002G112100 Sapur.001G057600 Sapur.003G116600 Sapur.008G063700 Sapur.010G137300

	6220			nucleotide metabolic process	Sapur.014G098600
	GO:0006221	6	17	pyrimidine nucleotide biosynthetic process	Sapur.002G112100 Sapur.001G057600 Sapur.003G116600 Sapur.008G063700 Sapur.010G137300 Sapur.014G098600
	GO:0007051	6	17	spindle organization	Sapur.004G071100 Sapur.007G123700 Sapur.012G099900 Sapur.017G006400 Sapur.017G006500 Sapur.15WG062900
	GO:0006298	5	13	mismatch repair	Sapur.004G027800 Sapur.010G046700 Sapur.012G000700 Sapur.014G104700 Sapur.019G075900
	GO:0009605	5	13	response to external stimulus	Sapur.009G010000 Sapur.010G175000 Sapur.010G113700 Sapur.013G000400 Sapur.15WG060500
	GO:0044281	101	536	small molecule metabolic process	Sapur.001G057600 Sapur.001G129600 Sapur.001G129800 Sapur.001G183500 Sapur.002G085400 Sapur.002G112100 Sapur.002G115900 Sapur.002G158200 Sapur.002G161200 Sapur.003G092400 Sapur.004G008500 Sapur.004G122100 Sapur.004G122800 Sapur.005G058900 Sapur.005G061300 Sapur.005G147100 Sapur.006G027800 Sapur.006G049700 Sapur.006G083400 Sapur.007G002900 Sapur.007G008900 Sapur.007G081700 Sapur.007G115600 Sapur.008G002300 Sapur.008G022600 Sapur.008G066800 Sapur.008G102800 Sapur.008G116400 Sapur.008G161100 Sapur.009G056800 Sapur.009G098500 Sapur.009G112500 Sapur.010G014000 Sapur.010G086900 Sapur.010G087900 Sapur.010G093900 Sapur.010G121200 Sapur.010G134600 Sapur.010G151500 Sapur.010G186700 Sapur.010G193600 Sapur.010G206100 Sapur.011G008400 Sapur.011G014000 Sapur.011G064300 Sapur.012G021100 Sapur.012G055400 Sapur.012G084400 Sapur.013G004800 Sapur.013G054200 Sapur.013G056200 Sapur.013G108500 Sapur.013G115700 Sapur.014G098600 Sapur.014G135900 Sapur.016G101200 Sapur.016G104000 Sapur.016G228700 Sapur.016G231000 Sapur.017G106300 Sapur.017G108800 Sapur.017G123100 Sapur.018G022500 Sapur.018G045400 Sapur.018G047900 Sapur.018G050900 Sapur.019G070000 Sapur.15ZG085300 Sapur.15ZG101500 Sapur.003G056300 Sapur.004G156500 Sapur.009G066300 Sapur.017G003500 Sapur.017G124700 Sapur.001G180200 Sapur.002G047600 Sapur.004G123200 Sapur.008G019800 Sapur.008G054900 Sapur.009G099900 Sapur.011G120500 Sapur.013G094800 Sapur.013G094900 Sapur.013G095000 Sapur.013G095100 Sapur.016G153900 Sapur.15ZG092000 Sapur.002G194100 Sapur.003G002000 Sapur.003G116600 Sapur.003G168000 Sapur.006G136200 Sapur.007G112200 Sapur.008G063700 Sapur.010G009500 Sapur.010G137300 Sapur.010G163200 Sapur.017G020600 Sapur.004G028700 Sapur.004G108600 Sapur.011G036500
m_down_CC	GO:0012505	96	184	endomembrane system	Sapur.001G037800 Sapur.002G118400 Sapur.003G100300 Sapur.003G137200 Sapur.005G058800 Sapur.006G150200 Sapur.014G048500 Sapur.016G098500 Sapur.017G027100 Sapur.018G081000 Sapur.001G046700 Sapur.002G004300 Sapur.002G138300 Sapur.002G161300 Sapur.002G190100 Sapur.004G005200 Sapur.005G013100 Sapur.005G204000 Sapur.006G012300 Sapur.008G028500 Sapur.008G108600 Sapur.009G079600 Sapur.010G081400

				Sapur.010G105000 Sapur.010G115400 Sapur.010G178800 Sapur.011G006300 Sapur.012G028100 Sapur.012G083500 Sapur.013G008900 Sapur.014G018800 Sapur.014G098300 Sapur.014G105300 Sapur.016G198200 Sapur.017G016100 Sapur.017G052500 Sapur.15WG081100 Sapur.15ZG059400 Sapur.15ZG109000 Sapur.T064000 Sapur.001G069700 Sapur.001G112600 Sapur.001G126200 Sapur.002G013700 Sapur.002G028200 Sapur.002G046500 Sapur.002G116800 Sapur.002G189800 Sapur.003G051000 Sapur.003G060700 Sapur.003G062100 Sapur.003G063400 Sapur.003G074700 Sapur.003G107200 Sapur.003G139000 Sapur.004G020700 Sapur.004G026500 Sapur.004G095900 Sapur.004G115300 Sapur.004G127400 Sapur.005G028800 Sapur.005G101600 Sapur.005G160500 Sapur.005G180500 Sapur.006G185800 Sapur.006G194300 Sapur.006G223800 Sapur.007G028100 Sapur.008G051200 Sapur.008G051300 Sapur.008G142600 Sapur.009G009400 Sapur.009G054300 Sapur.009G057100 Sapur.009G091000 Sapur.009G103300 Sapur.009G109200 Sapur.010G051000 Sapur.010G108800 Sapur.010G146400 Sapur.010G149500 Sapur.011G034100 Sapur.012G098100 Sapur.013G030300 Sapur.014G040300 Sapur.014G044700 Sapur.014G079400 Sapur.014G136300 Sapur.016G163500 Sapur.016G218100 Sapur.018G006400 Sapur.019G022400 Sapur.019G097900 Sapur.15ZG071300 Sapur.T049800 Sapur.T080400
GO:0016020	296	1182	membrane	Sapur.001G037800 Sapur.002G118400 Sapur.003G100300 Sapur.003G137200 Sapur.003G157700 Sapur.005G058800 Sapur.006G150200 Sapur.014G048500 Sapur.016G098500 Sapur.017G027100 Sapur.018G081000 Sapur.001G009500 Sapur.001G009600 Sapur.001G030600 Sapur.001G033600 Sapur.001G037600 Sapur.001G043500 Sapur.001G089300 Sapur.001G110200 Sapur.001G146600 Sapur.001G164900 Sapur.001G185400 Sapur.001G185600 Sapur.001G187000 Sapur.002G006100 Sapur.002G038200 Sapur.002G047800 Sapur.002G063400 Sapur.002G072000 Sapur.002G086200 Sapur.002G092100 Sapur.002G098200 Sapur.002G108400 Sapur.002G110800 Sapur.002G139100 Sapur.002G176800 Sapur.002G193600 Sapur.003G029600 Sapur.003G034800 Sapur.003G076900 Sapur.003G082000 Sapur.003G086600 Sapur.003G092800 Sapur.003G095000 Sapur.003G099600 Sapur.003G138600 Sapur.003G141500 Sapur.003G149100 Sapur.004G034200 Sapur.004G070600 Sapur.004G102500 Sapur.004G103700 Sapur.004G116800 Sapur.004G132700 Sapur.004G141700 Sapur.004G143900 Sapur.004G168100 Sapur.004G168900 Sapur.005G027800 Sapur.005G044700 Sapur.005G086200 Sapur.005G125100 Sapur.005G180700 Sapur.005G201800 Sapur.006G008700 Sapur.006G008800 Sapur.006G023800 Sapur.006G049200 Sapur.006G050300 Sapur.006G054000 Sapur.006G061500 Sapur.006G062000 Sapur.006G067800 Sapur.006G084000 Sapur.006G105100 Sapur.006G105200 Sapur.006G107800 Sapur.006G192900 Sapur.006G198200 Sapur.006G198700 Sapur.006G206200 Sapur.006G212700 Sapur.006G217000 Sapur.006G217200 Sapur.007G044700 Sapur.008G003400 Sapur.008G004000 Sapur.008G011900 Sapur.008G038400 Sapur.008G065200 Sapur.008G070800 Sapur.008G103100 Sapur.008G105500 Sapur.008G113200 Sapur.008G126700 Sapur.008G149700 Sapur.008G162700 Sapur.009G006300 Sapur.009G019400 Sapur.009G062300 Sapur.009G063600 Sapur.009G068000 Sapur.009G079300 Sapur.009G109000 Sapur.009G117600 Sapur.009G120200 Sapur.009G130600 Sapur.010G007300 Sapur.010G023600 Sapur.010G048300 Sapur.010G073400 Sapur.010G087400 Sapur.010G090500 Sapur.010G092800 Sapur.010G098800 Sapur.010G108800 Sapur.010G118100 Sapur.010G124000 Sapur.010G129800

				Sapur.010G134800 Sapur.010G150300 Sapur.010G165600 Sapur.010G199900 Sapur.010G203100 Sapur.010G205600 Sapur.011G008300 Sapur.011G041500 Sapur.011G050700 Sapur.011G054900 Sapur.011G071500 Sapur.011G075900 Sapur.011G101900 Sapur.011G112000 Sapur.012G025200 Sapur.012G052600 Sapur.012G060700 Sapur.013G013900 Sapur.013G023800 Sapur.013G064300 Sapur.013G068300 Sapur.013G080100 Sapur.013G090600 Sapur.013G100800 Sapur.013G101200 Sapur.013G109400 Sapur.013G121500 Sapur.014G030900 Sapur.014G092800 Sapur.014G125100 Sapur.014G128700 Sapur.016G010700 Sapur.016G010900 Sapur.016G047900 Sapur.016G056800 Sapur.016G082300 Sapur.016G097500 Sapur.016G109800 Sapur.016G111800 Sapur.016G113700 Sapur.016G127900 Sapur.016G147600 Sapur.016G203700 Sapur.016G221000 Sapur.016G229400 Sapur.016G231500 Sapur.016G231600 Sapur.016G242000 Sapur.016G244800 Sapur.016G248500 Sapur.016G252400 Sapur.016G290200 Sapur.016G310000 Sapur.017G056200 Sapur.017G062700 Sapur.017G073700 Sapur.017G114600 Sapur.018G005200 Sapur.018G009200 Sapur.018G015400 Sapur.018G020900 Sapur.018G026100 Sapur.018G026500 Sapur.018G032300 Sapur.018G053400 Sapur.018G062800 Sapur.018G069900 Sapur.018G094800 Sapur.018G096200 Sapur.018G097400 Sapur.018G108000 Sapur.018G110200 Sapur.019G035900 Sapur.019G076600 Sapur.019G079900 Sapur.019G099900 Sapur.15WG064400 Sapur.15ZG001200 Sapur.15ZG004600 Sapur.15ZG018200 Sapur.15ZG021300 Sapur.15ZG052500 Sapur.15ZG072800 Sapur.15ZG073700 Sapur.15ZG079800 Sapur.15ZG093800 Sapur.T001500 Sapur.T052300 Sapur.T140400 Sapur.001G069700 Sapur.001G112600 Sapur.001G126200 Sapur.001G187900 Sapur.002G013700 Sapur.002G028200 Sapur.002G116800 Sapur.002G189800 Sapur.003G020000 Sapur.003G051000 Sapur.003G054000 Sapur.003G060700 Sapur.003G062100 Sapur.003G063400 Sapur.003G107200 Sapur.004G020700 Sapur.004G026500 Sapur.004G050300 Sapur.004G095900 Sapur.004G115300 Sapur.004G127400 Sapur.004G142200 Sapur.005G028800 Sapur.005G101600 Sapur.005G121300 Sapur.005G180500 Sapur.005G199000 Sapur.006G062900 Sapur.006G194300 Sapur.006G223800 Sapur.007G006700 Sapur.007G023000 Sapur.007G028100 Sapur.007G051300 Sapur.008G051200 Sapur.008G051300 Sapur.008G142600 Sapur.008G142900 Sapur.009G009400 Sapur.009G057100 Sapur.009G091000 Sapur.009G103300 Sapur.009G118300 Sapur.010G051000 Sapur.010G115400 Sapur.010G146400 Sapur.010G149500 Sapur.010G159200 Sapur.010G173300 Sapur.010G177400 Sapur.010G179000 Sapur.010G202000 Sapur.011G034100 Sapur.012G071300 Sapur.012G098100 Sapur.013G030300 Sapur.013G064900 Sapur.014G040300 Sapur.014G044700 Sapur.014G079400 Sapur.014G123600 Sapur.016G163500 Sapur.016G175100 Sapur.016G204500 Sapur.016G218100 Sapur.016G275200 Sapur.018G006400 Sapur.018G041100 Sapur.019G022400 Sapur.019G097900 Sapur.15ZG071300 Sapur.T049800 Sapur.001G082900 Sapur.001G154800 Sapur.003G029700 Sapur.003G094300 Sapur.006G076200 Sapur.006G201300 Sapur.15WG044900 Sapur.15ZG049100 Sapur.002G021300 Sapur.005G186900 Sapur.010G205000 Sapur.013G047900 Sapur.017G065500 Sapur.T003500 Sapur.T012400 Sapur.T031900
GO:0005783	64	110	endoplasmic reticulum	Sapur.006G150200 Sapur.018G081000 Sapur.001G046700 Sapur.002G004300 Sapur.002G138300 Sapur.002G161300 Sapur.002G190100 Sapur.004G005200 Sapur.005G013100 Sapur.005G204000 Sapur.006G012300 Sapur.008G028500 Sapur.008G108600 Sapur.009G079600 Sapur.010G081400 Sapur.010G105000 Sapur.010G115400

				Sapur.010G178800 Sapur.011G006300 Sapur.012G028100 Sapur.012G083500 Sapur.013G008900 Sapur.014G098300 Sapur.014G105300 Sapur.016G198200 Sapur.017G016100 Sapur.017G052500 Sapur.15WG081100 Sapur.15ZG059400 Sapur.15ZG109000 Sapur.T064000 Sapur.001G069700 Sapur.001G112600 Sapur.001G126200 Sapur.002G028200 Sapur.002G116800 Sapur.002G189800 Sapur.003G051000 Sapur.003G060700 Sapur.003G062100 Sapur.003G063400 Sapur.003G107200 Sapur.004G020700 Sapur.004G095900 Sapur.004G127400 Sapur.005G028800 Sapur.005G101600 Sapur.005G180500 Sapur.006G194300 Sapur.007G028100 Sapur.008G051200 Sapur.008G051300 Sapur.008G142600 Sapur.009G009400 Sapur.009G103300 Sapur.010G051000 Sapur.012G098100 Sapur.013G030300 Sapur.014G040300 Sapur.014G044700 Sapur.014G079400 Sapur.016G218100 Sapur.019G022400 Sapur.T049800
GO:0031090	77	157	organelle membrane	Sapur.001G037800 Sapur.002G118400 Sapur.003G100300 Sapur.003G137200 Sapur.005G058800 Sapur.006G150200 Sapur.014G048500 Sapur.016G098500 Sapur.017G027100 Sapur.018G081000 Sapur.001G069700 Sapur.001G112600 Sapur.001G126200 Sapur.001G187900 Sapur.002G028200 Sapur.002G116800 Sapur.002G189800 Sapur.003G020000 Sapur.003G051000 Sapur.003G060700 Sapur.003G062100 Sapur.003G063400 Sapur.003G107200 Sapur.004G020700 Sapur.004G026500 Sapur.004G050300 Sapur.004G095900 Sapur.004G115300 Sapur.004G127400 Sapur.004G142200 Sapur.005G028800 Sapur.005G101600 Sapur.005G121300 Sapur.005G180500 Sapur.005G199000 Sapur.006G062900 Sapur.006G194300 Sapur.006G223800 Sapur.007G006700 Sapur.007G028100 Sapur.007G051300 Sapur.008G051200 Sapur.008G051300 Sapur.008G142600 Sapur.008G142900 Sapur.009G009400 Sapur.009G057100 Sapur.009G091000 Sapur.009G103300 Sapur.009G118300 Sapur.010G051000 Sapur.010G115400 Sapur.010G146400 Sapur.010G149500 Sapur.010G173300 Sapur.010G177400 Sapur.010G179000 Sapur.010G202000 Sapur.011G034100 Sapur.012G071300 Sapur.012G098100 Sapur.013G030300 Sapur.013G064900 Sapur.014G040300 Sapur.014G044700 Sapur.014G079400 Sapur.014G123600 Sapur.016G163500 Sapur.016G175100 Sapur.016G204500 Sapur.016G218100 Sapur.018G006400 Sapur.018G041100 Sapur.019G022400 Sapur.019G097900 Sapur.15ZG071300 Sapur.T049800
GO:0098796	67	146	membrane protein complex	Sapur.006G150200 Sapur.018G081000 Sapur.001G112600 Sapur.001G187900 Sapur.002G013700 Sapur.002G028200 Sapur.002G116800 Sapur.002G189800 Sapur.003G020000 Sapur.003G054000 Sapur.003G063400 Sapur.004G026500 Sapur.004G115300 Sapur.004G142200 Sapur.005G028800 Sapur.005G180500 Sapur.005G199000 Sapur.006G062900 Sapur.006G194300 Sapur.006G223800 Sapur.007G006700 Sapur.007G023000 Sapur.008G051200 Sapur.008G051300 Sapur.008G142600 Sapur.008G142900 Sapur.009G009400 Sapur.009G057100 Sapur.009G091000 Sapur.009G118300 Sapur.010G051000 Sapur.010G115400 Sapur.010G146400 Sapur.010G149500 Sapur.010G159200 Sapur.010G173300 Sapur.010G177400 Sapur.010G202000 Sapur.011G034100 Sapur.013G064900 Sapur.014G044700 Sapur.014G123600 Sapur.016G163500 Sapur.016G204500 Sapur.016G218100 Sapur.018G006400 Sapur.018G041100 Sapur.019G022400 Sapur.019G097900 Sapur.15ZG071300 Sapur.T049800 Sapur.001G082900 Sapur.001G154800 Sapur.003G029700 Sapur.003G094300 Sapur.006G076200 Sapur.006G201300 Sapur.15WG044900 Sapur.15ZG049100 Sapur.002G021300 Sapur.005G186900 Sapur.010G205000 Sapur.013G047900 Sapur.017G065500 Sapur.T003500 Sapur.T012400 Sapur.T031900

GO:0005737	165	572	cytoplasm	Sapur.001G037800 Sapur.002G118400 Sapur.003G100300 Sapur.003G137200 Sapur.005G058800 Sapur.006G150200 Sapur.014G048500 Sapur.016G098500 Sapur.017G027100 Sapur.018G081000 Sapur.001G046700 Sapur.001G187900 Sapur.002G004300 Sapur.002G137300 Sapur.002G138300 Sapur.002G161300 Sapur.002G176700 Sapur.002G181500 Sapur.002G190100 Sapur.002G202100 Sapur.003G020000 Sapur.003G076300 Sapur.004G005200 Sapur.004G007800 Sapur.005G013100 Sapur.005G156500 Sapur.005G204000 Sapur.006G012300 Sapur.006G067100 Sapur.006G223800 Sapur.008G007500 Sapur.008G028500 Sapur.008G043100 Sapur.008G108600 Sapur.008G114600 Sapur.009G079600 Sapur.009G099500 Sapur.010G042500 Sapur.010G045000 Sapur.010G081400 Sapur.010G105000 Sapur.010G115400 Sapur.010G160600 Sapur.010G178800 Sapur.011G003200 Sapur.011G006300 Sapur.012G028100 Sapur.012G083500 Sapur.013G008900 Sapur.013G024200 Sapur.013G105300 Sapur.014G018800 Sapur.014G062000 Sapur.014G098300 Sapur.014G099300 Sapur.014G105300 Sapur.016G096000 Sapur.016G198200 Sapur.016G227000 Sapur.017G016100 Sapur.017G052500 Sapur.018G006400 Sapur.018G031600 Sapur.018G075900 Sapur.018G083400 Sapur.15WG081100 Sapur.15ZG059400 Sapur.15ZG109000 Sapur.T064000 Sapur.001G069700 Sapur.001G112600 Sapur.001G126200 Sapur.001G144500 Sapur.002G028200 Sapur.002G046500 Sapur.002G053800 Sapur.002G116800 Sapur.002G189800 Sapur.003G051000 Sapur.003G060700 Sapur.003G062100 Sapur.003G063400 Sapur.003G074700 Sapur.003G107200 Sapur.003G139000 Sapur.004G020700 Sapur.004G026500 Sapur.004G050300 Sapur.004G095900 Sapur.004G115300 Sapur.004G127400 Sapur.004G142200 Sapur.005G028800 Sapur.005G101600 Sapur.005G121300 Sapur.005G160500 Sapur.005G180500 Sapur.005G199000 Sapur.006G062900 Sapur.006G167000 Sapur.006G185800 Sapur.006G194300 Sapur.007G006700 Sapur.007G023000 Sapur.007G028100 Sapur.007G051300 Sapur.008G051200 Sapur.008G051300 Sapur.008G142600 Sapur.008G142900 Sapur.009G009400 Sapur.009G054300 Sapur.009G057100 Sapur.009G091000 Sapur.009G103300 Sapur.009G109200 Sapur.009G118300 Sapur.010G051000 Sapur.010G108800 Sapur.010G144600 Sapur.010G146400 Sapur.010G149500 Sapur.010G159200 Sapur.010G173300 Sapur.010G177400 Sapur.010G179000 Sapur.010G195700 Sapur.010G202000 Sapur.011G034100 Sapur.012G071300 Sapur.012G098100 Sapur.013G030300 Sapur.013G064900 Sapur.013G109100 Sapur.014G040300 Sapur.014G044700 Sapur.014G079400 Sapur.014G123600 Sapur.014G136300 Sapur.016G065300 Sapur.016G163500 Sapur.016G175100 Sapur.016G204500 Sapur.016G218100 Sapur.016G258100 Sapur.018G041100 Sapur.019G022400 Sapur.019G097900 Sapur.15ZG071300 Sapur.T049800 Sapur.T080400 Sapur.001G082900 Sapur.001G154800 Sapur.003G029700 Sapur.003G094300 Sapur.005G174600 Sapur.006G076200 Sapur.006G201300 Sapur.012G030400 Sapur.15WG044900 Sapur.15ZG029200 Sapur.15ZG029900 Sapur.15ZG031600 Sapur.15ZG032200 Sapur.15ZG049100
GO:0005794	31	40	Golgi apparatus	Sapur.001G037800 Sapur.002G118400 Sapur.003G100300 Sapur.003G137200 Sapur.005G058800 Sapur.014G048500 Sapur.016G098500 Sapur.017G027100 Sapur.014G018800 Sapur.002G046500 Sapur.003G074700 Sapur.003G139000 Sapur.004G026500 Sapur.004G115300 Sapur.005G160500 Sapur.006G185800 Sapur.006G223800 Sapur.009G054300 Sapur.009G057100 Sapur.009G091000 Sapur.009G109200 Sapur.010G108800 Sapur.010G146400 Sapur.010G149500 Sapur.011G034100 Sapur.014G136300 Sapur.016G163500 Sapur.018G006400 Sapur.019G097900

				Sapur.15ZG071300 Sapur.T080400
GO:0031984	37	56	organelle subcompartment	Sapur.006G150200 Sapur.018G081000 Sapur.001G069700 Sapur.001G112600 Sapur.001G126200 Sapur.002G028200 Sapur.002G116800 Sapur.002G189800 Sapur.003G051000 Sapur.003G060700 Sapur.003G062100 Sapur.003G063400 Sapur.003G107200 Sapur.004G020700 Sapur.004G095900 Sapur.004G127400 Sapur.005G028800 Sapur.005G101600 Sapur.005G180500 Sapur.006G194300 Sapur.007G028100 Sapur.008G051200 Sapur.008G051300 Sapur.008G142600 Sapur.009G009400 Sapur.009G103300 Sapur.010G051000 Sapur.010G115400 Sapur.012G098100 Sapur.013G030300 Sapur.014G040300 Sapur.014G044700 Sapur.014G079400 Sapur.014G136300 Sapur.016G218100 Sapur.019G022400 Sapur.T049800
GO:0042175	36	55	nuclear outer membrane-endoplasmic reticulum membrane network	Sapur.006G150200 Sapur.018G081000 Sapur.001G069700 Sapur.001G112600 Sapur.001G126200 Sapur.002G028200 Sapur.002G116800 Sapur.002G189800 Sapur.003G051000 Sapur.003G060700 Sapur.003G062100 Sapur.003G063400 Sapur.003G107200 Sapur.004G020700 Sapur.004G095900 Sapur.004G127400 Sapur.005G028800 Sapur.005G101600 Sapur.005G180500 Sapur.006G194300 Sapur.007G028100 Sapur.008G051200 Sapur.008G051300 Sapur.008G142600 Sapur.009G009400 Sapur.009G103300 Sapur.010G051000 Sapur.010G115400 Sapur.012G098100 Sapur.013G030300 Sapur.014G040300 Sapur.014G044700 Sapur.014G079400 Sapur.016G218100 Sapur.019G022400 Sapur.T049800
GO:0098827	36	55	endoplasmic reticulum subcompartment	Sapur.006G150200 Sapur.018G081000 Sapur.001G069700 Sapur.001G112600 Sapur.001G126200 Sapur.002G028200 Sapur.002G116800 Sapur.002G189800 Sapur.003G051000 Sapur.003G060700 Sapur.003G062100 Sapur.003G063400 Sapur.003G107200 Sapur.004G020700 Sapur.004G095900 Sapur.004G127400 Sapur.005G028800 Sapur.005G101600 Sapur.005G180500 Sapur.006G194300 Sapur.007G028100 Sapur.008G051200 Sapur.008G051300 Sapur.008G142600 Sapur.009G009400 Sapur.009G103300 Sapur.010G051000 Sapur.010G115400 Sapur.012G098100 Sapur.013G030300 Sapur.014G040300 Sapur.014G044700 Sapur.014G079400 Sapur.016G218100 Sapur.019G022400 Sapur.T049800
GO:0005789	36	55	endoplasmic reticulum membrane	Sapur.006G150200 Sapur.018G081000 Sapur.001G069700 Sapur.001G112600 Sapur.001G126200 Sapur.002G028200 Sapur.002G116800 Sapur.002G189800 Sapur.003G051000 Sapur.003G060700 Sapur.003G062100 Sapur.003G063400 Sapur.003G107200 Sapur.004G020700 Sapur.004G095900 Sapur.004G127400 Sapur.005G028800 Sapur.005G101600 Sapur.005G180500 Sapur.006G194300 Sapur.007G028100 Sapur.008G051200 Sapur.008G051300 Sapur.008G142600 Sapur.009G009400 Sapur.009G103300 Sapur.010G051000 Sapur.010G115400 Sapur.012G098100 Sapur.013G030300 Sapur.014G040300 Sapur.014G044700 Sapur.014G079400 Sapur.016G218100 Sapur.019G022400 Sapur.T049800
GO:0140534	20	23	endoplasmic reticulum protein-containing complex	Sapur.006G150200 Sapur.018G081000 Sapur.001G112600 Sapur.002G028200 Sapur.002G116800 Sapur.002G189800 Sapur.003G063400 Sapur.005G028800 Sapur.005G180500 Sapur.006G194300 Sapur.008G051200 Sapur.008G051300 Sapur.008G142600 Sapur.009G009400 Sapur.010G051000 Sapur.010G115400 Sapur.014G044700 Sapur.016G218100 Sapur.019G022400 Sapur.T049800

GO:0005856	73	220	cytoskeleton	Sapur.001G000500 Sapur.001G086500 Sapur.002G015900 Sapur.002G090500 Sapur.002G122000 Sapur.002G146800 Sapur.002G159800 Sapur.002G182100 Sapur.003G028400 Sapur.003G090100 Sapur.003G091100 Sapur.003G166900 Sapur.003G169700 Sapur.004G039300 Sapur.004G120900 Sapur.004G122400 Sapur.004G130100 Sapur.005G015200 Sapur.005G070300 Sapur.005G156700 Sapur.005G192400 Sapur.006G025400 Sapur.006G086600 Sapur.006G120600 Sapur.006G159000 Sapur.007G012500 Sapur.007G043900 Sapur.008G139000 Sapur.008G147700 Sapur.008G161000 Sapur.009G018700 Sapur.009G052100 Sapur.009G052200 Sapur.009G066800 Sapur.009G097000 Sapur.010G008800 Sapur.010G044700 Sapur.010G164300 Sapur.010G206600 Sapur.011G046000 Sapur.011G091100 Sapur.011G097800 Sapur.011G108500 Sapur.011G119600 Sapur.012G020300 Sapur.013G010400 Sapur.013G017100 Sapur.014G014400 Sapur.014G034600 Sapur.014G114900 Sapur.016G030100 Sapur.016G054000 Sapur.016G056900 Sapur.016G077400 Sapur.016G094800 Sapur.016G112500 Sapur.016G127100 Sapur.016G129900 Sapur.016G130000 Sapur.016G158100 Sapur.016G158200 Sapur.016G172500 Sapur.016G201500 Sapur.016G265600 Sapur.016G275100 Sapur.016G302200 Sapur.016G302700 Sapur.017G045900 Sapur.017G125500 Sapur.018G096500 Sapur.019G035200 Sapur.15WG079100 Sapur.T001400
GO:0098588	32	73	bounding membrane of organelle	Sapur.001G037800 Sapur.002G118400 Sapur.003G100300 Sapur.003G137200 Sapur.005G058800 Sapur.006G150200 Sapur.014G048500 Sapur.016G098500 Sapur.017G027100 Sapur.018G081000 Sapur.002G116800 Sapur.004G026500 Sapur.004G115300 Sapur.005G121300 Sapur.006G062900 Sapur.006G223800 Sapur.007G051300 Sapur.009G009400 Sapur.009G057100 Sapur.009G091000 Sapur.010G146400 Sapur.010G149500 Sapur.010G177400 Sapur.011G034100 Sapur.013G064900 Sapur.014G044700 Sapur.016G163500 Sapur.016G175100 Sapur.016G204500 Sapur.018G006400 Sapur.019G097900 Sapur.15ZG071300
GO:0099081	23	44	supramolecular polymer	Sapur.001G086500 Sapur.002G015900 Sapur.002G090500 Sapur.002G146800 Sapur.003G090100 Sapur.003G091100 Sapur.003G166900 Sapur.005G192400 Sapur.006G025400 Sapur.009G052100 Sapur.009G052200 Sapur.009G066800 Sapur.011G119600 Sapur.016G030100 Sapur.016G094800 Sapur.016G158100 Sapur.016G158200 Sapur.016G172500 Sapur.016G302200 Sapur.016G302700 Sapur.017G125500 Sapur.019G035200 Sapur.15WG079100
GO:0099512	23	44	supramolecular fiber	Sapur.001G086500 Sapur.002G015900 Sapur.002G090500 Sapur.002G146800 Sapur.003G090100 Sapur.003G091100 Sapur.003G166900 Sapur.005G192400 Sapur.006G025400 Sapur.009G052100 Sapur.009G052200 Sapur.009G066800 Sapur.011G119600 Sapur.016G030100 Sapur.016G094800 Sapur.016G158100 Sapur.016G158200 Sapur.016G172500 Sapur.016G302200 Sapur.016G302700 Sapur.017G125500 Sapur.019G035200 Sapur.15WG079100
GO:0099513	23	44	polymeric cytoskeletal fiber	Sapur.001G086500 Sapur.002G015900 Sapur.002G090500 Sapur.002G146800 Sapur.003G090100 Sapur.003G091100 Sapur.003G166900 Sapur.005G192400 Sapur.006G025400 Sapur.009G052100 Sapur.009G052200 Sapur.009G066800 Sapur.011G119600 Sapur.016G030100 Sapur.016G094800 Sapur.016G158100 Sapur.016G158200 Sapur.016G172500 Sapur.016G302200 Sapur.016G302700 Sapur.017G125500 Sapur.019G035200 Sapur.15WG079100
GO:0005874	22	42	microtubule	Sapur.001G086500 Sapur.002G015900 Sapur.002G090500 Sapur.002G146800 Sapur.003G090100 Sapur.003G091100 Sapur.003G166900 Sapur.005G192400 Sapur.006G025400 Sapur.009G052100 Sapur.009G052200

				Sapur.009G066800 Sapur.011G119600 Sapur.016G030100 Sapur.016G094800 Sapur.016G158100 Sapur.016G158200 Sapur.016G172500 Sapur.016G302200 Sapur.016G302700 Sapur.017G125500 Sapur.019G035200
GO:0099080	23	46	supramolecular complex	Sapur.001G086500 Sapur.002G015900 Sapur.002G090500 Sapur.002G146800 Sapur.003G090100 Sapur.003G091100 Sapur.003G166900 Sapur.005G192400 Sapur.006G025400 Sapur.009G052100 Sapur.009G052200 Sapur.009G066800 Sapur.011G119600 Sapur.016G030100 Sapur.016G094800 Sapur.016G158100 Sapur.016G158200 Sapur.016G172500 Sapur.016G302200 Sapur.016G302700 Sapur.017G125500 Sapur.019G035200 Sapur.15WG079100
GO:0030660	12	16	Golgi-associated vesicle membrane	Sapur.004G026500 Sapur.004G115300 Sapur.006G223800 Sapur.009G057100 Sapur.009G091000 Sapur.010G146400 Sapur.010G149500 Sapur.011G034100 Sapur.016G163500 Sapur.018G006400 Sapur.019G097900 Sapur.15ZG071300
GO:0005798	12	16	Golgi-associated vesicle	Sapur.004G026500 Sapur.004G115300 Sapur.006G223800 Sapur.009G057100 Sapur.009G091000 Sapur.010G146400 Sapur.010G149500 Sapur.011G034100 Sapur.016G163500 Sapur.018G006400 Sapur.019G097900 Sapur.15ZG071300
GO:0030117	22	47	membrane coat	Sapur.004G026500 Sapur.004G115300 Sapur.006G223800 Sapur.007G023000 Sapur.009G057100 Sapur.009G091000 Sapur.010G146400 Sapur.010G149500 Sapur.010G159200 Sapur.011G034100 Sapur.016G163500 Sapur.018G006400 Sapur.019G097900 Sapur.15ZG071300 Sapur.001G082900 Sapur.001G154800 Sapur.003G029700 Sapur.003G094300 Sapur.006G076200 Sapur.006G201300 Sapur.15WG044900 Sapur.15ZG049100
GO:0048475	22	47	coated membrane	Sapur.004G026500 Sapur.004G115300 Sapur.006G223800 Sapur.007G023000 Sapur.009G057100 Sapur.009G091000 Sapur.010G146400 Sapur.010G149500 Sapur.010G159200 Sapur.011G034100 Sapur.016G163500 Sapur.018G006400 Sapur.019G097900 Sapur.15ZG071300 Sapur.001G082900 Sapur.001G154800 Sapur.003G029700 Sapur.003G094300 Sapur.006G076200 Sapur.006G201300 Sapur.15WG044900 Sapur.15ZG049100
GO:0030118	11	16	clathrin coat	Sapur.004G026500 Sapur.009G057100 Sapur.010G146400 Sapur.010G149500 Sapur.011G034100 Sapur.016G163500 Sapur.019G097900 Sapur.001G082900 Sapur.003G094300 Sapur.006G076200 Sapur.006G201300
GO:0015629	20	46	actin cytoskeleton	Sapur.004G130100 Sapur.005G070300 Sapur.005G156700 Sapur.006G120600 Sapur.008G139000 Sapur.008G147700 Sapur.010G044700 Sapur.010G164300 Sapur.011G091100 Sapur.012G020300 Sapur.013G017100 Sapur.014G034600 Sapur.014G114900 Sapur.016G129900 Sapur.016G130000 Sapur.016G201500 Sapur.016G265600 Sapur.017G045900 Sapur.018G096500 Sapur.15WG079100
GO:0015630	49	170	microtubule cytoskeleton	Sapur.001G000500 Sapur.001G086500 Sapur.002G015900 Sapur.002G090500 Sapur.002G122000 Sapur.002G146800 Sapur.002G159800 Sapur.002G182100 Sapur.003G028400 Sapur.003G090100 Sapur.003G091100 Sapur.003G166900 Sapur.003G169700 Sapur.004G120900 Sapur.004G122400 Sapur.005G015200 Sapur.005G192400 Sapur.006G025400 Sapur.006G159000 Sapur.007G012500 Sapur.007G043900 Sapur.008G161000 Sapur.009G018700 Sapur.009G052100 Sapur.009G052200 Sapur.009G066800 Sapur.009G097000 Sapur.010G008800 Sapur.010G206600 Sapur.011G097800 Sapur.011G108500 Sapur.011G119600 Sapur.013G010400 Sapur.014G014400 Sapur.016G030100 Sapur.016G054000 Sapur.016G056900 Sapur.016G077400 Sapur.016G094800 Sapur.016G127100 Sapur.016G158100

				Sapur.016G158200 Sapur.016G172500 Sapur.016G275100 Sapur.016G302200 Sapur.016G302700 Sapur.017G125500 Sapur.019G035200 Sapur.T001400
GO:0000139	8	10	Golgi membrane	Sapur.001G037800 Sapur.002G118400 Sapur.003G100300 Sapur.003G137200 Sapur.005G058800 Sapur.014G048500 Sapur.016G098500 Sapur.017G027100
GO:0005801	8	11	cis-Golgi network	Sapur.001G037800 Sapur.002G118400 Sapur.003G137200 Sapur.003G139000 Sapur.009G054300 Sapur.009G109200 Sapur.010G108800 Sapur.014G048500
GO:0098590	7	9	plasma membrane region	Sapur.004G026500 Sapur.009G057100 Sapur.010G146400 Sapur.010G149500 Sapur.011G034100 Sapur.016G163500 Sapur.019G097900
GO:0030125	7	9	clathrin vesicle coat	Sapur.004G026500 Sapur.009G057100 Sapur.010G146400 Sapur.010G149500 Sapur.011G034100 Sapur.016G163500 Sapur.019G097900
GO:0005905	7	9	clathrin-coated pit	Sapur.004G026500 Sapur.009G057100 Sapur.010G146400 Sapur.010G149500 Sapur.011G034100 Sapur.016G163500 Sapur.019G097900
GO:0030140	7	9	trans-Golgi network transport vesicle	Sapur.004G026500 Sapur.009G057100 Sapur.010G146400 Sapur.010G149500 Sapur.011G034100 Sapur.016G163500 Sapur.019G097900
GO:0030130	7	9	clathrin coat of trans-Golgi network vesicle	Sapur.004G026500 Sapur.009G057100 Sapur.010G146400 Sapur.010G149500 Sapur.011G034100 Sapur.016G163500 Sapur.019G097900
GO:0030132	7	9	clathrin coat of coated pit	Sapur.004G026500 Sapur.009G057100 Sapur.010G146400 Sapur.010G149500 Sapur.011G034100 Sapur.016G163500 Sapur.019G097900
GO:0030665	7	9	clathrin-coated vesicle membrane	Sapur.004G026500 Sapur.009G057100 Sapur.010G146400 Sapur.010G149500 Sapur.011G034100 Sapur.016G163500 Sapur.019G097900
GO:0030136	7	9	clathrin-coated vesicle	Sapur.004G026500 Sapur.009G057100 Sapur.010G146400 Sapur.010G149500 Sapur.011G034100 Sapur.016G163500 Sapur.019G097900
GO:0012510	7	9	trans-Golgi network transport vesicle membrane	Sapur.004G026500 Sapur.009G057100 Sapur.010G146400 Sapur.010G149500 Sapur.011G034100 Sapur.016G163500 Sapur.019G097900
GO:0005740	21	57	mitochondrial envelope	Sapur.001G144500 Sapur.001G187900 Sapur.003G020000 Sapur.004G050300 Sapur.004G142200 Sapur.005G121300 Sapur.005G199000 Sapur.006G062900 Sapur.007G006700 Sapur.007G051300 Sapur.008G142900 Sapur.009G118300 Sapur.010G173300 Sapur.010G179000 Sapur.010G202000 Sapur.012G071300 Sapur.014G123600 Sapur.016G175100 Sapur.016G204500 Sapur.016G258100 Sapur.018G041100
GO:0017119	5	5	Golgi transport complex	Sapur.002G046500 Sapur.003G074700 Sapur.005G160500 Sapur.006G185800 Sapur.T080400

GO:0005791	5	5	rough endoplasmic reticulum	Sapur.006G150200 Sapur.018G081000 Sapur.002G116800 Sapur.009G009400 Sapur.014G044700
GO:0030867	5	5	rough endoplasmic reticulum membrane	Sapur.006G150200 Sapur.018G081000 Sapur.002G116800 Sapur.009G009400 Sapur.014G044700
GO:0005885	8	12	Arp2/3 protein complex	Sapur.008G139000 Sapur.008G147700 Sapur.010G044700 Sapur.014G034600 Sapur.014G114900 Sapur.016G201500 Sapur.017G045900 Sapur.018G096500
GO:0031966	19	53	mitochondrial membrane	Sapur.001G187900 Sapur.003G020000 Sapur.004G050300 Sapur.004G142200 Sapur.005G121300 Sapur.005G199000 Sapur.006G062900 Sapur.007G006700 Sapur.007G051300 Sapur.008G142900 Sapur.009G118300 Sapur.010G173300 Sapur.010G179000 Sapur.010G202000 Sapur.012G071300 Sapur.014G123600 Sapur.016G175100 Sapur.016G204500 Sapur.018G041100
GO:0033176	7	11	proton-transporting V-type ATPase complex	Sapur.010G177400 Sapur.013G064900 Sapur.002G021300 Sapur.005G186900 Sapur.017G065500 Sapur.T003500 Sapur.T012400
GO:0098798	13	31	mitochondrial protein-containing complex	Sapur.001G187900 Sapur.003G020000 Sapur.004G142200 Sapur.005G199000 Sapur.006G062900 Sapur.007G006700 Sapur.008G142900 Sapur.009G118300 Sapur.010G173300 Sapur.010G202000 Sapur.014G123600 Sapur.016G204500 Sapur.018G041100
GO:0008303	4	4	caspase complex	Sapur.002G189800 Sapur.016G218100 Sapur.019G022400 Sapur.T049800
GO:0042765	4	4	GPI-anchor transamidase complex	Sapur.002G189800 Sapur.016G218100 Sapur.019G022400 Sapur.T049800
GO:0008250	4	4	oligosaccharyltransferase complex	Sapur.001G112600 Sapur.002G028200 Sapur.003G063400 Sapur.005G180500
GO:0005787	6	9	signal peptidase complex	Sapur.005G028800 Sapur.006G194300 Sapur.008G051200 Sapur.008G051300 Sapur.008G142600 Sapur.010G051000
GO:0030120	12	29	vesicle coat	Sapur.004G026500 Sapur.004G115300 Sapur.006G223800 Sapur.009G057100 Sapur.009G091000 Sapur.010G146400 Sapur.010G149500 Sapur.011G034100 Sapur.016G163500 Sapur.018G006400 Sapur.019G097900 Sapur.15ZG071300
GO:0030662	12	29	coated vesicle membrane	Sapur.004G026500 Sapur.004G115300 Sapur.006G223800 Sapur.009G057100 Sapur.009G091000 Sapur.010G146400 Sapur.010G149500 Sapur.011G034100 Sapur.016G163500 Sapur.018G006400 Sapur.019G097900 Sapur.15ZG071300
GO:003	12	29	coated vesicle	Sapur.004G026500 Sapur.004G115300 Sapur.006G223800 Sapur.009G057100 Sapur.009G091000

0135				Sapur.010G146400 Sapur.010G149500 Sapur.011G034100 Sapur.016G163500 Sapur.018G006400 Sapur.019G097900 Sapur.15ZG071300
GO:0030659	12	29	cytoplasmic vesicle membrane	Sapur.004G026500 Sapur.004G115300 Sapur.006G223800 Sapur.009G057100 Sapur.009G091000 Sapur.010G146400 Sapur.010G149500 Sapur.011G034100 Sapur.016G163500 Sapur.018G006400 Sapur.019G097900 Sapur.15ZG071300
GO:0031982	12	29	vesicle	Sapur.004G026500 Sapur.004G115300 Sapur.006G223800 Sapur.009G057100 Sapur.009G091000 Sapur.010G146400 Sapur.010G149500 Sapur.011G034100 Sapur.016G163500 Sapur.018G006400 Sapur.019G097900 Sapur.15ZG071300
GO:0097708	12	29	intracellular vesicle	Sapur.004G026500 Sapur.004G115300 Sapur.006G223800 Sapur.009G057100 Sapur.009G091000 Sapur.010G146400 Sapur.010G149500 Sapur.011G034100 Sapur.016G163500 Sapur.018G006400 Sapur.019G097900 Sapur.15ZG071300
GO:0031410	12	29	cytoplasmic vesicle	Sapur.004G026500 Sapur.004G115300 Sapur.006G223800 Sapur.009G057100 Sapur.009G091000 Sapur.010G146400 Sapur.010G149500 Sapur.011G034100 Sapur.016G163500 Sapur.018G006400 Sapur.019G097900 Sapur.15ZG071300
GO:0012506	12	29	vesicle membrane	Sapur.004G026500 Sapur.004G115300 Sapur.006G223800 Sapur.009G057100 Sapur.009G091000 Sapur.010G146400 Sapur.010G149500 Sapur.011G034100 Sapur.016G163500 Sapur.018G006400 Sapur.019G097900 Sapur.15ZG071300
GO:0019866	13	33	organelle inner membrane	Sapur.001G187900 Sapur.003G020000 Sapur.004G050300 Sapur.004G142200 Sapur.005G199000 Sapur.007G006700 Sapur.008G142900 Sapur.009G118300 Sapur.010G173300 Sapur.010G202000 Sapur.012G071300 Sapur.014G123600 Sapur.018G041100
GO:0005743	13	33	mitochondrial inner membrane	Sapur.001G187900 Sapur.003G020000 Sapur.004G050300 Sapur.004G142200 Sapur.005G199000 Sapur.007G006700 Sapur.008G142900 Sapur.009G118300 Sapur.010G173300 Sapur.010G202000 Sapur.012G071300 Sapur.014G123600 Sapur.018G041100
GO:0030126	5	7	COPI vesicle coat	Sapur.004G115300 Sapur.006G223800 Sapur.009G091000 Sapur.018G006400 Sapur.15ZG071300
GO:0030663	5	7	COPI-coated vesicle membrane	Sapur.004G115300 Sapur.006G223800 Sapur.009G091000 Sapur.018G006400 Sapur.15ZG071300
GO:0030137	5	7	COPI-coated vesicle	Sapur.004G115300 Sapur.006G223800 Sapur.009G091000 Sapur.018G006400 Sapur.15ZG071300
GO:0033179	5	7	proton-transporting V-type ATPase, V0 domain	Sapur.002G021300 Sapur.005G186900 Sapur.017G065500 Sapur.T003500 Sapur.T012400
GO:009	11	27	inner	Sapur.001G187900 Sapur.003G020000 Sapur.004G142200 Sapur.005G199000 Sapur.007G006700

8800			mitochondrial membrane protein complex	Sapur.008G142900 Sapur.009G118300 Sapur.010G173300 Sapur.010G202000 Sapur.014G123600 Sapur.018G041100
GO:0070469	6	11	respirasome	Sapur.001G187900 Sapur.003G020000 Sapur.004G142200 Sapur.005G199000 Sapur.007G006700 Sapur.009G118300
GO:0005746	6	11	mitochondrial respirasome	Sapur.001G187900 Sapur.003G020000 Sapur.004G142200 Sapur.005G199000 Sapur.007G006700 Sapur.009G118300
GO:0030119	6	11	AP-type membrane coat adaptor complex	Sapur.007G023000 Sapur.010G159200 Sapur.001G082900 Sapur.003G094300 Sapur.006G076200 Sapur.006G201300
GO:0098803	6	11	respiratory chain complex	Sapur.001G187900 Sapur.003G020000 Sapur.004G142200 Sapur.005G199000 Sapur.007G006700 Sapur.009G118300
GO:0071256	3	3	translocon complex	Sapur.002G116800 Sapur.009G009400 Sapur.014G044700
GO:0005784	3	3	Sec61 translocon complex	Sapur.002G116800 Sapur.009G009400 Sapur.014G044700
GO:0016469	14	42	proton-transporting two-sector ATPase complex	Sapur.003G054000 Sapur.010G173300 Sapur.010G177400 Sapur.010G202000 Sapur.013G064900 Sapur.018G041100 Sapur.002G021300 Sapur.005G186900 Sapur.010G205000 Sapur.013G047900 Sapur.017G065500 Sapur.T003500 Sapur.T012400 Sapur.T031900
GO:1905368	18	60	peptidase complex	Sapur.001G135000 Sapur.002G189800 Sapur.003G105400 Sapur.004G130500 Sapur.004G173100 Sapur.005G028800 Sapur.006G115800 Sapur.006G194300 Sapur.008G051200 Sapur.008G051300 Sapur.008G142600 Sapur.009G106500 Sapur.010G051000 Sapur.012G086400 Sapur.016G218100 Sapur.019G022400 Sapur.T049800 Sapur.006G063300
GO:0031967	21	74	organelle envelope	Sapur.001G144500 Sapur.001G187900 Sapur.003G020000 Sapur.004G050300 Sapur.004G142200 Sapur.005G121300 Sapur.005G199000 Sapur.006G062900 Sapur.007G006700 Sapur.007G051300 Sapur.008G142900 Sapur.009G118300 Sapur.010G173300 Sapur.010G179000 Sapur.010G202000 Sapur.012G071300 Sapur.014G123600 Sapur.016G175100 Sapur.016G204500 Sapur.016G258100 Sapur.018G041100
GO:0031975	21	74	envelope	Sapur.001G144500 Sapur.001G187900 Sapur.003G020000 Sapur.004G050300 Sapur.004G142200 Sapur.005G121300 Sapur.005G199000 Sapur.006G062900 Sapur.007G006700 Sapur.007G051300 Sapur.008G142900 Sapur.009G118300 Sapur.010G173300 Sapur.010G179000 Sapur.010G202000 Sapur.012G071300 Sapur.014G123600 Sapur.016G175100 Sapur.016G204500 Sapur.016G258100 Sapur.018G041100
GO:0033177	7	16	proton-transporting two-sector	Sapur.010G173300 Sapur.018G041100 Sapur.002G021300 Sapur.005G186900 Sapur.017G065500 Sapur.T003500 Sapur.T012400

GO:0098797	7	16	ATPase complex, proton-transporting domain plasma membrane protein complex	Sapur.004G026500 Sapur.009G057100 Sapur.010G146400 Sapur.010G149500 Sapur.011G034100 Sapur.016G163500 Sapur.019G097900
GO:0030131	4	7	clathrin adaptor complex	Sapur.001G082900 Sapur.003G094300 Sapur.006G076200 Sapur.006G201300
GO:1902495	5	11	transmembrane transporter complex	Sapur.001G187900 Sapur.003G020000 Sapur.004G142200 Sapur.005G199000 Sapur.009G118300
GO:1990351	5	11	transporter complex	Sapur.001G187900 Sapur.003G020000 Sapur.004G142200 Sapur.005G199000 Sapur.009G118300
GO:0008290	2	2	F-actin capping protein complex	Sapur.005G156700 Sapur.013G017100
GO:0005785	2	2	signal recognition particle receptor complex	Sapur.006G150200 Sapur.018G081000
GO:0110165	522	3209	cellular anatomical entity	Sapur.001G037800 Sapur.001G088000 Sapur.001G091700 Sapur.001G091900 Sapur.001G156100 Sapur.002G082900 Sapur.002G118400 Sapur.002G199900 Sapur.003G036600 Sapur.003G100300 Sapur.003G137200 Sapur.003G137500 Sapur.003G157700 Sapur.004G140700 Sapur.005G058800 Sapur.005G094400 Sapur.005G094500 Sapur.005G107600 Sapur.005G124800 Sapur.005G173300 Sapur.006G025500 Sapur.006G083100 Sapur.006G107100 Sapur.006G150200 Sapur.006G168600 Sapur.006G211000 Sapur.007G039500 Sapur.007G040800 Sapur.007G123900 Sapur.008G130800 Sapur.008G173100 Sapur.009G052900 Sapur.009G067900 Sapur.010G068800 Sapur.011G026600 Sapur.012G013000 Sapur.013G132900 Sapur.014G004600 Sapur.014G048500 Sapur.014G077700 Sapur.014G099300 Sapur.016G083000 Sapur.016G098500 Sapur.017G027100 Sapur.018G081000 Sapur.018G100000 Sapur.15WG068500 Sapur.15WG080800 Sapur.15ZG010600 Sapur.001G009500 Sapur.001G009600 Sapur.001G030600 Sapur.001G033600 Sapur.001G037600 Sapur.001G043500 Sapur.001G046700 Sapur.001G089300 Sapur.001G110200 Sapur.001G146600 Sapur.001G164900 Sapur.001G185400 Sapur.001G185600 Sapur.001G187000 Sapur.001G187900 Sapur.002G004300 Sapur.002G006100 Sapur.002G038200 Sapur.002G044500 Sapur.002G047800 Sapur.002G063400 Sapur.002G072000 Sapur.002G086200 Sapur.002G092100 Sapur.002G096700 Sapur.002G098200 Sapur.002G108400 Sapur.002G110800 Sapur.002G137300 Sapur.002G138300 Sapur.002G139100 Sapur.002G161300 Sapur.002G176700 Sapur.002G176800 Sapur.002G181500 Sapur.002G188000 Sapur.002G190100 Sapur.002G193600 Sapur.002G202100 Sapur.003G020000

Sapur.003G029600	Sapur.003G034800	Sapur.003G068500	Sapur.003G076300	Sapur.003G076900	Sapur.003G082000
Sapur.003G086600	Sapur.003G092800	Sapur.003G095000	Sapur.003G099600	Sapur.003G138600	Sapur.003G141500
Sapur.003G149100	Sapur.004G005200	Sapur.004G007800	Sapur.004G034200	Sapur.004G070600	Sapur.004G102500
Sapur.004G103700	Sapur.004G104700	Sapur.004G116800	Sapur.004G132700	Sapur.004G141700	Sapur.004G143900
Sapur.004G168100	Sapur.004G168900	Sapur.005G013100	Sapur.005G027800	Sapur.005G044700	Sapur.005G086200
Sapur.005G125100	Sapur.005G156500	Sapur.005G180700	Sapur.005G201800	Sapur.005G204000	Sapur.006G008700
Sapur.006G008800	Sapur.006G012300	Sapur.006G023800	Sapur.006G049200	Sapur.006G050300	Sapur.006G054000
Sapur.006G061500	Sapur.006G062000	Sapur.006G067100	Sapur.006G067800	Sapur.006G084000	Sapur.006G105100
Sapur.006G105200	Sapur.006G107800	Sapur.006G192900	Sapur.006G198200	Sapur.006G198700	Sapur.006G206200
Sapur.006G212700	Sapur.006G217000	Sapur.006G217200	Sapur.006G223800	Sapur.007G044700	Sapur.007G066700
Sapur.008G003400	Sapur.008G004000	Sapur.008G007500	Sapur.008G011900	Sapur.008G028500	Sapur.008G038400
Sapur.008G043100	Sapur.008G065200	Sapur.008G070800	Sapur.008G103100	Sapur.008G105500	Sapur.008G108600
Sapur.008G113200	Sapur.008G114600	Sapur.008G126700	Sapur.008G149700	Sapur.008G162700	Sapur.009G006300
Sapur.009G019400	Sapur.009G062300	Sapur.009G063600	Sapur.009G067000	Sapur.009G068000	Sapur.009G079300
Sapur.009G079600	Sapur.009G099500	Sapur.009G109000	Sapur.009G117600	Sapur.009G120200	Sapur.009G130600
Sapur.010G007300	Sapur.010G023600	Sapur.010G042500	Sapur.010G045000	Sapur.010G048300	Sapur.010G073400
Sapur.010G081400	Sapur.010G087400	Sapur.010G090500	Sapur.010G092800	Sapur.010G098800	Sapur.010G105000
Sapur.010G108800	Sapur.010G115400	Sapur.010G118100	Sapur.010G124000	Sapur.010G129800	Sapur.010G134800
Sapur.010G150300	Sapur.010G160600	Sapur.010G165600	Sapur.010G178800	Sapur.010G199900	Sapur.010G203100
Sapur.010G205600	Sapur.011G003200	Sapur.011G006300	Sapur.011G008300	Sapur.011G036400	Sapur.011G041500
Sapur.011G050700	Sapur.011G054900	Sapur.011G071500	Sapur.011G075900	Sapur.011G101900	Sapur.011G112000
Sapur.012G025200	Sapur.012G028100	Sapur.012G052600	Sapur.012G060700	Sapur.012G065200	Sapur.012G083500
Sapur.013G008900	Sapur.013G013900	Sapur.013G023800	Sapur.013G024200	Sapur.013G064300	Sapur.013G068300
Sapur.013G080100	Sapur.013G090600	Sapur.013G091200	Sapur.013G100800	Sapur.013G101200	Sapur.013G105300
Sapur.013G109400	Sapur.013G121500	Sapur.014G009000	Sapur.014G018800	Sapur.014G030900	Sapur.014G062000
Sapur.014G092800	Sapur.014G098300	Sapur.014G105300	Sapur.014G125100	Sapur.014G128700	Sapur.016G010700
Sapur.016G010900	Sapur.016G047900	Sapur.016G056800	Sapur.016G082300	Sapur.016G096000	Sapur.016G097500
Sapur.016G109800	Sapur.016G111800	Sapur.016G113700	Sapur.016G127900	Sapur.016G147600	Sapur.016G198200
Sapur.016G203700	Sapur.016G221000	Sapur.016G227000	Sapur.016G229400	Sapur.016G231500	Sapur.016G231600
Sapur.016G242000	Sapur.016G244800	Sapur.016G248500	Sapur.016G252400	Sapur.016G290200	Sapur.016G310000
Sapur.017G016100	Sapur.017G052500	Sapur.017G056200	Sapur.017G062700	Sapur.017G069700	Sapur.017G073700
Sapur.017G114600	Sapur.018G005200	Sapur.018G006400	Sapur.018G009200	Sapur.018G015400	Sapur.018G020900
Sapur.018G026100	Sapur.018G026500	Sapur.018G031600	Sapur.018G032300	Sapur.018G053400	Sapur.018G062800

Sapur.018G069900 Sapur.018G075900 Sapur.018G083400 Sapur.018G094800 Sapur.018G096200 Sapur.018G097400
Sapur.018G108000 Sapur.018G110200 Sapur.019G035900 Sapur.019G076600 Sapur.019G079900 Sapur.019G099900
Sapur.15WG064400 Sapur.15WG081100 Sapur.15ZG001200 Sapur.15ZG004600 Sapur.15ZG018200 Sapur.15ZG021300
Sapur.15ZG052500 Sapur.15ZG059400 Sapur.15ZG072800 Sapur.15ZG073700 Sapur.15ZG079800 Sapur.15ZG093800
Sapur.15ZG109000 Sapur.15ZG111000 Sapur.T001500 Sapur.T052300 Sapur.T064000 Sapur.T140400
Sapur.001G000500 Sapur.001G046900 Sapur.001G069700 Sapur.001G081000 Sapur.001G086500 Sapur.001G112600
Sapur.001G126200 Sapur.001G135000 Sapur.001G139800 Sapur.001G144500 Sapur.002G013700 Sapur.002G015900
Sapur.002G019800 Sapur.002G028200 Sapur.002G046500 Sapur.002G053800 Sapur.002G090500 Sapur.002G116800
Sapur.002G122000 Sapur.002G146800 Sapur.002G159800 Sapur.002G182100 Sapur.002G189800 Sapur.003G028400
Sapur.003G051000 Sapur.003G054000 Sapur.003G060700 Sapur.003G062100 Sapur.003G063400 Sapur.003G074700
Sapur.003G090100 Sapur.003G091100 Sapur.003G105400 Sapur.003G107200 Sapur.003G127800 Sapur.003G139000
Sapur.003G166900 Sapur.003G169700 Sapur.004G020700 Sapur.004G026500 Sapur.004G039300 Sapur.004G050300
Sapur.004G095900 Sapur.004G115300 Sapur.004G120900 Sapur.004G122400 Sapur.004G127400 Sapur.004G130100
Sapur.004G130500 Sapur.004G142200 Sapur.004G173100 Sapur.005G015200 Sapur.005G021200 Sapur.005G028800
Sapur.005G055200 Sapur.005G070300 Sapur.005G101600 Sapur.005G121300 Sapur.005G156700 Sapur.005G160500
Sapur.005G180500 Sapur.005G192400 Sapur.005G199000 Sapur.006G025400 Sapur.006G062900 Sapur.006G086600
Sapur.006G115800 Sapur.006G120600 Sapur.006G159000 Sapur.006G167000 Sapur.006G185800 Sapur.006G194300
Sapur.007G006700 Sapur.007G012500 Sapur.007G023000 Sapur.007G028100 Sapur.007G043900 Sapur.007G051300
Sapur.008G021400 Sapur.008G051200 Sapur.008G051300 Sapur.008G076300 Sapur.008G139000 Sapur.008G142600
Sapur.008G142900 Sapur.008G147700 Sapur.008G161000 Sapur.009G009400 Sapur.009G018700 Sapur.009G052100
Sapur.009G052200 Sapur.009G054300 Sapur.009G057100 Sapur.009G066800 Sapur.009G091000 Sapur.009G097000
Sapur.009G103300 Sapur.009G106500 Sapur.009G109200 Sapur.009G118300 Sapur.010G008800 Sapur.010G044700
Sapur.010G051000 Sapur.010G144600 Sapur.010G146400 Sapur.010G149500 Sapur.010G159200 Sapur.010G164300
Sapur.010G173300 Sapur.010G177400 Sapur.010G179000 Sapur.010G195700 Sapur.010G202000 Sapur.010G206600
Sapur.011G034100 Sapur.011G046000 Sapur.011G091100 Sapur.011G097800 Sapur.011G108500 Sapur.011G119600
Sapur.012G020300 Sapur.012G071300 Sapur.012G085200 Sapur.012G086400 Sapur.012G098100 Sapur.013G010400
Sapur.013G017100 Sapur.013G030300 Sapur.013G064900 Sapur.013G066200 Sapur.013G066500 Sapur.013G109100
Sapur.014G014400 Sapur.014G034600 Sapur.014G040300 Sapur.014G043900 Sapur.014G044700 Sapur.014G079400
Sapur.014G114900 Sapur.014G123600 Sapur.014G136300 Sapur.016G030100 Sapur.016G054000 Sapur.016G056900
Sapur.016G065300 Sapur.016G077400 Sapur.016G094800 Sapur.016G112500 Sapur.016G127100 Sapur.016G129900
Sapur.016G130000 Sapur.016G158100 Sapur.016G158200 Sapur.016G163500 Sapur.016G172500 Sapur.016G175100
Sapur.016G201500 Sapur.016G204500 Sapur.016G218100 Sapur.016G258100 Sapur.016G265600 Sapur.016G275100
Sapur.016G275200 Sapur.016G302200 Sapur.016G302700 Sapur.017G032000 Sapur.017G045900 Sapur.017G078400

				Sapur.017G125500 Sapur.018G031900 Sapur.018G041100 Sapur.018G096500 Sapur.019G022400 Sapur.019G035200 Sapur.019G097900 Sapur.15WG076900 Sapur.15WG079100 Sapur.15ZG063600 Sapur.15ZG071300 Sapur.15ZG110700 Sapur.T001400 Sapur.T049800 Sapur.T080400 Sapur.001G082900 Sapur.001G154800 Sapur.003G029700 Sapur.003G094300 Sapur.005G174600 Sapur.006G076200 Sapur.006G201300 Sapur.012G030400 Sapur.15WG044900 Sapur.15ZG029200 Sapur.15ZG029900 Sapur.15ZG031600 Sapur.15ZG032200 Sapur.15ZG049100 Sapur.002G021300 Sapur.005G186900 Sapur.010G205000 Sapur.013G047900 Sapur.017G065500 Sapur.T003500 Sapur.T012400 Sapur.T031900
GO:0070069	3	5	cytochrome complex	Sapur.004G142200 Sapur.007G006700 Sapur.009G118300
GO:0005871	24	103	kinesin complex	Sapur.001G000500 Sapur.002G122000 Sapur.002G159800 Sapur.002G182100 Sapur.003G028400 Sapur.003G169700 Sapur.004G120900 Sapur.004G122400 Sapur.005G015200 Sapur.006G159000 Sapur.007G012500 Sapur.007G043900 Sapur.009G018700 Sapur.009G097000 Sapur.010G008800 Sapur.011G097800 Sapur.011G108500 Sapur.013G010400 Sapur.014G014400 Sapur.016G054000 Sapur.016G056900 Sapur.016G077400 Sapur.016G127100 Sapur.016G275100
GO:0005875	27	120	microtubule associated complex	Sapur.001G000500 Sapur.002G122000 Sapur.002G159800 Sapur.002G182100 Sapur.003G028400 Sapur.003G169700 Sapur.004G120900 Sapur.004G122400 Sapur.005G015200 Sapur.006G159000 Sapur.007G012500 Sapur.007G043900 Sapur.008G161000 Sapur.009G018700 Sapur.009G097000 Sapur.010G008800 Sapur.010G206600 Sapur.011G097800 Sapur.011G108500 Sapur.013G010400 Sapur.014G014400 Sapur.016G054000 Sapur.016G056900 Sapur.016G077400 Sapur.016G127100 Sapur.016G275100 Sapur.T001400
GO:0032991	152	842	protein-containing complex	Sapur.006G150200 Sapur.018G081000 Sapur.001G000500 Sapur.001G112600 Sapur.001G135000 Sapur.001G167400 Sapur.001G187900 Sapur.002G013700 Sapur.002G019800 Sapur.002G028200 Sapur.002G046500 Sapur.002G053800 Sapur.002G116800 Sapur.002G122000 Sapur.002G146800 Sapur.002G159800 Sapur.002G182100 Sapur.002G189800 Sapur.003G020000 Sapur.003G028400 Sapur.003G054000 Sapur.003G063400 Sapur.003G074700 Sapur.003G105400 Sapur.003G169700 Sapur.004G026500 Sapur.004G115300 Sapur.004G120900 Sapur.004G122400 Sapur.004G130500 Sapur.004G142200 Sapur.004G173100 Sapur.005G015200 Sapur.005G021200 Sapur.005G028800 Sapur.005G042300 Sapur.005G055200 Sapur.005G070300 Sapur.005G156700 Sapur.005G160500 Sapur.005G180500 Sapur.005G199000 Sapur.006G062900 Sapur.006G102300 Sapur.006G115800 Sapur.006G120600 Sapur.006G159000 Sapur.006G167000 Sapur.006G185800 Sapur.006G194300 Sapur.006G204500 Sapur.006G223800 Sapur.007G006700 Sapur.007G012500 Sapur.007G023000 Sapur.007G043900 Sapur.008G021400 Sapur.008G051200 Sapur.008G051300 Sapur.008G139000 Sapur.008G142600 Sapur.008G142900 Sapur.008G147700 Sapur.008G161000 Sapur.009G009400 Sapur.009G018700 Sapur.009G057100 Sapur.009G091000 Sapur.009G097000 Sapur.009G106500 Sapur.009G118300 Sapur.010G008800 Sapur.010G044700 Sapur.010G051000 Sapur.010G115400 Sapur.010G144600 Sapur.010G146400 Sapur.010G149500 Sapur.010G159200 Sapur.010G173300 Sapur.010G177400 Sapur.010G195700 Sapur.010G202000

					Sapur.010G206600 Sapur.011G034100 Sapur.011G091100 Sapur.011G097800 Sapur.011G106700 Sapur.011G108500 Sapur.012G020300 Sapur.012G023200 Sapur.012G085200 Sapur.012G086400 Sapur.013G010400 Sapur.013G017100 Sapur.013G064900 Sapur.013G109100 Sapur.014G014400 Sapur.014G034600 Sapur.014G044700 Sapur.014G114900 Sapur.014G123600 Sapur.016G054000 Sapur.016G056900 Sapur.016G065300 Sapur.016G077400 Sapur.016G088900 Sapur.016G127100 Sapur.016G163500 Sapur.016G201500 Sapur.016G204500 Sapur.016G218100 Sapur.016G265600 Sapur.016G275100 Sapur.017G045900 Sapur.018G006400 Sapur.018G028200 Sapur.018G031600 Sapur.018G041100 Sapur.018G096500 Sapur.019G022400 Sapur.019G097900 Sapur.15WG068500 Sapur.15ZG071300 Sapur.15ZG110700 Sapur.T001400 Sapur.T049800 Sapur.T080400 Sapur.001G082900 Sapur.001G154800 Sapur.003G029700 Sapur.003G094300 Sapur.005G174600 Sapur.006G063300 Sapur.006G076200 Sapur.006G201300 Sapur.012G030400 Sapur.15WG044900 Sapur.15ZG029200 Sapur.15ZG029900 Sapur.15ZG031600 Sapur.15ZG032200 Sapur.15ZG049100 Sapur.002G021300 Sapur.002G188000 Sapur.005G186900 Sapur.010G205000 Sapur.013G047900 Sapur.017G065500 Sapur.T003500 Sapur.T012400 Sapur.T031900
	GO:0005871	31	103	kinesin complex	Sapur.001G094200 Sapur.002G064800 Sapur.002G085200 Sapur.002G089300 Sapur.004G019100 Sapur.004G109700 Sapur.004G147000 Sapur.006G036800 Sapur.006G067400 Sapur.006G069500 Sapur.006G112500 Sapur.006G172300 Sapur.008G137200 Sapur.009G122200 Sapur.010G042700 Sapur.010G119100 Sapur.011G018500 Sapur.011G103700 Sapur.012G037400 Sapur.012G040700 Sapur.014G005100 Sapur.014G052300 Sapur.016G071200 Sapur.018G057600 Sapur.15WG040400 Sapur.15WG044400 Sapur.15ZG044100 Sapur.15ZG044300 Sapur.15ZG044900 Sapur.15ZG049500 Sapur.T003900
m_up_CC	GO:0005875	33	120	microtubule associated complex	Sapur.001G094200 Sapur.002G064800 Sapur.002G085200 Sapur.002G089300 Sapur.004G019100 Sapur.004G109700 Sapur.004G147000 Sapur.006G036800 Sapur.006G067400 Sapur.006G069500 Sapur.006G112500 Sapur.006G172300 Sapur.008G137200 Sapur.009G122200 Sapur.010G042700 Sapur.010G081900 Sapur.010G119100 Sapur.011G018500 Sapur.011G103700 Sapur.012G037400 Sapur.012G040700 Sapur.014G005100 Sapur.014G052300 Sapur.016G071200 Sapur.016G259400 Sapur.018G057600 Sapur.15WG040400 Sapur.15WG044400 Sapur.15ZG044100 Sapur.15ZG044300 Sapur.15ZG044900 Sapur.15ZG049500 Sapur.T003900
	GO:0015630	41	170	microtubule cytoskeleton	Sapur.001G094200 Sapur.002G064800 Sapur.002G085200 Sapur.002G089300 Sapur.004G019100 Sapur.004G071100 Sapur.004G109700 Sapur.004G147000 Sapur.006G036800 Sapur.006G067400 Sapur.006G069500 Sapur.006G112500 Sapur.006G172300 Sapur.007G123700 Sapur.008G137200 Sapur.009G029500 Sapur.009G122200 Sapur.010G042700 Sapur.010G081900 Sapur.010G119100 Sapur.011G018500 Sapur.011G103700 Sapur.012G037400 Sapur.012G040700 Sapur.012G099900 Sapur.014G005100 Sapur.014G052300 Sapur.016G071200 Sapur.016G259400 Sapur.017G006400 Sapur.017G006500 Sapur.017G067300 Sapur.018G057600 Sapur.15WG040400 Sapur.15WG044400 Sapur.15WG062900 Sapur.15ZG044100 Sapur.15ZG044300 Sapur.15ZG044900 Sapur.15ZG049500 Sapur.T003900
	GO:0019898	10	29	extrinsic component of	Sapur.002G044200 Sapur.004G019000 Sapur.005G007700 Sapur.005G020600 Sapur.005G103700 Sapur.005G205500 Sapur.007G031300 Sapur.009G048400 Sapur.010G165900 Sapur.011G018600

			membrane	
GO:0005856	44	220	cytoskeleton	Sapur.001G094200 Sapur.001G167000 Sapur.002G032100 Sapur.002G064800 Sapur.002G085200 Sapur.002G089300 Sapur.004G019100 Sapur.004G071100 Sapur.004G109700 Sapur.004G147000 Sapur.006G036800 Sapur.006G067400 Sapur.006G069500 Sapur.006G112500 Sapur.006G172300 Sapur.007G123700 Sapur.008G137200 Sapur.009G018000 Sapur.009G029500 Sapur.009G122200 Sapur.010G042700 Sapur.010G081900 Sapur.010G119100 Sapur.011G018500 Sapur.011G103700 Sapur.012G037400 Sapur.012G040700 Sapur.012G099900 Sapur.014G005100 Sapur.014G052300 Sapur.016G071200 Sapur.016G259400 Sapur.017G006400 Sapur.017G006500 Sapur.017G067300 Sapur.018G057600 Sapur.15WG040400 Sapur.15WG044400 Sapur.15WG062900 Sapur.15ZG044100 Sapur.15ZG044300 Sapur.15ZG044900 Sapur.15ZG049500 Sapur.T003900
GO:0005634	141	831	nucleus	Sapur.001G025400 Sapur.001G047800 Sapur.001G055600 Sapur.001G058800 Sapur.001G072500 Sapur.001G108900 Sapur.001G129000 Sapur.001G156400 Sapur.001G164600 Sapur.002G006500 Sapur.002G031600 Sapur.002G043300 Sapur.002G063700 Sapur.002G091700 Sapur.002G099000 Sapur.002G100000 Sapur.002G110600 Sapur.002G116100 Sapur.002G151100 Sapur.002G160400 Sapur.003G022200 Sapur.003G023600 Sapur.003G026500 Sapur.003G031000 Sapur.003G047900 Sapur.003G067100 Sapur.003G068400 Sapur.003G129600 Sapur.003G163200 Sapur.004G030900 Sapur.004G054800 Sapur.004G062200 Sapur.005G000100 Sapur.005G042000 Sapur.005G079500 Sapur.005G086700 Sapur.005G093100 Sapur.005G142500 Sapur.005G163900 Sapur.005G201400 Sapur.006G004100 Sapur.006G021400 Sapur.006G025200 Sapur.006G043000 Sapur.006G063200 Sapur.006G065100 Sapur.006G070000 Sapur.006G104700 Sapur.006G114200 Sapur.006G122700 Sapur.006G152600 Sapur.006G195200 Sapur.006G214400 Sapur.007G050900 Sapur.007G098200 Sapur.008G053100 Sapur.008G118600 Sapur.008G131800 Sapur.009G003100 Sapur.009G009700 Sapur.009G010000 Sapur.009G072700 Sapur.009G081200 Sapur.009G115000 Sapur.009G129900 Sapur.010G035800 Sapur.010G046500 Sapur.010G065900 Sapur.010G068700 Sapur.010G075900 Sapur.010G077600 Sapur.010G078000 Sapur.010G120000 Sapur.010G120300 Sapur.010G149100 Sapur.010G175000 Sapur.010G183500 Sapur.010G193700 Sapur.011G035500 Sapur.011G041100 Sapur.011G066500 Sapur.012G020900 Sapur.012G067900 Sapur.012G100600 Sapur.013G000100 Sapur.013G008000 Sapur.013G038800 Sapur.013G059000 Sapur.013G126600 Sapur.014G011600 Sapur.014G034500 Sapur.014G053000 Sapur.014G066300 Sapur.014G078500 Sapur.016G018700 Sapur.016G029900 Sapur.016G030200 Sapur.016G043500 Sapur.016G058600 Sapur.016G085100 Sapur.016G157600 Sapur.016G159000 Sapur.016G167800 Sapur.016G181200 Sapur.016G195300 Sapur.016G229800 Sapur.017G004800 Sapur.017G101800 Sapur.017G102100 Sapur.017G115700 Sapur.018G022700 Sapur.018G028100 Sapur.018G057700 Sapur.019G060100 Sapur.019G074500 Sapur.15WG063800 Sapur.15WG067500 Sapur.15WG072800 Sapur.15ZG040500 Sapur.15ZG043500 Sapur.15ZG044500 Sapur.15ZG080300 Sapur.15ZG080900 Sapur.15ZG103400 Sapur.T006300 Sapur.T012000 Sapur.T045800 Sapur.T058600 Sapur.002G183800 Sapur.003G016800 Sapur.003G136700 Sapur.003G155000 Sapur.006G118700 Sapur.006G121100 Sapur.007G110800 Sapur.008G055600 Sapur.010G169600 Sapur.011G056100 Sapur.013G054700 Sapur.014G065300 Sapur.017G027400

	GO:0009331	3	5	glycerol-3-phosphate dehydrogenase complex	Sapur.001G017800 Sapur.013G085300 Sapur.016G040000
	GO:0000786	12	47	nucleosome	Sapur.001G013800 Sapur.002G021500 Sapur.002G160400 Sapur.003G159400 Sapur.004G062200 Sapur.005G093100 Sapur.005G186700 Sapur.008G021500 Sapur.008G131800 Sapur.010G035800 Sapur.010G185200 Sapur.014G075400
	GO:0005576	4	10	extracellular region	Sapur.002G093900 Sapur.007G005100 Sapur.014G003500 Sapur.014G025900
	GO:0005819	6	19	spindle	Sapur.004G071100 Sapur.007G123700 Sapur.012G099900 Sapur.017G006400 Sapur.017G006500 Sapur.15WG062900
m_down_MF	GO:0003824	1207	7297	catalytic activity	Sapur.001G001400 Sapur.001G005700 Sapur.001G015800 Sapur.001G031800 Sapur.001G033500 Sapur.001G042400 Sapur.001G043100 Sapur.001G066800 Sapur.001G078000 Sapur.001G080400 Sapur.001G086500 Sapur.001G086800 Sapur.001G088500 Sapur.001G093700 Sapur.001G095200 Sapur.001G098400 Sapur.001G098800 Sapur.001G100800 Sapur.001G103900 Sapur.001G124800 Sapur.001G135000 Sapur.001G137500 Sapur.001G139800 Sapur.001G146600 Sapur.001G148700 Sapur.001G171200 Sapur.001G175000 Sapur.001G175900 Sapur.001G185700 Sapur.001G190300 Sapur.001G191000 Sapur.002G009400 Sapur.002G010500 Sapur.002G015900 Sapur.002G022900 Sapur.002G028000 Sapur.002G028100 Sapur.002G034900 Sapur.002G046900 Sapur.002G055700 Sapur.002G063400 Sapur.002G065200 Sapur.002G072700 Sapur.002G075000 Sapur.002G084000 Sapur.002G087900 Sapur.002G090500 Sapur.002G097300 Sapur.002G101500 Sapur.002G106900 Sapur.002G108400 Sapur.002G119600 Sapur.002G120400 Sapur.002G124000 Sapur.002G130600 Sapur.002G139100 Sapur.002G143900 Sapur.002G146800 Sapur.002G150900 Sapur.002G164000 Sapur.002G164400 Sapur.002G176000 Sapur.002G179500 Sapur.002G179600 Sapur.002G180800 Sapur.002G188100 Sapur.002G192000 Sapur.002G197600 Sapur.002G198100 Sapur.002G199800 Sapur.002G201200 Sapur.002G201300 Sapur.003G000800 Sapur.003G013500 Sapur.003G034800 Sapur.003G036900 Sapur.003G037300 Sapur.003G037900 Sapur.003G042700 Sapur.003G045000 Sapur.003G051100 Sapur.003G052500 Sapur.003G061600 Sapur.003G061800 Sapur.003G067600 Sapur.003G073200 Sapur.003G075500 Sapur.003G077900 Sapur.003G082200 Sapur.003G085300 Sapur.003G087600 Sapur.003G087900 Sapur.003G088100 Sapur.003G089700 Sapur.003G090100 Sapur.003G091100 Sapur.003G092600 Sapur.003G095700 Sapur.003G099700 Sapur.003G115200 Sapur.003G120000 Sapur.003G131900 Sapur.003G133000 Sapur.003G138700 Sapur.003G151400 Sapur.003G153700 Sapur.003G156300 Sapur.003G162900 Sapur.003G166900 Sapur.004G005000 Sapur.004G006100 Sapur.004G009000 Sapur.004G017100 Sapur.004G017500 Sapur.004G021200 Sapur.004G021600 Sapur.004G023900 Sapur.004G029200 Sapur.004G032900 Sapur.004G033000 Sapur.004G035300 Sapur.004G044400 Sapur.004G046500 Sapur.004G047000 Sapur.004G057300 Sapur.004G060000 Sapur.004G061000 Sapur.004G067800 Sapur.004G068600 Sapur.004G080700 Sapur.004G087900

Sapur.004G098500 Sapur.004G099400 Sapur.004G115800 Sapur.004G121200 Sapur.004G129800 Sapur.004G130500
Sapur.004G135100 Sapur.004G135900 Sapur.004G164900 Sapur.004G165000 Sapur.004G168300 Sapur.004G170800
Sapur.004G172200 Sapur.004G173100 Sapur.004G177200 Sapur.005G001600 Sapur.005G001800 Sapur.005G011700
Sapur.005G028800 Sapur.005G031100 Sapur.005G039800 Sapur.005G040300 Sapur.005G047400 Sapur.005G047600
Sapur.005G050800 Sapur.005G053000 Sapur.005G057200 Sapur.005G062300 Sapur.005G065000 Sapur.005G065300
Sapur.005G081600 Sapur.005G085600 Sapur.005G086400 Sapur.005G086600 Sapur.005G086800 Sapur.005G101300
Sapur.005G106500 Sapur.005G109200 Sapur.005G122400 Sapur.005G123400 Sapur.005G140600 Sapur.005G140700
Sapur.005G143900 Sapur.005G148400 Sapur.005G153800 Sapur.005G157000 Sapur.005G160000 Sapur.005G180900
Sapur.005G185800 Sapur.005G192400 Sapur.005G194900 Sapur.005G196400 Sapur.005G198500 Sapur.005G202000
Sapur.005G203500 Sapur.005G205600 Sapur.005G208100 Sapur.006G025400 Sapur.006G030600 Sapur.006G037500
Sapur.006G039400 Sapur.006G043700 Sapur.006G044000 Sapur.006G059800 Sapur.006G061500 Sapur.006G063800
Sapur.006G064600 Sapur.006G070700 Sapur.006G071400 Sapur.006G071900 Sapur.006G077000 Sapur.006G079700
Sapur.006G079800 Sapur.006G088300 Sapur.006G090100 Sapur.006G093700 Sapur.006G096300 Sapur.006G102100
Sapur.006G106900 Sapur.006G107000 Sapur.006G110900 Sapur.006G115800 Sapur.006G117000 Sapur.006G124800
Sapur.006G130400 Sapur.006G137500 Sapur.006G141900 Sapur.006G150200 Sapur.006G151400 Sapur.006G161800
Sapur.006G164600 Sapur.006G169900 Sapur.006G180500 Sapur.006G185100 Sapur.006G192500 Sapur.006G194300
Sapur.006G195000 Sapur.006G196200 Sapur.006G210500 Sapur.006G215000 Sapur.006G217500 Sapur.007G003100
Sapur.007G012300 Sapur.007G015200 Sapur.007G020600 Sapur.007G026600 Sapur.007G036000 Sapur.007G037800
Sapur.007G037900 Sapur.007G042300 Sapur.007G044000 Sapur.007G054000 Sapur.007G060300 Sapur.007G077400
Sapur.007G085900 Sapur.007G094200 Sapur.007G095100 Sapur.007G099400 Sapur.007G112300 Sapur.007G116000
Sapur.007G121300 Sapur.007G124000 Sapur.007G124100 Sapur.008G008800 Sapur.008G016100 Sapur.008G030600
Sapur.008G045500 Sapur.008G050800 Sapur.008G051200 Sapur.008G051300 Sapur.008G054200 Sapur.008G054500
Sapur.008G058600 Sapur.008G058700 Sapur.008G067000 Sapur.008G076100 Sapur.008G079500 Sapur.008G089200
Sapur.008G093300 Sapur.008G103500 Sapur.008G107400 Sapur.008G111800 Sapur.008G113700 Sapur.008G116700
Sapur.008G116800 Sapur.008G117900 Sapur.008G136200 Sapur.008G136400 Sapur.008G142600 Sapur.008G143200
Sapur.008G165900 Sapur.008G172500 Sapur.009G000500 Sapur.009G023600 Sapur.009G024900 Sapur.009G025400
Sapur.009G025500 Sapur.009G030800 Sapur.009G035100 Sapur.009G052100 Sapur.009G052200 Sapur.009G055600
Sapur.009G058900 Sapur.009G059000 Sapur.009G059300 Sapur.009G066800 Sapur.009G071300 Sapur.009G075100
Sapur.009G078200 Sapur.009G078700 Sapur.009G080300 Sapur.009G084400 Sapur.009G091500 Sapur.009G092000
Sapur.009G106500 Sapur.009G106700 Sapur.009G107300 Sapur.009G122800 Sapur.009G126900 Sapur.010G000800
Sapur.010G008100 Sapur.010G016400 Sapur.010G028600 Sapur.010G041100 Sapur.010G041500 Sapur.010G051000
Sapur.010G052000 Sapur.010G064500 Sapur.010G069300 Sapur.010G070900 Sapur.010G075700 Sapur.010G076000
Sapur.010G076900 Sapur.010G098600 Sapur.010G112700 Sapur.010G112900 Sapur.010G127100 Sapur.010G143300

Sapur.010G143400	Sapur.010G145100	Sapur.010G147600	Sapur.010G147800	Sapur.010G148200	Sapur.010G151400
Sapur.010G169400	Sapur.010G176400	Sapur.010G180200	Sapur.010G189000	Sapur.011G006500	Sapur.011G020400
Sapur.011G021000	Sapur.011G030700	Sapur.011G036900	Sapur.011G040500	Sapur.011G040600	Sapur.011G042800
Sapur.011G046900	Sapur.011G047800	Sapur.011G050200	Sapur.011G050900	Sapur.011G080000	Sapur.011G080600
Sapur.011G083300	Sapur.011G089900	Sapur.011G095700	Sapur.011G096100	Sapur.011G097200	Sapur.011G101300
Sapur.011G108300	Sapur.011G118000	Sapur.011G118200	Sapur.011G118300	Sapur.011G119400	Sapur.011G119600
Sapur.011G121500	Sapur.012G002900	Sapur.012G016400	Sapur.012G018300	Sapur.012G020400	Sapur.012G029700
Sapur.012G032600	Sapur.012G041400	Sapur.012G048500	Sapur.012G048700	Sapur.012G051500	Sapur.012G052000
Sapur.012G054500	Sapur.012G063200	Sapur.012G064400	Sapur.012G064700	Sapur.012G064900	Sapur.012G071500
Sapur.012G086400	Sapur.012G093400	Sapur.012G097700	Sapur.012G101000	Sapur.012G106800	Sapur.012G107200
Sapur.012G109300	Sapur.013G003400	Sapur.013G016100	Sapur.013G034500	Sapur.013G037300	Sapur.013G055800
Sapur.013G068900	Sapur.013G070000	Sapur.013G070400	Sapur.013G098000	Sapur.013G108700	Sapur.013G141900
Sapur.014G010900	Sapur.014G014200	Sapur.014G017800	Sapur.014G030900	Sapur.014G043900	Sapur.014G049700
Sapur.014G050200	Sapur.014G054500	Sapur.014G065600	Sapur.014G065800	Sapur.014G067100	Sapur.014G067400
Sapur.014G069000	Sapur.014G084200	Sapur.014G089900	Sapur.014G091100	Sapur.014G093700	Sapur.014G095000
Sapur.014G095200	Sapur.014G096400	Sapur.014G104400	Sapur.014G110500	Sapur.014G119600	Sapur.014G122000
Sapur.014G126200	Sapur.016G018300	Sapur.016G030100	Sapur.016G032800	Sapur.016G034600	Sapur.016G045300
Sapur.016G046200	Sapur.016G048600	Sapur.016G049500	Sapur.016G064900	Sapur.016G066000	Sapur.016G067200
Sapur.016G089700	Sapur.016G090700	Sapur.016G094000	Sapur.016G094800	Sapur.016G097600	Sapur.016G113500
Sapur.016G115900	Sapur.016G122900	Sapur.016G133200	Sapur.016G133500	Sapur.016G139000	Sapur.016G158100
Sapur.016G158200	Sapur.016G161600	Sapur.016G164800	Sapur.016G165000	Sapur.016G165400	Sapur.016G172500
Sapur.016G177200	Sapur.016G182600	Sapur.016G183900	Sapur.016G193400	Sapur.016G195400	Sapur.016G215200
Sapur.016G222400	Sapur.016G226600	Sapur.016G228400	Sapur.016G256600	Sapur.016G259100	Sapur.016G259200
Sapur.016G259300	Sapur.016G263500	Sapur.016G267500	Sapur.016G267900	Sapur.016G269800	Sapur.016G272100
Sapur.016G272200	Sapur.016G274100	Sapur.016G280000	Sapur.016G293800	Sapur.016G294500	Sapur.016G294800
Sapur.016G300700	Sapur.016G300800	Sapur.016G301000	Sapur.016G304300	Sapur.017G001800	Sapur.017G002000
Sapur.017G019000	Sapur.017G019900	Sapur.017G046000	Sapur.017G050000	Sapur.017G054200	Sapur.017G060600
Sapur.017G067400	Sapur.017G067800	Sapur.017G072700	Sapur.017G096500	Sapur.017G096900	Sapur.017G097600
Sapur.017G107900	Sapur.017G109200	Sapur.017G111400	Sapur.017G114100	Sapur.018G005200	Sapur.018G010500
Sapur.018G010800	Sapur.018G010900	Sapur.018G011100	Sapur.018G011200	Sapur.018G018700	Sapur.018G032300
Sapur.018G035500	Sapur.018G048200	Sapur.018G053200	Sapur.018G053400	Sapur.018G058300	Sapur.018G058400
Sapur.018G060300	Sapur.018G067300	Sapur.018G072700	Sapur.018G081000	Sapur.018G082400	Sapur.018G086200
Sapur.018G104600	Sapur.018G107900	Sapur.018G114200	Sapur.019G000600	Sapur.019G000700	Sapur.019G000900

Sapur.019G002100 Sapur.019G002700 Sapur.019G008900 Sapur.019G016700 Sapur.019G022400 Sapur.019G035200
Sapur.019G053400 Sapur.019G061700 Sapur.019G062400 Sapur.019G083400 Sapur.019G099500 Sapur.019G113000
Sapur.15WG028200 Sapur.15WG031300 Sapur.15WG066000 Sapur.15WG072000 Sapur.15WG078100 Sapur.15WG079300
Sapur.15ZG004600 Sapur.15ZG018000 Sapur.15ZG022100 Sapur.15ZG027900 Sapur.15ZG035300 Sapur.15ZG042700
Sapur.15ZG054200 Sapur.15ZG082200 Sapur.15ZG082400 Sapur.15ZG085600 Sapur.15ZG093000 Sapur.15ZG124400
Sapur.15ZG124800 Sapur.15ZG130400 Sapur.15ZG134100 Sapur.T023100 Sapur.T026500 Sapur.T044700
Sapur.T047800 Sapur.T051500 Sapur.T072500 Sapur.T114400 Sapur.T185900 Sapur.001G006400
Sapur.001G033400 Sapur.001G043500 Sapur.001G050000 Sapur.001G051400 Sapur.001G072900 Sapur.001G079700
Sapur.001G089300 Sapur.001G107900 Sapur.001G112100 Sapur.001G112600 Sapur.001G121300 Sapur.001G136600
Sapur.001G144500 Sapur.001G161400 Sapur.001G167900 Sapur.001G171300 Sapur.001G173200 Sapur.001G185400
Sapur.001G191100 Sapur.001G191900 Sapur.002G001800 Sapur.002G002500 Sapur.002G003700 Sapur.002G004300
Sapur.002G004900 Sapur.002G011200 Sapur.002G017900 Sapur.002G018700 Sapur.002G022300 Sapur.002G028300
Sapur.002G028400 Sapur.002G047800 Sapur.002G054000 Sapur.002G056400 Sapur.002G057800 Sapur.002G059600
Sapur.002G060900 Sapur.002G064200 Sapur.002G065700 Sapur.002G072000 Sapur.002G081200 Sapur.002G081300
Sapur.002G086200 Sapur.002G103600 Sapur.002G108100 Sapur.002G108300 Sapur.002G119700 Sapur.002G125200
Sapur.002G126200 Sapur.002G134700 Sapur.002G135800 Sapur.002G137300 Sapur.002G147500 Sapur.002G149100
Sapur.002G152400 Sapur.002G157000 Sapur.002G160200 Sapur.002G162300 Sapur.002G166700 Sapur.002G168400
Sapur.002G176800 Sapur.002G182500 Sapur.002G184100 Sapur.002G191300 Sapur.002G197100 Sapur.002G200900
Sapur.002G203000 Sapur.002G203700 Sapur.003G008700 Sapur.003G010200 Sapur.003G013400 Sapur.003G022500
Sapur.003G024100 Sapur.003G038600 Sapur.003G040900 Sapur.003G054000 Sapur.003G060700 Sapur.003G063400
Sapur.003G063600 Sapur.003G068300 Sapur.003G079300 Sapur.003G086600 Sapur.003G095000 Sapur.003G099600
Sapur.003G112400 Sapur.003G118000 Sapur.003G123600 Sapur.003G125600 Sapur.003G132900 Sapur.003G165600
Sapur.004G011800 Sapur.004G012100 Sapur.004G027700 Sapur.004G036300 Sapur.004G050300 Sapur.004G052600
Sapur.004G052900 Sapur.004G071000 Sapur.004G076800 Sapur.004G077000 Sapur.004G102500 Sapur.004G103700
Sapur.004G106300 Sapur.004G114100 Sapur.004G114400 Sapur.004G120300 Sapur.004G127000 Sapur.004G129600
Sapur.004G138500 Sapur.004G147800 Sapur.004G152100 Sapur.004G152300 Sapur.004G154700 Sapur.004G155200
Sapur.004G162500 Sapur.004G173300 Sapur.004G177100 Sapur.005G000700 Sapur.005G015300 Sapur.005G030000
Sapur.005G047100 Sapur.005G049400 Sapur.005G052600 Sapur.005G067900 Sapur.005G072700 Sapur.005G073900
Sapur.005G084200 Sapur.005G118500 Sapur.005G125000 Sapur.005G125100 Sapur.005G147300 Sapur.005G147500
Sapur.005G149900 Sapur.005G150200 Sapur.005G150300 Sapur.005G169900 Sapur.005G173800 Sapur.005G174000
Sapur.005G180300 Sapur.005G186400 Sapur.005G189700 Sapur.005G190700 Sapur.005G203300 Sapur.005G204000
Sapur.005G204800 Sapur.005G206700 Sapur.006G001400 Sapur.006G004200 Sapur.006G008700 Sapur.006G015700
Sapur.006G026100 Sapur.006G031900 Sapur.006G036700 Sapur.006G042500 Sapur.006G049200 Sapur.006G050300

Sapur.006G050900	Sapur.006G056800	Sapur.006G060000	Sapur.006G062000	Sapur.006G063300	Sapur.006G067100
Sapur.006G069200	Sapur.006G069300	Sapur.006G085900	Sapur.006G090000	Sapur.006G093600	Sapur.006G101400
Sapur.006G107800	Sapur.006G119400	Sapur.006G123700	Sapur.006G124700	Sapur.006G126300	Sapur.006G126600
Sapur.006G131700	Sapur.006G135500	Sapur.006G157100	Sapur.006G166700	Sapur.006G175000	Sapur.006G177400
Sapur.006G180700	Sapur.006G189300	Sapur.006G192900	Sapur.006G194700	Sapur.006G198700	Sapur.006G204700
Sapur.006G212700	Sapur.006G217000	Sapur.007G003500	Sapur.007G006700	Sapur.007G010500	Sapur.007G018200
Sapur.007G018500	Sapur.007G018700	Sapur.007G029400	Sapur.007G044700	Sapur.007G045200	Sapur.007G051800
Sapur.007G052700	Sapur.007G064600	Sapur.007G074100	Sapur.007G078800	Sapur.007G089900	Sapur.007G090100
Sapur.007G091200	Sapur.007G093300	Sapur.007G096100	Sapur.007G100600	Sapur.007G116800	Sapur.008G003400
Sapur.008G004000	Sapur.008G011900	Sapur.008G013500	Sapur.008G024600	Sapur.008G028500	Sapur.008G031500
Sapur.008G038800	Sapur.008G041000	Sapur.008G043700	Sapur.008G043800	Sapur.008G044000	Sapur.008G046900
Sapur.008G048100	Sapur.008G057700	Sapur.008G061500	Sapur.008G062900	Sapur.008G070800	Sapur.008G072800
Sapur.008G074200	Sapur.008G074800	Sapur.008G078200	Sapur.008G094100	Sapur.008G102200	Sapur.008G103100
Sapur.008G112200	Sapur.008G114600	Sapur.008G119300	Sapur.008G120900	Sapur.008G122100	Sapur.008G154100
Sapur.008G162300	Sapur.008G171500	Sapur.009G001100	Sapur.009G003700	Sapur.009G009100	Sapur.009G014300
Sapur.009G034000	Sapur.009G042700	Sapur.009G044900	Sapur.009G047600	Sapur.009G054400	Sapur.009G065500
Sapur.009G068900	Sapur.009G088500	Sapur.009G096300	Sapur.009G103000	Sapur.009G108500	Sapur.009G113800
Sapur.009G120900	Sapur.009G128100	Sapur.009G129200	Sapur.009G130700	Sapur.010G003300	Sapur.010G013500
Sapur.010G013800	Sapur.010G015200	Sapur.010G019700	Sapur.010G021300	Sapur.010G022800	Sapur.010G029000
Sapur.010G031200	Sapur.010G059500	Sapur.010G071500	Sapur.010G073200	Sapur.010G078700	Sapur.010G082200
Sapur.010G087400	Sapur.010G092800	Sapur.010G099900	Sapur.010G104400	Sapur.010G110600	Sapur.010G110700
Sapur.010G115800	Sapur.010G118000	Sapur.010G118100	Sapur.010G119300	Sapur.010G119600	Sapur.010G122700
Sapur.010G123100	Sapur.010G123700	Sapur.010G124000	Sapur.010G129800	Sapur.010G144300	Sapur.010G144700
Sapur.010G148600	Sapur.010G151600	Sapur.010G156300	Sapur.010G159800	Sapur.010G160100	Sapur.010G163100
Sapur.010G167500	Sapur.010G170500	Sapur.010G170600	Sapur.010G178800	Sapur.010G199900	Sapur.010G202000
Sapur.010G203100	Sapur.010G205600	Sapur.011G002500	Sapur.011G008300	Sapur.011G009700	Sapur.011G029700
Sapur.011G032700	Sapur.011G032800	Sapur.011G033200	Sapur.011G044000	Sapur.011G072100	Sapur.011G080400
Sapur.011G098300	Sapur.011G109500	Sapur.011G112000	Sapur.011G112600	Sapur.011G122000	Sapur.012G030800
Sapur.012G033500	Sapur.012G034900	Sapur.012G045600	Sapur.012G050000	Sapur.012G052600	Sapur.012G053100
Sapur.012G060600	Sapur.012G066100	Sapur.012G066200	Sapur.012G067800	Sapur.012G080500	Sapur.012G083700
Sapur.012G109200	Sapur.013G005200	Sapur.013G010500	Sapur.013G013500	Sapur.013G024200	Sapur.013G026100
Sapur.013G045800	Sapur.013G050400	Sapur.013G055400	Sapur.013G068300	Sapur.013G091800	Sapur.013G096200
Sapur.013G106000	Sapur.013G113400	Sapur.013G117800	Sapur.013G119000	Sapur.013G129800	Sapur.013G132500

Sapur.013G142400 Sapur.014G013900 Sapur.014G018800 Sapur.014G030400 Sapur.014G034800 Sapur.014G039700
Sapur.014G049800 Sapur.014G055700 Sapur.014G057800 Sapur.014G059500 Sapur.014G059800 Sapur.014G062000
Sapur.014G072000 Sapur.014G077500 Sapur.014G087900 Sapur.014G091500 Sapur.014G091800 Sapur.014G097700
Sapur.014G100100 Sapur.014G103300 Sapur.014G116400 Sapur.014G116700 Sapur.014G118100 Sapur.014G118900
Sapur.014G125100 Sapur.014G136300 Sapur.016G000600 Sapur.016G001300 Sapur.016G010900 Sapur.016G018000
Sapur.016G030700 Sapur.016G039800 Sapur.016G041700 Sapur.016G045200 Sapur.016G047300 Sapur.016G054400
Sapur.016G054600 Sapur.016G074400 Sapur.016G082300 Sapur.016G113700 Sapur.016G130500 Sapur.016G132100
Sapur.016G133000 Sapur.016G140600 Sapur.016G148200 Sapur.016G149600 Sapur.016G160500 Sapur.016G180500
Sapur.016G181700 Sapur.016G195500 Sapur.016G206000 Sapur.016G206300 Sapur.016G212300 Sapur.016G215600
Sapur.016G221000 Sapur.016G222900 Sapur.016G240600 Sapur.016G244800 Sapur.016G246100 Sapur.016G248100
Sapur.016G252400 Sapur.016G253800 Sapur.016G265100 Sapur.016G266500 Sapur.016G275400 Sapur.016G280900
Sapur.016G287300 Sapur.016G289400 Sapur.016G290200 Sapur.017G000100 Sapur.017G032700 Sapur.017G044200
Sapur.017G046300 Sapur.017G049800 Sapur.017G050100 Sapur.017G062700 Sapur.017G065900 Sapur.017G072900
Sapur.017G073000 Sapur.017G074700 Sapur.017G075600 Sapur.017G083200 Sapur.017G086800 Sapur.017G100800
Sapur.017G111600 Sapur.017G112500 Sapur.017G124600 Sapur.018G013300 Sapur.018G021100 Sapur.018G051900
Sapur.018G053800 Sapur.018G060800 Sapur.018G061200 Sapur.018G064500 Sapur.018G066500 Sapur.018G075900
Sapur.018G078800 Sapur.018G083400 Sapur.018G094800 Sapur.018G096200 Sapur.018G101200 Sapur.018G103200
Sapur.018G108000 Sapur.018G110200 Sapur.019G033100 Sapur.019G037200 Sapur.019G052800 Sapur.019G060800
Sapur.019G098500 Sapur.019G099900 Sapur.019G100200 Sapur.019G109000 Sapur.15WG027600 Sapur.15WG064400
Sapur.15WG071900 Sapur.15ZG000700 Sapur.15ZG018200 Sapur.15ZG024700 Sapur.15ZG027200 Sapur.15ZG033400
Sapur.15ZG038800 Sapur.15ZG052500 Sapur.15ZG060800 Sapur.15ZG072800 Sapur.15ZG080600 Sapur.15ZG081500
Sapur.15ZG095400 Sapur.15ZG101800 Sapur.15ZG102400 Sapur.15ZG102900 Sapur.15ZG103100 Sapur.15ZG118900
Sapur.15ZG133000 Sapur.T004100 Sapur.T004200 Sapur.T045900 Sapur.T051700 Sapur.T052100 Sapur.T058900
Sapur.T080300 Sapur.T091200 Sapur.T153300 Sapur.001G026000 Sapur.001G032700 Sapur.001G081000
Sapur.001G091600 Sapur.001G099000 Sapur.001G105100 Sapur.001G115400 Sapur.002G007000 Sapur.002G040700
Sapur.002G046100 Sapur.002G061200 Sapur.002G146100 Sapur.002G170400 Sapur.003G032100 Sapur.003G043700
Sapur.003G048600 Sapur.003G049600 Sapur.003G066700 Sapur.003G139500 Sapur.004G006900 Sapur.004G036700
Sapur.004G052000 Sapur.004G082800 Sapur.004G095900 Sapur.004G116800 Sapur.004G176800 Sapur.005G138900
Sapur.005G184400 Sapur.006G071500 Sapur.006G071600 Sapur.006G093200 Sapur.006G124900 Sapur.006G145900
Sapur.006G206200 Sapur.008G040600 Sapur.008G066400 Sapur.008G083400 Sapur.008G102400 Sapur.009G019400
Sapur.009G033500 Sapur.009G033700 Sapur.009G053300 Sapur.009G079300 Sapur.010G052900 Sapur.010G057200
Sapur.010G088200 Sapur.011G107900 Sapur.011G121100 Sapur.012G058000 Sapur.012G079500 Sapur.013G013900
Sapur.013G047900 Sapur.014G040300 Sapur.014G056500 Sapur.014G074600 Sapur.014G105300 Sapur.016G020000

				Sapur.016G020300 Sapur.016G095700 Sapur.016G127900 Sapur.016G288100 Sapur.016G297800 Sapur.017G040100 Sapur.017G078500 Sapur.018G007400 Sapur.018G026500 Sapur.018G103900 Sapur.019G024100 Sapur.019G079900 Sapur.15WG069800 Sapur.15ZG020900 Sapur.15ZG039900 Sapur.15ZG089500 Sapur.15ZG091400 Sapur.15ZG117500 Sapur.T031900 Sapur.T167500 Sapur.001G053400 Sapur.001G089000 Sapur.001G093200 Sapur.002G025200 Sapur.002G032600 Sapur.002G048700 Sapur.002G099200 Sapur.002G099300 Sapur.002G108700 Sapur.002G143800 Sapur.002G180700 Sapur.003G119600 Sapur.005G034700 Sapur.005G093900 Sapur.005G123600 Sapur.006G023400 Sapur.006G063500 Sapur.006G115900 Sapur.006G140400 Sapur.006G151000 Sapur.006G195400 Sapur.007G001800 Sapur.007G013700 Sapur.007G023900 Sapur.007G047100 Sapur.007G047200 Sapur.007G067000 Sapur.007G075400 Sapur.008G007500 Sapur.008G043100 Sapur.008G053200 Sapur.008G068400 Sapur.008G090000 Sapur.009G050100 Sapur.009G050200 Sapur.009G050400 Sapur.009G099500 Sapur.010G021600 Sapur.010G082500 Sapur.010G107500 Sapur.010G160600 Sapur.011G066900 Sapur.011G067400 Sapur.013G141300 Sapur.013G143000 Sapur.014G143600 Sapur.016G023600 Sapur.016G028000 Sapur.016G096000 Sapur.016G134900 Sapur.016G156100 Sapur.016G162100 Sapur.016G170800 Sapur.016G204800 Sapur.016G214600 Sapur.018G113700 Sapur.019G112500 Sapur.004G005600 Sapur.006G054000 Sapur.008G149700 Sapur.010G023600 Sapur.011G005900 Sapur.003G077800 Sapur.018G062800 Sapur.15ZG021300
GO:0008233	133	445	peptidase activity	Sapur.001G001400 Sapur.001G031800 Sapur.001G088500 Sapur.001G098400 Sapur.001G135000 Sapur.001G139800 Sapur.001G146600 Sapur.002G009400 Sapur.002G063400 Sapur.002G072700 Sapur.002G084000 Sapur.002G097300 Sapur.002G101500 Sapur.002G108400 Sapur.002G124000 Sapur.002G130600 Sapur.002G143900 Sapur.002G150900 Sapur.002G179600 Sapur.003G034800 Sapur.003G037900 Sapur.003G045000 Sapur.003G087600 Sapur.003G087900 Sapur.003G088100 Sapur.003G092600 Sapur.003G151400 Sapur.004G005000 Sapur.004G060000 Sapur.004G130500 Sapur.004G164900 Sapur.004G165000 Sapur.004G173100 Sapur.005G001600 Sapur.005G028800 Sapur.005G047600 Sapur.005G053000 Sapur.005G062300 Sapur.005G086400 Sapur.005G086600 Sapur.005G122400 Sapur.005G123400 Sapur.005G194900 Sapur.006G061500 Sapur.006G070700 Sapur.006G115800 Sapur.006G161800 Sapur.006G169900 Sapur.006G192500 Sapur.006G194300 Sapur.007G042300 Sapur.007G060300 Sapur.007G095100 Sapur.007G116000 Sapur.008G030600 Sapur.008G045500 Sapur.008G051200 Sapur.008G051300 Sapur.008G093300 Sapur.008G142600 Sapur.009G000500 Sapur.009G023600 Sapur.009G024900 Sapur.009G055600 Sapur.009G106500 Sapur.009G126900 Sapur.010G051000 Sapur.010G098600 Sapur.010G176400 Sapur.011G006500 Sapur.012G018300 Sapur.012G086400 Sapur.012G101000 Sapur.012G107200 Sapur.012G109300 Sapur.013G070400 Sapur.014G010900 Sapur.014G030900 Sapur.014G043900 Sapur.014G065800 Sapur.014G096400 Sapur.014G122000 Sapur.016G066000 Sapur.016G133500 Sapur.016G161600 Sapur.016G183900 Sapur.016G228400 Sapur.016G294800 Sapur.017G109200 Sapur.018G005200 Sapur.018G010500 Sapur.018G010800 Sapur.018G010900 Sapur.018G011100 Sapur.018G011200 Sapur.018G058400 Sapur.019G016700 Sapur.019G022400 Sapur.019G053400 Sapur.15WG072000 Sapur.15ZG042700 Sapur.15ZG130400 Sapur.T026500 Sapur.001G051400 Sapur.002G003700 Sapur.002G022300 Sapur.002G056400

				Sapur.002G134700 Sapur.004G120300 Sapur.004G162500 Sapur.005G072700 Sapur.005G125000 Sapur.005G147300 Sapur.005G204800 Sapur.006G026100 Sapur.006G063300 Sapur.008G024600 Sapur.008G031500 Sapur.009G001100 Sapur.009G042700 Sapur.009G096300 Sapur.010G115800 Sapur.012G080500 Sapur.014G013900 Sapur.014G039700 Sapur.016G030700 Sapur.016G181700 Sapur.018G078800 Sapur.019G052800 Sapur.15ZG102400 Sapur.T045900 Sapur.016G095700 Sapur.016G248100
GO:0016787	387	1881	hydrolase activity	Sapur.001G001400 Sapur.001G031800 Sapur.001G086500 Sapur.001G088500 Sapur.001G098400 Sapur.001G135000 Sapur.001G139800 Sapur.001G146600 Sapur.001G175000 Sapur.001G185700 Sapur.002G009400 Sapur.002G015900 Sapur.002G028100 Sapur.002G034900 Sapur.002G063400 Sapur.002G072700 Sapur.002G084000 Sapur.002G090500 Sapur.002G097300 Sapur.002G101500 Sapur.002G108400 Sapur.002G124000 Sapur.002G130600 Sapur.002G143900 Sapur.002G146800 Sapur.002G150900 Sapur.002G179600 Sapur.003G000800 Sapur.003G034800 Sapur.003G037900 Sapur.003G045000 Sapur.003G061600 Sapur.003G087600 Sapur.003G087900 Sapur.003G088100 Sapur.003G090100 Sapur.003G091100 Sapur.003G092600 Sapur.003G151400 Sapur.003G166900 Sapur.004G005000 Sapur.004G021600 Sapur.004G060000 Sapur.004G130500 Sapur.004G164900 Sapur.004G165000 Sapur.004G173100 Sapur.005G001600 Sapur.005G028800 Sapur.005G047400 Sapur.005G047600 Sapur.005G053000 Sapur.005G062300 Sapur.005G086400 Sapur.005G086600 Sapur.005G122400 Sapur.005G123400 Sapur.005G180900 Sapur.005G192400 Sapur.005G194900 Sapur.006G025400 Sapur.006G037500 Sapur.006G061500 Sapur.006G070700 Sapur.006G102100 Sapur.006G115800 Sapur.006G150200 Sapur.006G161800 Sapur.006G169900 Sapur.006G180500 Sapur.006G192500 Sapur.006G194300 Sapur.007G042300 Sapur.007G060300 Sapur.007G095100 Sapur.007G116000 Sapur.008G030600 Sapur.008G045500 Sapur.008G051200 Sapur.008G051300 Sapur.008G093300 Sapur.008G142600 Sapur.009G000500 Sapur.009G023600 Sapur.009G024900 Sapur.009G052100 Sapur.009G052200 Sapur.009G055600 Sapur.009G066800 Sapur.009G106500 Sapur.009G126900 Sapur.010G051000 Sapur.010G069300 Sapur.010G098600 Sapur.010G176400 Sapur.010G180200 Sapur.011G006500 Sapur.011G119600 Sapur.012G018300 Sapur.012G086400 Sapur.012G101000 Sapur.012G107200 Sapur.012G109300 Sapur.013G016100 Sapur.013G070400 Sapur.014G010900 Sapur.014G030900 Sapur.014G043900 Sapur.014G065800 Sapur.014G089900 Sapur.014G096400 Sapur.014G104400 Sapur.014G122000 Sapur.016G030100 Sapur.016G066000 Sapur.016G094800 Sapur.016G133500 Sapur.016G158100 Sapur.016G158200 Sapur.016G161600 Sapur.016G172500 Sapur.016G183900 Sapur.016G193400 Sapur.016G226600 Sapur.016G228400 Sapur.016G294800 Sapur.017G046000 Sapur.017G067400 Sapur.017G109200 Sapur.018G005200 Sapur.018G010500 Sapur.018G010800 Sapur.018G010900 Sapur.018G011100 Sapur.018G011200 Sapur.018G053400 Sapur.018G058400 Sapur.018G081000 Sapur.019G002100 Sapur.019G002700 Sapur.019G016700 Sapur.019G022400 Sapur.019G035200 Sapur.019G053400 Sapur.15WG072000 Sapur.15WG078100 Sapur.15ZG004600 Sapur.15ZG042700 Sapur.15ZG130400 Sapur.T026500 Sapur.T047800 Sapur.T114400 Sapur.001G006400 Sapur.001G051400 Sapur.001G072900 Sapur.001G112100 Sapur.001G136600 Sapur.001G167900 Sapur.001G171300 Sapur.001G191100 Sapur.001G191900 Sapur.002G003700 Sapur.002G004900 Sapur.002G017900 Sapur.002G022300 Sapur.002G056400 Sapur.002G064200

Sapur.002G125200	Sapur.002G134700	Sapur.002G135800	Sapur.002G137300	Sapur.002G162300	Sapur.002G166700
Sapur.002G182500	Sapur.002G184100	Sapur.002G197100	Sapur.002G200900	Sapur.002G203700	Sapur.003G013400
Sapur.003G022500	Sapur.003G024100	Sapur.003G040900	Sapur.003G063600	Sapur.003G112400	Sapur.003G118000
Sapur.003G132900	Sapur.003G165600	Sapur.004G011800	Sapur.004G012100	Sapur.004G027700	Sapur.004G036300
Sapur.004G102500	Sapur.004G120300	Sapur.004G129600	Sapur.004G147800	Sapur.004G152100	Sapur.004G162500
Sapur.004G173300	Sapur.005G000700	Sapur.005G015300	Sapur.005G030000	Sapur.005G052600	Sapur.005G072700
Sapur.005G084200	Sapur.005G118500	Sapur.005G125000	Sapur.005G147300	Sapur.005G169900	Sapur.005G186400
Sapur.005G190700	Sapur.005G203300	Sapur.005G203500	Sapur.005G204800	Sapur.006G001400	Sapur.006G004200
Sapur.006G026100	Sapur.006G049200	Sapur.006G050300	Sapur.006G050900	Sapur.006G056800	Sapur.006G060000
Sapur.006G063300	Sapur.006G067100	Sapur.006G069200	Sapur.006G069300	Sapur.006G085900	Sapur.006G090000
Sapur.006G119400	Sapur.006G123700	Sapur.006G124700	Sapur.006G126600	Sapur.006G131700	Sapur.006G157100
Sapur.006G166700	Sapur.006G177400	Sapur.006G180700	Sapur.007G045200	Sapur.007G051800	Sapur.007G052700
Sapur.007G054000	Sapur.007G064600	Sapur.007G089900	Sapur.007G090100	Sapur.007G116800	Sapur.008G004000
Sapur.008G024600	Sapur.008G031500	Sapur.008G038800	Sapur.008G043700	Sapur.008G043800	Sapur.008G057700
Sapur.008G061500	Sapur.008G062900	Sapur.008G102200	Sapur.008G112200	Sapur.008G120900	Sapur.008G154100
Sapur.009G001100	Sapur.009G003700	Sapur.009G009100	Sapur.009G034000	Sapur.009G042700	Sapur.009G054400
Sapur.009G065500	Sapur.009G068900	Sapur.009G096300	Sapur.009G120900	Sapur.009G128100	Sapur.010G003300
Sapur.010G021300	Sapur.010G022800	Sapur.010G071500	Sapur.010G082200	Sapur.010G110600	Sapur.010G110700
Sapur.010G115800	Sapur.010G118000	Sapur.010G123700	Sapur.010G124000	Sapur.010G144300	Sapur.010G148600
Sapur.010G151600	Sapur.010G160100	Sapur.010G167500	Sapur.010G170500	Sapur.010G170600	Sapur.010G203100
Sapur.010G205600	Sapur.011G002500	Sapur.011G029700	Sapur.011G044000	Sapur.011G072100	Sapur.011G080400
Sapur.011G112600	Sapur.012G033500	Sapur.012G053100	Sapur.012G060600	Sapur.012G066100	Sapur.012G066200
Sapur.012G080500	Sapur.012G109200	Sapur.013G005200	Sapur.013G010500	Sapur.013G013500	Sapur.013G026100
Sapur.013G050400	Sapur.013G055400	Sapur.013G091800	Sapur.013G113400	Sapur.013G117800	Sapur.013G119000
Sapur.014G013900	Sapur.014G034800	Sapur.014G039700	Sapur.014G057800	Sapur.014G072000	Sapur.014G087900
Sapur.014G091800	Sapur.014G097700	Sapur.014G116700	Sapur.014G118900	Sapur.016G000600	Sapur.016G030700
Sapur.016G039800	Sapur.016G054400	Sapur.016G054600	Sapur.016G132100	Sapur.016G140600	Sapur.016G160500
Sapur.016G181700	Sapur.016G195500	Sapur.016G212300	Sapur.016G222900	Sapur.016G248100	Sapur.016G265100
Sapur.016G280900	Sapur.016G287300	Sapur.016G289400	Sapur.017G032700	Sapur.017G044200	Sapur.017G046300
Sapur.017G050100	Sapur.017G065900	Sapur.017G112500	Sapur.018G013300	Sapur.018G021100	Sapur.018G053800
Sapur.018G061200	Sapur.018G078800	Sapur.018G086200	Sapur.018G094800	Sapur.018G096200	Sapur.018G101200
Sapur.018G103200	Sapur.018G108000	Sapur.019G033100	Sapur.019G037200	Sapur.019G052800	Sapur.019G109000
Sapur.15WG064400	Sapur.15WG071900	Sapur.15ZG000700	Sapur.15ZG024700	Sapur.15ZG052500	Sapur.15ZG072800

				Sapur.15ZG081500 Sapur.15ZG095400 Sapur.15ZG102400 Sapur.15ZG102900 Sapur.15ZG103100 Sapur.15ZG118900 Sapur.15ZG133000 Sapur.T004200 Sapur.T045900 Sapur.T051700 Sapur.T080300 Sapur.T091200 Sapur.003G048600 Sapur.016G095700 Sapur.002G108700 Sapur.006G151000 Sapur.008G068400 Sapur.008G090000 Sapur.010G107500 Sapur.014G143600 Sapur.016G023600 Sapur.003G077800
GO:0004190	46	93	aspartic-type endopeptidase activity	Sapur.001G031800 Sapur.002G084000 Sapur.002G130600 Sapur.002G143900 Sapur.002G179600 Sapur.003G045000 Sapur.003G092600 Sapur.003G151400 Sapur.004G005000 Sapur.004G060000 Sapur.004G165000 Sapur.005G001600 Sapur.005G047600 Sapur.005G053000 Sapur.005G062300 Sapur.005G086400 Sapur.005G086600 Sapur.005G123400 Sapur.007G060300 Sapur.007G095100 Sapur.008G045500 Sapur.008G093300 Sapur.009G000500 Sapur.009G055600 Sapur.009G126900 Sapur.010G098600 Sapur.011G006500 Sapur.012G109300 Sapur.013G070400 Sapur.014G010900 Sapur.014G065800 Sapur.014G122000 Sapur.016G066000 Sapur.016G161600 Sapur.016G183900 Sapur.016G228400 Sapur.017G109200 Sapur.018G010500 Sapur.018G010800 Sapur.018G010900 Sapur.018G011100 Sapur.018G011200 Sapur.019G016700 Sapur.019G053400 Sapur.15WG072000 Sapur.15ZG042700
GO:0070001	46	93	aspartic-type peptidase activity	Sapur.001G031800 Sapur.002G084000 Sapur.002G130600 Sapur.002G143900 Sapur.002G179600 Sapur.003G045000 Sapur.003G092600 Sapur.003G151400 Sapur.004G005000 Sapur.004G060000 Sapur.004G165000 Sapur.005G001600 Sapur.005G047600 Sapur.005G053000 Sapur.005G062300 Sapur.005G086400 Sapur.005G086600 Sapur.005G123400 Sapur.007G060300 Sapur.007G095100 Sapur.008G045500 Sapur.008G093300 Sapur.009G000500 Sapur.009G055600 Sapur.009G126900 Sapur.010G098600 Sapur.011G006500 Sapur.012G109300 Sapur.013G070400 Sapur.014G010900 Sapur.014G065800 Sapur.014G122000 Sapur.016G066000 Sapur.016G161600 Sapur.016G183900 Sapur.016G228400 Sapur.017G109200 Sapur.018G010500 Sapur.018G010800 Sapur.018G010900 Sapur.018G011100 Sapur.018G011200 Sapur.019G016700 Sapur.019G053400 Sapur.15WG072000 Sapur.15ZG042700
GO:0004175	90	290	endopeptidase activity	Sapur.001G001400 Sapur.001G031800 Sapur.001G088500 Sapur.001G135000 Sapur.001G139800 Sapur.002G009400 Sapur.002G072700 Sapur.002G084000 Sapur.002G097300 Sapur.002G101500 Sapur.002G108400 Sapur.002G124000 Sapur.002G130600 Sapur.002G143900 Sapur.002G150900 Sapur.002G179600 Sapur.003G037900 Sapur.003G045000 Sapur.003G087600 Sapur.003G087900 Sapur.003G088100 Sapur.003G092600 Sapur.003G151400 Sapur.004G005000 Sapur.004G060000 Sapur.004G130500 Sapur.004G165000 Sapur.004G173100 Sapur.005G001600 Sapur.005G047600 Sapur.005G053000 Sapur.005G062300 Sapur.005G086400 Sapur.005G086600 Sapur.005G122400 Sapur.005G123400 Sapur.005G194900 Sapur.006G061500 Sapur.006G070700 Sapur.006G115800 Sapur.006G161800 Sapur.007G042300 Sapur.007G060300 Sapur.007G095100 Sapur.007G116000 Sapur.008G030600 Sapur.008G045500 Sapur.008G093300 Sapur.009G000500 Sapur.009G023600 Sapur.009G024900 Sapur.009G055600 Sapur.009G106500 Sapur.009G126900 Sapur.010G098600 Sapur.010G176400 Sapur.011G006500 Sapur.012G018300 Sapur.012G086400 Sapur.012G101000 Sapur.012G107200 Sapur.012G109300 Sapur.013G070400 Sapur.014G010900 Sapur.014G030900 Sapur.014G043900 Sapur.014G065800 Sapur.014G122000 Sapur.016G066000 Sapur.016G133500 Sapur.016G161600 Sapur.016G183900 Sapur.016G228400 Sapur.016G294800 Sapur.017G109200 Sapur.018G010500 Sapur.018G010800

				Sapur.018G010900 Sapur.018G011100 Sapur.018G011200 Sapur.018G058400 Sapur.019G016700 Sapur.019G053400 Sapur.15WG072000 Sapur.15ZG042700 Sapur.15ZG130400 Sapur.T026500 Sapur.006G063300 Sapur.014G039700 Sapur.019G022400
GO:0015078	35	67	proton transmembrane transporter activity	Sapur.001G144500 Sapur.002G054000 Sapur.003G054000 Sapur.003G120000 Sapur.005G150200 Sapur.005G150300 Sapur.005G172700 Sapur.007G006700 Sapur.009G047600 Sapur.010G202000 Sapur.013G047900 Sapur.013G064900 Sapur.013G142400 Sapur.018G075900 Sapur.018G083400 Sapur.T031900 Sapur.002G020100 Sapur.002G021300 Sapur.002G066000 Sapur.005G186900 Sapur.005G188200 Sapur.006G205800 Sapur.007G012200 Sapur.009G099300 Sapur.010G173300 Sapur.016G197500 Sapur.017G051500 Sapur.017G065500 Sapur.018G041100 Sapur.T003500 Sapur.T012400 Sapur.003G100300 Sapur.005G058800 Sapur.016G098500 Sapur.017G027100
GO:0008194	31	55	UDP-glycosyltransferase activity	Sapur.001G043500 Sapur.001G185400 Sapur.002G086200 Sapur.003G010200 Sapur.004G103700 Sapur.004G155200 Sapur.005G125100 Sapur.006G062000 Sapur.006G107800 Sapur.006G192900 Sapur.006G198700 Sapur.006G217000 Sapur.007G044700 Sapur.008G003400 Sapur.008G011900 Sapur.008G103100 Sapur.010G087400 Sapur.010G092800 Sapur.011G008300 Sapur.013G068300 Sapur.014G018800 Sapur.014G136300 Sapur.016G082300 Sapur.016G113700 Sapur.016G221000 Sapur.017G062700 Sapur.018G110200 Sapur.019G099900 Sapur.006G054000 Sapur.008G149700 Sapur.010G023600
GO:0016757	116	445	glycosyltransferase activity	Sapur.001G043500 Sapur.001G112600 Sapur.001G185400 Sapur.002G004300 Sapur.002G072000 Sapur.002G086200 Sapur.002G108100 Sapur.002G160200 Sapur.002G176800 Sapur.002G191300 Sapur.003G010200 Sapur.003G063400 Sapur.003G095000 Sapur.003G099600 Sapur.004G076800 Sapur.004G103700 Sapur.004G106300 Sapur.004G138500 Sapur.004G154700 Sapur.004G155200 Sapur.004G177100 Sapur.005G047100 Sapur.005G125100 Sapur.005G147500 Sapur.005G174000 Sapur.005G204000 Sapur.006G008700 Sapur.006G036700 Sapur.006G062000 Sapur.006G093600 Sapur.006G107800 Sapur.006G192900 Sapur.006G198700 Sapur.006G212700 Sapur.006G217000 Sapur.007G029400 Sapur.007G044700 Sapur.007G096100 Sapur.007G100600 Sapur.008G003400 Sapur.008G011900 Sapur.008G028500 Sapur.008G070800 Sapur.008G072800 Sapur.008G094100 Sapur.008G103100 Sapur.008G162300 Sapur.009G130700 Sapur.010G015200 Sapur.010G031200 Sapur.010G087400 Sapur.010G092800 Sapur.010G099900 Sapur.010G118100 Sapur.010G129800 Sapur.010G178800 Sapur.011G008300 Sapur.011G098300 Sapur.011G112000 Sapur.012G034900 Sapur.012G045600 Sapur.012G052600 Sapur.013G045800 Sapur.013G068300 Sapur.013G129800 Sapur.014G018800 Sapur.014G030400 Sapur.014G055700 Sapur.014G100100 Sapur.014G118100 Sapur.014G125100 Sapur.014G136300 Sapur.016G001300 Sapur.016G010900 Sapur.016G082300 Sapur.016G113700 Sapur.016G221000 Sapur.016G252400 Sapur.016G253800 Sapur.016G275400 Sapur.016G290200 Sapur.017G062700 Sapur.017G074700 Sapur.017G086800 Sapur.018G064500 Sapur.018G110200 Sapur.019G099900 Sapur.019G100200 Sapur.15ZG018200 Sapur.15ZG038800 Sapur.15ZG060800 Sapur.15ZG080600 Sapur.15ZG101800 Sapur.T004100 Sapur.T058900 Sapur.T153300 Sapur.001G026000 Sapur.002G146100 Sapur.004G095900 Sapur.006G145900 Sapur.009G033500 Sapur.009G079300 Sapur.014G040300 Sapur.014G074600 Sapur.014G105300 Sapur.016G020000 Sapur.016G020300

				Sapur.017G040100 Sapur.018G007400 Sapur.019G079900 Sapur.15ZG020900 Sapur.006G054000 Sapur.008G149700 Sapur.010G023600 Sapur.018G062800 Sapur.15ZG021300
GO:0140096	445	2472	catalytic activity, acting on a protein	Sapur.001G001400 Sapur.001G015800 Sapur.001G031800 Sapur.001G033500 Sapur.001G042400 Sapur.001G043100 Sapur.001G066800 Sapur.001G078000 Sapur.001G086800 Sapur.001G088500 Sapur.001G093700 Sapur.001G098400 Sapur.001G103900 Sapur.001G124800 Sapur.001G135000 Sapur.001G137500 Sapur.001G139800 Sapur.001G146600 Sapur.001G148700 Sapur.001G171200 Sapur.001G175900 Sapur.001G190300 Sapur.001G191000 Sapur.002G009400 Sapur.002G010500 Sapur.002G028000 Sapur.002G046900 Sapur.002G055700 Sapur.002G063400 Sapur.002G072700 Sapur.002G084000 Sapur.002G097300 Sapur.002G101500 Sapur.002G106900 Sapur.002G108400 Sapur.002G120400 Sapur.002G124000 Sapur.002G130600 Sapur.002G143900 Sapur.002G150900 Sapur.002G164000 Sapur.002G164400 Sapur.002G176000 Sapur.002G179600 Sapur.002G180800 Sapur.002G188100 Sapur.002G192000 Sapur.002G197600 Sapur.002G198100 Sapur.002G199800 Sapur.002G201200 Sapur.002G201300 Sapur.003G013500 Sapur.003G034800 Sapur.003G036900 Sapur.003G037300 Sapur.003G037900 Sapur.003G042700 Sapur.003G045000 Sapur.003G051100 Sapur.003G052500 Sapur.003G061800 Sapur.003G067600 Sapur.003G073200 Sapur.003G075500 Sapur.003G082200 Sapur.003G085300 Sapur.003G087600 Sapur.003G087900 Sapur.003G088100 Sapur.003G089700 Sapur.003G092600 Sapur.003G095700 Sapur.003G099700 Sapur.003G131900 Sapur.003G133000 Sapur.003G138700 Sapur.003G151400 Sapur.003G153700 Sapur.004G005000 Sapur.004G009000 Sapur.004G017100 Sapur.004G023900 Sapur.004G029200 Sapur.004G032900 Sapur.004G033000 Sapur.004G044400 Sapur.004G046500 Sapur.004G057300 Sapur.004G060000 Sapur.004G061000 Sapur.004G067800 Sapur.004G068600 Sapur.004G080700 Sapur.004G087900 Sapur.004G115800 Sapur.004G129800 Sapur.004G130500 Sapur.004G135100 Sapur.004G164900 Sapur.004G165000 Sapur.004G168300 Sapur.004G170800 Sapur.004G172200 Sapur.004G173100 Sapur.004G177200 Sapur.005G001600 Sapur.005G001800 Sapur.005G011700 Sapur.005G028800 Sapur.005G031100 Sapur.005G039800 Sapur.005G047600 Sapur.005G050800 Sapur.005G053000 Sapur.005G057200 Sapur.005G062300 Sapur.005G065000 Sapur.005G065300 Sapur.005G081600 Sapur.005G086400 Sapur.005G086600 Sapur.005G109200 Sapur.005G122400 Sapur.005G123400 Sapur.005G143900 Sapur.005G148400 Sapur.005G160000 Sapur.005G185800 Sapur.005G194900 Sapur.005G202000 Sapur.005G203500 Sapur.005G205600 Sapur.005G208100 Sapur.006G030600 Sapur.006G039400 Sapur.006G043700 Sapur.006G044000 Sapur.006G059800 Sapur.006G061500 Sapur.006G063800 Sapur.006G064600 Sapur.006G070700 Sapur.006G077000 Sapur.006G088300 Sapur.006G090100 Sapur.006G093700 Sapur.006G096300 Sapur.006G107000 Sapur.006G115800 Sapur.006G117000 Sapur.006G124800 Sapur.006G151400 Sapur.006G161800 Sapur.006G164600 Sapur.006G169900 Sapur.006G185100 Sapur.006G192500 Sapur.006G194300 Sapur.006G195000 Sapur.006G196200 Sapur.006G210500 Sapur.006G215000 Sapur.006G217500 Sapur.007G003100 Sapur.007G012300 Sapur.007G015200 Sapur.007G037800 Sapur.007G042300 Sapur.007G044000 Sapur.007G054000 Sapur.007G060300 Sapur.007G077400 Sapur.007G085900 Sapur.007G094200 Sapur.007G095100 Sapur.007G099400 Sapur.007G116000 Sapur.007G121300 Sapur.007G124000 Sapur.007G124100 Sapur.008G008800 Sapur.008G016100 Sapur.008G030600 Sapur.008G045500

Sapur.008G051200	Sapur.008G051300	Sapur.008G054200	Sapur.008G054500	Sapur.008G076100	Sapur.008G089200
Sapur.008G093300	Sapur.008G103500	Sapur.008G107400	Sapur.008G111800	Sapur.008G113700	Sapur.008G116700
Sapur.008G116800	Sapur.008G136200	Sapur.008G136400	Sapur.008G142600	Sapur.008G143200	Sapur.008G165900
Sapur.009G000500	Sapur.009G023600	Sapur.009G024900	Sapur.009G030800	Sapur.009G035100	Sapur.009G055600
Sapur.009G058900	Sapur.009G059000	Sapur.009G059300	Sapur.009G078700	Sapur.009G091500	Sapur.009G092000
Sapur.009G106500	Sapur.009G107300	Sapur.009G126900	Sapur.010G000800	Sapur.010G008100	Sapur.010G028600
Sapur.010G041500	Sapur.010G051000	Sapur.010G052000	Sapur.010G064500	Sapur.010G070900	Sapur.010G075700
Sapur.010G076000	Sapur.010G076900	Sapur.010G098600	Sapur.010G112900	Sapur.010G127100	Sapur.010G145100
Sapur.010G147600	Sapur.010G147800	Sapur.010G148200	Sapur.010G169400	Sapur.010G176400	Sapur.010G189000
Sapur.011G006500	Sapur.011G021000	Sapur.011G030700	Sapur.011G036900	Sapur.011G040500	Sapur.011G040600
Sapur.011G046900	Sapur.011G047800	Sapur.011G050200	Sapur.011G050900	Sapur.011G080000	Sapur.011G080600
Sapur.011G089900	Sapur.011G095700	Sapur.011G097200	Sapur.011G101300	Sapur.011G108300	Sapur.011G121500
Sapur.012G018300	Sapur.012G020400	Sapur.012G029700	Sapur.012G032600	Sapur.012G041400	Sapur.012G048500
Sapur.012G051500	Sapur.012G052000	Sapur.012G063200	Sapur.012G064400	Sapur.012G064900	Sapur.012G086400
Sapur.012G093400	Sapur.012G097700	Sapur.012G101000	Sapur.012G106800	Sapur.012G107200	Sapur.012G109300
Sapur.013G003400	Sapur.013G034500	Sapur.013G055800	Sapur.013G070400	Sapur.013G108700	Sapur.013G141900
Sapur.014G010900	Sapur.014G014200	Sapur.014G017800	Sapur.014G030900	Sapur.014G043900	Sapur.014G050200
Sapur.014G054500	Sapur.014G065600	Sapur.014G065800	Sapur.014G067100	Sapur.014G067400	Sapur.014G069000
Sapur.014G084200	Sapur.014G091100	Sapur.014G095000	Sapur.014G095200	Sapur.014G096400	Sapur.014G110500
Sapur.014G119600	Sapur.014G122000	Sapur.014G126200	Sapur.016G034600	Sapur.016G045300	Sapur.016G046200
Sapur.016G048600	Sapur.016G049500	Sapur.016G064900	Sapur.016G066000	Sapur.016G089700	Sapur.016G090700
Sapur.016G113500	Sapur.016G115900	Sapur.016G122900	Sapur.016G133200	Sapur.016G133500	Sapur.016G161600
Sapur.016G164800	Sapur.016G165000	Sapur.016G165400	Sapur.016G183900	Sapur.016G222400	Sapur.016G228400
Sapur.016G256600	Sapur.016G263500	Sapur.016G267500	Sapur.016G267900	Sapur.016G269800	Sapur.016G272100
Sapur.016G274100	Sapur.016G293800	Sapur.016G294500	Sapur.016G294800	Sapur.016G304300	Sapur.017G001800
Sapur.017G002000	Sapur.017G019000	Sapur.017G019900	Sapur.017G067800	Sapur.017G072700	Sapur.017G096900
Sapur.017G097600	Sapur.017G107900	Sapur.017G109200	Sapur.017G111400	Sapur.017G114100	Sapur.018G005200
Sapur.018G010500	Sapur.018G010800	Sapur.018G010900	Sapur.018G011100	Sapur.018G011200	Sapur.018G018700
Sapur.018G035500	Sapur.018G048200	Sapur.018G053200	Sapur.018G058300	Sapur.018G058400	Sapur.018G060300
Sapur.018G067300	Sapur.018G072700	Sapur.018G082400	Sapur.018G086200	Sapur.018G104600	Sapur.018G107900
Sapur.018G114200	Sapur.019G008900	Sapur.019G016700	Sapur.019G022400	Sapur.019G053400	Sapur.019G061700
Sapur.019G062400	Sapur.019G083400	Sapur.019G099500	Sapur.019G113000	Sapur.15WG028200	Sapur.15WG031300
Sapur.15WG066000	Sapur.15WG072000	Sapur.15WG079300	Sapur.15ZG018000	Sapur.15ZG027900	Sapur.15ZG035300

				Sapur.15ZG042700 Sapur.15ZG054200 Sapur.15ZG082200 Sapur.15ZG085600 Sapur.15ZG093000 Sapur.15ZG124400 Sapur.15ZG130400 Sapur.15ZG134100 Sapur.T026500 Sapur.T044700 Sapur.T051500 Sapur.001G051400 Sapur.002G003700 Sapur.002G004900 Sapur.002G022300 Sapur.002G056400 Sapur.002G134700 Sapur.004G120300 Sapur.004G162500 Sapur.005G015300 Sapur.005G072700 Sapur.005G125000 Sapur.005G147300 Sapur.005G169900 Sapur.005G203300 Sapur.005G204800 Sapur.006G026100 Sapur.006G063300 Sapur.006G069300 Sapur.006G085900 Sapur.007G045200 Sapur.008G024600 Sapur.008G031500 Sapur.008G120900 Sapur.009G001100 Sapur.009G042700 Sapur.009G096300 Sapur.010G115800 Sapur.010G118000 Sapur.010G170600 Sapur.011G080400 Sapur.012G080500 Sapur.013G010500 Sapur.014G013900 Sapur.014G034800 Sapur.014G039700 Sapur.014G136300 Sapur.016G030700 Sapur.016G132100 Sapur.016G181700 Sapur.018G013300 Sapur.018G078800 Sapur.019G052800 Sapur.15ZG102400 Sapur.15ZG102900 Sapur.15ZG103100 Sapur.T045900 Sapur.016G095700 Sapur.016G248100 Sapur.006G054000 Sapur.008G149700 Sapur.010G023600
GO:0015318	63	202	inorganic molecular entity transmembrane transporter activity	Sapur.001G144500 Sapur.001G185600 Sapur.002G038200 Sapur.002G054000 Sapur.003G005100 Sapur.003G029600 Sapur.003G054000 Sapur.003G120000 Sapur.005G150200 Sapur.005G150300 Sapur.005G172700 Sapur.006G084000 Sapur.006G186200 Sapur.007G006700 Sapur.008G105500 Sapur.008G126700 Sapur.009G036200 Sapur.009G047600 Sapur.010G048300 Sapur.010G202000 Sapur.013G047900 Sapur.013G064900 Sapur.013G105800 Sapur.013G142400 Sapur.014G113300 Sapur.016G310000 Sapur.018G075900 Sapur.018G083400 Sapur.018G097400 Sapur.019G082100 Sapur.T031900 Sapur.002G020100 Sapur.002G021300 Sapur.002G066000 Sapur.005G186900 Sapur.005G188200 Sapur.006G116300 Sapur.006G180200 Sapur.006G205800 Sapur.007G012200 Sapur.008G113200 Sapur.009G062300 Sapur.009G099300 Sapur.010G173300 Sapur.014G092800 Sapur.016G197500 Sapur.016G231500 Sapur.016G231600 Sapur.017G051500 Sapur.017G065500 Sapur.018G026100 Sapur.018G041100 Sapur.T003500 Sapur.T012400 Sapur.003G108000 Sapur.007G049600 Sapur.15ZG006400 Sapur.008G162700 Sapur.013G090600 Sapur.003G100300 Sapur.005G058800 Sapur.016G098500 Sapur.017G027100
GO:0022890	51	160	inorganic cation transmembrane transporter activity	Sapur.001G144500 Sapur.002G054000 Sapur.003G005100 Sapur.003G054000 Sapur.003G120000 Sapur.005G150200 Sapur.005G150300 Sapur.005G172700 Sapur.006G084000 Sapur.006G186200 Sapur.007G006700 Sapur.009G036200 Sapur.009G047600 Sapur.010G048300 Sapur.010G202000 Sapur.013G047900 Sapur.013G064900 Sapur.013G105800 Sapur.013G142400 Sapur.014G113300 Sapur.018G075900 Sapur.018G083400 Sapur.019G082100 Sapur.T031900 Sapur.002G020100 Sapur.002G021300 Sapur.002G066000 Sapur.005G186900 Sapur.005G188200 Sapur.006G116300 Sapur.006G180200 Sapur.006G205800 Sapur.007G012200 Sapur.008G113200 Sapur.009G062300 Sapur.009G099300 Sapur.010G173300 Sapur.016G197500 Sapur.016G231500 Sapur.016G231600 Sapur.017G051500 Sapur.017G065500 Sapur.018G041100 Sapur.T003500 Sapur.T012400 Sapur.008G162700 Sapur.013G090600 Sapur.003G100300 Sapur.005G058800 Sapur.016G098500 Sapur.017G027100
GO:0017171	49	153	serine hydrolase activity	Sapur.001G001400 Sapur.001G088500 Sapur.002G009400 Sapur.002G063400 Sapur.002G072700 Sapur.002G097300 Sapur.002G101500 Sapur.002G124000 Sapur.003G037900 Sapur.003G087600 Sapur.003G087900

				Sapur.003G088100 Sapur.004G164900 Sapur.005G122400 Sapur.005G194900 Sapur.006G061500 Sapur.006G070700 Sapur.006G161800 Sapur.006G169900 Sapur.007G042300 Sapur.007G116000 Sapur.008G030600 Sapur.009G023600 Sapur.010G176400 Sapur.012G101000 Sapur.012G107200 Sapur.014G096400 Sapur.016G133500 Sapur.016G294800 Sapur.018G058400 Sapur.15ZG130400 Sapur.T026500 Sapur.001G051400 Sapur.002G056400 Sapur.004G162500 Sapur.005G072700 Sapur.005G147300 Sapur.006G026100 Sapur.008G024600 Sapur.008G031500 Sapur.009G001100 Sapur.009G042700 Sapur.010G115800 Sapur.012G080500 Sapur.016G030700 Sapur.018G078800 Sapur.019G052800 Sapur.15ZG102400 Sapur.T045900
GO:0008236	49	153	serine-type peptidase activity	Sapur.001G001400 Sapur.001G088500 Sapur.002G009400 Sapur.002G063400 Sapur.002G072700 Sapur.002G097300 Sapur.002G101500 Sapur.002G124000 Sapur.003G037900 Sapur.003G087600 Sapur.003G087900 Sapur.003G088100 Sapur.004G164900 Sapur.005G122400 Sapur.005G194900 Sapur.006G061500 Sapur.006G070700 Sapur.006G161800 Sapur.006G169900 Sapur.007G042300 Sapur.007G116000 Sapur.008G030600 Sapur.009G023600 Sapur.010G176400 Sapur.012G101000 Sapur.012G107200 Sapur.014G096400 Sapur.016G133500 Sapur.016G294800 Sapur.018G058400 Sapur.15ZG130400 Sapur.T026500 Sapur.001G051400 Sapur.002G056400 Sapur.004G162500 Sapur.005G072700 Sapur.005G147300 Sapur.006G026100 Sapur.008G024600 Sapur.008G031500 Sapur.009G001100 Sapur.009G042700 Sapur.010G115800 Sapur.012G080500 Sapur.016G030700 Sapur.018G078800 Sapur.019G052800 Sapur.15ZG102400 Sapur.T045900
GO:0008324	53	172	monoatomic cation transmembrane transporter activity	Sapur.001G144500 Sapur.002G054000 Sapur.003G005100 Sapur.003G054000 Sapur.003G120000 Sapur.005G150200 Sapur.005G150300 Sapur.005G172700 Sapur.006G186200 Sapur.007G006700 Sapur.009G036200 Sapur.009G047600 Sapur.010G202000 Sapur.013G047900 Sapur.013G064900 Sapur.013G105800 Sapur.013G142400 Sapur.014G113300 Sapur.018G075900 Sapur.018G083400 Sapur.019G082100 Sapur.T031900 Sapur.002G020100 Sapur.002G021300 Sapur.002G066000 Sapur.005G186900 Sapur.005G188200 Sapur.006G116300 Sapur.006G180200 Sapur.006G205800 Sapur.007G012200 Sapur.008G113200 Sapur.009G062300 Sapur.009G099300 Sapur.010G173300 Sapur.016G197500 Sapur.016G231500 Sapur.016G231600 Sapur.017G051500 Sapur.017G065500 Sapur.018G041100 Sapur.T003500 Sapur.T012400 Sapur.008G162700 Sapur.010G133600 Sapur.013G090600 Sapur.014G083800 Sapur.016G040900 Sapur.017G095100 Sapur.003G100300 Sapur.005G058800 Sapur.016G098500 Sapur.017G027100
GO:0008375	23	47	acetylglucosaminyltransferase activity	Sapur.001G043500 Sapur.001G185400 Sapur.004G103700 Sapur.004G155200 Sapur.006G062000 Sapur.006G192900 Sapur.006G217000 Sapur.008G003400 Sapur.008G011900 Sapur.008G103100 Sapur.010G087400 Sapur.010G092800 Sapur.011G008300 Sapur.013G068300 Sapur.014G136300 Sapur.016G113700 Sapur.016G221000 Sapur.017G062700 Sapur.018G110200 Sapur.019G099900 Sapur.006G054000 Sapur.008G149700 Sapur.010G023600
GO:0016798	92	379	hydrolase activity, acting on glycosyl bonds	Sapur.001G072900 Sapur.001G112100 Sapur.001G167900 Sapur.001G171300 Sapur.002G017900 Sapur.002G064200 Sapur.002G125200 Sapur.002G162300 Sapur.002G182500 Sapur.002G184100 Sapur.003G013400 Sapur.003G022500 Sapur.003G024100 Sapur.003G063600 Sapur.003G112400 Sapur.003G118000 Sapur.003G165600 Sapur.004G011800 Sapur.004G012100 Sapur.004G102500 Sapur.004G129600 Sapur.004G152100 Sapur.005G052600

				Sapur.005G118500 Sapur.005G190700 Sapur.006G001400 Sapur.006G049200 Sapur.006G090000 Sapur.006G119400 Sapur.006G180700 Sapur.007G051800 Sapur.007G064600 Sapur.007G089900 Sapur.007G090100 Sapur.008G004000 Sapur.008G038800 Sapur.008G043700 Sapur.008G043800 Sapur.008G062900 Sapur.008G112200 Sapur.009G003700 Sapur.009G009100 Sapur.009G065500 Sapur.009G068900 Sapur.009G120900 Sapur.009G128100 Sapur.010G021300 Sapur.010G071500 Sapur.010G082200 Sapur.010G110600 Sapur.010G110700 Sapur.010G124000 Sapur.010G160100 Sapur.010G167500 Sapur.010G203100 Sapur.011G002500 Sapur.011G072100 Sapur.011G112600 Sapur.012G033500 Sapur.013G005200 Sapur.013G113400 Sapur.013G117800 Sapur.013G119000 Sapur.014G072000 Sapur.014G091800 Sapur.014G097700 Sapur.014G116700 Sapur.014G118900 Sapur.016G000600 Sapur.016G054400 Sapur.016G054600 Sapur.016G222900 Sapur.016G287300 Sapur.016G289400 Sapur.017G046300 Sapur.018G061200 Sapur.018G086200 Sapur.018G094800 Sapur.018G101200 Sapur.018G108000 Sapur.019G037200 Sapur.019G109000 Sapur.15WG064400 Sapur.15ZG052500 Sapur.15ZG072800 Sapur.15ZG118900 Sapur.002G108700 Sapur.006G151000 Sapur.008G090000 Sapur.010G107500 Sapur.016G023600 Sapur.003G077800
GO:0022853	19	38	active monoatomic ion transmembrane transporter activity	Sapur.001G144500 Sapur.002G054000 Sapur.003G054000 Sapur.003G120000 Sapur.005G150200 Sapur.005G150300 Sapur.005G172700 Sapur.007G006700 Sapur.010G202000 Sapur.013G047900 Sapur.013G064900 Sapur.013G142400 Sapur.018G075900 Sapur.018G083400 Sapur.T031900 Sapur.003G100300 Sapur.005G058800 Sapur.016G098500 Sapur.017G027100
GO:0003924	26	67	GTPase activity	Sapur.001G086500 Sapur.001G185700 Sapur.002G015900 Sapur.002G034900 Sapur.002G090500 Sapur.002G146800 Sapur.003G000800 Sapur.003G090100 Sapur.003G091100 Sapur.003G166900 Sapur.005G192400 Sapur.006G025400 Sapur.006G150200 Sapur.006G180500 Sapur.009G052100 Sapur.009G052200 Sapur.009G066800 Sapur.010G069300 Sapur.011G119600 Sapur.016G030100 Sapur.016G094800 Sapur.016G158100 Sapur.016G158200 Sapur.016G172500 Sapur.018G081000 Sapur.019G035200
GO:0016740	504	3067	transferase activity	Sapur.001G015800 Sapur.001G033500 Sapur.001G042400 Sapur.001G043100 Sapur.001G066800 Sapur.001G078000 Sapur.001G086800 Sapur.001G093700 Sapur.001G103900 Sapur.001G137500 Sapur.001G148700 Sapur.001G171200 Sapur.001G175900 Sapur.001G190300 Sapur.001G191000 Sapur.002G010500 Sapur.002G028000 Sapur.002G046900 Sapur.002G055700 Sapur.002G087900 Sapur.002G106900 Sapur.002G119600 Sapur.002G120400 Sapur.002G164000 Sapur.002G176000 Sapur.002G180800 Sapur.002G188100 Sapur.002G192000 Sapur.002G198100 Sapur.002G199800 Sapur.002G201200 Sapur.002G201300 Sapur.003G013500 Sapur.003G036900 Sapur.003G037300 Sapur.003G042700 Sapur.003G051100 Sapur.003G052500 Sapur.003G061800 Sapur.003G067600 Sapur.003G073200 Sapur.003G075500 Sapur.003G082200 Sapur.003G085300 Sapur.003G089700 Sapur.003G095700 Sapur.003G099700 Sapur.003G131900 Sapur.003G133000 Sapur.003G138700 Sapur.004G009000 Sapur.004G017100 Sapur.004G023900 Sapur.004G029200 Sapur.004G032900 Sapur.004G033000 Sapur.004G044400 Sapur.004G046500 Sapur.004G057300 Sapur.004G061000 Sapur.004G067800 Sapur.004G068600 Sapur.004G080700 Sapur.004G087900 Sapur.004G115800 Sapur.004G121200 Sapur.004G135100 Sapur.004G168300 Sapur.004G170800 Sapur.004G172200 Sapur.004G177200

Sapur.005G001800	Sapur.005G011700	Sapur.005G031100	Sapur.005G039800	Sapur.005G050800	Sapur.005G057200
Sapur.005G065000	Sapur.005G065300	Sapur.005G081600	Sapur.005G109200	Sapur.005G143900	Sapur.005G148400
Sapur.005G160000	Sapur.005G202000	Sapur.005G203500	Sapur.005G205600	Sapur.005G208100	Sapur.006G039400
Sapur.006G043700	Sapur.006G044000	Sapur.006G059800	Sapur.006G063800	Sapur.006G064600	Sapur.006G077000
Sapur.006G088300	Sapur.006G090100	Sapur.006G093700	Sapur.006G096300	Sapur.006G107000	Sapur.006G117000
Sapur.006G141900	Sapur.006G151400	Sapur.006G185100	Sapur.006G195000	Sapur.006G196200	Sapur.006G210500
Sapur.006G215000	Sapur.006G217500	Sapur.007G003100	Sapur.007G012300	Sapur.007G037800	Sapur.007G044000
Sapur.007G054000	Sapur.007G077400	Sapur.007G085900	Sapur.007G094200	Sapur.007G099400	Sapur.007G124000
Sapur.007G124100	Sapur.008G008800	Sapur.008G016100	Sapur.008G054200	Sapur.008G054500	Sapur.008G076100
Sapur.008G089200	Sapur.008G103500	Sapur.008G107400	Sapur.008G111800	Sapur.008G113700	Sapur.008G116800
Sapur.008G117900	Sapur.008G136200	Sapur.008G136400	Sapur.008G143200	Sapur.008G165900	Sapur.009G030800
Sapur.009G058900	Sapur.009G059300	Sapur.009G078200	Sapur.009G091500	Sapur.009G092000	Sapur.009G107300
Sapur.010G000800	Sapur.010G028600	Sapur.010G041500	Sapur.010G052000	Sapur.010G064500	Sapur.010G070900
Sapur.010G075700	Sapur.010G076000	Sapur.010G076900	Sapur.010G127100	Sapur.010G145100	Sapur.010G147600
Sapur.010G147800	Sapur.010G169400	Sapur.010G189000	Sapur.011G021000	Sapur.011G030700	Sapur.011G036900
Sapur.011G040500	Sapur.011G040600	Sapur.011G046900	Sapur.011G047800	Sapur.011G050200	Sapur.011G050900
Sapur.011G080000	Sapur.011G080600	Sapur.011G089900	Sapur.011G095700	Sapur.011G097200	Sapur.011G101300
Sapur.011G108300	Sapur.011G121500	Sapur.012G002900	Sapur.012G020400	Sapur.012G029700	Sapur.012G032600
Sapur.012G041400	Sapur.012G048500	Sapur.012G051500	Sapur.012G052000	Sapur.012G054500	Sapur.012G063200
Sapur.012G064400	Sapur.012G064900	Sapur.012G071500	Sapur.012G093400	Sapur.012G097700	Sapur.012G106800
Sapur.013G003400	Sapur.013G034500	Sapur.013G055800	Sapur.013G108700	Sapur.013G141900	Sapur.014G014200
Sapur.014G017800	Sapur.014G049700	Sapur.014G050200	Sapur.014G054500	Sapur.014G065600	Sapur.014G067100
Sapur.014G067400	Sapur.014G069000	Sapur.014G084200	Sapur.014G091100	Sapur.014G095200	Sapur.014G110500
Sapur.014G119600	Sapur.014G126200	Sapur.016G045300	Sapur.016G046200	Sapur.016G048600	Sapur.016G049500
Sapur.016G064900	Sapur.016G089700	Sapur.016G090700	Sapur.016G113500	Sapur.016G115900	Sapur.016G122900
Sapur.016G133200	Sapur.016G164800	Sapur.016G165400	Sapur.016G182600	Sapur.016G195400	Sapur.016G222400
Sapur.016G256600	Sapur.016G263500	Sapur.016G267500	Sapur.016G267900	Sapur.016G269800	Sapur.016G272100
Sapur.016G274100	Sapur.016G293800	Sapur.016G294500	Sapur.016G304300	Sapur.017G001800	Sapur.017G002000
Sapur.017G019000	Sapur.017G019900	Sapur.017G050000	Sapur.017G072700	Sapur.017G096900	Sapur.017G097600
Sapur.017G107900	Sapur.017G111400	Sapur.017G114100	Sapur.018G018700	Sapur.018G035500	Sapur.018G048200
Sapur.018G053200	Sapur.018G058300	Sapur.018G060300	Sapur.018G067300	Sapur.018G072700	Sapur.018G082400
Sapur.018G086200	Sapur.018G107900	Sapur.018G114200	Sapur.019G008900	Sapur.019G061700	Sapur.019G062400
Sapur.019G083400	Sapur.019G099500	Sapur.019G113000	Sapur.15WG028200	Sapur.15WG031300	Sapur.15WG066000

Sapur.15WG079300 Sapur.15ZG018000 Sapur.15ZG027900 Sapur.15ZG035300 Sapur.15ZG054200 Sapur.15ZG082200
Sapur.15ZG085600 Sapur.15ZG093000 Sapur.15ZG124400 Sapur.15ZG134100 Sapur.T044700 Sapur.T051500
Sapur.001G043500 Sapur.001G107900 Sapur.001G112600 Sapur.001G121300 Sapur.001G161400 Sapur.001G185400
Sapur.002G001800 Sapur.002G004300 Sapur.002G011200 Sapur.002G018700 Sapur.002G028300 Sapur.002G028400
Sapur.002G047800 Sapur.002G059600 Sapur.002G060900 Sapur.002G072000 Sapur.002G086200 Sapur.002G108100
Sapur.002G108300 Sapur.002G126200 Sapur.002G149100 Sapur.002G160200 Sapur.002G168400 Sapur.002G176800
Sapur.002G191300 Sapur.002G203000 Sapur.003G010200 Sapur.003G038600 Sapur.003G060700 Sapur.003G063400
Sapur.003G068300 Sapur.003G095000 Sapur.003G099600 Sapur.004G076800 Sapur.004G103700 Sapur.004G106300
Sapur.004G114100 Sapur.004G114400 Sapur.004G138500 Sapur.004G154700 Sapur.004G155200 Sapur.004G177100
Sapur.005G047100 Sapur.005G049400 Sapur.005G067900 Sapur.005G125100 Sapur.005G147500 Sapur.005G173800
Sapur.005G174000 Sapur.005G180300 Sapur.005G189700 Sapur.005G204000 Sapur.005G206700 Sapur.006G008700
Sapur.006G015700 Sapur.006G031900 Sapur.006G036700 Sapur.006G042500 Sapur.006G062000 Sapur.006G093600
Sapur.006G101400 Sapur.006G107800 Sapur.006G126300 Sapur.006G192900 Sapur.006G194700 Sapur.006G198700
Sapur.006G212700 Sapur.006G217000 Sapur.007G018200 Sapur.007G018500 Sapur.007G018700 Sapur.007G029400
Sapur.007G044700 Sapur.007G074100 Sapur.007G078800 Sapur.007G093300 Sapur.007G096100 Sapur.007G100600
Sapur.008G003400 Sapur.008G011900 Sapur.008G028500 Sapur.008G046900 Sapur.008G070800 Sapur.008G072800
Sapur.008G074800 Sapur.008G078200 Sapur.008G094100 Sapur.008G103100 Sapur.008G119300 Sapur.008G162300
Sapur.009G044900 Sapur.009G088500 Sapur.009G113800 Sapur.009G130700 Sapur.010G015200 Sapur.010G019700
Sapur.010G031200 Sapur.010G078700 Sapur.010G087400 Sapur.010G092800 Sapur.010G099900 Sapur.010G118100
Sapur.010G119300 Sapur.010G119600 Sapur.010G122700 Sapur.010G129800 Sapur.010G144700 Sapur.010G156300
Sapur.010G178800 Sapur.010G199900 Sapur.011G008300 Sapur.011G009700 Sapur.011G032700 Sapur.011G032800
Sapur.011G033200 Sapur.011G098300 Sapur.011G109500 Sapur.011G112000 Sapur.011G122000 Sapur.012G034900
Sapur.012G045600 Sapur.012G050000 Sapur.012G052600 Sapur.012G083700 Sapur.013G024200 Sapur.013G045800
Sapur.013G068300 Sapur.013G106000 Sapur.013G129800 Sapur.013G132500 Sapur.014G018800 Sapur.014G030400
Sapur.014G055700 Sapur.014G059500 Sapur.014G059800 Sapur.014G077500 Sapur.014G091500 Sapur.014G100100
Sapur.014G116400 Sapur.014G118100 Sapur.014G125100 Sapur.014G136300 Sapur.016G001300 Sapur.016G010900
Sapur.016G047300 Sapur.016G082300 Sapur.016G113700 Sapur.016G148200 Sapur.016G149600 Sapur.016G215600
Sapur.016G221000 Sapur.016G244800 Sapur.016G252400 Sapur.016G253800 Sapur.016G266500 Sapur.016G275400
Sapur.016G290200 Sapur.017G062700 Sapur.017G074700 Sapur.017G086800 Sapur.017G100800 Sapur.017G111600
Sapur.018G060800 Sapur.018G064500 Sapur.018G110200 Sapur.019G098500 Sapur.019G099900 Sapur.019G100200
Sapur.15ZG018200 Sapur.15ZG033400 Sapur.15ZG038800 Sapur.15ZG060800 Sapur.15ZG080600 Sapur.15ZG101800
Sapur.T004100 Sapur.T052100 Sapur.T058900 Sapur.T153300 Sapur.001G026000 Sapur.001G032700
Sapur.001G105100 Sapur.002G061200 Sapur.002G146100 Sapur.002G170400 Sapur.003G043700 Sapur.003G139500

				Sapur.004G036700 Sapur.004G052000 Sapur.004G095900 Sapur.004G116800 Sapur.005G184400 Sapur.006G145900 Sapur.006G206200 Sapur.009G019400 Sapur.009G033500 Sapur.009G079300 Sapur.010G057200 Sapur.011G107900 Sapur.013G013900 Sapur.013G047900 Sapur.014G040300 Sapur.014G074600 Sapur.014G105300 Sapur.016G020000 Sapur.016G020300 Sapur.016G127900 Sapur.016G288100 Sapur.016G297800 Sapur.017G040100 Sapur.017G078500 Sapur.018G007400 Sapur.018G026500 Sapur.019G079900 Sapur.15ZG020900 Sapur.15ZG117500 Sapur.T031900 Sapur.004G005600 Sapur.006G054000 Sapur.008G149700 Sapur.010G023600 Sapur.011G005900 Sapur.018G062800 Sapur.15ZG021300
GO:0005507	30	85	copper ion binding	Sapur.001G098800 Sapur.003G077900 Sapur.004G006100 Sapur.004G135900 Sapur.005G198500 Sapur.006G071400 Sapur.006G071900 Sapur.006G079700 Sapur.006G079800 Sapur.007G020600 Sapur.007G036000 Sapur.008G050800 Sapur.008G058600 Sapur.008G058700 Sapur.009G025400 Sapur.009G025500 Sapur.009G080300 Sapur.010G143300 Sapur.010G143400 Sapur.010G151400 Sapur.011G083300 Sapur.016G094000 Sapur.016G097600 Sapur.016G139000 Sapur.016G215200 Sapur.016G258100 Sapur.016G259100 Sapur.016G259200 Sapur.016G259300 Sapur.T185900
GO:0004553	83	357	hydrolase activity, hydrolyzing O-glycosyl compounds	Sapur.001G072900 Sapur.001G112100 Sapur.001G167900 Sapur.001G171300 Sapur.002G017900 Sapur.002G064200 Sapur.002G125200 Sapur.002G162300 Sapur.002G182500 Sapur.002G184100 Sapur.003G013400 Sapur.003G022500 Sapur.003G024100 Sapur.003G063600 Sapur.003G112400 Sapur.003G118000 Sapur.003G165600 Sapur.004G011800 Sapur.004G012100 Sapur.004G102500 Sapur.004G129600 Sapur.004G152100 Sapur.005G052600 Sapur.005G118500 Sapur.005G190700 Sapur.006G001400 Sapur.006G090000 Sapur.006G119400 Sapur.006G180700 Sapur.007G051800 Sapur.007G064600 Sapur.007G089900 Sapur.007G090100 Sapur.008G004000 Sapur.008G038800 Sapur.008G043700 Sapur.008G043800 Sapur.008G062900 Sapur.008G112200 Sapur.009G003700 Sapur.009G009100 Sapur.009G065500 Sapur.009G068900 Sapur.009G120900 Sapur.009G128100 Sapur.010G021300 Sapur.010G071500 Sapur.010G082200 Sapur.010G110600 Sapur.010G110700 Sapur.010G160100 Sapur.010G167500 Sapur.010G203100 Sapur.011G002500 Sapur.011G072100 Sapur.011G112600 Sapur.012G033500 Sapur.013G005200 Sapur.013G113400 Sapur.013G117800 Sapur.013G119000 Sapur.014G072000 Sapur.014G091800 Sapur.014G097700 Sapur.014G116700 Sapur.014G118900 Sapur.016G000600 Sapur.016G054400 Sapur.016G054600 Sapur.016G222900 Sapur.016G287300 Sapur.016G289400 Sapur.017G046300 Sapur.018G061200 Sapur.018G086200 Sapur.018G101200 Sapur.018G108000 Sapur.019G037200 Sapur.019G109000 Sapur.15ZG072800 Sapur.15ZG118900 Sapur.016G023600 Sapur.003G077800
GO:0015020	7	7	glucuronosyltransferase activity	Sapur.002G086200 Sapur.005G125100 Sapur.006G107800 Sapur.006G198700 Sapur.007G044700 Sapur.014G018800 Sapur.016G082300
GO:0008378	14	26	galactosyltransferase activity	Sapur.002G072000 Sapur.002G176800 Sapur.003G099600 Sapur.006G008700 Sapur.006G212700 Sapur.010G118100 Sapur.010G129800 Sapur.011G112000 Sapur.012G052600 Sapur.014G125100 Sapur.016G010900 Sapur.016G252400 Sapur.016G290200 Sapur.15ZG018200
GO:001	63	257	monoatomic ion	Sapur.001G144500 Sapur.001G185600 Sapur.002G054000 Sapur.003G005100 Sapur.003G054000

5075			transmembrane transporter activity	Sapur.003G120000 Sapur.005G150200 Sapur.005G150300 Sapur.005G172700 Sapur.006G186200 Sapur.007G006700 Sapur.009G036200 Sapur.009G047600 Sapur.010G202000 Sapur.013G047900 Sapur.013G064900 Sapur.013G105800 Sapur.013G142400 Sapur.014G113300 Sapur.016G310000 Sapur.018G075900 Sapur.018G083400 Sapur.018G097400 Sapur.019G082100 Sapur.T031900 Sapur.001G033600 Sapur.002G020100 Sapur.002G021300 Sapur.002G066000 Sapur.003G138600 Sapur.005G186900 Sapur.005G188200 Sapur.006G116300 Sapur.006G180200 Sapur.006G205800 Sapur.007G012200 Sapur.008G113200 Sapur.009G006300 Sapur.009G062300 Sapur.009G099300 Sapur.010G173300 Sapur.012G025200 Sapur.016G197500 Sapur.016G231500 Sapur.016G231600 Sapur.017G051500 Sapur.017G056200 Sapur.017G065500 Sapur.017G073700 Sapur.018G009200 Sapur.018G041100 Sapur.T003500 Sapur.T012400 Sapur.008G162700 Sapur.010G133600 Sapur.013G090600 Sapur.014G083800 Sapur.016G040900 Sapur.017G095100 Sapur.003G100300 Sapur.005G058800 Sapur.016G098500 Sapur.017G027100
GO:0009055	42	151	electron transfer activity	Sapur.001G033400 Sapur.001G089300 Sapur.001G144500 Sapur.001G173200 Sapur.002G054000 Sapur.002G057800 Sapur.002G081200 Sapur.002G081300 Sapur.002G197600 Sapur.003G008700 Sapur.003G086600 Sapur.003G120000 Sapur.003G153700 Sapur.004G152300 Sapur.005G150200 Sapur.005G150300 Sapur.006G030600 Sapur.006G164600 Sapur.006G204700 Sapur.007G006700 Sapur.007G121300 Sapur.008G013500 Sapur.008G114600 Sapur.008G116700 Sapur.008G122100 Sapur.009G059000 Sapur.009G108500 Sapur.009G129200 Sapur.010G059500 Sapur.013G096200 Sapur.013G142400 Sapur.016G018000 Sapur.016G034600 Sapur.016G045200 Sapur.016G165000 Sapur.016G206000 Sapur.017G067800 Sapur.017G072900 Sapur.017G073000 Sapur.018G075900 Sapur.018G083400 Sapur.018G104600
GO:0004576	8	10	oligosaccharyl transferase activity	Sapur.001G112600 Sapur.002G004300 Sapur.003G063400 Sapur.005G204000 Sapur.008G028500 Sapur.010G178800 Sapur.018G062800 Sapur.15ZG021300
GO:0015018	6	6	galactosylgalactosylxylosylprotein 3-beta-glucuronosyltransferase activity	Sapur.002G086200 Sapur.005G125100 Sapur.006G107800 Sapur.006G198700 Sapur.007G044700 Sapur.016G082300
GO:0004579	6	6	dolichyl-diphosphooligosaccharide-protein glycotransferase activity	Sapur.001G112600 Sapur.002G004300 Sapur.003G063400 Sapur.005G204000 Sapur.008G028500 Sapur.010G178800
GO:001	9	13	mannosidase	Sapur.002G184100 Sapur.004G102500 Sapur.007G051800 Sapur.008G004000 Sapur.010G203100

5923			activity	Sapur.014G116700 Sapur.016G222900 Sapur.018G108000 Sapur.15ZG072800
GO:0004559	9	13	alpha-mannosidase activity	Sapur.002G184100 Sapur.004G102500 Sapur.007G051800 Sapur.008G004000 Sapur.010G203100 Sapur.014G116700 Sapur.016G222900 Sapur.018G108000 Sapur.15ZG072800
GO:0015453	12	22	oxidoreduction-driven active transmembrane transporter activity	Sapur.001G089300 Sapur.001G144500 Sapur.002G054000 Sapur.003G086600 Sapur.003G120000 Sapur.005G150200 Sapur.005G150300 Sapur.007G006700 Sapur.008G114600 Sapur.013G142400 Sapur.018G075900 Sapur.018G083400
GO:0004185	17	42	serine-type carboxypeptidase activity	Sapur.001G051400 Sapur.002G056400 Sapur.004G162500 Sapur.005G072700 Sapur.005G147300 Sapur.006G026100 Sapur.008G024600 Sapur.008G031500 Sapur.009G001100 Sapur.009G042700 Sapur.010G115800 Sapur.012G080500 Sapur.016G030700 Sapur.018G078800 Sapur.019G052800 Sapur.15ZG102400 Sapur.T045900
GO:0004252	28	92	serine-type endopeptidase activity	Sapur.001G001400 Sapur.001G088500 Sapur.002G009400 Sapur.002G072700 Sapur.002G097300 Sapur.002G101500 Sapur.002G124000 Sapur.003G037900 Sapur.003G087600 Sapur.003G087900 Sapur.003G088100 Sapur.005G122400 Sapur.005G194900 Sapur.006G061500 Sapur.006G070700 Sapur.006G161800 Sapur.007G042300 Sapur.007G116000 Sapur.008G030600 Sapur.009G023600 Sapur.010G176400 Sapur.012G101000 Sapur.012G107200 Sapur.016G133500 Sapur.016G294800 Sapur.018G058400 Sapur.15ZG130400 Sapur.T026500
GO:0070008	18	48	serine-type exopeptidase activity	Sapur.001G051400 Sapur.002G056400 Sapur.004G162500 Sapur.005G072700 Sapur.005G147300 Sapur.006G026100 Sapur.008G024600 Sapur.008G031500 Sapur.009G001100 Sapur.009G042700 Sapur.010G115800 Sapur.012G080500 Sapur.016G030700 Sapur.018G078800 Sapur.019G052800 Sapur.15ZG102400 Sapur.T045900 Sapur.002G009400
GO:0004180	17	44	carboxypeptidase activity	Sapur.001G051400 Sapur.002G056400 Sapur.004G162500 Sapur.005G072700 Sapur.005G147300 Sapur.006G026100 Sapur.008G024600 Sapur.008G031500 Sapur.009G001100 Sapur.009G042700 Sapur.010G115800 Sapur.012G080500 Sapur.016G030700 Sapur.018G078800 Sapur.019G052800 Sapur.15ZG102400 Sapur.T045900
GO:0005215	137	729	transporter activity	Sapur.002G110800 Sapur.010G090500 Sapur.016G242000 Sapur.T001500 Sapur.001G037600 Sapur.001G089300 Sapur.001G144500 Sapur.001G164900 Sapur.001G185600 Sapur.001G187000 Sapur.002G038200 Sapur.002G054000 Sapur.002G098200 Sapur.002G193600 Sapur.003G005100 Sapur.003G029600 Sapur.003G054000 Sapur.003G061600 Sapur.003G076900 Sapur.003G086600 Sapur.003G092800 Sapur.003G115900 Sapur.003G120000 Sapur.003G149100 Sapur.004G021600 Sapur.004G025800 Sapur.004G132700 Sapur.004G141700 Sapur.004G168100 Sapur.004G168900 Sapur.005G031200 Sapur.005G150200 Sapur.005G150300 Sapur.005G172700 Sapur.005G204400 Sapur.006G023800 Sapur.006G037500 Sapur.006G049300 Sapur.006G084000 Sapur.006G102100 Sapur.006G105100 Sapur.006G105200 Sapur.006G186200 Sapur.006G198200 Sapur.007G006700 Sapur.008G005900 Sapur.008G038400 Sapur.008G105500 Sapur.008G114600 Sapur.008G126700 Sapur.009G036200 Sapur.009G047600 Sapur.009G063600 Sapur.009G068000 Sapur.009G090000 Sapur.009G109000 Sapur.009G117600 Sapur.010G007300 Sapur.010G048300 Sapur.010G073400

				Sapur.010G150300 Sapur.010G165600 Sapur.010G202000 Sapur.011G050700 Sapur.012G040200 Sapur.013G047900 Sapur.013G064900 Sapur.013G100800 Sapur.013G101200 Sapur.013G105800 Sapur.013G142400 Sapur.014G033300 Sapur.014G089900 Sapur.014G104400 Sapur.014G113300 Sapur.016G047900 Sapur.016G096400 Sapur.016G097500 Sapur.016G147600 Sapur.016G226600 Sapur.016G310000 Sapur.017G067400 Sapur.018G075900 Sapur.018G083400 Sapur.018G097400 Sapur.018G101700 Sapur.019G076600 Sapur.019G082100 Sapur.15WG078100 Sapur.15ZG073700 Sapur.T031900 Sapur.T047800 Sapur.001G033600 Sapur.002G020100 Sapur.002G021300 Sapur.002G066000 Sapur.003G138600 Sapur.005G186900 Sapur.005G188200 Sapur.006G116300 Sapur.006G180200 Sapur.006G205800 Sapur.007G012200 Sapur.008G113200 Sapur.008G142900 Sapur.009G006300 Sapur.009G062300 Sapur.009G099300 Sapur.010G042500 Sapur.010G173300 Sapur.012G025200 Sapur.014G092800 Sapur.016G197500 Sapur.016G231500 Sapur.016G231600 Sapur.017G051500 Sapur.017G056200 Sapur.017G065500 Sapur.017G073700 Sapur.018G009200 Sapur.018G026100 Sapur.018G041100 Sapur.T003500 Sapur.T012400 Sapur.003G108000 Sapur.007G049600 Sapur.15ZG006400 Sapur.008G162700 Sapur.010G133600 Sapur.013G090600 Sapur.014G083800 Sapur.016G040900 Sapur.017G095100 Sapur.003G100300 Sapur.005G058800 Sapur.016G098500 Sapur.017G027100
GO:0003854	15	37	3-beta-hydroxy-delta5-steroid dehydrogenase activity	Sapur.002G002500 Sapur.002G103600 Sapur.002G119700 Sapur.002G157000 Sapur.003G079300 Sapur.006G175000 Sapur.008G041000 Sapur.010G163100 Sapur.014G049800 Sapur.014G103300 Sapur.016G074400 Sapur.016G130500 Sapur.016G240600 Sapur.016G246100 Sapur.017G049800
GO:0033764	15	37	steroid dehydrogenase activity, acting on the CH-OH group of donors, NAD or NADP as acceptor	Sapur.002G002500 Sapur.002G103600 Sapur.002G119700 Sapur.002G157000 Sapur.003G079300 Sapur.006G175000 Sapur.008G041000 Sapur.010G163100 Sapur.014G049800 Sapur.014G103300 Sapur.016G074400 Sapur.016G130500 Sapur.016G240600 Sapur.016G246100 Sapur.017G049800
GO:0016229	15	37	steroid dehydrogenase activity	Sapur.002G002500 Sapur.002G103600 Sapur.002G119700 Sapur.002G157000 Sapur.003G079300 Sapur.006G175000 Sapur.008G041000 Sapur.010G163100 Sapur.014G049800 Sapur.014G103300 Sapur.016G074400 Sapur.016G130500 Sapur.016G240600 Sapur.016G246100 Sapur.017G049800
GO:0005525	54	236	GTP binding	Sapur.001G122500 Sapur.001G127100 Sapur.001G184300 Sapur.001G185700 Sapur.002G013800 Sapur.002G015100 Sapur.002G034900 Sapur.002G049800 Sapur.002G058200 Sapur.002G109900 Sapur.002G112600 Sapur.003G000800 Sapur.003G028100 Sapur.003G050300 Sapur.003G054600 Sapur.004G061100 Sapur.004G115200 Sapur.005G006600 Sapur.005G048100 Sapur.005G058600 Sapur.006G000900 Sapur.006G140000 Sapur.006G150200 Sapur.007G094400 Sapur.008G025400 Sapur.008G078900 Sapur.009G020400 Sapur.009G068500 Sapur.009G090900 Sapur.009G107200 Sapur.009G124500 Sapur.010G069300 Sapur.010G110900 Sapur.010G118800 Sapur.010G183900 Sapur.011G042000 Sapur.011G042200 Sapur.011G100700 Sapur.012G057100 Sapur.012G086800 Sapur.012G088300

				Sapur.013G009200 Sapur.013G009300 Sapur.013G084200 Sapur.013G112200 Sapur.013G112400 Sapur.014G039100 Sapur.016G174600 Sapur.016G277800 Sapur.018G009700 Sapur.018G072500 Sapur.018G081000 Sapur.019G087800 Sapur.019G087900
GO:0019001	54	236	guanyl nucleotide binding	Sapur.001G122500 Sapur.001G127100 Sapur.001G184300 Sapur.001G185700 Sapur.002G013800 Sapur.002G015100 Sapur.002G034900 Sapur.002G049800 Sapur.002G058200 Sapur.002G109900 Sapur.002G112600 Sapur.003G000800 Sapur.003G028100 Sapur.003G050300 Sapur.003G054600 Sapur.004G061100 Sapur.004G115200 Sapur.005G006600 Sapur.005G048100 Sapur.005G058600 Sapur.006G000900 Sapur.006G140000 Sapur.006G150200 Sapur.007G094400 Sapur.008G025400 Sapur.008G078900 Sapur.009G020400 Sapur.009G068500 Sapur.009G090900 Sapur.009G107200 Sapur.009G124500 Sapur.010G069300 Sapur.010G110900 Sapur.010G118800 Sapur.010G183900 Sapur.011G042000 Sapur.011G042200 Sapur.011G100700 Sapur.012G057100 Sapur.012G086800 Sapur.012G088300 Sapur.013G009200 Sapur.013G009300 Sapur.013G084200 Sapur.013G112200 Sapur.013G112400 Sapur.014G039100 Sapur.016G174600 Sapur.016G277800 Sapur.018G009700 Sapur.018G072500 Sapur.018G081000 Sapur.019G087800 Sapur.019G087900
GO:0032561	54	236	guanyl ribonucleotide binding	Sapur.001G122500 Sapur.001G127100 Sapur.001G184300 Sapur.001G185700 Sapur.002G013800 Sapur.002G015100 Sapur.002G034900 Sapur.002G049800 Sapur.002G058200 Sapur.002G109900 Sapur.002G112600 Sapur.003G000800 Sapur.003G028100 Sapur.003G050300 Sapur.003G054600 Sapur.004G061100 Sapur.004G115200 Sapur.005G006600 Sapur.005G048100 Sapur.005G058600 Sapur.006G000900 Sapur.006G140000 Sapur.006G150200 Sapur.007G094400 Sapur.008G025400 Sapur.008G078900 Sapur.009G020400 Sapur.009G068500 Sapur.009G090900 Sapur.009G107200 Sapur.009G124500 Sapur.010G069300 Sapur.010G110900 Sapur.010G118800 Sapur.010G183900 Sapur.011G042000 Sapur.011G042200 Sapur.011G100700 Sapur.012G057100 Sapur.012G086800 Sapur.012G088300 Sapur.013G009200 Sapur.013G009300 Sapur.013G084200 Sapur.013G112200 Sapur.013G112400 Sapur.014G039100 Sapur.016G174600 Sapur.016G277800 Sapur.018G009700 Sapur.018G072500 Sapur.018G081000 Sapur.019G087800 Sapur.019G087900
GO:0016758	69	324	hexosyltransferase activity	Sapur.001G043500 Sapur.001G112600 Sapur.001G185400 Sapur.002G004300 Sapur.002G072000 Sapur.002G086200 Sapur.002G176800 Sapur.003G010200 Sapur.003G063400 Sapur.003G099600 Sapur.004G103700 Sapur.004G155200 Sapur.005G125100 Sapur.005G204000 Sapur.006G008700 Sapur.006G062000 Sapur.006G107800 Sapur.006G192900 Sapur.006G198700 Sapur.006G212700 Sapur.006G217000 Sapur.007G044700 Sapur.008G003400 Sapur.008G011900 Sapur.008G028500 Sapur.008G070800 Sapur.008G103100 Sapur.010G087400 Sapur.010G092800 Sapur.010G118100 Sapur.010G129800 Sapur.010G178800 Sapur.011G008300 Sapur.011G112000 Sapur.012G052600 Sapur.013G068300 Sapur.014G018800 Sapur.014G125100 Sapur.014G136300 Sapur.016G010900 Sapur.016G082300 Sapur.016G113700 Sapur.016G221000 Sapur.016G252400 Sapur.016G290200 Sapur.017G062700 Sapur.018G110200 Sapur.019G099900 Sapur.15ZG018200 Sapur.001G026000 Sapur.002G146100 Sapur.004G095900 Sapur.006G145900 Sapur.009G033500 Sapur.009G079300 Sapur.014G040300 Sapur.014G074600 Sapur.014G105300 Sapur.016G020000

				Sapur.016G020300 Sapur.017G040100 Sapur.018G007400 Sapur.019G079900 Sapur.15ZG020900 Sapur.006G054000 Sapur.008G149700 Sapur.010G023600 Sapur.018G062800 Sapur.15ZG021300
GO:0015399	28	102	primary active transmembrane transporter activity	Sapur.001G089300 Sapur.001G144500 Sapur.002G054000 Sapur.003G054000 Sapur.003G061600 Sapur.003G086600 Sapur.003G120000 Sapur.004G021600 Sapur.005G150200 Sapur.005G150300 Sapur.005G172700 Sapur.006G102100 Sapur.007G006700 Sapur.008G114600 Sapur.010G202000 Sapur.013G047900 Sapur.013G064900 Sapur.013G142400 Sapur.014G089900 Sapur.014G104400 Sapur.016G226600 Sapur.017G067400 Sapur.018G075900 Sapur.018G083400 Sapur.15WG078100 Sapur.T031900 Sapur.T047800 Sapur.008G142900
GO:0033218	10	21	amide binding	Sapur.002G016500 Sapur.003G069400 Sapur.004G126600 Sapur.005G020400 Sapur.009G102500 Sapur.012G011100 Sapur.013G019000 Sapur.016G192300 Sapur.017G044400 Sapur.017G044500
GO:0004478	5	6	methionine adenosyltransferase activity	Sapur.002G168400 Sapur.006G101400 Sapur.008G078200 Sapur.010G119600 Sapur.014G091500
GO:0004807	5	6	triose-phosphate isomerase activity	Sapur.004G127000 Sapur.008G044000 Sapur.009G103000 Sapur.010G159800 Sapur.016G133000
GO:0022857	105	556	transmembrane transporter activity	Sapur.002G110800 Sapur.010G090500 Sapur.016G242000 Sapur.T001500 Sapur.001G089300 Sapur.001G144500 Sapur.001G185600 Sapur.002G038200 Sapur.002G054000 Sapur.003G005100 Sapur.003G029600 Sapur.003G054000 Sapur.003G061600 Sapur.003G086600 Sapur.003G120000 Sapur.003G149100 Sapur.004G021600 Sapur.004G025800 Sapur.004G168900 Sapur.005G031200 Sapur.005G150200 Sapur.005G150300 Sapur.005G172700 Sapur.005G204400 Sapur.006G049300 Sapur.006G084000 Sapur.006G102100 Sapur.006G186200 Sapur.007G006700 Sapur.008G005900 Sapur.008G105500 Sapur.008G114600 Sapur.008G126700 Sapur.009G036200 Sapur.009G047600 Sapur.009G090000 Sapur.010G007300 Sapur.010G048300 Sapur.010G202000 Sapur.012G040200 Sapur.013G047900 Sapur.013G064900 Sapur.013G105800 Sapur.013G142400 Sapur.014G089900 Sapur.014G104400 Sapur.014G113300 Sapur.016G047900 Sapur.016G096400 Sapur.016G226600 Sapur.016G310000 Sapur.017G067400 Sapur.018G075900 Sapur.018G083400 Sapur.018G097400 Sapur.018G101700 Sapur.019G082100 Sapur.15WG078100 Sapur.T031900 Sapur.T047800 Sapur.001G033600 Sapur.002G020100 Sapur.002G021300 Sapur.002G066000 Sapur.003G138600 Sapur.004G168100 Sapur.005G186900 Sapur.005G188200 Sapur.006G116300 Sapur.006G180200 Sapur.006G205800 Sapur.007G012200 Sapur.008G113200 Sapur.008G142900 Sapur.009G006300 Sapur.009G062300 Sapur.009G099300 Sapur.010G173300 Sapur.012G025200 Sapur.014G092800 Sapur.016G197500 Sapur.016G231500 Sapur.016G231600 Sapur.017G051500 Sapur.017G056200 Sapur.017G065500 Sapur.017G073700 Sapur.018G009200 Sapur.018G026100 Sapur.018G041100 Sapur.T003500 Sapur.T012400 Sapur.003G108000 Sapur.007G049600 Sapur.15ZG006400 Sapur.008G162700 Sapur.010G133600 Sapur.013G090600 Sapur.014G083800 Sapur.016G040900 Sapur.017G095100 Sapur.003G100300 Sapur.005G058800 Sapur.016G098500 Sapur.017G027100

GO:0004672	259	1565	protein kinase activity	Sapur.001G015800 Sapur.001G033500 Sapur.001G042400 Sapur.001G043100 Sapur.001G066800 Sapur.001G078000 Sapur.001G086800 Sapur.001G093700 Sapur.001G103900 Sapur.001G137500 Sapur.001G148700 Sapur.001G171200 Sapur.001G175900 Sapur.001G190300 Sapur.001G191000 Sapur.002G010500 Sapur.002G028000 Sapur.002G046900 Sapur.002G055700 Sapur.002G106900 Sapur.002G120400 Sapur.002G164000 Sapur.002G176000 Sapur.002G180800 Sapur.002G188100 Sapur.002G192000 Sapur.002G198100 Sapur.002G199800 Sapur.002G201200 Sapur.002G201300 Sapur.003G013500 Sapur.003G037300 Sapur.003G042700 Sapur.003G051100 Sapur.003G052500 Sapur.003G061800 Sapur.003G067600 Sapur.003G073200 Sapur.003G075500 Sapur.003G082200 Sapur.003G085300 Sapur.003G089700 Sapur.003G095700 Sapur.003G099700 Sapur.003G131900 Sapur.003G133000 Sapur.003G138700 Sapur.004G009000 Sapur.004G017100 Sapur.004G023900 Sapur.004G029200 Sapur.004G032900 Sapur.004G033000 Sapur.004G044400 Sapur.004G046500 Sapur.004G057300 Sapur.004G061000 Sapur.004G067800 Sapur.004G068600 Sapur.004G080700 Sapur.004G087900 Sapur.004G115800 Sapur.004G135100 Sapur.004G170800 Sapur.004G172200 Sapur.004G177200 Sapur.005G001800 Sapur.005G011700 Sapur.005G031100 Sapur.005G039800 Sapur.005G050800 Sapur.005G057200 Sapur.005G065000 Sapur.005G065300 Sapur.005G081600 Sapur.005G109200 Sapur.005G143900 Sapur.005G148400 Sapur.005G160000 Sapur.005G202000 Sapur.005G203500 Sapur.005G205600 Sapur.005G208100 Sapur.006G039400 Sapur.006G043700 Sapur.006G044000 Sapur.006G059800 Sapur.006G063800 Sapur.006G064600 Sapur.006G077000 Sapur.006G090100 Sapur.006G093700 Sapur.006G096300 Sapur.006G107000 Sapur.006G117000 Sapur.006G151400 Sapur.006G185100 Sapur.006G195000 Sapur.006G196200 Sapur.006G210500 Sapur.006G215000 Sapur.006G217500 Sapur.007G003100 Sapur.007G012300 Sapur.007G037800 Sapur.007G044000 Sapur.007G054000 Sapur.007G077400 Sapur.007G085900 Sapur.007G124000 Sapur.007G124100 Sapur.008G008800 Sapur.008G016100 Sapur.008G054200 Sapur.008G054500 Sapur.008G076100 Sapur.008G089200 Sapur.008G107400 Sapur.008G111800 Sapur.008G113700 Sapur.008G116800 Sapur.008G136200 Sapur.008G136400 Sapur.008G143200 Sapur.008G165900 Sapur.009G030800 Sapur.009G058900 Sapur.009G059300 Sapur.009G091500 Sapur.009G092000 Sapur.009G107300 Sapur.010G000800 Sapur.010G028600 Sapur.010G041500 Sapur.010G052000 Sapur.010G064500 Sapur.010G070900 Sapur.010G075700 Sapur.010G076000 Sapur.010G076900 Sapur.010G127100 Sapur.010G145100 Sapur.010G147600 Sapur.010G147800 Sapur.010G169400 Sapur.010G189000 Sapur.011G021000 Sapur.011G030700 Sapur.011G036900 Sapur.011G040500 Sapur.011G040600 Sapur.011G046900 Sapur.011G047800 Sapur.011G050900 Sapur.011G080000 Sapur.011G080600 Sapur.011G089900 Sapur.011G095700 Sapur.011G097200 Sapur.011G101300 Sapur.011G108300 Sapur.011G121500 Sapur.012G020400 Sapur.012G029700 Sapur.012G032600 Sapur.012G041400 Sapur.012G048500 Sapur.012G051500 Sapur.012G052000 Sapur.012G063200 Sapur.012G064400 Sapur.012G064900 Sapur.012G093400 Sapur.012G097700 Sapur.012G106800 Sapur.013G003400 Sapur.013G034500 Sapur.013G055800 Sapur.013G108700 Sapur.013G141900 Sapur.014G014200 Sapur.014G017800 Sapur.014G050200 Sapur.014G054500 Sapur.014G065600 Sapur.014G067100 Sapur.014G067400 Sapur.014G069000 Sapur.014G084200 Sapur.014G091100 Sapur.014G095200 Sapur.014G110500 Sapur.014G119600 Sapur.014G126200 Sapur.016G045300 Sapur.016G046200 Sapur.016G048600

				Sapur.016G049500 Sapur.016G064900 Sapur.016G090700 Sapur.016G113500 Sapur.016G115900 Sapur.016G122900 Sapur.016G133200 Sapur.016G164800 Sapur.016G165400 Sapur.016G222400 Sapur.016G256600 Sapur.016G263500 Sapur.016G267500 Sapur.016G267900 Sapur.016G269800 Sapur.016G272100 Sapur.016G274100 Sapur.016G293800 Sapur.016G294500 Sapur.016G304300 Sapur.017G001800 Sapur.017G002000 Sapur.017G019000 Sapur.017G072700 Sapur.017G096900 Sapur.017G097600 Sapur.017G107900 Sapur.017G111400 Sapur.017G114100 Sapur.018G018700 Sapur.018G035500 Sapur.018G048200 Sapur.018G053200 Sapur.018G058300 Sapur.018G060300 Sapur.018G067300 Sapur.018G072700 Sapur.018G082400 Sapur.018G086200 Sapur.018G107900 Sapur.018G114200 Sapur.019G008900 Sapur.019G061700 Sapur.019G062400 Sapur.019G083400 Sapur.019G099500 Sapur.019G113000 Sapur.15WG028200 Sapur.15WG031300 Sapur.15WG066000 Sapur.15WG079300 Sapur.15ZG018000 Sapur.15ZG027900 Sapur.15ZG035300 Sapur.15ZG054200 Sapur.15ZG082200 Sapur.15ZG085600 Sapur.15ZG093000 Sapur.15ZG124400 Sapur.15ZG134100 Sapur.T044700 Sapur.T051500
GO:0016773	275	1675	phosphotransferase activity, alcohol group as acceptor	Sapur.001G015800 Sapur.001G033500 Sapur.001G042400 Sapur.001G043100 Sapur.001G066800 Sapur.001G078000 Sapur.001G086800 Sapur.001G093700 Sapur.001G103900 Sapur.001G137500 Sapur.001G148700 Sapur.001G171200 Sapur.001G175900 Sapur.001G190300 Sapur.001G191000 Sapur.002G010500 Sapur.002G028000 Sapur.002G046900 Sapur.002G055700 Sapur.002G106900 Sapur.002G120400 Sapur.002G164000 Sapur.002G176000 Sapur.002G180800 Sapur.002G188100 Sapur.002G192000 Sapur.002G198100 Sapur.002G199800 Sapur.002G201200 Sapur.002G201300 Sapur.003G013500 Sapur.003G037300 Sapur.003G042700 Sapur.003G051100 Sapur.003G052500 Sapur.003G061800 Sapur.003G067600 Sapur.003G073200 Sapur.003G075500 Sapur.003G082200 Sapur.003G085300 Sapur.003G089700 Sapur.003G095700 Sapur.003G099700 Sapur.003G131900 Sapur.003G133000 Sapur.003G138700 Sapur.004G009000 Sapur.004G017100 Sapur.004G023900 Sapur.004G029200 Sapur.004G032900 Sapur.004G033000 Sapur.004G044400 Sapur.004G046500 Sapur.004G057300 Sapur.004G061000 Sapur.004G067800 Sapur.004G068600 Sapur.004G080700 Sapur.004G087900 Sapur.004G115800 Sapur.004G135100 Sapur.004G170800 Sapur.004G172200 Sapur.004G177200 Sapur.005G001800 Sapur.005G011700 Sapur.005G031100 Sapur.005G039800 Sapur.005G050800 Sapur.005G057200 Sapur.005G065000 Sapur.005G065300 Sapur.005G081600 Sapur.005G109200 Sapur.005G143900 Sapur.005G148400 Sapur.005G160000 Sapur.005G202000 Sapur.005G203500 Sapur.005G205600 Sapur.005G208100 Sapur.006G039400 Sapur.006G043700 Sapur.006G044000 Sapur.006G059800 Sapur.006G063800 Sapur.006G064600 Sapur.006G077000 Sapur.006G090100 Sapur.006G093700 Sapur.006G096300 Sapur.006G107000 Sapur.006G117000 Sapur.006G151400 Sapur.006G185100 Sapur.006G195000 Sapur.006G196200 Sapur.006G210500 Sapur.006G215000 Sapur.006G217500 Sapur.007G003100 Sapur.007G012300 Sapur.007G037800 Sapur.007G044000 Sapur.007G054000 Sapur.007G077400 Sapur.007G085900 Sapur.007G124000 Sapur.007G124100 Sapur.008G008800 Sapur.008G016100 Sapur.008G054200 Sapur.008G054500 Sapur.008G076100 Sapur.008G089200 Sapur.008G107400 Sapur.008G111800 Sapur.008G113700 Sapur.008G116800 Sapur.008G136200 Sapur.008G136400 Sapur.008G143200 Sapur.008G165900 Sapur.009G030800 Sapur.009G058900 Sapur.009G059300 Sapur.009G091500 Sapur.009G092000 Sapur.009G107300

				Sapur.010G000800 Sapur.010G028600 Sapur.010G041500 Sapur.010G052000 Sapur.010G064500 Sapur.010G070900 Sapur.010G075700 Sapur.010G076000 Sapur.010G076900 Sapur.010G127100 Sapur.010G145100 Sapur.010G147600 Sapur.010G147800 Sapur.010G169400 Sapur.010G189000 Sapur.011G021000 Sapur.011G030700 Sapur.011G036900 Sapur.011G040500 Sapur.011G040600 Sapur.011G046900 Sapur.011G047800 Sapur.011G050900 Sapur.011G080000 Sapur.011G080600 Sapur.011G089900 Sapur.011G095700 Sapur.011G097200 Sapur.011G101300 Sapur.011G108300 Sapur.011G121500 Sapur.012G020400 Sapur.012G029700 Sapur.012G032600 Sapur.012G041400 Sapur.012G048500 Sapur.012G051500 Sapur.012G052000 Sapur.012G063200 Sapur.012G064400 Sapur.012G064900 Sapur.012G093400 Sapur.012G097700 Sapur.012G106800 Sapur.013G003400 Sapur.013G034500 Sapur.013G055800 Sapur.013G108700 Sapur.013G141900 Sapur.014G014200 Sapur.014G017800 Sapur.014G050200 Sapur.014G054500 Sapur.014G065600 Sapur.014G067100 Sapur.014G067400 Sapur.014G069000 Sapur.014G084200 Sapur.014G091100 Sapur.014G095200 Sapur.014G110500 Sapur.014G119600 Sapur.014G126200 Sapur.016G045300 Sapur.016G046200 Sapur.016G048600 Sapur.016G049500 Sapur.016G064900 Sapur.016G090700 Sapur.016G113500 Sapur.016G115900 Sapur.016G122900 Sapur.016G133200 Sapur.016G164800 Sapur.016G165400 Sapur.016G222400 Sapur.016G256600 Sapur.016G263500 Sapur.016G267500 Sapur.016G267900 Sapur.016G269800 Sapur.016G272100 Sapur.016G274100 Sapur.016G293800 Sapur.016G294500 Sapur.016G304300 Sapur.017G001800 Sapur.017G002000 Sapur.017G019000 Sapur.017G072700 Sapur.017G096900 Sapur.017G097600 Sapur.017G107900 Sapur.017G111400 Sapur.017G114100 Sapur.018G018700 Sapur.018G035500 Sapur.018G048200 Sapur.018G053200 Sapur.018G058300 Sapur.018G060300 Sapur.018G067300 Sapur.018G072700 Sapur.018G082400 Sapur.018G086200 Sapur.018G107900 Sapur.018G114200 Sapur.019G008900 Sapur.019G061700 Sapur.019G062400 Sapur.019G083400 Sapur.019G099500 Sapur.019G113000 Sapur.15WG028200 Sapur.15WG031300 Sapur.15WG066000 Sapur.15WG079300 Sapur.15ZG018000 Sapur.15ZG027900 Sapur.15ZG035300 Sapur.15ZG054200 Sapur.15ZG082200 Sapur.15ZG085600 Sapur.15ZG093000 Sapur.15ZG124400 Sapur.15ZG134100 Sapur.T044700 Sapur.T051500 Sapur.001G161400 Sapur.002G001800 Sapur.002G047800 Sapur.004G114100 Sapur.004G114400 Sapur.005G206700 Sapur.006G126300 Sapur.006G194700 Sapur.010G199900 Sapur.011G009700 Sapur.012G083700 Sapur.013G024200 Sapur.013G132500 Sapur.014G077500 Sapur.016G244800 Sapur.016G266500
GO:0003993	7	12	acid phosphatase activity	Sapur.001G191100 Sapur.002G200900 Sapur.004G173300 Sapur.006G126600 Sapur.008G057700 Sapur.010G144300 Sapur.010G151600
GO:0046923	6	9	ER retention sequence binding	Sapur.002G016500 Sapur.004G126600 Sapur.009G102500 Sapur.016G192300 Sapur.017G044400 Sapur.017G044500
GO:0016301	273	1669	kinase activity	Sapur.001G015800 Sapur.001G033500 Sapur.001G042400 Sapur.001G043100 Sapur.001G066800 Sapur.001G078000 Sapur.001G086800 Sapur.001G093700 Sapur.001G103900 Sapur.001G137500 Sapur.001G148700 Sapur.001G171200 Sapur.001G175900 Sapur.001G190300 Sapur.001G191000 Sapur.002G010500 Sapur.002G028000 Sapur.002G046900 Sapur.002G055700 Sapur.002G106900 Sapur.002G120400 Sapur.002G164000 Sapur.002G176000 Sapur.002G180800 Sapur.002G188100 Sapur.002G192000 Sapur.002G198100 Sapur.002G199800 Sapur.002G201200

Sapur.002G201300	Sapur.003G013500	Sapur.003G037300	Sapur.003G042700	Sapur.003G051100	Sapur.003G052500
Sapur.003G061800	Sapur.003G067600	Sapur.003G073200	Sapur.003G075500	Sapur.003G082200	Sapur.003G085300
Sapur.003G089700	Sapur.003G095700	Sapur.003G099700	Sapur.003G131900	Sapur.003G133000	Sapur.003G138700
Sapur.004G009000	Sapur.004G017100	Sapur.004G023900	Sapur.004G029200	Sapur.004G032900	Sapur.004G033000
Sapur.004G044400	Sapur.004G046500	Sapur.004G057300	Sapur.004G061000	Sapur.004G067800	Sapur.004G068600
Sapur.004G080700	Sapur.004G087900	Sapur.004G115800	Sapur.004G135100	Sapur.004G170800	Sapur.004G172200
Sapur.004G177200	Sapur.005G001800	Sapur.005G011700	Sapur.005G031100	Sapur.005G039800	Sapur.005G050800
Sapur.005G057200	Sapur.005G065000	Sapur.005G065300	Sapur.005G081600	Sapur.005G109200	Sapur.005G143900
Sapur.005G148400	Sapur.005G160000	Sapur.005G202000	Sapur.005G203500	Sapur.005G205600	Sapur.005G208100
Sapur.006G039400	Sapur.006G043700	Sapur.006G044000	Sapur.006G059800	Sapur.006G063800	Sapur.006G064600
Sapur.006G077000	Sapur.006G090100	Sapur.006G093700	Sapur.006G096300	Sapur.006G107000	Sapur.006G117000
Sapur.006G151400	Sapur.006G185100	Sapur.006G195000	Sapur.006G196200	Sapur.006G210500	Sapur.006G215000
Sapur.006G217500	Sapur.007G003100	Sapur.007G012300	Sapur.007G037800	Sapur.007G044000	Sapur.007G054000
Sapur.007G077400	Sapur.007G085900	Sapur.007G124000	Sapur.007G124100	Sapur.008G008800	Sapur.008G016100
Sapur.008G054200	Sapur.008G054500	Sapur.008G076100	Sapur.008G089200	Sapur.008G107400	Sapur.008G111800
Sapur.008G113700	Sapur.008G116800	Sapur.008G136200	Sapur.008G136400	Sapur.008G143200	Sapur.008G165900
Sapur.009G030800	Sapur.009G058900	Sapur.009G059300	Sapur.009G091500	Sapur.009G092000	Sapur.009G107300
Sapur.010G000800	Sapur.010G028600	Sapur.010G041500	Sapur.010G052000	Sapur.010G064500	Sapur.010G070900
Sapur.010G075700	Sapur.010G076000	Sapur.010G076900	Sapur.010G127100	Sapur.010G145100	Sapur.010G147600
Sapur.010G147800	Sapur.010G169400	Sapur.010G189000	Sapur.011G021000	Sapur.011G030700	Sapur.011G036900
Sapur.011G040500	Sapur.011G040600	Sapur.011G046900	Sapur.011G047800	Sapur.011G050900	Sapur.011G080000
Sapur.011G080600	Sapur.011G089900	Sapur.011G095700	Sapur.011G097200	Sapur.011G101300	Sapur.011G108300
Sapur.011G121500	Sapur.012G020400	Sapur.012G029700	Sapur.012G032600	Sapur.012G041400	Sapur.012G048500
Sapur.012G051500	Sapur.012G052000	Sapur.012G063200	Sapur.012G064400	Sapur.012G064900	Sapur.012G071500
Sapur.012G093400	Sapur.012G097700	Sapur.012G106800	Sapur.013G003400	Sapur.013G034500	Sapur.013G055800
Sapur.013G108700	Sapur.013G141900	Sapur.014G014200	Sapur.014G017800	Sapur.014G050200	Sapur.014G054500
Sapur.014G065600	Sapur.014G067100	Sapur.014G067400	Sapur.014G069000	Sapur.014G084200	Sapur.014G091100
Sapur.014G095200	Sapur.014G110500	Sapur.014G119600	Sapur.014G126200	Sapur.016G045300	Sapur.016G046200
Sapur.016G048600	Sapur.016G049500	Sapur.016G064900	Sapur.016G090700	Sapur.016G113500	Sapur.016G115900
Sapur.016G122900	Sapur.016G133200	Sapur.016G164800	Sapur.016G165400	Sapur.016G222400	Sapur.016G256600
Sapur.016G263500	Sapur.016G267500	Sapur.016G267900	Sapur.016G269800	Sapur.016G272100	Sapur.016G274100
Sapur.016G293800	Sapur.016G294500	Sapur.016G304300	Sapur.017G001800	Sapur.017G002000	Sapur.017G019000
Sapur.017G072700	Sapur.017G096900	Sapur.017G097600	Sapur.017G107900	Sapur.017G111400	Sapur.017G114100

				Sapur.018G018700 Sapur.018G035500 Sapur.018G048200 Sapur.018G053200 Sapur.018G058300 Sapur.018G060300 Sapur.018G067300 Sapur.018G072700 Sapur.018G082400 Sapur.018G086200 Sapur.018G107900 Sapur.018G114200 Sapur.019G008900 Sapur.019G061700 Sapur.019G062400 Sapur.019G083400 Sapur.019G099500 Sapur.019G113000 Sapur.15WG028200 Sapur.15WG031300 Sapur.15WG066000 Sapur.15WG079300 Sapur.15ZG018000 Sapur.15ZG027900 Sapur.15ZG035300 Sapur.15ZG054200 Sapur.15ZG082200 Sapur.15ZG085600 Sapur.15ZG093000 Sapur.15ZG124400 Sapur.15ZG134100 Sapur.T044700 Sapur.T051500 Sapur.002G001800 Sapur.003G038600 Sapur.005G173800 Sapur.005G206700 Sapur.006G126300 Sapur.006G194700 Sapur.011G009700 Sapur.011G122000 Sapur.012G083700 Sapur.013G024200 Sapur.013G132500 Sapur.016G266500 Sapur.001G161400
GO:0008238	18	58	exopeptidase activity	Sapur.001G051400 Sapur.002G056400 Sapur.004G162500 Sapur.005G072700 Sapur.005G147300 Sapur.006G026100 Sapur.008G024600 Sapur.008G031500 Sapur.009G001100 Sapur.009G042700 Sapur.010G115800 Sapur.012G080500 Sapur.016G030700 Sapur.018G078800 Sapur.019G052800 Sapur.15ZG102400 Sapur.T045900 Sapur.002G009400
GO:0042277	6	10	peptide binding	Sapur.002G016500 Sapur.004G126600 Sapur.009G102500 Sapur.016G192300 Sapur.017G044400 Sapur.017G044500
GO:0005048	6	10	signal sequence binding	Sapur.002G016500 Sapur.004G126600 Sapur.009G102500 Sapur.016G192300 Sapur.017G044400 Sapur.017G044500
GO:0008092	37	163	cytoskeletal protein binding	Sapur.001G000500 Sapur.001G044900 Sapur.002G122000 Sapur.002G159800 Sapur.002G182100 Sapur.003G028400 Sapur.003G130500 Sapur.003G169700 Sapur.004G120900 Sapur.004G122400 Sapur.004G130100 Sapur.005G015200 Sapur.006G028600 Sapur.006G038100 Sapur.006G135400 Sapur.006G159000 Sapur.007G012500 Sapur.007G043900 Sapur.008G109800 Sapur.008G113100 Sapur.009G018700 Sapur.009G033900 Sapur.009G097000 Sapur.010G008800 Sapur.010G164300 Sapur.010G206600 Sapur.011G097800 Sapur.011G108500 Sapur.013G010400 Sapur.014G014400 Sapur.016G056900 Sapur.016G077400 Sapur.016G127100 Sapur.016G129900 Sapur.016G130000 Sapur.016G275100 Sapur.T108200
GO:0003774	29	121	cytoskeletal motor activity	Sapur.001G000500 Sapur.002G122000 Sapur.002G159800 Sapur.002G182100 Sapur.003G028400 Sapur.003G169700 Sapur.004G120900 Sapur.004G122400 Sapur.005G015200 Sapur.006G159000 Sapur.007G012500 Sapur.007G043900 Sapur.009G018700 Sapur.009G097000 Sapur.010G008800 Sapur.011G097800 Sapur.011G108500 Sapur.013G010400 Sapur.014G014400 Sapur.016G054000 Sapur.016G056900 Sapur.016G077400 Sapur.016G127100 Sapur.016G275100 Sapur.005G070300 Sapur.006G120600 Sapur.011G091100 Sapur.012G020300 Sapur.016G265600
GO:0005402	4	5	carbohydrate:monoatomic cation symporter activity	Sapur.003G100300 Sapur.005G058800 Sapur.016G098500 Sapur.017G027100
GO:001	4	5	carbohydrate	Sapur.003G100300 Sapur.005G058800 Sapur.016G098500 Sapur.017G027100

5144			transmembrane transporter activity	
GO:0015293	4	5	symporter activity	Sapur.003G100300 Sapur.005G058800 Sapur.016G098500 Sapur.017G027100
GO:0015294	4	5	solute:monoatomic cation symporter activity	Sapur.003G100300 Sapur.005G058800 Sapur.016G098500 Sapur.017G027100
GO:0015295	4	5	solute:proton symporter activity	Sapur.003G100300 Sapur.005G058800 Sapur.016G098500 Sapur.017G027100
GO:0005351	4	5	carbohydrate:proton symporter activity	Sapur.003G100300 Sapur.005G058800 Sapur.016G098500 Sapur.017G027100
GO:0008121	5	8	ubiquinol-cytochrome-c reductase activity	Sapur.002G054000 Sapur.003G120000 Sapur.005G150200 Sapur.005G150300 Sapur.013G142400
GO:0015924	5	8	mannosyl-oligosaccharide mannosidase activity	Sapur.004G102500 Sapur.008G004000 Sapur.010G203100 Sapur.018G108000 Sapur.15ZG072800
GO:0004571	5	8	mannosyl-oligosaccharide 1,2-alpha-mannosidase activity	Sapur.004G102500 Sapur.008G004000 Sapur.010G203100 Sapur.018G108000 Sapur.15ZG072800
GO:0008017	29	123	microtubule binding	Sapur.001G000500 Sapur.001G044900 Sapur.002G122000 Sapur.002G159800 Sapur.002G182100 Sapur.003G028400 Sapur.003G130500 Sapur.003G169700 Sapur.004G120900 Sapur.004G122400 Sapur.005G015200 Sapur.006G028600 Sapur.006G159000 Sapur.007G012500 Sapur.007G043900 Sapur.008G109800 Sapur.008G113100 Sapur.009G018700 Sapur.009G097000 Sapur.010G008800 Sapur.010G206600 Sapur.011G097800 Sapur.011G108500 Sapur.013G010400 Sapur.014G014400 Sapur.016G056900 Sapur.016G077400 Sapur.016G127100 Sapur.016G275100
GO:0015631	29	124	tubulin binding	Sapur.001G000500 Sapur.001G044900 Sapur.002G122000 Sapur.002G159800 Sapur.002G182100 Sapur.003G028400 Sapur.003G130500 Sapur.003G169700 Sapur.004G120900 Sapur.004G122400 Sapur.005G015200

				Sapur.006G028600 Sapur.006G159000 Sapur.007G012500 Sapur.007G043900 Sapur.008G109800 Sapur.008G113100 Sapur.009G018700 Sapur.009G097000 Sapur.010G008800 Sapur.010G206600 Sapur.011G097800 Sapur.011G108500 Sapur.013G010400 Sapur.014G014400 Sapur.016G056900 Sapur.016G077400 Sapur.016G127100 Sapur.016G275100
GO:0004623	3	3	phospholipase A2 activity	Sapur.006G056800 Sapur.006G123700 Sapur.018G103200
GO:0008158	3	3	hedgehog receptor activity	Sapur.002G006100 Sapur.005G201800 Sapur.009G130600
GO:0015095	6	12	magnesium ion transmembrane transporter activity	Sapur.003G005100 Sapur.006G186200 Sapur.009G036200 Sapur.013G105800 Sapur.014G113300 Sapur.019G082100
GO:0140103	4	6	catalytic activity, acting on a glycoprotein	Sapur.014G136300 Sapur.006G054000 Sapur.008G149700 Sapur.010G023600
GO:0015103	12	40	inorganic anion transmembrane transporter activity	Sapur.001G185600 Sapur.002G038200 Sapur.003G029600 Sapur.008G105500 Sapur.008G126700 Sapur.016G310000 Sapur.018G097400 Sapur.014G092800 Sapur.018G026100 Sapur.003G108000 Sapur.007G049600 Sapur.15ZG006400
GO:0022804	41	204	active transmembrane transporter activity	Sapur.001G089300 Sapur.001G144500 Sapur.002G054000 Sapur.003G054000 Sapur.003G061600 Sapur.003G086600 Sapur.003G120000 Sapur.004G021600 Sapur.004G168900 Sapur.005G150200 Sapur.005G150300 Sapur.005G172700 Sapur.006G102100 Sapur.007G006700 Sapur.008G114600 Sapur.010G007300 Sapur.010G202000 Sapur.013G047900 Sapur.013G064900 Sapur.013G142400 Sapur.014G089900 Sapur.014G104400 Sapur.016G047900 Sapur.016G226600 Sapur.017G067400 Sapur.018G075900 Sapur.018G083400 Sapur.15WG078100 Sapur.T031900 Sapur.T047800 Sapur.002G038200 Sapur.003G029600 Sapur.008G105500 Sapur.008G126700 Sapur.008G142900 Sapur.014G092800 Sapur.018G026100 Sapur.003G100300 Sapur.005G058800 Sapur.016G098500 Sapur.017G027100
GO:0003872	5	10	6-phosphofructokinase activity	Sapur.002G001800 Sapur.005G206700 Sapur.006G126300 Sapur.006G194700 Sapur.011G009700
GO:0016679	5	10	oxidoreductase activity, acting on diphenols and related substances as donors	Sapur.002G054000 Sapur.003G120000 Sapur.005G150200 Sapur.005G150300 Sapur.013G142400

GO:0003777	24	106	microtubule motor activity	Sapur.001G000500 Sapur.002G122000 Sapur.002G159800 Sapur.002G182100 Sapur.003G028400 Sapur.003G169700 Sapur.004G120900 Sapur.004G122400 Sapur.005G015200 Sapur.006G159000 Sapur.007G012500 Sapur.007G043900 Sapur.009G018700 Sapur.009G097000 Sapur.010G008800 Sapur.011G097800 Sapur.011G108500 Sapur.013G010400 Sapur.014G014400 Sapur.016G054000 Sapur.016G056900 Sapur.016G077400 Sapur.016G127100 Sapur.016G275100
GO:0016462	52	278	pyrophosphatase activity	Sapur.001G086500 Sapur.001G185700 Sapur.002G015900 Sapur.002G028100 Sapur.002G034900 Sapur.002G090500 Sapur.002G146800 Sapur.003G000800 Sapur.003G061600 Sapur.003G090100 Sapur.003G091100 Sapur.003G166900 Sapur.004G021600 Sapur.005G047400 Sapur.005G180900 Sapur.005G192400 Sapur.006G025400 Sapur.006G037500 Sapur.006G102100 Sapur.006G150200 Sapur.006G180500 Sapur.009G052100 Sapur.009G052200 Sapur.009G066800 Sapur.010G069300 Sapur.010G180200 Sapur.011G119600 Sapur.012G018300 Sapur.013G016100 Sapur.014G089900 Sapur.014G104400 Sapur.016G030100 Sapur.016G094800 Sapur.016G158100 Sapur.016G158200 Sapur.016G172500 Sapur.016G226600 Sapur.017G067400 Sapur.018G053400 Sapur.018G081000 Sapur.019G002100 Sapur.019G002700 Sapur.019G035200 Sapur.15WG078100 Sapur.15ZG004600 Sapur.T047800 Sapur.T114400 Sapur.002G137300 Sapur.006G050300 Sapur.006G067100 Sapur.010G205600 Sapur.018G096200
GO:0016614	27	127	oxidoreductase activity, acting on CH-OH group of donors	Sapur.001G100800 Sapur.002G002500 Sapur.002G103600 Sapur.002G119700 Sapur.002G157000 Sapur.003G079300 Sapur.004G052600 Sapur.004G071000 Sapur.004G077000 Sapur.006G175000 Sapur.008G041000 Sapur.008G074200 Sapur.010G041100 Sapur.010G123100 Sapur.010G163100 Sapur.014G049800 Sapur.014G103300 Sapur.016G074400 Sapur.016G130500 Sapur.016G180500 Sapur.016G240600 Sapur.016G246100 Sapur.017G049800 Sapur.017G075600 Sapur.017G083200 Sapur.017G124600 Sapur.006G195400
GO:0016616	27	127	oxidoreductase activity, acting on the CH-OH group of donors, NAD or NADP as acceptor	Sapur.001G100800 Sapur.002G002500 Sapur.002G103600 Sapur.002G119700 Sapur.002G157000 Sapur.003G079300 Sapur.004G052600 Sapur.004G071000 Sapur.004G077000 Sapur.006G175000 Sapur.008G041000 Sapur.008G074200 Sapur.010G041100 Sapur.010G123100 Sapur.010G163100 Sapur.014G049800 Sapur.014G103300 Sapur.016G074400 Sapur.016G130500 Sapur.016G180500 Sapur.016G240600 Sapur.016G246100 Sapur.017G049800 Sapur.017G075600 Sapur.017G083200 Sapur.017G124600 Sapur.006G195400
GO:0016861	7	20	intramolecular oxidoreductase activity, interconverting aldoses and ketoses	Sapur.005G040300 Sapur.013G037300 Sapur.004G127000 Sapur.008G044000 Sapur.009G103000 Sapur.010G159800 Sapur.016G133000
GO:0016675	4	8	oxidoreductase activity, acting on a heme group of	Sapur.001G144500 Sapur.007G006700 Sapur.018G075900 Sapur.018G083400

			donors	
GO:0004129	4	8	cytochrome-c oxidase activity	Sapur.001G144500 Sapur.007G006700 Sapur.018G075900 Sapur.018G083400
GO:0008443	5	12	phosphofructokinase activity	Sapur.002G001800 Sapur.005G206700 Sapur.006G126300 Sapur.006G194700 Sapur.011G009700
GO:0004427	5	12	inorganic diphosphate phosphatase activity	Sapur.002G137300 Sapur.006G050300 Sapur.006G067100 Sapur.010G205600 Sapur.018G096200
GO:0051087	7	21	protein-folding chaperone binding	Sapur.003G086100 Sapur.006G158500 Sapur.009G058100 Sapur.012G102400 Sapur.016G056600 Sapur.016G164200 Sapur.15ZG125600
GO:0046508	2	2	hydrolase activity, acting on carbon-sulfur bonds	Sapur.016G195500 Sapur.017G050100
GO:0004738	2	2	pyruvate dehydrogenase activity	Sapur.001G050000 Sapur.003G123600
GO:0004739	2	2	pyruvate dehydrogenase (acetyl-transferring) activity	Sapur.001G050000 Sapur.003G123600
GO:0004013	2	2	adenosylhomocysteinase activity	Sapur.016G195500 Sapur.017G050100
GO:0004047	2	2	aminomethyltransferase activity	Sapur.004G005600 Sapur.011G005900
GO:0005047	2	2	signal recognition particle binding	Sapur.006G150200 Sapur.018G081000
GO:0016491	243	1583	oxidoreductase activity	Sapur.001G005700 Sapur.001G080400 Sapur.001G095200 Sapur.001G098800 Sapur.001G100800 Sapur.002G022900 Sapur.002G065200 Sapur.002G139100 Sapur.002G179500 Sapur.002G197600 Sapur.003G077900 Sapur.003G115200 Sapur.003G120000 Sapur.003G153700 Sapur.003G156300 Sapur.003G162900 Sapur.004G006100 Sapur.004G017500 Sapur.004G021200 Sapur.004G035300 Sapur.004G047000 Sapur.004G098500 Sapur.004G099400

Sapur.004G135900 Sapur.005G085600 Sapur.005G086800 Sapur.005G101300 Sapur.005G106500 Sapur.005G140600
Sapur.005G140700 Sapur.005G153800 Sapur.005G157000 Sapur.005G185800 Sapur.005G196400 Sapur.005G198500
Sapur.006G030600 Sapur.006G071400 Sapur.006G071900 Sapur.006G079700 Sapur.006G079800 Sapur.006G106900
Sapur.006G110900 Sapur.006G124800 Sapur.006G130400 Sapur.006G137500 Sapur.006G164600 Sapur.007G015200
Sapur.007G020600 Sapur.007G026600 Sapur.007G036000 Sapur.007G037900 Sapur.007G112300 Sapur.007G121300
Sapur.008G050800 Sapur.008G058600 Sapur.008G058700 Sapur.008G067000 Sapur.008G079500 Sapur.008G116700
Sapur.008G172500 Sapur.009G025400 Sapur.009G025500 Sapur.009G059000 Sapur.009G071300 Sapur.009G075100
Sapur.009G078700 Sapur.009G080300 Sapur.009G084400 Sapur.009G106700 Sapur.009G122800 Sapur.010G016400
Sapur.010G041100 Sapur.010G112700 Sapur.010G143300 Sapur.010G143400 Sapur.010G151400 Sapur.011G020400
Sapur.011G042800 Sapur.011G083300 Sapur.011G096100 Sapur.011G118000 Sapur.011G118200 Sapur.011G118300
Sapur.011G119400 Sapur.012G016400 Sapur.012G048700 Sapur.012G064700 Sapur.013G068900 Sapur.013G070000
Sapur.013G098000 Sapur.014G093700 Sapur.016G018300 Sapur.016G032800 Sapur.016G034600 Sapur.016G067200
Sapur.016G094000 Sapur.016G097600 Sapur.016G139000 Sapur.016G165000 Sapur.016G177200 Sapur.016G215200
Sapur.016G259100 Sapur.016G259200 Sapur.016G259300 Sapur.016G272200 Sapur.016G280000 Sapur.016G300700
Sapur.016G300800 Sapur.016G301000 Sapur.017G054200 Sapur.017G060600 Sapur.017G067800 Sapur.017G096500
Sapur.018G032300 Sapur.018G104600 Sapur.019G000600 Sapur.019G000700 Sapur.019G000900 Sapur.15ZG022100
Sapur.15ZG082400 Sapur.15ZG124800 Sapur.T023100 Sapur.T072500 Sapur.T185900 Sapur.001G033400
Sapur.001G050000 Sapur.001G089300 Sapur.001G144500 Sapur.001G173200 Sapur.002G002500 Sapur.002G054000
Sapur.002G057800 Sapur.002G081200 Sapur.002G081300 Sapur.002G103600 Sapur.002G119700 Sapur.002G152400
Sapur.002G157000 Sapur.003G008700 Sapur.003G079300 Sapur.003G086600 Sapur.003G123600 Sapur.004G050300
Sapur.004G052600 Sapur.004G071000 Sapur.004G077000 Sapur.004G152300 Sapur.005G150200 Sapur.005G150300
Sapur.006G175000 Sapur.006G189300 Sapur.006G204700 Sapur.007G003500 Sapur.007G006700 Sapur.007G010500
Sapur.007G091200 Sapur.008G013500 Sapur.008G041000 Sapur.008G074200 Sapur.008G114600 Sapur.008G122100
Sapur.009G108500 Sapur.009G129200 Sapur.010G013800 Sapur.010G059500 Sapur.010G123100 Sapur.010G163100
Sapur.012G067800 Sapur.013G096200 Sapur.013G142400 Sapur.014G049800 Sapur.014G103300 Sapur.016G018000
Sapur.016G041700 Sapur.016G045200 Sapur.016G074400 Sapur.016G130500 Sapur.016G180500 Sapur.016G206000
Sapur.016G240600 Sapur.016G246100 Sapur.017G000100 Sapur.017G049800 Sapur.017G072900 Sapur.017G073000
Sapur.017G075600 Sapur.017G083200 Sapur.017G124600 Sapur.018G075900 Sapur.018G083400 Sapur.019G060800
Sapur.008G066400 Sapur.008G102400 Sapur.010G088200 Sapur.15ZG091400 Sapur.001G053400 Sapur.001G089000
Sapur.001G093200 Sapur.002G025200 Sapur.002G032600 Sapur.002G048700 Sapur.002G099200 Sapur.002G099300
Sapur.002G143800 Sapur.002G180700 Sapur.003G119600 Sapur.005G034700 Sapur.005G093900 Sapur.005G123600
Sapur.006G023400 Sapur.006G063500 Sapur.006G140400 Sapur.006G195400 Sapur.007G001800 Sapur.007G013700
Sapur.007G023900 Sapur.007G047100 Sapur.007G047200 Sapur.007G067000 Sapur.007G075400 Sapur.008G007500

				Sapur.008G043100 Sapur.008G053200 Sapur.009G050100 Sapur.009G050200 Sapur.009G050400 Sapur.009G099500 Sapur.010G021600 Sapur.010G160600 Sapur.011G066900 Sapur.011G067400 Sapur.013G141300 Sapur.013G143000 Sapur.016G028000 Sapur.016G096000 Sapur.016G134900 Sapur.016G156100 Sapur.016G162100 Sapur.016G170800 Sapur.016G204800 Sapur.016G214600 Sapur.018G113700 Sapur.019G112500
GO:0016741	44	238	transferase activity, transferring one-carbon groups	Sapur.009G078200 Sapur.012G002900 Sapur.001G107900 Sapur.001G121300 Sapur.002G011200 Sapur.002G018700 Sapur.002G028300 Sapur.002G028400 Sapur.002G059600 Sapur.002G060900 Sapur.002G126200 Sapur.002G149100 Sapur.002G203000 Sapur.003G068300 Sapur.005G049400 Sapur.005G180300 Sapur.005G189700 Sapur.006G031900 Sapur.007G018200 Sapur.007G018500 Sapur.007G018700 Sapur.007G093300 Sapur.008G046900 Sapur.008G074800 Sapur.008G119300 Sapur.009G044900 Sapur.010G019700 Sapur.010G078700 Sapur.010G119300 Sapur.010G122700 Sapur.010G144700 Sapur.010G156300 Sapur.011G109500 Sapur.012G050000 Sapur.014G059500 Sapur.014G059800 Sapur.016G149600 Sapur.016G215600 Sapur.017G100800 Sapur.017G111600 Sapur.018G060800 Sapur.15ZG033400 Sapur.004G005600 Sapur.011G005900
GO:0016783	3	5	sulfurtransferase activity	Sapur.005G067900 Sapur.007G074100 Sapur.019G098500
GO:0015098	3	5	molybdate ion transmembrane transporter activity	Sapur.003G108000 Sapur.007G049600 Sapur.15ZG006400
GO:0008198	3	5	ferrous iron binding	Sapur.004G099400 Sapur.019G000700 Sapur.019G000900
GO:0016992	3	5	lipoate synthase activity	Sapur.005G067900 Sapur.007G074100 Sapur.019G098500
GO:0003830	3	5	beta-1,4-mannosylglycoprotein 4-beta-N-acetylglucosaminyltransferase activity	Sapur.006G054000 Sapur.008G149700 Sapur.010G023600
GO:0016670	3	5	oxidoreductase activity, acting on a sulfur group of donors, oxygen as acceptor	Sapur.007G091200 Sapur.008G102400 Sapur.010G088200
GO:004	6	17	proton-transport	Sapur.003G054000 Sapur.005G172700 Sapur.010G202000 Sapur.013G047900 Sapur.013G064900 Sapur.T031900

6961			ing ATPase activity, rotational mechanism	
GO:0044769	6	17	ATPase activity, coupled to transmembrane movement of ions, rotational mechanism	Sapur.003G054000 Sapur.005G172700 Sapur.010G202000 Sapur.013G047900 Sapur.013G064900 Sapur.T031900
GO:0042625	6	17	ATPase-coupled ion transmembrane transporter activity	Sapur.003G054000 Sapur.005G172700 Sapur.010G202000 Sapur.013G047900 Sapur.013G064900 Sapur.T031900
GO:0019829	6	17	ATPase-coupled monoatomic cation transmembrane transporter activity	Sapur.003G054000 Sapur.005G172700 Sapur.010G202000 Sapur.013G047900 Sapur.013G064900 Sapur.T031900
GO:0120227	4	9	acyl-CoA binding	Sapur.003G069400 Sapur.005G020400 Sapur.012G011100 Sapur.013G019000
GO:0000062	4	9	fatty-acyl-CoA binding	Sapur.003G069400 Sapur.005G020400 Sapur.012G011100 Sapur.013G019000
GO:1901567	4	9	fatty acid derivative binding	Sapur.003G069400 Sapur.005G020400 Sapur.012G011100 Sapur.013G019000
GO:0016817	52	293	hydrolase activity, acting on acid anhydrides	Sapur.001G086500 Sapur.001G185700 Sapur.002G015900 Sapur.002G028100 Sapur.002G034900 Sapur.002G090500 Sapur.002G146800 Sapur.003G000800 Sapur.003G061600 Sapur.003G090100 Sapur.003G091100 Sapur.003G166900 Sapur.004G021600 Sapur.005G047400 Sapur.005G180900 Sapur.005G192400 Sapur.006G025400 Sapur.006G037500 Sapur.006G102100 Sapur.006G150200 Sapur.006G180500 Sapur.009G052100 Sapur.009G052200 Sapur.009G066800 Sapur.010G069300 Sapur.010G180200 Sapur.011G119600 Sapur.012G018300 Sapur.013G016100 Sapur.014G089900 Sapur.014G104400 Sapur.016G030100 Sapur.016G094800 Sapur.016G158100 Sapur.016G158200 Sapur.016G172500 Sapur.016G226600 Sapur.017G067400 Sapur.018G053400 Sapur.018G081000 Sapur.019G002100

				Sapur.019G002700 Sapur.019G035200 Sapur.15WG078100 Sapur.15ZG004600 Sapur.T047800 Sapur.T114400 Sapur.002G137300 Sapur.006G050300 Sapur.006G067100 Sapur.010G205600 Sapur.018G096200
GO:0016818	52	293	hydrolase activity, acting on acid anhydrides, in phosphorus-containing anhydrides	Sapur.001G086500 Sapur.001G185700 Sapur.002G015900 Sapur.002G028100 Sapur.002G034900 Sapur.002G090500 Sapur.002G146800 Sapur.003G000800 Sapur.003G061600 Sapur.003G090100 Sapur.003G091100 Sapur.003G166900 Sapur.004G021600 Sapur.005G047400 Sapur.005G180900 Sapur.005G192400 Sapur.006G025400 Sapur.006G037500 Sapur.006G102100 Sapur.006G150200 Sapur.006G180500 Sapur.009G052100 Sapur.009G052200 Sapur.009G066800 Sapur.010G069300 Sapur.010G180200 Sapur.011G119600 Sapur.012G018300 Sapur.013G016100 Sapur.014G089900 Sapur.014G104400 Sapur.016G030100 Sapur.016G094800 Sapur.016G158100 Sapur.016G158200 Sapur.016G172500 Sapur.016G226600 Sapur.017G067400 Sapur.018G053400 Sapur.018G081000 Sapur.019G002100 Sapur.019G002700 Sapur.019G035200 Sapur.15WG078100 Sapur.15ZG004600 Sapur.T047800 Sapur.T114400 Sapur.002G137300 Sapur.006G050300 Sapur.006G067100 Sapur.010G205600 Sapur.018G096200
GO:0017111	47	266	ribonucleoside triphosphate phosphatase activity	Sapur.001G086500 Sapur.001G185700 Sapur.002G015900 Sapur.002G028100 Sapur.002G034900 Sapur.002G090500 Sapur.002G146800 Sapur.003G000800 Sapur.003G061600 Sapur.003G090100 Sapur.003G091100 Sapur.003G166900 Sapur.004G021600 Sapur.005G047400 Sapur.005G180900 Sapur.005G192400 Sapur.006G025400 Sapur.006G037500 Sapur.006G102100 Sapur.006G150200 Sapur.006G180500 Sapur.009G052100 Sapur.009G052200 Sapur.009G066800 Sapur.010G069300 Sapur.010G180200 Sapur.011G119600 Sapur.012G018300 Sapur.013G016100 Sapur.014G089900 Sapur.014G104400 Sapur.016G030100 Sapur.016G094800 Sapur.016G158100 Sapur.016G158200 Sapur.016G172500 Sapur.016G226600 Sapur.017G067400 Sapur.018G053400 Sapur.018G081000 Sapur.019G002100 Sapur.019G002700 Sapur.019G035200 Sapur.15WG078100 Sapur.15ZG004600 Sapur.T047800 Sapur.T114400
GO:0008312	6	19	7S RNA binding	Sapur.005G174600 Sapur.012G030400 Sapur.15ZG029200 Sapur.15ZG029900 Sapur.15ZG031600 Sapur.15ZG032200
GO:0035639	307	2059	purine ribonucleoside triphosphate binding	Sapur.001G000500 Sapur.001G015800 Sapur.001G033500 Sapur.001G043100 Sapur.001G078000 Sapur.001G081000 Sapur.001G093700 Sapur.001G103900 Sapur.001G122500 Sapur.001G127100 Sapur.001G148700 Sapur.001G161400 Sapur.001G171200 Sapur.001G175900 Sapur.001G184300 Sapur.001G185700 Sapur.001G190300 Sapur.001G191000 Sapur.002G013800 Sapur.002G015100 Sapur.002G028000 Sapur.002G028100 Sapur.002G034900 Sapur.002G046900 Sapur.002G047800 Sapur.002G049800 Sapur.002G055700 Sapur.002G058200 Sapur.002G106900 Sapur.002G109200 Sapur.002G109900 Sapur.002G112600 Sapur.002G120400 Sapur.002G122000 Sapur.002G141400 Sapur.002G159800 Sapur.002G168400 Sapur.002G176000 Sapur.002G180800 Sapur.002G182100 Sapur.002G188100 Sapur.002G192000 Sapur.002G199800 Sapur.002G201200 Sapur.002G201300 Sapur.003G000800 Sapur.003G013500 Sapur.003G020100 Sapur.003G021100 Sapur.003G028100 Sapur.003G028400 Sapur.003G036600 Sapur.003G050300 Sapur.003G051100 Sapur.003G052500 Sapur.003G054600 Sapur.003G061600 Sapur.003G061800 Sapur.003G067600 Sapur.003G073200 Sapur.003G085300 Sapur.003G095700 Sapur.003G099700 Sapur.003G131900 Sapur.003G138700 Sapur.003G169700 Sapur.004G021600 Sapur.004G029200 Sapur.004G032900 Sapur.004G046500 Sapur.004G061000

Sapur.004G061100	Sapur.004G067800	Sapur.004G068600	Sapur.004G087900	Sapur.004G115200	Sapur.004G115800
Sapur.004G120900	Sapur.004G122400	Sapur.004G155300	Sapur.004G170800	Sapur.004G171500	Sapur.005G001800
Sapur.005G006600	Sapur.005G015200	Sapur.005G031100	Sapur.005G039800	Sapur.005G047400	Sapur.005G048100
Sapur.005G050800	Sapur.005G057200	Sapur.005G058600	Sapur.005G065000	Sapur.005G065300	Sapur.005G070300
Sapur.005G081600	Sapur.005G109200	Sapur.005G143900	Sapur.005G148400	Sapur.005G160000	Sapur.005G173800
Sapur.005G180900	Sapur.005G193300	Sapur.005G202000	Sapur.005G205600	Sapur.005G208100	Sapur.006G000900
Sapur.006G037500	Sapur.006G043700	Sapur.006G056900	Sapur.006G063800	Sapur.006G064600	Sapur.006G096300
Sapur.006G101400	Sapur.006G102100	Sapur.006G107000	Sapur.006G108600	Sapur.006G120600	Sapur.006G140000
Sapur.006G150200	Sapur.006G151400	Sapur.006G159000	Sapur.006G177500	Sapur.006G185100	Sapur.006G195000
Sapur.006G196200	Sapur.006G210500	Sapur.006G217500	Sapur.007G003100	Sapur.007G012300	Sapur.007G012500
Sapur.007G017700	Sapur.007G037800	Sapur.007G043900	Sapur.007G064500	Sapur.007G077400	Sapur.007G085900
Sapur.007G094400	Sapur.007G124000	Sapur.007G124100	Sapur.008G016100	Sapur.008G025400	Sapur.008G048100
Sapur.008G054200	Sapur.008G076100	Sapur.008G078200	Sapur.008G078900	Sapur.008G113700	Sapur.008G136200
Sapur.008G136400	Sapur.008G143200	Sapur.008G147700	Sapur.008G153800	Sapur.008G165900	Sapur.009G018700
Sapur.009G020400	Sapur.009G024900	Sapur.009G030800	Sapur.009G059300	Sapur.009G068500	Sapur.009G090900
Sapur.009G091500	Sapur.009G097000	Sapur.009G107200	Sapur.009G107300	Sapur.009G124500	Sapur.010G000800
Sapur.010G008800	Sapur.010G041500	Sapur.010G069300	Sapur.010G073200	Sapur.010G075700	Sapur.010G076000
Sapur.010G076900	Sapur.010G103300	Sapur.010G110900	Sapur.010G118800	Sapur.010G119600	Sapur.010G147800
Sapur.010G180200	Sapur.010G183900	Sapur.010G189000	Sapur.010G199900	Sapur.010G205000	Sapur.011G036600
Sapur.011G036900	Sapur.011G040500	Sapur.011G040600	Sapur.011G042000	Sapur.011G042200	Sapur.011G047800
Sapur.011G080000	Sapur.011G080600	Sapur.011G091100	Sapur.011G097800	Sapur.011G100700	Sapur.011G101300
Sapur.011G108300	Sapur.011G108500	Sapur.011G122000	Sapur.012G018300	Sapur.012G020400	Sapur.012G029700
Sapur.012G032600	Sapur.012G041400	Sapur.012G052500	Sapur.012G057100	Sapur.012G063200	Sapur.012G064400
Sapur.012G064900	Sapur.012G071500	Sapur.012G086800	Sapur.012G088300	Sapur.012G093400	Sapur.013G003400
Sapur.013G009200	Sapur.013G009300	Sapur.013G010400	Sapur.013G016100	Sapur.013G024200	Sapur.013G055800
Sapur.013G084200	Sapur.013G108700	Sapur.013G112200	Sapur.013G112400	Sapur.013G141900	Sapur.014G004600
Sapur.014G014200	Sapur.014G014400	Sapur.014G017800	Sapur.014G039100	Sapur.014G050200	Sapur.014G054500
Sapur.014G065600	Sapur.014G067100	Sapur.014G067400	Sapur.014G069000	Sapur.014G082600	Sapur.014G084200
Sapur.014G089900	Sapur.014G091500	Sapur.014G095200	Sapur.014G104400	Sapur.014G110500	Sapur.014G119600
Sapur.014G126200	Sapur.016G002900	Sapur.016G045300	Sapur.016G046200	Sapur.016G056900	Sapur.016G077400
Sapur.016G090700	Sapur.016G115900	Sapur.016G127100	Sapur.016G164800	Sapur.016G165400	Sapur.016G174600
Sapur.016G201500	Sapur.016G220800	Sapur.016G226600	Sapur.016G240200	Sapur.016G244800	Sapur.016G256600
Sapur.016G265600	Sapur.016G275100	Sapur.016G277800	Sapur.016G293800	Sapur.016G294500	Sapur.017G001800

				Sapur.017G002000 Sapur.017G067400 Sapur.017G072700 Sapur.017G096900 Sapur.017G097600 Sapur.017G107900 Sapur.017G111400 Sapur.018G009700 Sapur.018G035500 Sapur.018G053200 Sapur.018G053400 Sapur.018G058100 Sapur.018G060300 Sapur.018G067300 Sapur.018G072500 Sapur.018G081000 Sapur.019G008900 Sapur.019G062400 Sapur.019G083400 Sapur.019G087800 Sapur.019G087900 Sapur.019G113000 Sapur.15WG028200 Sapur.15WG031300 Sapur.15WG078100 Sapur.15WG134200 Sapur.15ZG004600 Sapur.15ZG018000 Sapur.15ZG027900 Sapur.15ZG035300 Sapur.15ZG082200 Sapur.15ZG116400 Sapur.15ZG124400 Sapur.15ZG134100 Sapur.T017000 Sapur.T044700 Sapur.T044800 Sapur.T047800
GO:0008137	3	6	NADH dehydrogenase (ubiquinone) activity	Sapur.001G089300 Sapur.003G086600 Sapur.008G114600
GO:0004014	3	6	adenosylmethionine decarboxylase activity	Sapur.008G171500 Sapur.010G013500 Sapur.010G104400
GO:0003954	3	6	NADH dehydrogenase activity	Sapur.001G089300 Sapur.003G086600 Sapur.008G114600
GO:0003955	3	6	NAD(P)H dehydrogenase (quinone) activity	Sapur.001G089300 Sapur.003G086600 Sapur.008G114600
GO:0008373	3	6	sialyltransferase activity	Sapur.004G138500 Sapur.014G118100 Sapur.15ZG101800
GO:0050136	3	6	NADH dehydrogenase (quinone) activity	Sapur.001G089300 Sapur.003G086600 Sapur.008G114600
GO:0008168	42	235	methyltransferase activity	Sapur.009G078200 Sapur.012G002900 Sapur.001G107900 Sapur.001G121300 Sapur.002G011200 Sapur.002G018700 Sapur.002G028300 Sapur.002G028400 Sapur.002G059600 Sapur.002G060900 Sapur.002G126200 Sapur.002G149100 Sapur.002G203000 Sapur.003G068300 Sapur.005G049400 Sapur.005G180300 Sapur.005G189700 Sapur.006G031900 Sapur.007G018200 Sapur.007G018500 Sapur.007G018700 Sapur.007G093300 Sapur.008G046900 Sapur.008G074800 Sapur.008G119300 Sapur.009G044900 Sapur.010G019700 Sapur.010G078700 Sapur.010G119300 Sapur.010G122700 Sapur.010G144700 Sapur.010G156300 Sapur.011G109500 Sapur.012G050000 Sapur.014G059500

				Sapur.014G059800 Sapur.016G149600 Sapur.016G215600 Sapur.017G100800 Sapur.017G111600 Sapur.018G060800 Sapur.15ZG033400
GO:0016772	283	1899	transferase activity, transferring phosphorus-containing groups	Sapur.001G015800 Sapur.001G033500 Sapur.001G042400 Sapur.001G043100 Sapur.001G066800 Sapur.001G078000 Sapur.001G086800 Sapur.001G093700 Sapur.001G103900 Sapur.001G137500 Sapur.001G148700 Sapur.001G171200 Sapur.001G175900 Sapur.001G190300 Sapur.001G191000 Sapur.002G010500 Sapur.002G028000 Sapur.002G046900 Sapur.002G055700 Sapur.002G106900 Sapur.002G120400 Sapur.002G164000 Sapur.002G176000 Sapur.002G180800 Sapur.002G188100 Sapur.002G192000 Sapur.002G198100 Sapur.002G199800 Sapur.002G201200 Sapur.002G201300 Sapur.003G013500 Sapur.003G037300 Sapur.003G042700 Sapur.003G051100 Sapur.003G052500 Sapur.003G061800 Sapur.003G067600 Sapur.003G073200 Sapur.003G075500 Sapur.003G082200 Sapur.003G085300 Sapur.003G089700 Sapur.003G095700 Sapur.003G099700 Sapur.003G131900 Sapur.003G133000 Sapur.003G138700 Sapur.004G009000 Sapur.004G017100 Sapur.004G023900 Sapur.004G029200 Sapur.004G032900 Sapur.004G033000 Sapur.004G044400 Sapur.004G046500 Sapur.004G057300 Sapur.004G061000 Sapur.004G067800 Sapur.004G068600 Sapur.004G080700 Sapur.004G087900 Sapur.004G115800 Sapur.004G135100 Sapur.004G170800 Sapur.004G172200 Sapur.004G177200 Sapur.005G001800 Sapur.005G011700 Sapur.005G031100 Sapur.005G039800 Sapur.005G050800 Sapur.005G057200 Sapur.005G065000 Sapur.005G065300 Sapur.005G081600 Sapur.005G109200 Sapur.005G143900 Sapur.005G148400 Sapur.005G160000 Sapur.005G202000 Sapur.005G203500 Sapur.005G205600 Sapur.005G208100 Sapur.006G039400 Sapur.006G043700 Sapur.006G044000 Sapur.006G059800 Sapur.006G063800 Sapur.006G064600 Sapur.006G077000 Sapur.006G090100 Sapur.006G093700 Sapur.006G096300 Sapur.006G107000 Sapur.006G117000 Sapur.006G151400 Sapur.006G185100 Sapur.006G195000 Sapur.006G196200 Sapur.006G210500 Sapur.006G215000 Sapur.006G217500 Sapur.007G003100 Sapur.007G012300 Sapur.007G037800 Sapur.007G044000 Sapur.007G054000 Sapur.007G077400 Sapur.007G085900 Sapur.007G124000 Sapur.007G124100 Sapur.008G008800 Sapur.008G016100 Sapur.008G054200 Sapur.008G054500 Sapur.008G076100 Sapur.008G089200 Sapur.008G107400 Sapur.008G111800 Sapur.008G113700 Sapur.008G116800 Sapur.008G136200 Sapur.008G136400 Sapur.008G143200 Sapur.008G165900 Sapur.009G030800 Sapur.009G058900 Sapur.009G059300 Sapur.009G091500 Sapur.009G092000 Sapur.009G107300 Sapur.010G000800 Sapur.010G028600 Sapur.010G041500 Sapur.010G052000 Sapur.010G064500 Sapur.010G070900 Sapur.010G075700 Sapur.010G076000 Sapur.010G076900 Sapur.010G127100 Sapur.010G145100 Sapur.010G147600 Sapur.010G147800 Sapur.010G169400 Sapur.010G189000 Sapur.011G021000 Sapur.011G030700 Sapur.011G036900 Sapur.011G040500 Sapur.011G040600 Sapur.011G046900 Sapur.011G047800 Sapur.011G050900 Sapur.011G080000 Sapur.011G080600 Sapur.011G089900 Sapur.011G095700 Sapur.011G097200 Sapur.011G101300 Sapur.011G108300 Sapur.011G121500 Sapur.012G020400 Sapur.012G029700 Sapur.012G032600 Sapur.012G041400 Sapur.012G048500 Sapur.012G051500 Sapur.012G052000 Sapur.012G054500 Sapur.012G063200 Sapur.012G064400 Sapur.012G064900 Sapur.012G071500 Sapur.012G093400 Sapur.012G097700 Sapur.012G106800 Sapur.013G003400 Sapur.013G034500 Sapur.013G055800 Sapur.013G108700 Sapur.013G141900 Sapur.014G014200 Sapur.014G017800 Sapur.014G050200

					Sapur.014G054500 Sapur.014G065600 Sapur.014G067100 Sapur.014G067400 Sapur.014G069000 Sapur.014G084200 Sapur.014G091100 Sapur.014G095200 Sapur.014G110500 Sapur.014G119600 Sapur.014G126200 Sapur.016G045300 Sapur.016G046200 Sapur.016G048600 Sapur.016G049500 Sapur.016G064900 Sapur.016G090700 Sapur.016G113500 Sapur.016G115900 Sapur.016G122900 Sapur.016G133200 Sapur.016G164800 Sapur.016G165400 Sapur.016G222400 Sapur.016G256600 Sapur.016G263500 Sapur.016G267500 Sapur.016G267900 Sapur.016G269800 Sapur.016G272100 Sapur.016G274100 Sapur.016G293800 Sapur.016G294500 Sapur.016G304300 Sapur.017G001800 Sapur.017G002000 Sapur.017G019000 Sapur.017G072700 Sapur.017G096900 Sapur.017G097600 Sapur.017G107900 Sapur.017G111400 Sapur.017G114100 Sapur.018G018700 Sapur.018G035500 Sapur.018G048200 Sapur.018G053200 Sapur.018G058300 Sapur.018G060300 Sapur.018G067300 Sapur.018G072700 Sapur.018G082400 Sapur.018G086200 Sapur.018G107900 Sapur.018G114200 Sapur.019G008900 Sapur.019G061700 Sapur.019G062400 Sapur.019G083400 Sapur.019G099500 Sapur.019G113000 Sapur.15WG028200 Sapur.15WG031300 Sapur.15WG066000 Sapur.15WG079300 Sapur.15ZG018000 Sapur.15ZG027900 Sapur.15ZG035300 Sapur.15ZG054200 Sapur.15ZG082200 Sapur.15ZG085600 Sapur.15ZG093000 Sapur.15ZG124400 Sapur.15ZG134100 Sapur.T044700 Sapur.T051500 Sapur.001G161400 Sapur.002G001800 Sapur.002G047800 Sapur.003G038600 Sapur.004G114100 Sapur.004G114400 Sapur.005G173800 Sapur.005G206700 Sapur.006G126300 Sapur.006G194700 Sapur.010G199900 Sapur.011G009700 Sapur.011G122000 Sapur.012G083700 Sapur.013G024200 Sapur.013G132500 Sapur.014G077500 Sapur.016G244800 Sapur.016G266500 Sapur.002G061200 Sapur.003G043700 Sapur.004G052000
	GO:0016860	7	25	intramolecular oxidoreductase activity	Sapur.005G040300 Sapur.013G037300 Sapur.004G127000 Sapur.008G044000 Sapur.009G103000 Sapur.010G159800 Sapur.016G133000
	GO:0019200	6	20	carbohydrate kinase activity	Sapur.002G001800 Sapur.005G206700 Sapur.006G126300 Sapur.006G194700 Sapur.011G009700 Sapur.001G161400
**m_up_MF**	GO:0043531	136	351	ADP binding	Sapur.001G023800 Sapur.003G155400 Sapur.004G147900 Sapur.005G005000 Sapur.005G005700 Sapur.005G007100 Sapur.005G011200 Sapur.005G022300 Sapur.005G023100 Sapur.005G024800 Sapur.005G025000 Sapur.005G025400 Sapur.005G031700 Sapur.005G096300 Sapur.006G010200 Sapur.006G218800 Sapur.006G223700 Sapur.006G224200 Sapur.006G225500 Sapur.007G036500 Sapur.009G117400 Sapur.011G031900 Sapur.011G088900 Sapur.012G086700 Sapur.012G087900 Sapur.012G091400 Sapur.012G091800 Sapur.012G092600 Sapur.012G092900 Sapur.013G117900 Sapur.013G118200 Sapur.013G118400 Sapur.013G120100 Sapur.013G120600 Sapur.013G123000 Sapur.013G123300 Sapur.013G123700 Sapur.013G124400 Sapur.014G001300 Sapur.014G001700 Sapur.014G002600 Sapur.014G025600 Sapur.016G009300 Sapur.016G051900 Sapur.016G052700 Sapur.016G150200 Sapur.016G189500 Sapur.016G189900 Sapur.016G200900 Sapur.016G243200 Sapur.016G243400 Sapur.016G264800 Sapur.016G282700 Sapur.016G288500 Sapur.016G302100 Sapur.016G302500 Sapur.017G010500 Sapur.017G018700 Sapur.017G023600 Sapur.017G023800 Sapur.017G052400 Sapur.017G057300 Sapur.017G100600 Sapur.017G101100 Sapur.017G101200

				Sapur.017G108100 Sapur.017G108200 Sapur.017G108700 Sapur.017G110500 Sapur.017G110700 Sapur.017G111200 Sapur.017G112200 Sapur.017G112400 Sapur.017G113400 Sapur.017G123300 Sapur.018G002400 Sapur.018G003500 Sapur.018G003800 Sapur.018G004300 Sapur.018G008500 Sapur.018G016300 Sapur.018G016500 Sapur.018G016600 Sapur.018G016800 Sapur.018G017000 Sapur.018G105800 Sapur.019G001100 Sapur.019G005900 Sapur.019G007100 Sapur.019G007900 Sapur.019G009300 Sapur.019G009600 Sapur.019G010500 Sapur.019G010700 Sapur.019G011500 Sapur.019G011600 Sapur.019G011700 Sapur.019G011900 Sapur.019G012500 Sapur.019G012700 Sapur.019G012800 Sapur.019G013700 Sapur.019G016400 Sapur.019G017000 Sapur.019G022800 Sapur.019G023100 Sapur.019G027100 Sapur.019G027300 Sapur.019G034300 Sapur.019G042100 Sapur.019G042400 Sapur.019G042500 Sapur.019G043100 Sapur.019G043200 Sapur.019G044000 Sapur.019G044100 Sapur.019G046700 Sapur.019G091100 Sapur.019G091300 Sapur.019G091700 Sapur.019G092300 Sapur.019G092600 Sapur.019G092700 Sapur.019G092800 Sapur.019G093100 Sapur.019G093400 Sapur.019G093600 Sapur.019G093900 Sapur.15WG043400 Sapur.15ZG050400 Sapur.15ZG050500 Sapur.T096600 Sapur.T115500 Sapur.T116500 Sapur.T131900 Sapur.T150000
GO:0032559	447	2180	adenyl ribonucleotide binding	Sapur.001G023800 Sapur.001G029800 Sapur.001G055600 Sapur.001G058300 Sapur.001G058800 Sapur.001G078400 Sapur.001G084200 Sapur.001G094200 Sapur.001G103000 Sapur.001G108900 Sapur.001G110300 Sapur.001G151600 Sapur.001G167000 Sapur.001G181600 Sapur.002G005300 Sapur.002G013600 Sapur.002G014300 Sapur.002G024400 Sapur.002G032100 Sapur.002G051400 Sapur.002G061600 Sapur.002G061700 Sapur.002G064800 Sapur.002G085200 Sapur.002G085900 Sapur.002G089300 Sapur.002G090300 Sapur.002G130500 Sapur.002G132500 Sapur.002G145300 Sapur.002G156100 Sapur.002G161200 Sapur.002G169300 Sapur.002G194100 Sapur.003G002000 Sapur.003G018100 Sapur.003G025000 Sapur.003G031900 Sapur.003G066000 Sapur.003G067100 Sapur.003G092400 Sapur.003G099500 Sapur.003G116100 Sapur.003G125800 Sapur.003G138500 Sapur.003G139700 Sapur.003G155400 Sapur.003G160700 Sapur.003G168000 Sapur.004G014400 Sapur.004G019100 Sapur.004G027800 Sapur.004G041000 Sapur.004G051400 Sapur.004G063300 Sapur.004G077700 Sapur.004G103000 Sapur.004G109700 Sapur.004G131500 Sapur.004G137600 Sapur.004G140300 Sapur.004G146400 Sapur.004G147000 Sapur.004G147900 Sapur.004G172100 Sapur.005G000100 Sapur.005G005000 Sapur.005G005700 Sapur.005G007100 Sapur.005G011200 Sapur.005G022300 Sapur.005G023100 Sapur.005G024800 Sapur.005G025000 Sapur.005G025400 Sapur.005G031700 Sapur.005G038400 Sapur.005G043600 Sapur.005G043700 Sapur.005G048900 Sapur.005G054500 Sapur.005G055500 Sapur.005G069000 Sapur.005G085100 Sapur.005G095700 Sapur.005G096300 Sapur.005G096400 Sapur.005G109500 Sapur.005G193700 Sapur.005G199500 Sapur.005G206400 Sapur.006G010200 Sapur.006G018700 Sapur.006G025100 Sapur.006G036800 Sapur.006G067400 Sapur.006G069500 Sapur.006G072700 Sapur.006G094200 Sapur.006G094300 Sapur.006G110200 Sapur.006G112500 Sapur.006G136200 Sapur.006G136800 Sapur.006G138300 Sapur.006G154200 Sapur.006G154300 Sapur.006G163000 Sapur.006G172300 Sapur.006G181100 Sapur.006G194600 Sapur.006G218800 Sapur.006G223700 Sapur.006G224200 Sapur.006G225500 Sapur.007G002900 Sapur.007G007000 Sapur.007G007100 Sapur.007G008900 Sapur.007G016800 Sapur.007G029800 Sapur.007G036500 Sapur.007G069600 Sapur.007G071200 Sapur.007G075800

Sapur.007G077000 Sapur.007G077600 Sapur.007G087100 Sapur.007G091900 Sapur.007G111300 Sapur.007G111400
Sapur.007G111700 Sapur.007G112200 Sapur.007G115600 Sapur.008G004700 Sapur.008G009100 Sapur.008G026800
Sapur.008G045900 Sapur.008G046000 Sapur.008G058400 Sapur.008G060600 Sapur.008G066900 Sapur.008G077900
Sapur.008G081500 Sapur.008G088300 Sapur.008G090300 Sapur.008G091700 Sapur.008G115400 Sapur.008G128300
Sapur.008G131900 Sapur.008G137200 Sapur.008G151300 Sapur.008G151900 Sapur.008G156200 Sapur.009G002900
Sapur.009G004200 Sapur.009G005200 Sapur.009G018000 Sapur.009G041000 Sapur.009G045200 Sapur.009G051100
Sapur.009G057000 Sapur.009G064100 Sapur.009G066300 Sapur.009G094100 Sapur.009G094200 Sapur.009G112500
Sapur.009G116000 Sapur.009G117400 Sapur.009G122200 Sapur.010G001900 Sapur.010G009500 Sapur.010G014000
Sapur.010G024600 Sapur.010G026800 Sapur.010G042700 Sapur.010G046700 Sapur.010G052700 Sapur.010G053300
Sapur.010G055900 Sapur.010G057900 Sapur.010G060700 Sapur.010G070700 Sapur.010G086100 Sapur.010G087600
Sapur.010G089800 Sapur.010G092300 Sapur.010G092700 Sapur.010G093900 Sapur.010G100000 Sapur.010G102500
Sapur.010G104500 Sapur.010G118700 Sapur.010G119700 Sapur.010G119800 Sapur.010G137200 Sapur.010G138500
Sapur.010G141100 Sapur.010G143600 Sapur.010G157400 Sapur.010G163200 Sapur.010G187200 Sapur.010G193600
Sapur.011G008400 Sapur.011G013300 Sapur.011G018500 Sapur.011G028400 Sapur.011G031900 Sapur.011G034200
Sapur.011G073000 Sapur.011G088900 Sapur.011G103500 Sapur.011G103700 Sapur.012G000700 Sapur.012G037400
Sapur.012G040700 Sapur.012G048900 Sapur.012G050600 Sapur.012G067300 Sapur.012G070700 Sapur.012G073300
Sapur.012G084200 Sapur.012G086700 Sapur.012G087900 Sapur.012G091400 Sapur.012G091800 Sapur.012G092600
Sapur.012G092900 Sapur.012G100600 Sapur.012G101100 Sapur.013G000100 Sapur.013G021200 Sapur.013G045400
Sapur.013G055900 Sapur.013G100200 Sapur.013G117900 Sapur.013G118200 Sapur.013G118400 Sapur.013G120100
Sapur.013G120600 Sapur.013G123000 Sapur.013G123300 Sapur.013G123700 Sapur.013G124400 Sapur.014G001300
Sapur.014G001700 Sapur.014G002600 Sapur.014G005100 Sapur.014G025600 Sapur.014G036100 Sapur.014G052300
Sapur.014G053000 Sapur.014G061800 Sapur.014G073900 Sapur.014G076200 Sapur.014G084500 Sapur.014G090200
Sapur.014G090300 Sapur.014G104700 Sapur.014G106600 Sapur.014G110600 Sapur.014G113100 Sapur.014G113600
Sapur.014G117400 Sapur.014G117700 Sapur.014G120400 Sapur.014G132000 Sapur.014G134700 Sapur.016G009300
Sapur.016G031400 Sapur.016G051900 Sapur.016G052700 Sapur.016G058000 Sapur.016G071200 Sapur.016G120800
Sapur.016G125300 Sapur.016G141300 Sapur.016G150200 Sapur.016G163400 Sapur.016G189500 Sapur.016G189900
Sapur.016G200900 Sapur.016G230700 Sapur.016G230800 Sapur.016G243200 Sapur.016G243400 Sapur.016G247200
Sapur.016G262400 Sapur.016G264800 Sapur.016G281900 Sapur.016G282700 Sapur.016G288500 Sapur.016G302100
Sapur.016G302500 Sapur.017G003500 Sapur.017G009300 Sapur.017G010500 Sapur.017G018700 Sapur.017G020600
Sapur.017G023600 Sapur.017G023800 Sapur.017G029100 Sapur.017G036400 Sapur.017G036800 Sapur.017G037700
Sapur.017G052400 Sapur.017G055300 Sapur.017G057300 Sapur.017G057900 Sapur.017G074500 Sapur.017G100600
Sapur.017G101100 Sapur.017G101200 Sapur.017G102500 Sapur.017G104800 Sapur.017G108100 Sapur.017G108200
Sapur.017G108700 Sapur.017G110500 Sapur.017G110700 Sapur.017G111200 Sapur.017G112200 Sapur.017G112400

				Sapur.017G113400 Sapur.017G123300 Sapur.017G129000 Sapur.018G002400 Sapur.018G003500 Sapur.018G003800 Sapur.018G004300 Sapur.018G008500 Sapur.018G015000 Sapur.018G015800 Sapur.018G016300 Sapur.018G016500 Sapur.018G016600 Sapur.018G016800 Sapur.018G017000 Sapur.018G023200 Sapur.018G045400 Sapur.018G057600 Sapur.018G063900 Sapur.018G067200 Sapur.018G077200 Sapur.018G087600 Sapur.018G088200 Sapur.018G105800 Sapur.018G114900 Sapur.018G115800 Sapur.018G115900 Sapur.018G117400 Sapur.019G001100 Sapur.019G003100 Sapur.019G005200 Sapur.019G005900 Sapur.019G007100 Sapur.019G007800 Sapur.019G007900 Sapur.019G009300 Sapur.019G009600 Sapur.019G010500 Sapur.019G010700 Sapur.019G011000 Sapur.019G011200 Sapur.019G011500 Sapur.019G011600 Sapur.019G011700 Sapur.019G011900 Sapur.019G012100 Sapur.019G012500 Sapur.019G012700 Sapur.019G012800 Sapur.019G013700 Sapur.019G016400 Sapur.019G017000 Sapur.019G022800 Sapur.019G023100 Sapur.019G027100 Sapur.019G027300 Sapur.019G028700 Sapur.019G029100 Sapur.019G034300 Sapur.019G042100 Sapur.019G042400 Sapur.019G042500 Sapur.019G043100 Sapur.019G043200 Sapur.019G044000 Sapur.019G044100 Sapur.019G046700 Sapur.019G048800 Sapur.019G053500 Sapur.019G058700 Sapur.019G075900 Sapur.019G080700 Sapur.019G082400 Sapur.019G091100 Sapur.019G091300 Sapur.019G091700 Sapur.019G092300 Sapur.019G092600 Sapur.019G092700 Sapur.019G092800 Sapur.019G093100 Sapur.019G093400 Sapur.019G093600 Sapur.019G093900 Sapur.019G094300 Sapur.019G107300 Sapur.019G111400 Sapur.019G113800 Sapur.15WG036700 Sapur.15WG040400 Sapur.15WG043400 Sapur.15WG044400 Sapur.15WG076100 Sapur.15ZG018400 Sapur.15ZG038900 Sapur.15ZG044100 Sapur.15ZG044300 Sapur.15ZG044900 Sapur.15ZG049500 Sapur.15ZG050400 Sapur.15ZG050500 Sapur.15ZG103200 Sapur.15ZG109900 Sapur.15ZG116100 Sapur.15ZG120200 Sapur.15ZG123200 Sapur.15ZG126400 Sapur.T003900 Sapur.T005400 Sapur.T005500 Sapur.T028300 Sapur.T029400 Sapur.T030800 Sapur.T058800 Sapur.T080500 Sapur.T096600 Sapur.T115500 Sapur.T116500 Sapur.T117000 Sapur.T131900 Sapur.T133700 Sapur.T150000 Sapur.T191500 Sapur.T192100
GO:0030554	465	2280	adenyl nucleotide binding	Sapur.001G017800 Sapur.001G023800 Sapur.001G029800 Sapur.001G055600 Sapur.001G058300 Sapur.001G058800 Sapur.001G078400 Sapur.001G084200 Sapur.001G094200 Sapur.001G103000 Sapur.001G108900 Sapur.001G110300 Sapur.001G151600 Sapur.001G167000 Sapur.001G174900 Sapur.001G181600 Sapur.002G005300 Sapur.002G013600 Sapur.002G014300 Sapur.002G024400 Sapur.002G032100 Sapur.002G051400 Sapur.002G061600 Sapur.002G061700 Sapur.002G064800 Sapur.002G085200 Sapur.002G085900 Sapur.002G089300 Sapur.002G090300 Sapur.002G123400 Sapur.002G130500 Sapur.002G132500 Sapur.002G145300 Sapur.002G156100 Sapur.002G161200 Sapur.002G169300 Sapur.002G194100 Sapur.003G002000 Sapur.003G018100 Sapur.003G025000 Sapur.003G031900 Sapur.003G066000 Sapur.003G067100 Sapur.003G092400 Sapur.003G099500 Sapur.003G116100 Sapur.003G125800 Sapur.003G138500 Sapur.003G139700 Sapur.003G155400 Sapur.003G160700 Sapur.003G168000 Sapur.004G014400 Sapur.004G019100 Sapur.004G027800 Sapur.004G041000 Sapur.004G051400 Sapur.004G063300 Sapur.004G077700 Sapur.004G103000 Sapur.004G108600 Sapur.004G109700 Sapur.004G131500 Sapur.004G132200 Sapur.004G137600 Sapur.004G140300 Sapur.004G146400 Sapur.004G147000 Sapur.004G147900 Sapur.004G172100 Sapur.005G000100

	Sapur.005G005000 Sapur.005G005700 Sapur.005G007100 Sapur.005G011200 Sapur.005G022300 Sapur.005G023100<br>Sapur.005G024800 Sapur.005G025000 Sapur.005G025400 Sapur.005G031700 Sapur.005G038400 Sapur.005G043600<br>Sapur.005G043700 Sapur.005G048900 Sapur.005G054500 Sapur.005G055500 Sapur.005G069000 Sapur.005G085100<br>Sapur.005G095700 Sapur.005G096300 Sapur.005G096400 Sapur.005G109500 Sapur.005G113600 Sapur.005G193700<br>Sapur.005G199500 Sapur.005G206400 Sapur.006G010200 Sapur.006G018700 Sapur.006G025100 Sapur.006G036800<br>Sapur.006G067400 Sapur.006G069500 Sapur.006G072700 Sapur.006G094200 Sapur.006G094300 Sapur.006G110200<br>Sapur.006G112500 Sapur.006G136200 Sapur.006G136800 Sapur.006G138300 Sapur.006G154200 Sapur.006G154300<br>Sapur.006G163000 Sapur.006G172300 Sapur.006G181100 Sapur.006G194600 Sapur.006G218800 Sapur.006G223700<br>Sapur.006G224200 Sapur.006G225500 Sapur.007G002900 Sapur.007G007000 Sapur.007G007100 Sapur.007G008900<br>Sapur.007G016800 Sapur.007G029800 Sapur.007G036500 Sapur.007G069600 Sapur.007G071200 Sapur.007G075800<br>Sapur.007G077000 Sapur.007G077600 Sapur.007G087100 Sapur.007G091900 Sapur.007G111300 Sapur.007G111400<br>Sapur.007G111700 Sapur.007G112200 Sapur.007G115600 Sapur.008G004700 Sapur.008G004900 Sapur.008G009100<br>Sapur.008G026800 Sapur.008G045900 Sapur.008G046000 Sapur.008G058400 Sapur.008G060600 Sapur.008G066900<br>Sapur.008G077900 Sapur.008G081500 Sapur.008G088300 Sapur.008G090300 Sapur.008G091700 Sapur.008G115400<br>Sapur.008G128300 Sapur.008G131900 Sapur.008G137200 Sapur.008G151300 Sapur.008G151900 Sapur.008G156200<br>Sapur.009G002900 Sapur.009G004200 Sapur.009G005200 Sapur.009G018000 Sapur.009G041000 Sapur.009G045200<br>Sapur.009G051100 Sapur.009G057000 Sapur.009G064100 Sapur.009G066300 Sapur.009G094100 Sapur.009G094200<br>Sapur.009G112500 Sapur.009G116000 Sapur.009G117400 Sapur.009G122200 Sapur.010G001900 Sapur.010G009500<br>Sapur.010G014000 Sapur.010G024600 Sapur.010G026800 Sapur.010G042700 Sapur.010G046700 Sapur.010G052700<br>Sapur.010G053300 Sapur.010G055900 Sapur.010G057900 Sapur.010G060700 Sapur.010G070700 Sapur.010G086100<br>Sapur.010G087600 Sapur.010G089800 Sapur.010G092300 Sapur.010G092700 Sapur.010G093900 Sapur.010G100000<br>Sapur.010G102500 Sapur.010G104500 Sapur.010G118700 Sapur.010G119700 Sapur.010G119800 Sapur.010G137200<br>Sapur.010G138500 Sapur.010G141100 Sapur.010G143600 Sapur.010G157400 Sapur.010G163200 Sapur.010G187200<br>Sapur.010G193600 Sapur.011G008400 Sapur.011G013300 Sapur.011G018500 Sapur.011G028400 Sapur.011G031900<br>Sapur.011G034200 Sapur.011G073000 Sapur.011G088900 Sapur.011G103500 Sapur.011G103700 Sapur.011G120500<br>Sapur.012G000700 Sapur.012G037400 Sapur.012G040700 Sapur.012G048900 Sapur.012G050600 Sapur.012G067300<br>Sapur.012G070700 Sapur.012G073300 Sapur.012G084200 Sapur.012G086700 Sapur.012G087900 Sapur.012G091400<br>Sapur.012G091800 Sapur.012G092600 Sapur.012G092900 Sapur.012G100600 Sapur.012G101100 Sapur.013G000100<br>Sapur.013G004800 Sapur.013G021200 Sapur.013G045400 Sapur.013G055900 Sapur.013G100200 Sapur.013G117900<br>Sapur.013G118200 Sapur.013G118400 Sapur.013G120100 Sapur.013G120600 Sapur.013G123000 Sapur.013G123300<br>Sapur.013G123700 Sapur.013G124400 Sapur.014G001300 Sapur.014G001700 Sapur.014G002600 Sapur.014G005100<br>Sapur.014G025600 Sapur.014G036100 Sapur.014G052300 Sapur.014G053000 Sapur.014G055200 Sapur.014G055500<br>Sapur.014G061800 Sapur.014G073900 Sapur.014G076200 Sapur.014G084500 Sapur.014G090200 Sapur.014G090300

Sapur.014G104700 Sapur.014G106600 Sapur.014G110600 Sapur.014G113100 Sapur.014G113600 Sapur.014G117400
Sapur.014G117700 Sapur.014G120400 Sapur.014G132000 Sapur.014G134700 Sapur.014G135900 Sapur.016G009300
Sapur.016G031400 Sapur.016G040000 Sapur.016G051900 Sapur.016G052700 Sapur.016G058000 Sapur.016G071200
Sapur.016G120800 Sapur.016G125300 Sapur.016G141300 Sapur.016G150200 Sapur.016G163400 Sapur.016G189500
Sapur.016G189900 Sapur.016G200900 Sapur.016G230700 Sapur.016G230800 Sapur.016G243200 Sapur.016G243400
Sapur.016G247200 Sapur.016G262400 Sapur.016G264800 Sapur.016G281900 Sapur.016G282700 Sapur.016G288500
Sapur.016G302100 Sapur.016G302500 Sapur.016G304100 Sapur.017G003500 Sapur.017G009300 Sapur.017G010500
Sapur.017G018700 Sapur.017G020600 Sapur.017G023600 Sapur.017G023800 Sapur.017G029100 Sapur.017G036400
Sapur.017G036800 Sapur.017G037700 Sapur.017G052400 Sapur.017G055300 Sapur.017G057300 Sapur.017G057900
Sapur.017G074500 Sapur.017G100600 Sapur.017G101100 Sapur.017G101200 Sapur.017G102500 Sapur.017G104800
Sapur.017G108100 Sapur.017G108200 Sapur.017G108700 Sapur.017G110500 Sapur.017G110700 Sapur.017G111200
Sapur.017G112200 Sapur.017G112400 Sapur.017G113400 Sapur.017G123300 Sapur.017G129000 Sapur.018G002400
Sapur.018G003500 Sapur.018G003800 Sapur.018G004300 Sapur.018G008500 Sapur.018G015000 Sapur.018G015800
Sapur.018G016300 Sapur.018G016500 Sapur.018G016600 Sapur.018G016800 Sapur.018G017000 Sapur.018G023200
Sapur.018G045400 Sapur.018G057600 Sapur.018G063600 Sapur.018G063900 Sapur.018G067200 Sapur.018G077200
Sapur.018G087600 Sapur.018G088200 Sapur.018G105800 Sapur.018G114900 Sapur.018G115800 Sapur.018G115900
Sapur.018G117400 Sapur.019G001100 Sapur.019G003100 Sapur.019G005200 Sapur.019G005900 Sapur.019G007100
Sapur.019G007800 Sapur.019G007900 Sapur.019G009300 Sapur.019G009600 Sapur.019G010500 Sapur.019G010700
Sapur.019G011000 Sapur.019G011200 Sapur.019G011500 Sapur.019G011600 Sapur.019G011700 Sapur.019G011900
Sapur.019G012100 Sapur.019G012500 Sapur.019G012700 Sapur.019G012800 Sapur.019G013700 Sapur.019G016400
Sapur.019G017000 Sapur.019G022800 Sapur.019G023100 Sapur.019G027100 Sapur.019G027300 Sapur.019G028700
Sapur.019G029100 Sapur.019G034300 Sapur.019G042100 Sapur.019G042400 Sapur.019G042500 Sapur.019G043100
Sapur.019G043200 Sapur.019G044000 Sapur.019G044100 Sapur.019G046700 Sapur.019G048800 Sapur.019G053500
Sapur.019G058700 Sapur.019G070000 Sapur.019G075900 Sapur.019G080700 Sapur.019G082400 Sapur.019G091100
Sapur.019G091300 Sapur.019G091700 Sapur.019G092300 Sapur.019G092600 Sapur.019G092700 Sapur.019G092800
Sapur.019G093100 Sapur.019G093400 Sapur.019G093600 Sapur.019G093900 Sapur.019G094300 Sapur.019G107300
Sapur.019G111400 Sapur.019G113800 Sapur.15WG030400 Sapur.15WG036700 Sapur.15WG040400 Sapur.15WG043400
Sapur.15WG044400 Sapur.15WG076100 Sapur.15ZG018400 Sapur.15ZG038900 Sapur.15ZG044100 Sapur.15ZG044300
Sapur.15ZG044900 Sapur.15ZG049500 Sapur.15ZG050400 Sapur.15ZG050500 Sapur.15ZG078500 Sapur.15ZG103200
Sapur.15ZG109900 Sapur.15ZG116100 Sapur.15ZG120200 Sapur.15ZG123200 Sapur.15ZG126400 Sapur.T003900
Sapur.T005400 Sapur.T005500 Sapur.T028300 Sapur.T029400 Sapur.T030800 Sapur.T058800 Sapur.T080500
Sapur.T096600 Sapur.T115500 Sapur.T116500 Sapur.T117000 Sapur.T131900 Sapur.T133700 Sapur.T150000
Sapur.T191500 Sapur.T192100

GO:0097367	495	2474	carbohydrate derivative binding	Sapur.001G023800 Sapur.001G029800 Sapur.001G055600 Sapur.001G058300 Sapur.001G058800 Sapur.001G078400 Sapur.001G078600 Sapur.001G084200 Sapur.001G094200 Sapur.001G103000 Sapur.001G108900 Sapur.001G110300 Sapur.001G122200 Sapur.001G137000 Sapur.001G151600 Sapur.001G167000 Sapur.001G181600 Sapur.001G189000 Sapur.002G005300 Sapur.002G013600 Sapur.002G014300 Sapur.002G023300 Sapur.002G024400 Sapur.002G032100 Sapur.002G051400 Sapur.002G061600 Sapur.002G061700 Sapur.002G064800 Sapur.002G085200 Sapur.002G085900 Sapur.002G089300 Sapur.002G090300 Sapur.002G109500 Sapur.002G130500 Sapur.002G132500 Sapur.002G145300 Sapur.002G156100 Sapur.002G161200 Sapur.002G169300 Sapur.002G191900 Sapur.002G194100 Sapur.003G002000 Sapur.003G002100 Sapur.003G014300 Sapur.003G018100 Sapur.003G025000 Sapur.003G031900 Sapur.003G066000 Sapur.003G067100 Sapur.003G078600 Sapur.003G092400 Sapur.003G099500 Sapur.003G116100 Sapur.003G125800 Sapur.003G138500 Sapur.003G139700 Sapur.003G155400 Sapur.003G160700 Sapur.003G168000 Sapur.004G014400 Sapur.004G019100 Sapur.004G027800 Sapur.004G033200 Sapur.004G041000 Sapur.004G051400 Sapur.004G063300 Sapur.004G077700 Sapur.004G103000 Sapur.004G109700 Sapur.004G128600 Sapur.004G131500 Sapur.004G137100 Sapur.004G137600 Sapur.004G140300 Sapur.004G146400 Sapur.004G147000 Sapur.004G147900 Sapur.004G172100 Sapur.005G000100 Sapur.005G005000 Sapur.005G005700 Sapur.005G007100 Sapur.005G011200 Sapur.005G022300 Sapur.005G023100 Sapur.005G024800 Sapur.005G025000 Sapur.005G025400 Sapur.005G031700 Sapur.005G038400 Sapur.005G043600 Sapur.005G043700 Sapur.005G048900 Sapur.005G054500 Sapur.005G055500 Sapur.005G069000 Sapur.005G085100 Sapur.005G095700 Sapur.005G096300 Sapur.005G096400 Sapur.005G109500 Sapur.005G165600 Sapur.005G193700 Sapur.005G199500 Sapur.005G206400 Sapur.006G010200 Sapur.006G018700 Sapur.006G025100 Sapur.006G036800 Sapur.006G037200 Sapur.006G067400 Sapur.006G069500 Sapur.006G072700 Sapur.006G094200 Sapur.006G094300 Sapur.006G110200 Sapur.006G112500 Sapur.006G118100 Sapur.006G118200 Sapur.006G136200 Sapur.006G136800 Sapur.006G138300 Sapur.006G154200 Sapur.006G154300 Sapur.006G163000 Sapur.006G172300 Sapur.006G173100 Sapur.006G181100 Sapur.006G194600 Sapur.006G218800 Sapur.006G221300 Sapur.006G223700 Sapur.006G224200 Sapur.006G225500 Sapur.007G002900 Sapur.007G007000 Sapur.007G007100 Sapur.007G008900 Sapur.007G016800 Sapur.007G029800 Sapur.007G036500 Sapur.007G069600 Sapur.007G071200 Sapur.007G075800 Sapur.007G077000 Sapur.007G077600 Sapur.007G087100 Sapur.007G091900 Sapur.007G111300 Sapur.007G111400 Sapur.007G111700 Sapur.007G112200 Sapur.007G115600 Sapur.008G004700 Sapur.008G009100 Sapur.008G022900 Sapur.008G026800 Sapur.008G045900 Sapur.008G046000 Sapur.008G058400 Sapur.008G060600 Sapur.008G066900 Sapur.008G077900 Sapur.008G081500 Sapur.008G088300 Sapur.008G090300 Sapur.008G091700 Sapur.008G115400 Sapur.008G128300 Sapur.008G131900 Sapur.008G137200 Sapur.008G151300 Sapur.008G151900 Sapur.008G156200 Sapur.008G162000 Sapur.009G002900 Sapur.009G004200 Sapur.009G005200 Sapur.009G018000 Sapur.009G041000 Sapur.009G045200 Sapur.009G051100 Sapur.009G057000 Sapur.009G064100 Sapur.009G066300 Sapur.009G094100 Sapur.009G094200 Sapur.009G112500 Sapur.009G112900 Sapur.009G116000 Sapur.009G117400 Sapur.009G120100 Sapur.009G122200 Sapur.010G001900 Sapur.010G009400 Sapur.010G009500 Sapur.010G014000

Sapur.010G024600 Sapur.010G026800 Sapur.010G042700 Sapur.010G046700 Sapur.010G052700 Sapur.010G053300
Sapur.010G055900 Sapur.010G057900 Sapur.010G060700 Sapur.010G070700 Sapur.010G086100 Sapur.010G087600
Sapur.010G089800 Sapur.010G092300 Sapur.010G092700 Sapur.010G093900 Sapur.010G100000 Sapur.010G102500
Sapur.010G104500 Sapur.010G118700 Sapur.010G119700 Sapur.010G119800 Sapur.010G137200 Sapur.010G138500
Sapur.010G141100 Sapur.010G143600 Sapur.010G157400 Sapur.010G163200 Sapur.010G187200 Sapur.010G193600
Sapur.011G008400 Sapur.011G013300 Sapur.011G018500 Sapur.011G028400 Sapur.011G031900 Sapur.011G034200
Sapur.011G073000 Sapur.011G088900 Sapur.011G103500 Sapur.011G103700 Sapur.011G103800 Sapur.012G000700
Sapur.012G037400 Sapur.012G040700 Sapur.012G048900 Sapur.012G050600 Sapur.012G061900 Sapur.012G067300
Sapur.012G070700 Sapur.012G073300 Sapur.012G084200 Sapur.012G086700 Sapur.012G087900 Sapur.012G091400
Sapur.012G091800 Sapur.012G092600 Sapur.012G092900 Sapur.012G100600 Sapur.012G101100 Sapur.013G000100
Sapur.013G021200 Sapur.013G045400 Sapur.013G055900 Sapur.013G097100 Sapur.013G100200 Sapur.013G110800
Sapur.013G117900 Sapur.013G118200 Sapur.013G118400 Sapur.013G120100 Sapur.013G120600 Sapur.013G123000
Sapur.013G123300 Sapur.013G123700 Sapur.013G124400 Sapur.014G001300 Sapur.014G001700 Sapur.014G002600
Sapur.014G005100 Sapur.014G025600 Sapur.014G032900 Sapur.014G036100 Sapur.014G052300 Sapur.014G053000
Sapur.014G061800 Sapur.014G073900 Sapur.014G076200 Sapur.014G080400 Sapur.014G084500 Sapur.014G090200
Sapur.014G090300 Sapur.014G104700 Sapur.014G106600 Sapur.014G110600 Sapur.014G113100 Sapur.014G113600
Sapur.014G117400 Sapur.014G117700 Sapur.014G120400 Sapur.014G132000 Sapur.014G134700 Sapur.016G009300
Sapur.016G018900 Sapur.016G031400 Sapur.016G035600 Sapur.016G051900 Sapur.016G052700 Sapur.016G058000
Sapur.016G071200 Sapur.016G077600 Sapur.016G120800 Sapur.016G125300 Sapur.016G132300 Sapur.016G132400
Sapur.016G141300 Sapur.016G147900 Sapur.016G150200 Sapur.016G163400 Sapur.016G189500 Sapur.016G189900
Sapur.016G200900 Sapur.016G230700 Sapur.016G230800 Sapur.016G243200 Sapur.016G243400 Sapur.016G247200
Sapur.016G262400 Sapur.016G264800 Sapur.016G281900 Sapur.016G282700 Sapur.016G288500 Sapur.016G302100
Sapur.016G302500 Sapur.017G003500 Sapur.017G009300 Sapur.017G010500 Sapur.017G018700 Sapur.017G020600
Sapur.017G023600 Sapur.017G023800 Sapur.017G029100 Sapur.017G036400 Sapur.017G036800 Sapur.017G037700
Sapur.017G052400 Sapur.017G055300 Sapur.017G057300 Sapur.017G057900 Sapur.017G074500 Sapur.017G100600
Sapur.017G101100 Sapur.017G101200 Sapur.017G102500 Sapur.017G104800 Sapur.017G108100 Sapur.017G108200
Sapur.017G108700 Sapur.017G110500 Sapur.017G110700 Sapur.017G111200 Sapur.017G112200 Sapur.017G112400
Sapur.017G113400 Sapur.017G123300 Sapur.017G129000 Sapur.018G002400 Sapur.018G003500 Sapur.018G003800
Sapur.018G004300 Sapur.018G008500 Sapur.018G015000 Sapur.018G015800 Sapur.018G016300 Sapur.018G016500
Sapur.018G016600 Sapur.018G016800 Sapur.018G017000 Sapur.018G023200 Sapur.018G025700 Sapur.018G045400
Sapur.018G047200 Sapur.018G057100 Sapur.018G057600 Sapur.018G060400 Sapur.018G063900 Sapur.018G067200
Sapur.018G077200 Sapur.018G087600 Sapur.018G088200 Sapur.018G105800 Sapur.018G114900 Sapur.018G115800
Sapur.018G115900 Sapur.018G117400 Sapur.019G001100 Sapur.019G003100 Sapur.019G005200 Sapur.019G005900

				Sapur.019G007100 Sapur.019G007800 Sapur.019G007900 Sapur.019G009300 Sapur.019G009600 Sapur.019G010500 Sapur.019G010700 Sapur.019G011000 Sapur.019G011200 Sapur.019G011500 Sapur.019G011600 Sapur.019G011700 Sapur.019G011900 Sapur.019G012100 Sapur.019G012500 Sapur.019G012700 Sapur.019G012800 Sapur.019G013700 Sapur.019G016400 Sapur.019G017000 Sapur.019G022800 Sapur.019G023100 Sapur.019G027100 Sapur.019G027300 Sapur.019G028700 Sapur.019G029100 Sapur.019G034300 Sapur.019G042100 Sapur.019G042400 Sapur.019G042500 Sapur.019G043100 Sapur.019G043200 Sapur.019G044000 Sapur.019G044100 Sapur.019G046700 Sapur.019G048800 Sapur.019G053500 Sapur.019G058700 Sapur.019G075900 Sapur.019G080700 Sapur.019G082400 Sapur.019G091100 Sapur.019G091300 Sapur.019G091700 Sapur.019G092300 Sapur.019G092600 Sapur.019G092700 Sapur.019G092800 Sapur.019G093100 Sapur.019G093400 Sapur.019G093600 Sapur.019G093900 Sapur.019G094300 Sapur.019G107300 Sapur.019G110800 Sapur.019G111400 Sapur.019G113800 Sapur.15WG036700 Sapur.15WG040400 Sapur.15WG043400 Sapur.15WG044400 Sapur.15WG076100 Sapur.15ZG018400 Sapur.15ZG038900 Sapur.15ZG044100 Sapur.15ZG044300 Sapur.15ZG044900 Sapur.15ZG049500 Sapur.15ZG050400 Sapur.15ZG050500 Sapur.15ZG103200 Sapur.15ZG109900 Sapur.15ZG116100 Sapur.15ZG120200 Sapur.15ZG123200 Sapur.15ZG126400 Sapur.T003900 Sapur.T005400 Sapur.T005500 Sapur.T028300 Sapur.T029400 Sapur.T030800 Sapur.T058800 Sapur.T080500 Sapur.T096600 Sapur.T115500 Sapur.T116500 Sapur.T117000 Sapur.T131900 Sapur.T133700 Sapur.T147200 Sapur.T150000 Sapur.T191500 Sapur.T192100 Sapur.002G055900 Sapur.008G096200 Sapur.008G137000 Sapur.001G025400 Sapur.019G101700 Sapur.15ZG002000
GO:0043167	858	4562	ion binding	Sapur.001G029700 Sapur.001G067900 Sapur.001G081300 Sapur.001G130300 Sapur.001G136400 Sapur.001G148900 Sapur.001G149000 Sapur.001G149100 Sapur.001G167600 Sapur.001G193000 Sapur.002G003300 Sapur.002G009700 Sapur.002G019400 Sapur.002G036100 Sapur.002G044200 Sapur.002G061100 Sapur.002G103300 Sapur.002G106000 Sapur.002G107000 Sapur.002G141800 Sapur.002G150200 Sapur.003G003700 Sapur.003G037400 Sapur.003G037500 Sapur.003G060800 Sapur.003G082800 Sapur.003G090400 Sapur.003G093200 Sapur.003G109000 Sapur.003G130700 Sapur.003G142300 Sapur.003G156600 Sapur.004G019000 Sapur.004G051000 Sapur.004G079500 Sapur.004G088800 Sapur.004G096100 Sapur.004G139700 Sapur.004G144500 Sapur.004G152000 Sapur.004G162100 Sapur.004G175300 Sapur.005G007700 Sapur.005G019400 Sapur.005G020600 Sapur.005G026800 Sapur.005G026900 Sapur.005G028900 Sapur.005G040700 Sapur.005G054500 Sapur.005G103700 Sapur.005G119500 Sapur.005G205500 Sapur.006G004800 Sapur.006G014400 Sapur.006G021100 Sapur.006G035300 Sapur.006G041400 Sapur.006G045100 Sapur.006G045200 Sapur.006G069400 Sapur.006G098500 Sapur.006G116900 Sapur.006G174900 Sapur.006G181200 Sapur.006G186800 Sapur.006G194000 Sapur.006G224700 Sapur.007G031300 Sapur.007G078000 Sapur.007G080400 Sapur.007G080700 Sapur.007G080800 Sapur.007G103200 Sapur.008G004200 Sapur.008G009100 Sapur.008G032900 Sapur.008G054800 Sapur.008G082500 Sapur.008G116400 Sapur.008G135800 Sapur.008G143900 Sapur.008G166400 Sapur.009G022800 Sapur.009G030600 Sapur.009G048400 Sapur.009G053600 Sapur.009G085400 Sapur.009G085600 Sapur.009G085700 Sapur.009G086400 Sapur.009G107600 Sapur.009G120800 Sapur.009G128000 Sapur.010G004000

Sapur.010G015700 Sapur.010G021800 Sapur.010G022000 Sapur.010G079400 Sapur.010G086800 Sapur.010G088800
Sapur.010G088900 Sapur.010G109100 Sapur.010G109200 Sapur.010G142500 Sapur.010G147200 Sapur.010G149000
Sapur.010G165900 Sapur.010G186700 Sapur.010G206100 Sapur.011G018600 Sapur.011G027800 Sapur.011G066700
Sapur.011G097000 Sapur.012G003700 Sapur.012G013200 Sapur.012G021100 Sapur.012G043000 Sapur.012G066500
Sapur.013G018100 Sapur.013G051600 Sapur.013G056200 Sapur.013G080500 Sapur.013G084400 Sapur.014G011100
Sapur.014G011200 Sapur.014G011900 Sapur.014G019000 Sapur.014G019100 Sapur.014G019200 Sapur.014G020000
Sapur.014G020100 Sapur.014G020300 Sapur.014G020400 Sapur.014G027000 Sapur.014G027100 Sapur.014G027200
Sapur.014G027400 Sapur.014G027800 Sapur.014G028400 Sapur.014G028500 Sapur.014G029000 Sapur.014G080900
Sapur.014G104200 Sapur.014G126900 Sapur.016G007300 Sapur.016G007700 Sapur.016G007900 Sapur.016G023300
Sapur.016G028200 Sapur.016G038600 Sapur.016G040200 Sapur.016G050900 Sapur.016G074300 Sapur.016G087600
Sapur.016G132500 Sapur.016G161800 Sapur.016G205100 Sapur.016G209200 Sapur.016G211300 Sapur.016G285400
Sapur.016G285500 Sapur.016G291800 Sapur.017G120700 Sapur.017G121300 Sapur.017G121600 Sapur.017G121700
Sapur.017G122100 Sapur.017G123100 Sapur.018G005400 Sapur.018G050900 Sapur.018G092500 Sapur.018G106800
Sapur.019G065400 Sapur.019G095300 Sapur.15ZG002800 Sapur.15ZG078500 Sapur.T032000 Sapur.T045600
Sapur.T047000 Sapur.T073900 Sapur.T079400 Sapur.T079500 Sapur.T080000 Sapur.T131000 Sapur.001G023800
Sapur.001G029800 Sapur.001G048800 Sapur.001G055600 Sapur.001G058300 Sapur.001G058800 Sapur.001G068000
Sapur.001G072400 Sapur.001G078400 Sapur.001G078600 Sapur.001G084200 Sapur.001G094200 Sapur.001G103000
Sapur.001G108900 Sapur.001G110300 Sapur.001G122200 Sapur.001G135100 Sapur.001G137000 Sapur.001G138100
Sapur.001G151600 Sapur.001G167000 Sapur.001G180200 Sapur.001G181600 Sapur.001G189000 Sapur.001G191700
Sapur.002G004200 Sapur.002G005300 Sapur.002G012000 Sapur.002G013600 Sapur.002G014300 Sapur.002G020900
Sapur.002G023300 Sapur.002G024400 Sapur.002G031600 Sapur.002G032100 Sapur.002G051400 Sapur.002G056700
Sapur.002G061600 Sapur.002G061700 Sapur.002G064800 Sapur.002G068500 Sapur.002G069500 Sapur.002G071000
Sapur.002G085200 Sapur.002G085900 Sapur.002G089300 Sapur.002G090300 Sapur.002G109500 Sapur.002G115000
Sapur.002G128300 Sapur.002G130500 Sapur.002G132300 Sapur.002G132500 Sapur.002G145300 Sapur.002G156100
Sapur.002G161200 Sapur.002G169300 Sapur.002G176300 Sapur.002G191900 Sapur.002G194100 Sapur.002G198400
Sapur.003G002000 Sapur.003G002100 Sapur.003G014300 Sapur.003G018100 Sapur.003G025000 Sapur.003G031900
Sapur.003G033600 Sapur.003G056300 Sapur.003G060500 Sapur.003G066000 Sapur.003G067100 Sapur.003G068400
Sapur.003G078600 Sapur.003G092400 Sapur.003G099500 Sapur.003G114500 Sapur.003G116100 Sapur.003G124900
Sapur.003G125800 Sapur.003G138500 Sapur.003G139700 Sapur.003G141900 Sapur.003G155400 Sapur.003G160700
Sapur.003G168000 Sapur.004G014400 Sapur.004G018300 Sapur.004G019100 Sapur.004G023100 Sapur.004G027800
Sapur.004G033200 Sapur.004G037400 Sapur.004G041000 Sapur.004G051400 Sapur.004G063300 Sapur.004G077500
Sapur.004G077700 Sapur.004G103000 Sapur.004G109700 Sapur.004G121500 Sapur.004G128600 Sapur.004G131500
Sapur.004G137600 Sapur.004G140300 Sapur.004G146400 Sapur.004G147000 Sapur.004G147900 Sapur.004G169900

Sapur.004G172100	Sapur.005G000100	Sapur.005G005000	Sapur.005G005700	Sapur.005G007100	Sapur.005G011200
Sapur.005G022300	Sapur.005G023100	Sapur.005G024800	Sapur.005G025000	Sapur.005G025400	Sapur.005G031700
Sapur.005G038400	Sapur.005G043600	Sapur.005G043700	Sapur.005G046200	Sapur.005G048900	Sapur.005G050200
Sapur.005G055500	Sapur.005G069000	Sapur.005G075800	Sapur.005G085100	Sapur.005G090600	Sapur.005G095700
Sapur.005G096300	Sapur.005G096400	Sapur.005G098100	Sapur.005G109500	Sapur.005G113600	Sapur.005G141400
Sapur.005G145700	Sapur.005G165600	Sapur.005G166000	Sapur.005G187300	Sapur.005G193700	Sapur.005G199500
Sapur.005G204200	Sapur.005G206400	Sapur.006G010200	Sapur.006G018700	Sapur.006G021400	Sapur.006G025100
Sapur.006G036800	Sapur.006G037200	Sapur.006G043000	Sapur.006G067400	Sapur.006G069500	Sapur.006G072700
Sapur.006G084800	Sapur.006G094200	Sapur.006G094300	Sapur.006G100100	Sapur.006G106100	Sapur.006G110200
Sapur.006G112500	Sapur.006G118100	Sapur.006G118200	Sapur.006G126200	Sapur.006G136200	Sapur.006G136800
Sapur.006G138300	Sapur.006G139300	Sapur.006G140600	Sapur.006G154200	Sapur.006G154300	Sapur.006G163000
Sapur.006G163500	Sapur.006G172300	Sapur.006G173100	Sapur.006G177100	Sapur.006G178000	Sapur.006G181100
Sapur.006G182100	Sapur.006G194600	Sapur.006G196800	Sapur.006G198000	Sapur.006G203200	Sapur.006G208000
Sapur.006G218800	Sapur.006G221300	Sapur.006G223700	Sapur.006G224200	Sapur.006G225500	Sapur.007G002900
Sapur.007G007000	Sapur.007G007100	Sapur.007G008900	Sapur.007G012800	Sapur.007G016800	Sapur.007G020200
Sapur.007G021900	Sapur.007G029800	Sapur.007G036500	Sapur.007G044500	Sapur.007G050900	Sapur.007G069600
Sapur.007G071200	Sapur.007G073200	Sapur.007G075800	Sapur.007G077000	Sapur.007G077600	Sapur.007G087100
Sapur.007G088300	Sapur.007G091900	Sapur.007G111300	Sapur.007G111400	Sapur.007G111700	Sapur.007G112200
Sapur.007G115600	Sapur.008G004700	Sapur.008G004900	Sapur.008G022900	Sapur.008G026800	Sapur.008G045900
Sapur.008G046000	Sapur.008G058400	Sapur.008G060600	Sapur.008G062600	Sapur.008G065300	Sapur.008G066900
Sapur.008G077900	Sapur.008G081500	Sapur.008G088300	Sapur.008G090300	Sapur.008G091700	Sapur.008G097000
Sapur.008G115400	Sapur.008G120600	Sapur.008G128300	Sapur.008G131900	Sapur.008G137200	Sapur.008G151300
Sapur.008G151900	Sapur.008G156200	Sapur.008G162000	Sapur.009G002700	Sapur.009G002900	Sapur.009G004200
Sapur.009G005200	Sapur.009G008500	Sapur.009G010000	Sapur.009G018000	Sapur.009G021900	Sapur.009G025800
Sapur.009G041000	Sapur.009G045200	Sapur.009G051100	Sapur.009G057000	Sapur.009G064100	Sapur.009G064400
Sapur.009G066300	Sapur.009G073500	Sapur.009G089000	Sapur.009G094100	Sapur.009G094200	Sapur.009G097900
Sapur.009G098700	Sapur.009G099800	Sapur.009G104900	Sapur.009G112500	Sapur.009G114600	Sapur.009G116000
Sapur.009G117400	Sapur.009G120100	Sapur.009G122200	Sapur.010G001900	Sapur.010G009400	Sapur.010G009500
Sapur.010G013000	Sapur.010G014000	Sapur.010G023400	Sapur.010G024600	Sapur.010G026800	Sapur.010G042700
Sapur.010G042900	Sapur.010G046700	Sapur.010G052700	Sapur.010G053300	Sapur.010G055900	Sapur.010G057900
Sapur.010G060700	Sapur.010G070700	Sapur.010G075900	Sapur.010G086100	Sapur.010G087600	Sapur.010G089800
Sapur.010G090000	Sapur.010G092300	Sapur.010G092700	Sapur.010G093900	Sapur.010G100000	Sapur.010G102500
Sapur.010G104500	Sapur.010G109700	Sapur.010G118700	Sapur.010G119700	Sapur.010G119800	Sapur.010G137200

	Sapur.010G138200	Sapur.010G138400	Sapur.010G138500	Sapur.010G141100	Sapur.010G143600	Sapur.010G152700
	Sapur.010G154500	Sapur.010G157400	Sapur.010G163200	Sapur.010G175000	Sapur.010G187200	Sapur.010G193600
	Sapur.011G008400	Sapur.011G013300	Sapur.011G018500	Sapur.011G028400	Sapur.011G031900	Sapur.011G034200
	Sapur.011G062300	Sapur.011G063500	Sapur.011G071900	Sapur.011G073000	Sapur.011G088900	Sapur.011G102200
	Sapur.011G103500	Sapur.011G103700	Sapur.011G103800	Sapur.011G109700	Sapur.011G109800	Sapur.011G120500
	Sapur.012G000700	Sapur.012G031100	Sapur.012G037400	Sapur.012G040700	Sapur.012G048900	Sapur.012G050600
	Sapur.012G061900	Sapur.012G067300	Sapur.012G067900	Sapur.012G070700	Sapur.012G071400	Sapur.012G073300
	Sapur.012G074000	Sapur.012G074300	Sapur.012G079600	Sapur.012G084200	Sapur.012G086700	Sapur.012G087900
	Sapur.012G091400	Sapur.012G091800	Sapur.012G092600	Sapur.012G092900	Sapur.012G100600	Sapur.012G101100
	Sapur.012G103800	Sapur.012G103900	Sapur.012G104300	Sapur.013G000100	Sapur.013G021200	Sapur.013G025600
	Sapur.013G035500	Sapur.013G045400	Sapur.013G055500	Sapur.013G055900	Sapur.013G084100	Sapur.013G097100
	Sapur.013G100200	Sapur.013G110800	Sapur.013G117900	Sapur.013G118200	Sapur.013G118400	Sapur.013G120100
	Sapur.013G120600	Sapur.013G123000	Sapur.013G123300	Sapur.013G123700	Sapur.013G124400	Sapur.013G134100
	Sapur.013G134200	Sapur.014G001300	Sapur.014G001700	Sapur.014G002600	Sapur.014G003400	Sapur.014G005100
	Sapur.014G025600	Sapur.014G032900	Sapur.014G036100	Sapur.014G040600	Sapur.014G052300	Sapur.014G053000
	Sapur.014G061800	Sapur.014G070700	Sapur.014G073900	Sapur.014G076200	Sapur.014G080400	Sapur.014G084500
	Sapur.014G088900	Sapur.014G090200	Sapur.014G090300	Sapur.014G099500	Sapur.014G104700	Sapur.014G106600
	Sapur.014G107900	Sapur.014G110600	Sapur.014G113000	Sapur.014G113100	Sapur.014G113600	Sapur.014G117400
	Sapur.014G117700	Sapur.014G120400	Sapur.014G132000	Sapur.014G134700	Sapur.016G001600	Sapur.016G009300
	Sapur.016G018900	Sapur.016G031400	Sapur.016G035600	Sapur.016G051900	Sapur.016G052700	Sapur.016G058000
	Sapur.016G060400	Sapur.016G071200	Sapur.016G071600	Sapur.016G077600	Sapur.016G104800	Sapur.016G107800
	Sapur.016G120800	Sapur.016G123500	Sapur.016G125300	Sapur.016G132300	Sapur.016G132400	Sapur.016G141300
	Sapur.016G147900	Sapur.016G150200	Sapur.016G152700	Sapur.016G163400	Sapur.016G184500	Sapur.016G187000
	Sapur.016G189500	Sapur.016G189900	Sapur.016G200900	Sapur.016G201800	Sapur.016G216200	Sapur.016G228700
	Sapur.016G230700	Sapur.016G230800	Sapur.016G243200	Sapur.016G243400	Sapur.016G246600	Sapur.016G247200
	Sapur.016G254800	Sapur.016G262400	Sapur.016G264800	Sapur.016G267000	Sapur.016G281900	Sapur.016G282700
	Sapur.016G288500	Sapur.016G293000	Sapur.016G302100	Sapur.016G302500	Sapur.016G311100	Sapur.017G001400
	Sapur.017G003500	Sapur.017G004800	Sapur.017G009300	Sapur.017G010500	Sapur.017G018700	Sapur.017G020600
	Sapur.017G023600	Sapur.017G023800	Sapur.017G029100	Sapur.017G029300	Sapur.017G036400	Sapur.017G036800
	Sapur.017G037700	Sapur.017G052400	Sapur.017G055300	Sapur.017G057300	Sapur.017G057900	Sapur.017G074500
	Sapur.017G100600	Sapur.017G101100	Sapur.017G101200	Sapur.017G101400	Sapur.017G102500	Sapur.017G104800
	Sapur.017G108100	Sapur.017G108200	Sapur.017G108700	Sapur.017G110500	Sapur.017G110700	Sapur.017G111200
	Sapur.017G112200	Sapur.017G112400	Sapur.017G113400	Sapur.017G119200	Sapur.017G123300	Sapur.017G124700

				Sapur.017G129000 Sapur.018G002400 Sapur.018G003500 Sapur.018G003800 Sapur.018G004300 Sapur.018G008500 Sapur.018G015000 Sapur.018G015800 Sapur.018G016300 Sapur.018G016500 Sapur.018G016600 Sapur.018G016800 Sapur.018G017000 Sapur.018G023200 Sapur.018G025100 Sapur.018G025700 Sapur.018G035300 Sapur.018G045400 Sapur.018G047200 Sapur.018G047900 Sapur.018G057100 Sapur.018G057600 Sapur.018G060400 Sapur.018G063900 Sapur.018G067200 Sapur.018G072600 Sapur.018G074200 Sapur.018G074300 Sapur.018G077200 Sapur.018G087600 Sapur.018G088200 Sapur.018G089300 Sapur.018G105800 Sapur.018G114900 Sapur.018G115800 Sapur.018G115900 Sapur.018G117400 Sapur.019G001100 Sapur.019G003100 Sapur.019G005200 Sapur.019G005900 Sapur.019G007100 Sapur.019G007800 Sapur.019G007900 Sapur.019G009200 Sapur.019G009300 Sapur.019G009500 Sapur.019G009600 Sapur.019G010500 Sapur.019G010600 Sapur.019G010700 Sapur.019G011000 Sapur.019G011100 Sapur.019G011200 Sapur.019G011500 Sapur.019G011600 Sapur.019G011700 Sapur.019G011800 Sapur.019G011900 Sapur.019G012000 Sapur.019G012100 Sapur.019G012400 Sapur.019G012500 Sapur.019G012700 Sapur.019G012800 Sapur.019G013700 Sapur.019G015500 Sapur.019G016400 Sapur.019G017000 Sapur.019G020700 Sapur.019G022800 Sapur.019G023100 Sapur.019G027100 Sapur.019G027300 Sapur.019G027700 Sapur.019G028700 Sapur.019G029100 Sapur.019G034300 Sapur.019G041400 Sapur.019G042100 Sapur.019G042400 Sapur.019G042500 Sapur.019G042900 Sapur.019G043100 Sapur.019G043200 Sapur.019G044000 Sapur.019G044100 Sapur.019G046700 Sapur.019G048000 Sapur.019G048800 Sapur.019G053500 Sapur.019G058700 Sapur.019G060100 Sapur.019G061100 Sapur.019G070000 Sapur.019G075900 Sapur.019G080700 Sapur.019G082400 Sapur.019G084800 Sapur.019G084900 Sapur.019G091100 Sapur.019G091300 Sapur.019G091700 Sapur.019G092300 Sapur.019G092600 Sapur.019G092700 Sapur.019G092800 Sapur.019G093100 Sapur.019G093400 Sapur.019G093600 Sapur.019G093900 Sapur.019G094300 Sapur.019G106100 Sapur.019G107300 Sapur.019G110800 Sapur.019G111400 Sapur.019G113800 Sapur.15WG036700 Sapur.15WG040400 Sapur.15WG043400 Sapur.15WG044400 Sapur.15WG063800 Sapur.15WG076100 Sapur.15ZG018400 Sapur.15ZG038900 Sapur.15ZG044100 Sapur.15ZG044300 Sapur.15ZG044900 Sapur.15ZG049500 Sapur.15ZG050400 Sapur.15ZG050500 Sapur.15ZG068500 Sapur.15ZG073500 Sapur.15ZG078300 Sapur.15ZG095900 Sapur.15ZG099200 Sapur.15ZG103200 Sapur.15ZG109900 Sapur.15ZG116100 Sapur.15ZG120200 Sapur.15ZG123100 Sapur.15ZG123200 Sapur.15ZG126400 Sapur.T003900 Sapur.T005400 Sapur.T005500 Sapur.T028300 Sapur.T029400 Sapur.T030800 Sapur.T046000 Sapur.T051400 Sapur.T058800 Sapur.T080500 Sapur.T096600 Sapur.T115500 Sapur.T116500 Sapur.T117000 Sapur.T131900 Sapur.T133700 Sapur.T147200 Sapur.T150000 Sapur.T175200 Sapur.T191500 Sapur.T192100 Sapur.002G081000 Sapur.002G188700 Sapur.005G201100 Sapur.008G143800
GO:0032553	487	2442	ribonucleotide binding	Sapur.001G023800 Sapur.001G029800 Sapur.001G055600 Sapur.001G058300 Sapur.001G058800 Sapur.001G078400 Sapur.001G078600 Sapur.001G084200 Sapur.001G094200 Sapur.001G103000 Sapur.001G108900 Sapur.001G110300 Sapur.001G122200 Sapur.001G137000 Sapur.001G151600 Sapur.001G167000 Sapur.001G181600 Sapur.001G189000 Sapur.002G005300 Sapur.002G013600 Sapur.002G014300 Sapur.002G023300 Sapur.002G024400 Sapur.002G032100 Sapur.002G051400 Sapur.002G061600 Sapur.002G061700 Sapur.002G064800 Sapur.002G085200

Sapur.002G085900 Sapur.002G089300 Sapur.002G090300 Sapur.002G109500 Sapur.002G130500 Sapur.002G132500
Sapur.002G145300 Sapur.002G156100 Sapur.002G161200 Sapur.002G169300 Sapur.002G191900 Sapur.002G194100
Sapur.003G002000 Sapur.003G002100 Sapur.003G014300 Sapur.003G018100 Sapur.003G025000 Sapur.003G031900
Sapur.003G066000 Sapur.003G067100 Sapur.003G078600 Sapur.003G092400 Sapur.003G099500 Sapur.003G116100
Sapur.003G125800 Sapur.003G138500 Sapur.003G139700 Sapur.003G155400 Sapur.003G160700 Sapur.003G168000
Sapur.004G014400 Sapur.004G019100 Sapur.004G027800 Sapur.004G033200 Sapur.004G041000 Sapur.004G051400
Sapur.004G063300 Sapur.004G077700 Sapur.004G103000 Sapur.004G109700 Sapur.004G128600 Sapur.004G131500
Sapur.004G137600 Sapur.004G140300 Sapur.004G146400 Sapur.004G147000 Sapur.004G147900 Sapur.004G172100
Sapur.005G000100 Sapur.005G005000 Sapur.005G005700 Sapur.005G007100 Sapur.005G011200 Sapur.005G022300
Sapur.005G023100 Sapur.005G024800 Sapur.005G025000 Sapur.005G025400 Sapur.005G031700 Sapur.005G038400
Sapur.005G043600 Sapur.005G043700 Sapur.005G048900 Sapur.005G054500 Sapur.005G055500 Sapur.005G069000
Sapur.005G085100 Sapur.005G095700 Sapur.005G096300 Sapur.005G096400 Sapur.005G109500 Sapur.005G165600
Sapur.005G193700 Sapur.005G199500 Sapur.005G206400 Sapur.006G010200 Sapur.006G018700 Sapur.006G025100
Sapur.006G036800 Sapur.006G037200 Sapur.006G067400 Sapur.006G069500 Sapur.006G072700 Sapur.006G094200
Sapur.006G094300 Sapur.006G110200 Sapur.006G112500 Sapur.006G118100 Sapur.006G118200 Sapur.006G136200
Sapur.006G136800 Sapur.006G138300 Sapur.006G154200 Sapur.006G154300 Sapur.006G163000 Sapur.006G172300
Sapur.006G173100 Sapur.006G181100 Sapur.006G194600 Sapur.006G218800 Sapur.006G221300 Sapur.006G223700
Sapur.006G224200 Sapur.006G225500 Sapur.007G002900 Sapur.007G007000 Sapur.007G007100 Sapur.007G008900
Sapur.007G016800 Sapur.007G029800 Sapur.007G036500 Sapur.007G069600 Sapur.007G071200 Sapur.007G075800
Sapur.007G077000 Sapur.007G077600 Sapur.007G087100 Sapur.007G091900 Sapur.007G111300 Sapur.007G111400
Sapur.007G111700 Sapur.007G112200 Sapur.007G115600 Sapur.008G004700 Sapur.008G009100 Sapur.008G022900
Sapur.008G026800 Sapur.008G045900 Sapur.008G046000 Sapur.008G058400 Sapur.008G060600 Sapur.008G066900
Sapur.008G077900 Sapur.008G081500 Sapur.008G088300 Sapur.008G090300 Sapur.008G091700 Sapur.008G115400
Sapur.008G128300 Sapur.008G131900 Sapur.008G137200 Sapur.008G151300 Sapur.008G151900 Sapur.008G156200
Sapur.008G162000 Sapur.009G002900 Sapur.009G004200 Sapur.009G005200 Sapur.009G018000 Sapur.009G041000
Sapur.009G045200 Sapur.009G051100 Sapur.009G057000 Sapur.009G064100 Sapur.009G066300 Sapur.009G094100
Sapur.009G094200 Sapur.009G112500 Sapur.009G116000 Sapur.009G117400 Sapur.009G120100 Sapur.009G122200
Sapur.010G001900 Sapur.010G009400 Sapur.010G009500 Sapur.010G014000 Sapur.010G024600 Sapur.010G026800
Sapur.010G042700 Sapur.010G046700 Sapur.010G052700 Sapur.010G053300 Sapur.010G055900 Sapur.010G057900
Sapur.010G060700 Sapur.010G070700 Sapur.010G086100 Sapur.010G087600 Sapur.010G089800 Sapur.010G092300
Sapur.010G092700 Sapur.010G093900 Sapur.010G100000 Sapur.010G102500 Sapur.010G104500 Sapur.010G118700
Sapur.010G119700 Sapur.010G119800 Sapur.010G137200 Sapur.010G138500 Sapur.010G141100 Sapur.010G143600
Sapur.010G157400 Sapur.010G163200 Sapur.010G187200 Sapur.010G193600 Sapur.011G008400 Sapur.011G013300

Sapur.011G018500	Sapur.011G028400	Sapur.011G031900	Sapur.011G034200	Sapur.011G073000	Sapur.011G088900
Sapur.011G103500	Sapur.011G103700	Sapur.011G103800	Sapur.012G000700	Sapur.012G037400	Sapur.012G040700
Sapur.012G048900	Sapur.012G050600	Sapur.012G061900	Sapur.012G067300	Sapur.012G070700	Sapur.012G073300
Sapur.012G084200	Sapur.012G086700	Sapur.012G087900	Sapur.012G091400	Sapur.012G091800	Sapur.012G092600
Sapur.012G092900	Sapur.012G100600	Sapur.012G101100	Sapur.013G000100	Sapur.013G021200	Sapur.013G045400
Sapur.013G055900	Sapur.013G097100	Sapur.013G100200	Sapur.013G110800	Sapur.013G117900	Sapur.013G118200
Sapur.013G118400	Sapur.013G120100	Sapur.013G120600	Sapur.013G123000	Sapur.013G123300	Sapur.013G123700
Sapur.013G124400	Sapur.014G001300	Sapur.014G001700	Sapur.014G002600	Sapur.014G005100	Sapur.014G025600
Sapur.014G032900	Sapur.014G036100	Sapur.014G052300	Sapur.014G053000	Sapur.014G061800	Sapur.014G073900
Sapur.014G076200	Sapur.014G080400	Sapur.014G084500	Sapur.014G090200	Sapur.014G090300	Sapur.014G104700
Sapur.014G106600	Sapur.014G110600	Sapur.014G113100	Sapur.014G113600	Sapur.014G117400	Sapur.014G117700
Sapur.014G120400	Sapur.014G132000	Sapur.014G134700	Sapur.016G009300	Sapur.016G018900	Sapur.016G031400
Sapur.016G035600	Sapur.016G051900	Sapur.016G052700	Sapur.016G058000	Sapur.016G071200	Sapur.016G077600
Sapur.016G120800	Sapur.016G125300	Sapur.016G132300	Sapur.016G132400	Sapur.016G141300	Sapur.016G147900
Sapur.016G150200	Sapur.016G163400	Sapur.016G189500	Sapur.016G189900	Sapur.016G200900	Sapur.016G230700
Sapur.016G230800	Sapur.016G243200	Sapur.016G243400	Sapur.016G247200	Sapur.016G262400	Sapur.016G264800
Sapur.016G281900	Sapur.016G282700	Sapur.016G288500	Sapur.016G302100	Sapur.016G302500	Sapur.017G003500
Sapur.017G009300	Sapur.017G010500	Sapur.017G018700	Sapur.017G020600	Sapur.017G023600	Sapur.017G023800
Sapur.017G029100	Sapur.017G036400	Sapur.017G036800	Sapur.017G037700	Sapur.017G052400	Sapur.017G055300
Sapur.017G057300	Sapur.017G057900	Sapur.017G074500	Sapur.017G100600	Sapur.017G101100	Sapur.017G101200
Sapur.017G102500	Sapur.017G104800	Sapur.017G108100	Sapur.017G108200	Sapur.017G108700	Sapur.017G110500
Sapur.017G110700	Sapur.017G111200	Sapur.017G112200	Sapur.017G112400	Sapur.017G113400	Sapur.017G123300
Sapur.017G129000	Sapur.018G002400	Sapur.018G003500	Sapur.018G003800	Sapur.018G004300	Sapur.018G008500
Sapur.018G015000	Sapur.018G015800	Sapur.018G016300	Sapur.018G016500	Sapur.018G016600	Sapur.018G016800
Sapur.018G017000	Sapur.018G023200	Sapur.018G025700	Sapur.018G045400	Sapur.018G047200	Sapur.018G057100
Sapur.018G057600	Sapur.018G060400	Sapur.018G063900	Sapur.018G067200	Sapur.018G077200	Sapur.018G087600
Sapur.018G088200	Sapur.018G105800	Sapur.018G114900	Sapur.018G115800	Sapur.018G115900	Sapur.018G117400
Sapur.019G001100	Sapur.019G003100	Sapur.019G005200	Sapur.019G005900	Sapur.019G007100	Sapur.019G007800
Sapur.019G007900	Sapur.019G009300	Sapur.019G009600	Sapur.019G010500	Sapur.019G010700	Sapur.019G011000
Sapur.019G011200	Sapur.019G011500	Sapur.019G011600	Sapur.019G011700	Sapur.019G011900	Sapur.019G012100
Sapur.019G012500	Sapur.019G012700	Sapur.019G012800	Sapur.019G013700	Sapur.019G016400	Sapur.019G017000
Sapur.019G022800	Sapur.019G023100	Sapur.019G027100	Sapur.019G027300	Sapur.019G028700	Sapur.019G029100
Sapur.019G034300	Sapur.019G042100	Sapur.019G042400	Sapur.019G042500	Sapur.019G043100	Sapur.019G043200

GO:0017076	500	2516	purine nucleotide binding	Sapur.019G044000 Sapur.019G044100 Sapur.019G046700 Sapur.019G048800 Sapur.019G053500 Sapur.019G058700 Sapur.019G075900 Sapur.019G080700 Sapur.019G082400 Sapur.019G091100 Sapur.019G091300 Sapur.019G091700 Sapur.019G092300 Sapur.019G092600 Sapur.019G092700 Sapur.019G092800 Sapur.019G093100 Sapur.019G093400 Sapur.019G093600 Sapur.019G093900 Sapur.019G094300 Sapur.019G107300 Sapur.019G110800 Sapur.019G111400 Sapur.019G113800 Sapur.15WG036700 Sapur.15WG040400 Sapur.15WG043400 Sapur.15WG044400 Sapur.15WG076100 Sapur.15ZG018400 Sapur.15ZG038900 Sapur.15ZG044100 Sapur.15ZG044300 Sapur.15ZG044900 Sapur.15ZG049500 Sapur.15ZG050400 Sapur.15ZG050500 Sapur.15ZG103200 Sapur.15ZG109900 Sapur.15ZG116100 Sapur.15ZG120200 Sapur.15ZG123200 Sapur.15ZG126400 Sapur.T003900 Sapur.T005400 Sapur.T005500 Sapur.T028300 Sapur.T029400 Sapur.T030800 Sapur.T058800 Sapur.T080500 Sapur.T096600 Sapur.T115500 Sapur.T116500 Sapur.T117000 Sapur.T131900 Sapur.T133700 Sapur.T147200 Sapur.T150000 Sapur.T191500 Sapur.T192100 Sapur.001G017800 Sapur.001G023800 Sapur.001G029800 Sapur.001G055600 Sapur.001G058300 Sapur.001G058800 Sapur.001G078400 Sapur.001G078600 Sapur.001G084200 Sapur.001G094200 Sapur.001G103000 Sapur.001G108900 Sapur.001G110300 Sapur.001G122200 Sapur.001G137000 Sapur.001G151600 Sapur.001G167000 Sapur.001G174900 Sapur.001G181600 Sapur.001G189000 Sapur.002G005300 Sapur.002G013600 Sapur.002G014300 Sapur.002G023300 Sapur.002G024400 Sapur.002G032100 Sapur.002G051400 Sapur.002G061600 Sapur.002G061700 Sapur.002G064800 Sapur.002G085200 Sapur.002G085900 Sapur.002G089300 Sapur.002G090300 Sapur.002G109500 Sapur.002G123400 Sapur.002G130500 Sapur.002G132500 Sapur.002G145300 Sapur.002G156100 Sapur.002G161200 Sapur.002G169300 Sapur.002G191900 Sapur.002G194100 Sapur.003G002000 Sapur.003G002100 Sapur.003G014300 Sapur.003G018100 Sapur.003G025000 Sapur.003G031900 Sapur.003G066000 Sapur.003G067100 Sapur.003G078600 Sapur.003G092400 Sapur.003G099500 Sapur.003G116100 Sapur.003G125800 Sapur.003G138500 Sapur.003G139700 Sapur.003G155400 Sapur.003G160700 Sapur.003G168000 Sapur.004G014400 Sapur.004G019100 Sapur.004G027800 Sapur.004G033200 Sapur.004G041000 Sapur.004G051400 Sapur.004G063300 Sapur.004G077700 Sapur.004G103000 Sapur.004G108600 Sapur.004G109700 Sapur.004G128600 Sapur.004G131500 Sapur.004G132200 Sapur.004G137600 Sapur.004G140300 Sapur.004G146400 Sapur.004G147000 Sapur.004G147900 Sapur.004G172100 Sapur.005G000100 Sapur.005G005000 Sapur.005G005700 Sapur.005G007100 Sapur.005G011200 Sapur.005G022300 Sapur.005G023100 Sapur.005G024800 Sapur.005G025000 Sapur.005G025400 Sapur.005G031700 Sapur.005G038400 Sapur.005G043600 Sapur.005G043700 Sapur.005G048900 Sapur.005G054500 Sapur.005G055500 Sapur.005G069000 Sapur.005G085100 Sapur.005G095700 Sapur.005G096300 Sapur.005G096400 Sapur.005G109500 Sapur.005G113600 Sapur.005G165600 Sapur.005G193700 Sapur.005G199500 Sapur.005G206400 Sapur.006G010200 Sapur.006G018700 Sapur.006G025100 Sapur.006G036800 Sapur.006G037200 Sapur.006G067400 Sapur.006G069500 Sapur.006G072700 Sapur.006G094200 Sapur.006G094300 Sapur.006G110200 Sapur.006G112500 Sapur.006G136200 Sapur.006G136800 Sapur.006G138300 Sapur.006G154200 Sapur.006G154300 Sapur.006G163000 Sapur.006G172300 Sapur.006G173100 Sapur.006G181100 Sapur.006G194600 Sapur.006G218800 Sapur.006G221300 Sapur.006G223700 Sapur.006G224200 Sapur.006G225500

Sapur.007G002900 Sapur.007G007000 Sapur.007G007100 Sapur.007G008900 Sapur.007G016800 Sapur.007G029800
Sapur.007G036500 Sapur.007G069600 Sapur.007G071200 Sapur.007G075800 Sapur.007G077000 Sapur.007G077600
Sapur.007G087100 Sapur.007G091900 Sapur.007G111300 Sapur.007G111400 Sapur.007G111700 Sapur.007G112200
Sapur.007G115600 Sapur.008G004700 Sapur.008G004900 Sapur.008G009100 Sapur.008G022900 Sapur.008G026800
Sapur.008G045900 Sapur.008G046000 Sapur.008G058400 Sapur.008G060600 Sapur.008G066900 Sapur.008G077900
Sapur.008G081500 Sapur.008G088300 Sapur.008G090300 Sapur.008G091700 Sapur.008G115400 Sapur.008G128300
Sapur.008G131900 Sapur.008G137200 Sapur.008G151300 Sapur.008G151900 Sapur.008G156200 Sapur.008G162000
Sapur.009G002900 Sapur.009G004200 Sapur.009G005200 Sapur.009G018000 Sapur.009G041000 Sapur.009G045200
Sapur.009G051100 Sapur.009G057000 Sapur.009G064100 Sapur.009G066300 Sapur.009G094100 Sapur.009G094200
Sapur.009G112500 Sapur.009G116000 Sapur.009G117400 Sapur.009G120100 Sapur.009G122200 Sapur.010G001900
Sapur.010G009400 Sapur.010G009500 Sapur.010G014000 Sapur.010G024600 Sapur.010G026800 Sapur.010G042700
Sapur.010G046700 Sapur.010G052700 Sapur.010G053300 Sapur.010G055900 Sapur.010G057900 Sapur.010G060700
Sapur.010G070700 Sapur.010G086100 Sapur.010G087600 Sapur.010G089800 Sapur.010G092300 Sapur.010G092700
Sapur.010G093900 Sapur.010G100000 Sapur.010G102500 Sapur.010G104500 Sapur.010G118700 Sapur.010G119700
Sapur.010G119800 Sapur.010G137200 Sapur.010G138500 Sapur.010G141100 Sapur.010G143600 Sapur.010G157400
Sapur.010G163200 Sapur.010G187200 Sapur.010G193600 Sapur.011G008400 Sapur.011G013300 Sapur.011G018500
Sapur.011G028400 Sapur.011G031900 Sapur.011G034200 Sapur.011G073000 Sapur.011G088900 Sapur.011G103500
Sapur.011G103700 Sapur.011G103800 Sapur.011G120500 Sapur.012G000700 Sapur.012G037400 Sapur.012G040700
Sapur.012G048900 Sapur.012G050600 Sapur.012G061900 Sapur.012G067300 Sapur.012G070700 Sapur.012G073300
Sapur.012G084200 Sapur.012G086700 Sapur.012G087900 Sapur.012G091400 Sapur.012G091800 Sapur.012G092600
Sapur.012G092900 Sapur.012G100600 Sapur.012G101100 Sapur.013G000100 Sapur.013G004800 Sapur.013G021200
Sapur.013G045400 Sapur.013G055900 Sapur.013G100200 Sapur.013G110800 Sapur.013G117900 Sapur.013G118200
Sapur.013G118400 Sapur.013G120100 Sapur.013G120600 Sapur.013G123000 Sapur.013G123300 Sapur.013G123700
Sapur.013G124400 Sapur.014G001300 Sapur.014G001700 Sapur.014G002600 Sapur.014G005100 Sapur.014G025600
Sapur.014G032900 Sapur.014G036100 Sapur.014G052300 Sapur.014G053000 Sapur.014G055200 Sapur.014G055500
Sapur.014G061800 Sapur.014G073900 Sapur.014G076200 Sapur.014G080400 Sapur.014G084500 Sapur.014G090200
Sapur.014G090300 Sapur.014G104700 Sapur.014G106600 Sapur.014G110600 Sapur.014G113100 Sapur.014G113600
Sapur.014G117400 Sapur.014G117700 Sapur.014G120400 Sapur.014G132000 Sapur.014G134700 Sapur.014G135900
Sapur.016G009300 Sapur.016G031400 Sapur.016G035600 Sapur.016G040000 Sapur.016G051900 Sapur.016G052700
Sapur.016G058000 Sapur.016G071200 Sapur.016G077600 Sapur.016G120800 Sapur.016G125300 Sapur.016G132300
Sapur.016G132400 Sapur.016G141300 Sapur.016G147900 Sapur.016G150200 Sapur.016G163400 Sapur.016G189500
Sapur.016G189900 Sapur.016G200900 Sapur.016G230700 Sapur.016G230800 Sapur.016G243200 Sapur.016G243400
Sapur.016G247200 Sapur.016G262400 Sapur.016G264800 Sapur.016G281900 Sapur.016G282700 Sapur.016G288500

				Sapur.016G302100 Sapur.016G302500 Sapur.016G304100 Sapur.017G003500 Sapur.017G009300 Sapur.017G010500 Sapur.017G018700 Sapur.017G020600 Sapur.017G023600 Sapur.017G023800 Sapur.017G029100 Sapur.017G036400 Sapur.017G036800 Sapur.017G037700 Sapur.017G052400 Sapur.017G055300 Sapur.017G057300 Sapur.017G057900 Sapur.017G074500 Sapur.017G100600 Sapur.017G101100 Sapur.017G101200 Sapur.017G102500 Sapur.017G104800 Sapur.017G108100 Sapur.017G108200 Sapur.017G108700 Sapur.017G110500 Sapur.017G110700 Sapur.017G111200 Sapur.017G112200 Sapur.017G112400 Sapur.017G113400 Sapur.017G123300 Sapur.017G129000 Sapur.018G002400 Sapur.018G003500 Sapur.018G003800 Sapur.018G004300 Sapur.018G008500 Sapur.018G015000 Sapur.018G015800 Sapur.018G016300 Sapur.018G016500 Sapur.018G016600 Sapur.018G016800 Sapur.018G017000 Sapur.018G023200 Sapur.018G025700 Sapur.018G045400 Sapur.018G057100 Sapur.018G057600 Sapur.018G060400 Sapur.018G063600 Sapur.018G063900 Sapur.018G067200 Sapur.018G077200 Sapur.018G087600 Sapur.018G088200 Sapur.018G105800 Sapur.018G114900 Sapur.018G115800 Sapur.018G115900 Sapur.018G117400 Sapur.019G001100 Sapur.019G003100 Sapur.019G005200 Sapur.019G005900 Sapur.019G007100 Sapur.019G007800 Sapur.019G007900 Sapur.019G009300 Sapur.019G009600 Sapur.019G010500 Sapur.019G010700 Sapur.019G011000 Sapur.019G011200 Sapur.019G011500 Sapur.019G011600 Sapur.019G011700 Sapur.019G011900 Sapur.019G012100 Sapur.019G012500 Sapur.019G012700 Sapur.019G012800 Sapur.019G013700 Sapur.019G016400 Sapur.019G017000 Sapur.019G022800 Sapur.019G023100 Sapur.019G027100 Sapur.019G027300 Sapur.019G028700 Sapur.019G029100 Sapur.019G034300 Sapur.019G042100 Sapur.019G042400 Sapur.019G042500 Sapur.019G043100 Sapur.019G043200 Sapur.019G044000 Sapur.019G044100 Sapur.019G046700 Sapur.019G048800 Sapur.019G053500 Sapur.019G058700 Sapur.019G070000 Sapur.019G075900 Sapur.019G080700 Sapur.019G082400 Sapur.019G091100 Sapur.019G091300 Sapur.019G091700 Sapur.019G092300 Sapur.019G092600 Sapur.019G092700 Sapur.019G092800 Sapur.019G093100 Sapur.019G093400 Sapur.019G093600 Sapur.019G093900 Sapur.019G094300 Sapur.019G107300 Sapur.019G110800 Sapur.019G111400 Sapur.019G113800 Sapur.15WG030400 Sapur.15WG036700 Sapur.15WG040400 Sapur.15WG043400 Sapur.15WG044400 Sapur.15WG076100 Sapur.15ZG018400 Sapur.15ZG038900 Sapur.15ZG044100 Sapur.15ZG044300 Sapur.15ZG044900 Sapur.15ZG049500 Sapur.15ZG050400 Sapur.15ZG050500 Sapur.15ZG078500 Sapur.15ZG103200 Sapur.15ZG109900 Sapur.15ZG116100 Sapur.15ZG120200 Sapur.15ZG123200 Sapur.15ZG126400 Sapur.T003900 Sapur.T005400 Sapur.T005500 Sapur.T028300 Sapur.T029400 Sapur.T030800 Sapur.T058800 Sapur.T080500 Sapur.T096600 Sapur.T115500 Sapur.T116500 Sapur.T117000 Sapur.T131900 Sapur.T133700 Sapur.T147200 Sapur.T150000 Sapur.T191500 Sapur.T192100
GO:0032555	482	2416	purine ribonucleotide binding	Sapur.001G023800 Sapur.001G029800 Sapur.001G055600 Sapur.001G058300 Sapur.001G058800 Sapur.001G078400 Sapur.001G078600 Sapur.001G084200 Sapur.001G094200 Sapur.001G103000 Sapur.001G108900 Sapur.001G110300 Sapur.001G122200 Sapur.001G137000 Sapur.001G151600 Sapur.001G167000 Sapur.001G181600 Sapur.001G189000 Sapur.002G005300 Sapur.002G013600 Sapur.002G014300 Sapur.002G023300 Sapur.002G024400 Sapur.002G032100 Sapur.002G051400 Sapur.002G061600 Sapur.002G061700 Sapur.002G064800 Sapur.002G085200

Sapur.002G085900	Sapur.002G089300	Sapur.002G090300	Sapur.002G109500	Sapur.002G130500	Sapur.002G132500
Sapur.002G145300	Sapur.002G156100	Sapur.002G161200	Sapur.002G169300	Sapur.002G191900	Sapur.002G194100
Sapur.003G002000	Sapur.003G002100	Sapur.003G014300	Sapur.003G018100	Sapur.003G025000	Sapur.003G031900
Sapur.003G066000	Sapur.003G067100	Sapur.003G078600	Sapur.003G092400	Sapur.003G099500	Sapur.003G116100
Sapur.003G125800	Sapur.003G138500	Sapur.003G139700	Sapur.003G155400	Sapur.003G160700	Sapur.003G168000
Sapur.004G014400	Sapur.004G019100	Sapur.004G027800	Sapur.004G033200	Sapur.004G041000	Sapur.004G051400
Sapur.004G063300	Sapur.004G077700	Sapur.004G103000	Sapur.004G109700	Sapur.004G128600	Sapur.004G131500
Sapur.004G137600	Sapur.004G140300	Sapur.004G146400	Sapur.004G147000	Sapur.004G147900	Sapur.004G172100
Sapur.005G000100	Sapur.005G005000	Sapur.005G005700	Sapur.005G007100	Sapur.005G011200	Sapur.005G022300
Sapur.005G023100	Sapur.005G024800	Sapur.005G025000	Sapur.005G025400	Sapur.005G031700	Sapur.005G038400
Sapur.005G043600	Sapur.005G043700	Sapur.005G048900	Sapur.005G054500	Sapur.005G055500	Sapur.005G069000
Sapur.005G085100	Sapur.005G095700	Sapur.005G096300	Sapur.005G096400	Sapur.005G109500	Sapur.005G165600
Sapur.005G193700	Sapur.005G199500	Sapur.005G206400	Sapur.006G010200	Sapur.006G018700	Sapur.006G025100
Sapur.006G036800	Sapur.006G037200	Sapur.006G067400	Sapur.006G069500	Sapur.006G072700	Sapur.006G094200
Sapur.006G094300	Sapur.006G110200	Sapur.006G112500	Sapur.006G136200	Sapur.006G136800	Sapur.006G138300
Sapur.006G154200	Sapur.006G154300	Sapur.006G163000	Sapur.006G172300	Sapur.006G173100	Sapur.006G181100
Sapur.006G194600	Sapur.006G218800	Sapur.006G221300	Sapur.006G223700	Sapur.006G224200	Sapur.006G225500
Sapur.007G002900	Sapur.007G007000	Sapur.007G007100	Sapur.007G008900	Sapur.007G016800	Sapur.007G029800
Sapur.007G036500	Sapur.007G069600	Sapur.007G071200	Sapur.007G075800	Sapur.007G077000	Sapur.007G077600
Sapur.007G087100	Sapur.007G091900	Sapur.007G111300	Sapur.007G111400	Sapur.007G111700	Sapur.007G112200
Sapur.007G115600	Sapur.008G004700	Sapur.008G009100	Sapur.008G022900	Sapur.008G026800	Sapur.008G045900
Sapur.008G046000	Sapur.008G058400	Sapur.008G060600	Sapur.008G066900	Sapur.008G077900	Sapur.008G081500
Sapur.008G088300	Sapur.008G090300	Sapur.008G091700	Sapur.008G115400	Sapur.008G128300	Sapur.008G131900
Sapur.008G137200	Sapur.008G151300	Sapur.008G151900	Sapur.008G156200	Sapur.008G162000	Sapur.009G002900
Sapur.009G004200	Sapur.009G005200	Sapur.009G018000	Sapur.009G041000	Sapur.009G045200	Sapur.009G051100
Sapur.009G057000	Sapur.009G064100	Sapur.009G066300	Sapur.009G094100	Sapur.009G094200	Sapur.009G112500
Sapur.009G116000	Sapur.009G117400	Sapur.009G120100	Sapur.009G122200	Sapur.010G001900	Sapur.010G009400
Sapur.010G009500	Sapur.010G014000	Sapur.010G024600	Sapur.010G026800	Sapur.010G042700	Sapur.010G046700
Sapur.010G052700	Sapur.010G053300	Sapur.010G055900	Sapur.010G057900	Sapur.010G060700	Sapur.010G070700
Sapur.010G086100	Sapur.010G087600	Sapur.010G089800	Sapur.010G092300	Sapur.010G092700	Sapur.010G093900
Sapur.010G100000	Sapur.010G102500	Sapur.010G104500	Sapur.010G118700	Sapur.010G119700	Sapur.010G119800
Sapur.010G137200	Sapur.010G138500	Sapur.010G141100	Sapur.010G143600	Sapur.010G157400	Sapur.010G163200
Sapur.010G187200	Sapur.010G193600	Sapur.011G008400	Sapur.011G013300	Sapur.011G018500	Sapur.011G028400

Sapur.011G031900	Sapur.011G034200	Sapur.011G073000	Sapur.011G088900	Sapur.011G103500	Sapur.011G103700
Sapur.011G103800	Sapur.012G000700	Sapur.012G037400	Sapur.012G040700	Sapur.012G048900	Sapur.012G050600
Sapur.012G061900	Sapur.012G067300	Sapur.012G070700	Sapur.012G073300	Sapur.012G084200	Sapur.012G086700
Sapur.012G087900	Sapur.012G091400	Sapur.012G091800	Sapur.012G092600	Sapur.012G092900	Sapur.012G100600
Sapur.012G101100	Sapur.013G000100	Sapur.013G021200	Sapur.013G045400	Sapur.013G055900	Sapur.013G100200
Sapur.013G110800	Sapur.013G117900	Sapur.013G118200	Sapur.013G118400	Sapur.013G120100	Sapur.013G120600
Sapur.013G123000	Sapur.013G123300	Sapur.013G123700	Sapur.013G124400	Sapur.014G001300	Sapur.014G001700
Sapur.014G002600	Sapur.014G005100	Sapur.014G025600	Sapur.014G032900	Sapur.014G036100	Sapur.014G052300
Sapur.014G053000	Sapur.014G061800	Sapur.014G073900	Sapur.014G076200	Sapur.014G080400	Sapur.014G084500
Sapur.014G090200	Sapur.014G090300	Sapur.014G104700	Sapur.014G106600	Sapur.014G110600	Sapur.014G113100
Sapur.014G113600	Sapur.014G117400	Sapur.014G117700	Sapur.014G120400	Sapur.014G132000	Sapur.014G134700
Sapur.016G009300	Sapur.016G031400	Sapur.016G035600	Sapur.016G051900	Sapur.016G052700	Sapur.016G058000
Sapur.016G071200	Sapur.016G077600	Sapur.016G120800	Sapur.016G125300	Sapur.016G132300	Sapur.016G132400
Sapur.016G141300	Sapur.016G147900	Sapur.016G150200	Sapur.016G163400	Sapur.016G189500	Sapur.016G189900
Sapur.016G200900	Sapur.016G230700	Sapur.016G230800	Sapur.016G243200	Sapur.016G243400	Sapur.016G247200
Sapur.016G262400	Sapur.016G264800	Sapur.016G281900	Sapur.016G282700	Sapur.016G288500	Sapur.016G302100
Sapur.016G302500	Sapur.017G003500	Sapur.017G009300	Sapur.017G010500	Sapur.017G018700	Sapur.017G020600
Sapur.017G023600	Sapur.017G023800	Sapur.017G029100	Sapur.017G036400	Sapur.017G036800	Sapur.017G037700
Sapur.017G052400	Sapur.017G055300	Sapur.017G057300	Sapur.017G057900	Sapur.017G074500	Sapur.017G100600
Sapur.017G101100	Sapur.017G101200	Sapur.017G102500	Sapur.017G104800	Sapur.017G108100	Sapur.017G108200
Sapur.017G108700	Sapur.017G110500	Sapur.017G110700	Sapur.017G111200	Sapur.017G112200	Sapur.017G112400
Sapur.017G113400	Sapur.017G123300	Sapur.017G129000	Sapur.018G002400	Sapur.018G003500	Sapur.018G003800
Sapur.018G004300	Sapur.018G008500	Sapur.018G015000	Sapur.018G015800	Sapur.018G016300	Sapur.018G016500
Sapur.018G016600	Sapur.018G016800	Sapur.018G017000	Sapur.018G023200	Sapur.018G025700	Sapur.018G045400
Sapur.018G057100	Sapur.018G057600	Sapur.018G060400	Sapur.018G063900	Sapur.018G067200	Sapur.018G077200
Sapur.018G087600	Sapur.018G088200	Sapur.018G105800	Sapur.018G114900	Sapur.018G115800	Sapur.018G115900
Sapur.018G117400	Sapur.019G001100	Sapur.019G003100	Sapur.019G005200	Sapur.019G005900	Sapur.019G007100
Sapur.019G007800	Sapur.019G007900	Sapur.019G009300	Sapur.019G009600	Sapur.019G010500	Sapur.019G010700
Sapur.019G011000	Sapur.019G011200	Sapur.019G011500	Sapur.019G011600	Sapur.019G011700	Sapur.019G011900
Sapur.019G012100	Sapur.019G012500	Sapur.019G012700	Sapur.019G012800	Sapur.019G013700	Sapur.019G016400
Sapur.019G017000	Sapur.019G022800	Sapur.019G023100	Sapur.019G027100	Sapur.019G027300	Sapur.019G028700
Sapur.019G029100	Sapur.019G034300	Sapur.019G042100	Sapur.019G042400	Sapur.019G042500	Sapur.019G043100
Sapur.019G043200	Sapur.019G044000	Sapur.019G044100	Sapur.019G046700	Sapur.019G048800	Sapur.019G053500

				Sapur.019G058700 Sapur.019G075900 Sapur.019G080700 Sapur.019G082400 Sapur.019G091100 Sapur.019G091300 Sapur.019G091700 Sapur.019G092300 Sapur.019G092600 Sapur.019G092700 Sapur.019G092800 Sapur.019G093100 Sapur.019G093400 Sapur.019G093600 Sapur.019G093900 Sapur.019G094300 Sapur.019G107300 Sapur.019G110800 Sapur.019G111400 Sapur.019G113800 Sapur.15WG036700 Sapur.15WG040400 Sapur.15WG043400 Sapur.15WG044400 Sapur.15WG076100 Sapur.15ZG018400 Sapur.15ZG038900 Sapur.15ZG044100 Sapur.15ZG044300 Sapur.15ZG044900 Sapur.15ZG049500 Sapur.15ZG050400 Sapur.15ZG050500 Sapur.15ZG103200 Sapur.15ZG109900 Sapur.15ZG116100 Sapur.15ZG120200 Sapur.15ZG123200 Sapur.15ZG126400 Sapur.T003900 Sapur.T005400 Sapur.T005500 Sapur.T028300 Sapur.T029400 Sapur.T030800 Sapur.T058800 Sapur.T080500 Sapur.T096600 Sapur.T115500 Sapur.T116500 Sapur.T117000 Sapur.T131900 Sapur.T133700 Sapur.T147200 Sapur.T150000 Sapur.T191500 Sapur.T192100
GO:0036094	555	2832	small molecule binding	Sapur.001G017800 Sapur.001G023800 Sapur.001G029800 Sapur.001G048800 Sapur.001G055600 Sapur.001G058300 Sapur.001G058800 Sapur.001G068000 Sapur.001G078400 Sapur.001G078600 Sapur.001G084200 Sapur.001G094200 Sapur.001G103000 Sapur.001G108900 Sapur.001G110300 Sapur.001G122200 Sapur.001G135100 Sapur.001G136400 Sapur.001G137000 Sapur.001G151600 Sapur.001G167000 Sapur.001G174900 Sapur.001G181600 Sapur.001G189000 Sapur.002G005300 Sapur.002G013600 Sapur.002G014300 Sapur.002G023300 Sapur.002G024400 Sapur.002G032100 Sapur.002G036100 Sapur.002G051400 Sapur.002G061600 Sapur.002G061700 Sapur.002G064800 Sapur.002G085200 Sapur.002G085900 Sapur.002G089300 Sapur.002G090300 Sapur.002G109500 Sapur.002G123400 Sapur.002G130500 Sapur.002G132500 Sapur.002G145300 Sapur.002G156100 Sapur.002G161200 Sapur.002G169300 Sapur.002G191900 Sapur.002G194100 Sapur.003G002000 Sapur.003G002100 Sapur.003G014300 Sapur.003G018100 Sapur.003G025000 Sapur.003G031900 Sapur.003G066000 Sapur.003G067100 Sapur.003G078600 Sapur.003G090400 Sapur.003G092400 Sapur.003G099500 Sapur.003G114500 Sapur.003G116100 Sapur.003G124900 Sapur.003G125800 Sapur.003G138500 Sapur.003G139700 Sapur.003G141900 Sapur.003G155400 Sapur.003G156600 Sapur.003G160700 Sapur.003G168000 Sapur.004G014400 Sapur.004G019100 Sapur.004G027800 Sapur.004G033200 Sapur.004G037400 Sapur.004G041000 Sapur.004G051400 Sapur.004G063300 Sapur.004G077700 Sapur.004G103000 Sapur.004G108600 Sapur.004G109700 Sapur.004G128600 Sapur.004G131500 Sapur.004G132200 Sapur.004G137600 Sapur.004G140300 Sapur.004G146400 Sapur.004G147000 Sapur.004G147900 Sapur.004G172100 Sapur.005G000100 Sapur.005G005000 Sapur.005G005700 Sapur.005G007100 Sapur.005G011200 Sapur.005G022300 Sapur.005G023100 Sapur.005G024800 Sapur.005G025000 Sapur.005G025400 Sapur.005G031700 Sapur.005G038400 Sapur.005G040700 Sapur.005G043600 Sapur.005G043700 Sapur.005G046200 Sapur.005G048900 Sapur.005G050200 Sapur.005G054500 Sapur.005G055500 Sapur.005G069000 Sapur.005G075800 Sapur.005G085100 Sapur.005G095700 Sapur.005G096300 Sapur.005G096400 Sapur.005G109500 Sapur.005G113600 Sapur.005G119500 Sapur.005G145700 Sapur.005G165600 Sapur.005G193700 Sapur.005G199500 Sapur.005G206400 Sapur.006G010200 Sapur.006G018700 Sapur.006G025100 Sapur.006G036800 Sapur.006G037200 Sapur.006G067400 Sapur.006G069500 Sapur.006G072700 Sapur.006G094200 Sapur.006G094300

Sapur.006G110200 Sapur.006G112500 Sapur.006G118100 Sapur.006G118200 Sapur.006G126200 Sapur.006G136200
Sapur.006G136800 Sapur.006G138300 Sapur.006G139300 Sapur.006G154200 Sapur.006G154300 Sapur.006G163000
Sapur.006G172300 Sapur.006G173100 Sapur.006G181100 Sapur.006G181200 Sapur.006G182100 Sapur.006G194600
Sapur.006G218800 Sapur.006G221300 Sapur.006G223700 Sapur.006G224200 Sapur.006G224700 Sapur.006G225500
Sapur.007G002900 Sapur.007G007000 Sapur.007G007100 Sapur.007G008900 Sapur.007G016800 Sapur.007G029800
Sapur.007G036500 Sapur.007G044500 Sapur.007G069600 Sapur.007G071200 Sapur.007G075800 Sapur.007G077000
Sapur.007G077600 Sapur.007G087100 Sapur.007G091900 Sapur.007G111300 Sapur.007G111400 Sapur.007G111700
Sapur.007G112200 Sapur.007G115600 Sapur.008G004200 Sapur.008G004700 Sapur.008G004900 Sapur.008G009100
Sapur.008G022900 Sapur.008G026800 Sapur.008G045900 Sapur.008G046000 Sapur.008G058400 Sapur.008G060600
Sapur.008G062600 Sapur.008G066900 Sapur.008G077900 Sapur.008G081500 Sapur.008G088300 Sapur.008G090300
Sapur.008G091700 Sapur.008G115400 Sapur.008G128300 Sapur.008G131900 Sapur.008G137200 Sapur.008G151300
Sapur.008G151900 Sapur.008G156200 Sapur.008G162000 Sapur.009G002900 Sapur.009G004200 Sapur.009G005200
Sapur.009G018000 Sapur.009G041000 Sapur.009G045200 Sapur.009G051100 Sapur.009G057000 Sapur.009G064100
Sapur.009G064400 Sapur.009G066300 Sapur.009G087200 Sapur.009G094100 Sapur.009G094200 Sapur.009G099800
Sapur.009G112500 Sapur.009G116000 Sapur.009G117400 Sapur.009G120100 Sapur.009G122200 Sapur.010G001900
Sapur.010G009400 Sapur.010G009500 Sapur.010G014000 Sapur.010G024600 Sapur.010G026800 Sapur.010G042700
Sapur.010G042900 Sapur.010G046700 Sapur.010G052700 Sapur.010G053300 Sapur.010G055900 Sapur.010G057900
Sapur.010G060700 Sapur.010G070700 Sapur.010G086100 Sapur.010G087600 Sapur.010G089800 Sapur.010G092300
Sapur.010G092700 Sapur.010G093900 Sapur.010G100000 Sapur.010G102500 Sapur.010G104500 Sapur.010G118700
Sapur.010G119700 Sapur.010G119800 Sapur.010G137200 Sapur.010G138500 Sapur.010G141100 Sapur.010G143600
Sapur.010G157400 Sapur.010G163200 Sapur.010G187200 Sapur.010G193600 Sapur.011G008400 Sapur.011G013300
Sapur.011G018500 Sapur.011G028400 Sapur.011G031900 Sapur.011G034200 Sapur.011G073000 Sapur.011G088900
Sapur.011G103500 Sapur.011G103700 Sapur.011G103800 Sapur.011G120500 Sapur.012G000700 Sapur.012G037400
Sapur.012G040700 Sapur.012G048900 Sapur.012G050600 Sapur.012G061900 Sapur.012G067300 Sapur.012G070700
Sapur.012G073300 Sapur.012G074000 Sapur.012G074300 Sapur.012G084200 Sapur.012G086700 Sapur.012G087900
Sapur.012G091400 Sapur.012G091800 Sapur.012G092600 Sapur.012G092900 Sapur.012G100600 Sapur.012G101100
Sapur.012G104300 Sapur.013G000100 Sapur.013G004800 Sapur.013G021200 Sapur.013G045400 Sapur.013G055900
Sapur.013G097100 Sapur.013G100200 Sapur.013G110800 Sapur.013G117900 Sapur.013G118200 Sapur.013G118400
Sapur.013G120100 Sapur.013G120600 Sapur.013G123000 Sapur.013G123300 Sapur.013G123700 Sapur.013G124400
Sapur.014G001300 Sapur.014G001700 Sapur.014G002600 Sapur.014G003400 Sapur.014G005100 Sapur.014G025600
Sapur.014G032900 Sapur.014G036100 Sapur.014G052300 Sapur.014G053000 Sapur.014G055200 Sapur.014G055500
Sapur.014G061800 Sapur.014G073900 Sapur.014G076200 Sapur.014G080400 Sapur.014G084500 Sapur.014G090200
Sapur.014G090300 Sapur.014G104700 Sapur.014G106600 Sapur.014G110600 Sapur.014G113100 Sapur.014G113600

Sapur.014G117400	Sapur.014G117700	Sapur.014G120400	Sapur.014G132000	Sapur.014G134700	Sapur.014G135900
Sapur.016G009300	Sapur.016G018900	Sapur.016G031400	Sapur.016G035600	Sapur.016G040000	Sapur.016G051900
Sapur.016G052700	Sapur.016G058000	Sapur.016G060400	Sapur.016G071200	Sapur.016G077600	Sapur.016G104800
Sapur.016G120800	Sapur.016G123500	Sapur.016G125300	Sapur.016G132300	Sapur.016G132400	Sapur.016G141300
Sapur.016G147900	Sapur.016G150200	Sapur.016G152700	Sapur.016G163400	Sapur.016G189500	Sapur.016G189900
Sapur.016G200900	Sapur.016G201800	Sapur.016G228700	Sapur.016G230700	Sapur.016G230800	Sapur.016G243200
Sapur.016G243400	Sapur.016G247200	Sapur.016G254800	Sapur.016G262400	Sapur.016G264800	Sapur.016G281900
Sapur.016G282700	Sapur.016G288500	Sapur.016G302100	Sapur.016G302500	Sapur.016G304100	Sapur.017G003500
Sapur.017G009300	Sapur.017G010500	Sapur.017G018700	Sapur.017G020600	Sapur.017G023600	Sapur.017G023800
Sapur.017G029100	Sapur.017G036400	Sapur.017G036800	Sapur.017G037700	Sapur.017G052400	Sapur.017G055300
Sapur.017G057300	Sapur.017G057900	Sapur.017G074500	Sapur.017G100600	Sapur.017G101100	Sapur.017G101200
Sapur.017G102500	Sapur.017G104800	Sapur.017G108100	Sapur.017G108200	Sapur.017G108700	Sapur.017G110500
Sapur.017G110700	Sapur.017G111200	Sapur.017G112200	Sapur.017G112400	Sapur.017G113400	Sapur.017G123300
Sapur.017G124700	Sapur.017G129000	Sapur.018G002400	Sapur.018G003500	Sapur.018G003800	Sapur.018G004300
Sapur.018G005400	Sapur.018G008500	Sapur.018G015000	Sapur.018G015800	Sapur.018G016300	Sapur.018G016500
Sapur.018G016600	Sapur.018G016800	Sapur.018G017000	Sapur.018G023200	Sapur.018G025700	Sapur.018G045400
Sapur.018G047200	Sapur.018G057100	Sapur.018G057600	Sapur.018G060400	Sapur.018G063600	Sapur.018G063900
Sapur.018G067200	Sapur.018G077200	Sapur.018G087600	Sapur.018G088200	Sapur.018G105800	Sapur.018G106800
Sapur.018G114900	Sapur.018G115800	Sapur.018G115900	Sapur.018G117400	Sapur.019G001100	Sapur.019G003100
Sapur.019G005200	Sapur.019G005900	Sapur.019G007100	Sapur.019G007800	Sapur.019G007900	Sapur.019G009300
Sapur.019G009600	Sapur.019G010500	Sapur.019G010700	Sapur.019G011000	Sapur.019G011200	Sapur.019G011500
Sapur.019G011600	Sapur.019G011700	Sapur.019G011900	Sapur.019G012100	Sapur.019G012500	Sapur.019G012700
Sapur.019G012800	Sapur.019G013700	Sapur.019G016400	Sapur.019G017000	Sapur.019G022800	Sapur.019G023100
Sapur.019G027100	Sapur.019G027300	Sapur.019G027700	Sapur.019G028700	Sapur.019G029100	Sapur.019G034300
Sapur.019G042100	Sapur.019G042400	Sapur.019G042500	Sapur.019G043100	Sapur.019G043200	Sapur.019G044000
Sapur.019G044100	Sapur.019G046700	Sapur.019G048000	Sapur.019G048800	Sapur.019G053500	Sapur.019G058700
Sapur.019G070000	Sapur.019G075900	Sapur.019G080700	Sapur.019G082400	Sapur.019G091100	Sapur.019G091300
Sapur.019G091700	Sapur.019G092300	Sapur.019G092600	Sapur.019G092700	Sapur.019G092800	Sapur.019G093100
Sapur.019G093400	Sapur.019G093600	Sapur.019G093900	Sapur.019G094300	Sapur.019G107300	Sapur.019G110800
Sapur.019G111400	Sapur.019G113800	Sapur.15WG030400	Sapur.15WG036700	Sapur.15WG040400	Sapur.15WG043400
Sapur.15WG044400	Sapur.15WG076100	Sapur.15ZG018400	Sapur.15ZG038900	Sapur.15ZG044100	Sapur.15ZG044300
Sapur.15ZG044900	Sapur.15ZG049500	Sapur.15ZG050400	Sapur.15ZG050500	Sapur.15ZG078500	Sapur.15ZG099200
Sapur.15ZG103200	Sapur.15ZG109900	Sapur.15ZG116100	Sapur.15ZG120200	Sapur.15ZG123100	Sapur.15ZG123200

				Sapur.15ZG126400 Sapur.T003900 Sapur.T005400 Sapur.T005500 Sapur.T028300 Sapur.T029400 Sapur.T030800 Sapur.T058800 Sapur.T080500 Sapur.T096600 Sapur.T115500 Sapur.T116500 Sapur.T117000 Sapur.T131900 Sapur.T133700 Sapur.T147200 Sapur.T150000 Sapur.T191500 Sapur.T192100 Sapur.001G025400 Sapur.019G101700 Sapur.15ZG002000
GO:1901363	980	5307	heterocyclic compound binding	Sapur.001G013800 Sapur.001G025400 Sapur.001G047800 Sapur.001G055600 Sapur.001G058800 Sapur.001G067700 Sapur.001G067900 Sapur.001G069800 Sapur.001G072500 Sapur.001G090900 Sapur.001G119600 Sapur.001G129000 Sapur.001G137000 Sapur.001G148900 Sapur.001G149000 Sapur.001G149100 Sapur.001G151600 Sapur.001G166100 Sapur.001G167600 Sapur.001G167800 Sapur.001G178100 Sapur.001G179000 Sapur.001G193000 Sapur.002G019400 Sapur.002G021500 Sapur.002G023100 Sapur.002G028500 Sapur.002G031600 Sapur.002G035300 Sapur.002G043300 Sapur.002G048900 Sapur.002G064600 Sapur.002G069500 Sapur.002G076800 Sapur.002G080700 Sapur.002G091700 Sapur.002G106000 Sapur.002G107000 Sapur.002G110600 Sapur.002G116100 Sapur.002G125900 Sapur.002G126000 Sapur.002G136900 Sapur.002G141800 Sapur.002G142300 Sapur.002G145300 Sapur.002G160400 Sapur.003G003700 Sapur.003G016500 Sapur.003G016800 Sapur.003G025800 Sapur.003G037400 Sapur.003G037500 Sapur.003G047900 Sapur.003G057500 Sapur.003G068400 Sapur.003G082800 Sapur.003G093200 Sapur.003G109000 Sapur.003G123800 Sapur.003G129600 Sapur.003G130700 Sapur.003G150500 Sapur.003G152800 Sapur.003G159400 Sapur.003G168000 Sapur.004G030500 Sapur.004G030800 Sapur.004G030900 Sapur.004G034900 Sapur.004G054800 Sapur.004G056900 Sapur.004G062200 Sapur.004G088800 Sapur.004G089500 Sapur.004G096100 Sapur.004G101700 Sapur.004G103000 Sapur.004G110300 Sapur.004G121800 Sapur.004G137000 Sapur.004G139700 Sapur.004G165200 Sapur.005G000100 Sapur.005G005600 Sapur.005G019900 Sapur.005G032200 Sapur.005G052900 Sapur.005G054500 Sapur.005G054800 Sapur.005G055700 Sapur.005G085100 Sapur.005G089700 Sapur.005G093100 Sapur.005G095300 Sapur.005G105500 Sapur.005G115200 Sapur.005G119200 Sapur.005G141300 Sapur.005G158900 Sapur.005G163900 Sapur.005G186700 Sapur.005G205700 Sapur.006G018700 Sapur.006G022000 Sapur.006G026800 Sapur.006G043000 Sapur.006G045100 Sapur.006G045200 Sapur.006G063200 Sapur.006G065100 Sapur.006G068600 Sapur.006G098500 Sapur.006G103400 Sapur.006G104700 Sapur.006G114200 Sapur.006G116900 Sapur.006G118700 Sapur.006G121100 Sapur.006G123200 Sapur.006G134000 Sapur.006G136200 Sapur.006G154200 Sapur.006G165100 Sapur.006G166000 Sapur.006G170000 Sapur.006G186800 Sapur.006G194100 Sapur.006G214400 Sapur.007G034000 Sapur.007G035800 Sapur.007G049700 Sapur.007G058700 Sapur.007G078000 Sapur.007G080700 Sapur.007G080800 Sapur.007G087800 Sapur.007G089800 Sapur.007G091900 Sapur.007G094000 Sapur.007G103200 Sapur.007G108700 Sapur.007G115600 Sapur.008G021500 Sapur.008G042700 Sapur.008G045800 Sapur.008G053100 Sapur.008G060600 Sapur.008G064800 Sapur.008G066900 Sapur.008G069200 Sapur.008G080600 Sapur.008G090400 Sapur.008G093800 Sapur.008G094300 Sapur.008G104300 Sapur.008G118700 Sapur.008G119700 Sapur.008G131800 Sapur.008G140000 Sapur.008G156200 Sapur.008G162200 Sapur.008G166400 Sapur.009G002900 Sapur.009G005300 Sapur.009G009700 Sapur.009G011600 Sapur.009G021700 Sapur.009G030600 Sapur.009G039700 Sapur.009G045200 Sapur.009G053600 Sapur.009G079400

Sapur.009G080900	Sapur.009G082700	Sapur.009G085400	Sapur.009G085600	Sapur.009G085700	Sapur.009G086400
Sapur.009G087200	Sapur.009G089000	Sapur.009G097200	Sapur.009G104900	Sapur.009G112500	Sapur.009G112800
Sapur.009G114600	Sapur.009G126000	Sapur.010G021800	Sapur.010G022000	Sapur.010G035500	Sapur.010G035800
Sapur.010G035900	Sapur.010G043500	Sapur.010G044400	Sapur.010G052700	Sapur.010G053300	Sapur.010G053800
Sapur.010G075900	Sapur.010G078000	Sapur.010G088800	Sapur.010G088900	Sapur.010G089800	Sapur.010G099700
Sapur.010G100200	Sapur.010G109100	Sapur.010G109200	Sapur.010G114800	Sapur.010G116400	Sapur.010G118700
Sapur.010G120000	Sapur.010G120300	Sapur.010G128300	Sapur.010G141100	Sapur.010G149000	Sapur.010G149100
Sapur.010G154300	Sapur.010G157800	Sapur.010G161100	Sapur.010G185200	Sapur.010G187200	Sapur.010G193700
Sapur.010G201700	Sapur.011G032100	Sapur.011G037100	Sapur.011G037900	Sapur.011G038200	Sapur.011G041100
Sapur.011G045100	Sapur.011G045800	Sapur.011G056100	Sapur.011G066700	Sapur.011G082900	Sapur.011G088400
Sapur.011G088500	Sapur.011G097000	Sapur.011G101200	Sapur.011G109600	Sapur.012G004000	Sapur.012G010400
Sapur.012G017700	Sapur.012G018600	Sapur.012G024500	Sapur.012G043000	Sapur.012G051400	Sapur.012G060100
Sapur.012G066500	Sapur.012G073300	Sapur.012G078700	Sapur.012G088900	Sapur.012G100600	Sapur.012G105100
Sapur.013G000100	Sapur.013G008000	Sapur.013G014300	Sapur.013G028900	Sapur.013G050700	Sapur.013G050900
Sapur.013G051000	Sapur.013G056700	Sapur.013G067900	Sapur.013G068800	Sapur.013G080500	Sapur.013G082200
Sapur.013G084100	Sapur.013G084400	Sapur.013G103900	Sapur.013G117300	Sapur.013G138200	Sapur.013G140300
Sapur.014G011100	Sapur.014G011200	Sapur.014G011900	Sapur.014G024600	Sapur.014G026600	Sapur.014G027000
Sapur.014G027100	Sapur.014G027200	Sapur.014G027400	Sapur.014G027800	Sapur.014G028400	Sapur.014G028500
Sapur.014G029000	Sapur.014G034500	Sapur.014G059000	Sapur.014G059100	Sapur.014G059200	Sapur.014G075400
Sapur.014G076200	Sapur.014G078500	Sapur.014G079300	Sapur.014G080900	Sapur.014G083400	Sapur.014G085400
Sapur.014G085500	Sapur.014G113600	Sapur.014G117700	Sapur.014G120800	Sapur.014G126900	Sapur.014G135200
Sapur.014G142400	Sapur.016G007300	Sapur.016G007700	Sapur.016G007900	Sapur.016G018700	Sapur.016G026400
Sapur.016G026800	Sapur.016G028200	Sapur.016G030200	Sapur.016G043500	Sapur.016G055400	Sapur.016G058000
Sapur.016G058600	Sapur.016G064600	Sapur.016G070400	Sapur.016G072900	Sapur.016G085100	Sapur.016G114700
Sapur.016G117100	Sapur.016G131300	Sapur.016G144700	Sapur.016G145900	Sapur.016G167800	Sapur.016G183700
Sapur.016G194900	Sapur.016G199700	Sapur.016G205100	Sapur.016G209200	Sapur.016G216900	Sapur.016G223300
Sapur.016G229800	Sapur.016G233500	Sapur.016G241300	Sapur.016G264800	Sapur.016G285400	Sapur.016G285500
Sapur.016G310400	Sapur.016G311100	Sapur.017G028400	Sapur.017G028500	Sapur.017G053300	Sapur.017G058200
Sapur.017G069900	Sapur.017G085900	Sapur.017G096600	Sapur.017G114800	Sapur.017G115700	Sapur.017G119200
Sapur.018G015800	Sapur.018G019900	Sapur.018G030000	Sapur.018G060900	Sapur.018G064600	Sapur.018G081500
Sapur.018G087600	Sapur.018G107600	Sapur.018G114600	Sapur.018G115000	Sapur.019G033200	Sapur.019G039400
Sapur.019G039900	Sapur.019G060100	Sapur.019G065400	Sapur.019G074500	Sapur.019G099800	Sapur.019G101700
Sapur.15WG036700	Sapur.15WG065100	Sapur.15WG067500	Sapur.15WG072800	Sapur.15ZG002000	Sapur.15ZG002400

Sapur.15ZG002500 Sapur.15ZG007300 Sapur.15ZG007500 Sapur.15ZG016900 Sapur.15ZG038900 Sapur.15ZG043500
Sapur.15ZG053200 Sapur.15ZG067200 Sapur.15ZG079000 Sapur.15ZG080900 Sapur.15ZG103400 Sapur.15ZG112800
Sapur.15ZG127500 Sapur.T012000 Sapur.T047000 Sapur.T058600 Sapur.T058800 Sapur.T073900 Sapur.T131000
Sapur.T158900 Sapur.001G017300 Sapur.001G017800 Sapur.001G023800 Sapur.001G027900 Sapur.001G028100
Sapur.001G028400 Sapur.001G029800 Sapur.001G034200 Sapur.001G058300 Sapur.001G068000 Sapur.001G078400
Sapur.001G078600 Sapur.001G084200 Sapur.001G094200 Sapur.001G103000 Sapur.001G108900 Sapur.001G110300
Sapur.001G122200 Sapur.001G135100 Sapur.001G136400 Sapur.001G167000 Sapur.001G174900 Sapur.001G179800
Sapur.001G181600 Sapur.001G189000 Sapur.002G005300 Sapur.002G012900 Sapur.002G013600 Sapur.002G014300
Sapur.002G023300 Sapur.002G024200 Sapur.002G024400 Sapur.002G032100 Sapur.002G033200 Sapur.002G036100
Sapur.002G051400 Sapur.002G061600 Sapur.002G061700 Sapur.002G064800 Sapur.002G073000 Sapur.002G085200
Sapur.002G085900 Sapur.002G089300 Sapur.002G090300 Sapur.002G109500 Sapur.002G123400 Sapur.002G130500
Sapur.002G132500 Sapur.002G146000 Sapur.002G156100 Sapur.002G161200 Sapur.002G163600 Sapur.002G169300
Sapur.002G175800 Sapur.002G191900 Sapur.002G194100 Sapur.003G002000 Sapur.003G002100 Sapur.003G014300
Sapur.003G018100 Sapur.003G025000 Sapur.003G031900 Sapur.003G066000 Sapur.003G067100 Sapur.003G078600
Sapur.003G090400 Sapur.003G092400 Sapur.003G099500 Sapur.003G102000 Sapur.003G114500 Sapur.003G116100
Sapur.003G117300 Sapur.003G125800 Sapur.003G125900 Sapur.003G138200 Sapur.003G138500 Sapur.003G139700
Sapur.003G141900 Sapur.003G145700 Sapur.003G150800 Sapur.003G155400 Sapur.003G156600 Sapur.003G160700
Sapur.004G007000 Sapur.004G014400 Sapur.004G019100 Sapur.004G027800 Sapur.004G033200 Sapur.004G037400
Sapur.004G041000 Sapur.004G051400 Sapur.004G063300 Sapur.004G077700 Sapur.004G108600 Sapur.004G109700
Sapur.004G118800 Sapur.004G121500 Sapur.004G123100 Sapur.004G128600 Sapur.004G131500 Sapur.004G131600
Sapur.004G132200 Sapur.004G137600 Sapur.004G140300 Sapur.004G146400 Sapur.004G147000 Sapur.004G147900
Sapur.004G171400 Sapur.004G172100 Sapur.005G005000 Sapur.005G005700 Sapur.005G007100 Sapur.005G011200
Sapur.005G022300 Sapur.005G023100 Sapur.005G024800 Sapur.005G025000 Sapur.005G025400 Sapur.005G031700
Sapur.005G038400 Sapur.005G040700 Sapur.005G043600 Sapur.005G043700 Sapur.005G046200 Sapur.005G048900
Sapur.005G050200 Sapur.005G055500 Sapur.005G067000 Sapur.005G069000 Sapur.005G075800 Sapur.005G087400
Sapur.005G095700 Sapur.005G096300 Sapur.005G096400 Sapur.005G098100 Sapur.005G109500 Sapur.005G113600
Sapur.005G119500 Sapur.005G133200 Sapur.005G150000 Sapur.005G165600 Sapur.005G193700 Sapur.005G199500
Sapur.005G206400 Sapur.006G010200 Sapur.006G025100 Sapur.006G033400 Sapur.006G036800 Sapur.006G037200
Sapur.006G067400 Sapur.006G067500 Sapur.006G069500 Sapur.006G072700 Sapur.006G089400 Sapur.006G094200
Sapur.006G094300 Sapur.006G110100 Sapur.006G110200 Sapur.006G112500 Sapur.006G118100 Sapur.006G118200
Sapur.006G122700 Sapur.006G126200 Sapur.006G136800 Sapur.006G138300 Sapur.006G139300 Sapur.006G154300
Sapur.006G163000 Sapur.006G168000 Sapur.006G172300 Sapur.006G173100 Sapur.006G181100 Sapur.006G181200
Sapur.006G182100 Sapur.006G194600 Sapur.006G218000 Sapur.006G218800 Sapur.006G221300 Sapur.006G223700

Sapur.006G224200 Sapur.006G224700 Sapur.006G225500 Sapur.006G228700 Sapur.006G228900 Sapur.007G001300
Sapur.007G002900 Sapur.007G007000 Sapur.007G007100 Sapur.007G008900 Sapur.007G016800 Sapur.007G021900
Sapur.007G029800 Sapur.007G036500 Sapur.007G044500 Sapur.007G044600 Sapur.007G062200 Sapur.007G069600
Sapur.007G071200 Sapur.007G075800 Sapur.007G077000 Sapur.007G077600 Sapur.007G087100 Sapur.007G111300
Sapur.007G111400 Sapur.007G111700 Sapur.007G112200 Sapur.008G004200 Sapur.008G004700 Sapur.008G004900
Sapur.008G009100 Sapur.008G022900 Sapur.008G026800 Sapur.008G045900 Sapur.008G046000 Sapur.008G058400
Sapur.008G060200 Sapur.008G077900 Sapur.008G081500 Sapur.008G081900 Sapur.008G088300 Sapur.008G090300
Sapur.008G091700 Sapur.008G115400 Sapur.008G128300 Sapur.008G131900 Sapur.008G137200 Sapur.008G151300
Sapur.008G151900 Sapur.008G162000 Sapur.009G004200 Sapur.009G005200 Sapur.009G018000 Sapur.009G041000
Sapur.009G051100 Sapur.009G057000 Sapur.009G064100 Sapur.009G064400 Sapur.009G066300 Sapur.009G094100
Sapur.009G094200 Sapur.009G094800 Sapur.009G097900 Sapur.009G099800 Sapur.009G116000 Sapur.009G117400
Sapur.009G120100 Sapur.009G122200 Sapur.010G001900 Sapur.010G003100 Sapur.010G009400 Sapur.010G009500
Sapur.010G014000 Sapur.010G024600 Sapur.010G026800 Sapur.010G042700 Sapur.010G042900 Sapur.010G046700
Sapur.010G055900 Sapur.010G057900 Sapur.010G060700 Sapur.010G065900 Sapur.010G070700 Sapur.010G086100
Sapur.010G087600 Sapur.010G092300 Sapur.010G092700 Sapur.010G093900 Sapur.010G100000 Sapur.010G102500
Sapur.010G104500 Sapur.010G114700 Sapur.010G119700 Sapur.010G119800 Sapur.010G123400 Sapur.010G137200
Sapur.010G138500 Sapur.010G143600 Sapur.010G157400 Sapur.010G163200 Sapur.010G193600 Sapur.011G006600
Sapur.011G008400 Sapur.011G013300 Sapur.011G018500 Sapur.011G028400 Sapur.011G031900 Sapur.011G034200
Sapur.011G035500 Sapur.011G037200 Sapur.011G049300 Sapur.011G054600 Sapur.011G061100 Sapur.011G073000
Sapur.011G088900 Sapur.011G099500 Sapur.011G103500 Sapur.011G103700 Sapur.011G103800 Sapur.011G120500
Sapur.012G000700 Sapur.012G031400 Sapur.012G037400 Sapur.012G040700 Sapur.012G048900 Sapur.012G050600
Sapur.012G061900 Sapur.012G067300 Sapur.012G070700 Sapur.012G074000 Sapur.012G074300 Sapur.012G084200
Sapur.012G086700 Sapur.012G087900 Sapur.012G091400 Sapur.012G091800 Sapur.012G092600 Sapur.012G092900
Sapur.012G101100 Sapur.012G104300 Sapur.013G004800 Sapur.013G008600 Sapur.013G010700 Sapur.013G021200
Sapur.013G039500 Sapur.013G045400 Sapur.013G055500 Sapur.013G055900 Sapur.013G087600 Sapur.013G097100
Sapur.013G100200 Sapur.013G110800 Sapur.013G117900 Sapur.013G118200 Sapur.013G118400 Sapur.013G120100
Sapur.013G120600 Sapur.013G123000 Sapur.013G123300 Sapur.013G123700 Sapur.013G124400 Sapur.013G137400
Sapur.014G001300 Sapur.014G001700 Sapur.014G002600 Sapur.014G003700 Sapur.014G005100 Sapur.014G005200
Sapur.014G025600 Sapur.014G032900 Sapur.014G036100 Sapur.014G052300 Sapur.014G053000 Sapur.014G055200
Sapur.014G055500 Sapur.014G061800 Sapur.014G073900 Sapur.014G080400 Sapur.014G084500 Sapur.014G090200
Sapur.014G090300 Sapur.014G094500 Sapur.014G095700 Sapur.014G099500 Sapur.014G104700 Sapur.014G106600
Sapur.014G110600 Sapur.014G113100 Sapur.014G117400 Sapur.014G120400 Sapur.014G132000 Sapur.014G134700
Sapur.014G135900 Sapur.016G009300 Sapur.016G018900 Sapur.016G031400 Sapur.016G035600 Sapur.016G040000

Sapur.016G051900 Sapur.016G052700 Sapur.016G060400 Sapur.016G071200 Sapur.016G077600 Sapur.016G079900
Sapur.016G080200 Sapur.016G104800 Sapur.016G111900 Sapur.016G120800 Sapur.016G123500 Sapur.016G125300
Sapur.016G132300 Sapur.016G132400 Sapur.016G141300 Sapur.016G147900 Sapur.016G150200 Sapur.016G152700
Sapur.016G159000 Sapur.016G163400 Sapur.016G189500 Sapur.016G189900 Sapur.016G200900 Sapur.016G224700
Sapur.016G230700 Sapur.016G230800 Sapur.016G243200 Sapur.016G243400 Sapur.016G247200 Sapur.016G254800
Sapur.016G262400 Sapur.016G281900 Sapur.016G282700 Sapur.016G288500 Sapur.016G298800 Sapur.016G299400
Sapur.016G302100 Sapur.016G302500 Sapur.016G304100 Sapur.017G003500 Sapur.017G009300 Sapur.017G010500
Sapur.017G018700 Sapur.017G020600 Sapur.017G023600 Sapur.017G023800 Sapur.017G029100 Sapur.017G036400
Sapur.017G036800 Sapur.017G037700 Sapur.017G052400 Sapur.017G055300 Sapur.017G057100 Sapur.017G057300
Sapur.017G057900 Sapur.017G074500 Sapur.017G100600 Sapur.017G101100 Sapur.017G101200 Sapur.017G102500
Sapur.017G104800 Sapur.017G108100 Sapur.017G108200 Sapur.017G108700 Sapur.017G110500 Sapur.017G110700
Sapur.017G111200 Sapur.017G112200 Sapur.017G112400 Sapur.017G113400 Sapur.017G123300 Sapur.017G124700
Sapur.017G129000 Sapur.018G002400 Sapur.018G003500 Sapur.018G003800 Sapur.018G004300 Sapur.018G005400
Sapur.018G007100 Sapur.018G008500 Sapur.018G015000 Sapur.018G016300 Sapur.018G016500 Sapur.018G016600
Sapur.018G016800 Sapur.018G017000 Sapur.018G023200 Sapur.018G025000 Sapur.018G025700 Sapur.018G035300
Sapur.018G039600 Sapur.018G045400 Sapur.018G047200 Sapur.018G056400 Sapur.018G057100 Sapur.018G057600
Sapur.018G057700 Sapur.018G060400 Sapur.018G063600 Sapur.018G063900 Sapur.018G067200 Sapur.018G077200
Sapur.018G088200 Sapur.018G105800 Sapur.018G106800 Sapur.018G114900 Sapur.018G115800 Sapur.018G115900
Sapur.018G117400 Sapur.019G001100 Sapur.019G003100 Sapur.019G005200 Sapur.019G005900 Sapur.019G007100
Sapur.019G007800 Sapur.019G007900 Sapur.019G009300 Sapur.019G009600 Sapur.019G010500 Sapur.019G010700
Sapur.019G011000 Sapur.019G011200 Sapur.019G011500 Sapur.019G011600 Sapur.019G011700 Sapur.019G011900
Sapur.019G012100 Sapur.019G012500 Sapur.019G012700 Sapur.019G012800 Sapur.019G013700 Sapur.019G016400
Sapur.019G017000 Sapur.019G022800 Sapur.019G023100 Sapur.019G027100 Sapur.019G027300 Sapur.019G027700
Sapur.019G028700 Sapur.019G029100 Sapur.019G034300 Sapur.019G042100 Sapur.019G042400 Sapur.019G042500
Sapur.019G043100 Sapur.019G043200 Sapur.019G044000 Sapur.019G044100 Sapur.019G046700 Sapur.019G048000
Sapur.019G048800 Sapur.019G053500 Sapur.019G058700 Sapur.019G065000 Sapur.019G070000 Sapur.019G075900
Sapur.019G080700 Sapur.019G082400 Sapur.019G091100 Sapur.019G091300 Sapur.019G091700 Sapur.019G092300
Sapur.019G092600 Sapur.019G092700 Sapur.019G092800 Sapur.019G093100 Sapur.019G093400 Sapur.019G093600
Sapur.019G093900 Sapur.019G094300 Sapur.019G107300 Sapur.019G108100 Sapur.019G110800 Sapur.019G111400
Sapur.019G113800 Sapur.15WG030400 Sapur.15WG040400 Sapur.15WG043400 Sapur.15WG044400 Sapur.15WG076100
Sapur.15ZG018400 Sapur.15ZG044100 Sapur.15ZG044300 Sapur.15ZG044900 Sapur.15ZG049500 Sapur.15ZG050400
Sapur.15ZG050500 Sapur.15ZG078500 Sapur.15ZG103200 Sapur.15ZG109900 Sapur.15ZG116100 Sapur.15ZG120200
Sapur.15ZG123100 Sapur.15ZG123200 Sapur.15ZG126400 Sapur.T003900 Sapur.T005400 Sapur.T005500

GO:0097159	980	5307	organic cyclic compound binding	Sapur.T028300 Sapur.T029400 Sapur.T030800 Sapur.T080500 Sapur.T096600 Sapur.T115500 Sapur.T116500 Sapur.T117000 Sapur.T131900 Sapur.T133700 Sapur.T147200 Sapur.T150000 Sapur.T191500 Sapur.T192100 Sapur.001G013800 Sapur.001G025400 Sapur.001G047800 Sapur.001G055600 Sapur.001G058800 Sapur.001G067700 Sapur.001G067900 Sapur.001G069800 Sapur.001G072500 Sapur.001G090900 Sapur.001G119600 Sapur.001G129000 Sapur.001G137000 Sapur.001G148900 Sapur.001G149000 Sapur.001G149100 Sapur.001G151600 Sapur.001G166100 Sapur.001G167600 Sapur.001G167800 Sapur.001G178100 Sapur.001G179000 Sapur.001G193000 Sapur.002G019400 Sapur.002G021500 Sapur.002G023100 Sapur.002G028500 Sapur.002G031600 Sapur.002G035300 Sapur.002G043300 Sapur.002G048900 Sapur.002G064600 Sapur.002G069500 Sapur.002G076800 Sapur.002G080700 Sapur.002G091700 Sapur.002G106000 Sapur.002G107000 Sapur.002G110600 Sapur.002G116100 Sapur.002G125900 Sapur.002G126000 Sapur.002G136900 Sapur.002G141800 Sapur.002G142300 Sapur.002G145300 Sapur.002G160400 Sapur.003G003700 Sapur.003G016500 Sapur.003G016800 Sapur.003G025800 Sapur.003G037400 Sapur.003G037500 Sapur.003G047900 Sapur.003G057500 Sapur.003G068400 Sapur.003G082800 Sapur.003G093200 Sapur.003G109000 Sapur.003G123800 Sapur.003G129600 Sapur.003G130700 Sapur.003G150500 Sapur.003G152800 Sapur.003G159400 Sapur.003G168000 Sapur.004G030500 Sapur.004G030800 Sapur.004G030900 Sapur.004G034900 Sapur.004G054800 Sapur.004G056900 Sapur.004G062200 Sapur.004G088800 Sapur.004G089500 Sapur.004G096100 Sapur.004G101700 Sapur.004G103000 Sapur.004G110300 Sapur.004G121800 Sapur.004G137000 Sapur.004G139700 Sapur.004G165200 Sapur.005G000100 Sapur.005G005600 Sapur.005G019900 Sapur.005G032200 Sapur.005G052900 Sapur.005G054500 Sapur.005G054800 Sapur.005G055700 Sapur.005G085100 Sapur.005G089700 Sapur.005G093100 Sapur.005G095300 Sapur.005G105500 Sapur.005G115200 Sapur.005G119200 Sapur.005G141300 Sapur.005G158900 Sapur.005G163900 Sapur.005G186700 Sapur.005G205700 Sapur.006G018700 Sapur.006G022000 Sapur.006G026800 Sapur.006G043000 Sapur.006G045100 Sapur.006G045200 Sapur.006G063200 Sapur.006G065100 Sapur.006G068600 Sapur.006G098500 Sapur.006G103400 Sapur.006G104700 Sapur.006G114200 Sapur.006G116900 Sapur.006G118700 Sapur.006G121100 Sapur.006G123200 Sapur.006G134000 Sapur.006G136200 Sapur.006G154200 Sapur.006G165100 Sapur.006G166000 Sapur.006G170000 Sapur.006G186800 Sapur.006G194100 Sapur.006G214400 Sapur.007G034000 Sapur.007G035800 Sapur.007G049700 Sapur.007G058700 Sapur.007G078000 Sapur.007G080700 Sapur.007G080800 Sapur.007G087800 Sapur.007G089800 Sapur.007G091900 Sapur.007G094000 Sapur.007G103200 Sapur.007G108700 Sapur.007G115600 Sapur.008G021500 Sapur.008G042700 Sapur.008G045800 Sapur.008G053100 Sapur.008G060600 Sapur.008G064800 Sapur.008G066900 Sapur.008G069200 Sapur.008G080600 Sapur.008G090400 Sapur.008G093800 Sapur.008G094300 Sapur.008G104300 Sapur.008G118700 Sapur.008G119700 Sapur.008G131800 Sapur.008G140000 Sapur.008G156200 Sapur.008G162200 Sapur.008G166400 Sapur.009G002900 Sapur.009G005300 Sapur.009G009700 Sapur.009G011600 Sapur.009G021700 Sapur.009G030600 Sapur.009G039700 Sapur.009G045200 Sapur.009G053600 Sapur.009G079400 Sapur.009G080900 Sapur.009G082700 Sapur.009G085400 Sapur.009G085600 Sapur.009G085700 Sapur.009G086400 Sapur.009G087200 Sapur.009G089000 Sapur.009G097200 Sapur.009G104900 Sapur.009G112500 Sapur.009G112800

Sapur.009G114600	Sapur.009G126000	Sapur.010G021800	Sapur.010G022000	Sapur.010G035500	Sapur.010G035800
Sapur.010G035900	Sapur.010G043500	Sapur.010G044400	Sapur.010G052700	Sapur.010G053300	Sapur.010G053800
Sapur.010G075900	Sapur.010G078000	Sapur.010G088800	Sapur.010G088900	Sapur.010G089800	Sapur.010G099700
Sapur.010G100200	Sapur.010G109100	Sapur.010G109200	Sapur.010G114800	Sapur.010G116400	Sapur.010G118700
Sapur.010G120000	Sapur.010G120300	Sapur.010G128300	Sapur.010G141100	Sapur.010G149000	Sapur.010G149100
Sapur.010G154300	Sapur.010G157800	Sapur.010G161100	Sapur.010G185200	Sapur.010G187200	Sapur.010G193700
Sapur.010G201700	Sapur.011G032100	Sapur.011G037100	Sapur.011G037900	Sapur.011G038200	Sapur.011G041100
Sapur.011G045100	Sapur.011G045800	Sapur.011G056100	Sapur.011G066700	Sapur.011G082900	Sapur.011G088400
Sapur.011G088500	Sapur.011G097000	Sapur.011G101200	Sapur.011G109600	Sapur.012G004000	Sapur.012G010400
Sapur.012G017700	Sapur.012G018600	Sapur.012G024500	Sapur.012G043000	Sapur.012G051400	Sapur.012G060100
Sapur.012G066500	Sapur.012G073300	Sapur.012G078700	Sapur.012G088900	Sapur.012G100600	Sapur.012G105100
Sapur.013G000100	Sapur.013G008000	Sapur.013G014300	Sapur.013G028900	Sapur.013G050700	Sapur.013G050900
Sapur.013G051000	Sapur.013G056700	Sapur.013G067900	Sapur.013G068800	Sapur.013G080500	Sapur.013G082200
Sapur.013G084100	Sapur.013G084400	Sapur.013G103900	Sapur.013G117300	Sapur.013G138200	Sapur.013G140300
Sapur.014G011100	Sapur.014G011200	Sapur.014G011900	Sapur.014G024600	Sapur.014G026600	Sapur.014G027000
Sapur.014G027100	Sapur.014G027200	Sapur.014G027400	Sapur.014G027800	Sapur.014G028400	Sapur.014G028500
Sapur.014G029000	Sapur.014G034500	Sapur.014G059000	Sapur.014G059100	Sapur.014G059200	Sapur.014G075400
Sapur.014G076200	Sapur.014G078500	Sapur.014G079300	Sapur.014G080900	Sapur.014G083400	Sapur.014G085400
Sapur.014G085500	Sapur.014G113600	Sapur.014G117700	Sapur.014G120800	Sapur.014G126900	Sapur.014G135200
Sapur.014G142400	Sapur.016G007300	Sapur.016G007700	Sapur.016G007900	Sapur.016G018700	Sapur.016G026400
Sapur.016G026800	Sapur.016G028200	Sapur.016G030200	Sapur.016G043500	Sapur.016G055400	Sapur.016G058000
Sapur.016G058600	Sapur.016G064600	Sapur.016G070400	Sapur.016G072900	Sapur.016G085100	Sapur.016G114700
Sapur.016G117100	Sapur.016G131300	Sapur.016G144700	Sapur.016G145900	Sapur.016G167800	Sapur.016G183700
Sapur.016G194900	Sapur.016G199700	Sapur.016G205100	Sapur.016G209200	Sapur.016G216900	Sapur.016G223300
Sapur.016G229800	Sapur.016G233500	Sapur.016G241300	Sapur.016G264800	Sapur.016G285400	Sapur.016G285500
Sapur.016G310400	Sapur.016G311100	Sapur.017G028400	Sapur.017G028500	Sapur.017G053300	Sapur.017G058200
Sapur.017G069900	Sapur.017G085900	Sapur.017G096600	Sapur.017G114800	Sapur.017G115700	Sapur.017G119200
Sapur.018G015800	Sapur.018G019900	Sapur.018G030000	Sapur.018G060900	Sapur.018G064600	Sapur.018G081500
Sapur.018G087600	Sapur.018G107600	Sapur.018G114600	Sapur.018G115000	Sapur.019G033200	Sapur.019G039400
Sapur.019G039900	Sapur.019G060100	Sapur.019G065400	Sapur.019G074500	Sapur.019G099800	Sapur.019G101700
Sapur.15WG036700	Sapur.15WG065100	Sapur.15WG067500	Sapur.15WG072800	Sapur.15ZG002000	Sapur.15ZG002400
Sapur.15ZG002500	Sapur.15ZG007300	Sapur.15ZG007500	Sapur.15ZG016900	Sapur.15ZG038900	Sapur.15ZG043500
Sapur.15ZG053200	Sapur.15ZG067200	Sapur.15ZG079000	Sapur.15ZG080900	Sapur.15ZG103400	Sapur.15ZG112800

Sapur.15ZG127500 Sapur.T012000 Sapur.T047000 Sapur.T058600 Sapur.T058800 Sapur.T073900 Sapur.T131000
Sapur.T158900 Sapur.001G017300 Sapur.001G017800 Sapur.001G023800 Sapur.001G027900 Sapur.001G028100
Sapur.001G028400 Sapur.001G029800 Sapur.001G034200 Sapur.001G058300 Sapur.001G068000 Sapur.001G078400
Sapur.001G078600 Sapur.001G084200 Sapur.001G094200 Sapur.001G103000 Sapur.001G108900 Sapur.001G110300
Sapur.001G122200 Sapur.001G135100 Sapur.001G136400 Sapur.001G167000 Sapur.001G174900 Sapur.001G179800
Sapur.001G181600 Sapur.001G189000 Sapur.002G005300 Sapur.002G012900 Sapur.002G013600 Sapur.002G014300
Sapur.002G023300 Sapur.002G024200 Sapur.002G024400 Sapur.002G032100 Sapur.002G033200 Sapur.002G036100
Sapur.002G051400 Sapur.002G061600 Sapur.002G061700 Sapur.002G064800 Sapur.002G073000 Sapur.002G085200
Sapur.002G085900 Sapur.002G089300 Sapur.002G090300 Sapur.002G109500 Sapur.002G123400 Sapur.002G130500
Sapur.002G132500 Sapur.002G146000 Sapur.002G156100 Sapur.002G161200 Sapur.002G163600 Sapur.002G169300
Sapur.002G175800 Sapur.002G191900 Sapur.002G194100 Sapur.003G002000 Sapur.003G002100 Sapur.003G014300
Sapur.003G018100 Sapur.003G025000 Sapur.003G031900 Sapur.003G066000 Sapur.003G067100 Sapur.003G078600
Sapur.003G090400 Sapur.003G092400 Sapur.003G099500 Sapur.003G102000 Sapur.003G114500 Sapur.003G116100
Sapur.003G117300 Sapur.003G125800 Sapur.003G125900 Sapur.003G138200 Sapur.003G138500 Sapur.003G139700
Sapur.003G141900 Sapur.003G145700 Sapur.003G150800 Sapur.003G155400 Sapur.003G156600 Sapur.003G160700
Sapur.004G007000 Sapur.004G014400 Sapur.004G019100 Sapur.004G027800 Sapur.004G033200 Sapur.004G037400
Sapur.004G041000 Sapur.004G051400 Sapur.004G063300 Sapur.004G077700 Sapur.004G108600 Sapur.004G109700
Sapur.004G118800 Sapur.004G121500 Sapur.004G123100 Sapur.004G128600 Sapur.004G131500 Sapur.004G131600
Sapur.004G132200 Sapur.004G137600 Sapur.004G140300 Sapur.004G146400 Sapur.004G147000 Sapur.004G147900
Sapur.004G171400 Sapur.004G172100 Sapur.005G005000 Sapur.005G005700 Sapur.005G007100 Sapur.005G011200
Sapur.005G022300 Sapur.005G023100 Sapur.005G024800 Sapur.005G025000 Sapur.005G025400 Sapur.005G031700
Sapur.005G038400 Sapur.005G040700 Sapur.005G043600 Sapur.005G043700 Sapur.005G046200 Sapur.005G048900
Sapur.005G050200 Sapur.005G055500 Sapur.005G067000 Sapur.005G069000 Sapur.005G075800 Sapur.005G087400
Sapur.005G095700 Sapur.005G096300 Sapur.005G096400 Sapur.005G098100 Sapur.005G109500 Sapur.005G113600
Sapur.005G119500 Sapur.005G133200 Sapur.005G150000 Sapur.005G165600 Sapur.005G193700 Sapur.005G199500
Sapur.005G206400 Sapur.006G010200 Sapur.006G025100 Sapur.006G033400 Sapur.006G036800 Sapur.006G037200
Sapur.006G067400 Sapur.006G067500 Sapur.006G069500 Sapur.006G072700 Sapur.006G089400 Sapur.006G094200
Sapur.006G094300 Sapur.006G110100 Sapur.006G110200 Sapur.006G112500 Sapur.006G118100 Sapur.006G118200
Sapur.006G122700 Sapur.006G126200 Sapur.006G136800 Sapur.006G138300 Sapur.006G139300 Sapur.006G154300
Sapur.006G163000 Sapur.006G168000 Sapur.006G172300 Sapur.006G173100 Sapur.006G181100 Sapur.006G181200
Sapur.006G182100 Sapur.006G194600 Sapur.006G218000 Sapur.006G218800 Sapur.006G221300 Sapur.006G223700
Sapur.006G224200 Sapur.006G224700 Sapur.006G225500 Sapur.006G228700 Sapur.006G228900 Sapur.007G001300
Sapur.007G002900 Sapur.007G007000 Sapur.007G007100 Sapur.007G008900 Sapur.007G016800 Sapur.007G021900

Sapur.007G029800	Sapur.007G036500	Sapur.007G044500	Sapur.007G044600	Sapur.007G062200	Sapur.007G069600
Sapur.007G071200	Sapur.007G075800	Sapur.007G077000	Sapur.007G077600	Sapur.007G087100	Sapur.007G111300
Sapur.007G111400	Sapur.007G111700	Sapur.007G112200	Sapur.008G004200	Sapur.008G004700	Sapur.008G004900
Sapur.008G009100	Sapur.008G022900	Sapur.008G026800	Sapur.008G045900	Sapur.008G046000	Sapur.008G058400
Sapur.008G060200	Sapur.008G077900	Sapur.008G081500	Sapur.008G081900	Sapur.008G088300	Sapur.008G090300
Sapur.008G091700	Sapur.008G115400	Sapur.008G128300	Sapur.008G131900	Sapur.008G137200	Sapur.008G151300
Sapur.008G151900	Sapur.008G162000	Sapur.009G004200	Sapur.009G005200	Sapur.009G018000	Sapur.009G041000
Sapur.009G051100	Sapur.009G057000	Sapur.009G064100	Sapur.009G064400	Sapur.009G066300	Sapur.009G094100
Sapur.009G094200	Sapur.009G094800	Sapur.009G097900	Sapur.009G099800	Sapur.009G116000	Sapur.009G117400
Sapur.009G120100	Sapur.009G122200	Sapur.010G001900	Sapur.010G003100	Sapur.010G009400	Sapur.010G009500
Sapur.010G014000	Sapur.010G024600	Sapur.010G026800	Sapur.010G042700	Sapur.010G042900	Sapur.010G046700
Sapur.010G055900	Sapur.010G057900	Sapur.010G060700	Sapur.010G065900	Sapur.010G070700	Sapur.010G086100
Sapur.010G087600	Sapur.010G092300	Sapur.010G092700	Sapur.010G093900	Sapur.010G100000	Sapur.010G102500
Sapur.010G104500	Sapur.010G114700	Sapur.010G119700	Sapur.010G119800	Sapur.010G123400	Sapur.010G137200
Sapur.010G138500	Sapur.010G143600	Sapur.010G157400	Sapur.010G163200	Sapur.010G193600	Sapur.011G006600
Sapur.011G008400	Sapur.011G013300	Sapur.011G018500	Sapur.011G028400	Sapur.011G031900	Sapur.011G034200
Sapur.011G035500	Sapur.011G037200	Sapur.011G049300	Sapur.011G054600	Sapur.011G061100	Sapur.011G073000
Sapur.011G088900	Sapur.011G099500	Sapur.011G103500	Sapur.011G103700	Sapur.011G103800	Sapur.011G120500
Sapur.012G000700	Sapur.012G031400	Sapur.012G037400	Sapur.012G040700	Sapur.012G048900	Sapur.012G050600
Sapur.012G061900	Sapur.012G067300	Sapur.012G070700	Sapur.012G074000	Sapur.012G074300	Sapur.012G084200
Sapur.012G086700	Sapur.012G087900	Sapur.012G091400	Sapur.012G091800	Sapur.012G092600	Sapur.012G092900
Sapur.012G101100	Sapur.012G104300	Sapur.013G004800	Sapur.013G008600	Sapur.013G010700	Sapur.013G021200
Sapur.013G039500	Sapur.013G045400	Sapur.013G055500	Sapur.013G055900	Sapur.013G087600	Sapur.013G097100
Sapur.013G100200	Sapur.013G110800	Sapur.013G117900	Sapur.013G118200	Sapur.013G118400	Sapur.013G120100
Sapur.013G120600	Sapur.013G123000	Sapur.013G123300	Sapur.013G123700	Sapur.013G124400	Sapur.013G137400
Sapur.014G001300	Sapur.014G001700	Sapur.014G002600	Sapur.014G003700	Sapur.014G005100	Sapur.014G005200
Sapur.014G025600	Sapur.014G032900	Sapur.014G036100	Sapur.014G052300	Sapur.014G053000	Sapur.014G055200
Sapur.014G055500	Sapur.014G061800	Sapur.014G073900	Sapur.014G080400	Sapur.014G084500	Sapur.014G090200
Sapur.014G090300	Sapur.014G094500	Sapur.014G095700	Sapur.014G099500	Sapur.014G104700	Sapur.014G106600
Sapur.014G110600	Sapur.014G113100	Sapur.014G117400	Sapur.014G120400	Sapur.014G132000	Sapur.014G134700
Sapur.014G135900	Sapur.016G009300	Sapur.016G018900	Sapur.016G031400	Sapur.016G035600	Sapur.016G040000
Sapur.016G051900	Sapur.016G052700	Sapur.016G060400	Sapur.016G071200	Sapur.016G077600	Sapur.016G079900
Sapur.016G080200	Sapur.016G104800	Sapur.016G111900	Sapur.016G120800	Sapur.016G123500	Sapur.016G125300

Sapur.016G132300	Sapur.016G132400	Sapur.016G141300	Sapur.016G147900	Sapur.016G150200	Sapur.016G152700
Sapur.016G159000	Sapur.016G163400	Sapur.016G189500	Sapur.016G189900	Sapur.016G200900	Sapur.016G224700
Sapur.016G230700	Sapur.016G230800	Sapur.016G243200	Sapur.016G243400	Sapur.016G247200	Sapur.016G254800
Sapur.016G262400	Sapur.016G281900	Sapur.016G282700	Sapur.016G288500	Sapur.016G298800	Sapur.016G299400
Sapur.016G302100	Sapur.016G302500	Sapur.016G304100	Sapur.017G003500	Sapur.017G009300	Sapur.017G010500
Sapur.017G018700	Sapur.017G020600	Sapur.017G023600	Sapur.017G023800	Sapur.017G029100	Sapur.017G036400
Sapur.017G036800	Sapur.017G037700	Sapur.017G052400	Sapur.017G055300	Sapur.017G057100	Sapur.017G057300
Sapur.017G057900	Sapur.017G074500	Sapur.017G100600	Sapur.017G101100	Sapur.017G101200	Sapur.017G102500
Sapur.017G104800	Sapur.017G108100	Sapur.017G108200	Sapur.017G108700	Sapur.017G110500	Sapur.017G110700
Sapur.017G111200	Sapur.017G112200	Sapur.017G112400	Sapur.017G113400	Sapur.017G123300	Sapur.017G124700
Sapur.017G129000	Sapur.018G002400	Sapur.018G003500	Sapur.018G003800	Sapur.018G004300	Sapur.018G005400
Sapur.018G007100	Sapur.018G008500	Sapur.018G015000	Sapur.018G016300	Sapur.018G016500	Sapur.018G016600
Sapur.018G016800	Sapur.018G017000	Sapur.018G023200	Sapur.018G025000	Sapur.018G025700	Sapur.018G035300
Sapur.018G039600	Sapur.018G045400	Sapur.018G047200	Sapur.018G056400	Sapur.018G057100	Sapur.018G057600
Sapur.018G057700	Sapur.018G060400	Sapur.018G063600	Sapur.018G063900	Sapur.018G067200	Sapur.018G077200
Sapur.018G088200	Sapur.018G105800	Sapur.018G106800	Sapur.018G114900	Sapur.018G115800	Sapur.018G115900
Sapur.018G117400	Sapur.019G001100	Sapur.019G003100	Sapur.019G005200	Sapur.019G005900	Sapur.019G007100
Sapur.019G007800	Sapur.019G007900	Sapur.019G009300	Sapur.019G009600	Sapur.019G010500	Sapur.019G010700
Sapur.019G011000	Sapur.019G011200	Sapur.019G011500	Sapur.019G011600	Sapur.019G011700	Sapur.019G011900
Sapur.019G012100	Sapur.019G012500	Sapur.019G012700	Sapur.019G012800	Sapur.019G013700	Sapur.019G016400
Sapur.019G017000	Sapur.019G022800	Sapur.019G023100	Sapur.019G027100	Sapur.019G027300	Sapur.019G027700
Sapur.019G028700	Sapur.019G029100	Sapur.019G034300	Sapur.019G042100	Sapur.019G042400	Sapur.019G042500
Sapur.019G043100	Sapur.019G043200	Sapur.019G044000	Sapur.019G044100	Sapur.019G046700	Sapur.019G048000
Sapur.019G048800	Sapur.019G053500	Sapur.019G058700	Sapur.019G065000	Sapur.019G070000	Sapur.019G075900
Sapur.019G080700	Sapur.019G082400	Sapur.019G091100	Sapur.019G091300	Sapur.019G091700	Sapur.019G092300
Sapur.019G092600	Sapur.019G092700	Sapur.019G092800	Sapur.019G093100	Sapur.019G093400	Sapur.019G093600
Sapur.019G093900	Sapur.019G094300	Sapur.019G107300	Sapur.019G108100	Sapur.019G110800	Sapur.019G111400
Sapur.019G113800	Sapur.15WG030400	Sapur.15WG040400	Sapur.15WG043400	Sapur.15WG044400	Sapur.15WG076100
Sapur.15ZG018400	Sapur.15ZG044100	Sapur.15ZG044300	Sapur.15ZG044900	Sapur.15ZG049500	Sapur.15ZG050400
Sapur.15ZG050500	Sapur.15ZG078500	Sapur.15ZG103200	Sapur.15ZG109900	Sapur.15ZG116100	Sapur.15ZG120200
Sapur.15ZG123100	Sapur.15ZG123200	Sapur.15ZG126400	Sapur.T003900	Sapur.T005400	Sapur.T005500

Sapur.T028300	Sapur.T029400	Sapur.T030800	Sapur.T080500	Sapur.T096600	Sapur.T115500	Sapur.T116500
Sapur.T117000	Sapur.T131900	Sapur.T133700	Sapur.T147200	Sapur.T150000	Sapur.T191500	Sapur.T192100

GO:0015297	30	79	antiporter activity	Sapur.001G094500 Sapur.002G043400 Sapur.002G086100 Sapur.003G081500 Sapur.004G009600 Sapur.004G009700 Sapur.004G138100 Sapur.005G082300 Sapur.005G114300 Sapur.005G163600 Sapur.005G163800 Sapur.008G101500 Sapur.008G101600 Sapur.008G101700 Sapur.009G040500 Sapur.009G097300 Sapur.010G089300 Sapur.011G001000 Sapur.011G080900 Sapur.012G102500 Sapur.012G111400 Sapur.013G071900 Sapur.013G072500 Sapur.013G072600 Sapur.013G072700 Sapur.016G153700 Sapur.019G046000 Sapur.019G064700 Sapur.019G083100 Sapur.003G031900
GO:1901265	528	2702	nucleoside phosphate binding	Sapur.001G017800 Sapur.001G023800 Sapur.001G029800 Sapur.001G055600 Sapur.001G058300 Sapur.001G058800 Sapur.001G078400 Sapur.001G078600 Sapur.001G084200 Sapur.001G094200 Sapur.001G103000 Sapur.001G108900 Sapur.001G110300 Sapur.001G122200 Sapur.001G136400 Sapur.001G137000 Sapur.001G151600 Sapur.001G167000 Sapur.001G174900 Sapur.001G181600 Sapur.001G189000 Sapur.002G005300 Sapur.002G013600 Sapur.002G014300 Sapur.002G023300 Sapur.002G024400 Sapur.002G032100 Sapur.002G036100 Sapur.002G051400 Sapur.002G061600 Sapur.002G061700 Sapur.002G064800 Sapur.002G085200 Sapur.002G085900 Sapur.002G089300 Sapur.002G090300 Sapur.002G109500 Sapur.002G123400 Sapur.002G130500 Sapur.002G132500 Sapur.002G145300 Sapur.002G156100 Sapur.002G161200 Sapur.002G169300 Sapur.002G191900 Sapur.002G194100 Sapur.003G002000 Sapur.003G002100 Sapur.003G014300 Sapur.003G018100 Sapur.003G025000 Sapur.003G031900 Sapur.003G066000 Sapur.003G067100 Sapur.003G078600 Sapur.003G090400 Sapur.003G092400 Sapur.003G099500 Sapur.003G116100 Sapur.003G125800 Sapur.003G138500 Sapur.003G139700 Sapur.003G155400 Sapur.003G156600 Sapur.003G160700 Sapur.003G168000 Sapur.004G014400 Sapur.004G019100 Sapur.004G027800 Sapur.004G033200 Sapur.004G041000 Sapur.004G051400 Sapur.004G063300 Sapur.004G077700 Sapur.004G103000 Sapur.004G108600 Sapur.004G109700 Sapur.004G128600 Sapur.004G131500 Sapur.004G132200 Sapur.004G137600 Sapur.004G140300 Sapur.004G146400 Sapur.004G147000 Sapur.004G147900 Sapur.004G172100 Sapur.005G000100 Sapur.005G005000 Sapur.005G005700 Sapur.005G007100 Sapur.005G011200 Sapur.005G022300 Sapur.005G023100 Sapur.005G024800 Sapur.005G025000 Sapur.005G025400 Sapur.005G031700 Sapur.005G038400 Sapur.005G040700 Sapur.005G043600 Sapur.005G043700 Sapur.005G046200 Sapur.005G048900 Sapur.005G050200 Sapur.005G054500 Sapur.005G055500 Sapur.005G069000 Sapur.005G085100 Sapur.005G095700 Sapur.005G096300 Sapur.005G096400 Sapur.005G109500 Sapur.005G113600 Sapur.005G119500 Sapur.005G165600 Sapur.005G193700 Sapur.005G199500 Sapur.005G206400 Sapur.006G010200 Sapur.006G018700 Sapur.006G025100 Sapur.006G036800 Sapur.006G037200 Sapur.006G067400 Sapur.006G069500 Sapur.006G072700 Sapur.006G094200 Sapur.006G094300 Sapur.006G110200 Sapur.006G112500 Sapur.006G118100 Sapur.006G118200 Sapur.006G126200 Sapur.006G136200 Sapur.006G136800 Sapur.006G138300 Sapur.006G154200 Sapur.006G154300 Sapur.006G163000 Sapur.006G172300 Sapur.006G173100 Sapur.006G181100 Sapur.006G181200 Sapur.006G182100 Sapur.006G194600 Sapur.006G218800 Sapur.006G221300 Sapur.006G223700 Sapur.006G224200 Sapur.006G224700 Sapur.006G225500 Sapur.007G002900 Sapur.007G007000 Sapur.007G007100 Sapur.007G008900 Sapur.007G016800 Sapur.007G029800 Sapur.007G036500 Sapur.007G044500 Sapur.007G069600 Sapur.007G071200

Sapur.007G075800 Sapur.007G077000 Sapur.007G077600 Sapur.007G087100 Sapur.007G091900 Sapur.007G111300
Sapur.007G111400 Sapur.007G111700 Sapur.007G112200 Sapur.007G115600 Sapur.008G004200 Sapur.008G004700
Sapur.008G004900 Sapur.008G009100 Sapur.008G022900 Sapur.008G026800 Sapur.008G045900 Sapur.008G046000
Sapur.008G058400 Sapur.008G060600 Sapur.008G066900 Sapur.008G077900 Sapur.008G081500 Sapur.008G088300
Sapur.008G090300 Sapur.008G091700 Sapur.008G115400 Sapur.008G128300 Sapur.008G131900 Sapur.008G137200
Sapur.008G151300 Sapur.008G151900 Sapur.008G156200 Sapur.008G162000 Sapur.009G002900 Sapur.009G004200
Sapur.009G005200 Sapur.009G018000 Sapur.009G041000 Sapur.009G045200 Sapur.009G051100 Sapur.009G057000
Sapur.009G064100 Sapur.009G066300 Sapur.009G094100 Sapur.009G094200 Sapur.009G099800 Sapur.009G112500
Sapur.009G116000 Sapur.009G117400 Sapur.009G120100 Sapur.009G122200 Sapur.010G001900 Sapur.010G009400
Sapur.010G009500 Sapur.010G014000 Sapur.010G024600 Sapur.010G026800 Sapur.010G042700 Sapur.010G042900
Sapur.010G046700 Sapur.010G052700 Sapur.010G053300 Sapur.010G055900 Sapur.010G057900 Sapur.010G060700
Sapur.010G070700 Sapur.010G086100 Sapur.010G087600 Sapur.010G089800 Sapur.010G092300 Sapur.010G092700
Sapur.010G093900 Sapur.010G100000 Sapur.010G102500 Sapur.010G104500 Sapur.010G118700 Sapur.010G119700
Sapur.010G119800 Sapur.010G137200 Sapur.010G138500 Sapur.010G141100 Sapur.010G143600 Sapur.010G157400
Sapur.010G163200 Sapur.010G187200 Sapur.010G193600 Sapur.011G008400 Sapur.011G013300 Sapur.011G018500
Sapur.011G028400 Sapur.011G031900 Sapur.011G034200 Sapur.011G073000 Sapur.011G088900 Sapur.011G103500
Sapur.011G103700 Sapur.011G103800 Sapur.011G120500 Sapur.012G000700 Sapur.012G037400 Sapur.012G040700
Sapur.012G048900 Sapur.012G050600 Sapur.012G061900 Sapur.012G067300 Sapur.012G070700 Sapur.012G073300
Sapur.012G084200 Sapur.012G086700 Sapur.012G087900 Sapur.012G091400 Sapur.012G091800 Sapur.012G092600
Sapur.012G092900 Sapur.012G100600 Sapur.012G101100 Sapur.012G104300 Sapur.013G000100 Sapur.013G004800
Sapur.013G021200 Sapur.013G045400 Sapur.013G055900 Sapur.013G097100 Sapur.013G100200 Sapur.013G110800
Sapur.013G117900 Sapur.013G118200 Sapur.013G118400 Sapur.013G120100 Sapur.013G120600 Sapur.013G123000
Sapur.013G123300 Sapur.013G123700 Sapur.013G124400 Sapur.014G001300 Sapur.014G001700 Sapur.014G002600
Sapur.014G005100 Sapur.014G025600 Sapur.014G032900 Sapur.014G036100 Sapur.014G052300 Sapur.014G053000
Sapur.014G055200 Sapur.014G055500 Sapur.014G061800 Sapur.014G073900 Sapur.014G076200 Sapur.014G080400
Sapur.014G084500 Sapur.014G090200 Sapur.014G090300 Sapur.014G104700 Sapur.014G106600 Sapur.014G110600
Sapur.014G113100 Sapur.014G113600 Sapur.014G117400 Sapur.014G117700 Sapur.014G120400 Sapur.014G132000
Sapur.014G134700 Sapur.014G135900 Sapur.016G009300 Sapur.016G018900 Sapur.016G031400 Sapur.016G035600
Sapur.016G040000 Sapur.016G051900 Sapur.016G052700 Sapur.016G058000 Sapur.016G060400 Sapur.016G071200
Sapur.016G077600 Sapur.016G120800 Sapur.016G123500 Sapur.016G125300 Sapur.016G132300 Sapur.016G132400
Sapur.016G141300 Sapur.016G147900 Sapur.016G150200 Sapur.016G152700 Sapur.016G163400 Sapur.016G189500
Sapur.016G189900 Sapur.016G200900 Sapur.016G230700 Sapur.016G230800 Sapur.016G243200 Sapur.016G243400
Sapur.016G247200 Sapur.016G262400 Sapur.016G264800 Sapur.016G281900 Sapur.016G282700 Sapur.016G288500

				Sapur.016G302100 Sapur.016G302500 Sapur.016G304100 Sapur.017G003500 Sapur.017G009300 Sapur.017G010500 Sapur.017G018700 Sapur.017G020600 Sapur.017G023600 Sapur.017G023800 Sapur.017G029100 Sapur.017G036400 Sapur.017G036800 Sapur.017G037700 Sapur.017G052400 Sapur.017G055300 Sapur.017G057300 Sapur.017G057900 Sapur.017G074500 Sapur.017G100600 Sapur.017G101100 Sapur.017G101200 Sapur.017G102500 Sapur.017G104800 Sapur.017G108100 Sapur.017G108200 Sapur.017G108700 Sapur.017G110500 Sapur.017G110700 Sapur.017G111200 Sapur.017G112200 Sapur.017G112400 Sapur.017G113400 Sapur.017G123300 Sapur.017G129000 Sapur.018G002400 Sapur.018G003500 Sapur.018G003800 Sapur.018G004300 Sapur.018G005400 Sapur.018G008500 Sapur.018G015000 Sapur.018G015800 Sapur.018G016300 Sapur.018G016500 Sapur.018G016600 Sapur.018G016800 Sapur.018G017000 Sapur.018G023200 Sapur.018G025700 Sapur.018G045400 Sapur.018G047200 Sapur.018G057100 Sapur.018G057600 Sapur.018G060400 Sapur.018G063600 Sapur.018G063900 Sapur.018G067200 Sapur.018G077200 Sapur.018G087600 Sapur.018G088200 Sapur.018G105800 Sapur.018G106800 Sapur.018G114900 Sapur.018G115800 Sapur.018G115900 Sapur.018G117400 Sapur.019G001100 Sapur.019G003100 Sapur.019G005200 Sapur.019G005900 Sapur.019G007100 Sapur.019G007800 Sapur.019G007900 Sapur.019G009300 Sapur.019G009600 Sapur.019G010500 Sapur.019G010700 Sapur.019G011000 Sapur.019G011200 Sapur.019G011500 Sapur.019G011600 Sapur.019G011700 Sapur.019G011900 Sapur.019G012100 Sapur.019G012500 Sapur.019G012700 Sapur.019G012800 Sapur.019G013700 Sapur.019G016400 Sapur.019G017000 Sapur.019G022800 Sapur.019G023100 Sapur.019G027100 Sapur.019G027300 Sapur.019G028700 Sapur.019G029100 Sapur.019G034300 Sapur.019G042100 Sapur.019G042400 Sapur.019G042500 Sapur.019G043100 Sapur.019G043200 Sapur.019G044000 Sapur.019G044100 Sapur.019G046700 Sapur.019G048000 Sapur.019G048800 Sapur.019G053500 Sapur.019G058700 Sapur.019G070000 Sapur.019G075900 Sapur.019G080700 Sapur.019G082400 Sapur.019G091100 Sapur.019G091300 Sapur.019G091700 Sapur.019G092300 Sapur.019G092600 Sapur.019G092700 Sapur.019G092800 Sapur.019G093100 Sapur.019G093400 Sapur.019G093600 Sapur.019G093900 Sapur.019G094300 Sapur.019G107300 Sapur.019G110800 Sapur.019G111400 Sapur.019G113800 Sapur.15WG030400 Sapur.15WG036700 Sapur.15WG040400 Sapur.15WG043400 Sapur.15WG044400 Sapur.15WG076100 Sapur.15ZG018400 Sapur.15ZG038900 Sapur.15ZG044100 Sapur.15ZG044300 Sapur.15ZG044900 Sapur.15ZG049500 Sapur.15ZG050400 Sapur.15ZG050500 Sapur.15ZG078500 Sapur.15ZG103200 Sapur.15ZG109900 Sapur.15ZG116100 Sapur.15ZG120200 Sapur.15ZG123200 Sapur.15ZG126400 Sapur.T003900 Sapur.T005400 Sapur.T005500 Sapur.T028300 Sapur.T029400 Sapur.T030800 Sapur.T058800 Sapur.T080500 Sapur.T096600 Sapur.T115500 Sapur.T116500 Sapur.T117000 Sapur.T131900 Sapur.T133700 Sapur.T147200 Sapur.T150000 Sapur.T191500 Sapur.T192100
GO:0000166	528	2702	nucleotide binding	Sapur.001G017800 Sapur.001G023800 Sapur.001G029800 Sapur.001G055600 Sapur.001G058300 Sapur.001G058800 Sapur.001G078400 Sapur.001G078600 Sapur.001G084200 Sapur.001G094200 Sapur.001G103000 Sapur.001G108900 Sapur.001G110300 Sapur.001G122200 Sapur.001G136400 Sapur.001G137000 Sapur.001G151600 Sapur.001G167000 Sapur.001G174900 Sapur.001G181600 Sapur.001G189000 Sapur.002G005300 Sapur.002G013600 Sapur.002G014300 Sapur.002G023300 Sapur.002G024400 Sapur.002G032100 Sapur.002G036100 Sapur.002G051400

Sapur.002G061600 Sapur.002G061700 Sapur.002G064800 Sapur.002G085200 Sapur.002G085900 Sapur.002G089300
Sapur.002G090300 Sapur.002G109500 Sapur.002G123400 Sapur.002G130500 Sapur.002G132500 Sapur.002G145300
Sapur.002G156100 Sapur.002G161200 Sapur.002G169300 Sapur.002G191900 Sapur.002G194100 Sapur.003G002000
Sapur.003G002100 Sapur.003G014300 Sapur.003G018100 Sapur.003G025000 Sapur.003G031900 Sapur.003G066000
Sapur.003G067100 Sapur.003G078600 Sapur.003G090400 Sapur.003G092400 Sapur.003G099500 Sapur.003G116100
Sapur.003G125800 Sapur.003G138500 Sapur.003G139700 Sapur.003G155400 Sapur.003G156600 Sapur.003G160700
Sapur.003G168000 Sapur.004G014400 Sapur.004G019100 Sapur.004G027800 Sapur.004G033200 Sapur.004G041000
Sapur.004G051400 Sapur.004G063300 Sapur.004G077700 Sapur.004G103000 Sapur.004G108600 Sapur.004G109700
Sapur.004G128600 Sapur.004G131500 Sapur.004G132200 Sapur.004G137600 Sapur.004G140300 Sapur.004G146400
Sapur.004G147000 Sapur.004G147900 Sapur.004G172100 Sapur.005G000100 Sapur.005G005000 Sapur.005G005700
Sapur.005G007100 Sapur.005G011200 Sapur.005G022300 Sapur.005G023100 Sapur.005G024800 Sapur.005G025000
Sapur.005G025400 Sapur.005G031700 Sapur.005G038400 Sapur.005G040700 Sapur.005G043600 Sapur.005G043700
Sapur.005G046200 Sapur.005G048900 Sapur.005G050200 Sapur.005G054500 Sapur.005G055500 Sapur.005G069000
Sapur.005G085100 Sapur.005G095700 Sapur.005G096300 Sapur.005G096400 Sapur.005G109500 Sapur.005G113600
Sapur.005G119500 Sapur.005G165600 Sapur.005G193700 Sapur.005G199500 Sapur.005G206400 Sapur.006G010200
Sapur.006G018700 Sapur.006G025100 Sapur.006G036800 Sapur.006G037200 Sapur.006G067400 Sapur.006G069500
Sapur.006G072700 Sapur.006G094200 Sapur.006G094300 Sapur.006G110200 Sapur.006G112500 Sapur.006G118100
Sapur.006G118200 Sapur.006G126200 Sapur.006G136200 Sapur.006G136800 Sapur.006G138300 Sapur.006G154200
Sapur.006G154300 Sapur.006G163000 Sapur.006G172300 Sapur.006G173100 Sapur.006G181100 Sapur.006G181200
Sapur.006G182100 Sapur.006G194600 Sapur.006G218800 Sapur.006G221300 Sapur.006G223700 Sapur.006G224200
Sapur.006G224700 Sapur.006G225500 Sapur.007G002900 Sapur.007G007000 Sapur.007G007100 Sapur.007G008900
Sapur.007G016800 Sapur.007G029800 Sapur.007G036500 Sapur.007G044500 Sapur.007G069600 Sapur.007G071200
Sapur.007G075800 Sapur.007G077000 Sapur.007G077600 Sapur.007G087100 Sapur.007G091900 Sapur.007G111300
Sapur.007G111400 Sapur.007G111700 Sapur.007G112200 Sapur.007G115600 Sapur.008G004200 Sapur.008G004700
Sapur.008G004900 Sapur.008G009100 Sapur.008G022900 Sapur.008G026800 Sapur.008G045900 Sapur.008G046000
Sapur.008G058400 Sapur.008G060600 Sapur.008G066900 Sapur.008G077900 Sapur.008G081500 Sapur.008G088300
Sapur.008G090300 Sapur.008G091700 Sapur.008G115400 Sapur.008G128300 Sapur.008G131900 Sapur.008G137200
Sapur.008G151300 Sapur.008G151900 Sapur.008G156200 Sapur.008G162000 Sapur.009G002900 Sapur.009G004200
Sapur.009G005200 Sapur.009G018000 Sapur.009G041000 Sapur.009G045200 Sapur.009G051100 Sapur.009G057000
Sapur.009G064100 Sapur.009G066300 Sapur.009G094100 Sapur.009G094200 Sapur.009G099800 Sapur.009G112500
Sapur.009G116000 Sapur.009G117400 Sapur.009G120100 Sapur.009G122200 Sapur.010G001900 Sapur.010G009400
Sapur.010G009500 Sapur.010G014000 Sapur.010G024600 Sapur.010G026800 Sapur.010G042700 Sapur.010G042900
Sapur.010G046700 Sapur.010G052700 Sapur.010G053300 Sapur.010G055900 Sapur.010G057900 Sapur.010G060700

Sapur.010G070700	Sapur.010G086100	Sapur.010G087600	Sapur.010G089800	Sapur.010G092300	Sapur.010G092700
Sapur.010G093900	Sapur.010G100000	Sapur.010G102500	Sapur.010G104500	Sapur.010G118700	Sapur.010G119700
Sapur.010G119800	Sapur.010G137200	Sapur.010G138500	Sapur.010G141100	Sapur.010G143600	Sapur.010G157400
Sapur.010G163200	Sapur.010G187200	Sapur.010G193600	Sapur.011G008400	Sapur.011G013300	Sapur.011G018500
Sapur.011G028400	Sapur.011G031900	Sapur.011G034200	Sapur.011G073000	Sapur.011G088900	Sapur.011G103500
Sapur.011G103700	Sapur.011G103800	Sapur.011G120500	Sapur.012G000700	Sapur.012G037400	Sapur.012G040700
Sapur.012G048900	Sapur.012G050600	Sapur.012G061900	Sapur.012G067300	Sapur.012G070700	Sapur.012G073300
Sapur.012G084200	Sapur.012G086700	Sapur.012G087900	Sapur.012G091400	Sapur.012G091800	Sapur.012G092600
Sapur.012G092900	Sapur.012G100600	Sapur.012G101100	Sapur.012G104300	Sapur.013G000100	Sapur.013G004800
Sapur.013G021200	Sapur.013G045400	Sapur.013G055900	Sapur.013G097100	Sapur.013G100200	Sapur.013G110800
Sapur.013G117900	Sapur.013G118200	Sapur.013G118400	Sapur.013G120100	Sapur.013G120600	Sapur.013G123000
Sapur.013G123300	Sapur.013G123700	Sapur.013G124400	Sapur.014G001300	Sapur.014G001700	Sapur.014G002600
Sapur.014G005100	Sapur.014G025600	Sapur.014G032900	Sapur.014G036100	Sapur.014G052300	Sapur.014G053000
Sapur.014G055200	Sapur.014G055500	Sapur.014G061800	Sapur.014G073900	Sapur.014G076200	Sapur.014G080400
Sapur.014G084500	Sapur.014G090200	Sapur.014G090300	Sapur.014G104700	Sapur.014G106600	Sapur.014G110600
Sapur.014G113100	Sapur.014G113600	Sapur.014G117400	Sapur.014G117700	Sapur.014G120400	Sapur.014G132000
Sapur.014G134700	Sapur.014G135900	Sapur.016G009300	Sapur.016G018900	Sapur.016G031400	Sapur.016G035600
Sapur.016G040000	Sapur.016G051900	Sapur.016G052700	Sapur.016G058000	Sapur.016G060400	Sapur.016G071200
Sapur.016G077600	Sapur.016G120800	Sapur.016G123500	Sapur.016G125300	Sapur.016G132300	Sapur.016G132400
Sapur.016G141300	Sapur.016G147900	Sapur.016G150200	Sapur.016G152700	Sapur.016G163400	Sapur.016G189500
Sapur.016G189900	Sapur.016G200900	Sapur.016G230700	Sapur.016G230800	Sapur.016G243200	Sapur.016G243400
Sapur.016G247200	Sapur.016G262400	Sapur.016G264800	Sapur.016G281900	Sapur.016G282700	Sapur.016G288500
Sapur.016G302100	Sapur.016G302500	Sapur.016G304100	Sapur.017G003500	Sapur.017G009300	Sapur.017G010500
Sapur.017G018700	Sapur.017G020600	Sapur.017G023600	Sapur.017G023800	Sapur.017G029100	Sapur.017G036400
Sapur.017G036800	Sapur.017G037700	Sapur.017G052400	Sapur.017G055300	Sapur.017G057300	Sapur.017G057900
Sapur.017G074500	Sapur.017G100600	Sapur.017G101100	Sapur.017G101200	Sapur.017G102500	Sapur.017G104800
Sapur.017G108100	Sapur.017G108200	Sapur.017G108700	Sapur.017G110500	Sapur.017G110700	Sapur.017G111200
Sapur.017G112200	Sapur.017G112400	Sapur.017G113400	Sapur.017G123300	Sapur.017G129000	Sapur.018G002400
Sapur.018G003500	Sapur.018G003800	Sapur.018G004300	Sapur.018G005400	Sapur.018G008500	Sapur.018G015000
Sapur.018G015800	Sapur.018G016300	Sapur.018G016500	Sapur.018G016600	Sapur.018G016800	Sapur.018G017000
Sapur.018G023200	Sapur.018G025700	Sapur.018G045400	Sapur.018G047200	Sapur.018G057100	Sapur.018G057600
Sapur.018G060400	Sapur.018G063600	Sapur.018G063900	Sapur.018G067200	Sapur.018G077200	Sapur.018G087600
Sapur.018G088200	Sapur.018G105800	Sapur.018G106800	Sapur.018G114900	Sapur.018G115800	Sapur.018G115900

				Sapur.018G117400 Sapur.019G001100 Sapur.019G003100 Sapur.019G005200 Sapur.019G005900 Sapur.019G007100 Sapur.019G007800 Sapur.019G007900 Sapur.019G009300 Sapur.019G009600 Sapur.019G010500 Sapur.019G010700 Sapur.019G011000 Sapur.019G011200 Sapur.019G011500 Sapur.019G011600 Sapur.019G011700 Sapur.019G011900 Sapur.019G012100 Sapur.019G012500 Sapur.019G012700 Sapur.019G012800 Sapur.019G013700 Sapur.019G016400 Sapur.019G017000 Sapur.019G022800 Sapur.019G023100 Sapur.019G027100 Sapur.019G027300 Sapur.019G028700 Sapur.019G029100 Sapur.019G034300 Sapur.019G042100 Sapur.019G042400 Sapur.019G042500 Sapur.019G043100 Sapur.019G043200 Sapur.019G044000 Sapur.019G044100 Sapur.019G046700 Sapur.019G048000 Sapur.019G048800 Sapur.019G053500 Sapur.019G058700 Sapur.019G070000 Sapur.019G075900 Sapur.019G080700 Sapur.019G082400 Sapur.019G091100 Sapur.019G091300 Sapur.019G091700 Sapur.019G092300 Sapur.019G092600 Sapur.019G092700 Sapur.019G092800 Sapur.019G093100 Sapur.019G093400 Sapur.019G093600 Sapur.019G093900 Sapur.019G094300 Sapur.019G107300 Sapur.019G110800 Sapur.019G111400 Sapur.019G113800 Sapur.15WG030400 Sapur.15WG036700 Sapur.15WG040400 Sapur.15WG043400 Sapur.15WG044400 Sapur.15WG076100 Sapur.15ZG018400 Sapur.15ZG038900 Sapur.15ZG044100 Sapur.15ZG044300 Sapur.15ZG044900 Sapur.15ZG049500 Sapur.15ZG050400 Sapur.15ZG050500 Sapur.15ZG078500 Sapur.15ZG103200 Sapur.15ZG109900 Sapur.15ZG116100 Sapur.15ZG120200 Sapur.15ZG123200 Sapur.15ZG126400 Sapur.T003900 Sapur.T005400 Sapur.T005500 Sapur.T028300 Sapur.T029400 Sapur.T030800 Sapur.T058800 Sapur.T080500 Sapur.T096600 Sapur.T115500 Sapur.T116500 Sapur.T117000 Sapur.T131900 Sapur.T133700 Sapur.T147200 Sapur.T150000 Sapur.T191500 Sapur.T192100
GO:0016491	326	1583	oxidoreductase activity	Sapur.001G004400 Sapur.001G004500 Sapur.001G070800 Sapur.001G081400 Sapur.001G092600 Sapur.001G141900 Sapur.001G162200 Sapur.001G191700 Sapur.002G022500 Sapur.002G055800 Sapur.002G056700 Sapur.002G066500 Sapur.002G071000 Sapur.002G115900 Sapur.002G127800 Sapur.002G128000 Sapur.002G152900 Sapur.002G153000 Sapur.002G158200 Sapur.002G176300 Sapur.002G198400 Sapur.003G003000 Sapur.003G015300 Sapur.003G038100 Sapur.003G046700 Sapur.003G090600 Sapur.003G090700 Sapur.003G104600 Sapur.003G166200 Sapur.004G001600 Sapur.004G023100 Sapur.004G028700 Sapur.004G055500 Sapur.004G055600 Sapur.004G117800 Sapur.004G144500 Sapur.004G144600 Sapur.004G169900 Sapur.005G046200 Sapur.005G055700 Sapur.005G089700 Sapur.005G090300 Sapur.005G090800 Sapur.005G090900 Sapur.005G095300 Sapur.005G115200 Sapur.005G119300 Sapur.005G176700 Sapur.005G185900 Sapur.005G196700 Sapur.006G021100 Sapur.006G027800 Sapur.006G101300 Sapur.006G113400 Sapur.006G118100 Sapur.006G118200 Sapur.006G126200 Sapur.006G182100 Sapur.006G187000 Sapur.006G219200 Sapur.006G220900 Sapur.007G014500 Sapur.007G032000 Sapur.007G049700 Sapur.007G052400 Sapur.007G089400 Sapur.007G098300 Sapur.007G108700 Sapur.008G022600 Sapur.008G080200 Sapur.008G156500 Sapur.008G161100 Sapur.009G018600 Sapur.009G039700 Sapur.009G079400 Sapur.009G084200 Sapur.009G084500 Sapur.009G120800 Sapur.010G076300 Sapur.010G081100 Sapur.010G138200 Sapur.010G138400 Sapur.010G199500 Sapur.011G015400 Sapur.011G027800 Sapur.011G032400 Sapur.011G036500 Sapur.011G071200 Sapur.011G077400 Sapur.011G087300 Sapur.011G109800 Sapur.011G111300 Sapur.011G111700 Sapur.011G116000 Sapur.012G002800

Sapur.012G063100 Sapur.012G082800 Sapur.012G103800 Sapur.012G103900 Sapur.012G110200 Sapur.012G110300
Sapur.013G054200 Sapur.013G056600 Sapur.013G097100 Sapur.013G116100 Sapur.013G140300 Sapur.014G055600
Sapur.014G061700 Sapur.014G068100 Sapur.014G107200 Sapur.014G107700 Sapur.014G107800 Sapur.014G129500
Sapur.016G018900 Sapur.016G022200 Sapur.016G023300 Sapur.016G055400 Sapur.016G056100 Sapur.016G060400
Sapur.016G061300 Sapur.016G071600 Sapur.016G073300 Sapur.016G097700 Sapur.016G102200 Sapur.016G144700
Sapur.016G162900 Sapur.016G184500 Sapur.016G184800 Sapur.016G202600 Sapur.016G216200 Sapur.016G226800
Sapur.016G226900 Sapur.016G241900 Sapur.016G245400 Sapur.016G249000 Sapur.016G253900 Sapur.016G261000
Sapur.016G301600 Sapur.017G001400 Sapur.017G010000 Sapur.017G028400 Sapur.017G028500 Sapur.017G036200
Sapur.017G037000 Sapur.017G037500 Sapur.017G037800 Sapur.017G039900 Sapur.017G055200 Sapur.017G112800
Sapur.017G123100 Sapur.018G011700 Sapur.018G015300 Sapur.018G045500 Sapur.018G047200 Sapur.018G095000
Sapur.018G095100 Sapur.019G009200 Sapur.019G009500 Sapur.019G010600 Sapur.019G011100 Sapur.019G011800
Sapur.019G012000 Sapur.019G012400 Sapur.019G033700 Sapur.019G041400 Sapur.019G042900 Sapur.019G070000
Sapur.019G084800 Sapur.019G084900 Sapur.15WG048400 Sapur.15ZG002400 Sapur.15ZG002500 Sapur.15ZG029600
Sapur.15ZG050200 Sapur.15ZG109600 Sapur.15ZG109800 Sapur.15ZG127500 Sapur.15ZG134000 Sapur.T046000
Sapur.T051400 Sapur.T175200 Sapur.T190200 Sapur.001G017800 Sapur.001G129600 Sapur.001G129800
Sapur.001G130300 Sapur.001G177000 Sapur.002G094800 Sapur.002G123400 Sapur.002G128100 Sapur.003G060900
Sapur.004G112400 Sapur.004G132200 Sapur.004G156500 Sapur.005G026800 Sapur.005G026900 Sapur.005G069000
Sapur.005G113600 Sapur.006G054500 Sapur.006G150600 Sapur.007G044500 Sapur.007G071200 Sapur.008G004900
Sapur.008G055000 Sapur.008G116400 Sapur.008G143900 Sapur.009G086900 Sapur.009G099800 Sapur.009G120300
Sapur.010G050700 Sapur.010G050800 Sapur.010G050900 Sapur.010G134600 Sapur.010G147100 Sapur.011G077900
Sapur.011G099900 Sapur.013G004800 Sapur.013G058600 Sapur.014G054300 Sapur.014G055200 Sapur.014G055500
Sapur.014G135900 Sapur.016G012200 Sapur.016G016700 Sapur.016G028600 Sapur.016G040000 Sapur.016G050900
Sapur.016G102700 Sapur.016G123800 Sapur.016G142600 Sapur.016G152700 Sapur.016G256900 Sapur.016G276900
Sapur.017G089700 Sapur.018G013500 Sapur.018G050900 Sapur.018G103000 Sapur.15WG030400 Sapur.15ZG111600
Sapur.T007600 Sapur.T045600 Sapur.002G133000 Sapur.004G108600 Sapur.005G203600 Sapur.007G095200
Sapur.009G070700 Sapur.014G114000 Sapur.001G067900 Sapur.001G148900 Sapur.001G149000 Sapur.001G149100
Sapur.001G167600 Sapur.001G193000 Sapur.002G019400 Sapur.002G106000 Sapur.002G107000 Sapur.002G141800
Sapur.003G003700 Sapur.003G037400 Sapur.003G037500 Sapur.003G082800 Sapur.003G093200 Sapur.003G109000
Sapur.003G130700 Sapur.004G088800 Sapur.004G096100 Sapur.004G139700 Sapur.004G148900 Sapur.006G045100
Sapur.006G045200 Sapur.006G098500 Sapur.006G116900 Sapur.006G121800 Sapur.006G186800 Sapur.007G078000
Sapur.007G080700 Sapur.007G080800 Sapur.007G103200 Sapur.008G166400 Sapur.009G030600 Sapur.009G053600
Sapur.009G085400 Sapur.009G085600 Sapur.009G085700 Sapur.009G086400 Sapur.010G021800 Sapur.010G022000
Sapur.010G088800 Sapur.010G088900 Sapur.010G109100 Sapur.010G109200 Sapur.010G149000 Sapur.010G198400

				Sapur.011G066700 Sapur.011G097000 Sapur.012G043000 Sapur.012G066500 Sapur.013G080500 Sapur.013G084400 Sapur.014G011100 Sapur.014G011200 Sapur.014G011900 Sapur.014G027000 Sapur.014G027100 Sapur.014G027200 Sapur.014G027400 Sapur.014G027800 Sapur.014G028400 Sapur.014G028500 Sapur.014G029000 Sapur.014G080900 Sapur.014G126900 Sapur.014G127800 Sapur.016G007300 Sapur.016G007700 Sapur.016G007900 Sapur.016G028200 Sapur.016G205100 Sapur.016G209200 Sapur.016G285400 Sapur.016G285500 Sapur.018G063600 Sapur.019G065400 Sapur.T047000 Sapur.T073900 Sapur.T131000
GO:0043168	525	2695	anion binding	Sapur.001G023800 Sapur.001G029800 Sapur.001G048800 Sapur.001G055600 Sapur.001G058300 Sapur.001G058800 Sapur.001G068000 Sapur.001G078400 Sapur.001G078600 Sapur.001G084200 Sapur.001G094200 Sapur.001G103000 Sapur.001G108900 Sapur.001G110300 Sapur.001G122200 Sapur.001G135100 Sapur.001G137000 Sapur.001G151600 Sapur.001G167000 Sapur.001G181600 Sapur.001G189000 Sapur.002G005300 Sapur.002G013600 Sapur.002G014300 Sapur.002G023300 Sapur.002G024400 Sapur.002G032100 Sapur.002G051400 Sapur.002G061600 Sapur.002G061700 Sapur.002G064800 Sapur.002G085200 Sapur.002G085900 Sapur.002G089300 Sapur.002G090300 Sapur.002G109500 Sapur.002G130500 Sapur.002G132500 Sapur.002G145300 Sapur.002G156100 Sapur.002G161200 Sapur.002G169300 Sapur.002G191900 Sapur.002G194100 Sapur.003G002000 Sapur.003G002100 Sapur.003G014300 Sapur.003G018100 Sapur.003G025000 Sapur.003G031900 Sapur.003G066000 Sapur.003G067100 Sapur.003G078600 Sapur.003G092400 Sapur.003G099500 Sapur.003G114500 Sapur.003G116100 Sapur.003G124900 Sapur.003G125800 Sapur.003G138500 Sapur.003G139700 Sapur.003G141900 Sapur.003G155400 Sapur.003G160700 Sapur.003G168000 Sapur.004G014400 Sapur.004G019100 Sapur.004G027800 Sapur.004G033200 Sapur.004G037400 Sapur.004G041000 Sapur.004G051400 Sapur.004G063300 Sapur.004G077700 Sapur.004G103000 Sapur.004G109700 Sapur.004G128600 Sapur.004G131500 Sapur.004G137600 Sapur.004G140300 Sapur.004G146400 Sapur.004G147000 Sapur.004G147900 Sapur.004G172100 Sapur.005G000100 Sapur.005G005000 Sapur.005G005700 Sapur.005G007100 Sapur.005G011200 Sapur.005G022300 Sapur.005G023100 Sapur.005G024800 Sapur.005G025000 Sapur.005G025400 Sapur.005G031700 Sapur.005G038400 Sapur.005G043600 Sapur.005G043700 Sapur.005G046200 Sapur.005G048900 Sapur.005G050200 Sapur.005G054500 Sapur.005G055500 Sapur.005G069000 Sapur.005G075800 Sapur.005G085100 Sapur.005G095700 Sapur.005G096300 Sapur.005G096400 Sapur.005G109500 Sapur.005G145700 Sapur.005G165600 Sapur.005G193700 Sapur.005G199500 Sapur.005G206400 Sapur.006G010200 Sapur.006G018700 Sapur.006G025100 Sapur.006G036800 Sapur.006G037200 Sapur.006G067400 Sapur.006G069500 Sapur.006G072700 Sapur.006G094200 Sapur.006G094300 Sapur.006G110200 Sapur.006G112500 Sapur.006G118100 Sapur.006G118200 Sapur.006G126200 Sapur.006G136200 Sapur.006G136800 Sapur.006G138300 Sapur.006G139300 Sapur.006G154200 Sapur.006G154300 Sapur.006G163000 Sapur.006G172300 Sapur.006G173100 Sapur.006G181100 Sapur.006G182100 Sapur.006G194600 Sapur.006G218800 Sapur.006G221300 Sapur.006G223700 Sapur.006G224200 Sapur.006G225500 Sapur.007G002900 Sapur.007G007000 Sapur.007G007100 Sapur.007G008900 Sapur.007G016800 Sapur.007G029800 Sapur.007G036500 Sapur.007G044500 Sapur.007G069600 Sapur.007G071200 Sapur.007G075800 Sapur.007G077000 Sapur.007G077600 Sapur.007G087100

Sapur.007G091900 Sapur.007G111300 Sapur.007G111400 Sapur.007G111700 Sapur.007G112200 Sapur.007G115600
Sapur.008G004700 Sapur.008G004900 Sapur.008G009100 Sapur.008G022900 Sapur.008G026800 Sapur.008G045900
Sapur.008G046000 Sapur.008G058400 Sapur.008G060600 Sapur.008G062600 Sapur.008G066900 Sapur.008G077900
Sapur.008G081500 Sapur.008G088300 Sapur.008G090300 Sapur.008G091700 Sapur.008G115400 Sapur.008G128300
Sapur.008G131900 Sapur.008G137200 Sapur.008G151300 Sapur.008G151900 Sapur.008G156200 Sapur.008G162000
Sapur.009G002900 Sapur.009G004200 Sapur.009G005200 Sapur.009G018000 Sapur.009G041000 Sapur.009G045200
Sapur.009G051100 Sapur.009G057000 Sapur.009G064100 Sapur.009G064400 Sapur.009G066300 Sapur.009G094100
Sapur.009G094200 Sapur.009G099800 Sapur.009G112500 Sapur.009G116000 Sapur.009G117400 Sapur.009G120100
Sapur.009G122200 Sapur.010G001900 Sapur.010G009400 Sapur.010G009500 Sapur.010G014000 Sapur.010G024600
Sapur.010G026800 Sapur.010G042700 Sapur.010G042900 Sapur.010G046700 Sapur.010G052700 Sapur.010G053300
Sapur.010G055900 Sapur.010G057900 Sapur.010G060700 Sapur.010G070700 Sapur.010G086100 Sapur.010G087600
Sapur.010G089800 Sapur.010G092300 Sapur.010G092700 Sapur.010G093900 Sapur.010G100000 Sapur.010G102500
Sapur.010G104500 Sapur.010G118700 Sapur.010G119700 Sapur.010G119800 Sapur.010G137200 Sapur.010G138500
Sapur.010G141100 Sapur.010G143600 Sapur.010G157400 Sapur.010G163200 Sapur.010G187200 Sapur.010G193600
Sapur.011G008400 Sapur.011G013300 Sapur.011G018500 Sapur.011G028400 Sapur.011G031900 Sapur.011G034200
Sapur.011G073000 Sapur.011G088900 Sapur.011G103500 Sapur.011G103700 Sapur.011G103800 Sapur.012G000700
Sapur.012G037400 Sapur.012G040700 Sapur.012G048900 Sapur.012G050600 Sapur.012G061900 Sapur.012G067300
Sapur.012G070700 Sapur.012G073300 Sapur.012G074000 Sapur.012G074300 Sapur.012G084200 Sapur.012G086700
Sapur.012G087900 Sapur.012G091400 Sapur.012G091800 Sapur.012G092600 Sapur.012G092900 Sapur.012G100600
Sapur.012G101100 Sapur.012G104300 Sapur.013G000100 Sapur.013G021200 Sapur.013G045400 Sapur.013G055900
Sapur.013G097100 Sapur.013G100200 Sapur.013G110800 Sapur.013G117900 Sapur.013G118200 Sapur.013G118400
Sapur.013G120100 Sapur.013G120600 Sapur.013G123000 Sapur.013G123300 Sapur.013G123700 Sapur.013G124400
Sapur.014G001300 Sapur.014G001700 Sapur.014G002600 Sapur.014G003400 Sapur.014G005100 Sapur.014G025600
Sapur.014G032900 Sapur.014G036100 Sapur.014G052300 Sapur.014G053000 Sapur.014G061800 Sapur.014G073900
Sapur.014G076200 Sapur.014G080400 Sapur.014G084500 Sapur.014G090200 Sapur.014G090300 Sapur.014G104700
Sapur.014G106600 Sapur.014G110600 Sapur.014G113100 Sapur.014G113600 Sapur.014G117400 Sapur.014G117700
Sapur.014G120400 Sapur.014G132000 Sapur.014G134700 Sapur.016G009300 Sapur.016G018900 Sapur.016G031400
Sapur.016G035600 Sapur.016G051900 Sapur.016G052700 Sapur.016G058000 Sapur.016G060400 Sapur.016G071200
Sapur.016G077600 Sapur.016G104800 Sapur.016G120800 Sapur.016G123500 Sapur.016G125300 Sapur.016G132300
Sapur.016G132400 Sapur.016G141300 Sapur.016G147900 Sapur.016G150200 Sapur.016G152700 Sapur.016G163400
Sapur.016G189500 Sapur.016G189900 Sapur.016G200900 Sapur.016G201800 Sapur.016G228700 Sapur.016G230700
Sapur.016G230800 Sapur.016G243200 Sapur.016G243400 Sapur.016G247200 Sapur.016G254800 Sapur.016G262400
Sapur.016G264800 Sapur.016G281900 Sapur.016G282700 Sapur.016G288500 Sapur.016G302100 Sapur.016G302500

				Sapur.017G003500 Sapur.017G009300 Sapur.017G010500 Sapur.017G018700 Sapur.017G020600 Sapur.017G023600 Sapur.017G023800 Sapur.017G029100 Sapur.017G036400 Sapur.017G036800 Sapur.017G037700 Sapur.017G052400 Sapur.017G055300 Sapur.017G057300 Sapur.017G057900 Sapur.017G074500 Sapur.017G100600 Sapur.017G101100 Sapur.017G101200 Sapur.017G102500 Sapur.017G104800 Sapur.017G108100 Sapur.017G108200 Sapur.017G108700 Sapur.017G110500 Sapur.017G110700 Sapur.017G111200 Sapur.017G112200 Sapur.017G112400 Sapur.017G113400 Sapur.017G123300 Sapur.017G124700 Sapur.017G129000 Sapur.018G002400 Sapur.018G003500 Sapur.018G003800 Sapur.018G004300 Sapur.018G008500 Sapur.018G015000 Sapur.018G015800 Sapur.018G016300 Sapur.018G016500 Sapur.018G016600 Sapur.018G016800 Sapur.018G017000 Sapur.018G023200 Sapur.018G025700 Sapur.018G045400 Sapur.018G047200 Sapur.018G057100 Sapur.018G057600 Sapur.018G060400 Sapur.018G063900 Sapur.018G067200 Sapur.018G077200 Sapur.018G087600 Sapur.018G088200 Sapur.018G105800 Sapur.018G114900 Sapur.018G115800 Sapur.018G115900 Sapur.018G117400 Sapur.019G001100 Sapur.019G003100 Sapur.019G005200 Sapur.019G005900 Sapur.019G007100 Sapur.019G007800 Sapur.019G007900 Sapur.019G009300 Sapur.019G009600 Sapur.019G010500 Sapur.019G010700 Sapur.019G011000 Sapur.019G011200 Sapur.019G011500 Sapur.019G011600 Sapur.019G011700 Sapur.019G011900 Sapur.019G012100 Sapur.019G012500 Sapur.019G012700 Sapur.019G012800 Sapur.019G013700 Sapur.019G016400 Sapur.019G017000 Sapur.019G022800 Sapur.019G023100 Sapur.019G027100 Sapur.019G027300 Sapur.019G027700 Sapur.019G028700 Sapur.019G029100 Sapur.019G034300 Sapur.019G042100 Sapur.019G042400 Sapur.019G042500 Sapur.019G043100 Sapur.019G043200 Sapur.019G044000 Sapur.019G044100 Sapur.019G046700 Sapur.019G048000 Sapur.019G048800 Sapur.019G053500 Sapur.019G058700 Sapur.019G070000 Sapur.019G075900 Sapur.019G080700 Sapur.019G082400 Sapur.019G091100 Sapur.019G091300 Sapur.019G091700 Sapur.019G092300 Sapur.019G092600 Sapur.019G092700 Sapur.019G092800 Sapur.019G093100 Sapur.019G093400 Sapur.019G093600 Sapur.019G093900 Sapur.019G094300 Sapur.019G107300 Sapur.019G110800 Sapur.019G111400 Sapur.019G113800 Sapur.15WG036700 Sapur.15WG040400 Sapur.15WG043400 Sapur.15WG044400 Sapur.15WG076100 Sapur.15ZG018400 Sapur.15ZG038900 Sapur.15ZG044100 Sapur.15ZG044300 Sapur.15ZG044900 Sapur.15ZG049500 Sapur.15ZG050400 Sapur.15ZG050500 Sapur.15ZG078500 Sapur.15ZG099200 Sapur.15ZG103200 Sapur.15ZG109900 Sapur.15ZG116100 Sapur.15ZG120200 Sapur.15ZG123100 Sapur.15ZG123200 Sapur.15ZG126400 Sapur.T003900 Sapur.T005400 Sapur.T005500 Sapur.T028300 Sapur.T029400 Sapur.T030800 Sapur.T058800 Sapur.T080500 Sapur.T096600 Sapur.T115500 Sapur.T116500 Sapur.T117000 Sapur.T131900 Sapur.T133700 Sapur.T147200 Sapur.T150000 Sapur.T191500 Sapur.T192100
GO:0003677	290	1390	DNA binding	Sapur.001G013800 Sapur.001G025400 Sapur.001G047800 Sapur.001G055600 Sapur.001G058800 Sapur.001G067700 Sapur.001G069800 Sapur.001G072500 Sapur.001G090900 Sapur.001G119600 Sapur.001G129000 Sapur.001G151600 Sapur.001G166100 Sapur.001G178100 Sapur.001G179000 Sapur.002G021500 Sapur.002G023100 Sapur.002G028500 Sapur.002G031600 Sapur.002G035300 Sapur.002G043300 Sapur.002G064600 Sapur.002G069500 Sapur.002G080700 Sapur.002G091700 Sapur.002G110600 Sapur.002G116100 Sapur.002G125900 Sapur.002G126000

Sapur.002G136900 Sapur.002G142300 Sapur.002G145300 Sapur.002G160400 Sapur.003G016500 Sapur.003G025800
Sapur.003G047900 Sapur.003G057500 Sapur.003G068400 Sapur.003G123800 Sapur.003G129600 Sapur.003G152800
Sapur.003G159400 Sapur.004G030500 Sapur.004G030800 Sapur.004G030900 Sapur.004G034900 Sapur.004G054800
Sapur.004G056900 Sapur.004G062200 Sapur.004G089500 Sapur.004G101700 Sapur.004G103000 Sapur.004G110300
Sapur.004G121800 Sapur.004G137000 Sapur.005G000100 Sapur.005G005600 Sapur.005G019900 Sapur.005G032200
Sapur.005G052900 Sapur.005G054800 Sapur.005G093100 Sapur.005G105500 Sapur.005G141300 Sapur.005G163900
Sapur.005G186700 Sapur.006G026800 Sapur.006G043000 Sapur.006G063200 Sapur.006G065100 Sapur.006G068600
Sapur.006G103400 Sapur.006G104700 Sapur.006G114200 Sapur.006G118700 Sapur.006G166000 Sapur.006G170000
Sapur.006G194100 Sapur.006G214400 Sapur.007G034000 Sapur.007G035800 Sapur.007G058700 Sapur.007G087800
Sapur.007G089800 Sapur.007G091900 Sapur.007G094000 Sapur.008G021500 Sapur.008G042700 Sapur.008G053100
Sapur.008G060600 Sapur.008G064800 Sapur.008G066900 Sapur.008G069200 Sapur.008G090400 Sapur.008G093800
Sapur.008G094300 Sapur.008G104300 Sapur.008G119700 Sapur.008G131800 Sapur.009G002900 Sapur.009G005300
Sapur.009G009700 Sapur.009G011600 Sapur.009G021700 Sapur.009G045200 Sapur.009G080900 Sapur.009G104900
Sapur.009G112800 Sapur.009G114600 Sapur.009G126000 Sapur.010G035800 Sapur.010G035900 Sapur.010G053800
Sapur.010G078000 Sapur.010G099700 Sapur.010G100200 Sapur.010G118700 Sapur.010G120000 Sapur.010G120300
Sapur.010G128300 Sapur.010G141100 Sapur.010G149100 Sapur.010G154300 Sapur.010G161100 Sapur.010G185200
Sapur.010G193700 Sapur.011G032100 Sapur.011G037100 Sapur.011G037900 Sapur.011G038200 Sapur.011G041100
Sapur.011G045800 Sapur.011G056100 Sapur.011G088400 Sapur.011G088500 Sapur.011G101200 Sapur.012G004000
Sapur.012G010400 Sapur.012G017700 Sapur.012G018600 Sapur.012G024500 Sapur.012G051400 Sapur.012G060100
Sapur.012G078700 Sapur.012G105100 Sapur.013G000100 Sapur.013G008000 Sapur.013G028900 Sapur.013G050700
Sapur.013G050900 Sapur.013G068800 Sapur.013G117300 Sapur.014G026600 Sapur.014G034500 Sapur.014G059000
Sapur.014G059100 Sapur.014G059200 Sapur.014G075400 Sapur.014G076200 Sapur.014G078500 Sapur.014G079300
Sapur.014G085400 Sapur.014G085500 Sapur.014G142400 Sapur.016G018700 Sapur.016G026800 Sapur.016G030200
Sapur.016G043500 Sapur.016G064600 Sapur.016G070400 Sapur.016G085100 Sapur.016G114700 Sapur.016G131300
Sapur.016G167800 Sapur.016G183700 Sapur.016G199700 Sapur.016G216900 Sapur.016G229800 Sapur.016G264800
Sapur.017G053300 Sapur.017G069900 Sapur.017G085900 Sapur.017G114800 Sapur.017G115700 Sapur.018G015800
Sapur.018G019900 Sapur.018G087600 Sapur.018G107600 Sapur.019G033200 Sapur.019G039900 Sapur.019G060100
Sapur.019G074500 Sapur.019G099800 Sapur.019G101700 Sapur.15WG065100 Sapur.15WG072800 Sapur.15ZG002000
Sapur.15ZG007300 Sapur.15ZG016900 Sapur.15ZG043500 Sapur.15ZG053200 Sapur.15ZG067200 Sapur.15ZG079000
Sapur.15ZG080900 Sapur.15ZG103400 Sapur.T012000 Sapur.001G017300 Sapur.001G027900 Sapur.001G028100
Sapur.001G028400 Sapur.001G034200 Sapur.001G179800 Sapur.002G012900 Sapur.002G024200 Sapur.002G033200
Sapur.002G073000 Sapur.002G146000 Sapur.002G163600 Sapur.002G175800 Sapur.003G102000 Sapur.003G125900
Sapur.003G138200 Sapur.003G145700 Sapur.003G150800 Sapur.004G007000 Sapur.004G027800 Sapur.004G118800

				Sapur.004G121500 Sapur.004G123100 Sapur.004G131600 Sapur.004G171400 Sapur.005G067000 Sapur.005G087400 Sapur.005G098100 Sapur.005G133200 Sapur.005G150000 Sapur.006G067500 Sapur.006G089400 Sapur.006G110100 Sapur.006G122700 Sapur.006G168000 Sapur.006G218000 Sapur.006G228700 Sapur.006G228900 Sapur.007G001300 Sapur.007G021900 Sapur.007G044600 Sapur.007G062200 Sapur.008G081900 Sapur.008G128300 Sapur.009G094800 Sapur.009G097900 Sapur.010G003100 Sapur.010G046700 Sapur.010G065900 Sapur.010G114700 Sapur.010G123400 Sapur.011G006600 Sapur.011G035500 Sapur.011G049300 Sapur.011G054600 Sapur.011G061100 Sapur.012G000700 Sapur.012G031400 Sapur.013G010700 Sapur.013G039500 Sapur.013G055500 Sapur.013G087600 Sapur.013G137400 Sapur.014G003700 Sapur.014G005200 Sapur.014G094500 Sapur.014G095700 Sapur.014G099500 Sapur.014G104700 Sapur.016G079900 Sapur.016G080200 Sapur.016G111900 Sapur.016G159000 Sapur.016G224700 Sapur.016G298800 Sapur.016G299400 Sapur.017G057100 Sapur.018G007100 Sapur.018G025000 Sapur.018G035300 Sapur.018G056400 Sapur.018G057700 Sapur.019G075900 Sapur.019G108100
GO:0015291	34	102	secondary active transmembrane transporter activity	Sapur.001G094500 Sapur.002G043400 Sapur.002G086100 Sapur.003G081500 Sapur.004G009600 Sapur.004G009700 Sapur.004G138100 Sapur.005G082300 Sapur.005G114300 Sapur.005G163600 Sapur.005G163800 Sapur.008G101500 Sapur.008G101600 Sapur.008G101700 Sapur.009G040500 Sapur.009G097300 Sapur.010G024300 Sapur.010G089300 Sapur.011G001000 Sapur.011G080900 Sapur.012G102500 Sapur.012G111400 Sapur.013G071900 Sapur.013G072500 Sapur.013G072600 Sapur.013G072700 Sapur.016G153700 Sapur.019G046000 Sapur.019G064700 Sapur.019G083100 Sapur.003G031900 Sapur.005G061600 Sapur.005G131700 Sapur.007G081600
GO:0022804	57	204	active transmembrane transporter activity	Sapur.001G078400 Sapur.001G094500 Sapur.002G043400 Sapur.002G086100 Sapur.002G132500 Sapur.002G156100 Sapur.003G025000 Sapur.003G081500 Sapur.003G099500 Sapur.003G138500 Sapur.004G008500 Sapur.004G009600 Sapur.004G009700 Sapur.004G138100 Sapur.004G173400 Sapur.005G082300 Sapur.005G114300 Sapur.005G163600 Sapur.005G163800 Sapur.008G101500 Sapur.008G101600 Sapur.008G101700 Sapur.009G040500 Sapur.009G097300 Sapur.010G001900 Sapur.010G024300 Sapur.010G024600 Sapur.010G089300 Sapur.011G001000 Sapur.011G014000 Sapur.011G028400 Sapur.011G080900 Sapur.012G102500 Sapur.012G111400 Sapur.013G071900 Sapur.013G072500 Sapur.013G072600 Sapur.013G072700 Sapur.014G090200 Sapur.014G090300 Sapur.016G153700 Sapur.016G230700 Sapur.016G230800 Sapur.017G057900 Sapur.018G063900 Sapur.019G046000 Sapur.019G064700 Sapur.019G083100 Sapur.019G113800 Sapur.15ZG018400 Sapur.15ZG126400 Sapur.T005400 Sapur.T005500 Sapur.003G031900 Sapur.005G061600 Sapur.005G131700 Sapur.007G081600
GO:0003700	134	584	DNA-binding transcription factor activity	Sapur.001G017300 Sapur.001G034200 Sapur.001G064300 Sapur.001G067700 Sapur.001G075700 Sapur.001G129000 Sapur.001G130000 Sapur.001G163400 Sapur.002G012900 Sapur.002G024200 Sapur.002G030000 Sapur.002G030100 Sapur.002G033200 Sapur.002G073000 Sapur.002G080700 Sapur.002G110600 Sapur.002G115400 Sapur.002G143500 Sapur.002G146000 Sapur.002G163600 Sapur.002G175800 Sapur.002G190900 Sapur.003G017600 Sapur.003G047900 Sapur.003G049200 Sapur.003G099900 Sapur.003G102000 Sapur.003G111600 Sapur.003G125900 Sapur.003G138200 Sapur.003G150800 Sapur.004G031600 Sapur.004G118800 Sapur.004G121500 Sapur.004G123100

				Sapur.004G131600 Sapur.004G171400 Sapur.005G054800 Sapur.005G067000 Sapur.005G087400 Sapur.005G098100 Sapur.005G108500 Sapur.005G133200 Sapur.005G150000 Sapur.006G041100 Sapur.006G067500 Sapur.006G089400 Sapur.006G103400 Sapur.006G110100 Sapur.006G120000 Sapur.006G122700 Sapur.006G137200 Sapur.006G194100 Sapur.006G197300 Sapur.006G218000 Sapur.006G228700 Sapur.006G228900 Sapur.007G005500 Sapur.007G021900 Sapur.007G044600 Sapur.007G082500 Sapur.007G087800 Sapur.007G123400 Sapur.008G034200 Sapur.008G081900 Sapur.008G094300 Sapur.008G096700 Sapur.008G104300 Sapur.008G119700 Sapur.008G134800 Sapur.008G160400 Sapur.009G040000 Sapur.009G094800 Sapur.009G097900 Sapur.009G126600 Sapur.010G003100 Sapur.010G005200 Sapur.010G040000 Sapur.010G040100 Sapur.010G065900 Sapur.010G096300 Sapur.010G099700 Sapur.010G114700 Sapur.010G123400 Sapur.011G006600 Sapur.011G035500 Sapur.011G039300 Sapur.011G042400 Sapur.011G049300 Sapur.011G061100 Sapur.011G066500 Sapur.011G079300 Sapur.012G018600 Sapur.012G051400 Sapur.013G039500 Sapur.013G041800 Sapur.013G055500 Sapur.013G087600 Sapur.013G137400 Sapur.013G141800 Sapur.014G003700 Sapur.014G004000 Sapur.014G005200 Sapur.014G034500 Sapur.014G078300 Sapur.014G094500 Sapur.014G095700 Sapur.014G099500 Sapur.014G101000 Sapur.016G018700 Sapur.016G079900 Sapur.016G080200 Sapur.016G087700 Sapur.016G111900 Sapur.016G153200 Sapur.016G159000 Sapur.016G167800 Sapur.016G224700 Sapur.016G255700 Sapur.016G298800 Sapur.017G007100 Sapur.018G007100 Sapur.018G025000 Sapur.018G034800 Sapur.018G035300 Sapur.018G057700 Sapur.018G067800 Sapur.019G025000 Sapur.019G074500 Sapur.019G098000 Sapur.019G108100 Sapur.15ZG016900 Sapur.15ZG067200 Sapur.15ZG071900
GO:0140110	140	619	transcription regulator activity	Sapur.001G017300 Sapur.001G034200 Sapur.001G064300 Sapur.001G067700 Sapur.001G075700 Sapur.001G129000 Sapur.001G130000 Sapur.001G163400 Sapur.002G012900 Sapur.002G024200 Sapur.002G030000 Sapur.002G030100 Sapur.002G033200 Sapur.002G073000 Sapur.002G080700 Sapur.002G110600 Sapur.002G115400 Sapur.002G143500 Sapur.002G146000 Sapur.002G163600 Sapur.002G175800 Sapur.002G190900 Sapur.003G017600 Sapur.003G047900 Sapur.003G049200 Sapur.003G099900 Sapur.003G102000 Sapur.003G111600 Sapur.003G125900 Sapur.003G138200 Sapur.003G150800 Sapur.004G031600 Sapur.004G118800 Sapur.004G121500 Sapur.004G123100 Sapur.004G131600 Sapur.004G171400 Sapur.005G054800 Sapur.005G055100 Sapur.005G067000 Sapur.005G087400 Sapur.005G098100 Sapur.005G108500 Sapur.005G133200 Sapur.005G150000 Sapur.006G041100 Sapur.006G067500 Sapur.006G089400 Sapur.006G103400 Sapur.006G110100 Sapur.006G120000 Sapur.006G122700 Sapur.006G137200 Sapur.006G194100 Sapur.006G197300 Sapur.006G218000 Sapur.006G228700 Sapur.006G228900 Sapur.007G005500 Sapur.007G021900 Sapur.007G044600 Sapur.007G050900 Sapur.007G082500 Sapur.007G087500 Sapur.007G087800 Sapur.007G098200 Sapur.007G123400 Sapur.008G034200 Sapur.008G081900 Sapur.008G094300 Sapur.008G096700 Sapur.008G104300 Sapur.008G119700 Sapur.008G134800 Sapur.008G160400 Sapur.009G040000 Sapur.009G094800 Sapur.009G097900 Sapur.009G126600 Sapur.010G003100 Sapur.010G005200 Sapur.010G040000 Sapur.010G040100 Sapur.010G065900 Sapur.010G096300 Sapur.010G099700 Sapur.010G114700 Sapur.010G123400 Sapur.011G006600 Sapur.011G035500 Sapur.011G039300 Sapur.011G042400 Sapur.011G049300 Sapur.011G061100 Sapur.011G066500

				Sapur.011G079300 Sapur.012G018600 Sapur.012G051400 Sapur.012G067900 Sapur.013G039500 Sapur.013G041800 Sapur.013G055500 Sapur.013G087600 Sapur.013G137400 Sapur.013G141800 Sapur.014G003700 Sapur.014G004000 Sapur.014G005200 Sapur.014G034500 Sapur.014G078300 Sapur.014G094500 Sapur.014G095700 Sapur.014G099500 Sapur.014G101000 Sapur.016G018700 Sapur.016G079900 Sapur.016G080200 Sapur.016G087700 Sapur.016G111900 Sapur.016G153200 Sapur.016G159000 Sapur.016G167800 Sapur.016G224700 Sapur.016G255700 Sapur.016G298800 Sapur.017G004800 Sapur.017G007100 Sapur.018G007100 Sapur.018G025000 Sapur.018G034800 Sapur.018G035300 Sapur.018G057700 Sapur.018G067800 Sapur.019G025000 Sapur.019G074500 Sapur.019G098000 Sapur.019G108100 Sapur.15ZG016900 Sapur.15ZG067200 Sapur.15ZG071900
GO:0005215	161	729	transporter activity	Sapur.001G006200 Sapur.003G069300 Sapur.010G194900 Sapur.012G100800 Sapur.013G059700<br>Sapur.001G026600 Sapur.001G034600 Sapur.001G036000 Sapur.001G053700 Sapur.001G058900 Sapur.001G078400 Sapur.001G094500 Sapur.001G120100 Sapur.001G122300 Sapur.001G139700 Sapur.001G164100 Sapur.002G004100 Sapur.002G043400 Sapur.002G067900 Sapur.002G079800 Sapur.002G086100 Sapur.002G132500 Sapur.002G156100 Sapur.002G201900 Sapur.003G025000 Sapur.003G081500 Sapur.003G099500 Sapur.003G137300 Sapur.003G138500 Sapur.003G148600 Sapur.003G152900 Sapur.004G008500 Sapur.004G009600 Sapur.004G009700 Sapur.004G019600 Sapur.004G043800 Sapur.004G138100 Sapur.004G157800 Sapur.004G173400 Sapur.005G009200 Sapur.005G061600 Sapur.005G082300 Sapur.005G114000 Sapur.005G114100 Sapur.005G114300 Sapur.005G131700 Sapur.005G137600 Sapur.005G137700 Sapur.005G142100 Sapur.005G163600 Sapur.005G163800 Sapur.005G177000 Sapur.005G204900 Sapur.006G010900 Sapur.006G023600 Sapur.006G079600 Sapur.006G080400 Sapur.006G100000 Sapur.007G081600 Sapur.008G051900 Sapur.008G101500 Sapur.008G101600 Sapur.008G101700 Sapur.008G118300 Sapur.008G134200 Sapur.008G138300 Sapur.009G002500 Sapur.009G031200 Sapur.009G034100 Sapur.009G040500 Sapur.009G097300 Sapur.010G001900 Sapur.010G024300 Sapur.010G024600 Sapur.010G077300 Sapur.010G089300 Sapur.010G137200 Sapur.010G164900 Sapur.011G001000 Sapur.011G014000 Sapur.011G028400 Sapur.011G047400 Sapur.011G080900 Sapur.011G110300 Sapur.012G050600 Sapur.012G051800 Sapur.012G102500 Sapur.012G111400 Sapur.013G071900 Sapur.013G072500 Sapur.013G072600 Sapur.013G072700 Sapur.013G108500 Sapur.014G085900 Sapur.014G090200 Sapur.014G090300 Sapur.014G128400 Sapur.014G128800 Sapur.016G028400 Sapur.016G084700 Sapur.016G092200 Sapur.016G106100 Sapur.016G153700 Sapur.016G194200 Sapur.016G223600 Sapur.016G224500 Sapur.016G230700 Sapur.016G230800 Sapur.016G292400 Sapur.017G057900 Sapur.017G063700 Sapur.018G033700 Sapur.018G063900 Sapur.019G046000 Sapur.019G054300 Sapur.019G064700 Sapur.019G076400 Sapur.019G083100 Sapur.019G113800 Sapur.15ZG018400 Sapur.15ZG074200 Sapur.15ZG126400 Sapur.T005400 Sapur.T005500 Sapur.T148900<br>Sapur.001G137400 Sapur.002G055900 Sapur.002G177500 Sapur.002G177600 Sapur.003G031900 Sapur.006G076500 Sapur.006G206900 Sapur.008G065700 Sapur.008G096200 Sapur.008G137000 Sapur.009G028800 Sapur.009G028900 Sapur.009G057800 Sapur.011G043000 Sapur.011G043200 Sapur.012G017000 Sapur.012G025300 Sapur.012G028700 Sapur.012G055400 Sapur.014G124400 Sapur.016G225300 Sapur.016G239700 Sapur.016G266000 Sapur.018G009000

GO:0008017	38	123	microtubule binding	Sapur.018G022500 Sapur.018G026000 Sapur.15ZG026500 Sapur.001G008300 Sapur.001G054900 Sapur.005G122600 Sapur.008G066200 Sapur.008G163400 Sapur.009G057300 Sapur.010G133500 Sapur.017G059600 Sapur.018G103300 Sapur.001G094200 Sapur.002G064800 Sapur.002G085200 Sapur.002G089300 Sapur.004G019100 Sapur.004G109700 Sapur.004G147000 Sapur.005G022300 Sapur.005G124600 Sapur.006G036800 Sapur.006G067400 Sapur.006G069500 Sapur.006G112500 Sapur.006G172300 Sapur.007G045800 Sapur.008G137200 Sapur.009G122200 Sapur.010G042700 Sapur.011G018500 Sapur.011G103700 Sapur.012G037400 Sapur.012G040700 Sapur.012G050300 Sapur.012G099300 Sapur.014G005100 Sapur.014G051500 Sapur.014G052300 Sapur.016G071200 Sapur.018G057600 Sapur.15WG040400 Sapur.15WG044400 Sapur.15WG081000 Sapur.15ZG044100 Sapur.15ZG044300 Sapur.15ZG044900 Sapur.15ZG049500 Sapur.15ZG059500 Sapur.T003900
GO:0043565	76	300	sequence-specific DNA binding	Sapur.001G017300 Sapur.001G034200 Sapur.001G067700 Sapur.001G129000 Sapur.002G012900 Sapur.002G024200 Sapur.002G033200 Sapur.002G073000 Sapur.002G080700 Sapur.002G110600 Sapur.002G146000 Sapur.002G163600 Sapur.002G175800 Sapur.003G047900 Sapur.003G102000 Sapur.003G125900 Sapur.003G138200 Sapur.003G150800 Sapur.004G118800 Sapur.004G121500 Sapur.004G123100 Sapur.004G131600 Sapur.004G171400 Sapur.005G054800 Sapur.005G067000 Sapur.005G087400 Sapur.005G098100 Sapur.005G133200 Sapur.005G150000 Sapur.006G067500 Sapur.006G089400 Sapur.006G110100 Sapur.006G122700 Sapur.006G218000 Sapur.006G228700 Sapur.006G228900 Sapur.007G021900 Sapur.007G044600 Sapur.007G087800 Sapur.008G081900 Sapur.008G104300 Sapur.008G119700 Sapur.009G094800 Sapur.009G097900 Sapur.010G003100 Sapur.010G065900 Sapur.010G114700 Sapur.010G123400 Sapur.011G006600 Sapur.011G035500 Sapur.011G049300 Sapur.011G061100 Sapur.012G051400 Sapur.013G039500 Sapur.013G055500 Sapur.013G087600 Sapur.013G137400 Sapur.014G003700 Sapur.014G005200 Sapur.014G034500 Sapur.014G094500 Sapur.014G095700 Sapur.014G099500 Sapur.016G079900 Sapur.016G080200 Sapur.016G111900 Sapur.016G159000 Sapur.016G224700 Sapur.016G298800 Sapur.018G007100 Sapur.018G025000 Sapur.018G035300 Sapur.018G056400 Sapur.018G057700 Sapur.019G108100 Sapur.15ZG067200
GO:0015631	38	124	tubulin binding	Sapur.001G094200 Sapur.002G064800 Sapur.002G085200 Sapur.002G089300 Sapur.004G019100 Sapur.004G109700 Sapur.004G147000 Sapur.005G022300 Sapur.005G124600 Sapur.006G036800 Sapur.006G067400 Sapur.006G069500 Sapur.006G112500 Sapur.006G172300 Sapur.007G045800 Sapur.008G137200 Sapur.009G122200 Sapur.010G042700 Sapur.011G018500 Sapur.011G103700 Sapur.012G037400 Sapur.012G040700 Sapur.012G050300 Sapur.012G099300 Sapur.014G005100 Sapur.014G051500 Sapur.014G052300 Sapur.016G071200 Sapur.018G057600 Sapur.15WG040400 Sapur.15WG044400 Sapur.15WG081000 Sapur.15ZG044100 Sapur.15ZG044300 Sapur.15ZG044900 Sapur.15ZG049500 Sapur.15ZG059500 Sapur.T003900
GO:0140657	107	460	ATP-dependent activity	Sapur.001G078400 Sapur.002G132500 Sapur.002G156100 Sapur.002G169300 Sapur.003G018100 Sapur.003G025000 Sapur.003G099500 Sapur.003G125800 Sapur.003G138500 Sapur.004G027800 Sapur.005G048900 Sapur.006G094300 Sapur.006G163000 Sapur.007G075800 Sapur.008G077900 Sapur.008G091700 Sapur.009G004200 Sapur.010G001900 Sapur.010G024600 Sapur.010G055900 Sapur.010G087600 Sapur.010G102500 Sapur.010G119700

				Sapur.010G119800 Sapur.010G137200 Sapur.011G028400 Sapur.012G050600 Sapur.012G084200 Sapur.014G061800 Sapur.014G090200 Sapur.014G090300 Sapur.014G113100 Sapur.016G141300 Sapur.016G195300 Sapur.016G230700 Sapur.016G230800 Sapur.017G029100 Sapur.017G055300 Sapur.017G057900 Sapur.017G074500 Sapur.017G129000 Sapur.018G063900 Sapur.019G048800 Sapur.019G080700 Sapur.019G113800 Sapur.15ZG018400 Sapur.15ZG103200 Sapur.15ZG109900 Sapur.15ZG126400 Sapur.T005400 Sapur.T005500 Sapur.T080500 Sapur.001G055600 Sapur.001G058800 Sapur.001G094200 Sapur.002G064800 Sapur.002G085200 Sapur.002G089300 Sapur.004G008500 Sapur.004G019100 Sapur.004G109700 Sapur.004G147000 Sapur.004G173400 Sapur.005G000100 Sapur.005G022300 Sapur.005G045400 Sapur.005G054500 Sapur.006G025100 Sapur.006G036800 Sapur.006G067400 Sapur.006G069500 Sapur.006G112500 Sapur.006G172300 Sapur.006G195300 Sapur.008G137200 Sapur.009G122200 Sapur.010G042700 Sapur.010G052700 Sapur.010G089800 Sapur.010G119100 Sapur.011G014000 Sapur.011G018500 Sapur.011G103700 Sapur.012G037400 Sapur.012G040700 Sapur.012G100600 Sapur.013G000100 Sapur.013G100200 Sapur.013G135300 Sapur.014G005100 Sapur.014G052300 Sapur.014G106600 Sapur.016G071200 Sapur.016G299400 Sapur.018G057600 Sapur.15WG040400 Sapur.15WG044400 Sapur.15ZG020500 Sapur.15ZG037200 Sapur.15ZG038900 Sapur.15ZG044100 Sapur.15ZG044300 Sapur.15ZG044900 Sapur.15ZG049500 Sapur.T003900 Sapur.T058800 Sapur.009G045200
GO:0003777	32	106	microtubule motor activity	Sapur.001G094200 Sapur.002G064800 Sapur.002G085200 Sapur.002G089300 Sapur.004G019100 Sapur.004G109700 Sapur.004G147000 Sapur.005G022300 Sapur.006G036800 Sapur.006G067400 Sapur.006G069500 Sapur.006G112500 Sapur.006G172300 Sapur.008G137200 Sapur.009G122200 Sapur.010G042700 Sapur.010G119100 Sapur.011G018500 Sapur.011G103700 Sapur.012G037400 Sapur.012G040700 Sapur.014G005100 Sapur.014G052300 Sapur.016G071200 Sapur.018G057600 Sapur.15WG040400 Sapur.15WG044400 Sapur.15ZG044100 Sapur.15ZG044300 Sapur.15ZG044900 Sapur.15ZG049500 Sapur.T003900
GO:0016887	52	199	ATP hydrolysis activity	Sapur.001G078400 Sapur.002G132500 Sapur.002G156100 Sapur.002G169300 Sapur.003G018100 Sapur.003G025000 Sapur.003G099500 Sapur.003G125800 Sapur.003G138500 Sapur.004G027800 Sapur.005G048900 Sapur.006G094300 Sapur.006G163000 Sapur.007G075800 Sapur.008G077900 Sapur.008G091700 Sapur.009G004200 Sapur.010G001900 Sapur.010G024600 Sapur.010G055900 Sapur.010G087600 Sapur.010G102500 Sapur.010G119700 Sapur.010G119800 Sapur.010G137200 Sapur.011G028400 Sapur.012G050600 Sapur.012G084200 Sapur.014G061800 Sapur.014G090200 Sapur.014G090300 Sapur.014G113100 Sapur.016G141300 Sapur.016G195300 Sapur.016G230700 Sapur.016G230800 Sapur.017G029100 Sapur.017G055300 Sapur.017G057900 Sapur.017G074500 Sapur.017G129000 Sapur.018G063900 Sapur.019G048800 Sapur.019G080700 Sapur.019G113800 Sapur.15ZG018400 Sapur.15ZG103200 Sapur.15ZG109900 Sapur.15ZG126400 Sapur.T005400 Sapur.T005500 Sapur.T080500
GO:0003774	35	121	cytoskeletal motor activity	Sapur.001G094200 Sapur.002G064800 Sapur.002G085200 Sapur.002G089300 Sapur.004G019100 Sapur.004G109700 Sapur.004G147000 Sapur.005G022300 Sapur.006G036800 Sapur.006G067400 Sapur.006G069500 Sapur.006G112500 Sapur.006G172300 Sapur.008G137200 Sapur.009G122200 Sapur.010G042700 Sapur.010G119100 Sapur.011G018500 Sapur.011G103700 Sapur.012G037400 Sapur.012G040700 Sapur.014G005100 Sapur.014G052300

				Sapur.016G071200 Sapur.018G057600 Sapur.15WG040400 Sapur.15WG044400 Sapur.15ZG044100 Sapur.15ZG044300 Sapur.15ZG044900 Sapur.15ZG049500 Sapur.T003900 Sapur.001G167000 Sapur.002G032100 Sapur.009G018000
GO:0047134	7	11	protein-disulfide reductase (NAD(P)) activity	Sapur.004G112400 Sapur.009G086900 Sapur.009G120300 Sapur.010G050700 Sapur.010G050800 Sapur.010G050900 Sapur.016G123800
GO:0016668	7	11	oxidoreductase activity, acting on a sulfur group of donors, NAD(P) as acceptor	Sapur.004G112400 Sapur.009G086900 Sapur.009G120300 Sapur.010G050700 Sapur.010G050800 Sapur.010G050900 Sapur.016G123800
GO:0017111	65	266	ribonucleoside triphosphate phosphatase activity	Sapur.001G078400 Sapur.001G078600 Sapur.001G122200 Sapur.002G109500 Sapur.002G132500 Sapur.002G156100 Sapur.002G169300 Sapur.002G188700 Sapur.003G014300 Sapur.003G018100 Sapur.003G025000 Sapur.003G099500 Sapur.003G125800 Sapur.003G138500 Sapur.004G027800 Sapur.004G033200 Sapur.004G111400 Sapur.005G048900 Sapur.006G094300 Sapur.006G163000 Sapur.007G075800 Sapur.008G077900 Sapur.008G091700 Sapur.009G004200 Sapur.009G029500 Sapur.009G086200 Sapur.009G128500 Sapur.010G001900 Sapur.010G024600 Sapur.010G055900 Sapur.010G087600 Sapur.010G102500 Sapur.010G119700 Sapur.010G119800 Sapur.010G137200 Sapur.011G028400 Sapur.012G050600 Sapur.012G084200 Sapur.013G110800 Sapur.014G032900 Sapur.014G061800 Sapur.014G090200 Sapur.014G090300 Sapur.014G113100 Sapur.016G141300 Sapur.016G195300 Sapur.016G230700 Sapur.016G230800 Sapur.017G029100 Sapur.017G055300 Sapur.017G057900 Sapur.017G067300 Sapur.017G074500 Sapur.017G129000 Sapur.018G063900 Sapur.019G048800 Sapur.019G080700 Sapur.019G113800 Sapur.15ZG018400 Sapur.15ZG103200 Sapur.15ZG109900 Sapur.15ZG126400 Sapur.T005400 Sapur.T005500 Sapur.T080500
GO:0003883	5	6	CTP synthase activity	Sapur.001G057600 Sapur.003G116600 Sapur.008G063700 Sapur.010G137300 Sapur.014G098600
GO:0016462	67	278	pyrophosphatase activity	Sapur.001G078400 Sapur.001G078600 Sapur.001G122200 Sapur.002G109500 Sapur.002G132500 Sapur.002G156100 Sapur.002G169300 Sapur.002G188700 Sapur.003G014300 Sapur.003G018100 Sapur.003G025000 Sapur.003G099500 Sapur.003G125800 Sapur.003G138500 Sapur.004G027800 Sapur.004G033200 Sapur.004G111400 Sapur.005G048900 Sapur.006G094300 Sapur.006G163000 Sapur.007G075800 Sapur.008G077900 Sapur.008G091700 Sapur.009G004200 Sapur.009G029500 Sapur.009G086200 Sapur.009G128500 Sapur.010G001900 Sapur.010G024600 Sapur.010G055900 Sapur.010G087600 Sapur.010G102500 Sapur.010G119700 Sapur.010G119800 Sapur.010G137200 Sapur.011G028400 Sapur.012G050600 Sapur.012G084200 Sapur.013G110800 Sapur.014G032900 Sapur.014G061800 Sapur.014G090200 Sapur.014G090300 Sapur.014G113100 Sapur.016G141300 Sapur.016G195300 Sapur.016G230700 Sapur.016G230800 Sapur.017G029100 Sapur.017G055300 Sapur.017G057900 Sapur.017G067300 Sapur.017G074500 Sapur.017G129000 Sapur.018G063900 Sapur.019G048800 Sapur.019G080700 Sapur.019G113800 Sapur.15ZG018400

				Sapur.15ZG103200 Sapur.15ZG109900 Sapur.15ZG126400 Sapur.T005400 Sapur.T005500 Sapur.T080500 Sapur.005G013000 Sapur.007G020200
GO:0016705	82	354	oxidoreductase activity, acting on paired donors, with incorporation or reduction of molecular oxygen	Sapur.008G055000 Sapur.010G147100 Sapur.016G102700 Sapur.016G142600 Sapur.018G103000 Sapur.001G067900 Sapur.001G148900 Sapur.001G149000 Sapur.001G149100 Sapur.001G167600 Sapur.001G193000 Sapur.002G019400 Sapur.002G106000 Sapur.002G107000 Sapur.002G141800 Sapur.003G003700 Sapur.003G037400 Sapur.003G037500 Sapur.003G082800 Sapur.003G093200 Sapur.003G109000 Sapur.003G130700 Sapur.004G088800 Sapur.004G096100 Sapur.004G139700 Sapur.004G148900 Sapur.006G045100 Sapur.006G045200 Sapur.006G098500 Sapur.006G116900 Sapur.006G121800 Sapur.006G186800 Sapur.007G078000 Sapur.007G080700 Sapur.007G080800 Sapur.007G103200 Sapur.008G166400 Sapur.009G030600 Sapur.009G053600 Sapur.009G085400 Sapur.009G085600 Sapur.009G085700 Sapur.009G086400 Sapur.010G021800 Sapur.010G022000 Sapur.010G088800 Sapur.010G088900 Sapur.010G109100 Sapur.010G109200 Sapur.010G149000 Sapur.011G066700 Sapur.011G097000 Sapur.012G043000 Sapur.012G066500 Sapur.013G080500 Sapur.013G084400 Sapur.014G011100 Sapur.014G011200 Sapur.014G011900 Sapur.014G027000 Sapur.014G027100 Sapur.014G027200 Sapur.014G027400 Sapur.014G027800 Sapur.014G028400 Sapur.014G028500 Sapur.014G029000 Sapur.014G080900 Sapur.014G126900 Sapur.014G127800 Sapur.016G007300 Sapur.016G007700 Sapur.016G007900 Sapur.016G028200 Sapur.016G205100 Sapur.016G209200 Sapur.016G285400 Sapur.016G285500 Sapur.019G065400 Sapur.T047000 Sapur.T073900 Sapur.T131000
GO:0022857	119	556	transmembrane transporter activity	Sapur.001G006200 Sapur.003G069300 Sapur.010G194900 Sapur.012G100800 Sapur.013G059700 Sapur.001G026600 Sapur.001G078400 Sapur.001G094500 Sapur.001G139700 Sapur.002G004100 Sapur.002G043400 Sapur.002G067900 Sapur.002G086100 Sapur.002G132500 Sapur.002G156100 Sapur.003G025000 Sapur.003G081500 Sapur.003G099500 Sapur.003G138500 Sapur.003G148600 Sapur.004G008500 Sapur.004G009600 Sapur.004G009700 Sapur.004G019600 Sapur.004G138100 Sapur.004G157800 Sapur.004G173400 Sapur.005G009200 Sapur.005G061600 Sapur.005G082300 Sapur.005G114300 Sapur.005G131700 Sapur.005G137600 Sapur.005G137700 Sapur.005G163600 Sapur.005G163800 Sapur.005G177000 Sapur.005G204900 Sapur.006G010900 Sapur.006G023600 Sapur.007G081600 Sapur.008G101500 Sapur.008G101600 Sapur.008G101700 Sapur.008G134200 Sapur.009G034100 Sapur.009G040500 Sapur.009G097300 Sapur.010G001900 Sapur.010G024300 Sapur.010G024600 Sapur.010G089300 Sapur.010G164900 Sapur.011G001000 Sapur.011G014000 Sapur.011G028400 Sapur.011G080900 Sapur.011G110300 Sapur.012G102500 Sapur.012G111400 Sapur.013G071900 Sapur.013G072500 Sapur.013G072600 Sapur.013G072700 Sapur.013G108500 Sapur.014G085900 Sapur.014G090200 Sapur.014G090300 Sapur.016G028400 Sapur.016G106100 Sapur.016G153700 Sapur.016G194200 Sapur.016G230700 Sapur.016G230800 Sapur.016G292400 Sapur.017G057900 Sapur.018G063900 Sapur.019G046000 Sapur.019G064700 Sapur.019G083100 Sapur.019G113800 Sapur.15ZG018400 Sapur.15ZG074200 Sapur.15ZG126400 Sapur.T005400 Sapur.T005500 Sapur.001G137400 Sapur.002G177500 Sapur.002G177600 Sapur.003G031900 Sapur.006G076500 Sapur.006G206900 Sapur.008G065700 Sapur.009G028800 Sapur.009G028900 Sapur.009G057800 Sapur.011G043000 Sapur.011G043200 Sapur.012G017000 Sapur.012G025300 Sapur.012G028700

GO:0005509	37	139	calcium ion binding	Sapur.012G055400 Sapur.014G124400 Sapur.016G225300 Sapur.016G239700 Sapur.016G266000 Sapur.018G009000 Sapur.018G022500 Sapur.018G026000 Sapur.15ZG026500 Sapur.001G008300 Sapur.001G054900 Sapur.005G122600 Sapur.008G066200 Sapur.008G163400 Sapur.009G057300 Sapur.010G133500 Sapur.017G059600 Sapur.018G103300 Sapur.002G009700 Sapur.002G044200 Sapur.002G061100 Sapur.002G103300 Sapur.004G019000 Sapur.004G152000 Sapur.004G175300 Sapur.005G007700 Sapur.005G020600 Sapur.005G103700 Sapur.005G205500 Sapur.006G035300 Sapur.007G031300 Sapur.008G032900 Sapur.008G082500 Sapur.009G048400 Sapur.009G128000 Sapur.010G079400 Sapur.010G165900 Sapur.011G018600 Sapur.011G027800 Sapur.012G013200 Sapur.013G051600 Sapur.014G019000 Sapur.014G019100 Sapur.014G019200 Sapur.014G020000 Sapur.014G020100 Sapur.014G020300 Sapur.014G020400 Sapur.016G038600 Sapur.016G040200 Sapur.018G092500 Sapur.T032000 Sapur.T079400 Sapur.T079500 Sapur.T080000
GO:0016874	25	84	ligase activity	Sapur.003G092400 Sapur.001G057600 Sapur.001G085300 Sapur.001G183500 Sapur.002G161200 Sapur.003G116600 Sapur.004G008500 Sapur.005G058900 Sapur.007G002900 Sapur.007G115600 Sapur.008G063700 Sapur.009G002900 Sapur.009G056800 Sapur.009G112500 Sapur.010G093900 Sapur.010G121200 Sapur.010G137300 Sapur.010G193600 Sapur.011G008400 Sapur.011G014000 Sapur.013G108500 Sapur.014G098600 Sapur.017G108800 Sapur.001G084200 Sapur.010G047000
GO:0016817	68	293	hydrolase activity, acting on acid anhydrides	Sapur.001G078400 Sapur.001G078600 Sapur.001G122200 Sapur.002G109500 Sapur.002G132500 Sapur.002G156100 Sapur.002G169300 Sapur.002G188700 Sapur.003G014300 Sapur.003G018100 Sapur.003G025000 Sapur.003G099500 Sapur.003G125800 Sapur.003G138500 Sapur.004G027800 Sapur.004G033200 Sapur.004G111400 Sapur.005G048900 Sapur.006G094300 Sapur.006G163000 Sapur.007G075800 Sapur.008G077900 Sapur.008G091700 Sapur.009G004200 Sapur.009G029500 Sapur.009G086200 Sapur.009G128500 Sapur.010G001900 Sapur.010G024600 Sapur.010G055900 Sapur.010G087600 Sapur.010G102500 Sapur.010G119700 Sapur.010G119800 Sapur.010G137200 Sapur.011G028400 Sapur.012G050600 Sapur.012G084200 Sapur.013G110800 Sapur.014G032900 Sapur.014G061800 Sapur.014G090200 Sapur.014G090300 Sapur.014G113100 Sapur.016G141300 Sapur.016G195300 Sapur.016G230700 Sapur.016G230800 Sapur.017G029100 Sapur.017G055300 Sapur.017G057900 Sapur.017G067300 Sapur.017G074500 Sapur.017G129000 Sapur.018G063900 Sapur.019G048800 Sapur.019G080700 Sapur.019G113800 Sapur.15ZG018400 Sapur.15ZG103200 Sapur.15ZG109900 Sapur.15ZG126400 Sapur.T005400 Sapur.T005500 Sapur.T080500 Sapur.005G013000 Sapur.007G020200 Sapur.001G151600
GO:0016818	68	293	hydrolase activity, acting on acid anhydrides, in phosphorus-containing anhydrides	Sapur.001G078400 Sapur.001G078600 Sapur.001G122200 Sapur.002G109500 Sapur.002G132500 Sapur.002G156100 Sapur.002G169300 Sapur.002G188700 Sapur.003G014300 Sapur.003G018100 Sapur.003G025000 Sapur.003G099500 Sapur.003G125800 Sapur.003G138500 Sapur.004G027800 Sapur.004G033200 Sapur.004G111400 Sapur.005G048900 Sapur.006G094300 Sapur.006G163000 Sapur.007G075800 Sapur.008G077900 Sapur.008G091700 Sapur.009G004200 Sapur.009G029500 Sapur.009G086200 Sapur.009G128500 Sapur.010G001900 Sapur.010G024600 Sapur.010G055900 Sapur.010G087600 Sapur.010G102500 Sapur.010G119700 Sapur.010G119800 Sapur.010G137200

				Sapur.011G028400 Sapur.012G050600 Sapur.012G084200 Sapur.013G110800 Sapur.014G032900 Sapur.014G061800 Sapur.014G090200 Sapur.014G090300 Sapur.014G113100 Sapur.016G141300 Sapur.016G195300 Sapur.016G230700 Sapur.016G230800 Sapur.017G029100 Sapur.017G055300 Sapur.017G057900 Sapur.017G067300 Sapur.017G074500 Sapur.017G129000 Sapur.018G063900 Sapur.019G048800 Sapur.019G080700 Sapur.019G113800 Sapur.15ZG018400 Sapur.15ZG103200 Sapur.15ZG109900 Sapur.15ZG126400 Sapur.T005400 Sapur.T005500 Sapur.T080500 Sapur.005G013000 Sapur.007G020200 Sapur.001G151600
GO:0042626	23	80	ATPase-coupled transmembrane transporter activity	Sapur.001G078400 Sapur.002G132500 Sapur.002G156100 Sapur.003G025000 Sapur.003G099500 Sapur.003G138500 Sapur.004G008500 Sapur.004G173400 Sapur.010G001900 Sapur.010G024600 Sapur.011G014000 Sapur.011G028400 Sapur.014G090200 Sapur.014G090300 Sapur.016G230700 Sapur.016G230800 Sapur.017G057900 Sapur.018G063900 Sapur.019G113800 Sapur.15ZG018400 Sapur.15ZG126400 Sapur.T005400 Sapur.T005500
GO:0005488	1554	9008	binding	Sapur.001G013800 Sapur.001G020500 Sapur.001G025400 Sapur.001G029700 Sapur.001G047800 Sapur.001G055600 Sapur.001G058800 Sapur.001G067700 Sapur.001G067900 Sapur.001G069800 Sapur.001G072500 Sapur.001G081300 Sapur.001G090900 Sapur.001G119600 Sapur.001G129000 Sapur.001G130300 Sapur.001G136400 Sapur.001G137000 Sapur.001G148900 Sapur.001G149000 Sapur.001G149100 Sapur.001G151600 Sapur.001G166100 Sapur.001G167600 Sapur.001G167800 Sapur.001G178100 Sapur.001G179000 Sapur.001G193000 Sapur.002G003300 Sapur.002G009700 Sapur.002G019400 Sapur.002G021500 Sapur.002G023100 Sapur.002G028500 Sapur.002G031600 Sapur.002G035300 Sapur.002G036100 Sapur.002G043300 Sapur.002G044200 Sapur.002G048900 Sapur.002G061100 Sapur.002G064600 Sapur.002G069500 Sapur.002G076800 Sapur.002G080700 Sapur.002G091700 Sapur.002G103300 Sapur.002G106000 Sapur.002G107000 Sapur.002G110600 Sapur.002G116100 Sapur.002G125900 Sapur.002G126000 Sapur.002G136900 Sapur.002G141800 Sapur.002G142300 Sapur.002G145300 Sapur.002G150200 Sapur.002G160400 Sapur.003G003700 Sapur.003G016500 Sapur.003G016800 Sapur.003G025800 Sapur.003G037400 Sapur.003G037500 Sapur.003G047900 Sapur.003G057500 Sapur.003G060800 Sapur.003G068400 Sapur.003G082800 Sapur.003G090400 Sapur.003G093200 Sapur.003G102800 Sapur.003G109000 Sapur.003G123800 Sapur.003G129600 Sapur.003G130700 Sapur.003G142300 Sapur.003G150500 Sapur.003G152800 Sapur.003G155100 Sapur.003G156600 Sapur.003G159400 Sapur.003G168000 Sapur.004G019000 Sapur.004G030500 Sapur.004G030800 Sapur.004G030900 Sapur.004G034900 Sapur.004G051000 Sapur.004G054800 Sapur.004G056900 Sapur.004G062200 Sapur.004G079500 Sapur.004G088800 Sapur.004G089500 Sapur.004G096100 Sapur.004G101700 Sapur.004G103000 Sapur.004G110300 Sapur.004G121800 Sapur.004G137000 Sapur.004G139700 Sapur.004G144500 Sapur.004G152000 Sapur.004G162100 Sapur.004G165200 Sapur.004G175300 Sapur.005G000100 Sapur.005G005600 Sapur.005G007700 Sapur.005G019400 Sapur.005G019900 Sapur.005G020600 Sapur.005G026800 Sapur.005G026900 Sapur.005G028900 Sapur.005G032200 Sapur.005G040700 Sapur.005G047900 Sapur.005G052900 Sapur.005G054500 Sapur.005G054800 Sapur.005G055700 Sapur.005G085100 Sapur.005G089700 Sapur.005G093100 Sapur.005G095300 Sapur.005G103700 Sapur.005G105500 Sapur.005G115200 Sapur.005G119200 Sapur.005G119500 Sapur.005G141300 Sapur.005G158900 Sapur.005G163900 Sapur.005G180000

Sapur.005G186700 Sapur.005G205500 Sapur.005G205700 Sapur.006G004800 Sapur.006G014400 Sapur.006G018700
Sapur.006G021100 Sapur.006G022000 Sapur.006G026800 Sapur.006G035300 Sapur.006G041400 Sapur.006G043000
Sapur.006G045100 Sapur.006G045200 Sapur.006G063200 Sapur.006G065100 Sapur.006G068600 Sapur.006G069400
Sapur.006G072700 Sapur.006G098500 Sapur.006G103400 Sapur.006G104700 Sapur.006G114200 Sapur.006G116900
Sapur.006G118700 Sapur.006G121100 Sapur.006G123200 Sapur.006G134000 Sapur.006G136200 Sapur.006G154200
Sapur.006G165100 Sapur.006G166000 Sapur.006G170000 Sapur.006G174900 Sapur.006G181200 Sapur.006G186800
Sapur.006G194000 Sapur.006G194100 Sapur.006G214400 Sapur.006G224700 Sapur.007G031300 Sapur.007G034000
Sapur.007G035800 Sapur.007G049700 Sapur.007G058700 Sapur.007G078000 Sapur.007G080400 Sapur.007G080700
Sapur.007G080800 Sapur.007G087800 Sapur.007G089800 Sapur.007G091900 Sapur.007G094000 Sapur.007G103200
Sapur.007G108700 Sapur.007G115600 Sapur.008G004200 Sapur.008G009100 Sapur.008G021500 Sapur.008G032900
Sapur.008G042700 Sapur.008G045800 Sapur.008G045900 Sapur.008G053100 Sapur.008G054800 Sapur.008G060600
Sapur.008G064800 Sapur.008G066900 Sapur.008G069200 Sapur.008G080600 Sapur.008G082500 Sapur.008G090400
Sapur.008G093800 Sapur.008G094300 Sapur.008G104300 Sapur.008G116400 Sapur.008G118700 Sapur.008G119700
Sapur.008G131800 Sapur.008G135800 Sapur.008G140000 Sapur.008G143900 Sapur.008G156200 Sapur.008G162200
Sapur.008G166400 Sapur.009G002900 Sapur.009G005300 Sapur.009G009700 Sapur.009G011600 Sapur.009G021700
Sapur.009G022800 Sapur.009G030600 Sapur.009G039700 Sapur.009G045200 Sapur.009G048400 Sapur.009G053600
Sapur.009G079400 Sapur.009G080900 Sapur.009G082700 Sapur.009G085400 Sapur.009G085600 Sapur.009G085700
Sapur.009G086400 Sapur.009G087200 Sapur.009G089000 Sapur.009G097200 Sapur.009G104900 Sapur.009G107600
Sapur.009G112500 Sapur.009G112800 Sapur.009G114600 Sapur.009G120800 Sapur.009G126000 Sapur.009G128000
Sapur.010G004000 Sapur.010G015700 Sapur.010G021800 Sapur.010G022000 Sapur.010G035500 Sapur.010G035800
Sapur.010G035900 Sapur.010G043500 Sapur.010G044400 Sapur.010G052700 Sapur.010G053300 Sapur.010G053800
Sapur.010G075900 Sapur.010G078000 Sapur.010G079400 Sapur.010G086800 Sapur.010G088800 Sapur.010G088900
Sapur.010G089800 Sapur.010G099700 Sapur.010G100200 Sapur.010G109100 Sapur.010G109200 Sapur.010G114800
Sapur.010G116400 Sapur.010G118700 Sapur.010G120000 Sapur.010G120300 Sapur.010G128300 Sapur.010G141100
Sapur.010G142500 Sapur.010G147200 Sapur.010G149000 Sapur.010G149100 Sapur.010G154300 Sapur.010G157800
Sapur.010G161100 Sapur.010G165900 Sapur.010G185200 Sapur.010G186700 Sapur.010G187200 Sapur.010G193700
Sapur.010G201700 Sapur.010G206100 Sapur.011G018600 Sapur.011G027800 Sapur.011G028600 Sapur.011G032100
Sapur.011G037100 Sapur.011G037900 Sapur.011G038200 Sapur.011G041100 Sapur.011G045100 Sapur.011G045800
Sapur.011G056100 Sapur.011G066700 Sapur.011G082900 Sapur.011G088400 Sapur.011G088500 Sapur.011G097000
Sapur.011G101200 Sapur.011G109600 Sapur.012G003700 Sapur.012G004000 Sapur.012G010400 Sapur.012G013200
Sapur.012G017700 Sapur.012G018600 Sapur.012G021100 Sapur.012G024500 Sapur.012G037700 Sapur.012G043000
Sapur.012G051400 Sapur.012G060100 Sapur.012G066500 Sapur.012G073300 Sapur.012G078700 Sapur.012G088900
Sapur.012G100600 Sapur.012G105100 Sapur.013G000100 Sapur.013G008000 Sapur.013G009700 Sapur.013G014300

Sapur.013G018100 Sapur.013G028900 Sapur.013G050700 Sapur.013G050900 Sapur.013G051000 Sapur.013G051600
Sapur.013G056200 Sapur.013G056700 Sapur.013G067900 Sapur.013G068800 Sapur.013G080500 Sapur.013G082200
Sapur.013G084100 Sapur.013G084400 Sapur.013G103900 Sapur.013G117300 Sapur.013G138200 Sapur.013G140300
Sapur.014G011100 Sapur.014G011200 Sapur.014G011900 Sapur.014G019000 Sapur.014G019100 Sapur.014G019200
Sapur.014G020000 Sapur.014G020100 Sapur.014G020300 Sapur.014G020400 Sapur.014G024600 Sapur.014G026600
Sapur.014G027000 Sapur.014G027100 Sapur.014G027200 Sapur.014G027400 Sapur.014G027800 Sapur.014G028400
Sapur.014G028500 Sapur.014G029000 Sapur.014G034500 Sapur.014G059000 Sapur.014G059100 Sapur.014G059200
Sapur.014G075400 Sapur.014G076200 Sapur.014G078500 Sapur.014G079300 Sapur.014G080900 Sapur.014G083400
Sapur.014G085400 Sapur.014G085500 Sapur.014G104200 Sapur.014G113600 Sapur.014G117700 Sapur.014G120800
Sapur.014G126900 Sapur.014G135200 Sapur.014G142400 Sapur.016G007300 Sapur.016G007700 Sapur.016G007900
Sapur.016G018700 Sapur.016G023300 Sapur.016G026400 Sapur.016G026800 Sapur.016G028200 Sapur.016G030200
Sapur.016G038600 Sapur.016G040200 Sapur.016G043500 Sapur.016G050900 Sapur.016G055400 Sapur.016G058000
Sapur.016G058600 Sapur.016G064600 Sapur.016G070400 Sapur.016G072900 Sapur.016G074300 Sapur.016G085100
Sapur.016G087600 Sapur.016G114700 Sapur.016G117100 Sapur.016G131300 Sapur.016G132500 Sapur.016G144700
Sapur.016G145900 Sapur.016G161800 Sapur.016G167800 Sapur.016G183700 Sapur.016G194900 Sapur.016G199700
Sapur.016G205100 Sapur.016G209200 Sapur.016G211300 Sapur.016G216900 Sapur.016G223300 Sapur.016G229800
Sapur.016G233500 Sapur.016G241300 Sapur.016G264800 Sapur.016G285400 Sapur.016G285500 Sapur.016G291800
Sapur.016G310400 Sapur.016G311100 Sapur.017G028400 Sapur.017G028500 Sapur.017G053300 Sapur.017G058200
Sapur.017G069900 Sapur.017G085900 Sapur.017G096600 Sapur.017G114800 Sapur.017G115700 Sapur.017G119200
Sapur.017G120700 Sapur.017G121300 Sapur.017G121600 Sapur.017G121700 Sapur.017G122100 Sapur.017G123100
Sapur.018G005400 Sapur.018G015800 Sapur.018G019900 Sapur.018G030000 Sapur.018G050900 Sapur.018G060900
Sapur.018G064600 Sapur.018G081500 Sapur.018G087600 Sapur.018G092500 Sapur.018G106800 Sapur.018G107600
Sapur.018G114600 Sapur.018G115000 Sapur.019G033200 Sapur.019G039400 Sapur.019G039900 Sapur.019G060100
Sapur.019G065400 Sapur.019G074500 Sapur.019G095300 Sapur.019G099800 Sapur.019G101700 Sapur.15WG036700
Sapur.15WG065100 Sapur.15WG067500 Sapur.15WG072800 Sapur.15ZG002000 Sapur.15ZG002400 Sapur.15ZG002500
Sapur.15ZG002800 Sapur.15ZG007300 Sapur.15ZG007500 Sapur.15ZG016900 Sapur.15ZG038900 Sapur.15ZG043500
Sapur.15ZG049200 Sapur.15ZG053200 Sapur.15ZG067200 Sapur.15ZG078500 Sapur.15ZG079000 Sapur.15ZG080900
Sapur.15ZG103400 Sapur.15ZG112800 Sapur.15ZG127500 Sapur.T012000 Sapur.T032000 Sapur.T045600
Sapur.T047000 Sapur.T058600 Sapur.T058800 Sapur.T073900 Sapur.T079400 Sapur.T079500 Sapur.T080000
Sapur.T131000 Sapur.T158900 Sapur.001G004400 Sapur.001G004500 Sapur.001G007900 Sapur.001G017300
Sapur.001G017800 Sapur.001G023800 Sapur.001G027900 Sapur.001G028100 Sapur.001G028400 Sapur.001G029800
Sapur.001G034200 Sapur.001G048800 Sapur.001G058300 Sapur.001G068000 Sapur.001G072400 Sapur.001G078400
Sapur.001G078600 Sapur.001G084200 Sapur.001G094200 Sapur.001G103000 Sapur.001G108900 Sapur.001G110300

Sapur.001G122200 Sapur.001G135100 Sapur.001G138100 Sapur.001G167000 Sapur.001G174900 Sapur.001G177000
Sapur.001G179800 Sapur.001G180200 Sapur.001G181600 Sapur.001G189000 Sapur.001G191700 Sapur.002G004200
Sapur.002G005300 Sapur.002G012000 Sapur.002G012900 Sapur.002G013600 Sapur.002G014300 Sapur.002G020900
Sapur.002G023300 Sapur.002G024200 Sapur.002G024400 Sapur.002G032100 Sapur.002G033200 Sapur.002G051400
Sapur.002G056700 Sapur.002G061600 Sapur.002G061700 Sapur.002G064800 Sapur.002G066500 Sapur.002G068500
Sapur.002G071000 Sapur.002G073000 Sapur.002G085200 Sapur.002G085900 Sapur.002G089300 Sapur.002G090300
Sapur.002G093900 Sapur.002G109500 Sapur.002G115000 Sapur.002G123400 Sapur.002G128300 Sapur.002G130500
Sapur.002G132300 Sapur.002G132500 Sapur.002G146000 Sapur.002G156100 Sapur.002G161200 Sapur.002G163600
Sapur.002G169300 Sapur.002G175800 Sapur.002G176300 Sapur.002G191900 Sapur.002G194100 Sapur.002G198400
Sapur.003G002000 Sapur.003G002100 Sapur.003G014300 Sapur.003G018100 Sapur.003G025000 Sapur.003G031900
Sapur.003G033600 Sapur.003G048700 Sapur.003G056300 Sapur.003G060500 Sapur.003G066000 Sapur.003G067100
Sapur.003G078600 Sapur.003G091600 Sapur.003G092400 Sapur.003G099500 Sapur.003G102000 Sapur.003G114500
Sapur.003G116100 Sapur.003G117300 Sapur.003G124900 Sapur.003G125800 Sapur.003G125900 Sapur.003G138200
Sapur.003G138500 Sapur.003G139700 Sapur.003G141900 Sapur.003G145700 Sapur.003G150800 Sapur.003G155400
Sapur.003G160700 Sapur.003G166200 Sapur.004G007000 Sapur.004G014400 Sapur.004G018300 Sapur.004G019100
Sapur.004G023100 Sapur.004G027800 Sapur.004G033200 Sapur.004G037400 Sapur.004G041000 Sapur.004G051400
Sapur.004G063300 Sapur.004G077500 Sapur.004G077700 Sapur.004G108600 Sapur.004G109700 Sapur.004G111400
Sapur.004G118800 Sapur.004G121500 Sapur.004G123100 Sapur.004G128600 Sapur.004G131500 Sapur.004G131600
Sapur.004G132200 Sapur.004G137100 Sapur.004G137600 Sapur.004G140300 Sapur.004G146400 Sapur.004G147000
Sapur.004G147900 Sapur.004G169900 Sapur.004G171400 Sapur.004G172100 Sapur.005G005000 Sapur.005G005700
Sapur.005G007100 Sapur.005G011200 Sapur.005G022300 Sapur.005G023100 Sapur.005G024800 Sapur.005G025000
Sapur.005G025400 Sapur.005G031700 Sapur.005G038400 Sapur.005G043600 Sapur.005G043700 Sapur.005G046200
Sapur.005G048900 Sapur.005G050200 Sapur.005G055500 Sapur.005G067000 Sapur.005G069000 Sapur.005G075800
Sapur.005G087400 Sapur.005G090600 Sapur.005G095700 Sapur.005G096300 Sapur.005G096400 Sapur.005G098100
Sapur.005G109500 Sapur.005G113600 Sapur.005G114400 Sapur.005G124600 Sapur.005G133200 Sapur.005G141400
Sapur.005G145700 Sapur.005G150000 Sapur.005G165600 Sapur.005G166000 Sapur.005G187300 Sapur.005G193700
Sapur.005G199500 Sapur.005G204200 Sapur.005G206400 Sapur.006G010200 Sapur.006G021400 Sapur.006G025100
Sapur.006G033400 Sapur.006G036800 Sapur.006G037200 Sapur.006G067400 Sapur.006G067500 Sapur.006G069500
Sapur.006G084800 Sapur.006G089400 Sapur.006G094200 Sapur.006G094300 Sapur.006G097400 Sapur.006G100100
Sapur.006G106100 Sapur.006G110100 Sapur.006G110200 Sapur.006G112500 Sapur.006G118100 Sapur.006G118200
Sapur.006G120300 Sapur.006G122700 Sapur.006G126200 Sapur.006G136800 Sapur.006G138300 Sapur.006G139300
Sapur.006G140600 Sapur.006G154300 Sapur.006G163000 Sapur.006G163500 Sapur.006G168000 Sapur.006G172300
Sapur.006G173100 Sapur.006G177100 Sapur.006G178000 Sapur.006G181100 Sapur.006G182100 Sapur.006G194600

Sapur.006G196800	Sapur.006G198000	Sapur.006G203200	Sapur.006G208000	Sapur.006G218000	Sapur.006G218800
Sapur.006G220900	Sapur.006G221300	Sapur.006G223700	Sapur.006G224200	Sapur.006G225500	Sapur.006G228700
Sapur.006G228900	Sapur.007G001300	Sapur.007G002900	Sapur.007G005100	Sapur.007G007000	Sapur.007G007100
Sapur.007G007600	Sapur.007G008900	Sapur.007G012800	Sapur.007G016800	Sapur.007G020200	Sapur.007G021900
Sapur.007G029800	Sapur.007G036500	Sapur.007G044500	Sapur.007G044600	Sapur.007G045800	Sapur.007G050900
Sapur.007G062200	Sapur.007G069600	Sapur.007G071200	Sapur.007G073200	Sapur.007G075800	Sapur.007G077000
Sapur.007G077600	Sapur.007G081900	Sapur.007G086000	Sapur.007G086100	Sapur.007G087100	Sapur.007G088300
Sapur.007G098200	Sapur.007G111300	Sapur.007G111400	Sapur.007G111700	Sapur.007G112200	Sapur.008G004700
Sapur.008G004900	Sapur.008G022900	Sapur.008G026800	Sapur.008G044200	Sapur.008G046000	Sapur.008G058400
Sapur.008G060200	Sapur.008G062600	Sapur.008G065300	Sapur.008G077900	Sapur.008G081500	Sapur.008G081900
Sapur.008G088300	Sapur.008G090300	Sapur.008G091700	Sapur.008G097000	Sapur.008G115400	Sapur.008G120600
Sapur.008G121600	Sapur.008G128300	Sapur.008G131900	Sapur.008G137200	Sapur.008G151300	Sapur.008G151900
Sapur.008G162000	Sapur.009G002700	Sapur.009G004200	Sapur.009G005200	Sapur.009G008500	Sapur.009G010000
Sapur.009G018000	Sapur.009G021900	Sapur.009G025800	Sapur.009G032000	Sapur.009G041000	Sapur.009G051100
Sapur.009G057000	Sapur.009G064100	Sapur.009G064400	Sapur.009G066300	Sapur.009G073500	Sapur.009G086200
Sapur.009G094100	Sapur.009G094200	Sapur.009G094800	Sapur.009G097900	Sapur.009G098700	Sapur.009G099800
Sapur.009G112900	Sapur.009G116000	Sapur.009G117400	Sapur.009G120100	Sapur.009G122200	Sapur.010G001900
Sapur.010G003100	Sapur.010G009400	Sapur.010G009500	Sapur.010G013000	Sapur.010G014000	Sapur.010G023400
Sapur.010G024600	Sapur.010G026800	Sapur.010G029400	Sapur.010G042700	Sapur.010G042900	Sapur.010G046700
Sapur.010G055900	Sapur.010G057900	Sapur.010G060700	Sapur.010G065900	Sapur.010G070700	Sapur.010G086100
Sapur.010G087600	Sapur.010G090000	Sapur.010G092300	Sapur.010G092700	Sapur.010G093900	Sapur.010G100000
Sapur.010G102500	Sapur.010G104500	Sapur.010G109700	Sapur.010G114700	Sapur.010G119000	Sapur.010G119700
Sapur.010G119800	Sapur.010G123400	Sapur.010G137200	Sapur.010G138200	Sapur.010G138400	Sapur.010G138500
Sapur.010G143600	Sapur.010G152700	Sapur.010G154500	Sapur.010G157400	Sapur.010G163200	Sapur.010G175000
Sapur.010G193600	Sapur.011G006600	Sapur.011G006700	Sapur.011G008400	Sapur.011G013300	Sapur.011G018500
Sapur.011G028400	Sapur.011G031900	Sapur.011G034200	Sapur.011G035500	Sapur.011G037200	Sapur.011G049300
Sapur.011G054600	Sapur.011G061100	Sapur.011G062300	Sapur.011G063500	Sapur.011G071900	Sapur.011G073000
Sapur.011G088900	Sapur.011G099500	Sapur.011G102200	Sapur.011G103500	Sapur.011G103700	Sapur.011G103800
Sapur.011G109700	Sapur.011G109800	Sapur.011G120500	Sapur.012G000700	Sapur.012G031100	Sapur.012G031400
Sapur.012G037400	Sapur.012G040700	Sapur.012G048900	Sapur.012G050300	Sapur.012G050600	Sapur.012G061900
Sapur.012G067300	Sapur.012G067900	Sapur.012G069200	Sapur.012G070700	Sapur.012G071400	Sapur.012G074000
Sapur.012G074300	Sapur.012G079600	Sapur.012G084200	Sapur.012G086700	Sapur.012G087900	Sapur.012G091400
Sapur.012G091800	Sapur.012G092600	Sapur.012G092900	Sapur.012G099300	Sapur.012G100300	Sapur.012G101100

	Sapur.012G103800 Sapur.012G103900 Sapur.012G104300 Sapur.013G004800 Sapur.013G008600 Sapur.013G010700
	Sapur.013G021200 Sapur.013G025600 Sapur.013G035500 Sapur.013G039500 Sapur.013G045400 Sapur.013G055500
	Sapur.013G055900 Sapur.013G080000 Sapur.013G087600 Sapur.013G097100 Sapur.013G100200 Sapur.013G110800
	Sapur.013G117900 Sapur.013G118200 Sapur.013G118400 Sapur.013G120100 Sapur.013G120600 Sapur.013G123000
	Sapur.013G123300 Sapur.013G123700 Sapur.013G124400 Sapur.013G134100 Sapur.013G134200 Sapur.013G134300
	Sapur.013G137400 Sapur.014G001300 Sapur.014G001700 Sapur.014G002600 Sapur.014G003400 Sapur.014G003500
	Sapur.014G003700 Sapur.014G005100 Sapur.014G005200 Sapur.014G025600 Sapur.014G032900 Sapur.014G036100
	Sapur.014G040600 Sapur.014G051500 Sapur.014G052300 Sapur.014G053000 Sapur.014G055200 Sapur.014G055500
	Sapur.014G061800 Sapur.014G070700 Sapur.014G073900 Sapur.014G080400 Sapur.014G084500 Sapur.014G088900
	Sapur.014G090200 Sapur.014G090300 Sapur.014G094500 Sapur.014G095700 Sapur.014G099500 Sapur.014G104700
	Sapur.014G106600 Sapur.014G107900 Sapur.014G110600 Sapur.014G113000 Sapur.014G113100 Sapur.014G117400
	Sapur.014G120400 Sapur.014G132000 Sapur.014G134700 Sapur.014G135900 Sapur.016G001600 Sapur.016G009300
	Sapur.016G012000 Sapur.016G018900 Sapur.016G031400 Sapur.016G035600 Sapur.016G040000 Sapur.016G051900
	Sapur.016G052700 Sapur.016G060400 Sapur.016G071200 Sapur.016G071600 Sapur.016G077600 Sapur.016G079900
	Sapur.016G080200 Sapur.016G104800 Sapur.016G107800 Sapur.016G111900 Sapur.016G120800 Sapur.016G123500
	Sapur.016G125300 Sapur.016G132300 Sapur.016G132400 Sapur.016G139500 Sapur.016G141300 Sapur.016G147900
	Sapur.016G150200 Sapur.016G152700 Sapur.016G159000 Sapur.016G163400 Sapur.016G184500 Sapur.016G187000
	Sapur.016G189500 Sapur.016G189900 Sapur.016G200900 Sapur.016G201800 Sapur.016G216200 Sapur.016G224700
	Sapur.016G228700 Sapur.016G230700 Sapur.016G230800 Sapur.016G243200 Sapur.016G243400 Sapur.016G246600
	Sapur.016G247200 Sapur.016G254800 Sapur.016G262400 Sapur.016G267000 Sapur.016G281900 Sapur.016G282700
	Sapur.016G288500 Sapur.016G293000 Sapur.016G298800 Sapur.016G299400 Sapur.016G302100 Sapur.016G302500
	Sapur.016G304100 Sapur.017G001400 Sapur.017G003500 Sapur.017G004800 Sapur.017G009300 Sapur.017G010500
	Sapur.017G018700 Sapur.017G020600 Sapur.017G023600 Sapur.017G023800 Sapur.017G029100 Sapur.017G029300
	Sapur.017G036400 Sapur.017G036800 Sapur.017G037700 Sapur.017G052400 Sapur.017G055300 Sapur.017G057100
	Sapur.017G057300 Sapur.017G057900 Sapur.017G074500 Sapur.017G100600 Sapur.017G101100 Sapur.017G101200
	Sapur.017G101400 Sapur.017G102500 Sapur.017G104800 Sapur.017G108100 Sapur.017G108200 Sapur.017G108700
	Sapur.017G110500 Sapur.017G110700 Sapur.017G111200 Sapur.017G112200 Sapur.017G112400 Sapur.017G113400
	Sapur.017G123300 Sapur.017G124700 Sapur.017G129000 Sapur.018G002400 Sapur.018G003500 Sapur.018G003800
	Sapur.018G004300 Sapur.018G007100 Sapur.018G008500 Sapur.018G011700 Sapur.018G015000 Sapur.018G016300
	Sapur.018G016500 Sapur.018G016600 Sapur.018G016800 Sapur.018G017000 Sapur.018G023200 Sapur.018G025000
	Sapur.018G025100 Sapur.018G025700 Sapur.018G035300 Sapur.018G039600 Sapur.018G045400 Sapur.018G047200
	Sapur.018G047900 Sapur.018G056400 Sapur.018G057100 Sapur.018G057600 Sapur.018G057700 Sapur.018G060400
	Sapur.018G063600 Sapur.018G063900 Sapur.018G067200 Sapur.018G072600 Sapur.018G074200 Sapur.018G074300

Sapur.018G077200 Sapur.018G088200 Sapur.018G089300 Sapur.018G100400 Sapur.018G105800 Sapur.018G114900
Sapur.018G115800 Sapur.018G115900 Sapur.018G117400 Sapur.019G001100 Sapur.019G003100 Sapur.019G005200
Sapur.019G005900 Sapur.019G007100 Sapur.019G007800 Sapur.019G007900 Sapur.019G009200 Sapur.019G009300
Sapur.019G009500 Sapur.019G009600 Sapur.019G010500 Sapur.019G010600 Sapur.019G010700 Sapur.019G011000
Sapur.019G011100 Sapur.019G011200 Sapur.019G011500 Sapur.019G011600 Sapur.019G011700 Sapur.019G011800
Sapur.019G011900 Sapur.019G012000 Sapur.019G012100 Sapur.019G012400 Sapur.019G012500 Sapur.019G012700
Sapur.019G012800 Sapur.019G013700 Sapur.019G015500 Sapur.019G016400 Sapur.019G017000 Sapur.019G020700
Sapur.019G022800 Sapur.019G023100 Sapur.019G027100 Sapur.019G027300 Sapur.019G027700 Sapur.019G028700
Sapur.019G029100 Sapur.019G034300 Sapur.019G041400 Sapur.019G042100 Sapur.019G042400 Sapur.019G042500
Sapur.019G042900 Sapur.019G043100 Sapur.019G043200 Sapur.019G044000 Sapur.019G044100 Sapur.019G046700
Sapur.019G048000 Sapur.019G048800 Sapur.019G053500 Sapur.019G058700 Sapur.019G061100 Sapur.019G065000
Sapur.019G070000 Sapur.019G075900 Sapur.019G080700 Sapur.019G082400 Sapur.019G084800 Sapur.019G084900
Sapur.019G091100 Sapur.019G091300 Sapur.019G091700 Sapur.019G092300 Sapur.019G092600 Sapur.019G092700
Sapur.019G092800 Sapur.019G093100 Sapur.019G093400 Sapur.019G093600 Sapur.019G093900 Sapur.019G094300
Sapur.019G106100 Sapur.019G106400 Sapur.019G107300 Sapur.019G108100 Sapur.019G110800 Sapur.019G111400
Sapur.019G113800 Sapur.15WG028000 Sapur.15WG030400 Sapur.15WG040400 Sapur.15WG043400 Sapur.15WG044400
Sapur.15WG063800 Sapur.15WG076100 Sapur.15WG081000 Sapur.15ZG018400 Sapur.15ZG044100 Sapur.15ZG044300
Sapur.15ZG044900 Sapur.15ZG049500 Sapur.15ZG050400 Sapur.15ZG050500 Sapur.15ZG059500 Sapur.15ZG068500
Sapur.15ZG073500 Sapur.15ZG078300 Sapur.15ZG085300 Sapur.15ZG095900 Sapur.15ZG099200 Sapur.15ZG103200
Sapur.15ZG109900 Sapur.15ZG116100 Sapur.15ZG120200 Sapur.15ZG123100 Sapur.15ZG123200 Sapur.15ZG126400
Sapur.T003900 Sapur.T005400 Sapur.T005500 Sapur.T028300 Sapur.T029400 Sapur.T030800 Sapur.T046000
Sapur.T051400 Sapur.T080500 Sapur.T096600 Sapur.T115500 Sapur.T116500 Sapur.T117000 Sapur.T131900
Sapur.T133700 Sapur.T147200 Sapur.T150000 Sapur.T175200 Sapur.T191500 Sapur.T192100 Sapur.001G005800
Sapur.001G014300 Sapur.001G040700 Sapur.001G052100 Sapur.001G056500 Sapur.001G073800 Sapur.001G084600
Sapur.001G107500 Sapur.001G117800 Sapur.001G119300 Sapur.001G144800 Sapur.001G173100 Sapur.001G190900
Sapur.002G021000 Sapur.002G028900 Sapur.002G042600 Sapur.002G043700 Sapur.002G045000 Sapur.002G055900
Sapur.002G092800 Sapur.002G095700 Sapur.002G137700 Sapur.002G137900 Sapur.002G142400 Sapur.002G154700
Sapur.002G154800 Sapur.002G168300 Sapur.002G172100 Sapur.002G196700 Sapur.002G202800 Sapur.003G007600
Sapur.003G033800 Sapur.003G057900 Sapur.003G068900 Sapur.003G071000 Sapur.003G088000 Sapur.003G110000
Sapur.003G123200 Sapur.003G158900 Sapur.003G163500 Sapur.004G010500 Sapur.004G021100 Sapur.004G023800
Sapur.004G063600 Sapur.004G067700 Sapur.004G099100 Sapur.004G099300 Sapur.004G111000 Sapur.004G134900
Sapur.004G153900 Sapur.004G154400 Sapur.004G173500 Sapur.005G002500 Sapur.005G002800 Sapur.005G004800
Sapur.005G020200 Sapur.005G044100 Sapur.005G044500 Sapur.005G083500 Sapur.005G083700 Sapur.005G124500

	Sapur.005G132500 Sapur.005G143100 Sapur.005G145800 Sapur.005G151600 Sapur.005G162300 Sapur.005G163400
	Sapur.005G164500 Sapur.005G165400 Sapur.005G173500 Sapur.005G187100 Sapur.005G188000 Sapur.005G188400
	Sapur.005G194300 Sapur.005G201300 Sapur.006G010800 Sapur.006G026600 Sapur.006G031800 Sapur.006G044800
	Sapur.006G046000 Sapur.006G048200 Sapur.006G060300 Sapur.006G060400 Sapur.006G079300 Sapur.006G080700
	Sapur.006G085200 Sapur.006G099400 Sapur.006G110400 Sapur.006G110500 Sapur.006G111900 Sapur.006G138100
	Sapur.006G145700 Sapur.006G157900 Sapur.006G161000 Sapur.006G170900 Sapur.006G179100 Sapur.006G186700
	Sapur.006G209900 Sapur.006G213200 Sapur.006G228400 Sapur.007G045900 Sapur.007G049900 Sapur.007G072000
	Sapur.007G072700 Sapur.007G082800 Sapur.007G085500 Sapur.007G100700 Sapur.007G120400 Sapur.008G002400
	Sapur.008G003800 Sapur.008G056000 Sapur.008G074900 Sapur.008G078500 Sapur.008G096200 Sapur.008G110000
	Sapur.008G115900 Sapur.008G135500 Sapur.008G135600 Sapur.008G137000 Sapur.008G142100 Sapur.008G142200
	Sapur.008G144900 Sapur.008G150100 Sapur.008G163800 Sapur.008G165600 Sapur.009G002600 Sapur.009G035200
	Sapur.009G050700 Sapur.009G056600 Sapur.009G070200 Sapur.009G111300 Sapur.009G115000 Sapur.009G122700
	Sapur.010G003400 Sapur.010G020800 Sapur.010G024000 Sapur.010G029900 Sapur.010G033000 Sapur.010G038000
	Sapur.010G045500 Sapur.010G051100 Sapur.010G091000 Sapur.010G113700 Sapur.010G131700 Sapur.010G143200
	Sapur.010G146200 Sapur.010G158700 Sapur.010G168100 Sapur.011G013700 Sapur.011G018300 Sapur.011G027600
	Sapur.011G041300 Sapur.011G060200 Sapur.011G065500 Sapur.011G084300 Sapur.011G101500 Sapur.011G104100
	Sapur.011G104200 Sapur.011G104400 Sapur.011G113400 Sapur.011G120600 Sapur.012G002400 Sapur.012G005800
	Sapur.012G005900 Sapur.012G013900 Sapur.012G021800 Sapur.012G028400 Sapur.012G045000 Sapur.012G046200
	Sapur.012G054100 Sapur.012G062700 Sapur.012G063300 Sapur.012G071800 Sapur.012G080900 Sapur.012G089300
	Sapur.012G107700 Sapur.012G111100 Sapur.013G000400 Sapur.013G001100 Sapur.013G015400 Sapur.013G018800
	Sapur.013G035400 Sapur.013G045600 Sapur.013G050200 Sapur.013G051900 Sapur.013G052500 Sapur.013G068400
	Sapur.013G068600 Sapur.013G086200 Sapur.013G093600 Sapur.013G096400 Sapur.013G098900 Sapur.013G101300
	Sapur.013G101500 Sapur.013G106400 Sapur.013G109200 Sapur.013G126600 Sapur.013G142000 Sapur.014G006200
	Sapur.014G012300 Sapur.014G015400 Sapur.014G029900 Sapur.014G048200 Sapur.014G063000 Sapur.014G078100
	Sapur.014G082400 Sapur.014G084100 Sapur.014G084300 Sapur.014G088300 Sapur.014G105800 Sapur.014G122600
	Sapur.014G133800 Sapur.014G137100 Sapur.016G004700 Sapur.016G008700 Sapur.016G009100 Sapur.016G009900
	Sapur.016G010000 Sapur.016G021300 Sapur.016G031300 Sapur.016G038500 Sapur.016G044100 Sapur.016G044700
	Sapur.016G058400 Sapur.016G060700 Sapur.016G070300 Sapur.016G099000 Sapur.016G118000 Sapur.016G142200
	Sapur.016G142300 Sapur.016G164700 Sapur.016G177700 Sapur.016G181300 Sapur.016G181500 Sapur.016G185900
	Sapur.016G186000 Sapur.016G186200 Sapur.016G211600 Sapur.016G213600 Sapur.016G218300 Sapur.016G291000
	Sapur.016G297200 Sapur.016G308700 Sapur.018G011800 Sapur.018G012000 Sapur.018G019300 Sapur.018G020200
	Sapur.018G022800 Sapur.018G048100 Sapur.018G083500 Sapur.018G104000 Sapur.018G108400 Sapur.018G108600
	Sapur.018G108800 Sapur.018G113900 Sapur.018G115600 Sapur.019G010100 Sapur.019G029700 Sapur.019G032900

				Sapur.019G047000 Sapur.019G066100 Sapur.019G082300 Sapur.019G088500 Sapur.019G092000 Sapur.019G093000 Sapur.019G106000 Sapur.019G113100 Sapur.15WG071100 Sapur.15WG079800 Sapur.15ZG000400 Sapur.15ZG002100 Sapur.15ZG023100 Sapur.15ZG037500 Sapur.15ZG076100 Sapur.15ZG078400 Sapur.15ZG078800 Sapur.15ZG103600 Sapur.15ZG112600 Sapur.T004300 Sapur.T051200 Sapur.T175100 Sapur.002G081000 Sapur.002G188700 Sapur.005G201100 Sapur.008G143800 Sapur.016G099500
GO:0008092	40	163	cytoskeletal protein binding	Sapur.001G094200 Sapur.002G064800 Sapur.002G085200 Sapur.002G089300 Sapur.004G019100 Sapur.004G109700 Sapur.004G147000 Sapur.005G022300 Sapur.005G124600 Sapur.006G036800 Sapur.006G067400 Sapur.006G069500 Sapur.006G112500 Sapur.006G172300 Sapur.007G007600 Sapur.007G045800 Sapur.007G081900 Sapur.008G137200 Sapur.009G122200 Sapur.010G042700 Sapur.011G018500 Sapur.011G103700 Sapur.012G037400 Sapur.012G040700 Sapur.012G050300 Sapur.012G099300 Sapur.014G005100 Sapur.014G051500 Sapur.014G052300 Sapur.016G071200 Sapur.018G057600 Sapur.15WG040400 Sapur.15WG044400 Sapur.15WG081000 Sapur.15ZG044100 Sapur.15ZG044300 Sapur.15ZG044900 Sapur.15ZG049500 Sapur.15ZG059500 Sapur.T003900
GO:0016879	13	38	ligase activity, forming carbon-nitrogen bonds	Sapur.003G092400 Sapur.001G057600 Sapur.001G085300 Sapur.001G183500 Sapur.003G116600 Sapur.005G058900 Sapur.008G063700 Sapur.009G056800 Sapur.010G121200 Sapur.010G137300 Sapur.010G193600 Sapur.014G098600 Sapur.017G108800
GO:0015036	27	101	disulfide oxidoreductase activity	Sapur.002G022500 Sapur.002G152900 Sapur.002G153000 Sapur.003G104600 Sapur.005G185900 Sapur.005G196700 Sapur.006G101300 Sapur.006G187000 Sapur.007G032000 Sapur.007G098300 Sapur.008G156500 Sapur.013G056600 Sapur.014G107200 Sapur.014G107700 Sapur.014G107800 Sapur.016G056100 Sapur.016G073300 Sapur.017G010000 Sapur.019G033700 Sapur.004G112400 Sapur.009G086900 Sapur.009G120300 Sapur.010G050700 Sapur.010G050800 Sapur.010G050900 Sapur.016G123800 Sapur.009G070700
GO:0005506	80	373	iron ion binding	Sapur.001G067900 Sapur.001G148900 Sapur.001G149000 Sapur.001G149100 Sapur.001G167600 Sapur.001G193000 Sapur.002G019400 Sapur.002G106000 Sapur.002G107000 Sapur.002G141800 Sapur.003G003700 Sapur.003G037400 Sapur.003G037500 Sapur.003G082800 Sapur.003G093200 Sapur.003G109000 Sapur.003G130700 Sapur.004G088800 Sapur.004G096100 Sapur.004G139700 Sapur.006G045100 Sapur.006G045200 Sapur.006G098500 Sapur.006G116900 Sapur.006G186800 Sapur.007G078000 Sapur.007G080700 Sapur.007G080800 Sapur.007G103200 Sapur.008G116400 Sapur.008G166400 Sapur.009G030600 Sapur.009G053600 Sapur.009G085400 Sapur.009G085600 Sapur.009G085700 Sapur.009G086400 Sapur.010G021800 Sapur.010G022000 Sapur.010G088800 Sapur.010G088900 Sapur.010G109100 Sapur.010G109200 Sapur.010G149000 Sapur.011G066700 Sapur.011G097000 Sapur.012G043000 Sapur.012G066500 Sapur.013G080500 Sapur.013G084400 Sapur.014G011100 Sapur.014G011200 Sapur.014G011900 Sapur.014G027000 Sapur.014G027100 Sapur.014G027200 Sapur.014G027400 Sapur.014G027800 Sapur.014G028400 Sapur.014G028500 Sapur.014G029000 Sapur.014G080900 Sapur.014G126900 Sapur.016G007300 Sapur.016G007700 Sapur.016G007900 Sapur.016G028200 Sapur.016G205100 Sapur.016G209200 Sapur.016G285400 Sapur.016G285500

				Sapur.017G123100 Sapur.018G050900 Sapur.019G065400 Sapur.T047000 Sapur.T073900 Sapur.T131000 Sapur.006G084800 Sapur.013G134100 Sapur.016G107800
GO:0015035	26	98	protein-disulfide reductase activity	Sapur.002G022500 Sapur.002G152900 Sapur.002G153000 Sapur.003G104600 Sapur.005G185900 Sapur.005G196700 Sapur.006G101300 Sapur.006G187000 Sapur.007G032000 Sapur.007G098300 Sapur.008G156500 Sapur.013G056600 Sapur.014G107200 Sapur.014G107700 Sapur.014G107800 Sapur.016G056100 Sapur.016G073300 Sapur.017G010000 Sapur.019G033700 Sapur.004G112400 Sapur.009G086900 Sapur.009G120300 Sapur.010G050700 Sapur.010G050800 Sapur.010G050900 Sapur.016G123800
GO:0051537	7	17	2 iron, 2 sulfur cluster binding	Sapur.001G004400 Sapur.001G004500 Sapur.002G066500 Sapur.003G166200 Sapur.006G220900 Sapur.012G100300 Sapur.018G011700
GO:0016597	10	29	amino acid binding	Sapur.001G048800 Sapur.003G124900 Sapur.005G145700 Sapur.008G004900 Sapur.008G062600 Sapur.014G003400 Sapur.016G201800 Sapur.016G228700 Sapur.019G070000 Sapur.15ZG099200
GO:0017150	3	4	tRNA dihydrouridine synthase activity	Sapur.007G044500 Sapur.009G099800 Sapur.016G152700
GO:0106413	3	4	RNA dihydrouridine synthase activity	Sapur.007G044500 Sapur.009G099800 Sapur.016G152700
GO:0016628	3	4	oxidoreductase activity, acting on the CH-CH group of donors, NAD or NADP as acceptor	Sapur.007G044500 Sapur.009G099800 Sapur.016G152700
GO:0016717	4	7	oxidoreductase activity, acting on paired donors, with oxidation of a pair of donors resulting in the reduction of molecular oxygen to two molecules of water	Sapur.008G055000 Sapur.010G147100 Sapur.016G102700 Sapur.016G142600
GO:001	4	7	carbon-nitrogen	Sapur.003G092400 Sapur.005G058900 Sapur.009G056800 Sapur.010G121200

6884			ligase activity, with glutamine as amido-N-donor	
GO:0019239	4	7	deaminase activity	Sapur.006G083400 Sapur.011G000700 Sapur.016G104000 Sapur.018G047900
GO:0008079	5	11	translation termination factor activity	Sapur.003G117300 Sapur.006G033400 Sapur.008G060200 Sapur.013G008600 Sapur.019G065000
GO:0003747	5	11	translation release factor activity	Sapur.003G117300 Sapur.006G033400 Sapur.008G060200 Sapur.013G008600 Sapur.019G065000
GO:0043177	10	31	organic acid binding	Sapur.001G048800 Sapur.003G124900 Sapur.005G145700 Sapur.008G004900 Sapur.008G062600 Sapur.014G003400 Sapur.016G201800 Sapur.016G228700 Sapur.019G070000 Sapur.15ZG099200
GO:0031406	10	31	carboxylic acid binding	Sapur.001G048800 Sapur.003G124900 Sapur.005G145700 Sapur.008G004900 Sapur.008G062600 Sapur.014G003400 Sapur.016G201800 Sapur.016G228700 Sapur.019G070000 Sapur.15ZG099200
GO:0016667	29	122	oxidoreductase activity, acting on a sulfur group of donors	Sapur.002G022500 Sapur.002G152900 Sapur.002G153000 Sapur.003G104600 Sapur.005G185900 Sapur.005G196700 Sapur.006G101300 Sapur.006G187000 Sapur.007G032000 Sapur.007G098300 Sapur.008G156500 Sapur.013G056600 Sapur.014G107200 Sapur.014G107700 Sapur.014G107800 Sapur.016G056100 Sapur.016G073300 Sapur.017G010000 Sapur.019G033700 Sapur.004G112400 Sapur.009G086900 Sapur.009G120300 Sapur.010G050700 Sapur.010G050800 Sapur.010G050900 Sapur.011G077900 Sapur.016G123800 Sapur.15ZG111600 Sapur.009G070700
GO:0016872	2	2	intramolecular lyase activity	Sapur.005G061300 Sapur.007G081700
GO:0016842	2	2	amidine-lyase activity	Sapur.002G085400 Sapur.010G151500
GO:0004712	2	2	protein serine/threonine/tyrosine kinase activity	Sapur.002G070300 Sapur.005G135200
GO:0004512	2	2	inositol-3-phosphate synthase activity	Sapur.005G061300 Sapur.007G081700
GO:0046906	91	454	tetrapyrrole binding	Sapur.001G067900 Sapur.001G148900 Sapur.001G149000 Sapur.001G149100 Sapur.001G167600 Sapur.001G193000 Sapur.002G019400 Sapur.002G106000 Sapur.002G107000 Sapur.002G141800 Sapur.003G003700

				Sapur.003G037400 Sapur.003G037500 Sapur.003G082800 Sapur.003G093200 Sapur.003G109000 Sapur.003G130700 Sapur.004G088800 Sapur.004G096100 Sapur.004G139700 Sapur.005G055700 Sapur.005G089700 Sapur.005G095300 Sapur.005G115200 Sapur.006G045100 Sapur.006G045200 Sapur.006G098500 Sapur.006G116900 Sapur.006G186800 Sapur.007G049700 Sapur.007G078000 Sapur.007G080700 Sapur.007G080800 Sapur.007G103200 Sapur.007G108700 Sapur.008G166400 Sapur.009G030600 Sapur.009G039700 Sapur.009G053600 Sapur.009G079400 Sapur.009G085400 Sapur.009G085600 Sapur.009G085700 Sapur.009G086400 Sapur.009G087200 Sapur.010G021800 Sapur.010G022000 Sapur.010G088800 Sapur.010G088900 Sapur.010G109100 Sapur.010G109200 Sapur.010G149000 Sapur.011G066700 Sapur.011G097000 Sapur.012G043000 Sapur.012G066500 Sapur.013G080500 Sapur.013G084400 Sapur.013G140300 Sapur.014G011100 Sapur.014G011200 Sapur.014G011900 Sapur.014G027000 Sapur.014G027100 Sapur.014G027200 Sapur.014G027400 Sapur.014G027800 Sapur.014G028400 Sapur.014G028500 Sapur.014G029000 Sapur.014G080900 Sapur.014G126900 Sapur.016G007300 Sapur.016G007700 Sapur.016G007900 Sapur.016G028200 Sapur.016G055400 Sapur.016G144700 Sapur.016G205100 Sapur.016G209200 Sapur.016G285400 Sapur.016G285500 Sapur.017G028400 Sapur.017G028500 Sapur.019G065400 Sapur.15ZG002400 Sapur.15ZG002500 Sapur.15ZG127500 Sapur.T047000 Sapur.T073900 Sapur.T131000
GO:0020037	91	454	heme binding	Sapur.001G067900 Sapur.001G148900 Sapur.001G149000 Sapur.001G149100 Sapur.001G167600 Sapur.001G193000 Sapur.002G019400 Sapur.002G106000 Sapur.002G107000 Sapur.002G141800 Sapur.003G003700 Sapur.003G037400 Sapur.003G037500 Sapur.003G082800 Sapur.003G093200 Sapur.003G109000 Sapur.003G130700 Sapur.004G088800 Sapur.004G096100 Sapur.004G139700 Sapur.005G055700 Sapur.005G089700 Sapur.005G095300 Sapur.005G115200 Sapur.006G045100 Sapur.006G045200 Sapur.006G098500 Sapur.006G116900 Sapur.006G186800 Sapur.007G049700 Sapur.007G078000 Sapur.007G080700 Sapur.007G080800 Sapur.007G103200 Sapur.007G108700 Sapur.008G166400 Sapur.009G030600 Sapur.009G039700 Sapur.009G053600 Sapur.009G079400 Sapur.009G085400 Sapur.009G085600 Sapur.009G085700 Sapur.009G086400 Sapur.009G087200 Sapur.010G021800 Sapur.010G022000 Sapur.010G088800 Sapur.010G088900 Sapur.010G109100 Sapur.010G109200 Sapur.010G149000 Sapur.011G066700 Sapur.011G097000 Sapur.012G043000 Sapur.012G066500 Sapur.013G080500 Sapur.013G084400 Sapur.013G140300 Sapur.014G011100 Sapur.014G011200 Sapur.014G011900 Sapur.014G027000 Sapur.014G027100 Sapur.014G027200 Sapur.014G027400 Sapur.014G027800 Sapur.014G028400 Sapur.014G028500 Sapur.014G029000 Sapur.014G080900 Sapur.014G126900 Sapur.016G007300 Sapur.016G007700 Sapur.016G007900 Sapur.016G028200 Sapur.016G055400 Sapur.016G144700 Sapur.016G205100 Sapur.016G209200 Sapur.016G285400 Sapur.016G285500 Sapur.017G028400 Sapur.017G028500 Sapur.019G065400 Sapur.15ZG002400 Sapur.15ZG002500 Sapur.15ZG127500 Sapur.T047000 Sapur.T073900 Sapur.T131000
GO:0051540	18	69	metal cluster binding	Sapur.001G004400 Sapur.001G004500 Sapur.001G177000 Sapur.002G066500 Sapur.003G166200 Sapur.004G144500 Sapur.006G220900 Sapur.008G044200 Sapur.009G032000 Sapur.009G039700 Sapur.009G079400 Sapur.009G120800 Sapur.012G069200 Sapur.012G100300 Sapur.016G139500 Sapur.016G144700 Sapur.018G011700

				Sapur.15ZG085300
GO:0051536	18	69	iron-sulfur cluster binding	Sapur.001G004400 Sapur.001G004500 Sapur.001G177000 Sapur.002G066500 Sapur.003G166200 Sapur.004G144500 Sapur.006G220900 Sapur.008G044200 Sapur.009G032000 Sapur.009G039700 Sapur.009G079400 Sapur.009G120800 Sapur.012G069200 Sapur.012G100300 Sapur.016G139500 Sapur.016G144700 Sapur.018G011700 Sapur.15ZG085300
GO:0046915	4	8	transition metal ion transmembrane transporter activity	Sapur.005G009200 Sapur.006G076500 Sapur.009G028800 Sapur.009G028900
GO:0016814	4	8	hydrolase activity, acting on carbon-nitrogen (but not peptide) bonds, in cyclic amidines	Sapur.006G083400 Sapur.010G086900 Sapur.016G104000 Sapur.018G047900
GO:0016149	4	8	translation release factor activity, codon specific	Sapur.003G117300 Sapur.006G033400 Sapur.008G060200 Sapur.013G008600
GO:0019203	3	5	carbohydrate phosphatase activity	Sapur.005G150700 Sapur.016G094400 Sapur.017G031900
GO:0003896	3	5	DNA primase activity	Sapur.003G070500 Sapur.017G119700 Sapur.15WG074800
GO:0050308	3	5	sugar-phosphatase activity	Sapur.005G150700 Sapur.016G094400 Sapur.017G031900
GO:0004066	3	5	asparagine synthase (glutamine-hydrolyzing) activity	Sapur.005G058900 Sapur.009G056800 Sapur.010G121200
GO:0042132	3	5	fructose 1,6-bisphosphate	Sapur.005G150700 Sapur.016G094400 Sapur.017G031900

			1-phosphatase activity	
GO:0005516	10	33	calmodulin binding	Sapur.001G020500 Sapur.003G155100 Sapur.003G156600 Sapur.005G047900 Sapur.008G004200 Sapur.011G028600 Sapur.012G037700 Sapur.013G009700 Sapur.018G106800 Sapur.15ZG049200
GO:0003690	17	67	double-stranded DNA binding	Sapur.001G027900 Sapur.001G028100 Sapur.001G028400 Sapur.001G179800 Sapur.003G145700 Sapur.004G007000 Sapur.004G027800 Sapur.006G168000 Sapur.007G001300 Sapur.007G062200 Sapur.010G046700 Sapur.011G054600 Sapur.012G000700 Sapur.012G031400 Sapur.014G104700 Sapur.017G057100 Sapur.019G075900
GO:0016701	10	34	oxidoreductase activity, acting on single donors with incorporation of molecular oxygen	Sapur.001G130300 Sapur.005G026800 Sapur.005G026900 Sapur.008G116400 Sapur.008G143900 Sapur.013G058600 Sapur.016G050900 Sapur.018G050900 Sapur.T007600 Sapur.T045600
GO:0032296	4	9	double-stranded RNA-specific ribonuclease activity	Sapur.001G167800 Sapur.006G154200 Sapur.008G060600 Sapur.010G141100
GO:0004525	4	9	ribonuclease III activity	Sapur.001G167800 Sapur.006G154200 Sapur.008G060600 Sapur.010G141100
