# Supplementary material for: Insights into Molecular Mechanism of Secondary Xylem Rapid Growth in Salix psammophila
Source: Plants (Basel). 2025 Feb 5;14(3):459. doi: 10.3390/plants14030459 (PMC11819810; doi:10.3390/plants14030459)
Supplement: Supplementary file 1 [file plants-14-00459-s001.zip › Supplementary Table/Table S5 .pdf]

Table S5 Comparison of phloem-specific differentially expressed genes GO annotation in 1, and 2-year-old.

	GOBPID	Count	Size	Term	gene_id
**r_down_BP**	GO:0007017	73	187	microtubule-based process	Sapur.001G086500 Sapur.002G085200 Sapur.002G085300 Sapur.002G089300 Sapur.002G093600 Sapur.002G182100 Sapur.003G082400 Sapur.003G090100 Sapur.003G091100 Sapur.003G092100 Sapur.003G148500 Sapur.004G019100 Sapur.004G071100 Sapur.004G109700 Sapur.004G120900 Sapur.004G122400 Sapur.004G147000 Sapur.005G015200 Sapur.005G022700 Sapur.005G024100 Sapur.005G025300 Sapur.005G029900 Sapur.005G093300 Sapur.006G025400 Sapur.006G029800 Sapur.006G036800 Sapur.006G036900 Sapur.006G065500 Sapur.006G067400 Sapur.006G069500 Sapur.006G112500 Sapur.006G159000 Sapur.006G172300 Sapur.007G123700 Sapur.008G137200 Sapur.009G018700 Sapur.009G029500 Sapur.009G052200 Sapur.009G122200 Sapur.010G042700 Sapur.010G119100 Sapur.010G206600 Sapur.011G018500 Sapur.011G103700 Sapur.011G119600 Sapur.011G122100 Sapur.012G037400 Sapur.012G040700 Sapur.012G099300 Sapur.013G021800 Sapur.013G071200 Sapur.014G005100 Sapur.014G051500 Sapur.014G100600 Sapur.014G121100 Sapur.016G051800 Sapur.016G052600 Sapur.016G071200 Sapur.016G077400 Sapur.016G127100 Sapur.016G138000 Sapur.016G172500 Sapur.016G305500 Sapur.017G006400 Sapur.017G006500 Sapur.017G067300 Sapur.017G075700 Sapur.018G057600 Sapur.15WG044400 Sapur.15WG062900 Sapur.15ZG049500 Sapur.15ZG122400 Sapur.T003900
	GO:0007018	50	106	microtubule-based movement	Sapur.002G085200 Sapur.002G085300 Sapur.002G089300 Sapur.002G093600 Sapur.002G182100 Sapur.003G092100 Sapur.004G019100 Sapur.004G109700 Sapur.004G120900 Sapur.004G122400 Sapur.004G147000 Sapur.005G015200 Sapur.005G022700 Sapur.005G024100 Sapur.005G025300 Sapur.005G029900 Sapur.005G093300 Sapur.006G029800 Sapur.006G036800 Sapur.006G036900 Sapur.006G067400 Sapur.006G069500 Sapur.006G112500 Sapur.006G159000 Sapur.006G172300 Sapur.008G137200 Sapur.009G018700 Sapur.009G122200 Sapur.010G042700 Sapur.010G119100 Sapur.011G018500 Sapur.011G103700 Sapur.011G122100 Sapur.012G037400 Sapur.012G040700 Sapur.013G021800 Sapur.013G071200 Sapur.014G005100 Sapur.014G100600 Sapur.014G121100 Sapur.016G051800 Sapur.016G052600 Sapur.016G071200 Sapur.016G077400 Sapur.016G127100 Sapur.016G305500 Sapur.018G057600 Sapur.15WG044400 Sapur.15ZG049500 Sapur.T003900
	GO:0000278	18	31	mitotic cell cycle	Sapur.001G103000 Sapur.003G082400 Sapur.004G060500 Sapur.004G071100 Sapur.005G207000 Sapur.006G030400 Sapur.006G065500 Sapur.007G123700 Sapur.008G057000 Sapur.010G008600 Sapur.010G056400 Sapur.010G145400 Sapur.014G127000 Sapur.017G006400 Sapur.017G006500 Sapur.017G075700 Sapur.15WG061400 Sapur.15WG062900
	GO:1903047	17	30	mitotic cell cycle process	Sapur.001G103000 Sapur.003G082400 Sapur.004G060500 Sapur.004G071100 Sapur.005G207000 Sapur.006G030400 Sapur.007G123700 Sapur.008G057000 Sapur.010G008600 Sapur.010G056400 Sapur.010G145400 Sapur.014G127000 Sapur.017G006400 Sapur.017G006500 Sapur.017G075700 Sapur.15WG061400 Sapur.15WG062900
	GO:0022402	20	48	cell cycle process	Sapur.001G103000 Sapur.003G082400 Sapur.004G060500 Sapur.004G071100 Sapur.004G163000 Sapur.005G207000 Sapur.006G030400 Sapur.006G065500 Sapur.007G123700 Sapur.008G057000 Sapur.009G003100 Sapur.010G008600 Sapur.010G056400 Sapur.010G145400 Sapur.014G127000 Sapur.017G006400 Sapur.017G006500 Sapur.017G075700 Sapur.15WG061400 Sapur.15WG062900
	GO:0000280	12	20	nuclear division	Sapur.001G103000 Sapur.004G060500 Sapur.004G163000 Sapur.005G207000 Sapur.006G030400 Sapur.008G057000 Sapur.009G003100 Sapur.010G008600 Sapur.010G056400 Sapur.010G145400 Sapur.014G127000 Sapur.15WG061400
	GO:0007049	24	89	cell cycle	Sapur.001G103000 Sapur.003G082400 Sapur.004G060500 Sapur.004G071100 Sapur.004G163000 Sapur.005G207000 Sapur.006G030400 Sapur.006G065500 Sapur.006G194600 Sapur.007G123700 Sapur.008G057000 Sapur.009G001600 Sapur.009G003100 Sapur.010G008600 Sapur.010G056400 Sapur.010G075900 Sapur.010G145400 Sapur.014G127000 Sapur.014G144100 Sapur.017G006400 Sapur.017G006500 Sapur.017G075700 Sapur.15WG061400 Sapur.15WG062900
	GO:0098813	12	21	nuclear chromosome segregation	Sapur.001G103000 Sapur.004G060500 Sapur.004G163000 Sapur.005G207000 Sapur.006G030400 Sapur.008G057000 Sapur.009G003100 Sapur.010G008600 Sapur.010G056400 Sapur.010G145400 Sapur.014G127000 Sapur.15WG061400
	GO:0006	19	59	DNA replication	Sapur.001G055600 Sapur.001G058800 Sapur.003G070500 Sapur.003G126100 Sapur.004G103000 Sapur.004G139900 Sapur.007G042700

260				Sapur.009G096500 Sapur.009G107100 Sapur.010G053800 Sapur.010G193700 Sapur.011G056100 Sapur.012G025800 Sapur.014G066300 Sapur.014G096700 Sapur.018G087600 Sapur.019G024300 Sapur.15WG072800 Sapur.15ZG043500
GO:0140014	10	15	mitotic nuclear division	Sapur.001G103000 Sapur.004G060500 Sapur.005G207000 Sapur.006G030400 Sapur.008G057000 Sapur.010G008600 Sapur.010G056400 Sapur.010G145400 Sapur.014G127000 Sapur.15WG061400
GO:0000070	10	15	mitotic sister chromatid segregation	Sapur.001G103000 Sapur.004G060500 Sapur.005G207000 Sapur.006G030400 Sapur.008G057000 Sapur.010G008600 Sapur.010G056400 Sapur.010G145400 Sapur.014G127000 Sapur.15WG061400
GO:0010564	13	29	regulation of cell cycle process	Sapur.003G082400 Sapur.004G071100 Sapur.005G207000 Sapur.006G065500 Sapur.007G123700 Sapur.008G057000 Sapur.010G056400 Sapur.010G145400 Sapur.017G006400 Sapur.017G006500 Sapur.017G075700 Sapur.15WG061400 Sapur.15WG062900
GO:0000819	10	17	sister chromatid segregation	Sapur.001G103000 Sapur.004G060500 Sapur.005G207000 Sapur.006G030400 Sapur.008G057000 Sapur.010G008600 Sapur.010G056400 Sapur.010G145400 Sapur.014G127000 Sapur.15WG061400
GO:0007059	13	32	chromosome segregation	Sapur.001G103000 Sapur.004G060500 Sapur.004G163000 Sapur.005G207000 Sapur.006G030400 Sapur.006G065500 Sapur.008G057000 Sapur.009G003100 Sapur.010G008600 Sapur.010G056400 Sapur.010G145400 Sapur.014G127000 Sapur.15WG061400
GO:0048285	12	31	organelle fission	Sapur.001G103000 Sapur.004G060500 Sapur.004G163000 Sapur.005G207000 Sapur.006G030400 Sapur.008G057000 Sapur.009G003100 Sapur.010G008600 Sapur.010G056400 Sapur.010G145400 Sapur.014G127000 Sapur.15WG061400
GO:0033043	12	33	regulation of organelle organization	Sapur.003G082400 Sapur.004G071100 Sapur.005G207000 Sapur.007G123700 Sapur.008G057000 Sapur.010G056400 Sapur.010G145400 Sapur.017G006400 Sapur.017G006500 Sapur.017G075700 Sapur.15WG061400 Sapur.15WG062900
GO:0000226	13	40	microtubule cytoskeleton organization	Sapur.003G082400 Sapur.003G148500 Sapur.004G071100 Sapur.006G065500 Sapur.007G123700 Sapur.010G206600 Sapur.012G099300 Sapur.014G051500 Sapur.017G006400 Sapur.017G006500 Sapur.017G075700 Sapur.15WG062900 Sapur.15ZG122400
GO:0001934	7	10	positive regulation of protein phosphorylation	Sapur.003G082400 Sapur.004G071100 Sapur.007G123700 Sapur.017G006400 Sapur.017G006500 Sapur.017G075700 Sapur.15WG062900
GO:0032147	7	10	activation of protein kinase activity	Sapur.003G082400 Sapur.004G071100 Sapur.007G123700 Sapur.017G006400 Sapur.017G006500 Sapur.017G075700 Sapur.15WG062900
GO:0090224	7	10	regulation of spindle organization	Sapur.003G082400 Sapur.004G071100 Sapur.007G123700 Sapur.017G006400 Sapur.017G006500 Sapur.017G075700 Sapur.15WG062900
GO:0045937	7	10	positive regulation of phosphate metabolic process	Sapur.003G082400 Sapur.004G071100 Sapur.007G123700 Sapur.017G006400 Sapur.017G006500 Sapur.017G075700 Sapur.15WG062900
GO:0042327	7	10	positive regulation of phosphorylation	Sapur.003G082400 Sapur.004G071100 Sapur.007G123700 Sapur.017G006400 Sapur.017G006500 Sapur.017G075700 Sapur.15WG062900
GO:0033674	7	10	positive regulation of kinase activity	Sapur.003G082400 Sapur.004G071100 Sapur.007G123700 Sapur.017G006400 Sapur.017G006500 Sapur.017G075700 Sapur.15WG062900
GO:0060236	7	10	regulation of mitotic spindle organization	Sapur.003G082400 Sapur.004G071100 Sapur.007G123700 Sapur.017G006400 Sapur.017G006500 Sapur.017G075700 Sapur.15WG062900
GO:0031401	7	10	positive regulation of protein	Sapur.003G082400 Sapur.004G071100 Sapur.007G123700 Sapur.017G006400 Sapur.017G006500 Sapur.017G075700 Sapur.15WG062900

			modification process	
GO:0045860	7	10	positive regulation of protein kinase activity	Sapur.003G082400 Sapur.004G071100 Sapur.007G123700 Sapur.017G006400 Sapur.017G006500 Sapur.017G075700 Sapur.15WG062900
GO:0051347	7	10	positive regulation of transferase activity	Sapur.003G082400 Sapur.004G071100 Sapur.007G123700 Sapur.017G006400 Sapur.017G006500 Sapur.017G075700 Sapur.15WG062900
GO:1902850	7	10	microtubule cytoskeleton organization involved in mitosis	Sapur.003G082400 Sapur.004G071100 Sapur.007G123700 Sapur.017G006400 Sapur.017G006500 Sapur.017G075700 Sapur.15WG062900
GO:0010562	7	10	positive regulation of phosphorus metabolic process	Sapur.003G082400 Sapur.004G071100 Sapur.007G123700 Sapur.017G006400 Sapur.017G006500 Sapur.017G075700 Sapur.15WG062900
GO:0007052	7	10	mitotic spindle organization	Sapur.003G082400 Sapur.004G071100 Sapur.007G123700 Sapur.017G006400 Sapur.017G006500 Sapur.017G075700 Sapur.15WG062900
GO:0043085	7	11	positive regulation of catalytic activity	Sapur.003G082400 Sapur.004G071100 Sapur.007G123700 Sapur.017G006400 Sapur.017G006500 Sapur.017G075700 Sapur.15WG062900
GO:0032886	7	11	regulation of microtubule-based process	Sapur.003G082400 Sapur.004G071100 Sapur.007G123700 Sapur.017G006400 Sapur.017G006500 Sapur.017G075700 Sapur.15WG062900
GO:0070507	7	11	regulation of microtubule cytoskeleton organization	Sapur.003G082400 Sapur.004G071100 Sapur.007G123700 Sapur.017G006400 Sapur.017G006500 Sapur.017G075700 Sapur.15WG062900
GO:0044093	7	11	positive regulation of molecular function	Sapur.003G082400 Sapur.004G071100 Sapur.007G123700 Sapur.017G006400 Sapur.017G006500 Sapur.017G075700 Sapur.15WG062900
GO:0051128	12	37	regulation of cellular component organization	Sapur.003G082400 Sapur.004G071100 Sapur.005G207000 Sapur.007G123700 Sapur.008G057000 Sapur.010G056400 Sapur.010G145400 Sapur.017G006400 Sapur.017G006500 Sapur.017G075700 Sapur.15WG061400 Sapur.15WG062900
GO:0030261	5	5	chromosome condensation	Sapur.001G103000 Sapur.004G060500 Sapur.006G030400 Sapur.010G008600 Sapur.014G127000
GO:0007076	5	5	mitotic chromosome condensation	Sapur.001G103000 Sapur.004G060500 Sapur.006G030400 Sapur.010G008600 Sapur.014G127000
GO:0051726	13	44	regulation of cell cycle	Sapur.003G082400 Sapur.004G071100 Sapur.005G207000 Sapur.006G065500 Sapur.007G123700 Sapur.008G057000 Sapur.010G056400 Sapur.010G145400 Sapur.017G006400 Sapur.017G006500 Sapur.017G075700 Sapur.15WG061400 Sapur.15WG062900
GO:0051247	7	14	positive regulation of protein metabolic	Sapur.003G082400 Sapur.004G071100 Sapur.007G123700 Sapur.017G006400 Sapur.017G006500 Sapur.017G075700 Sapur.15WG062900

			process	
GO:0007051	7	17	spindle organization	Sapur.003G082400 Sapur.004G071100 Sapur.007G123700 Sapur.017G006400 Sapur.017G006500 Sapur.017G075700 Sapur.15WG062900
GO:0006261	5	8	DNA-templated DNA replication	Sapur.001G055600 Sapur.001G058800 Sapur.003G070500 Sapur.003G126100 Sapur.014G096700
GO:0031325	7	18	positive regulation of cellular metabolic process	Sapur.003G082400 Sapur.004G071100 Sapur.007G123700 Sapur.017G006400 Sapur.017G006500 Sapur.017G075700 Sapur.15WG062900
GO:0009893	7	18	positive regulation of metabolic process	Sapur.003G082400 Sapur.004G071100 Sapur.007G123700 Sapur.017G006400 Sapur.017G006500 Sapur.017G075700 Sapur.15WG062900
GO:0051173	7	18	positive regulation of nitrogen compound metabolic process	Sapur.003G082400 Sapur.004G071100 Sapur.007G123700 Sapur.017G006400 Sapur.017G006500 Sapur.017G075700 Sapur.15WG062900
GO:0010604	7	18	positive regulation of macromolecule metabolic process	Sapur.003G082400 Sapur.004G071100 Sapur.007G123700 Sapur.017G006400 Sapur.017G006500 Sapur.017G075700 Sapur.15WG062900
GO:0006996	32	260	organelle organization	Sapur.001G103000 Sapur.003G082400 Sapur.003G148500 Sapur.004G060500 Sapur.004G071100 Sapur.004G163000 Sapur.005G005600 Sapur.005G207000 Sapur.006G028600 Sapur.006G030400 Sapur.006G065500 Sapur.007G123700 Sapur.008G057000 Sapur.009G003100 Sapur.009G033900 Sapur.009G045200 Sapur.010G008600 Sapur.010G056400 Sapur.010G145400 Sapur.010G206600 Sapur.012G099300 Sapur.014G051500 Sapur.014G127000 Sapur.016G152100 Sapur.016G193800 Sapur.017G006400 Sapur.017G006500 Sapur.017G075700 Sapur.15WG061400 Sapur.15WG062900 Sapur.15ZG122400 Sapur.15ZG061300
GO:0006468	127	1578	protein phosphorylation	Sapur.001G038800 Sapur.001G078000 Sapur.001G096300 Sapur.001G103900 Sapur.001G126100 Sapur.002G002000 Sapur.002G005900 Sapur.002G020300 Sapur.002G051400 Sapur.002G060000 Sapur.002G106900 Sapur.002G120400 Sapur.002G188100 Sapur.002G195600 Sapur.003G037300 Sapur.003G051100 Sapur.003G056800 Sapur.003G061800 Sapur.003G073200 Sapur.003G079800 Sapur.003G082400 Sapur.003G099700 Sapur.003G139700 Sapur.004G029200 Sapur.004G032900 Sapur.004G059100 Sapur.004G067800 Sapur.004G071100 Sapur.004G170800 Sapur.005G001800 Sapur.005G027300 Sapur.005G050800 Sapur.005G109200 Sapur.005G188000 Sapur.005G206400 Sapur.006G040500 Sapur.006G044000 Sapur.006G077000 Sapur.006G085200 Sapur.006G090100 Sapur.006G092500 Sapur.006G093700 Sapur.006G096300 Sapur.006G107000 Sapur.006G188300 Sapur.006G194600 Sapur.006G195000 Sapur.007G012300 Sapur.007G045900 Sapur.007G099800 Sapur.007G123700 Sapur.007G127200 Sapur.008G016100 Sapur.008G054500 Sapur.008G113700 Sapur.008G143200 Sapur.008G157700 Sapur.009G130800 Sapur.010G028600 Sapur.010G041500 Sapur.010G057900 Sapur.010G076000 Sapur.010G143200 Sapur.010G147600 Sapur.010G171000 Sapur.010G189000 Sapur.011G002200 Sapur.011G002300 Sapur.011G024200 Sapur.011G046900 Sapur.011G047800 Sapur.011G072400 Sapur.011G080600 Sapur.011G103500 Sapur.012G020400 Sapur.012G029700 Sapur.012G048500 Sapur.012G051500 Sapur.012G052000 Sapur.012G067300 Sapur.012G097700 Sapur.012G098500 Sapur.013G003400 Sapur.013G020900 Sapur.013G028400 Sapur.013G134300 Sapur.013G141900 Sapur.014G063400 Sapur.014G106100 Sapur.014G126200 Sapur.016G045300 Sapur.016G046200 Sapur.016G049500 Sapur.016G109100 Sapur.016G191700 Sapur.016G220500 Sapur.016G233300 Sapur.016G263500 Sapur.016G274100 Sapur.016G294500 Sapur.017G006400 Sapur.017G006500 Sapur.017G021900 Sapur.017G075700 Sapur.017G107900 Sapur.018G018700 Sapur.018G023200 Sapur.018G035500 Sapur.018G053200 Sapur.018G082400 Sapur.019G008900 Sapur.019G048400 Sapur.019G058700 Sapur.019G061700 Sapur.019G107300 Sapur.019G113000 Sapur.15WG028200 Sapur.15WG062900 Sapur.15WG063200

				Sapur.15WG079300 Sapur.15ZG005000 Sapur.15ZG013600 Sapur.15ZG018000 Sapur.15ZG027900 Sapur.15ZG068100 Sapur.15ZG121300 Sapur.T191300
GO:0016310	127	1593	phosphorylation	Sapur.001G038800 Sapur.001G078000 Sapur.001G096300 Sapur.001G103900 Sapur.001G126100 Sapur.002G002000 Sapur.002G005900 Sapur.002G020300 Sapur.002G051400 Sapur.002G060000 Sapur.002G106900 Sapur.002G120400 Sapur.002G188100 Sapur.002G195600 Sapur.003G037300 Sapur.003G051100 Sapur.003G056800 Sapur.003G061800 Sapur.003G073200 Sapur.003G079800 Sapur.003G082400 Sapur.003G099700 Sapur.003G139700 Sapur.004G029200 Sapur.004G032900 Sapur.004G059100 Sapur.004G067800 Sapur.004G071100 Sapur.004G170800 Sapur.005G001800 Sapur.005G027300 Sapur.005G050800 Sapur.005G109200 Sapur.005G188000 Sapur.005G206400 Sapur.006G040500 Sapur.006G044000 Sapur.006G077000 Sapur.006G085200 Sapur.006G090100 Sapur.006G092500 Sapur.006G093700 Sapur.006G096300 Sapur.006G107000 Sapur.006G188300 Sapur.006G194600 Sapur.006G195000 Sapur.007G012300 Sapur.007G045900 Sapur.007G099800 Sapur.007G123700 Sapur.007G127200 Sapur.008G016100 Sapur.008G054500 Sapur.008G113700 Sapur.008G143200 Sapur.008G157700 Sapur.009G130800 Sapur.010G028600 Sapur.010G041500 Sapur.010G057900 Sapur.010G076000 Sapur.010G143200 Sapur.010G147600 Sapur.010G171000 Sapur.010G189000 Sapur.011G002200 Sapur.011G002300 Sapur.011G024200 Sapur.011G046900 Sapur.011G047800 Sapur.011G072400 Sapur.011G080600 Sapur.011G103500 Sapur.012G020400 Sapur.012G029700 Sapur.012G048500 Sapur.012G051500 Sapur.012G052000 Sapur.012G067300 Sapur.012G097700 Sapur.012G098500 Sapur.013G003400 Sapur.013G020900 Sapur.013G028400 Sapur.013G134300 Sapur.013G141900 Sapur.014G063400 Sapur.014G106100 Sapur.014G126200 Sapur.016G045300 Sapur.016G046200 Sapur.016G049500 Sapur.016G109100 Sapur.016G191700 Sapur.016G220500 Sapur.016G233300 Sapur.016G263500 Sapur.016G274100 Sapur.016G294500 Sapur.017G006400 Sapur.017G006500 Sapur.017G021900 Sapur.017G075700 Sapur.017G107900 Sapur.018G018700 Sapur.018G023200 Sapur.018G035500 Sapur.018G053200 Sapur.018G082400 Sapur.019G008900 Sapur.019G048400 Sapur.019G058700 Sapur.019G061700 Sapur.019G107300 Sapur.019G113000 Sapur.15WG028200 Sapur.15WG062900 Sapur.15WG063200 Sapur.15WG079300 Sapur.15ZG005000 Sapur.15ZG013600 Sapur.15ZG018000 Sapur.15ZG027900 Sapur.15ZG068100 Sapur.15ZG121300 Sapur.T191300
GO:0007010	16	93	cytoskeleton organization	Sapur.003G082400 Sapur.003G148500 Sapur.004G071100 Sapur.006G028600 Sapur.006G065500 Sapur.007G123700 Sapur.009G033900 Sapur.010G206600 Sapur.012G099300 Sapur.014G051500 Sapur.017G006400 Sapur.017G006500 Sapur.017G075700 Sapur.15WG062900 Sapur.15ZG122400 Sapur.15ZG061300
GO:0048522	7	20	positive regulation of cellular process	Sapur.003G082400 Sapur.004G071100 Sapur.007G123700 Sapur.017G006400 Sapur.017G006500 Sapur.017G075700 Sapur.15WG062900
GO:0048518	7	20	positive regulation of biological process	Sapur.003G082400 Sapur.004G071100 Sapur.007G123700 Sapur.017G006400 Sapur.017G006500 Sapur.017G075700 Sapur.15WG062900
GO:0001932	7	21	regulation of protein phosphorylation	Sapur.003G082400 Sapur.004G071100 Sapur.007G123700 Sapur.017G006400 Sapur.017G006500 Sapur.017G075700 Sapur.15WG062900
GO:0045859	7	21	regulation of protein kinase activity	Sapur.003G082400 Sapur.004G071100 Sapur.007G123700 Sapur.017G006400 Sapur.017G006500 Sapur.017G075700 Sapur.15WG062900
GO:0051493	7	21	regulation of cytoskeleton organization	Sapur.003G082400 Sapur.004G071100 Sapur.007G123700 Sapur.017G006400 Sapur.017G006500 Sapur.017G075700 Sapur.15WG062900
GO:1905818	5	10	regulation of chromosome separation	Sapur.005G207000 Sapur.008G057000 Sapur.010G056400 Sapur.010G145400 Sapur.15WG061400
GO:1905	5	10	negative regulation	Sapur.005G207000 Sapur.008G057000 Sapur.010G056400 Sapur.010G145400 Sapur.15WG061400

819			of chromosome separation	
GO:0071173	5	10	spindle assembly checkpoint signaling	Sapur.005G207000 Sapur.008G057000 Sapur.010G056400 Sapur.010G145400 Sapur.15WG061400
GO:0071174	5	10	mitotic spindle checkpoint signaling	Sapur.005G207000 Sapur.008G057000 Sapur.010G056400 Sapur.010G145400 Sapur.15WG061400
GO:0051985	5	10	negative regulation of chromosome segregation	Sapur.005G207000 Sapur.008G057000 Sapur.010G056400 Sapur.010G145400 Sapur.15WG061400
GO:0051783	5	10	regulation of nuclear division	Sapur.005G207000 Sapur.008G057000 Sapur.010G056400 Sapur.010G145400 Sapur.15WG061400
GO:0051784	5	10	negative regulation of nuclear division	Sapur.005G207000 Sapur.008G057000 Sapur.010G056400 Sapur.010G145400 Sapur.15WG061400
GO:0031577	5	10	spindle checkpoint signaling	Sapur.005G207000 Sapur.008G057000 Sapur.010G056400 Sapur.010G145400 Sapur.15WG061400
GO:1902100	5	10	negative regulation of metaphase/anaphase transition of cell cycle	Sapur.005G207000 Sapur.008G057000 Sapur.010G056400 Sapur.010G145400 Sapur.15WG061400
GO:0010965	5	10	regulation of mitotic sister chromatid separation	Sapur.005G207000 Sapur.008G057000 Sapur.010G056400 Sapur.010G145400 Sapur.15WG061400
GO:2001251	5	10	negative regulation of chromosome organization	Sapur.005G207000 Sapur.008G057000 Sapur.010G056400 Sapur.010G145400 Sapur.15WG061400
GO:2000816	5	10	negative regulation of mitotic sister chromatid separation	Sapur.005G207000 Sapur.008G057000 Sapur.010G056400 Sapur.010G145400 Sapur.15WG061400
GO:0045841	5	10	negative regulation of mitotic metaphase/anaphase transition	Sapur.005G207000 Sapur.008G057000 Sapur.010G056400 Sapur.010G145400 Sapur.15WG061400
GO:0045839	5	10	negative regulation of mitotic nuclear division	Sapur.005G207000 Sapur.008G057000 Sapur.010G056400 Sapur.010G145400 Sapur.15WG061400
GO:0051306	5	10	mitotic sister chromatid	Sapur.005G207000 Sapur.008G057000 Sapur.010G056400 Sapur.010G145400 Sapur.15WG061400

			separation	
GO:0007094	5	10	mitotic spindle assembly checkpoint signaling	Sapur.005G207000 Sapur.008G057000 Sapur.010G056400 Sapur.010G145400 Sapur.15WG061400
GO:0007088	5	10	regulation of mitotic nuclear division	Sapur.005G207000 Sapur.008G057000 Sapur.010G056400 Sapur.010G145400 Sapur.15WG061400
GO:0033046	5	10	negative regulation of sister chromatid segregation	Sapur.005G207000 Sapur.008G057000 Sapur.010G056400 Sapur.010G145400 Sapur.15WG061400
GO:0033047	5	10	regulation of mitotic sister chromatid segregation	Sapur.005G207000 Sapur.008G057000 Sapur.010G056400 Sapur.010G145400 Sapur.15WG061400
GO:0033048	5	10	negative regulation of mitotic sister chromatid segregation	Sapur.005G207000 Sapur.008G057000 Sapur.010G056400 Sapur.010G145400 Sapur.15WG061400
GO:0051276	14	80	chromosome organization	Sapur.001G103000 Sapur.004G060500 Sapur.005G005600 Sapur.005G207000 Sapur.006G030400 Sapur.008G057000 Sapur.009G045200 Sapur.010G008600 Sapur.010G056400 Sapur.010G145400 Sapur.014G127000 Sapur.016G152100 Sapur.016G193800 Sapur.15WG061400
GO:0006259	32	282	DNA metabolic process	Sapur.001G055600 Sapur.001G058800 Sapur.002G108700 Sapur.003G070500 Sapur.003G126100 Sapur.004G103000 Sapur.004G139900 Sapur.005G005600 Sapur.006G018200 Sapur.006G111600 Sapur.007G042700 Sapur.008G128300 Sapur.009G045200 Sapur.009G096500 Sapur.009G107100 Sapur.010G053800 Sapur.010G094400 Sapur.010G193700 Sapur.011G056100 Sapur.012G025800 Sapur.014G031500 Sapur.014G066300 Sapur.014G096700 Sapur.016G152100 Sapur.016G193800 Sapur.018G087600 Sapur.019G024300 Sapur.15WG032400 Sapur.15WG072800 Sapur.15ZG036200 Sapur.15ZG043500 Sapur.15ZG131000
GO:1901991	5	11	negative regulation of mitotic cell cycle phase transition	Sapur.005G207000 Sapur.008G057000 Sapur.010G056400 Sapur.010G145400 Sapur.15WG061400
GO:0042325	7	23	regulation of phosphorylation	Sapur.003G082400 Sapur.004G071100 Sapur.007G123700 Sapur.017G006400 Sapur.017G006500 Sapur.017G075700 Sapur.15WG062900
GO:0051338	7	23	regulation of transferase activity	Sapur.003G082400 Sapur.004G071100 Sapur.007G123700 Sapur.017G006400 Sapur.017G006500 Sapur.017G075700 Sapur.15WG062900
GO:0043549	7	23	regulation of kinase activity	Sapur.003G082400 Sapur.004G071100 Sapur.007G123700 Sapur.017G006400 Sapur.017G006500 Sapur.017G075700 Sapur.15WG062900
GO:0031399	7	24	regulation of protein modification process	Sapur.003G082400 Sapur.004G071100 Sapur.007G123700 Sapur.017G006400 Sapur.017G006500 Sapur.017G075700 Sapur.15WG062900
GO:0044784	5	12	metaphase/anaphase transition of cell cycle	Sapur.005G207000 Sapur.008G057000 Sapur.010G056400 Sapur.010G145400 Sapur.15WG061400
GO:0051983	5	12	regulation of chromosome	Sapur.005G207000 Sapur.008G057000 Sapur.010G056400 Sapur.010G145400 Sapur.15WG061400

			segregation	
GO:1902099	5	12	regulation of metaphase/anaphase transition of cell cycle	Sapur.005G207000 Sapur.008G057000 Sapur.010G056400 Sapur.010G145400 Sapur.15WG061400
GO:0030071	5	12	regulation of mitotic metaphase/anaphase transition	Sapur.005G207000 Sapur.008G057000 Sapur.010G056400 Sapur.010G145400 Sapur.15WG061400
GO:0051304	5	12	chromosome separation	Sapur.005G207000 Sapur.008G057000 Sapur.010G056400 Sapur.010G145400 Sapur.15WG061400
GO:0007091	5	12	metaphase/anaphase transition of mitotic cell cycle	Sapur.005G207000 Sapur.008G057000 Sapur.010G056400 Sapur.010G145400 Sapur.15WG061400
GO:0051129	5	12	negative regulation of cellular component organization	Sapur.005G207000 Sapur.008G057000 Sapur.010G056400 Sapur.010G145400 Sapur.15WG061400
GO:0033044	5	12	regulation of chromosome organization	Sapur.005G207000 Sapur.008G057000 Sapur.010G056400 Sapur.010G145400 Sapur.15WG061400
GO:0033045	5	12	regulation of sister chromatid segregation	Sapur.005G207000 Sapur.008G057000 Sapur.010G056400 Sapur.010G145400 Sapur.15WG061400
GO:0010639	5	12	negative regulation of organelle organization	Sapur.005G207000 Sapur.008G057000 Sapur.010G056400 Sapur.010G145400 Sapur.15WG061400
GO:0044772	5	13	mitotic cell cycle phase transition	Sapur.005G207000 Sapur.008G057000 Sapur.010G056400 Sapur.010G145400 Sapur.15WG061400
GO:0045930	5	13	negative regulation of mitotic cell cycle	Sapur.005G207000 Sapur.008G057000 Sapur.010G056400 Sapur.010G145400 Sapur.15WG061400
GO:0007093	5	13	mitotic cell cycle checkpoint signaling	Sapur.005G207000 Sapur.008G057000 Sapur.010G056400 Sapur.010G145400 Sapur.15WG061400
GO:1901990	5	13	regulation of mitotic cell cycle phase transition	Sapur.005G207000 Sapur.008G057000 Sapur.010G056400 Sapur.010G145400 Sapur.15WG061400
GO:0051246	8	34	regulation of protein metabolic process	Sapur.003G082400 Sapur.004G071100 Sapur.007G123700 Sapur.017G006400 Sapur.017G006500 Sapur.017G075700 Sapur.15WG062900 Sapur.002G012200
GO:0019220	7	27	regulation of phosphate metabolic	Sapur.003G082400 Sapur.004G071100 Sapur.007G123700 Sapur.017G006400 Sapur.017G006500 Sapur.017G075700 Sapur.15WG062900

			process
GO:0051174	7	27	regulation of phosphorus metabolic process	Sapur.003G082400 Sapur.004G071100 Sapur.007G123700 Sapur.017G006400 Sapur.017G006500 Sapur.017G075700 Sapur.15WG062900
GO:0050790	7	29	regulation of catalytic activity	Sapur.003G082400 Sapur.004G071100 Sapur.007G123700 Sapur.017G006400 Sapur.017G006500 Sapur.017G075700 Sapur.15WG062900
GO:0007346	5	15	regulation of mitotic cell cycle	Sapur.005G207000 Sapur.008G057000 Sapur.010G056400 Sapur.010G145400 Sapur.15WG061400
GO:0036211	147	2018	protein modification process	Sapur.001G038800 Sapur.001G078000 Sapur.001G096300 Sapur.001G103900 Sapur.001G126100 Sapur.002G002000 Sapur.002G005900 Sapur.002G020300 Sapur.002G028900 Sapur.002G051400 Sapur.002G060000 Sapur.002G106900 Sapur.002G120400 Sapur.002G188100 Sapur.002G195600 Sapur.003G037300 Sapur.003G051100 Sapur.003G056800 Sapur.003G061800 Sapur.003G073200 Sapur.003G079800 Sapur.003G082400 Sapur.003G099700 Sapur.003G139700 Sapur.004G029200 Sapur.004G032900 Sapur.004G059100 Sapur.004G067800 Sapur.004G071100 Sapur.004G127600 Sapur.004G170800 Sapur.005G001800 Sapur.005G015300 Sapur.005G027300 Sapur.005G044000 Sapur.005G050800 Sapur.005G109200 Sapur.005G188000 Sapur.005G206400 Sapur.006G040500 Sapur.006G044000 Sapur.006G077000 Sapur.006G085200 Sapur.006G090100 Sapur.006G092500 Sapur.006G093700 Sapur.006G096300 Sapur.006G107000 Sapur.006G188300 Sapur.006G194600 Sapur.006G195000 Sapur.007G012300 Sapur.007G045900 Sapur.007G063800 Sapur.007G064100 Sapur.007G064200 Sapur.007G094200 Sapur.007G099400 Sapur.007G099800 Sapur.007G123700 Sapur.007G127200 Sapur.008G016100 Sapur.008G054500 Sapur.008G113700 Sapur.008G143200 Sapur.008G157700 Sapur.008G166200 Sapur.009G130800 Sapur.010G023000 Sapur.010G028600 Sapur.010G041500 Sapur.010G042000 Sapur.010G057900 Sapur.010G070800 Sapur.010G076000 Sapur.010G143200 Sapur.010G147600 Sapur.010G171000 Sapur.010G189000 Sapur.011G002200 Sapur.011G002300 Sapur.011G024200 Sapur.011G046900 Sapur.011G047800 Sapur.011G072400 Sapur.011G080600 Sapur.011G103500 Sapur.011G112000 Sapur.012G020400 Sapur.012G029700 Sapur.012G048500 Sapur.012G051500 Sapur.012G052000 Sapur.012G067300 Sapur.012G097700 Sapur.012G098500 Sapur.013G003400 Sapur.013G020900 Sapur.013G028400 Sapur.013G044800 Sapur.013G134300 Sapur.013G141900 Sapur.014G063400 Sapur.014G106100 Sapur.014G126200 Sapur.016G045300 Sapur.016G046200 Sapur.016G049500 Sapur.016G109100 Sapur.016G191700 Sapur.016G220500 Sapur.016G233300 Sapur.016G263500 Sapur.016G274100 Sapur.016G290200 Sapur.016G294500 Sapur.017G006400 Sapur.017G006500 Sapur.017G021900 Sapur.017G046600 Sapur.017G047500 Sapur.017G075700 Sapur.017G107900 Sapur.018G018700 Sapur.018G023200 Sapur.018G035500 Sapur.018G053200 Sapur.018G060600 Sapur.018G082400 Sapur.019G008900 Sapur.019G048400 Sapur.019G058700 Sapur.019G061700 Sapur.019G107300 Sapur.019G113000 Sapur.15WG028200 Sapur.15WG032000 Sapur.15WG062900 Sapur.15WG063200 Sapur.15WG079300 Sapur.15ZG005000 Sapur.15ZG013600 Sapur.15ZG018000 Sapur.15ZG027900 Sapur.15ZG068100 Sapur.15ZG121300 Sapur.T191300
GO:0065009	7	30	regulation of molecular function	Sapur.003G082400 Sapur.004G071100 Sapur.007G123700 Sapur.017G006400 Sapur.017G006500 Sapur.017G075700 Sapur.15WG062900
GO:0045786	5	16	negative regulation of cell cycle	Sapur.005G207000 Sapur.008G057000 Sapur.010G056400 Sapur.010G145400 Sapur.15WG061400
GO:0010948	5	16	negative regulation of cell cycle process	Sapur.005G207000 Sapur.008G057000 Sapur.010G056400 Sapur.010G145400 Sapur.15WG061400
GO:0000075	5	16	cell cycle checkpoint signaling	Sapur.005G207000 Sapur.008G057000 Sapur.010G056400 Sapur.010G145400 Sapur.15WG061400
GO:1901988	5	16	negative regulation of cell cycle phase	Sapur.005G207000 Sapur.008G057000 Sapur.010G056400 Sapur.010G145400 Sapur.15WG061400

			transition	
GO:0006270	3	5	DNA replication initiation	Sapur.001G055600 Sapur.001G058800 Sapur.014G096700
GO:0044770	5	18	cell cycle phase transition	Sapur.005G207000 Sapur.008G057000 Sapur.010G056400 Sapur.010G145400 Sapur.15WG061400
GO:1901987	5	18	regulation of cell cycle phase transition	Sapur.005G207000 Sapur.008G057000 Sapur.010G056400 Sapur.010G145400 Sapur.15WG061400
GO:0006269	2	2	DNA replication, synthesis of RNA primer	Sapur.003G070500 Sapur.003G126100
GO:0019953	3	6	sexual reproduction	Sapur.004G163000 Sapur.009G003100 Sapur.014G144100
GO:0022414	3	6	reproductive process	Sapur.004G163000 Sapur.009G003100 Sapur.014G144100
GO:0051321	3	6	meiotic cell cycle	Sapur.004G163000 Sapur.009G003100 Sapur.014G144100
GO:0000003	3	6	reproduction	Sapur.004G163000 Sapur.009G003100 Sapur.014G144100
GO:0043412	147	2067	macromolecule modification	Sapur.001G038800 Sapur.001G078000 Sapur.001G096300 Sapur.001G103900 Sapur.001G126100 Sapur.002G002000 Sapur.002G005900 Sapur.002G020300 Sapur.002G028900 Sapur.002G051400 Sapur.002G060000 Sapur.002G106900 Sapur.002G120400 Sapur.002G188100 Sapur.002G195600 Sapur.003G037300 Sapur.003G051100 Sapur.003G056800 Sapur.003G061800 Sapur.003G073200 Sapur.003G079800 Sapur.003G082400 Sapur.003G099700 Sapur.003G139700 Sapur.004G029200 Sapur.004G032900 Sapur.004G059100 Sapur.004G067800 Sapur.004G071100 Sapur.004G127600 Sapur.004G170800 Sapur.005G001800 Sapur.005G015300 Sapur.005G027300 Sapur.005G044000 Sapur.005G050800 Sapur.005G109200 Sapur.005G188000 Sapur.005G206400 Sapur.006G040500 Sapur.006G044000 Sapur.006G077000 Sapur.006G085200 Sapur.006G090100 Sapur.006G092500 Sapur.006G093700 Sapur.006G096300 Sapur.006G107000 Sapur.006G188300 Sapur.006G194600 Sapur.006G195000 Sapur.007G012300 Sapur.007G045900 Sapur.007G063800 Sapur.007G064100 Sapur.007G064200 Sapur.007G094200 Sapur.007G099400 Sapur.007G099800 Sapur.007G123700 Sapur.007G127200 Sapur.008G016100 Sapur.008G054500 Sapur.008G113700 Sapur.008G143200 Sapur.008G157700 Sapur.008G166200 Sapur.009G130800 Sapur.010G023000 Sapur.010G028600 Sapur.010G041500 Sapur.010G042000 Sapur.010G057900 Sapur.010G070800 Sapur.010G076000 Sapur.010G143200 Sapur.010G147600 Sapur.010G171000 Sapur.010G189000 Sapur.011G002200 Sapur.011G002300 Sapur.011G024200 Sapur.011G046900 Sapur.011G047800 Sapur.011G072400 Sapur.011G080600 Sapur.011G103500 Sapur.011G112000 Sapur.012G020400 Sapur.012G029700 Sapur.012G048500 Sapur.012G051500 Sapur.012G052000 Sapur.012G067300 Sapur.012G097700 Sapur.012G098500 Sapur.013G003400 Sapur.013G020900 Sapur.013G028400 Sapur.013G044800 Sapur.013G134300 Sapur.013G141900 Sapur.014G063400 Sapur.014G106100 Sapur.014G126200 Sapur.016G045300 Sapur.016G046200 Sapur.016G049500 Sapur.016G109100 Sapur.016G191700 Sapur.016G220500 Sapur.016G233300 Sapur.016G263500 Sapur.016G274100 Sapur.016G290200 Sapur.016G294500 Sapur.017G006400 Sapur.017G006500 Sapur.017G021900 Sapur.017G046600 Sapur.017G047500 Sapur.017G075700 Sapur.017G107900 Sapur.018G018700 Sapur.018G023200 Sapur.018G035500 Sapur.018G053200 Sapur.018G060600 Sapur.018G082400 Sapur.019G008900 Sapur.019G048400 Sapur.019G058700 Sapur.019G061700 Sapur.019G107300 Sapur.019G113000 Sapur.15WG028200 Sapur.15WG032000 Sapur.15WG062900 Sapur.15WG063200 Sapur.15WG079300

				Sapur.15ZG005000 Sapur.15ZG013600 Sapur.15ZG018000 Sapur.15ZG027900 Sapur.15ZG068100 Sapur.15ZG121300 Sapur.T191300
GO:0071103	4	13	DNA conformation change	Sapur.005G005600 Sapur.009G045200 Sapur.016G152100 Sapur.016G193800
GO:0006265	4	13	DNA topological change	Sapur.005G005600 Sapur.009G045200 Sapur.016G152100 Sapur.016G193800
GO:0005975	46	538	carbohydrate metabolic process	Sapur.001G112100 Sapur.001G167900 Sapur.001G171300 Sapur.002G004700 Sapur.002G048300 Sapur.002G182500 Sapur.002G192300 Sapur.003G013400 Sapur.003G118000 Sapur.003G165600 Sapur.004G061300 Sapur.005G190700 Sapur.006G114600 Sapur.006G180700 Sapur.007G051800 Sapur.007G089900 Sapur.008G043800 Sapur.008G062900 Sapur.008G076700 Sapur.008G107800 Sapur.008G112200 Sapur.009G059800 Sapur.009G066500 Sapur.009G120900 Sapur.010G071500 Sapur.010G081700 Sapur.010G120700 Sapur.011G072100 Sapur.011G112600 Sapur.013G135900 Sapur.014G071900 Sapur.014G072000 Sapur.014G091800 Sapur.014G097700 Sapur.014G101600 Sapur.014G129200 Sapur.016G040000 Sapur.017G066600 Sapur.018G070800 Sapur.019G037200 Sapur.019G068900 Sapur.019G109000 Sapur.15WG032300 Sapur.15ZG036100 Sapur.15ZG060400 Sapur.T171300
GO:0006796	137	1954	phosphate-containing compound metabolic process	Sapur.001G038800 Sapur.001G078000 Sapur.001G096300 Sapur.001G103900 Sapur.001G126100 Sapur.002G002000 Sapur.002G005900 Sapur.002G020300 Sapur.002G051400 Sapur.002G060000 Sapur.002G106900 Sapur.002G120400 Sapur.002G188100 Sapur.002G195600 Sapur.003G037300 Sapur.003G051100 Sapur.003G056800 Sapur.003G061800 Sapur.003G073200 Sapur.003G079800 Sapur.003G082400 Sapur.003G099700 Sapur.003G139700 Sapur.004G029200 Sapur.004G032900 Sapur.004G059100 Sapur.004G067800 Sapur.004G071100 Sapur.004G170800 Sapur.005G001800 Sapur.005G015300 Sapur.005G027300 Sapur.005G050800 Sapur.005G109200 Sapur.005G188000 Sapur.005G206400 Sapur.006G040500 Sapur.006G044000 Sapur.006G077000 Sapur.006G085200 Sapur.006G090100 Sapur.006G092500 Sapur.006G093700 Sapur.006G096300 Sapur.006G107000 Sapur.006G188300 Sapur.006G194600 Sapur.006G195000 Sapur.007G012300 Sapur.007G045900 Sapur.007G063800 Sapur.007G064100 Sapur.007G064200 Sapur.007G099800 Sapur.007G123700 Sapur.007G127200 Sapur.008G016100 Sapur.008G054500 Sapur.008G113700 Sapur.008G143200 Sapur.008G157700 Sapur.009G130800 Sapur.010G023000 Sapur.010G028600 Sapur.010G041500 Sapur.010G042000 Sapur.010G057900 Sapur.010G076000 Sapur.010G143200 Sapur.010G147600 Sapur.010G171000 Sapur.010G189000 Sapur.011G002200 Sapur.011G002300 Sapur.011G024200 Sapur.011G046900 Sapur.011G047800 Sapur.011G072400 Sapur.011G080600 Sapur.011G103500 Sapur.012G020400 Sapur.012G029700 Sapur.012G048500 Sapur.012G051500 Sapur.012G052000 Sapur.012G067300 Sapur.012G097700 Sapur.012G098500 Sapur.013G003400 Sapur.013G020900 Sapur.013G028400 Sapur.013G134300 Sapur.013G141900 Sapur.014G063400 Sapur.014G106100 Sapur.014G126200 Sapur.016G040000 Sapur.016G045300 Sapur.016G046200 Sapur.016G049500 Sapur.016G109100 Sapur.016G191700 Sapur.016G220500 Sapur.016G233300 Sapur.016G263500 Sapur.016G274100 Sapur.016G294500 Sapur.017G006400 Sapur.017G006500 Sapur.017G021900 Sapur.017G046600 Sapur.017G047500 Sapur.017G075700 Sapur.017G107900 Sapur.018G018700 Sapur.018G023200 Sapur.018G035500 Sapur.018G053200 Sapur.018G082400 Sapur.019G008900 Sapur.019G048400 Sapur.019G058700 Sapur.019G061700 Sapur.019G107300 Sapur.019G113000 Sapur.15WG028200 Sapur.15WG032000 Sapur.15WG062900 Sapur.15WG063200 Sapur.15WG079300 Sapur.15ZG005000 Sapur.15ZG013600 Sapur.15ZG018000 Sapur.15ZG027900 Sapur.15ZG068100 Sapur.15ZG121300 Sapur.T191300
GO:0006793	137	1955	phosphorus metabolic process	Sapur.001G038800 Sapur.001G078000 Sapur.001G096300 Sapur.001G103900 Sapur.001G126100 Sapur.002G002000 Sapur.002G005900 Sapur.002G020300 Sapur.002G051400 Sapur.002G060000 Sapur.002G106900 Sapur.002G120400 Sapur.002G188100 Sapur.002G195600 Sapur.003G037300 Sapur.003G051100 Sapur.003G056800 Sapur.003G061800 Sapur.003G073200 Sapur.003G079800 Sapur.003G082400 Sapur.003G099700 Sapur.003G139700 Sapur.004G029200 Sapur.004G032900 Sapur.004G059100 Sapur.004G067800 Sapur.004G071100 Sapur.004G170800 Sapur.005G001800 Sapur.005G015300 Sapur.005G027300 Sapur.005G050800 Sapur.005G109200 Sapur.005G188000 Sapur.005G206400 Sapur.006G040500 Sapur.006G044000 Sapur.006G077000 Sapur.006G085200 Sapur.006G090100 Sapur.006G092500 Sapur.006G093700 Sapur.006G096300 Sapur.006G107000 Sapur.006G188300 Sapur.006G194600 Sapur.006G195000 Sapur.007G012300

				Sapur.007G045900 Sapur.007G063800 Sapur.007G064100 Sapur.007G064200 Sapur.007G099800 Sapur.007G123700 Sapur.007G127200 Sapur.008G016100 Sapur.008G054500 Sapur.008G113700 Sapur.008G143200 Sapur.008G157700 Sapur.009G130800 Sapur.010G023000 Sapur.010G028600 Sapur.010G041500 Sapur.010G042000 Sapur.010G057900 Sapur.010G076000 Sapur.010G143200 Sapur.010G147600 Sapur.010G171000 Sapur.010G189000 Sapur.011G002200 Sapur.011G002300 Sapur.011G024200 Sapur.011G046900 Sapur.011G047800 Sapur.011G072400 Sapur.011G080600 Sapur.011G103500 Sapur.012G020400 Sapur.012G029700 Sapur.012G048500 Sapur.012G051500 Sapur.012G052000 Sapur.012G067300 Sapur.012G097700 Sapur.012G098500 Sapur.013G003400 Sapur.013G020900 Sapur.013G028400 Sapur.013G134300 Sapur.013G141900 Sapur.014G063400 Sapur.014G106100 Sapur.014G126200 Sapur.016G040000 Sapur.016G045300 Sapur.016G046200 Sapur.016G049500 Sapur.016G109100 Sapur.016G191700 Sapur.016G220500 Sapur.016G233300 Sapur.016G263500 Sapur.016G274100 Sapur.016G294500 Sapur.017G006400 Sapur.017G006500 Sapur.017G021900 Sapur.017G046600 Sapur.017G047500 Sapur.017G075700 Sapur.017G107900 Sapur.018G018700 Sapur.018G023200 Sapur.018G035500 Sapur.018G053200 Sapur.018G082400 Sapur.019G008900 Sapur.019G048400 Sapur.019G058700 Sapur.019G061700 Sapur.019G107300 Sapur.019G113000 Sapur.15WG028200 Sapur.15WG032000 Sapur.15WG062900 Sapur.15WG063200 Sapur.15WG079300 Sapur.15ZG005000 Sapur.15ZG013600 Sapur.15ZG018000 Sapur.15ZG027900 Sapur.15ZG068100 Sapur.15ZG121300 Sapur.T191300
GO:0045132	2	4	meiotic chromosome segregation	Sapur.004G163000 Sapur.009G003100
GO:0072330	9	70	monocarboxylic acid biosynthetic process	Sapur.001G095200 Sapur.003G080700 Sapur.004G051700 Sapur.004G098500 Sapur.006G131700 Sapur.006G206200 Sapur.011G119400 Sapur.018G026500 Sapur.018G075400
GO:0006633	9	70	fatty acid biosynthetic process	Sapur.001G095200 Sapur.003G080700 Sapur.004G051700 Sapur.004G098500 Sapur.006G131700 Sapur.006G206200 Sapur.011G119400 Sapur.018G026500 Sapur.018G075400
GO:1903046	2	5	meiotic cell cycle process	Sapur.004G163000 Sapur.009G003100
GO:0140013	2	5	meiotic nuclear division	Sapur.004G163000 Sapur.009G003100
GO:0015689	2	5	molybdate ion transport	Sapur.003G108000 Sapur.15ZG006400
GO:0016043	35	436	cellular component organization	Sapur.002G160400 Sapur.010G082600 Sapur.013G054700 Sapur.001G103000 Sapur.003G082400 Sapur.003G148500 Sapur.004G060500 Sapur.004G071100 Sapur.004G163000 Sapur.005G005600 Sapur.005G207000 Sapur.006G028600 Sapur.006G030400 Sapur.006G065500 Sapur.007G123700 Sapur.008G057000 Sapur.009G003100 Sapur.009G033900 Sapur.009G045200 Sapur.010G008600 Sapur.010G056400 Sapur.010G145400 Sapur.010G206600 Sapur.012G099300 Sapur.014G051500 Sapur.014G127000 Sapur.016G152100 Sapur.016G193800 Sapur.017G006400 Sapur.017G006500 Sapur.017G075700 Sapur.15WG061400 Sapur.15WG062900 Sapur.15ZG122400 Sapur.15ZG061300
GO:0048523	7	54	negative regulation of cellular process	Sapur.003G012100 Sapur.005G207000 Sapur.008G057000 Sapur.010G056400 Sapur.010G145400 Sapur.15WG061400 Sapur.15ZG109100
GO:0048519	7	54	negative regulation of biological process	Sapur.003G012100 Sapur.005G207000 Sapur.008G057000 Sapur.010G056400 Sapur.010G145400 Sapur.15WG061400 Sapur.15ZG109100
GO:0046500	2	6	S-adenosylmethionine metabolic process	Sapur.006G101400 Sapur.013G003500
GO:0008215	2	6	spermine metabolic process	Sapur.008G171500 Sapur.010G013500
GO:0006	2	6	spermine	Sapur.008G171500 Sapur.010G013500

	597			biosynthetic process	
	GO:0006556	2	6	S-adenosylmethionine biosynthetic process	Sapur.006G101400 Sapur.013G003500
	GO:0009116	4	30	nucleoside metabolic process	Sapur.013G094800 Sapur.013G094900 Sapur.013G095000 Sapur.013G095100
	GO:1901657	4	30	glycosyl compound metabolic process	Sapur.013G094800 Sapur.013G094900 Sapur.013G095000 Sapur.013G095100
	GO:0000041	3	15	transition metal ion transport	Sapur.006G084800 Sapur.016G107800 Sapur.009G028900
	GO:0098771	2	5	inorganic ion homeostasis	Sapur.006G084800 Sapur.016G107800
	GO:0006879	2	5	intracellular iron ion homeostasis	Sapur.006G084800 Sapur.016G107800
	GO:0030003	2	5	intracellular monoatomic cation homeostasis	Sapur.006G084800 Sapur.016G107800
	GO:0055080	2	5	monoatomic cation homeostasis	Sapur.006G084800 Sapur.016G107800
**r_up_BP**	GO:0050801	2	6	monoatomic ion homeostasis	Sapur.006G084800 Sapur.016G107800
	GO:0006873	2	6	intracellular monoatomic ion homeostasis	Sapur.006G084800 Sapur.016G107800
	GO:0006826	2	7	iron ion transport	Sapur.006G084800 Sapur.016G107800
	GO:0006351	32	1266	DNA-templated transcription	Sapur.001G009100 Sapur.002G021100 Sapur.002G030100 Sapur.002G036700 Sapur.002G140800 Sapur.004G118800 Sapur.005G141300 Sapur.007G050900 Sapur.007G102800 Sapur.008G069200 Sapur.008G077700 Sapur.008G134800 Sapur.010G005200 Sapur.010G065900 Sapur.010G085300 Sapur.010G120100 Sapur.010G135400 Sapur.011G042400 Sapur.012G067900 Sapur.012G076500 Sapur.013G050700 Sapur.014G014900 Sapur.014G056000 Sapur.016G202700 Sapur.016G261800 Sapur.017G053300 Sapur.017G071200 Sapur.018G057700 Sapur.15ZG067200 Sapur.15ZG132700 Sapur.T169500 Sapur.T175700
	GO:0032774	32	1268	RNA biosynthetic process	Sapur.001G009100 Sapur.002G021100 Sapur.002G030100 Sapur.002G036700 Sapur.002G140800 Sapur.004G118800 Sapur.005G141300 Sapur.007G050900 Sapur.007G102800 Sapur.008G069200 Sapur.008G077700 Sapur.008G134800 Sapur.010G005200 Sapur.010G065900 Sapur.010G085300 Sapur.010G120100 Sapur.010G135400 Sapur.011G042400 Sapur.012G067900 Sapur.012G076500 Sapur.013G050700 Sapur.014G014900 Sapur.014G056000 Sapur.016G202700 Sapur.016G261800 Sapur.017G053300 Sapur.017G071200 Sapur.018G057700 Sapur.15ZG067200 Sapur.15ZG132700 Sapur.T169500 Sapur.T175700
	GO:0019438	34	1389	aromatic compound biosynthetic process	Sapur.001G009100 Sapur.002G021100 Sapur.002G030100 Sapur.002G036700 Sapur.002G140800 Sapur.004G118800 Sapur.005G141300 Sapur.007G050900 Sapur.007G102800 Sapur.008G069200 Sapur.008G077700 Sapur.008G134800 Sapur.010G005200 Sapur.010G028300 Sapur.010G065900 Sapur.010G085300 Sapur.010G120100 Sapur.010G135400 Sapur.011G042400 Sapur.012G067900 Sapur.012G076500

				Sapur.013G050700 Sapur.014G014900 Sapur.014G056000 Sapur.016G202700 Sapur.016G261800 Sapur.017G053300 Sapur.017G071200 Sapur.018G057700 Sapur.15ZG067200 Sapur.15ZG110400 Sapur.15ZG132700 Sapur.T169500 Sapur.T175700
GO:0031326	30	1187	regulation of cellular biosynthetic process	Sapur.001G009100 Sapur.002G021100 Sapur.002G030100 Sapur.002G036700 Sapur.002G140800 Sapur.004G118800 Sapur.005G141300 Sapur.007G050900 Sapur.007G102800 Sapur.008G069200 Sapur.008G077700 Sapur.008G134800 Sapur.010G005200 Sapur.010G065900 Sapur.010G085300 Sapur.010G120100 Sapur.010G135400 Sapur.010G144500 Sapur.011G042400 Sapur.012G067900 Sapur.012G076500 Sapur.013G050700 Sapur.014G014900 Sapur.014G056000 Sapur.016G202700 Sapur.016G261800 Sapur.017G053300 Sapur.017G071200 Sapur.018G057700 Sapur.15ZG067200
GO:0010556	30	1187	regulation of macromolecule biosynthetic process	Sapur.001G009100 Sapur.002G021100 Sapur.002G030100 Sapur.002G036700 Sapur.002G140800 Sapur.004G118800 Sapur.005G141300 Sapur.007G050900 Sapur.007G102800 Sapur.008G069200 Sapur.008G077700 Sapur.008G134800 Sapur.010G005200 Sapur.010G065900 Sapur.010G085300 Sapur.010G120100 Sapur.010G135400 Sapur.010G144500 Sapur.011G042400 Sapur.012G067900 Sapur.012G076500 Sapur.013G050700 Sapur.014G014900 Sapur.014G056000 Sapur.016G202700 Sapur.016G261800 Sapur.017G053300 Sapur.017G071200 Sapur.018G057700 Sapur.15ZG067200
GO:0009889	30	1187	regulation of biosynthetic process	Sapur.001G009100 Sapur.002G021100 Sapur.002G030100 Sapur.002G036700 Sapur.002G140800 Sapur.004G118800 Sapur.005G141300 Sapur.007G050900 Sapur.007G102800 Sapur.008G069200 Sapur.008G077700 Sapur.008G134800 Sapur.010G005200 Sapur.010G065900 Sapur.010G085300 Sapur.010G120100 Sapur.010G135400 Sapur.010G144500 Sapur.011G042400 Sapur.012G067900 Sapur.012G076500 Sapur.013G050700 Sapur.014G014900 Sapur.014G056000 Sapur.016G202700 Sapur.016G261800 Sapur.017G053300 Sapur.017G071200 Sapur.018G057700 Sapur.15ZG067200
GO:0010468	30	1187	regulation of gene expression	Sapur.001G009100 Sapur.002G021100 Sapur.002G030100 Sapur.002G036700 Sapur.002G140800 Sapur.004G118800 Sapur.005G141300 Sapur.007G050900 Sapur.007G102800 Sapur.008G069200 Sapur.008G077700 Sapur.008G134800 Sapur.010G005200 Sapur.010G065900 Sapur.010G085300 Sapur.010G120100 Sapur.010G135400 Sapur.010G144500 Sapur.011G042400 Sapur.012G067900 Sapur.012G076500 Sapur.013G050700 Sapur.014G014900 Sapur.014G056000 Sapur.016G202700 Sapur.016G261800 Sapur.017G053300 Sapur.017G071200 Sapur.018G057700 Sapur.15ZG067200
GO:0051252	29	1141	regulation of RNA metabolic process	Sapur.001G009100 Sapur.002G021100 Sapur.002G030100 Sapur.002G036700 Sapur.002G140800 Sapur.004G118800 Sapur.005G141300 Sapur.007G050900 Sapur.007G102800 Sapur.008G069200 Sapur.008G077700 Sapur.008G134800 Sapur.010G005200 Sapur.010G065900 Sapur.010G085300 Sapur.010G120100 Sapur.010G135400 Sapur.011G042400 Sapur.012G067900 Sapur.012G076500 Sapur.013G050700 Sapur.014G014900 Sapur.014G056000 Sapur.016G202700 Sapur.016G261800 Sapur.017G053300 Sapur.017G071200 Sapur.018G057700 Sapur.15ZG067200
GO:2001141	29	1141	regulation of RNA biosynthetic process	Sapur.001G009100 Sapur.002G021100 Sapur.002G030100 Sapur.002G036700 Sapur.002G140800 Sapur.004G118800 Sapur.005G141300 Sapur.007G050900 Sapur.007G102800 Sapur.008G069200 Sapur.008G077700 Sapur.008G134800 Sapur.010G005200 Sapur.010G065900 Sapur.010G085300 Sapur.010G120100 Sapur.010G135400 Sapur.011G042400 Sapur.012G067900 Sapur.012G076500 Sapur.013G050700 Sapur.014G014900 Sapur.014G056000 Sapur.016G202700 Sapur.016G261800 Sapur.017G053300 Sapur.017G071200 Sapur.018G057700 Sapur.15ZG067200
GO:0006355	29	1141	regulation of DNA-templated transcription	Sapur.001G009100 Sapur.002G021100 Sapur.002G030100 Sapur.002G036700 Sapur.002G140800 Sapur.004G118800 Sapur.005G141300 Sapur.007G050900 Sapur.007G102800 Sapur.008G069200 Sapur.008G077700 Sapur.008G134800 Sapur.010G005200 Sapur.010G065900 Sapur.010G085300 Sapur.010G120100 Sapur.010G135400 Sapur.011G042400 Sapur.012G067900 Sapur.012G076500 Sapur.013G050700 Sapur.014G014900 Sapur.014G056000 Sapur.016G202700 Sapur.016G261800 Sapur.017G053300 Sapur.017G071200 Sapur.018G057700 Sapur.15ZG067200
GO:0034654	33	1354	nucleobase-containing compound	Sapur.001G009100 Sapur.002G021100 Sapur.002G030100 Sapur.002G036700 Sapur.002G140800 Sapur.004G118800 Sapur.005G141300 Sapur.007G050900 Sapur.007G102800 Sapur.008G069200 Sapur.008G077700 Sapur.008G134800 Sapur.010G005200 Sapur.010G028300

			biosynthetic process	Sapur.010G065900 Sapur.010G085300 Sapur.010G120100 Sapur.010G135400 Sapur.011G042400 Sapur.012G067900 Sapur.012G076500 Sapur.013G050700 Sapur.014G014900 Sapur.014G056000 Sapur.016G202700 Sapur.016G261800 Sapur.017G053300 Sapur.017G071200 Sapur.018G057700 Sapur.15ZG067200 Sapur.15ZG132700 Sapur.T169500 Sapur.T175700
GO:0019219	29	1148	regulation of nucleobase-containing compound metabolic process	Sapur.001G009100 Sapur.002G021100 Sapur.002G030100 Sapur.002G036700 Sapur.002G140800 Sapur.004G118800 Sapur.005G141300 Sapur.007G050900 Sapur.007G102800 Sapur.008G069200 Sapur.008G077700 Sapur.008G134800 Sapur.010G005200 Sapur.010G065900 Sapur.010G085300 Sapur.010G120100 Sapur.010G135400 Sapur.011G042400 Sapur.012G067900 Sapur.012G076500 Sapur.013G050700 Sapur.014G014900 Sapur.014G056000 Sapur.016G202700 Sapur.016G261800 Sapur.017G053300 Sapur.017G071200 Sapur.018G057700 Sapur.15ZG067200
GO:0018130	34	1411	heterocycle biosynthetic process	Sapur.001G009100 Sapur.002G021100 Sapur.002G030100 Sapur.002G036700 Sapur.002G140800 Sapur.004G118800 Sapur.005G141300 Sapur.007G050900 Sapur.007G102800 Sapur.008G069200 Sapur.008G077700 Sapur.008G134800 Sapur.010G005200 Sapur.010G028300 Sapur.010G065900 Sapur.010G085300 Sapur.010G120100 Sapur.010G135400 Sapur.011G042400 Sapur.012G067900 Sapur.012G076500 Sapur.013G050700 Sapur.014G014900 Sapur.014G056000 Sapur.016G202700 Sapur.016G261800 Sapur.017G053300 Sapur.017G071200 Sapur.018G057700 Sapur.15ZG067200 Sapur.15ZG110400 Sapur.15ZG132700 Sapur.T169500 Sapur.T175700
GO:0006952	4	53	defense response	Sapur.004G012300 Sapur.004G012400 Sapur.004G034700 Sapur.004G034800
GO:0031323	30	1210	regulation of cellular metabolic process	Sapur.001G009100 Sapur.002G021100 Sapur.002G030100 Sapur.002G036700 Sapur.002G140800 Sapur.004G118800 Sapur.005G141300 Sapur.007G050900 Sapur.007G102800 Sapur.008G069200 Sapur.008G077700 Sapur.008G134800 Sapur.010G005200 Sapur.010G065900 Sapur.010G085300 Sapur.010G120100 Sapur.010G135400 Sapur.010G144500 Sapur.011G042400 Sapur.012G067900 Sapur.012G076500 Sapur.013G050700 Sapur.014G014900 Sapur.014G056000 Sapur.016G202700 Sapur.016G261800 Sapur.017G053300 Sapur.017G071200 Sapur.018G057700 Sapur.15ZG067200
GO:0060255	30	1211	regulation of macromolecule metabolic process	Sapur.001G009100 Sapur.002G021100 Sapur.002G030100 Sapur.002G036700 Sapur.002G140800 Sapur.004G118800 Sapur.005G141300 Sapur.007G050900 Sapur.007G102800 Sapur.008G069200 Sapur.008G077700 Sapur.008G134800 Sapur.010G005200 Sapur.010G065900 Sapur.010G085300 Sapur.010G120100 Sapur.010G135400 Sapur.010G144500 Sapur.011G042400 Sapur.012G067900 Sapur.012G076500 Sapur.013G050700 Sapur.014G014900 Sapur.014G056000 Sapur.016G202700 Sapur.016G261800 Sapur.017G053300 Sapur.017G071200 Sapur.018G057700 Sapur.15ZG067200
GO:0019222	30	1214	regulation of metabolic process	Sapur.001G009100 Sapur.002G021100 Sapur.002G030100 Sapur.002G036700 Sapur.002G140800 Sapur.004G118800 Sapur.005G141300 Sapur.007G050900 Sapur.007G102800 Sapur.008G069200 Sapur.008G077700 Sapur.008G134800 Sapur.010G005200 Sapur.010G065900 Sapur.010G085300 Sapur.010G120100 Sapur.010G135400 Sapur.010G144500 Sapur.011G042400 Sapur.012G067900 Sapur.012G076500 Sapur.013G050700 Sapur.014G014900 Sapur.014G056000 Sapur.016G202700 Sapur.016G261800 Sapur.017G053300 Sapur.017G071200 Sapur.018G057700 Sapur.15ZG067200
GO:0080090	29	1172	regulation of primary metabolic process	Sapur.001G009100 Sapur.002G021100 Sapur.002G030100 Sapur.002G036700 Sapur.002G140800 Sapur.004G118800 Sapur.005G141300 Sapur.007G050900 Sapur.007G102800 Sapur.008G069200 Sapur.008G077700 Sapur.008G134800 Sapur.010G005200 Sapur.010G065900 Sapur.010G085300 Sapur.010G120100 Sapur.010G135400 Sapur.011G042400 Sapur.012G067900 Sapur.012G076500 Sapur.013G050700 Sapur.014G014900 Sapur.014G056000 Sapur.016G202700 Sapur.016G261800 Sapur.017G053300 Sapur.017G071200 Sapur.018G057700 Sapur.15ZG067200
GO:0051171	29	1172	regulation of nitrogen compound metabolic process	Sapur.001G009100 Sapur.002G021100 Sapur.002G030100 Sapur.002G036700 Sapur.002G140800 Sapur.004G118800 Sapur.005G141300 Sapur.007G050900 Sapur.007G102800 Sapur.008G069200 Sapur.008G077700 Sapur.008G134800 Sapur.010G005200 Sapur.010G065900 Sapur.010G085300 Sapur.010G120100 Sapur.010G135400 Sapur.011G042400 Sapur.012G067900 Sapur.012G076500 Sapur.013G050700 Sapur.014G014900 Sapur.014G056000 Sapur.016G202700 Sapur.016G261800 Sapur.017G053300 Sapur.017G071200 Sapur.018G057700

				Sapur.15ZG067200
GO:1901362	34	1454	organic cyclic compound biosynthetic process	Sapur.001G009100 Sapur.002G021100 Sapur.002G030100 Sapur.002G036700 Sapur.002G140800 Sapur.004G118800 Sapur.005G141300 Sapur.007G050900 Sapur.007G102800 Sapur.008G069200 Sapur.008G077700 Sapur.008G134800 Sapur.010G005200 Sapur.010G028300 Sapur.010G065900 Sapur.010G085300 Sapur.010G120100 Sapur.010G135400 Sapur.011G042400 Sapur.012G067900 Sapur.012G076500 Sapur.013G050700 Sapur.014G014900 Sapur.014G056000 Sapur.016G202700 Sapur.016G261800 Sapur.017G053300 Sapur.017G071200 Sapur.018G057700 Sapur.15ZG067200 Sapur.15ZG110400 Sapur.15ZG132700 Sapur.T169500 Sapur.T175700
GO:0048878	2	15	chemical homeostasis	Sapur.006G084800 Sapur.016G107800
GO:0055082	2	15	intracellular chemical homeostasis	Sapur.006G084800 Sapur.016G107800
GO:0009408	1	2	response to heat	Sapur.010G065900
GO:0015074	1	2	DNA integration	Sapur.014G138900
GO:0017062	1	2	respiratory chain complex III assembly	Sapur.006G138300
GO:0009266	1	2	response to temperature stimulus	Sapur.010G065900
GO:0034551	1	2	mitochondrial respiratory chain complex III assembly	Sapur.006G138300
GO:0006810	31	1398	transport	Sapur.001G008400 Sapur.001G034600 Sapur.001G036000 Sapur.001G053700 Sapur.001G120100 Sapur.004G051000 Sapur.005G131700 Sapur.006G014400 Sapur.006G023800 Sapur.006G084800 Sapur.006G174900 Sapur.008G065700 Sapur.008G101600 Sapur.010G040800 Sapur.010G194900 Sapur.012G000800 Sapur.012G004600 Sapur.012G020600 Sapur.012G035400 Sapur.014G090200 Sapur.014G119100 Sapur.016G089000 Sapur.016G107800 Sapur.017G057900 Sapur.017G063700 Sapur.019G009400 Sapur.15WG081100 Sapur.15ZG096600 Sapur.T148900 Sapur.002G055900 Sapur.009G028900
GO:0051234	31	1407	establishment of localization	Sapur.001G008400 Sapur.001G034600 Sapur.001G036000 Sapur.001G053700 Sapur.001G120100 Sapur.004G051000 Sapur.005G131700 Sapur.006G014400 Sapur.006G023800 Sapur.006G084800 Sapur.006G174900 Sapur.008G065700 Sapur.008G101600 Sapur.010G040800 Sapur.010G194900 Sapur.012G000800 Sapur.012G004600 Sapur.012G020600 Sapur.012G035400 Sapur.014G090200 Sapur.014G119100 Sapur.016G089000 Sapur.016G107800 Sapur.017G057900 Sapur.017G063700 Sapur.019G009400 Sapur.15WG081100 Sapur.15ZG096600 Sapur.T148900 Sapur.002G055900 Sapur.009G028900
GO:0030001	7	202	metal ion transport	Sapur.004G051000 Sapur.006G014400 Sapur.006G084800 Sapur.006G174900 Sapur.008G065700 Sapur.016G107800 Sapur.009G028900
GO:0051179	31	1416	localization	Sapur.001G008400 Sapur.001G034600 Sapur.001G036000 Sapur.001G053700 Sapur.001G120100 Sapur.004G051000 Sapur.005G131700 Sapur.006G014400 Sapur.006G023800 Sapur.006G084800 Sapur.006G174900 Sapur.008G065700 Sapur.008G101600 Sapur.010G040800 Sapur.010G194900 Sapur.012G000800 Sapur.012G004600 Sapur.012G020600 Sapur.012G035400 Sapur.014G090200 Sapur.014G119100 Sapur.016G089000 Sapur.016G107800 Sapur.017G057900 Sapur.017G063700 Sapur.019G009400 Sapur.15WG081100 Sapur.15ZG096600

					Sapur.T148900 Sapur.002G055900 Sapur.009G028900
	GO:0033108	1	3	mitochondrial respiratory chain complex assembly	Sapur.006G138300
	GO:0015630	67	170	microtubule cytoskeleton	Sapur.001G086500 Sapur.002G085200 Sapur.002G085300 Sapur.002G089300 Sapur.002G093600 Sapur.002G182100 Sapur.003G082400 Sapur.003G090100 Sapur.003G091100 Sapur.003G092100 Sapur.004G019100 Sapur.004G071100 Sapur.004G109700 Sapur.004G120900 Sapur.004G122400 Sapur.004G147000 Sapur.005G015200 Sapur.005G022700 Sapur.005G024100 Sapur.005G025300 Sapur.005G029900 Sapur.005G093300 Sapur.006G025400 Sapur.006G029800 Sapur.006G036800 Sapur.006G036900 Sapur.006G067400 Sapur.006G069500 Sapur.006G112500 Sapur.006G159000 Sapur.006G172300 Sapur.007G123700 Sapur.008G137200 Sapur.009G018700 Sapur.009G029500 Sapur.009G052200 Sapur.009G122200 Sapur.010G042700 Sapur.010G119100 Sapur.010G206600 Sapur.011G018500 Sapur.011G103700 Sapur.011G119600 Sapur.011G122100 Sapur.012G037400 Sapur.012G040700 Sapur.013G021800 Sapur.013G071200 Sapur.014G005100 Sapur.014G100600 Sapur.014G121100 Sapur.016G052600 Sapur.016G071200 Sapur.016G077400 Sapur.016G127100 Sapur.016G138000 Sapur.016G172500 Sapur.016G305500 Sapur.017G006400 Sapur.017G006500 Sapur.017G067300 Sapur.017G075700 Sapur.018G057600 Sapur.15WG044400 Sapur.15WG062900 Sapur.15ZG049500 Sapur.T003900
**r_down_CC**	GO:0005856	69	220	cytoskeleton	Sapur.001G086500 Sapur.002G085200 Sapur.002G085300 Sapur.002G089300 Sapur.002G093600 Sapur.002G182100 Sapur.003G082400 Sapur.003G090100 Sapur.003G091100 Sapur.003G092100 Sapur.004G019100 Sapur.004G071100 Sapur.004G109700 Sapur.004G120900 Sapur.004G122400 Sapur.004G147000 Sapur.005G015200 Sapur.005G022700 Sapur.005G024100 Sapur.005G025300 Sapur.005G029900 Sapur.005G093300 Sapur.006G025400 Sapur.006G029800 Sapur.006G036800 Sapur.006G036900 Sapur.006G067400 Sapur.006G069500 Sapur.006G112500 Sapur.006G159000 Sapur.006G172300 Sapur.007G123700 Sapur.008G137200 Sapur.009G018000 Sapur.009G018700 Sapur.009G029500 Sapur.009G052200 Sapur.009G122200 Sapur.010G042700 Sapur.010G119100 Sapur.010G206600 Sapur.011G018500 Sapur.011G103700 Sapur.011G119600 Sapur.011G122100 Sapur.012G037400 Sapur.012G040700 Sapur.013G021800 Sapur.013G071200 Sapur.014G005100 Sapur.014G100600 Sapur.014G121100 Sapur.016G052600 Sapur.016G071200 Sapur.016G077400 Sapur.016G127100 Sapur.016G138000 Sapur.016G172500 Sapur.016G305500 Sapur.017G006400 Sapur.017G006500 Sapur.017G067300 Sapur.017G075700 Sapur.018G057600 Sapur.15WG044400 Sapur.15WG062900 Sapur.15ZG049500 Sapur.15ZG061300 Sapur.T003900
	GO:0005871	49	103	kinesin complex	Sapur.002G085200 Sapur.002G085300 Sapur.002G089300 Sapur.002G093600 Sapur.002G182100 Sapur.003G092100 Sapur.004G019100 Sapur.004G109700 Sapur.004G120900 Sapur.004G122400 Sapur.004G147000 Sapur.005G015200 Sapur.005G022700 Sapur.005G024100 Sapur.005G025300 Sapur.005G029900 Sapur.005G093300 Sapur.006G029800 Sapur.006G036800 Sapur.006G036900 Sapur.006G067400 Sapur.006G069500 Sapur.006G112500 Sapur.006G159000 Sapur.006G172300 Sapur.008G137200 Sapur.009G018700 Sapur.009G122200 Sapur.010G042700 Sapur.010G119100 Sapur.011G018500 Sapur.011G103700 Sapur.011G122100 Sapur.012G037400 Sapur.012G040700 Sapur.013G021800 Sapur.013G071200 Sapur.014G005100 Sapur.014G100600 Sapur.014G121100 Sapur.016G052600 Sapur.016G071200 Sapur.016G077400 Sapur.016G127100 Sapur.016G305500 Sapur.018G057600 Sapur.15WG044400 Sapur.15ZG049500 Sapur.T003900
	GO:0005875	50	120	microtubule associated complex	Sapur.002G085200 Sapur.002G085300 Sapur.002G089300 Sapur.002G093600 Sapur.002G182100 Sapur.003G092100 Sapur.004G019100 Sapur.004G109700 Sapur.004G120900 Sapur.004G122400 Sapur.004G147000 Sapur.005G015200 Sapur.005G022700 Sapur.005G024100 Sapur.005G025300 Sapur.005G029900 Sapur.005G093300 Sapur.006G029800 Sapur.006G036800 Sapur.006G036900 Sapur.006G067400 Sapur.006G069500 Sapur.006G112500 Sapur.006G159000 Sapur.006G172300 Sapur.008G137200 Sapur.009G018700 Sapur.009G122200 Sapur.010G042700 Sapur.010G119100 Sapur.010G206600 Sapur.011G018500 Sapur.011G103700 Sapur.011G122100 Sapur.012G037400 Sapur.012G040700 Sapur.013G021800 Sapur.013G071200 Sapur.014G005100 Sapur.014G100600 Sapur.014G121100 Sapur.016G052600 Sapur.016G071200 Sapur.016G077400 Sapur.016G127100 Sapur.016G305500 Sapur.018G057600 Sapur.15WG044400 Sapur.15ZG049500 Sapur.T003900

GO:0043228	92	723	non-membrane-bounded organelle	Sapur.006G129500 Sapur.15ZG084800 Sapur.15ZG094200 Sapur.001G014000 Sapur.001G086500 Sapur.001G103000 Sapur.002G021500 Sapur.002G085200 Sapur.002G085300 Sapur.002G089300 Sapur.002G093600 Sapur.002G160400 Sapur.002G182100 Sapur.003G082400 Sapur.003G090100 Sapur.003G091100 Sapur.003G092100 Sapur.003G159300 Sapur.003G159400 Sapur.004G019100 Sapur.004G060500 Sapur.004G071100 Sapur.004G109700 Sapur.004G120900 Sapur.004G122400 Sapur.004G147000 Sapur.004G163000 Sapur.005G015200 Sapur.005G022700 Sapur.005G024100 Sapur.005G025300 Sapur.005G029900 Sapur.005G093300 Sapur.005G186700 Sapur.006G025400 Sapur.006G029800 Sapur.006G036800 Sapur.006G036900 Sapur.006G067400 Sapur.006G069500 Sapur.006G112500 Sapur.006G159000 Sapur.006G172300 Sapur.007G123700 Sapur.008G021500 Sapur.008G137200 Sapur.009G003100 Sapur.009G018000 Sapur.009G018700 Sapur.009G029500 Sapur.009G052200 Sapur.009G122200 Sapur.010G042700 Sapur.010G082600 Sapur.010G119100 Sapur.010G184700 Sapur.010G185200 Sapur.010G206600 Sapur.011G018500 Sapur.011G056100 Sapur.011G103700 Sapur.011G119600 Sapur.011G122100 Sapur.012G037400 Sapur.012G040700 Sapur.013G021800 Sapur.013G071200 Sapur.014G005100 Sapur.014G075000 Sapur.014G075400 Sapur.014G100600 Sapur.014G121100 Sapur.014G127000 Sapur.016G052600 Sapur.016G071200 Sapur.016G077400 Sapur.016G121300 Sapur.016G127100 Sapur.016G138000 Sapur.016G172500 Sapur.016G305500 Sapur.017G006400 Sapur.017G006500 Sapur.017G067300 Sapur.017G075700 Sapur.018G057600 Sapur.15WG044400 Sapur.15WG062900 Sapur.15ZG049500 Sapur.15ZG061300 Sapur.T003900 Sapur.014G066300
GO:0043232	92	723	intracellular non-membrane-bounded organelle	Sapur.006G129500 Sapur.15ZG084800 Sapur.15ZG094200 Sapur.001G014000 Sapur.001G086500 Sapur.001G103000 Sapur.002G021500 Sapur.002G085200 Sapur.002G085300 Sapur.002G089300 Sapur.002G093600 Sapur.002G160400 Sapur.002G182100 Sapur.003G082400 Sapur.003G090100 Sapur.003G091100 Sapur.003G092100 Sapur.003G159300 Sapur.003G159400 Sapur.004G019100 Sapur.004G060500 Sapur.004G071100 Sapur.004G109700 Sapur.004G120900 Sapur.004G122400 Sapur.004G147000 Sapur.004G163000 Sapur.005G015200 Sapur.005G022700 Sapur.005G024100 Sapur.005G025300 Sapur.005G029900 Sapur.005G093300 Sapur.005G186700 Sapur.006G025400 Sapur.006G029800 Sapur.006G036800 Sapur.006G036900 Sapur.006G067400 Sapur.006G069500 Sapur.006G112500 Sapur.006G159000 Sapur.006G172300 Sapur.007G123700 Sapur.008G021500 Sapur.008G137200 Sapur.009G003100 Sapur.009G018000 Sapur.009G018700 Sapur.009G029500 Sapur.009G052200 Sapur.009G122200 Sapur.010G042700 Sapur.010G082600 Sapur.010G119100 Sapur.010G184700 Sapur.010G185200 Sapur.010G206600 Sapur.011G018500 Sapur.011G056100 Sapur.011G103700 Sapur.011G119600 Sapur.011G122100 Sapur.012G037400 Sapur.012G040700 Sapur.013G021800 Sapur.013G071200 Sapur.014G005100 Sapur.014G075000 Sapur.014G075400 Sapur.014G100600 Sapur.014G121100 Sapur.014G127000 Sapur.016G052600 Sapur.016G071200 Sapur.016G077400 Sapur.016G121300 Sapur.016G127100 Sapur.016G138000 Sapur.016G172500 Sapur.016G305500 Sapur.017G006400 Sapur.017G006500 Sapur.017G067300 Sapur.017G075700 Sapur.018G057600 Sapur.15WG044400 Sapur.15WG062900 Sapur.15ZG049500 Sapur.15ZG061300 Sapur.T003900 Sapur.014G066300
GO:0099081	18	44	supramolecular polymer	Sapur.001G086500 Sapur.003G082400 Sapur.003G090100 Sapur.003G091100 Sapur.004G071100 Sapur.006G025400 Sapur.007G123700 Sapur.009G029500 Sapur.009G052200 Sapur.011G119600 Sapur.016G138000 Sapur.016G172500 Sapur.017G006400 Sapur.017G006500 Sapur.017G067300 Sapur.017G075700 Sapur.15WG062900 Sapur.15ZG061300
GO:0099512	18	44	supramolecular fiber	Sapur.001G086500 Sapur.003G082400 Sapur.003G090100 Sapur.003G091100 Sapur.004G071100 Sapur.006G025400 Sapur.007G123700 Sapur.009G029500 Sapur.009G052200 Sapur.011G119600 Sapur.016G138000 Sapur.016G172500 Sapur.017G006400 Sapur.017G006500 Sapur.017G067300 Sapur.017G075700 Sapur.15WG062900 Sapur.15ZG061300
GO:0099513	18	44	polymeric cytoskeletal fiber	Sapur.001G086500 Sapur.003G082400 Sapur.003G090100 Sapur.003G091100 Sapur.004G071100 Sapur.006G025400 Sapur.007G123700 Sapur.009G029500 Sapur.009G052200 Sapur.011G119600 Sapur.016G138000 Sapur.016G172500 Sapur.017G006400 Sapur.017G006500 Sapur.017G067300 Sapur.017G075700 Sapur.15WG062900 Sapur.15ZG061300
GO:0099080	18	46	supramolecular complex	Sapur.001G086500 Sapur.003G082400 Sapur.003G090100 Sapur.003G091100 Sapur.004G071100 Sapur.006G025400 Sapur.007G123700 Sapur.009G029500 Sapur.009G052200 Sapur.011G119600 Sapur.016G138000 Sapur.016G172500 Sapur.017G006400 Sapur.017G006500

				Sapur.017G067300 Sapur.017G075700 Sapur.15WG062900 Sapur.15ZG061300
GO:0005874	17	42	microtubule	Sapur.001G086500 Sapur.003G082400 Sapur.003G090100 Sapur.003G091100 Sapur.004G071100 Sapur.006G025400 Sapur.007G123700 Sapur.009G029500 Sapur.009G052200 Sapur.011G119600 Sapur.016G138000 Sapur.016G172500 Sapur.017G006400 Sapur.017G006500 Sapur.017G067300 Sapur.017G075700 Sapur.15WG062900
GO:0043226	141	1812	organelle	Sapur.001G055600 Sapur.001G058800 Sapur.001G156400 Sapur.002G006500 Sapur.002G018600 Sapur.002G063700 Sapur.002G099000 Sapur.002G101800 Sapur.002G160400 Sapur.003G126500 Sapur.004G139900 Sapur.004G150900 Sapur.004G163000 Sapur.005G079500 Sapur.005G094500 Sapur.005G142500 Sapur.005G189800 Sapur.005G201400 Sapur.006G025200 Sapur.006G037000 Sapur.006G204600 Sapur.007G005400 Sapur.007G051100 Sapur.007G123900 Sapur.008G077200 Sapur.009G003100 Sapur.009G067900 Sapur.009G072700 Sapur.009G076400 Sapur.009G107100 Sapur.009G126200 Sapur.009G129900 Sapur.010G046500 Sapur.010G068800 Sapur.010G075900 Sapur.010G082600 Sapur.010G193700 Sapur.013G044800 Sapur.014G003800 Sapur.014G013200 Sapur.014G016400 Sapur.014G066300 Sapur.014G096700 Sapur.014G099300 Sapur.016G029900 Sapur.016G157600 Sapur.016G174200 Sapur.016G181200 Sapur.018G047100 Sapur.15WG072800 Sapur.15ZG043500 Sapur.006G129500 Sapur.15ZG084800 Sapur.15ZG094200 Sapur.T189500 Sapur.001G014000 Sapur.001G086500 Sapur.001G103000 Sapur.002G021500 Sapur.002G085200 Sapur.002G085300 Sapur.002G089300 Sapur.002G093600 Sapur.002G182100 Sapur.003G082400 Sapur.003G090100 Sapur.003G091100 Sapur.003G092100 Sapur.003G159300 Sapur.003G159400 Sapur.004G019100 Sapur.004G060500 Sapur.004G071100 Sapur.004G109700 Sapur.004G120900 Sapur.004G122400 Sapur.004G147000 Sapur.005G015200 Sapur.005G022700 Sapur.005G024100 Sapur.005G025300 Sapur.005G029900 Sapur.005G093300 Sapur.005G186700 Sapur.006G025400 Sapur.006G029800 Sapur.006G036800 Sapur.006G036900 Sapur.006G067400 Sapur.006G069500 Sapur.006G112500 Sapur.006G159000 Sapur.006G172300 Sapur.007G123700 Sapur.008G021500 Sapur.008G137200 Sapur.009G018000 Sapur.009G018700 Sapur.009G029500 Sapur.009G052200 Sapur.009G122200 Sapur.010G042700 Sapur.010G119100 Sapur.010G184700 Sapur.010G185200 Sapur.010G206600 Sapur.011G018500 Sapur.011G056100 Sapur.011G103700 Sapur.011G119600 Sapur.011G122100 Sapur.012G037400 Sapur.012G040700 Sapur.013G021800 Sapur.013G054700 Sapur.013G071200 Sapur.013G131800 Sapur.014G005100 Sapur.014G075000 Sapur.014G075400 Sapur.014G100600 Sapur.014G121100 Sapur.014G127000 Sapur.016G052600 Sapur.016G071200 Sapur.016G077400 Sapur.016G121300 Sapur.016G127100 Sapur.016G138000 Sapur.016G172500 Sapur.016G305500 Sapur.017G006400 Sapur.017G006500 Sapur.017G067300 Sapur.017G075700 Sapur.018G057600 Sapur.15WG044400 Sapur.15WG062900 Sapur.15ZG049500 Sapur.15ZG061300 Sapur.T003900
GO:0043229	141	1812	intracellular organelle	Sapur.001G055600 Sapur.001G058800 Sapur.001G156400 Sapur.002G006500 Sapur.002G018600 Sapur.002G063700 Sapur.002G099000 Sapur.002G101800 Sapur.002G160400 Sapur.003G126500 Sapur.004G139900 Sapur.004G150900 Sapur.004G163000 Sapur.005G079500 Sapur.005G094500 Sapur.005G142500 Sapur.005G189800 Sapur.005G201400 Sapur.006G025200 Sapur.006G037000 Sapur.006G204600 Sapur.007G005400 Sapur.007G051100 Sapur.007G123900 Sapur.008G077200 Sapur.009G003100 Sapur.009G067900 Sapur.009G072700 Sapur.009G076400 Sapur.009G107100 Sapur.009G126200 Sapur.009G129900 Sapur.010G046500 Sapur.010G068800 Sapur.010G075900 Sapur.010G082600 Sapur.010G193700 Sapur.013G044800 Sapur.014G003800 Sapur.014G013200 Sapur.014G016400 Sapur.014G066300 Sapur.014G096700 Sapur.014G099300 Sapur.016G029900 Sapur.016G157600 Sapur.016G174200 Sapur.016G181200 Sapur.018G047100 Sapur.15WG072800 Sapur.15ZG043500 Sapur.006G129500 Sapur.15ZG084800 Sapur.15ZG094200 Sapur.T189500 Sapur.001G014000 Sapur.001G086500 Sapur.001G103000 Sapur.002G021500 Sapur.002G085200 Sapur.002G085300 Sapur.002G089300 Sapur.002G093600 Sapur.002G182100 Sapur.003G082400 Sapur.003G090100 Sapur.003G091100 Sapur.003G092100 Sapur.003G159300 Sapur.003G159400 Sapur.004G019100 Sapur.004G060500 Sapur.004G071100 Sapur.004G109700 Sapur.004G120900 Sapur.004G122400 Sapur.004G147000 Sapur.005G015200 Sapur.005G022700 Sapur.005G024100 Sapur.005G025300 Sapur.005G029900 Sapur.005G093300 Sapur.005G186700 Sapur.006G025400 Sapur.006G029800 Sapur.006G036800 Sapur.006G036900 Sapur.006G067400 Sapur.006G069500 Sapur.006G112500 Sapur.006G159000 Sapur.006G172300 Sapur.007G123700 Sapur.008G021500 Sapur.008G137200 Sapur.009G018000 Sapur.009G018700

				Sapur.009G029500 Sapur.009G052200 Sapur.009G122200 Sapur.010G042700 Sapur.010G119100 Sapur.010G184700 Sapur.010G185200 Sapur.010G206600 Sapur.011G018500 Sapur.011G056100 Sapur.011G103700 Sapur.011G119600 Sapur.011G122100 Sapur.012G037400 Sapur.012G040700 Sapur.013G021800 Sapur.013G054700 Sapur.013G071200 Sapur.013G131800 Sapur.014G005100 Sapur.014G075000 Sapur.014G075400 Sapur.014G100600 Sapur.014G121100 Sapur.014G127000 Sapur.016G052600 Sapur.016G071200 Sapur.016G077400 Sapur.016G121300 Sapur.016G127100 Sapur.016G138000 Sapur.016G172500 Sapur.016G305500 Sapur.017G006400 Sapur.017G006500 Sapur.017G067300 Sapur.017G075700 Sapur.018G057600 Sapur.15WG044400 Sapur.15WG062900 Sapur.15ZG049500 Sapur.15ZG061300 Sapur.T003900
GO:0032991	79	842	protein-containing complex	Sapur.001G014000 Sapur.001G055600 Sapur.001G058800 Sapur.001G103000 Sapur.002G012200 Sapur.002G021500 Sapur.002G085200 Sapur.002G085300 Sapur.002G089300 Sapur.002G093600 Sapur.002G160400 Sapur.002G182100 Sapur.003G092100 Sapur.003G159300 Sapur.003G159400 Sapur.004G019100 Sapur.004G060500 Sapur.004G109700 Sapur.004G120900 Sapur.004G122400 Sapur.004G147000 Sapur.005G015200 Sapur.005G022700 Sapur.005G024100 Sapur.005G025300 Sapur.005G029900 Sapur.005G093300 Sapur.005G186700 Sapur.006G029800 Sapur.006G036800 Sapur.006G036900 Sapur.006G067400 Sapur.006G069500 Sapur.006G112500 Sapur.006G159000 Sapur.006G172300 Sapur.008G021500 Sapur.008G137200 Sapur.009G018000 Sapur.009G018700 Sapur.009G122200 Sapur.010G042700 Sapur.010G082600 Sapur.010G119100 Sapur.010G184700 Sapur.010G185200 Sapur.010G206600 Sapur.011G018500 Sapur.011G056100 Sapur.011G103700 Sapur.011G122100 Sapur.012G037400 Sapur.012G040700 Sapur.012G054600 Sapur.013G021800 Sapur.013G054700 Sapur.013G071200 Sapur.014G005100 Sapur.014G075000 Sapur.014G075400 Sapur.014G096700 Sapur.014G100600 Sapur.014G121100 Sapur.014G127000 Sapur.016G052600 Sapur.016G071200 Sapur.016G077400 Sapur.016G127100 Sapur.016G128000 Sapur.016G305500 Sapur.018G057600 Sapur.15WG044400 Sapur.15ZG049500 Sapur.15ZG071900 Sapur.T003900 Sapur.014G066300 Sapur.15ZG029900 Sapur.016G040000 Sapur.15ZG094200
GO:0000786	12	47	nucleosome	Sapur.001G014000 Sapur.002G021500 Sapur.002G160400 Sapur.003G159300 Sapur.003G159400 Sapur.005G186700 Sapur.008G021500 Sapur.010G082600 Sapur.010G184700 Sapur.010G185200 Sapur.014G075000 Sapur.014G075400
GO:0005694	20	129	chromosome	Sapur.001G014000 Sapur.001G103000 Sapur.002G021500 Sapur.002G160400 Sapur.003G159300 Sapur.003G159400 Sapur.004G060500 Sapur.004G163000 Sapur.005G186700 Sapur.008G021500 Sapur.009G003100 Sapur.010G082600 Sapur.010G184700 Sapur.010G185200 Sapur.011G056100 Sapur.014G075000 Sapur.014G075400 Sapur.014G127000 Sapur.016G121300 Sapur.014G066300
GO:0005819	7	19	spindle	Sapur.003G082400 Sapur.004G071100 Sapur.007G123700 Sapur.017G006400 Sapur.017G006500 Sapur.017G075700 Sapur.15WG062900
GO:0000796	3	3	condensin complex	Sapur.001G103000 Sapur.004G060500 Sapur.014G127000
GO:0042555	3	3	MCM complex	Sapur.001G055600 Sapur.001G058800 Sapur.014G096700
GO:0005622	150	2201	intracellular anatomical structure	Sapur.001G055600 Sapur.001G058800 Sapur.001G156400 Sapur.002G006500 Sapur.002G018600 Sapur.002G063700 Sapur.002G099000 Sapur.002G101800 Sapur.002G160400 Sapur.003G126500 Sapur.004G139900 Sapur.004G150900 Sapur.004G163000 Sapur.005G079500 Sapur.005G094500 Sapur.005G142500 Sapur.005G189800 Sapur.005G201400 Sapur.006G025200 Sapur.006G037000 Sapur.006G204600 Sapur.007G005400 Sapur.007G051100 Sapur.007G123900 Sapur.008G077200 Sapur.009G003100 Sapur.009G067900 Sapur.009G072700 Sapur.009G076400 Sapur.009G107100 Sapur.009G126200 Sapur.009G129900 Sapur.010G046500 Sapur.010G068800 Sapur.010G075900 Sapur.010G082600 Sapur.010G193700 Sapur.013G044800 Sapur.014G003800 Sapur.014G013200 Sapur.014G016400 Sapur.014G066300 Sapur.014G096700 Sapur.014G099300 Sapur.016G029900 Sapur.016G157600 Sapur.016G174200 Sapur.016G181200 Sapur.018G047100 Sapur.15WG072800 Sapur.15ZG043500 Sapur.006G008400 Sapur.006G129500 Sapur.008G007500 Sapur.008G105600 Sapur.016G011100 Sapur.018G050900 Sapur.15ZG084800 Sapur.15ZG094200 Sapur.T189500 Sapur.001G014000 Sapur.001G086500 Sapur.001G103000

					Sapur.002G021500 Sapur.002G085200 Sapur.002G085300 Sapur.002G089300 Sapur.002G093600 Sapur.002G182100 Sapur.003G082400 Sapur.003G090100 Sapur.003G091100 Sapur.003G092100 Sapur.003G159300 Sapur.003G159400 Sapur.004G019100 Sapur.004G060500 Sapur.004G071100 Sapur.004G109700 Sapur.004G120900 Sapur.004G122400 Sapur.004G147000 Sapur.005G015200 Sapur.005G022700 Sapur.005G024100 Sapur.005G025300 Sapur.005G029900 Sapur.005G093300 Sapur.005G186700 Sapur.006G025400 Sapur.006G029800 Sapur.006G036800 Sapur.006G036900 Sapur.006G067400 Sapur.006G069500 Sapur.006G112500 Sapur.006G159000 Sapur.006G172300 Sapur.006G221200 Sapur.007G123700 Sapur.008G021500 Sapur.008G137200 Sapur.009G018000 Sapur.009G018700 Sapur.009G029500 Sapur.009G052200 Sapur.009G122200 Sapur.010G042700 Sapur.010G119100 Sapur.010G184700 Sapur.010G185200 Sapur.010G206600 Sapur.011G018500 Sapur.011G056100 Sapur.011G103700 Sapur.011G119600 Sapur.011G122100 Sapur.012G037400 Sapur.012G040700 Sapur.013G021800 Sapur.013G054700 Sapur.013G071200 Sapur.013G131800 Sapur.014G005100 Sapur.014G075000 Sapur.014G075400 Sapur.014G100600 Sapur.014G121100 Sapur.014G127000 Sapur.016G052600 Sapur.016G071200 Sapur.016G077400 Sapur.016G121300 Sapur.016G127100 Sapur.016G128000 Sapur.016G138000 Sapur.016G172500 Sapur.016G305500 Sapur.017G006400 Sapur.017G006500 Sapur.017G067300 Sapur.017G075700 Sapur.018G057600 Sapur.15WG044400 Sapur.15WG062900 Sapur.15ZG049500 Sapur.15ZG061300 Sapur.T003900 Sapur.15ZG029900 Sapur.016G040000
	GO:0000785	12	83	chromatin	Sapur.001G014000 Sapur.002G021500 Sapur.002G160400 Sapur.003G159300 Sapur.003G159400 Sapur.005G186700 Sapur.008G021500 Sapur.010G082600 Sapur.010G184700 Sapur.010G185200 Sapur.014G075000 Sapur.014G075400
	GO:0032993	12	83	protein-DNA complex	Sapur.001G014000 Sapur.002G021500 Sapur.002G160400 Sapur.003G159300 Sapur.003G159400 Sapur.005G186700 Sapur.008G021500 Sapur.010G082600 Sapur.010G184700 Sapur.010G185200 Sapur.014G075000 Sapur.014G075400
	GO:0000808	2	4	origin recognition complex	Sapur.011G056100 Sapur.014G066300
**r_up_CC**	GO:0016020	21	1182	membrane	Sapur.005G131700 Sapur.001G034600 Sapur.001G036000 Sapur.001G053700 Sapur.001G120100 Sapur.002G053300 Sapur.003G148600 Sapur.005G137600 Sapur.005G207200 Sapur.006G023600 Sapur.006G023800 Sapur.008G065700 Sapur.008G101600 Sapur.010G194900 Sapur.013G110500 Sapur.013G142900 Sapur.016G089000 Sapur.016G141300 Sapur.017G063700 Sapur.15ZG096600 Sapur.T148900
**r_down_MF**	GO:0008017	55	123	microtubule binding	Sapur.002G085200 Sapur.002G089300 Sapur.002G093600 Sapur.002G182100 Sapur.003G092100 Sapur.003G148500 Sapur.004G019100 Sapur.004G109700 Sapur.004G120900 Sapur.004G122400 Sapur.004G147000 Sapur.005G015200 Sapur.005G022700 Sapur.005G024100 Sapur.005G025300 Sapur.005G029900 Sapur.005G093300 Sapur.006G028600 Sapur.006G029800 Sapur.006G036800 Sapur.006G036900 Sapur.006G067400 Sapur.006G069500 Sapur.006G112500 Sapur.006G159000 Sapur.006G172300 Sapur.007G045800 Sapur.008G137200 Sapur.009G018700 Sapur.009G122200 Sapur.010G042700 Sapur.010G206600 Sapur.011G018500 Sapur.011G103700 Sapur.011G122100 Sapur.012G037400 Sapur.012G040700 Sapur.012G099300 Sapur.013G021800 Sapur.013G071200 Sapur.014G005100 Sapur.014G051500 Sapur.014G100600 Sapur.014G121100 Sapur.016G051800 Sapur.016G052600 Sapur.016G071200 Sapur.016G077400 Sapur.016G127100 Sapur.016G305500 Sapur.018G057600 Sapur.15WG044400 Sapur.15ZG049500 Sapur.15ZG122400 Sapur.T003900
	GO:0015631	55	124	tubulin binding	Sapur.002G085200 Sapur.002G089300 Sapur.002G093600 Sapur.002G182100 Sapur.003G092100 Sapur.003G148500 Sapur.004G019100 Sapur.004G109700 Sapur.004G120900 Sapur.004G122400 Sapur.004G147000 Sapur.005G015200 Sapur.005G022700 Sapur.005G024100 Sapur.005G025300 Sapur.005G029900 Sapur.005G093300 Sapur.006G028600 Sapur.006G029800 Sapur.006G036800 Sapur.006G036900 Sapur.006G067400 Sapur.006G069500 Sapur.006G112500 Sapur.006G159000 Sapur.006G172300 Sapur.007G045800 Sapur.008G137200 Sapur.009G018700 Sapur.009G122200 Sapur.010G042700 Sapur.010G206600 Sapur.011G018500 Sapur.011G103700 Sapur.011G122100 Sapur.012G037400 Sapur.012G040700 Sapur.012G099300 Sapur.013G021800 Sapur.013G071200 Sapur.014G005100 Sapur.014G051500 Sapur.014G100600 Sapur.014G121100 Sapur.016G051800 Sapur.016G052600 Sapur.016G071200 Sapur.016G077400 Sapur.016G127100 Sapur.016G305500 Sapur.018G057600 Sapur.15WG044400 Sapur.15ZG049500 Sapur.15ZG122400 Sapur.T003900
	GO:0003	50	106	microtubule motor	Sapur.002G085200 Sapur.002G085300 Sapur.002G089300 Sapur.002G093600 Sapur.002G182100 Sapur.003G092100 Sapur.004G019100

777			activity	Sapur.004G109700 Sapur.004G120900 Sapur.004G122400 Sapur.004G147000 Sapur.005G015200 Sapur.005G022700 Sapur.005G024100 Sapur.005G025300 Sapur.005G029900 Sapur.005G093300 Sapur.006G029800 Sapur.006G036800 Sapur.006G036900 Sapur.006G067400 Sapur.006G069500 Sapur.006G112500 Sapur.006G159000 Sapur.006G172300 Sapur.008G137200 Sapur.009G018700 Sapur.009G122200 Sapur.010G042700 Sapur.010G119100 Sapur.011G018500 Sapur.011G103700 Sapur.011G122100 Sapur.012G037400 Sapur.012G040700 Sapur.013G021800 Sapur.013G071200 Sapur.014G005100 Sapur.014G100600 Sapur.014G121100 Sapur.016G051800 Sapur.016G052600 Sapur.016G071200 Sapur.016G077400 Sapur.016G127100 Sapur.016G305500 Sapur.018G057600 Sapur.15WG044400 Sapur.15ZG049500 Sapur.T003900
GO:0003774	51	121	cytoskeletal motor activity	Sapur.002G085200 Sapur.002G085300 Sapur.002G089300 Sapur.002G093600 Sapur.002G182100 Sapur.003G092100 Sapur.004G019100 Sapur.004G109700 Sapur.004G120900 Sapur.004G122400 Sapur.004G147000 Sapur.005G015200 Sapur.005G022700 Sapur.005G024100 Sapur.005G025300 Sapur.005G029900 Sapur.005G093300 Sapur.006G029800 Sapur.006G036800 Sapur.006G036900 Sapur.006G067400 Sapur.006G069500 Sapur.006G112500 Sapur.006G159000 Sapur.006G172300 Sapur.008G137200 Sapur.009G018700 Sapur.009G122200 Sapur.010G042700 Sapur.010G119100 Sapur.011G018500 Sapur.011G103700 Sapur.011G122100 Sapur.012G037400 Sapur.012G040700 Sapur.013G021800 Sapur.013G071200 Sapur.014G005100 Sapur.014G100600 Sapur.014G121100 Sapur.016G051800 Sapur.016G052600 Sapur.016G071200 Sapur.016G077400 Sapur.016G127100 Sapur.016G305500 Sapur.018G057600 Sapur.15WG044400 Sapur.15ZG049500 Sapur.T003900 Sapur.009G018000
GO:0008092	56	163	cytoskeletal protein binding	Sapur.002G085200 Sapur.002G089300 Sapur.002G093600 Sapur.002G182100 Sapur.003G092100 Sapur.003G148500 Sapur.004G019100 Sapur.004G109700 Sapur.004G120900 Sapur.004G122400 Sapur.004G147000 Sapur.005G015200 Sapur.005G022700 Sapur.005G024100 Sapur.005G025300 Sapur.005G029900 Sapur.005G093300 Sapur.006G028600 Sapur.006G029800 Sapur.006G036800 Sapur.006G036900 Sapur.006G067400 Sapur.006G069500 Sapur.006G112500 Sapur.006G159000 Sapur.006G172300 Sapur.007G045800 Sapur.008G137200 Sapur.009G018700 Sapur.009G033900 Sapur.009G122200 Sapur.010G042700 Sapur.010G206600 Sapur.011G018500 Sapur.011G103700 Sapur.011G122100 Sapur.012G037400 Sapur.012G040700 Sapur.012G099300 Sapur.013G021800 Sapur.013G071200 Sapur.014G005100 Sapur.014G051500 Sapur.014G100600 Sapur.014G121100 Sapur.016G051800 Sapur.016G052600 Sapur.016G071200 Sapur.016G077400 Sapur.016G127100 Sapur.016G305500 Sapur.018G057600 Sapur.15WG044400 Sapur.15ZG049500 Sapur.15ZG122400 Sapur.T003900
GO:0005524	166	1823	ATP binding	Sapur.001G040400 Sapur.001G055600 Sapur.001G058800 Sapur.001G078000 Sapur.001G103000 Sapur.001G103900 Sapur.001G126100 Sapur.002G002000 Sapur.002G005900 Sapur.002G020300 Sapur.002G051400 Sapur.002G085200 Sapur.002G089300 Sapur.002G093600 Sapur.002G106900 Sapur.002G120400 Sapur.002G171500 Sapur.002G182100 Sapur.002G188100 Sapur.003G020100 Sapur.003G051100 Sapur.003G061800 Sapur.003G073200 Sapur.003G079800 Sapur.003G092100 Sapur.003G099500 Sapur.003G099700 Sapur.003G139700 Sapur.004G019100 Sapur.004G029200 Sapur.004G032900 Sapur.004G051400 Sapur.004G059100 Sapur.004G067800 Sapur.004G089200 Sapur.004G103000 Sapur.004G109700 Sapur.004G120900 Sapur.004G122400 Sapur.004G147000 Sapur.004G170800 Sapur.005G001800 Sapur.005G013900 Sapur.005G015200 Sapur.005G022700 Sapur.005G024100 Sapur.005G025300 Sapur.005G027300 Sapur.005G029900 Sapur.005G050800 Sapur.005G093300 Sapur.005G109200 Sapur.005G206400 Sapur.006G029800 Sapur.006G036800 Sapur.006G036900 Sapur.006G067400 Sapur.006G069500 Sapur.006G096300 Sapur.006G101400 Sapur.006G107000 Sapur.006G111600 Sapur.006G112500 Sapur.006G159000 Sapur.006G172300 Sapur.006G188300 Sapur.006G194600 Sapur.006G195000 Sapur.007G005400 Sapur.007G012300 Sapur.007G016800 Sapur.007G024100 Sapur.007G091900 Sapur.007G127200 Sapur.008G005500 Sapur.008G016100 Sapur.008G113700 Sapur.008G128300 Sapur.008G137200 Sapur.008G143200 Sapur.008G157700 Sapur.009G018000 Sapur.009G018700 Sapur.009G038600 Sapur.009G045200 Sapur.009G066000 Sapur.009G096500 Sapur.009G122200 Sapur.010G041500 Sapur.010G042700 Sapur.010G053300 Sapur.010G057900 Sapur.010G076000 Sapur.010G103300 Sapur.010G189000 Sapur.011G002200 Sapur.011G002300 Sapur.011G018500 Sapur.011G047800 Sapur.011G072400 Sapur.011G080600 Sapur.011G103500 Sapur.011G103700 Sapur.011G122100 Sapur.012G020400 Sapur.012G029700 Sapur.012G037400 Sapur.012G040700 Sapur.012G067300 Sapur.012G085700 Sapur.012G098500 Sapur.013G003400

				Sapur.013G003500 Sapur.013G021800 Sapur.013G071200 Sapur.013G141900 Sapur.014G003800 Sapur.014G005100 Sapur.014G096700 Sapur.014G100600 Sapur.014G121100 Sapur.014G126200 Sapur.016G045300 Sapur.016G046200 Sapur.016G051800 Sapur.016G052600 Sapur.016G071200 Sapur.016G077400 Sapur.016G087900 Sapur.016G127100 Sapur.016G152100 Sapur.016G191700 Sapur.016G205200 Sapur.016G220800 Sapur.016G233300 Sapur.016G294500 Sapur.016G305500 Sapur.017G021900 Sapur.017G107900 Sapur.018G023200 Sapur.018G035500 Sapur.018G047100 Sapur.018G053200 Sapur.018G057600 Sapur.018G087600 Sapur.019G008900 Sapur.019G024300 Sapur.019G028700 Sapur.019G028900 Sapur.019G048400 Sapur.019G058700 Sapur.019G075600 Sapur.019G107300 Sapur.019G113000 Sapur.15WG028200 Sapur.15WG044400 Sapur.15ZG005000 Sapur.15ZG013600 Sapur.15ZG018000 Sapur.15ZG027900 Sapur.15ZG049500 Sapur.15ZG121300 Sapur.T003900 Sapur.T124800 Sapur.T138000 Sapur.T191300
GO:0140657	58	460	ATP-dependent activity	Sapur.003G099500 Sapur.009G038600 Sapur.016G087900 Sapur.001G055600 Sapur.001G058800 Sapur.002G085200 Sapur.002G085300 Sapur.002G089300 Sapur.002G093600 Sapur.002G182100 Sapur.003G092100 Sapur.004G019100 Sapur.004G109700 Sapur.004G120900 Sapur.004G122400 Sapur.004G147000 Sapur.005G015200 Sapur.005G022700 Sapur.005G024100 Sapur.005G025300 Sapur.005G029900 Sapur.005G093300 Sapur.006G029800 Sapur.006G036800 Sapur.006G036900 Sapur.006G067400 Sapur.006G069500 Sapur.006G112500 Sapur.006G159000 Sapur.006G172300 Sapur.008G137200 Sapur.009G018700 Sapur.009G122200 Sapur.010G042700 Sapur.010G119100 Sapur.011G018500 Sapur.011G103700 Sapur.011G122100 Sapur.012G037400 Sapur.012G040700 Sapur.013G021800 Sapur.013G071200 Sapur.014G005100 Sapur.014G096700 Sapur.014G100600 Sapur.014G121100 Sapur.016G051800 Sapur.016G052600 Sapur.016G071200 Sapur.016G077400 Sapur.016G127100 Sapur.016G305500 Sapur.018G057600 Sapur.15WG044400 Sapur.15ZG049500 Sapur.T003900 Sapur.009G045200 Sapur.016G152100
GO:0035639	173	2059	purine ribonucleoside triphosphate binding	Sapur.001G040400 Sapur.001G055600 Sapur.001G058800 Sapur.001G078000 Sapur.001G078600 Sapur.001G103000 Sapur.001G103900 Sapur.001G122200 Sapur.001G126100 Sapur.002G002000 Sapur.002G005900 Sapur.002G020300 Sapur.002G051400 Sapur.002G085200 Sapur.002G089300 Sapur.002G093600 Sapur.002G106900 Sapur.002G120400 Sapur.002G171500 Sapur.002G182100 Sapur.002G188100 Sapur.003G020100 Sapur.003G051100 Sapur.003G061800 Sapur.003G073200 Sapur.003G079800 Sapur.003G092100 Sapur.003G099500 Sapur.003G099700 Sapur.003G139700 Sapur.004G019100 Sapur.004G029200 Sapur.004G032900 Sapur.004G051400 Sapur.004G059100 Sapur.004G067800 Sapur.004G089200 Sapur.004G103000 Sapur.004G109700 Sapur.004G120900 Sapur.004G122400 Sapur.004G147000 Sapur.004G170800 Sapur.005G001800 Sapur.005G013900 Sapur.005G015200 Sapur.005G022700 Sapur.005G024100 Sapur.005G025300 Sapur.005G027300 Sapur.005G029900 Sapur.005G050800 Sapur.005G093300 Sapur.005G109200 Sapur.005G206400 Sapur.006G029800 Sapur.006G036800 Sapur.006G036900 Sapur.006G067400 Sapur.006G069500 Sapur.006G096300 Sapur.006G101400 Sapur.006G107000 Sapur.006G111600 Sapur.006G112500 Sapur.006G159000 Sapur.006G172300 Sapur.006G188300 Sapur.006G194600 Sapur.006G195000 Sapur.007G005400 Sapur.007G012300 Sapur.007G016800 Sapur.007G024100 Sapur.007G091900 Sapur.007G127200 Sapur.008G005500 Sapur.008G016100 Sapur.008G113700 Sapur.008G128300 Sapur.008G137200 Sapur.008G143200 Sapur.008G157700 Sapur.009G018000 Sapur.009G018700 Sapur.009G038600 Sapur.009G045200 Sapur.009G066000 Sapur.009G096500 Sapur.009G122200 Sapur.010G041500 Sapur.010G042700 Sapur.010G053300 Sapur.010G057900 Sapur.010G076000 Sapur.010G103300 Sapur.010G118800 Sapur.010G189000 Sapur.011G002200 Sapur.011G002300 Sapur.011G018500 Sapur.011G047800 Sapur.011G072400 Sapur.011G080600 Sapur.011G103500 Sapur.011G103700 Sapur.011G122100 Sapur.012G020400 Sapur.012G029700 Sapur.012G037400 Sapur.012G040700 Sapur.012G067300 Sapur.012G085700 Sapur.012G098500 Sapur.013G003400 Sapur.013G003500 Sapur.013G021800 Sapur.013G071200 Sapur.013G112200 Sapur.013G112400 Sapur.013G141900 Sapur.014G003800 Sapur.014G005100 Sapur.014G039100 Sapur.014G096700 Sapur.014G100600 Sapur.014G121100 Sapur.014G126200 Sapur.016G045300 Sapur.016G046200 Sapur.016G051800 Sapur.016G052600 Sapur.016G071200 Sapur.016G077400 Sapur.016G087900 Sapur.016G127100 Sapur.016G152100 Sapur.016G191700 Sapur.016G205200 Sapur.016G220800 Sapur.016G233300 Sapur.016G294500 Sapur.016G305500 Sapur.017G021900 Sapur.017G107900 Sapur.018G023200 Sapur.018G035500 Sapur.018G047100 Sapur.018G053200 Sapur.018G057600 Sapur.018G087600 Sapur.019G008900 Sapur.019G024300 Sapur.019G028700

				Sapur.019G028900 Sapur.019G048400 Sapur.019G058700 Sapur.019G075600 Sapur.019G087800 Sapur.019G107300 Sapur.019G113000 Sapur.15WG028200 Sapur.15WG044400 Sapur.15ZG005000 Sapur.15ZG013600 Sapur.15ZG018000 Sapur.15ZG027900 Sapur.15ZG049500 Sapur.15ZG121300 Sapur.T003900 Sapur.T124800 Sapur.T138000 Sapur.T191300
GO:0030554	182	2280	adenyl nucleotide binding	Sapur.001G040400 Sapur.001G055600 Sapur.001G058800 Sapur.001G078000 Sapur.001G103000 Sapur.001G103900 Sapur.001G126100 Sapur.002G002000 Sapur.002G005900 Sapur.002G020300 Sapur.002G051400 Sapur.002G085200 Sapur.002G089300 Sapur.002G093600 Sapur.002G106900 Sapur.002G120400 Sapur.002G152400 Sapur.002G171500 Sapur.002G182100 Sapur.002G188100 Sapur.003G020100 Sapur.003G051100 Sapur.003G061800 Sapur.003G073200 Sapur.003G079800 Sapur.003G092100 Sapur.003G099500 Sapur.003G099700 Sapur.003G139700 Sapur.004G019100 Sapur.004G029200 Sapur.004G032900 Sapur.004G051400 Sapur.004G059100 Sapur.004G067800 Sapur.004G071000 Sapur.004G089200 Sapur.004G103000 Sapur.004G109700 Sapur.004G120900 Sapur.004G122400 Sapur.004G147000 Sapur.004G170800 Sapur.005G001800 Sapur.005G013900 Sapur.005G015200 Sapur.005G022700 Sapur.005G024100 Sapur.005G025300 Sapur.005G027300 Sapur.005G029900 Sapur.005G050800 Sapur.005G093300 Sapur.005G109200 Sapur.005G206400 Sapur.006G029800 Sapur.006G036800 Sapur.006G036900 Sapur.006G067400 Sapur.006G069500 Sapur.006G096300 Sapur.006G101400 Sapur.006G107000 Sapur.006G111600 Sapur.006G112500 Sapur.006G159000 Sapur.006G172300 Sapur.006G188300 Sapur.006G194600 Sapur.006G195000 Sapur.006G195400 Sapur.006G201600 Sapur.007G005400 Sapur.007G012300 Sapur.007G016800 Sapur.007G024100 Sapur.007G091900 Sapur.007G127200 Sapur.008G004900 Sapur.008G005500 Sapur.008G016100 Sapur.008G074200 Sapur.008G113700 Sapur.008G128300 Sapur.008G137200 Sapur.008G143200 Sapur.008G157700 Sapur.009G018000 Sapur.009G018700 Sapur.009G038600 Sapur.009G045200 Sapur.009G066000 Sapur.009G096500 Sapur.009G122200 Sapur.010G041500 Sapur.010G042700 Sapur.010G053300 Sapur.010G057900 Sapur.010G076000 Sapur.010G103300 Sapur.010G189000 Sapur.011G002200 Sapur.011G002300 Sapur.011G018500 Sapur.011G047800 Sapur.011G072400 Sapur.011G080600 Sapur.011G103500 Sapur.011G103700 Sapur.011G122100 Sapur.012G020400 Sapur.012G029700 Sapur.012G037400 Sapur.012G040700 Sapur.012G067300 Sapur.012G085700 Sapur.012G098500 Sapur.013G003400 Sapur.013G003500 Sapur.013G021800 Sapur.013G071200 Sapur.013G094000 Sapur.013G141900 Sapur.014G003800 Sapur.014G005100 Sapur.014G096700 Sapur.014G100600 Sapur.014G121100 Sapur.014G126200 Sapur.016G040000 Sapur.016G045300 Sapur.016G046200 Sapur.016G051800 Sapur.016G052600 Sapur.016G053300 Sapur.016G071200 Sapur.016G077400 Sapur.016G087900 Sapur.016G127100 Sapur.016G152100 Sapur.016G191700 Sapur.016G205200 Sapur.016G220800 Sapur.016G233300 Sapur.016G294500 Sapur.016G305500 Sapur.016G309400 Sapur.017G008100 Sapur.017G021900 Sapur.017G084100 Sapur.017G085100 Sapur.017G085800 Sapur.017G107900 Sapur.018G023200 Sapur.018G035500 Sapur.018G047100 Sapur.018G053200 Sapur.018G057600 Sapur.018G087600 Sapur.018G105800 Sapur.019G008900 Sapur.019G024300 Sapur.019G028700 Sapur.019G028900 Sapur.019G046400 Sapur.019G048400 Sapur.019G058700 Sapur.019G075600 Sapur.019G107300 Sapur.019G113000 Sapur.15WG028200 Sapur.15WG044400 Sapur.15ZG005000 Sapur.15ZG013600 Sapur.15ZG018000 Sapur.15ZG027900 Sapur.15ZG049500 Sapur.15ZG121300 Sapur.T003900 Sapur.T124800 Sapur.T138000 Sapur.T191300
GO:0032559	174	2180	adenyl ribonucleotide binding	Sapur.001G040400 Sapur.001G055600 Sapur.001G058800 Sapur.001G078000 Sapur.001G103000 Sapur.001G103900 Sapur.001G126100 Sapur.002G002000 Sapur.002G005900 Sapur.002G020300 Sapur.002G051400 Sapur.002G085200 Sapur.002G089300 Sapur.002G093600 Sapur.002G106900 Sapur.002G120400 Sapur.002G171500 Sapur.002G182100 Sapur.002G188100 Sapur.003G020100 Sapur.003G051100 Sapur.003G061800 Sapur.003G073200 Sapur.003G079800 Sapur.003G092100 Sapur.003G099500 Sapur.003G099700 Sapur.003G139700 Sapur.004G019100 Sapur.004G029200 Sapur.004G032900 Sapur.004G051400 Sapur.004G059100 Sapur.004G067800 Sapur.004G089200 Sapur.004G103000 Sapur.004G109700 Sapur.004G120900 Sapur.004G122400 Sapur.004G147000 Sapur.004G170800 Sapur.005G001800 Sapur.005G013900 Sapur.005G015200 Sapur.005G022700 Sapur.005G024100 Sapur.005G025300 Sapur.005G027300 Sapur.005G029900 Sapur.005G050800 Sapur.005G093300 Sapur.005G109200 Sapur.005G206400 Sapur.006G029800 Sapur.006G036800 Sapur.006G036900 Sapur.006G067400 Sapur.006G069500 Sapur.006G096300 Sapur.006G101400 Sapur.006G107000 Sapur.006G111600 Sapur.006G112500 Sapur.006G159000 Sapur.006G172300 Sapur.006G188300 Sapur.006G194600 Sapur.006G195000 Sapur.007G005400 Sapur.007G012300

				Sapur.007G016800 Sapur.007G024100 Sapur.007G091900 Sapur.007G127200 Sapur.008G005500 Sapur.008G016100 Sapur.008G113700 Sapur.008G128300 Sapur.008G137200 Sapur.008G143200 Sapur.008G157700 Sapur.009G018000 Sapur.009G018700 Sapur.009G038600 Sapur.009G045200 Sapur.009G066000 Sapur.009G096500 Sapur.009G122200 Sapur.010G041500 Sapur.010G042700 Sapur.010G053300 Sapur.010G057900 Sapur.010G076000 Sapur.010G103300 Sapur.010G189000 Sapur.011G002200 Sapur.011G002300 Sapur.011G018500 Sapur.011G047800 Sapur.011G072400 Sapur.011G080600 Sapur.011G103500 Sapur.011G103700 Sapur.011G122100 Sapur.012G020400 Sapur.012G029700 Sapur.012G037400 Sapur.012G040700 Sapur.012G067300 Sapur.012G085700 Sapur.012G098500 Sapur.013G003400 Sapur.013G003500 Sapur.013G021800 Sapur.013G071200 Sapur.013G141900 Sapur.014G003800 Sapur.014G005100 Sapur.014G096700 Sapur.014G100600 Sapur.014G121100 Sapur.014G126200 Sapur.016G045300 Sapur.016G046200 Sapur.016G051800 Sapur.016G052600 Sapur.016G053300 Sapur.016G071200 Sapur.016G077400 Sapur.016G087900 Sapur.016G127100 Sapur.016G152100 Sapur.016G191700 Sapur.016G205200 Sapur.016G220800 Sapur.016G233300 Sapur.016G294500 Sapur.016G305500 Sapur.016G309400 Sapur.017G008100 Sapur.017G021900 Sapur.017G084100 Sapur.017G085100 Sapur.017G085800 Sapur.017G107900 Sapur.018G023200 Sapur.018G035500 Sapur.018G047100 Sapur.018G053200 Sapur.018G057600 Sapur.018G087600 Sapur.018G105800 Sapur.019G008900 Sapur.019G024300 Sapur.019G028700 Sapur.019G028900 Sapur.019G046400 Sapur.019G048400 Sapur.019G058700 Sapur.019G075600 Sapur.019G107300 Sapur.019G113000 Sapur.15WG028200 Sapur.15WG044400 Sapur.15ZG005000 Sapur.15ZG013600 Sapur.15ZG018000 Sapur.15ZG027900 Sapur.15ZG049500 Sapur.15ZG121300 Sapur.T003900 Sapur.T124800 Sapur.T138000 Sapur.T191300
GO:0009055	24	151	electron transfer activity	Sapur.001G033400 Sapur.001G068800 Sapur.002G081300 Sapur.004G152300 Sapur.006G150600 Sapur.006G164600 Sapur.006G173800 Sapur.006G218300 Sapur.007G121300 Sapur.008G010200 Sapur.008G116700 Sapur.008G122100 Sapur.010G059500 Sapur.010G197200 Sapur.014G107200 Sapur.014G107800 Sapur.016G062700 Sapur.016G073300 Sapur.016G200600 Sapur.016G288700 Sapur.017G073000 Sapur.018G013500 Sapur.018G068500 Sapur.018G101000
GO:0016798	44	379	hydrolase activity, acting on glycosyl bonds	Sapur.001G112100 Sapur.001G167900 Sapur.001G171300 Sapur.002G004700 Sapur.002G048300 Sapur.002G182500 Sapur.002G192300 Sapur.003G013400 Sapur.003G118000 Sapur.003G165600 Sapur.004G061300 Sapur.005G190700 Sapur.006G114600 Sapur.006G180700 Sapur.007G051800 Sapur.007G089900 Sapur.008G043800 Sapur.008G062900 Sapur.008G107800 Sapur.008G112200 Sapur.009G059800 Sapur.009G120900 Sapur.010G071500 Sapur.010G081700 Sapur.011G072100 Sapur.011G112600 Sapur.013G135900 Sapur.014G071900 Sapur.014G072000 Sapur.014G091800 Sapur.014G097700 Sapur.014G101600 Sapur.014G129200 Sapur.018G070800 Sapur.019G037200 Sapur.019G068900 Sapur.019G109000 Sapur.15WG032300 Sapur.15ZG036100 Sapur.T171300 Sapur.002G108700 Sapur.014G031500 Sapur.003G077800 Sapur.15ZG117900
GO:0004553	42	357	hydrolase activity, hydrolyzing O-glycosyl compounds	Sapur.001G112100 Sapur.001G167900 Sapur.001G171300 Sapur.002G004700 Sapur.002G048300 Sapur.002G182500 Sapur.002G192300 Sapur.003G013400 Sapur.003G118000 Sapur.003G165600 Sapur.004G061300 Sapur.005G190700 Sapur.006G114600 Sapur.006G180700 Sapur.007G051800 Sapur.007G089900 Sapur.008G043800 Sapur.008G062900 Sapur.008G107800 Sapur.008G112200 Sapur.009G059800 Sapur.009G120900 Sapur.010G071500 Sapur.010G081700 Sapur.011G072100 Sapur.011G112600 Sapur.013G135900 Sapur.014G071900 Sapur.014G072000 Sapur.014G091800 Sapur.014G097700 Sapur.014G101600 Sapur.014G129200 Sapur.018G070800 Sapur.019G037200 Sapur.019G068900 Sapur.019G109000 Sapur.15WG032300 Sapur.15ZG036100 Sapur.T171300 Sapur.003G077800 Sapur.15ZG117900
GO:0005515	241	3312	protein binding	Sapur.001G017600 Sapur.013G009700 Sapur.001G052700 Sapur.002G085200 Sapur.002G089300 Sapur.002G093600 Sapur.002G182100 Sapur.003G086100 Sapur.003G092100 Sapur.003G120200 Sapur.003G148500 Sapur.004G019100 Sapur.004G051400 Sapur.004G109700 Sapur.004G120900 Sapur.004G122400 Sapur.004G147000 Sapur.005G015200 Sapur.005G022700 Sapur.005G024100 Sapur.005G025300 Sapur.005G029900 Sapur.005G093300 Sapur.006G028600 Sapur.006G029800 Sapur.006G036800 Sapur.006G036900 Sapur.006G067400 Sapur.006G069500 Sapur.006G112500 Sapur.006G159000 Sapur.006G172300 Sapur.007G045800 Sapur.008G137200 Sapur.009G018700 Sapur.009G033900 Sapur.009G122200 Sapur.010G042700 Sapur.010G206600 Sapur.011G018500 Sapur.011G103700 Sapur.011G122100 Sapur.012G037400 Sapur.012G040700 Sapur.012G099300 Sapur.013G021800 Sapur.013G071200 Sapur.014G005100 Sapur.014G051500

				Sapur.014G100600 Sapur.014G121100 Sapur.016G051800 Sapur.016G052600 Sapur.016G071200 Sapur.016G077400 Sapur.016G127100 Sapur.016G229500 Sapur.016G305500 Sapur.018G057600 Sapur.15WG044400 Sapur.15ZG049500 Sapur.15ZG122400 Sapur.T003900 Sapur.T124800 Sapur.T138000 Sapur.001G012900 Sapur.001G014400 Sapur.001G018000 Sapur.001G050200 Sapur.001G050300 Sapur.001G082700 Sapur.001G096300 Sapur.001G103000 Sapur.001G103900 Sapur.001G155400 Sapur.001G160100 Sapur.002G012000 Sapur.002G012200 Sapur.002G020300 Sapur.002G028900 Sapur.002G030600 Sapur.002G033900 Sapur.002G095500 Sapur.002G120400 Sapur.002G131700 Sapur.002G153800 Sapur.002G182000 Sapur.002G187700 Sapur.002G188100 Sapur.002G193000 Sapur.002G195500 Sapur.002G195600 Sapur.003G017500 Sapur.003G021500 Sapur.003G042300 Sapur.003G073200 Sapur.003G079800 Sapur.003G092200 Sapur.003G094600 Sapur.003G099700 Sapur.003G126500 Sapur.003G139700 Sapur.003G155800 Sapur.003G159800 Sapur.004G000300 Sapur.004G010500 Sapur.004G032900 Sapur.004G041400 Sapur.004G059100 Sapur.004G067800 Sapur.004G079700 Sapur.004G170800 Sapur.005G094100 Sapur.005G094500 Sapur.005G108800 Sapur.005G109000 Sapur.005G114600 Sapur.005G115800 Sapur.005G165400 Sapur.005G181800 Sapur.005G188000 Sapur.005G195900 Sapur.006G020100 Sapur.006G026600 Sapur.006G026800 Sapur.006G037000 Sapur.006G044000 Sapur.006G048200 Sapur.006G056400 Sapur.006G058700 Sapur.006G066100 Sapur.006G066400 Sapur.006G079300 Sapur.006G085200 Sapur.006G186700 Sapur.006G188300 Sapur.006G195000 Sapur.006G218100 Sapur.006G221200 Sapur.007G000800 Sapur.007G005600 Sapur.007G012300 Sapur.007G024400 Sapur.007G045900 Sapur.007G099600 Sapur.007G130800 Sapur.008G044400 Sapur.008G070300 Sapur.008G074900 Sapur.008G093400 Sapur.008G113700 Sapur.008G143200 Sapur.009G069800 Sapur.009G111300 Sapur.010G029700 Sapur.010G038100 Sapur.010G039900 Sapur.010G057800 Sapur.010G076000 Sapur.010G100500 Sapur.010G143200 Sapur.010G158700 Sapur.011G013000 Sapur.011G039000 Sapur.011G046900 Sapur.011G047800 Sapur.011G048700 Sapur.011G060200 Sapur.011G063700 Sapur.011G064200 Sapur.011G065500 Sapur.011G080600 Sapur.011G104100 Sapur.011G111500 Sapur.011G120600 Sapur.012G020400 Sapur.012G029700 Sapur.012G046200 Sapur.012G051500 Sapur.012G065600 Sapur.012G067300 Sapur.012G098500 Sapur.012G107400 Sapur.013G022800 Sapur.013G044800 Sapur.013G045600 Sapur.013G050200 Sapur.013G068400 Sapur.013G068600 Sapur.013G106400 Sapur.013G141900 Sapur.014G007400 Sapur.014G032600 Sapur.014G060200 Sapur.014G081700 Sapur.014G097800 Sapur.014G099300 Sapur.014G107000 Sapur.014G121000 Sapur.016G010400 Sapur.016G031300 Sapur.016G043900 Sapur.016G045300 Sapur.016G045700 Sapur.016G046200 Sapur.016G049500 Sapur.016G053300 Sapur.016G082200 Sapur.016G085200 Sapur.016G103300 Sapur.016G109100 Sapur.016G121300 Sapur.016G142200 Sapur.016G169900 Sapur.016G218300 Sapur.016G245500 Sapur.016G303900 Sapur.017G084100 Sapur.017G085100 Sapur.017G085800 Sapur.017G107900 Sapur.018G020000 Sapur.018G035500 Sapur.018G053200 Sapur.018G077800 Sapur.018G082400 Sapur.018G115600 Sapur.019G029700 Sapur.019G032900 Sapur.019G061700 Sapur.019G081500 Sapur.019G094800 Sapur.019G107300 Sapur.019G113000 Sapur.15WG028200 Sapur.15ZG000400 Sapur.15ZG018000 Sapur.15ZG027900 Sapur.15ZG121300 Sapur.T145000 Sapur.T191300
GO:0017076	189	2516	purine nucleotide binding	Sapur.001G040400 Sapur.001G055600 Sapur.001G058800 Sapur.001G078000 Sapur.001G078600 Sapur.001G103000 Sapur.001G103900 Sapur.001G122200 Sapur.001G126100 Sapur.002G002000 Sapur.002G005900 Sapur.002G020300 Sapur.002G051400 Sapur.002G085200 Sapur.002G089300 Sapur.002G093600 Sapur.002G106900 Sapur.002G120400 Sapur.002G152400 Sapur.002G171500 Sapur.002G182100 Sapur.002G188100 Sapur.003G020100 Sapur.003G051100 Sapur.003G061800 Sapur.003G073200 Sapur.003G079800 Sapur.003G092100 Sapur.003G099500 Sapur.003G099700 Sapur.003G139700 Sapur.004G019100 Sapur.004G029200 Sapur.004G032900 Sapur.004G051400 Sapur.004G059100 Sapur.004G067800 Sapur.004G071000 Sapur.004G089200 Sapur.004G103000 Sapur.004G109700 Sapur.004G120900 Sapur.004G122400 Sapur.004G147000 Sapur.004G170800 Sapur.005G001800 Sapur.005G013900 Sapur.005G015200 Sapur.005G022700 Sapur.005G024100 Sapur.005G025300 Sapur.005G027300 Sapur.005G029900 Sapur.005G050800 Sapur.005G093300 Sapur.005G109200 Sapur.005G206400 Sapur.006G029800 Sapur.006G036800 Sapur.006G036900 Sapur.006G067400 Sapur.006G069500 Sapur.006G096300 Sapur.006G101400 Sapur.006G107000 Sapur.006G111600 Sapur.006G112500 Sapur.006G159000 Sapur.006G172300 Sapur.006G188300 Sapur.006G194600 Sapur.006G195000 Sapur.006G195400 Sapur.006G201600 Sapur.007G005400 Sapur.007G012300 Sapur.007G016800

				Sapur.007G024100 Sapur.007G091900 Sapur.007G127200 Sapur.008G004900 Sapur.008G005500 Sapur.008G016100 Sapur.008G074200 Sapur.008G113700 Sapur.008G128300 Sapur.008G137200 Sapur.008G143200 Sapur.008G157700 Sapur.009G018000 Sapur.009G018700 Sapur.009G038600 Sapur.009G045200 Sapur.009G066000 Sapur.009G096500 Sapur.009G122200 Sapur.010G041500 Sapur.010G042700 Sapur.010G053300 Sapur.010G057900 Sapur.010G076000 Sapur.010G103300 Sapur.010G118800 Sapur.010G189000 Sapur.011G002200 Sapur.011G002300 Sapur.011G018500 Sapur.011G047800 Sapur.011G072400 Sapur.011G080600 Sapur.011G103500 Sapur.011G103700 Sapur.011G122100 Sapur.012G020400 Sapur.012G029700 Sapur.012G037400 Sapur.012G040700 Sapur.012G067300 Sapur.012G085700 Sapur.012G098500 Sapur.013G003400 Sapur.013G003500 Sapur.013G021800 Sapur.013G071200 Sapur.013G094000 Sapur.013G112200 Sapur.013G112400 Sapur.013G141900 Sapur.014G003800 Sapur.014G005100 Sapur.014G039100 Sapur.014G096700 Sapur.014G100600 Sapur.014G121100 Sapur.014G126200 Sapur.016G040000 Sapur.016G045300 Sapur.016G046200 Sapur.016G051800 Sapur.016G052600 Sapur.016G053300 Sapur.016G071200 Sapur.016G077400 Sapur.016G087900 Sapur.016G127100 Sapur.016G152100 Sapur.016G191700 Sapur.016G205200 Sapur.016G220800 Sapur.016G233300 Sapur.016G294500 Sapur.016G305500 Sapur.016G309400 Sapur.017G008100 Sapur.017G021900 Sapur.017G084100 Sapur.017G085100 Sapur.017G085800 Sapur.017G107900 Sapur.018G023200 Sapur.018G035500 Sapur.018G047100 Sapur.018G053200 Sapur.018G057600 Sapur.018G087600 Sapur.018G105800 Sapur.019G008900 Sapur.019G024300 Sapur.019G028700 Sapur.019G028900 Sapur.019G046400 Sapur.019G048400 Sapur.019G058700 Sapur.019G075600 Sapur.019G087800 Sapur.019G107300 Sapur.019G113000 Sapur.15WG028200 Sapur.15WG044400 Sapur.15ZG005000 Sapur.15ZG013600 Sapur.15ZG018000 Sapur.15ZG027900 Sapur.15ZG049500 Sapur.15ZG121300 Sapur.T003900 Sapur.T124800 Sapur.T138000 Sapur.T191300
GO:0032555	181	2416	purine ribonucleotide binding	Sapur.001G040400 Sapur.001G055600 Sapur.001G058800 Sapur.001G078000 Sapur.001G078600 Sapur.001G103000 Sapur.001G103900 Sapur.001G122200 Sapur.001G126100 Sapur.002G002000 Sapur.002G005900 Sapur.002G020300 Sapur.002G051400 Sapur.002G085200 Sapur.002G089300 Sapur.002G093600 Sapur.002G106900 Sapur.002G120400 Sapur.002G171500 Sapur.002G182100 Sapur.002G188100 Sapur.003G020100 Sapur.003G051100 Sapur.003G061800 Sapur.003G073200 Sapur.003G079800 Sapur.003G092100 Sapur.003G099500 Sapur.003G099700 Sapur.003G139700 Sapur.004G019100 Sapur.004G029200 Sapur.004G032900 Sapur.004G051400 Sapur.004G059100 Sapur.004G067800 Sapur.004G089200 Sapur.004G103000 Sapur.004G109700 Sapur.004G120900 Sapur.004G122400 Sapur.004G147000 Sapur.004G170800 Sapur.005G001800 Sapur.005G013900 Sapur.005G015200 Sapur.005G022700 Sapur.005G024100 Sapur.005G025300 Sapur.005G027300 Sapur.005G029900 Sapur.005G050800 Sapur.005G093300 Sapur.005G109200 Sapur.005G206400 Sapur.006G029800 Sapur.006G036800 Sapur.006G036900 Sapur.006G067400 Sapur.006G069500 Sapur.006G096300 Sapur.006G101400 Sapur.006G107000 Sapur.006G111600 Sapur.006G112500 Sapur.006G159000 Sapur.006G172300 Sapur.006G188300 Sapur.006G194600 Sapur.006G195000 Sapur.007G005400 Sapur.007G012300 Sapur.007G016800 Sapur.007G024100 Sapur.007G091900 Sapur.007G127200 Sapur.008G005500 Sapur.008G016100 Sapur.008G113700 Sapur.008G128300 Sapur.008G137200 Sapur.008G143200 Sapur.008G157700 Sapur.009G018000 Sapur.009G018700 Sapur.009G038600 Sapur.009G045200 Sapur.009G066000 Sapur.009G096500 Sapur.009G122200 Sapur.010G041500 Sapur.010G042700 Sapur.010G053300 Sapur.010G057900 Sapur.010G076000 Sapur.010G103300 Sapur.010G118800 Sapur.010G189000 Sapur.011G002200 Sapur.011G002300 Sapur.011G018500 Sapur.011G047800 Sapur.011G072400 Sapur.011G080600 Sapur.011G103500 Sapur.011G103700 Sapur.011G122100 Sapur.012G020400 Sapur.012G029700 Sapur.012G037400 Sapur.012G040700 Sapur.012G067300 Sapur.012G085700 Sapur.012G098500 Sapur.013G003400 Sapur.013G003500 Sapur.013G021800 Sapur.013G071200 Sapur.013G112200 Sapur.013G112400 Sapur.013G141900 Sapur.014G003800 Sapur.014G005100 Sapur.014G039100 Sapur.014G096700 Sapur.014G100600 Sapur.014G121100 Sapur.014G126200 Sapur.016G045300 Sapur.016G046200 Sapur.016G051800 Sapur.016G052600 Sapur.016G053300 Sapur.016G071200 Sapur.016G077400 Sapur.016G087900 Sapur.016G127100 Sapur.016G152100 Sapur.016G191700 Sapur.016G205200 Sapur.016G220800 Sapur.016G233300 Sapur.016G294500 Sapur.016G305500 Sapur.016G309400 Sapur.017G008100 Sapur.017G021900 Sapur.017G084100 Sapur.017G085100 Sapur.017G085800 Sapur.017G107900 Sapur.018G023200 Sapur.018G035500 Sapur.018G047100 Sapur.018G053200 Sapur.018G057600 Sapur.018G087600 Sapur.018G105800 Sapur.019G008900 Sapur.019G024300 Sapur.019G028700

				Sapur.019G028900 Sapur.019G046400 Sapur.019G048400 Sapur.019G058700 Sapur.019G075600 Sapur.019G087800 Sapur.019G107300 Sapur.019G113000 Sapur.15WG028200 Sapur.15WG044400 Sapur.15ZG005000 Sapur.15ZG013600 Sapur.15ZG018000 Sapur.15ZG027900 Sapur.15ZG049500 Sapur.15ZG121300 Sapur.T003900 Sapur.T124800 Sapur.T138000 Sapur.T191300
GO:1901265	199	2702	nucleoside phosphate binding	Sapur.001G017600 Sapur.001G040400 Sapur.001G055600 Sapur.001G058800 Sapur.001G078000 Sapur.001G078600 Sapur.001G103000 Sapur.001G103900 Sapur.001G122200 Sapur.001G126100 Sapur.002G002000 Sapur.002G005900 Sapur.002G020300 Sapur.002G051400 Sapur.002G085200 Sapur.002G089300 Sapur.002G093600 Sapur.002G106900 Sapur.002G120400 Sapur.002G152400 Sapur.002G171500 Sapur.002G182100 Sapur.002G188100 Sapur.003G020100 Sapur.003G051100 Sapur.003G061800 Sapur.003G073200 Sapur.003G079800 Sapur.003G092100 Sapur.003G099500 Sapur.003G099700 Sapur.003G139700 Sapur.004G019100 Sapur.004G029200 Sapur.004G032900 Sapur.004G051400 Sapur.004G059100 Sapur.004G067800 Sapur.004G071000 Sapur.004G089200 Sapur.004G103000 Sapur.004G109700 Sapur.004G120900 Sapur.004G122400 Sapur.004G139900 Sapur.004G147000 Sapur.004G170800 Sapur.005G001800 Sapur.005G013900 Sapur.005G015200 Sapur.005G022700 Sapur.005G024100 Sapur.005G025300 Sapur.005G027300 Sapur.005G029900 Sapur.005G050800 Sapur.005G093300 Sapur.005G109200 Sapur.005G206400 Sapur.006G029800 Sapur.006G036800 Sapur.006G036900 Sapur.006G067400 Sapur.006G069500 Sapur.006G096300 Sapur.006G101400 Sapur.006G107000 Sapur.006G111600 Sapur.006G112500 Sapur.006G159000 Sapur.006G172300 Sapur.006G188300 Sapur.006G194600 Sapur.006G195000 Sapur.006G195400 Sapur.006G201600 Sapur.007G005400 Sapur.007G012300 Sapur.007G016800 Sapur.007G024100 Sapur.007G042700 Sapur.007G091900 Sapur.007G127200 Sapur.008G004900 Sapur.008G005500 Sapur.008G016100 Sapur.008G067000 Sapur.008G074200 Sapur.008G113700 Sapur.008G128300 Sapur.008G129000 Sapur.008G137200 Sapur.008G143200 Sapur.008G157700 Sapur.009G018000 Sapur.009G018700 Sapur.009G038600 Sapur.009G045200 Sapur.009G066000 Sapur.009G096500 Sapur.009G122200 Sapur.010G041500 Sapur.010G042700 Sapur.010G053300 Sapur.010G057900 Sapur.010G076000 Sapur.010G103300 Sapur.010G118800 Sapur.010G129400 Sapur.010G189000 Sapur.011G002200 Sapur.011G002300 Sapur.011G018500 Sapur.011G047800 Sapur.011G072400 Sapur.011G080600 Sapur.011G103500 Sapur.011G103700 Sapur.011G122100 Sapur.012G020400 Sapur.012G021700 Sapur.012G029700 Sapur.012G037400 Sapur.012G040700 Sapur.012G067300 Sapur.012G085700 Sapur.012G098500 Sapur.013G003400 Sapur.013G003500 Sapur.013G021800 Sapur.013G037700 Sapur.013G071200 Sapur.013G094000 Sapur.013G112200 Sapur.013G112400 Sapur.013G141900 Sapur.014G003800 Sapur.014G005100 Sapur.014G039100 Sapur.014G096700 Sapur.014G100600 Sapur.014G121100 Sapur.014G126200 Sapur.016G018800 Sapur.016G040000 Sapur.016G045300 Sapur.016G046200 Sapur.016G051800 Sapur.016G052600 Sapur.016G053300 Sapur.016G071200 Sapur.016G077400 Sapur.016G087900 Sapur.016G127100 Sapur.016G152100 Sapur.016G191700 Sapur.016G205200 Sapur.016G220800 Sapur.016G233300 Sapur.016G294500 Sapur.016G300800 Sapur.016G305500 Sapur.016G309400 Sapur.017G008100 Sapur.017G021900 Sapur.017G084100 Sapur.017G085100 Sapur.017G085800 Sapur.017G107900 Sapur.018G023200 Sapur.018G035500 Sapur.018G047100 Sapur.018G053200 Sapur.018G057600 Sapur.018G087600 Sapur.018G105800 Sapur.019G008900 Sapur.019G024300 Sapur.019G028700 Sapur.019G028900 Sapur.019G046400 Sapur.019G048400 Sapur.019G058700 Sapur.019G075600 Sapur.019G087800 Sapur.019G107300 Sapur.019G113000 Sapur.15WG028200 Sapur.15WG044400 Sapur.15ZG005000 Sapur.15ZG013600 Sapur.15ZG018000 Sapur.15ZG027900 Sapur.15ZG049500 Sapur.15ZG121300 Sapur.T003900 Sapur.T124800 Sapur.T138000 Sapur.T191300
GO:0000166	199	2702	nucleotide binding	Sapur.001G017600 Sapur.001G040400 Sapur.001G055600 Sapur.001G058800 Sapur.001G078000 Sapur.001G078600 Sapur.001G103000 Sapur.001G103900 Sapur.001G122200 Sapur.001G126100 Sapur.002G002000 Sapur.002G005900 Sapur.002G020300 Sapur.002G051400 Sapur.002G085200 Sapur.002G089300 Sapur.002G093600 Sapur.002G106900 Sapur.002G120400 Sapur.002G152400 Sapur.002G171500 Sapur.002G182100 Sapur.002G188100 Sapur.003G020100 Sapur.003G051100 Sapur.003G061800 Sapur.003G073200 Sapur.003G079800 Sapur.003G092100 Sapur.003G099500 Sapur.003G099700 Sapur.003G139700 Sapur.004G019100 Sapur.004G029200 Sapur.004G032900 Sapur.004G051400 Sapur.004G059100 Sapur.004G067800 Sapur.004G071000 Sapur.004G089200 Sapur.004G103000 Sapur.004G109700 Sapur.004G120900 Sapur.004G122400 Sapur.004G139900 Sapur.004G147000 Sapur.004G170800 Sapur.005G001800 Sapur.005G013900

				Sapur.005G015200 Sapur.005G022700 Sapur.005G024100 Sapur.005G025300 Sapur.005G027300 Sapur.005G029900 Sapur.005G050800 Sapur.005G093300 Sapur.005G109200 Sapur.005G206400 Sapur.006G029800 Sapur.006G036800 Sapur.006G036900 Sapur.006G067400 Sapur.006G069500 Sapur.006G096300 Sapur.006G101400 Sapur.006G107000 Sapur.006G111600 Sapur.006G112500 Sapur.006G159000 Sapur.006G172300 Sapur.006G188300 Sapur.006G194600 Sapur.006G195000 Sapur.006G195400 Sapur.006G201600 Sapur.007G005400 Sapur.007G012300 Sapur.007G016800 Sapur.007G024100 Sapur.007G042700 Sapur.007G091900 Sapur.007G127200 Sapur.008G004900 Sapur.008G005500 Sapur.008G016100 Sapur.008G067000 Sapur.008G074200 Sapur.008G113700 Sapur.008G128300 Sapur.008G129000 Sapur.008G137200 Sapur.008G143200 Sapur.008G157700 Sapur.009G018000 Sapur.009G018700 Sapur.009G038600 Sapur.009G045200 Sapur.009G066000 Sapur.009G096500 Sapur.009G122200 Sapur.010G041500 Sapur.010G042700 Sapur.010G053300 Sapur.010G057900 Sapur.010G076000 Sapur.010G103300 Sapur.010G118800 Sapur.010G129400 Sapur.010G189000 Sapur.011G002200 Sapur.011G002300 Sapur.011G018500 Sapur.011G047800 Sapur.011G072400 Sapur.011G080600 Sapur.011G103500 Sapur.011G103700 Sapur.011G122100 Sapur.012G020400 Sapur.012G021700 Sapur.012G029700 Sapur.012G037400 Sapur.012G040700 Sapur.012G067300 Sapur.012G085700 Sapur.012G098500 Sapur.013G003400 Sapur.013G003500 Sapur.013G021800 Sapur.013G037700 Sapur.013G071200 Sapur.013G094000 Sapur.013G112200 Sapur.013G112400 Sapur.013G141900 Sapur.014G003800 Sapur.014G005100 Sapur.014G039100 Sapur.014G096700 Sapur.014G100600 Sapur.014G121100 Sapur.014G126200 Sapur.016G018800 Sapur.016G040000 Sapur.016G045300 Sapur.016G046200 Sapur.016G051800 Sapur.016G052600 Sapur.016G053300 Sapur.016G071200 Sapur.016G077400 Sapur.016G087900 Sapur.016G127100 Sapur.016G152100 Sapur.016G191700 Sapur.016G205200 Sapur.016G220800 Sapur.016G233300 Sapur.016G294500 Sapur.016G300800 Sapur.016G305500 Sapur.016G309400 Sapur.017G008100 Sapur.017G021900 Sapur.017G084100 Sapur.017G085100 Sapur.017G085800 Sapur.017G107900 Sapur.018G023200 Sapur.018G035500 Sapur.018G047100 Sapur.018G053200 Sapur.018G057600 Sapur.018G087600 Sapur.018G105800 Sapur.019G008900 Sapur.019G024300 Sapur.019G028700 Sapur.019G028900 Sapur.019G046400 Sapur.019G048400 Sapur.019G058700 Sapur.019G075600 Sapur.019G087800 Sapur.019G107300 Sapur.019G113000 Sapur.15WG028200 Sapur.15WG044400 Sapur.15ZG005000 Sapur.15ZG013600 Sapur.15ZG018000 Sapur.15ZG027900 Sapur.15ZG049500 Sapur.15ZG121300 Sapur.T003900 Sapur.T124800 Sapur.T138000 Sapur.T191300
GO:0032553	182	2442	ribonucleotide binding	Sapur.001G040400 Sapur.001G055600 Sapur.001G058800 Sapur.001G078000 Sapur.001G078600 Sapur.001G103000 Sapur.001G103900 Sapur.001G122200 Sapur.001G126100 Sapur.002G002000 Sapur.002G005900 Sapur.002G020300 Sapur.002G051400 Sapur.002G085200 Sapur.002G089300 Sapur.002G093600 Sapur.002G106900 Sapur.002G120400 Sapur.002G171500 Sapur.002G182100 Sapur.002G188100 Sapur.003G020100 Sapur.003G051100 Sapur.003G061800 Sapur.003G073200 Sapur.003G079800 Sapur.003G092100 Sapur.003G099500 Sapur.003G099700 Sapur.003G139700 Sapur.004G019100 Sapur.004G029200 Sapur.004G032900 Sapur.004G051400 Sapur.004G059100 Sapur.004G067800 Sapur.004G089200 Sapur.004G103000 Sapur.004G109700 Sapur.004G120900 Sapur.004G122400 Sapur.004G147000 Sapur.004G170800 Sapur.005G001800 Sapur.005G013900 Sapur.005G015200 Sapur.005G022700 Sapur.005G024100 Sapur.005G025300 Sapur.005G027300 Sapur.005G029900 Sapur.005G050800 Sapur.005G093300 Sapur.005G109200 Sapur.005G206400 Sapur.006G029800 Sapur.006G036800 Sapur.006G036900 Sapur.006G067400 Sapur.006G069500 Sapur.006G096300 Sapur.006G101400 Sapur.006G107000 Sapur.006G111600 Sapur.006G112500 Sapur.006G159000 Sapur.006G172300 Sapur.006G188300 Sapur.006G194600 Sapur.006G195000 Sapur.007G005400 Sapur.007G012300 Sapur.007G016800 Sapur.007G024100 Sapur.007G091900 Sapur.007G127200 Sapur.008G005500 Sapur.008G016100 Sapur.008G113700 Sapur.008G128300 Sapur.008G137200 Sapur.008G143200 Sapur.008G157700 Sapur.009G018000 Sapur.009G018700 Sapur.009G038600 Sapur.009G045200 Sapur.009G066000 Sapur.009G096500 Sapur.009G122200 Sapur.010G041500 Sapur.010G042700 Sapur.010G053300 Sapur.010G057900 Sapur.010G076000 Sapur.010G103300 Sapur.010G118800 Sapur.010G189000 Sapur.011G002200 Sapur.011G002300 Sapur.011G018500 Sapur.011G047800 Sapur.011G072400 Sapur.011G080600 Sapur.011G103500 Sapur.011G103700 Sapur.011G122100 Sapur.012G020400 Sapur.012G029700 Sapur.012G037400 Sapur.012G040700 Sapur.012G067300 Sapur.012G085700 Sapur.012G098500 Sapur.013G003400 Sapur.013G003500 Sapur.013G021800 Sapur.013G071200 Sapur.013G112200

				Sapur.013G112400 Sapur.013G141900 Sapur.014G003800 Sapur.014G005100 Sapur.014G039100 Sapur.014G096700 Sapur.014G100600 Sapur.014G121100 Sapur.014G126200 Sapur.016G018800 Sapur.016G045300 Sapur.016G046200 Sapur.016G051800 Sapur.016G052600 Sapur.016G053300 Sapur.016G071200 Sapur.016G077400 Sapur.016G087900 Sapur.016G127100 Sapur.016G152100 Sapur.016G191700 Sapur.016G205200 Sapur.016G220800 Sapur.016G233300 Sapur.016G294500 Sapur.016G305500 Sapur.016G309400 Sapur.017G008100 Sapur.017G021900 Sapur.017G084100 Sapur.017G085100 Sapur.017G085800 Sapur.017G107900 Sapur.018G023200 Sapur.018G035500 Sapur.018G047100 Sapur.018G053200 Sapur.018G057600 Sapur.018G087600 Sapur.018G105800 Sapur.019G008900 Sapur.019G024300 Sapur.019G028700 Sapur.019G028900 Sapur.019G046400 Sapur.019G048400 Sapur.019G058700 Sapur.019G075600 Sapur.019G087800 Sapur.019G107300 Sapur.019G113000 Sapur.15WG028200 Sapur.15WG044400 Sapur.15ZG005000 Sapur.15ZG013600 Sapur.15ZG018000 Sapur.15ZG027900 Sapur.15ZG049500 Sapur.15ZG121300 Sapur.T003900 Sapur.T124800 Sapur.T138000 Sapur.T191300
GO:0097367	183	2474	carbohydrate derivative binding	Sapur.001G040400 Sapur.001G055600 Sapur.001G058800 Sapur.001G078000 Sapur.001G078600 Sapur.001G103000 Sapur.001G103900 Sapur.001G122200 Sapur.001G126100 Sapur.002G002000 Sapur.002G005900 Sapur.002G020300 Sapur.002G051400 Sapur.002G085200 Sapur.002G089300 Sapur.002G093600 Sapur.002G106900 Sapur.002G120400 Sapur.002G171500 Sapur.002G182100 Sapur.002G188100 Sapur.003G020100 Sapur.003G051100 Sapur.003G061800 Sapur.003G073200 Sapur.003G079800 Sapur.003G092100 Sapur.003G099500 Sapur.003G099700 Sapur.003G139700 Sapur.004G019100 Sapur.004G029200 Sapur.004G032900 Sapur.004G051400 Sapur.004G059100 Sapur.004G067800 Sapur.004G089200 Sapur.004G103000 Sapur.004G109700 Sapur.004G120900 Sapur.004G122400 Sapur.004G147000 Sapur.004G170800 Sapur.005G001800 Sapur.005G013900 Sapur.005G015200 Sapur.005G022700 Sapur.005G024100 Sapur.005G025300 Sapur.005G027300 Sapur.005G029900 Sapur.005G050800 Sapur.005G093300 Sapur.005G109200 Sapur.005G206400 Sapur.006G029800 Sapur.006G036800 Sapur.006G036900 Sapur.006G067400 Sapur.006G069500 Sapur.006G096300 Sapur.006G101400 Sapur.006G107000 Sapur.006G111600 Sapur.006G112500 Sapur.006G159000 Sapur.006G172300 Sapur.006G188300 Sapur.006G194600 Sapur.006G195000 Sapur.007G005400 Sapur.007G012300 Sapur.007G016800 Sapur.007G024100 Sapur.007G091900 Sapur.007G127200 Sapur.008G005500 Sapur.008G016100 Sapur.008G113700 Sapur.008G128300 Sapur.008G137200 Sapur.008G143200 Sapur.008G157700 Sapur.009G018000 Sapur.009G018700 Sapur.009G038600 Sapur.009G045200 Sapur.009G066000 Sapur.009G096500 Sapur.009G122200 Sapur.010G041500 Sapur.010G042700 Sapur.010G053300 Sapur.010G057900 Sapur.010G076000 Sapur.010G103300 Sapur.010G118800 Sapur.010G189000 Sapur.011G002200 Sapur.011G002300 Sapur.011G018500 Sapur.011G047800 Sapur.011G072400 Sapur.011G080600 Sapur.011G103500 Sapur.011G103700 Sapur.011G122100 Sapur.012G020400 Sapur.012G029700 Sapur.012G037400 Sapur.012G040700 Sapur.012G067300 Sapur.012G085700 Sapur.012G098500 Sapur.013G003400 Sapur.013G003500 Sapur.013G021800 Sapur.013G071200 Sapur.013G112200 Sapur.013G112400 Sapur.013G141900 Sapur.014G003800 Sapur.014G005100 Sapur.014G039100 Sapur.014G096700 Sapur.014G100600 Sapur.014G121100 Sapur.014G126200 Sapur.016G018800 Sapur.016G045300 Sapur.016G046200 Sapur.016G051800 Sapur.016G052600 Sapur.016G053300 Sapur.016G071200 Sapur.016G077400 Sapur.016G087900 Sapur.016G127100 Sapur.016G152100 Sapur.016G191700 Sapur.016G205200 Sapur.016G220800 Sapur.016G233300 Sapur.016G294500 Sapur.016G305500 Sapur.016G309400 Sapur.017G008100 Sapur.017G021900 Sapur.017G084100 Sapur.017G085100 Sapur.017G085800 Sapur.017G107900 Sapur.018G023200 Sapur.018G035500 Sapur.018G047100 Sapur.018G053200 Sapur.018G057600 Sapur.018G087600 Sapur.018G105800 Sapur.019G008900 Sapur.019G024300 Sapur.019G028700 Sapur.019G028900 Sapur.019G046400 Sapur.019G048400 Sapur.019G058700 Sapur.019G075600 Sapur.019G087800 Sapur.019G107300 Sapur.019G113000 Sapur.15WG028200 Sapur.15WG044400 Sapur.15ZG005000 Sapur.15ZG013600 Sapur.15ZG018000 Sapur.15ZG027900 Sapur.15ZG049500 Sapur.15ZG121300 Sapur.T003900 Sapur.T124800 Sapur.T138000 Sapur.T191300 Sapur.007G042700
GO:0005507	15	85	copper ion binding	Sapur.001G098800 Sapur.002G009300 Sapur.003G077900 Sapur.004G006100 Sapur.004G135900 Sapur.007G036000 Sapur.008G058700 Sapur.010G143300 Sapur.012G096900 Sapur.014G079000 Sapur.016G116800 Sapur.019G084800 Sapur.019G084900 Sapur.019G085000 Sapur.15ZG118100
GO:0140	180	2472	catalytic activity,	Sapur.001G023400 Sapur.001G038800 Sapur.001G078000 Sapur.001G088500 Sapur.001G096300 Sapur.001G098400 Sapur.001G103900

096			acting on a protein	Sapur.001G126100 Sapur.001G138800 Sapur.002G002000 Sapur.002G005900 Sapur.002G013000 Sapur.002G020300 Sapur.002G028900 Sapur.002G051400 Sapur.002G060000 Sapur.002G074000 Sapur.002G097300 Sapur.002G101500 Sapur.002G106900 Sapur.002G120400 Sapur.002G143900 Sapur.002G188100 Sapur.002G195600 Sapur.003G037300 Sapur.003G051100 Sapur.003G056800 Sapur.003G061800 Sapur.003G073200 Sapur.003G079800 Sapur.003G088100 Sapur.003G099700 Sapur.003G139700 Sapur.004G029200 Sapur.004G032900 Sapur.004G059100 Sapur.004G060000 Sapur.004G067800 Sapur.004G127600 Sapur.004G164900 Sapur.004G170800 Sapur.005G001800 Sapur.005G027300 Sapur.005G044000 Sapur.005G050800 Sapur.005G109200 Sapur.005G161200 Sapur.005G188000 Sapur.005G206400 Sapur.006G040500 Sapur.006G044000 Sapur.006G061500 Sapur.006G065500 Sapur.006G077000 Sapur.006G085200 Sapur.006G090100 Sapur.006G092500 Sapur.006G093700 Sapur.006G096300 Sapur.006G107000 Sapur.006G164600 Sapur.006G169900 Sapur.006G173800 Sapur.006G188300 Sapur.006G194600 Sapur.006G195000 Sapur.007G012300 Sapur.007G042300 Sapur.007G045900 Sapur.007G094200 Sapur.007G099400 Sapur.007G099800 Sapur.007G121300 Sapur.007G127200 Sapur.008G010200 Sapur.008G016100 Sapur.008G054500 Sapur.008G093300 Sapur.008G113700 Sapur.008G116700 Sapur.008G143200 Sapur.008G157700 Sapur.009G130800 Sapur.010G028600 Sapur.010G041500 Sapur.010G057900 Sapur.010G070800 Sapur.010G076000 Sapur.010G098600 Sapur.010G143200 Sapur.010G147600 Sapur.010G171000 Sapur.010G189000 Sapur.010G197200 Sapur.011G002200 Sapur.011G002300 Sapur.011G024200 Sapur.011G046900 Sapur.011G047800 Sapur.011G072400 Sapur.011G080600 Sapur.011G103500 Sapur.012G020400 Sapur.012G029700 Sapur.012G048500 Sapur.012G051500 Sapur.012G052000 Sapur.012G067300 Sapur.012G097700 Sapur.012G098500 Sapur.013G003400 Sapur.013G020900 Sapur.013G028400 Sapur.013G134300 Sapur.013G141900 Sapur.014G043900 Sapur.014G063400 Sapur.014G106100 Sapur.014G107200 Sapur.014G107800 Sapur.014G126200 Sapur.016G045300 Sapur.016G046200 Sapur.016G049500 Sapur.016G062700 Sapur.016G073300 Sapur.016G109100 Sapur.016G133500 Sapur.016G191700 Sapur.016G200600 Sapur.016G220500 Sapur.016G233300 Sapur.016G263500 Sapur.016G274100 Sapur.016G285600 Sapur.016G288700 Sapur.016G294500 Sapur.017G021900 Sapur.017G107900 Sapur.018G018700 Sapur.018G023200 Sapur.018G035500 Sapur.018G053200 Sapur.018G060600 Sapur.018G082400 Sapur.018G084700 Sapur.019G008900 Sapur.019G048400 Sapur.019G058700 Sapur.019G061700 Sapur.019G065500 Sapur.019G107300 Sapur.019G113000 Sapur.15WG028200 Sapur.15WG063200 Sapur.15WG079300 Sapur.15ZG005000 Sapur.15ZG013600 Sapur.15ZG018000 Sapur.15ZG027900 Sapur.15ZG068100 Sapur.15ZG121300 Sapur.T191300 Sapur.001G051400 Sapur.002G056400 Sapur.003G121400 Sapur.005G015300 Sapur.006G026000 Sapur.006G026100 Sapur.007G063800 Sapur.007G064100 Sapur.007G064200 Sapur.009G042700 Sapur.010G023000 Sapur.010G042000 Sapur.012G080500 Sapur.017G046600 Sapur.017G047500 Sapur.15WG032000 Sapur.15ZG102400
GO:0036094	202	2832	small molecule binding	Sapur.001G017600 Sapur.001G040400 Sapur.001G055600 Sapur.001G058800 Sapur.001G078000 Sapur.001G078600 Sapur.001G081200 Sapur.001G103000 Sapur.001G103900 Sapur.001G122200 Sapur.001G126100 Sapur.002G002000 Sapur.002G005900 Sapur.002G020300 Sapur.002G051400 Sapur.002G085200 Sapur.002G089300 Sapur.002G093600 Sapur.002G106900 Sapur.002G120400 Sapur.002G152400 Sapur.002G171500 Sapur.002G182100 Sapur.002G188100 Sapur.003G020100 Sapur.003G051100 Sapur.003G061800 Sapur.003G073200 Sapur.003G079800 Sapur.003G092100 Sapur.003G099500 Sapur.003G099700 Sapur.003G139700 Sapur.004G019100 Sapur.004G029200 Sapur.004G032900 Sapur.004G051400 Sapur.004G059100 Sapur.004G067800 Sapur.004G071000 Sapur.004G089200 Sapur.004G103000 Sapur.004G109700 Sapur.004G120900 Sapur.004G122400 Sapur.004G139900 Sapur.004G147000 Sapur.004G170800 Sapur.005G001800 Sapur.005G013900 Sapur.005G015200 Sapur.005G022700 Sapur.005G024100 Sapur.005G025300 Sapur.005G027300 Sapur.005G029900 Sapur.005G050800 Sapur.005G093300 Sapur.005G109200 Sapur.005G206400 Sapur.006G029800 Sapur.006G036800 Sapur.006G036900 Sapur.006G067400 Sapur.006G069500 Sapur.006G096300 Sapur.006G101400 Sapur.006G107000 Sapur.006G111600 Sapur.006G112500 Sapur.006G159000 Sapur.006G172300 Sapur.006G188300 Sapur.006G194600 Sapur.006G195000 Sapur.006G195400 Sapur.006G201600 Sapur.007G005400 Sapur.007G012300 Sapur.007G016800 Sapur.007G024100 Sapur.007G042700 Sapur.007G091900 Sapur.007G127200 Sapur.008G004900 Sapur.008G005500 Sapur.008G016100 Sapur.008G067000 Sapur.008G074200 Sapur.008G113700 Sapur.008G128300 Sapur.008G129000 Sapur.008G137200 Sapur.008G143200 Sapur.008G157700 Sapur.009G018000 Sapur.009G018700 Sapur.009G038600

				Sapur.009G045200 Sapur.009G066000 Sapur.009G096500 Sapur.009G122200 Sapur.010G041500 Sapur.010G042700 Sapur.010G053300 Sapur.010G057900 Sapur.010G076000 Sapur.010G103300 Sapur.010G118800 Sapur.010G129400 Sapur.010G138600 Sapur.010G189000 Sapur.011G002200 Sapur.011G002300 Sapur.011G018500 Sapur.011G047800 Sapur.011G072400 Sapur.011G080600 Sapur.011G103500 Sapur.011G103700 Sapur.011G122100 Sapur.012G020400 Sapur.012G021700 Sapur.012G029700 Sapur.012G037400 Sapur.012G040700 Sapur.012G067300 Sapur.012G085700 Sapur.012G098500 Sapur.013G003400 Sapur.013G003500 Sapur.013G021800 Sapur.013G037700 Sapur.013G071200 Sapur.013G094000 Sapur.013G112200 Sapur.013G112400 Sapur.013G141900 Sapur.014G003800 Sapur.014G005100 Sapur.014G039100 Sapur.014G096700 Sapur.014G100600 Sapur.014G121100 Sapur.014G126200 Sapur.016G018800 Sapur.016G040000 Sapur.016G045300 Sapur.016G046200 Sapur.016G051800 Sapur.016G052600 Sapur.016G053300 Sapur.016G071200 Sapur.016G077400 Sapur.016G087900 Sapur.016G127100 Sapur.016G152100 Sapur.016G191700 Sapur.016G205200 Sapur.016G220800 Sapur.016G233300 Sapur.016G294500 Sapur.016G300800 Sapur.016G305500 Sapur.016G309400 Sapur.017G008100 Sapur.017G021900 Sapur.017G084100 Sapur.017G085100 Sapur.017G085800 Sapur.017G107900 Sapur.017G124700 Sapur.018G023200 Sapur.018G035500 Sapur.018G047100 Sapur.018G053200 Sapur.018G057600 Sapur.018G087600 Sapur.018G105800 Sapur.019G008900 Sapur.019G024300 Sapur.019G028700 Sapur.019G028900 Sapur.019G046400 Sapur.019G048400 Sapur.019G058700 Sapur.019G075600 Sapur.019G087800 Sapur.019G107300 Sapur.019G113000 Sapur.15WG028200 Sapur.15WG044400 Sapur.15ZG005000 Sapur.15ZG013600 Sapur.15ZG018000 Sapur.15ZG027900 Sapur.15ZG049500 Sapur.15ZG121300 Sapur.T003900 Sapur.T124800 Sapur.T138000 Sapur.T191300
GO:0043168	193	2695	anion binding	Sapur.001G040400 Sapur.001G055600 Sapur.001G058800 Sapur.001G078000 Sapur.001G078600 Sapur.001G081200 Sapur.001G103000 Sapur.001G103900 Sapur.001G122200 Sapur.001G126100 Sapur.002G002000 Sapur.002G005900 Sapur.002G020300 Sapur.002G051400 Sapur.002G085200 Sapur.002G089300 Sapur.002G093600 Sapur.002G106900 Sapur.002G120400 Sapur.002G152400 Sapur.002G171500 Sapur.002G182100 Sapur.002G188100 Sapur.003G020100 Sapur.003G051100 Sapur.003G061800 Sapur.003G073200 Sapur.003G079800 Sapur.003G092100 Sapur.003G099500 Sapur.003G099700 Sapur.003G139700 Sapur.004G019100 Sapur.004G029200 Sapur.004G032900 Sapur.004G051400 Sapur.004G059100 Sapur.004G067800 Sapur.004G089200 Sapur.004G103000 Sapur.004G109700 Sapur.004G120900 Sapur.004G122400 Sapur.004G147000 Sapur.004G170800 Sapur.005G001800 Sapur.005G013900 Sapur.005G015200 Sapur.005G022700 Sapur.005G024100 Sapur.005G025300 Sapur.005G027300 Sapur.005G029900 Sapur.005G050800 Sapur.005G093300 Sapur.005G109200 Sapur.005G206400 Sapur.006G029800 Sapur.006G036800 Sapur.006G036900 Sapur.006G067400 Sapur.006G069500 Sapur.006G096300 Sapur.006G101400 Sapur.006G107000 Sapur.006G111600 Sapur.006G112500 Sapur.006G159000 Sapur.006G172300 Sapur.006G188300 Sapur.006G194600 Sapur.006G195000 Sapur.006G201600 Sapur.007G005400 Sapur.007G012300 Sapur.007G016800 Sapur.007G024100 Sapur.007G091900 Sapur.007G127200 Sapur.008G004900 Sapur.008G005500 Sapur.008G016100 Sapur.008G067000 Sapur.008G113700 Sapur.008G128300 Sapur.008G137200 Sapur.008G143200 Sapur.008G157700 Sapur.009G018000 Sapur.009G018700 Sapur.009G038600 Sapur.009G045200 Sapur.009G066000 Sapur.009G096500 Sapur.009G122200 Sapur.010G041500 Sapur.010G042700 Sapur.010G053300 Sapur.010G057900 Sapur.010G076000 Sapur.010G103300 Sapur.010G118800 Sapur.010G129400 Sapur.010G138600 Sapur.010G189000 Sapur.011G002200 Sapur.011G002300 Sapur.011G018500 Sapur.011G047800 Sapur.011G072400 Sapur.011G080600 Sapur.011G103500 Sapur.011G103700 Sapur.011G122100 Sapur.012G020400 Sapur.012G021700 Sapur.012G029700 Sapur.012G037400 Sapur.012G040700 Sapur.012G067300 Sapur.012G085700 Sapur.012G098500 Sapur.013G003400 Sapur.013G003500 Sapur.013G021800 Sapur.013G071200 Sapur.013G094000 Sapur.013G112200 Sapur.013G112400 Sapur.013G141900 Sapur.014G003800 Sapur.014G005100 Sapur.014G039100 Sapur.014G096700 Sapur.014G100600 Sapur.014G121100 Sapur.014G126200 Sapur.016G018800 Sapur.016G045300 Sapur.016G046200 Sapur.016G051800 Sapur.016G052600 Sapur.016G053300 Sapur.016G071200 Sapur.016G077400 Sapur.016G087900 Sapur.016G127100 Sapur.016G152100 Sapur.016G191700 Sapur.016G205200 Sapur.016G220800 Sapur.016G233300 Sapur.016G294500 Sapur.016G300800 Sapur.016G305500 Sapur.016G309400 Sapur.017G008100 Sapur.017G021900 Sapur.017G084100 Sapur.017G085100 Sapur.017G085800 Sapur.017G107900 Sapur.017G124700 Sapur.018G023200 Sapur.018G035500 Sapur.018G047100 Sapur.018G053200 Sapur.018G057600

				Sapur.018G087600 Sapur.018G105800 Sapur.019G008900 Sapur.019G024300 Sapur.019G028700 Sapur.019G028900 Sapur.019G046400 Sapur.019G048400 Sapur.019G058700 Sapur.019G075600 Sapur.019G087800 Sapur.019G107300 Sapur.019G113000 Sapur.15WG028200 Sapur.15WG044400 Sapur.15ZG005000 Sapur.15ZG013600 Sapur.15ZG018000 Sapur.15ZG027900 Sapur.15ZG049500 Sapur.15ZG121300 Sapur.T003900 Sapur.T124800 Sapur.T138000 Sapur.T191300
GO:0004672	121	1565	protein kinase activity	Sapur.001G038800 Sapur.001G078000 Sapur.001G096300 Sapur.001G103900 Sapur.001G126100 Sapur.002G002000 Sapur.002G005900 Sapur.002G020300 Sapur.002G051400 Sapur.002G060000 Sapur.002G106900 Sapur.002G120400 Sapur.002G188100 Sapur.002G195600 Sapur.003G037300 Sapur.003G051100 Sapur.003G056800 Sapur.003G061800 Sapur.003G073200 Sapur.003G079800 Sapur.003G099700 Sapur.003G139700 Sapur.004G029200 Sapur.004G032900 Sapur.004G059100 Sapur.004G067800 Sapur.004G170800 Sapur.005G001800 Sapur.005G027300 Sapur.005G050800 Sapur.005G109200 Sapur.005G188000 Sapur.005G206400 Sapur.006G040500 Sapur.006G044000 Sapur.006G065500 Sapur.006G077000 Sapur.006G085200 Sapur.006G090100 Sapur.006G092500 Sapur.006G093700 Sapur.006G096300 Sapur.006G107000 Sapur.006G188300 Sapur.006G194600 Sapur.006G195000 Sapur.007G012300 Sapur.007G045900 Sapur.007G099800 Sapur.007G127200 Sapur.008G016100 Sapur.008G054500 Sapur.008G113700 Sapur.008G143200 Sapur.008G157700 Sapur.009G130800 Sapur.010G028600 Sapur.010G041500 Sapur.010G057900 Sapur.010G076000 Sapur.010G143200 Sapur.010G147600 Sapur.010G171000 Sapur.010G189000 Sapur.011G002200 Sapur.011G002300 Sapur.011G024200 Sapur.011G046900 Sapur.011G047800 Sapur.011G072400 Sapur.011G080600 Sapur.011G103500 Sapur.012G020400 Sapur.012G029700 Sapur.012G048500 Sapur.012G051500 Sapur.012G052000 Sapur.012G067300 Sapur.012G097700 Sapur.012G098500 Sapur.013G003400 Sapur.013G020900 Sapur.013G028400 Sapur.013G134300 Sapur.013G141900 Sapur.014G063400 Sapur.014G106100 Sapur.014G126200 Sapur.016G045300 Sapur.016G046200 Sapur.016G049500 Sapur.016G109100 Sapur.016G191700 Sapur.016G220500 Sapur.016G233300 Sapur.016G263500 Sapur.016G274100 Sapur.016G294500 Sapur.017G021900 Sapur.017G107900 Sapur.018G018700 Sapur.018G023200 Sapur.018G035500 Sapur.018G053200 Sapur.018G082400 Sapur.019G008900 Sapur.019G048400 Sapur.019G058700 Sapur.019G061700 Sapur.019G107300 Sapur.019G113000 Sapur.15WG028200 Sapur.15WG063200 Sapur.15WG079300 Sapur.15ZG005000 Sapur.15ZG013600 Sapur.15ZG018000 Sapur.15ZG027900 Sapur.15ZG068100 Sapur.15ZG121300 Sapur.T191300
GO:0003924	12	67	GTPase activity	Sapur.001G078600 Sapur.001G086500 Sapur.001G122200 Sapur.003G090100 Sapur.003G091100 Sapur.006G025400 Sapur.009G029500 Sapur.009G052200 Sapur.011G119600 Sapur.016G138000 Sapur.016G172500 Sapur.017G067300
GO:0017171	20	153	serine hydrolase activity	Sapur.001G088500 Sapur.002G013000 Sapur.002G097300 Sapur.002G101500 Sapur.003G088100 Sapur.004G164900 Sapur.006G061500 Sapur.006G169900 Sapur.007G042300 Sapur.016G133500 Sapur.016G285600 Sapur.018G084700 Sapur.001G051400 Sapur.002G056400 Sapur.003G121400 Sapur.006G026000 Sapur.006G026100 Sapur.009G042700 Sapur.012G080500 Sapur.15ZG102400
GO:0008236	20	153	serine-type peptidase activity	Sapur.001G088500 Sapur.002G013000 Sapur.002G097300 Sapur.002G101500 Sapur.003G088100 Sapur.004G164900 Sapur.006G061500 Sapur.006G169900 Sapur.007G042300 Sapur.016G133500 Sapur.016G285600 Sapur.018G084700 Sapur.001G051400 Sapur.002G056400 Sapur.003G121400 Sapur.006G026000 Sapur.006G026100 Sapur.009G042700 Sapur.012G080500 Sapur.15ZG102400
GO:0016301	123	1669	kinase activity	Sapur.001G038800 Sapur.001G078000 Sapur.001G096300 Sapur.001G103900 Sapur.001G126100 Sapur.002G002000 Sapur.002G005900 Sapur.002G020300 Sapur.002G051400 Sapur.002G060000 Sapur.002G106900 Sapur.002G120400 Sapur.002G188100 Sapur.002G195600 Sapur.003G037300 Sapur.003G051100 Sapur.003G056800 Sapur.003G061800 Sapur.003G073200 Sapur.003G079800 Sapur.003G099700 Sapur.003G139700 Sapur.004G029200 Sapur.004G032900 Sapur.004G059100 Sapur.004G067800 Sapur.004G170800 Sapur.005G001800 Sapur.005G027300 Sapur.005G050800 Sapur.005G109200 Sapur.005G188000 Sapur.005G206400 Sapur.006G040500 Sapur.006G044000 Sapur.006G065500 Sapur.006G077000 Sapur.006G085200 Sapur.006G090100 Sapur.006G092500 Sapur.006G093700 Sapur.006G096300 Sapur.006G107000 Sapur.006G188300 Sapur.006G194600 Sapur.006G195000 Sapur.007G012300 Sapur.007G045900 Sapur.007G099800 Sapur.007G127200 Sapur.008G016100 Sapur.008G054500 Sapur.008G113700 Sapur.008G143200 Sapur.008G157700 Sapur.009G130800 Sapur.010G028600 Sapur.010G041500 Sapur.010G057900 Sapur.010G076000 Sapur.010G143200 Sapur.010G147600 Sapur.010G171000

				Sapur.010G189000 Sapur.011G002200 Sapur.011G002300 Sapur.011G024200 Sapur.011G046900 Sapur.011G047800 Sapur.011G072400 Sapur.011G080600 Sapur.011G103500 Sapur.012G020400 Sapur.012G029700 Sapur.012G048500 Sapur.012G051500 Sapur.012G052000 Sapur.012G067300 Sapur.012G097700 Sapur.012G098500 Sapur.013G003400 Sapur.013G020900 Sapur.013G028400 Sapur.013G134300 Sapur.013G141900 Sapur.014G063400 Sapur.014G106100 Sapur.014G126200 Sapur.016G045300 Sapur.016G046200 Sapur.016G049500 Sapur.016G109100 Sapur.016G191700 Sapur.016G220500 Sapur.016G233300 Sapur.016G263500 Sapur.016G274100 Sapur.016G294500 Sapur.017G021900 Sapur.017G107900 Sapur.018G018700 Sapur.018G023200 Sapur.018G035500 Sapur.018G053200 Sapur.018G082400 Sapur.019G008900 Sapur.019G048400 Sapur.019G058700 Sapur.019G061700 Sapur.019G107300 Sapur.019G113000 Sapur.15WG028200 Sapur.15WG063200 Sapur.15WG079300 Sapur.15ZG005000 Sapur.15ZG013600 Sapur.15ZG018000 Sapur.15ZG027900 Sapur.15ZG068100 Sapur.15ZG121300 Sapur.T191300 Sapur.002G171500 Sapur.018G073200
GO:0016773	123	1675	phosphotransferase activity, alcohol group as acceptor	Sapur.001G038800 Sapur.001G078000 Sapur.001G096300 Sapur.001G103900 Sapur.001G126100 Sapur.002G002000 Sapur.002G005900 Sapur.002G020300 Sapur.002G051400 Sapur.002G060000 Sapur.002G106900 Sapur.002G120400 Sapur.002G188100 Sapur.002G195600 Sapur.003G037300 Sapur.003G051100 Sapur.003G056800 Sapur.003G061800 Sapur.003G073200 Sapur.003G079800 Sapur.003G099700 Sapur.003G139700 Sapur.004G029200 Sapur.004G032900 Sapur.004G059100 Sapur.004G067800 Sapur.004G170800 Sapur.005G001800 Sapur.005G027300 Sapur.005G050800 Sapur.005G109200 Sapur.005G188000 Sapur.005G206400 Sapur.006G040500 Sapur.006G044000 Sapur.006G065500 Sapur.006G077000 Sapur.006G085200 Sapur.006G090100 Sapur.006G092500 Sapur.006G093700 Sapur.006G096300 Sapur.006G107000 Sapur.006G188300 Sapur.006G194600 Sapur.006G195000 Sapur.007G012300 Sapur.007G045900 Sapur.007G099800 Sapur.007G127200 Sapur.008G016100 Sapur.008G054500 Sapur.008G113700 Sapur.008G143200 Sapur.008G157700 Sapur.009G130800 Sapur.010G028600 Sapur.010G041500 Sapur.010G057900 Sapur.010G076000 Sapur.010G143200 Sapur.010G147600 Sapur.010G171000 Sapur.010G189000 Sapur.011G002200 Sapur.011G002300 Sapur.011G024200 Sapur.011G046900 Sapur.011G047800 Sapur.011G072400 Sapur.011G080600 Sapur.011G103500 Sapur.012G020400 Sapur.012G029700 Sapur.012G048500 Sapur.012G051500 Sapur.012G052000 Sapur.012G067300 Sapur.012G097700 Sapur.012G098500 Sapur.013G003400 Sapur.013G020900 Sapur.013G028400 Sapur.013G134300 Sapur.013G141900 Sapur.014G063400 Sapur.014G106100 Sapur.014G126200 Sapur.016G045300 Sapur.016G046200 Sapur.016G049500 Sapur.016G109100 Sapur.016G191700 Sapur.016G220500 Sapur.016G233300 Sapur.016G263500 Sapur.016G274100 Sapur.016G294500 Sapur.017G021900 Sapur.017G107900 Sapur.018G018700 Sapur.018G023200 Sapur.018G035500 Sapur.018G053200 Sapur.018G082400 Sapur.019G008900 Sapur.019G048400 Sapur.019G058700 Sapur.019G061700 Sapur.019G107300 Sapur.019G113000 Sapur.15WG028200 Sapur.15WG063200 Sapur.15WG079300 Sapur.15ZG005000 Sapur.15ZG013600 Sapur.15ZG018000 Sapur.15ZG027900 Sapur.15ZG068100 Sapur.15ZG121300 Sapur.T191300 Sapur.009G066500 Sapur.018G073200
GO:0004185	8	42	serine-type carboxypeptidase activity	Sapur.001G051400 Sapur.002G056400 Sapur.003G121400 Sapur.006G026000 Sapur.006G026100 Sapur.009G042700 Sapur.012G080500 Sapur.15ZG102400
GO:0004180	8	44	carboxypeptidase activity	Sapur.001G051400 Sapur.002G056400 Sapur.003G121400 Sapur.006G026000 Sapur.006G026100 Sapur.009G042700 Sapur.012G080500 Sapur.15ZG102400
GO:0003916	4	13	DNA topoisomerase activity	Sapur.005G005600 Sapur.016G193800 Sapur.009G045200 Sapur.016G152100
GO:0008289	13	102	lipid binding	Sapur.001G162700 Sapur.003G027100 Sapur.006G196300 Sapur.008G028600 Sapur.009G007100 Sapur.009G007800 Sapur.011G067900 Sapur.016G237500 Sapur.018G035400 Sapur.005G059100 Sapur.006G054200 Sapur.006G061100 Sapur.019G064900
GO:0070008	8	48	serine-type exopeptidase activity	Sapur.001G051400 Sapur.002G056400 Sapur.003G121400 Sapur.006G026000 Sapur.006G026100 Sapur.009G042700 Sapur.012G080500 Sapur.15ZG102400
GO:0003	101	1390	DNA binding	Sapur.001G014000 Sapur.001G055600 Sapur.001G058800 Sapur.001G162700 Sapur.002G018600 Sapur.002G021500 Sapur.002G035300

677				Sapur.002G101000 Sapur.002G105900 Sapur.002G138900 Sapur.002G160400 Sapur.003G027100 Sapur.003G078200 Sapur.003G126500 Sapur.003G159300 Sapur.003G159400 Sapur.004G000200 Sapur.004G018900 Sapur.004G089200 Sapur.004G103000 Sapur.004G129100 Sapur.004G139900 Sapur.005G005600 Sapur.005G019900 Sapur.005G032200 Sapur.005G081300 Sapur.005G093200 Sapur.005G094500 Sapur.005G104600 Sapur.005G186700 Sapur.005G189800 Sapur.005G202600 Sapur.006G026800 Sapur.006G037000 Sapur.006G157800 Sapur.006G196300 Sapur.007G024100 Sapur.007G042700 Sapur.007G091900 Sapur.007G123900 Sapur.008G021500 Sapur.008G048500 Sapur.009G007100 Sapur.009G007800 Sapur.009G016800 Sapur.009G045200 Sapur.009G080800 Sapur.009G080900 Sapur.009G096500 Sapur.009G112800 Sapur.010G053800 Sapur.010G057800 Sapur.010G082600 Sapur.010G184700 Sapur.010G185200 Sapur.010G193700 Sapur.011G056100 Sapur.011G067900 Sapur.012G025800 Sapur.012G085700 Sapur.013G018500 Sapur.013G028900 Sapur.013G029000 Sapur.014G015000 Sapur.014G026400 Sapur.014G075000 Sapur.014G075400 Sapur.014G096700 Sapur.016G116500 Sapur.016G152100 Sapur.016G193800 Sapur.016G216900 Sapur.016G237500 Sapur.018G035400 Sapur.018G087600 Sapur.018G107600 Sapur.019G024300 Sapur.019G094800 Sapur.15WG072800 Sapur.15ZG023500 Sapur.15ZG043500 Sapur.T006500 Sapur.002G100300 Sapur.002G101800 Sapur.002G175800 Sapur.004G171400 Sapur.005G068200 Sapur.005G195200 Sapur.006G111600 Sapur.006G217600 Sapur.007G074000 Sapur.008G072300 Sapur.008G128300 Sapur.013G113000 Sapur.014G014000 Sapur.014G016400 Sapur.014G036800 Sapur.014G070100 Sapur.016G239300 Sapur.019G112000 Sapur.T189500
GO:0003887	4	14	DNA-directed DNA polymerase activity	Sapur.004G139900 Sapur.007G042700 Sapur.010G053800 Sapur.012G025800
GO:0016787	131	1881	hydrolase activity	Sapur.001G023400 Sapur.001G078600 Sapur.001G086500 Sapur.001G088500 Sapur.001G098400 Sapur.001G122200 Sapur.001G138800 Sapur.002G013000 Sapur.002G074000 Sapur.002G097300 Sapur.002G101500 Sapur.002G143900 Sapur.003G088100 Sapur.003G090100 Sapur.003G091100 Sapur.003G099500 Sapur.004G060000 Sapur.004G164900 Sapur.005G161200 Sapur.006G025400 Sapur.006G061500 Sapur.006G169900 Sapur.007G042300 Sapur.008G093300 Sapur.009G029500 Sapur.009G038600 Sapur.009G052200 Sapur.010G098600 Sapur.011G119600 Sapur.014G043900 Sapur.016G087900 Sapur.016G133500 Sapur.016G138000 Sapur.016G172500 Sapur.016G285600 Sapur.017G067300 Sapur.018G084700 Sapur.019G065500 Sapur.001G051400 Sapur.001G112100 Sapur.001G167900 Sapur.001G171300 Sapur.001G191900 Sapur.002G004700 Sapur.002G048300 Sapur.002G056400 Sapur.002G166700 Sapur.002G182500 Sapur.002G192300 Sapur.002G200900 Sapur.003G013400 Sapur.003G040900 Sapur.003G118000 Sapur.003G121400 Sapur.003G151000 Sapur.003G165600 Sapur.004G061300 Sapur.004G089200 Sapur.004G139900 Sapur.004G158400 Sapur.005G000700 Sapur.005G015300 Sapur.005G030000 Sapur.005G190700 Sapur.006G026000 Sapur.006G026100 Sapur.006G114600 Sapur.006G131700 Sapur.006G180700 Sapur.007G024100 Sapur.007G042700 Sapur.007G051800 Sapur.007G063800 Sapur.007G064100 Sapur.007G064200 Sapur.007G089900 Sapur.007G091900 Sapur.008G043800 Sapur.008G053700 Sapur.008G062900 Sapur.008G102200 Sapur.008G107800 Sapur.008G112200 Sapur.009G042700 Sapur.009G059800 Sapur.009G120900 Sapur.010G023000 Sapur.010G042000 Sapur.010G071500 Sapur.010G081700 Sapur.010G094400 Sapur.010G148600 Sapur.011G072100 Sapur.011G112600 Sapur.012G080500 Sapur.012G105600 Sapur.013G000800 Sapur.013G135900 Sapur.014G071900 Sapur.014G072000 Sapur.014G091800 Sapur.014G097700 Sapur.014G101600 Sapur.014G129200 Sapur.016G140600 Sapur.016G142200 Sapur.016G265200 Sapur.017G044200 Sapur.017G046600 Sapur.017G047500 Sapur.017G108600 Sapur.017G112500 Sapur.018G070800 Sapur.019G037200 Sapur.019G068900 Sapur.019G109000 Sapur.15WG032000 Sapur.15WG032300 Sapur.15WG032400 Sapur.15ZG036100 Sapur.15ZG036200 Sapur.15ZG081500 Sapur.15ZG102400 Sapur.15ZG131000 Sapur.15ZG133000 Sapur.T171300 Sapur.002G108700 Sapur.009G076200 Sapur.014G031500 Sapur.003G077800 Sapur.15ZG117900
GO:0034061	4	15	DNA polymerase activity	Sapur.004G139900 Sapur.007G042700 Sapur.010G053800 Sapur.012G025800
GO:0008158	2	3	hedgehog receptor activity	Sapur.002G006100 Sapur.005G201800

GO:0015926	2	3	glucosidase activity	Sapur.003G077800 Sapur.15ZG117900
GO:0090599	2	3	alpha-glucosidase activity	Sapur.003G077800 Sapur.15ZG117900
GO:0004564	2	3	beta-fructofuranosidase activity	Sapur.003G077800 Sapur.15ZG117900
GO:0004575	2	3	sucrose alpha-glucosidase activity	Sapur.003G077800 Sapur.15ZG117900
GO:0015035	12	98	protein-disulfide reductase activity	Sapur.006G164600 Sapur.006G173800 Sapur.007G121300 Sapur.008G010200 Sapur.008G116700 Sapur.010G197200 Sapur.014G107200 Sapur.014G107800 Sapur.016G062700 Sapur.016G073300 Sapur.016G200600 Sapur.016G288700
GO:0015036	12	101	disulfide oxidoreductase activity	Sapur.006G164600 Sapur.006G173800 Sapur.007G121300 Sapur.008G010200 Sapur.008G116700 Sapur.010G197200 Sapur.014G107200 Sapur.014G107800 Sapur.016G062700 Sapur.016G073300 Sapur.016G200600 Sapur.016G288700
GO:0016772	130	1899	transferase activity, transferring phosphorus-containing groups	Sapur.001G038800 Sapur.001G078000 Sapur.001G096300 Sapur.001G103900 Sapur.001G126100 Sapur.002G002000 Sapur.002G005900 Sapur.002G020300 Sapur.002G051400 Sapur.002G060000 Sapur.002G106900 Sapur.002G120400 Sapur.002G188100 Sapur.002G195600 Sapur.003G037300 Sapur.003G051100 Sapur.003G056800 Sapur.003G061800 Sapur.003G073200 Sapur.003G079800 Sapur.003G099700 Sapur.003G139700 Sapur.004G029200 Sapur.004G032900 Sapur.004G059100 Sapur.004G067800 Sapur.004G170800 Sapur.005G001800 Sapur.005G027300 Sapur.005G050800 Sapur.005G109200 Sapur.005G188000 Sapur.005G206400 Sapur.006G040500 Sapur.006G044000 Sapur.006G065500 Sapur.006G077000 Sapur.006G085200 Sapur.006G090100 Sapur.006G092500 Sapur.006G093700 Sapur.006G096300 Sapur.006G107000 Sapur.006G188300 Sapur.006G194600 Sapur.006G195000 Sapur.007G012300 Sapur.007G045900 Sapur.007G099800 Sapur.007G127200 Sapur.008G016100 Sapur.008G054500 Sapur.008G113700 Sapur.008G143200 Sapur.008G157700 Sapur.009G130800 Sapur.010G028600 Sapur.010G041500 Sapur.010G057900 Sapur.010G076000 Sapur.010G143200 Sapur.010G147600 Sapur.010G171000 Sapur.010G189000 Sapur.011G002200 Sapur.011G002300 Sapur.011G024200 Sapur.011G046900 Sapur.011G047800 Sapur.011G072400 Sapur.011G080600 Sapur.011G103500 Sapur.012G020400 Sapur.012G029700 Sapur.012G048500 Sapur.012G051500 Sapur.012G052000 Sapur.012G067300 Sapur.012G097700 Sapur.012G098500 Sapur.013G003400 Sapur.013G020900 Sapur.013G028400 Sapur.013G134300 Sapur.013G141900 Sapur.014G063400 Sapur.014G106100 Sapur.014G126200 Sapur.016G045300 Sapur.016G046200 Sapur.016G049500 Sapur.016G109100 Sapur.016G191700 Sapur.016G220500 Sapur.016G233300 Sapur.016G263500 Sapur.016G274100 Sapur.016G294500 Sapur.017G021900 Sapur.017G107900 Sapur.018G018700 Sapur.018G023200 Sapur.018G035500 Sapur.018G053200 Sapur.018G082400 Sapur.019G008900 Sapur.019G048400 Sapur.019G058700 Sapur.019G061700 Sapur.019G107300 Sapur.019G113000 Sapur.15WG028200 Sapur.15WG063200 Sapur.15WG079300 Sapur.15ZG005000 Sapur.15ZG013600 Sapur.15ZG018000 Sapur.15ZG027900 Sapur.15ZG068100 Sapur.15ZG121300 Sapur.T191300 Sapur.002G171500 Sapur.003G070500 Sapur.003G126100 Sapur.004G139900 Sapur.007G042700 Sapur.009G066500 Sapur.010G053800 Sapur.012G025800 Sapur.018G073200
GO:0008238	8	58	exopeptidase activity	Sapur.001G051400 Sapur.002G056400 Sapur.003G121400 Sapur.006G026000 Sapur.006G026100 Sapur.009G042700 Sapur.012G080500 Sapur.15ZG102400
GO:0015098	2	5	molybdate ion transmembrane transporter activity	Sapur.003G108000 Sapur.15ZG006400
GO:0003	2	5	DNA primase	Sapur.003G070500 Sapur.003G126100

	896			activity	
	GO:0005092	2	5	GDP-dissociation inhibitor activity	Sapur.006G008400 Sapur.016G011100
	GO:0005094	2	5	Rho GDP-dissociation inhibitor activity	Sapur.006G008400 Sapur.016G011100
	GO:0140097	13	129	catalytic activity, acting on DNA	Sapur.001G055600 Sapur.001G058800 Sapur.004G139900 Sapur.005G005600 Sapur.007G042700 Sapur.010G053800 Sapur.012G025800 Sapur.014G096700 Sapur.016G193800 Sapur.009G045200 Sapur.016G152100 Sapur.002G108700 Sapur.014G031500
	GO:0004478	2	6	methionine adenosyltransferase activity	Sapur.006G101400 Sapur.013G003500
	GO:0004014	2	6	adenosylmethionine decarboxylase activity	Sapur.008G171500 Sapur.010G013500
	GO:0030246	17	189	carbohydrate binding	Sapur.001G167900 Sapur.003G061800 Sapur.004G029200 Sapur.006G107000 Sapur.006G114600 Sapur.007G089900 Sapur.011G072400 Sapur.016G094700 Sapur.016G233300 Sapur.016G294500 Sapur.002G060000 Sapur.008G075700 Sapur.008G078700 Sapur.010G119000 Sapur.011G006700 Sapur.013G134300 Sapur.017G021900
	GO:0016667	12	122	oxidoreductase activity, acting on a sulfur group of donors	Sapur.006G164600 Sapur.006G173800 Sapur.007G121300 Sapur.008G010200 Sapur.008G116700 Sapur.010G197200 Sapur.014G107200 Sapur.014G107800 Sapur.016G062700 Sapur.016G073300 Sapur.016G200600 Sapur.016G288700
	GO:0046906	25	454	tetrapyrrole binding	Sapur.001G067900 Sapur.001G097100 Sapur.001G097300 Sapur.001G193000 Sapur.002G099300 Sapur.002G180700 Sapur.003G093200 Sapur.004G088800 Sapur.005G153900 Sapur.006G118800 Sapur.006G130500 Sapur.006G140500 Sapur.007G047300 Sapur.007G049700 Sapur.009G079400 Sapur.010G088700 Sapur.010G088800 Sapur.010G088900 Sapur.011G097000 Sapur.014G011100 Sapur.014G028500 Sapur.016G007400 Sapur.016G055400 Sapur.018G105100 Sapur.019G065400
	GO:0020037	25	454	heme binding	Sapur.001G067900 Sapur.001G097100 Sapur.001G097300 Sapur.001G193000 Sapur.002G099300 Sapur.002G180700 Sapur.003G093200 Sapur.004G088800 Sapur.005G153900 Sapur.006G118800 Sapur.006G130500 Sapur.006G140500 Sapur.007G047300 Sapur.007G049700 Sapur.009G079400 Sapur.010G088700 Sapur.010G088800 Sapur.010G088900 Sapur.011G097000 Sapur.014G011100 Sapur.014G028500 Sapur.016G007400 Sapur.016G055400 Sapur.018G105100 Sapur.019G065400
**r_up_MF**	GO:0016491	54	1583	oxidoreductase activity	Sapur.001G017900 Sapur.001G055900 Sapur.002G071000 Sapur.002G128000 Sapur.003G015300 Sapur.005G134500 Sapur.005G153900 Sapur.006G130500 Sapur.007G049700 Sapur.008G117200 Sapur.008G131500 Sapur.009G044500 Sapur.009G079400 Sapur.011G077400 Sapur.011G116000 Sapur.012G103800 Sapur.013G097200 Sapur.013G097700 Sapur.016G055400 Sapur.016G056100 Sapur.016G155100 Sapur.016G251200 Sapur.016G300700 Sapur.019G011100 Sapur.15WG080500 Sapur.15ZG083800 Sapur.T051400 Sapur.002G094800 Sapur.004G112400 Sapur.008G127300 Sapur.012G093900 Sapur.016G117300 Sapur.T007600 Sapur.013G142900 Sapur.001G067900 Sapur.001G097100 Sapur.001G097300 Sapur.001G193000 Sapur.002G099300 Sapur.002G180700 Sapur.003G093200 Sapur.004G088800 Sapur.006G118800 Sapur.006G140500 Sapur.007G047300 Sapur.010G088700 Sapur.010G088800 Sapur.010G088900 Sapur.011G097000 Sapur.014G011100 Sapur.014G028500 Sapur.016G007400 Sapur.018G105100 Sapur.019G065400
	GO:0005506	22	373	iron ion binding	Sapur.001G067900 Sapur.001G097100 Sapur.001G097300 Sapur.001G193000 Sapur.002G099300 Sapur.002G180700 Sapur.003G093200 Sapur.004G088800 Sapur.006G118800 Sapur.006G140500 Sapur.007G047300 Sapur.010G088700 Sapur.010G088800 Sapur.010G088900

				Sapur.011G097000 Sapur.014G011100 Sapur.014G028500 Sapur.016G007400 Sapur.018G105100 Sapur.019G065400 Sapur.006G084800 Sapur.016G107800
GO:0016705	20	354	oxidoreductase activity, acting on paired donors, with incorporation or reduction of molecular oxygen	Sapur.001G067900 Sapur.001G097100 Sapur.001G097300 Sapur.001G193000 Sapur.002G099300 Sapur.002G180700 Sapur.003G093200 Sapur.004G088800 Sapur.006G118800 Sapur.006G140500 Sapur.007G047300 Sapur.010G088700 Sapur.010G088800 Sapur.010G088900 Sapur.011G097000 Sapur.014G011100 Sapur.014G028500 Sapur.016G007400 Sapur.018G105100 Sapur.019G065400
GO:0043169	56	1941	cation binding	Sapur.001G067900 Sapur.001G097100 Sapur.001G097300 Sapur.001G193000 Sapur.002G009700 Sapur.002G099300 Sapur.002G103300 Sapur.002G180700 Sapur.003G056600 Sapur.003G093200 Sapur.004G051000 Sapur.004G088800 Sapur.006G014400 Sapur.006G069400 Sapur.006G118800 Sapur.006G140500 Sapur.006G174900 Sapur.007G047300 Sapur.009G022800 Sapur.010G088700 Sapur.010G088800 Sapur.010G088900 Sapur.011G097000 Sapur.014G011100 Sapur.014G019000 Sapur.014G019100 Sapur.014G019200 Sapur.014G020400 Sapur.014G028500 Sapur.014G035400 Sapur.016G007400 Sapur.018G105100 Sapur.019G065400 Sapur.15ZG089200 Sapur.15ZG126800 Sapur.001G009100 Sapur.002G071000 Sapur.004G037400 Sapur.005G134500 Sapur.006G084800 Sapur.007G050900 Sapur.008G131500 Sapur.011G071900 Sapur.012G067900 Sapur.012G103800 Sapur.016G107800 Sapur.016G155100 Sapur.016G187100 Sapur.016G251200 Sapur.018G072600 Sapur.019G011100 Sapur.019G015000 Sapur.019G020800 Sapur.019G024100 Sapur.T051400 Sapur.002G081000
GO:0046872	55	1929	metal ion binding	Sapur.001G067900 Sapur.001G097100 Sapur.001G097300 Sapur.001G193000 Sapur.002G009700 Sapur.002G099300 Sapur.002G103300 Sapur.002G180700 Sapur.003G056600 Sapur.003G093200 Sapur.004G051000 Sapur.004G088800 Sapur.006G014400 Sapur.006G069400 Sapur.006G118800 Sapur.006G140500 Sapur.006G174900 Sapur.007G047300 Sapur.009G022800 Sapur.010G088700 Sapur.010G088800 Sapur.010G088900 Sapur.011G097000 Sapur.014G011100 Sapur.014G019000 Sapur.014G019100 Sapur.014G019200 Sapur.014G020400 Sapur.014G028500 Sapur.014G035400 Sapur.016G007400 Sapur.018G105100 Sapur.019G065400 Sapur.15ZG089200 Sapur.15ZG126800 Sapur.001G009100 Sapur.002G071000 Sapur.004G037400 Sapur.005G134500 Sapur.006G084800 Sapur.007G050900 Sapur.008G131500 Sapur.011G071900 Sapur.012G067900 Sapur.012G103800 Sapur.016G107800 Sapur.016G155100 Sapur.016G187100 Sapur.016G251200 Sapur.018G072600 Sapur.019G011100 Sapur.019G015000 Sapur.019G020800 Sapur.019G024100 Sapur.T051400
GO:0003824	148	7297	catalytic activity	Sapur.001G009100 Sapur.001G017900 Sapur.001G055900 Sapur.001G189600 Sapur.001G190100 Sapur.001G190200 Sapur.002G071000 Sapur.002G085900 Sapur.002G128000 Sapur.003G015300 Sapur.003G087600 Sapur.004G044500 Sapur.004G044600 Sapur.005G099400 Sapur.005G122300 Sapur.005G134500 Sapur.005G153900 Sapur.006G070200 Sapur.006G098600 Sapur.006G130500 Sapur.007G049700 Sapur.007G050900 Sapur.007G087100 Sapur.008G117200 Sapur.008G131500 Sapur.009G044500 Sapur.009G079400 Sapur.010G054800 Sapur.011G025600 Sapur.011G077400 Sapur.011G095300 Sapur.011G116000 Sapur.012G020600 Sapur.012G067900 Sapur.012G101300 Sapur.012G103800 Sapur.013G047200 Sapur.013G097200 Sapur.013G097700 Sapur.014G090200 Sapur.016G048100 Sapur.016G055400 Sapur.016G056100 Sapur.016G141300 Sapur.016G155100 Sapur.016G186000 Sapur.016G251200 Sapur.016G291000 Sapur.016G300700 Sapur.017G057900 Sapur.019G011100 Sapur.019G088200 Sapur.019G088400 Sapur.019G111400 Sapur.15WG080500 Sapur.15ZG075400 Sapur.15ZG083800 Sapur.T017700 Sapur.T026400 Sapur.T051400 Sapur.002G009700 Sapur.002G081000 Sapur.002G094800 Sapur.003G056600 Sapur.003G117900 Sapur.004G112400 Sapur.005G139100 Sapur.006G105800 Sapur.006G192200 Sapur.007G006500 Sapur.008G055700 Sapur.008G127300 Sapur.009G003500 Sapur.009G096200 Sapur.009G098500 Sapur.010G026500 Sapur.010G028300 Sapur.010G182700 Sapur.011G060600 Sapur.011G114200 Sapur.012G073400 Sapur.012G093900 Sapur.016G054600 Sapur.016G084000 Sapur.016G117300 Sapur.016G163100 Sapur.016G304800 Sapur.017G102000 Sapur.15WG035700 Sapur.15WG076200 Sapur.15ZG013900 Sapur.15ZG110400 Sapur.15ZG132700 Sapur.T007600 Sapur.T169500 Sapur.T175700 Sapur.001G076400 Sapur.002G121000 Sapur.002G165800 Sapur.002G182700 Sapur.004G037400 Sapur.004G082800 Sapur.006G005600 Sapur.006G018000 Sapur.006G228200 Sapur.006G228300 Sapur.007G026800

				Sapur.007G077700 Sapur.007G125300 Sapur.008G051700 Sapur.008G085700 Sapur.008G112400 Sapur.009G086600 Sapur.010G150800 Sapur.013G094800 Sapur.013G094900 Sapur.013G095000 Sapur.013G095100 Sapur.013G110500 Sapur.013G142900 Sapur.016G020100 Sapur.016G151100 Sapur.016G189700 Sapur.016G270700 Sapur.018G076400 Sapur.019G015000 Sapur.019G024100 Sapur.T073800
				Sapur.001G067900 Sapur.001G097100 Sapur.001G097300 Sapur.001G193000 Sapur.002G099300 Sapur.002G180700 Sapur.003G093200 Sapur.004G088800 Sapur.006G118800 Sapur.006G140500 Sapur.007G047300 Sapur.010G088700 Sapur.010G088800 Sapur.010G088900 Sapur.011G097000 Sapur.014G011100 Sapur.014G028500 Sapur.016G007400 Sapur.018G105100 Sapur.019G065400
GO:0140110	22	619	transcription regulator activity	Sapur.001G009100 Sapur.002G021100 Sapur.002G030100 Sapur.002G036700 Sapur.002G140800 Sapur.004G118800 Sapur.007G050900 Sapur.007G102800 Sapur.008G077700 Sapur.008G134800 Sapur.010G005200 Sapur.010G065900 Sapur.010G085300 Sapur.010G120100 Sapur.011G042400 Sapur.012G067900 Sapur.012G076500 Sapur.014G014900 Sapur.014G056000 Sapur.016G202700 Sapur.018G057700 Sapur.15ZG067200
GO:0061733	3	14	peptide-lysine-N-acetyltransferase activity	Sapur.001G009100 Sapur.007G050900 Sapur.012G067900
GO:0034212	3	14	peptide N-acetyltransferase activity	Sapur.001G009100 Sapur.007G050900 Sapur.012G067900
GO:0140993	3	14	histone modifying activity	Sapur.001G009100 Sapur.007G050900 Sapur.012G067900
GO:0004402	3	14	histone acetyltransferase activity	Sapur.001G009100 Sapur.007G050900 Sapur.012G067900
GO:0008171	4	34	O-methyltransferase activity	Sapur.006G098600 Sapur.016G291000 Sapur.019G088200 Sapur.019G088400
GO:0046914	37	1370	transition metal ion binding	Sapur.001G067900 Sapur.001G097100 Sapur.001G097300 Sapur.001G193000 Sapur.002G099300 Sapur.002G180700 Sapur.003G093200 Sapur.004G088800 Sapur.006G118800 Sapur.006G140500 Sapur.007G047300 Sapur.010G088700 Sapur.010G088800 Sapur.010G088900 Sapur.011G097000 Sapur.014G011100 Sapur.014G028500 Sapur.016G007400 Sapur.018G105100 Sapur.019G065400 Sapur.001G009100 Sapur.002G071000 Sapur.005G134500 Sapur.006G084800 Sapur.007G050900 Sapur.008G131500 Sapur.011G071900 Sapur.012G067900 Sapur.012G103800 Sapur.016G107800 Sapur.016G155100 Sapur.016G187100 Sapur.016G251200 Sapur.018G072600 Sapur.019G011100 Sapur.019G020800 Sapur.T051400
GO:0030151	2	5	molybdenum ion binding	Sapur.002G071000 Sapur.005G134500
GO:0005215	23	729	transporter activity	Sapur.010G194900 Sapur.001G034600 Sapur.001G036000 Sapur.001G053700 Sapur.001G120100 Sapur.002G053300 Sapur.003G115900 Sapur.003G148600 Sapur.005G131700 Sapur.005G137600 Sapur.006G023600 Sapur.006G023800 Sapur.008G101600 Sapur.012G020600 Sapur.014G090200 Sapur.016G089000 Sapur.017G057900 Sapur.017G063700 Sapur.T148900 Sapur.002G055900 Sapur.008G065700 Sapur.009G028900 Sapur.001G008400
GO:0046983	13	318	protein dimerization activity	Sapur.002G021100 Sapur.002G117000 Sapur.005G041600 Sapur.006G098600 Sapur.007G102800 Sapur.008G077700 Sapur.010G120100 Sapur.014G056000 Sapur.014G088300 Sapur.016G291000 Sapur.019G088200 Sapur.019G088400 Sapur.15ZG070000
GO:0008199	2	6	ferric iron binding	Sapur.006G084800 Sapur.016G107800

GO:0003700	19	584	DNA-binding transcription factor activity	Sapur.002G021100 Sapur.002G030100 Sapur.002G036700 Sapur.002G140800 Sapur.004G118800 Sapur.007G102800 Sapur.008G077700 Sapur.008G134800 Sapur.010G005200 Sapur.010G065900 Sapur.010G085300 Sapur.010G120100 Sapur.011G042400 Sapur.012G076500 Sapur.014G014900 Sapur.014G056000 Sapur.016G202700 Sapur.018G057700 Sapur.15ZG067200
GO:1901681	3	29	sulfur compound binding	Sapur.004G037400 Sapur.006G084800 Sapur.016G107800
GO:0032549	2	11	ribonucleoside binding	Sapur.T169500 Sapur.T175700
GO:0001882	2	12	nucleoside binding	Sapur.T169500 Sapur.T175700
GO:0003712	3	35	transcription coregulator activity	Sapur.001G009100 Sapur.007G050900 Sapur.012G067900
GO:0016811	2	15	hydrolase activity, acting on carbon-nitrogen (but not peptide) bonds, in linear amides	Sapur.005G122300 Sapur.T026400
GO:0005509	6	139	calcium ion binding	Sapur.002G009700 Sapur.002G103300 Sapur.014G019000 Sapur.014G019100 Sapur.014G019200 Sapur.014G020400
GO:0010309	1	2	acireductone dioxygenase [iron(II)-requiring] activity	Sapur.008G127300
GO:0016651	2	19	oxidoreductase activity, acting on NAD(P)H	Sapur.002G094800 Sapur.013G142900
GO:0016209	6	155	antioxidant activity	Sapur.001G055900 Sapur.005G153900 Sapur.006G130500 Sapur.007G049700 Sapur.016G055400 Sapur.012G093900
GO:0016758	10	324	hexosyltransferase activity	Sapur.002G182700 Sapur.006G005600 Sapur.006G018000 Sapur.007G026800 Sapur.007G077700 Sapur.007G125300 Sapur.016G020100 Sapur.016G151100 Sapur.016G189700 Sapur.016G270700
