# Supplementary material for: Insights into Molecular Mechanism of Secondary Xylem Rapid Growth in Salix psammophila
Source: Plants (Basel). 2025 Feb 5;14(3):459. doi: 10.3390/plants14030459 (PMC11819810; doi:10.3390/plants14030459)
Supplement: Supplementary file 1 [file plants-14-00459-s001.zip › Supplementary Table/Table S6 .pdf]

Table S6 Comparison
of phloem-specific differentially expressed genes GO annotation in 2, and 3-year-old.

	GOBPID	Count	Size	Term	gene_id
r_down_BP	GO:0016052	5	60	carbohydrate catabolic process	Sapur.003G056600 Sapur.004G122100 Sapur.009G098500 Sapur.016G305700 Sapur.003G053400
	GO:0006096	4	47	glycolytic process	Sapur.003G056600 Sapur.004G122100 Sapur.009G098500 Sapur.016G305700
	GO:0006090	4	50	pyruvate metabolic process	Sapur.003G056600 Sapur.004G122100 Sapur.009G098500 Sapur.016G305700
	GO:0015986	3	24	proton motive force-driven ATP synthesis	Sapur.004G008500 Sapur.012G055400 Sapur.018G022500
	GO:0006754	3	26	ATP biosynthetic process	Sapur.004G008500 Sapur.012G055400 Sapur.018G022500
	GO:0044281	13	536	small molecule metabolic process	Sapur.003G056600 Sapur.004G008500 Sapur.004G079100 Sapur.004G122100 Sapur.009G098500 Sapur.012G055400 Sapur.013G110500 Sapur.016G163100 Sapur.016G305700 Sapur.018G022500 Sapur.15ZG110400 Sapur.013G094800 Sapur.013G095000
	GO:0046034	3	31	ATP metabolic process	Sapur.004G008500 Sapur.012G055400 Sapur.018G022500
	GO:0009142	3	32	nucleoside triphosphate biosynthetic process	Sapur.004G008500 Sapur.012G055400 Sapur.018G022500
	GO:0009145	3	32	purine nucleoside triphosphate biosynthetic process	Sapur.004G008500 Sapur.012G055400 Sapur.018G022500
	GO:0009201	3	32	ribonucleoside triphosphate biosynthetic process	Sapur.004G008500 Sapur.012G055400 Sapur.018G022500
	GO:0009206	3	32	purine ribonucleoside triphosphate biosynthetic process	Sapur.004G008500 Sapur.012G055400 Sapur.018G022500
	GO:0009199	3	37	ribonucleoside triphosphate metabolic process	Sapur.004G008500 Sapur.012G055400 Sapur.018G022500
	GO:000914	3	37	nucleoside triphosphate metabolic	Sapur.004G008500 Sapur.012G055400 Sapur.018G022500

1			process	
GO:0009144	3	37	purine nucleoside triphosphate metabolic process	Sapur.004G008500 Sapur.012G055400 Sapur.018G022500
GO:0009205	3	37	purine ribonucleoside triphosphate metabolic process	Sapur.004G008500 Sapur.012G055400 Sapur.018G022500
GO:0005975	12	538	carbohydrate metabolic process	Sapur.003G056600 Sapur.004G012100 Sapur.004G122100 Sapur.005G052600 Sapur.008G050600 Sapur.009G098500 Sapur.010G123300 Sapur.016G054400 Sapur.016G054600 Sapur.016G094400 Sapur.016G305700 Sapur.003G053400
GO:0006091	4	89	generation of precursor metabolites and energy	Sapur.003G056600 Sapur.004G122100 Sapur.009G098500 Sapur.016G305700
GO:0046390	3	51	ribose phosphate biosynthetic process	Sapur.004G008500 Sapur.012G055400 Sapur.018G022500
GO:0009152	3	51	purine ribonucleotide biosynthetic process	Sapur.004G008500 Sapur.012G055400 Sapur.018G022500
GO:0009260	3	51	ribonucleotide biosynthetic process	Sapur.004G008500 Sapur.012G055400 Sapur.018G022500
GO:0032787	5	144	monocarboxylic acid metabolic process	Sapur.003G056600 Sapur.004G122100 Sapur.009G098500 Sapur.013G110500 Sapur.016G305700
GO:0055086	5	147	nucleobase-containing small molecule metabolic process	Sapur.004G008500 Sapur.012G055400 Sapur.018G022500 Sapur.013G094800 Sapur.013G095000
GO:0006562	1	2	proline catabolic process	Sapur.004G079100
GO:0009150	3	63	purine ribonucleotide metabolic process	Sapur.004G008500 Sapur.012G055400 Sapur.018G022500
GO:0009259	3	63	ribonucleotide metabolic process	Sapur.004G008500 Sapur.012G055400 Sapur.018G022500
GO:0019693	3	63	ribose phosphate metabolic process	Sapur.004G008500 Sapur.012G055400 Sapur.018G022500
GO:1902680	1	3	positive regulation of RNA biosynthetic process	Sapur.019G060100

	GO:0051254	1	3	positive regulation of RNA metabolic process	Sapur.019G060100
	GO:0045893	1	3	positive regulation of DNA-templated transcription	Sapur.019G060100
	GO:0009065	1	3	glutamine family amino acid catabolic process	Sapur.004G079100
	GO:0006560	1	3	proline metabolic process	Sapur.004G079100
	GO:0006164	3	66	purine nucleotide biosynthetic process	Sapur.004G008500 Sapur.012G055400 Sapur.018G022500
	GO:0072522	3	68	purine-containing compound biosynthetic process	Sapur.004G008500 Sapur.012G055400 Sapur.018G022500
	GO:0009116	2	30	nucleoside metabolic process	Sapur.013G094800 Sapur.013G095000
	GO:1901657	2	30	glycosyl compound metabolic process	Sapur.013G094800 Sapur.013G095000
	GO:0045935	1	4	positive regulation of nucleobase-containing compound metabolic process	Sapur.019G060100
	GO:0006729	1	4	tetrahydrobiopterin biosynthetic process	Sapur.15ZG110400
	GO:0046146	1	4	tetrahydrobiopterin metabolic process	Sapur.15ZG110400
	GO:0034311	1	4	diol metabolic process	Sapur.15ZG110400
	GO:0034312	1	4	diol biosynthetic process	Sapur.15ZG110400
	GO:1901575	6	249	organic substance catabolic process	Sapur.003G056600 Sapur.004G079100 Sapur.004G122100 Sapur.009G098500 Sapur.016G305700 Sapur.003G053400
r_up_BP	GO:000602	2	2	inositol biosynthetic process	Sapur.005G061300 Sapur.007G081700

1				
GO:0017062	2	2	respiratory chain complex III assembly	Sapur.006G138300 Sapur.018G069600
GO:0034551	2	2	mitochondrial respiratory chain complex III assembly	Sapur.006G138300 Sapur.018G069600
GO:0033108	2	3	mitochondrial respiratory chain complex assembly	Sapur.006G138300 Sapur.018G069600
GO:0006796	47	1954	phosphate-containing compound metabolic process	Sapur.002G060000 Sapur.002G130500 Sapur.003G139700 Sapur.004G016100 Sapur.004G016200 Sapur.005G043600 Sapur.005G043700 Sapur.005G061300 Sapur.005G188000 Sapur.006G163900 Sapur.006G195000 Sapur.007G048900 Sapur.007G064100 Sapur.007G081700 Sapur.008G123300 Sapur.009G022500 Sapur.010G042300 Sapur.010G070700 Sapur.011G024200 Sapur.012G100000 Sapur.013G020900 Sapur.013G028400 Sapur.013G035400 Sapur.013G133400 Sapur.016G008900 Sapur.016G010000 Sapur.016G185800 Sapur.016G270800 Sapur.016G284500 Sapur.017G001700 Sapur.017G002000 Sapur.017G036500 Sapur.017G036800 Sapur.017G039000 Sapur.017G098300 Sapur.018G015000 Sapur.018G114900 Sapur.018G115800 Sapur.018G115900 Sapur.018G119100 Sapur.019G082400 Sapur.019G107300 Sapur.15WG014200 Sapur.15WG048900 Sapur.15WG052300 Sapur.15ZG015100 Sapur.003G116600
GO:0006793	47	1955	phosphorus metabolic process	Sapur.002G060000 Sapur.002G130500 Sapur.003G139700 Sapur.004G016100 Sapur.004G016200 Sapur.005G043600 Sapur.005G043700 Sapur.005G061300 Sapur.005G188000 Sapur.006G163900 Sapur.006G195000 Sapur.007G048900 Sapur.007G064100 Sapur.007G081700 Sapur.008G123300 Sapur.009G022500 Sapur.010G042300 Sapur.010G070700 Sapur.011G024200 Sapur.012G100000 Sapur.013G020900 Sapur.013G028400 Sapur.013G035400 Sapur.013G133400 Sapur.016G008900 Sapur.016G010000 Sapur.016G185800 Sapur.016G270800 Sapur.016G284500 Sapur.017G001700 Sapur.017G002000 Sapur.017G036500 Sapur.017G036800 Sapur.017G039000 Sapur.017G098300 Sapur.018G015000 Sapur.018G114900 Sapur.018G115800 Sapur.018G115900 Sapur.018G119100 Sapur.019G082400 Sapur.019G107300 Sapur.15WG014200 Sapur.15WG048900

				Sapur.15WG052300 Sapur.15ZG015100 Sapur.003G116600
GO:0006020	2	5	inositol metabolic process	Sapur.005G061300 Sapur.007G081700
GO:0046165	2	6	alcohol biosynthetic process	Sapur.005G061300 Sapur.007G081700
GO:0046173	2	6	polyol biosynthetic process	Sapur.005G061300 Sapur.007G081700
GO:0036211	47	2018	protein modification process	Sapur.002G060000 Sapur.002G130500 Sapur.003G139700 Sapur.004G016100 Sapur.004G016200 Sapur.005G043600 Sapur.005G043700 Sapur.005G182300 Sapur.005G188000 Sapur.006G163900 Sapur.006G195000 Sapur.007G048900 Sapur.007G064100 Sapur.008G123300 Sapur.009G022500 Sapur.009G083300 Sapur.010G042300 Sapur.010G070700 Sapur.011G024200 Sapur.012G009700 Sapur.012G100000 Sapur.013G020900 Sapur.013G028400 Sapur.013G035400 Sapur.013G133400 Sapur.016G008900 Sapur.016G010000 Sapur.016G185800 Sapur.016G270800 Sapur.016G284500 Sapur.017G001700 Sapur.017G002000 Sapur.017G036500 Sapur.017G036800 Sapur.017G039000 Sapur.017G098300 Sapur.018G015000 Sapur.018G114900 Sapur.018G115800 Sapur.018G115900 Sapur.018G119100 Sapur.019G082400 Sapur.019G107300 Sapur.15WG014200 Sapur.15WG048900 Sapur.15WG052300 Sapur.15ZG015100
GO:0017004	2	7	cytochrome complex assembly	Sapur.006G138300 Sapur.018G069600
GO:0030001	9	202	metal ion transport	Sapur.006G015100 Sapur.012G003800 Sapur.017G121300 Sapur.017G121400 Sapur.017G121500 Sapur.017G121600 Sapur.017G121700 Sapur.017G122100 Sapur.15ZG002800
GO:0006468	38	1578	protein phosphorylation	Sapur.002G060000 Sapur.002G130500 Sapur.003G139700 Sapur.004G016100 Sapur.004G016200 Sapur.005G043600 Sapur.005G043700 Sapur.005G188000 Sapur.006G163900 Sapur.006G195000 Sapur.008G123300 Sapur.010G042300 Sapur.010G070700 Sapur.011G024200 Sapur.012G100000 Sapur.013G020900 Sapur.013G028400 Sapur.013G035400 Sapur.013G133400 Sapur.016G008900 Sapur.016G010000 Sapur.016G185800 Sapur.016G270800 Sapur.016G284500

				Sapur.017G001700 Sapur.017G002000 Sapur.017G036500 Sapur.017G036800 Sapur.017G039000 Sapur.017G098300 Sapur.018G015000 Sapur.018G114900 Sapur.018G115800 Sapur.018G115900 Sapur.019G082400 Sapur.019G107300 Sapur.15WG048900 Sapur.15WG052300
GO:0043412	47	2067	macromolecule modification	Sapur.002G060000 Sapur.002G130500 Sapur.003G139700 Sapur.004G016100 Sapur.004G016200 Sapur.005G043600 Sapur.005G043700 Sapur.005G182300 Sapur.005G188000 Sapur.006G163900 Sapur.006G195000 Sapur.007G048900 Sapur.007G064100 Sapur.008G123300 Sapur.009G022500 Sapur.009G083300 Sapur.010G042300 Sapur.010G070700 Sapur.011G024200 Sapur.012G009700 Sapur.012G100000 Sapur.013G020900 Sapur.013G028400 Sapur.013G035400 Sapur.013G133400 Sapur.016G008900 Sapur.016G010000 Sapur.016G185800 Sapur.016G270800 Sapur.016G284500 Sapur.017G001700 Sapur.017G002000 Sapur.017G036500 Sapur.017G036800 Sapur.017G039000 Sapur.017G098300 Sapur.018G015000 Sapur.018G114900 Sapur.018G115800 Sapur.018G115900 Sapur.018G119100 Sapur.019G082400 Sapur.019G107300 Sapur.15WG014200 Sapur.15WG048900 Sapur.15WG052300 Sapur.15ZG015100
GO:0016310	38	1593	phosphorylation	Sapur.002G060000 Sapur.002G130500 Sapur.003G139700 Sapur.004G016100 Sapur.004G016200 Sapur.005G043600 Sapur.005G043700 Sapur.005G188000 Sapur.006G163900 Sapur.006G195000 Sapur.008G123300 Sapur.010G042300 Sapur.010G070700 Sapur.011G024200 Sapur.012G100000 Sapur.013G020900 Sapur.013G028400 Sapur.013G035400 Sapur.013G133400 Sapur.016G008900 Sapur.016G010000 Sapur.016G185800 Sapur.016G270800 Sapur.016G284500 Sapur.017G001700 Sapur.017G002000 Sapur.017G036500 Sapur.017G036800 Sapur.017G039000 Sapur.017G098300 Sapur.018G015000 Sapur.018G114900 Sapur.018G115800 Sapur.018G115900 Sapur.019G082400 Sapur.019G107300 Sapur.15WG048900 Sapur.15WG052300
GO:1901617	2	11	organic hydroxy compound biosynthetic process	Sapur.005G061300 Sapur.007G081700
GO:0006812	9	265	monoatomic cation transport	Sapur.006G015100 Sapur.012G003800 Sapur.017G121300 Sapur.017G121400 Sapur.017G121500 Sapur.017G121600 Sapur.017G121700 Sapur.017G122100

					Sapur.15ZG002800
	GO:0019751	2	19	polyol metabolic process	Sapur.005G061300 Sapur.007G081700
	GO:0016311	6	154	dephosphorylation	Sapur.007G048900 Sapur.007G064100 Sapur.009G022500 Sapur.018G119100 Sapur.15WG014200 Sapur.15ZG015100
	GO:0006470	6	154	protein dephosphorylation	Sapur.007G048900 Sapur.007G064100 Sapur.009G022500 Sapur.018G119100 Sapur.15WG014200 Sapur.15ZG015100
	GO:0007005	2	20	mitochondrion organization	Sapur.006G138300 Sapur.018G069600
r_down_CC	GO:0045259	3	22	proton-transporting ATP synthase complex	Sapur.004G008500 Sapur.012G055400 Sapur.018G022500
	GO:0016020	23	1182	membrane	Sapur.001G036000 Sapur.001G046200 Sapur.001G053700 Sapur.003G114300 Sapur.003G148600 Sapur.006G023800 Sapur.006G119100 Sapur.010G176600 Sapur.013G110500 Sapur.013G142900 Sapur.014G134900 Sapur.017G073700 Sapur.017G100000 Sapur.017G111900 Sapur.018G046900 Sapur.15ZG060300 Sapur.T148900 Sapur.004G008500 Sapur.005G103700 Sapur.010G165900 Sapur.016G275200 Sapur.012G055400 Sapur.018G022500
	GO:0019898	3	29	extrinsic component of membrane	Sapur.005G103700 Sapur.010G165900 Sapur.016G275200
	GO:0045263	2	9	proton-transporting ATP synthase complex, coupling factor F(o)	Sapur.012G055400 Sapur.018G022500
	GO:0016469	3	42	proton-transporting two-sector ATPase complex	Sapur.004G008500 Sapur.012G055400 Sapur.018G022500
	GO:0033177	2	16	proton-transporting two-sector ATPase complex, proton-transporting domain	Sapur.012G055400 Sapur.018G022500
	GO:1904949	3	57	ATPase complex	Sapur.004G008500 Sapur.012G055400 Sapur.018G022500
r_up_CC	GO:0005884	1	2	actin filament	Sapur.15ZG061300

r_down_MF	GO:0005509	12	139	calcium ion binding	Sapur.003G154600 Sapur.005G103700 Sapur.010G028700 Sapur.010G165900 Sapur.014G020100 Sapur.014G020300 Sapur.014G020400 Sapur.016G275200 Sapur.018G092500 Sapur.T078700 Sapur.T079500 Sapur.T080000
	GO:0008171	6	34	O-methyltransferase activity	Sapur.013G111600 Sapur.019G088200 Sapur.019G088400 Sapur.019G088500 Sapur.019G088900 Sapur.019G089000
	GO:0046983	15	318	protein dimerization activity	Sapur.002G021100 Sapur.002G117000 Sapur.003G127000 Sapur.005G054200 Sapur.013G111600 Sapur.014G048200 Sapur.017G032900 Sapur.017G095300 Sapur.019G088200 Sapur.019G088400 Sapur.019G088500 Sapur.019G088900 Sapur.019G089000 Sapur.15ZG070000 Sapur.15ZG125300
	GO:0016491	38	1583	oxidoreductase activity	Sapur.001G034400 Sapur.002G057500 Sapur.002G153700 Sapur.004G079100 Sapur.004G088700 Sapur.005G134500 Sapur.005G176700 Sapur.006G113400 Sapur.008G131500 Sapur.009G079400 Sapur.011G077400 Sapur.012G048700 Sapur.013G132800 Sapur.016G155100 Sapur.016G251200 Sapur.016G253900 Sapur.016G300700 Sapur.15WG080500 Sapur.15ZG124800 Sapur.T051400 Sapur.013G098800 Sapur.016G117300 Sapur.15ZG114600 Sapur.002G061400 Sapur.002G133000 Sapur.005G203600 Sapur.013G142900 Sapur.001G097300 Sapur.002G099200 Sapur.002G099300 Sapur.002G180700 Sapur.006G140500 Sapur.010G021800 Sapur.010G088700 Sapur.010G088800 Sapur.010G088900 Sapur.010G198400 Sapur.016G007400
	GO:0003824	116	7297	catalytic activity	Sapur.001G034400 Sapur.001G043100 Sapur.001G190100 Sapur.002G057500 Sapur.002G134000 Sapur.002G153700 Sapur.003G025000 Sapur.004G079100 Sapur.004G088700 Sapur.004G168700 Sapur.005G134500 Sapur.005G176700 Sapur.006G113400 Sapur.008G000600 Sapur.008G019100 Sapur.008G131500 Sapur.009G059300 Sapur.009G079400 Sapur.011G053100 Sapur.011G077400 Sapur.011G093500 Sapur.011G121600 Sapur.012G048700 Sapur.013G111600 Sapur.013G132800 Sapur.014G015800 Sapur.016G155100 Sapur.016G215400 Sapur.016G251200 Sapur.016G253900 Sapur.016G271300 Sapur.016G300700 Sapur.016G305200 Sapur.019G069600 Sapur.019G088200 Sapur.019G088400 Sapur.019G088500 Sapur.019G088900 Sapur.019G089000 Sapur.15WG080500 Sapur.15ZG075400 Sapur.15ZG124800 Sapur.T051400 Sapur.001G034000

				Sapur.002G202900 Sapur.003G056600 Sapur.003G149600 Sapur.004G008500 Sapur.004G012100 Sapur.004G122100 Sapur.004G162500 Sapur.005G052600 Sapur.005G184000 Sapur.007G018500 Sapur.008G050600 Sapur.009G098500 Sapur.010G029000 Sapur.010G123300 Sapur.012G073400 Sapur.013G098800 Sapur.016G054400 Sapur.016G054600 Sapur.016G084000 Sapur.016G094400 Sapur.016G117300 Sapur.016G163100 Sapur.016G305700 Sapur.019G052800 Sapur.15WG035700 Sapur.15WG076200 Sapur.15WG080400 Sapur.15ZG110400 Sapur.15ZG114600 Sapur.001G105100 Sapur.002G040700 Sapur.002G061400 Sapur.002G120900 Sapur.002G133000 Sapur.002G165800 Sapur.003G077700 Sapur.004G006900 Sapur.004G057500 Sapur.004G068100 Sapur.005G203600 Sapur.006G013200 Sapur.006G013300 Sapur.006G013500 Sapur.006G018000 Sapur.006G080000 Sapur.007G125000 Sapur.007G125300 Sapur.007G125900 Sapur.009G029000 Sapur.013G094800 Sapur.013G095000 Sapur.013G110500 Sapur.013G142900 Sapur.014G010200 Sapur.016G020000 Sapur.016G151100 Sapur.017G063800 Sapur.019G015000 Sapur.019G024100 Sapur.019G047800 Sapur.001G097300 Sapur.002G099200 Sapur.002G099300 Sapur.002G180700 Sapur.006G115900 Sapur.006G140500 Sapur.010G021800 Sapur.010G088700 Sapur.010G088800 Sapur.010G088900 Sapur.010G198400 Sapur.016G007400
GO:0016829	10	166	lyase activity	Sapur.004G122100 Sapur.009G098500 Sapur.010G029000 Sapur.016G305700 Sapur.15ZG110400 Sapur.002G040700 Sapur.019G015000 Sapur.019G024100 Sapur.019G047800 Sapur.006G115900
GO:0016832	3	8	aldehyde-lyase activity	Sapur.004G122100 Sapur.009G098500 Sapur.016G305700
GO:0004332	3	8	fructose-bisphosphate aldolase activity	Sapur.004G122100 Sapur.009G098500 Sapur.016G305700
GO:0008168	11	235	methyltransferase activity	Sapur.013G111600 Sapur.019G088200 Sapur.019G088400 Sapur.019G088500 Sapur.019G088900 Sapur.019G089000 Sapur.002G202900 Sapur.005G184000 Sapur.007G018500 Sapur.016G084000 Sapur.15WG080400
GO:0016741	11	238	transferase activity, transferring one-carbon groups	Sapur.013G111600 Sapur.019G088200 Sapur.019G088400 Sapur.019G088500 Sapur.019G088900 Sapur.019G089000 Sapur.002G202900 Sapur.005G184000

				Sapur.007G018500 Sapur.016G084000 Sapur.15WG080400
GO:0046872	41	1929	metal ion binding	Sapur.001G097300 Sapur.002G099200 Sapur.002G099300 Sapur.002G180700 Sapur.003G056600 Sapur.003G154600 Sapur.004G001300 Sapur.005G103700 Sapur.006G140500 Sapur.010G021800 Sapur.010G028700 Sapur.010G088700 Sapur.010G088800 Sapur.010G088900 Sapur.010G165900 Sapur.014G020100 Sapur.014G020300 Sapur.014G020400 Sapur.016G007400 Sapur.016G275200 Sapur.018G092500 Sapur.T078700 Sapur.T079500 Sapur.T080000 Sapur.002G040700 Sapur.005G134500 Sapur.008G019100 Sapur.008G131500 Sapur.010G019800 Sapur.010G029000 Sapur.014G108000 Sapur.016G155100 Sapur.016G187100 Sapur.016G251200 Sapur.019G015000 Sapur.019G020800 Sapur.019G024100 Sapur.019G047800 Sapur.019G060100 Sapur.15ZG132500 Sapur.T051400
GO:0043169	41	1941	cation binding	Sapur.001G097300 Sapur.002G099200 Sapur.002G099300 Sapur.002G180700 Sapur.003G056600 Sapur.003G154600 Sapur.004G001300 Sapur.005G103700 Sapur.006G140500 Sapur.010G021800 Sapur.010G028700 Sapur.010G088700 Sapur.010G088800 Sapur.010G088900 Sapur.010G165900 Sapur.014G020100 Sapur.014G020300 Sapur.014G020400 Sapur.016G007400 Sapur.016G275200 Sapur.018G092500 Sapur.T078700 Sapur.T079500 Sapur.T080000 Sapur.002G040700 Sapur.005G134500 Sapur.008G019100 Sapur.008G131500 Sapur.010G019800 Sapur.010G029000 Sapur.014G108000 Sapur.016G155100 Sapur.016G187100 Sapur.016G251200 Sapur.019G015000 Sapur.019G020800 Sapur.019G024100 Sapur.019G047800 Sapur.019G060100 Sapur.15ZG132500 Sapur.T051400
GO:0019203	2	5	carbohydrate phosphatase activity	Sapur.008G050600 Sapur.016G094400
GO:0050308	2	5	sugar-phosphatase activity	Sapur.008G050600 Sapur.016G094400
GO:0042132	2	5	fructose 1,6-bisphosphate 1-phosphatase activity	Sapur.008G050600 Sapur.016G094400
GO:001675	11	324	hexosyltransferase activity	Sapur.006G013200 Sapur.006G013300 Sapur.006G013500 Sapur.006G018000

8				Sapur.007G125000 Sapur.007G125300 Sapur.007G125900 Sapur.009G029000 Sapur.016G020000 Sapur.016G151100 Sapur.017G063800
GO:0016757	13	445	glycosyltransferase activity	Sapur.001G034000 Sapur.15WG035700 Sapur.006G013200 Sapur.006G013300 Sapur.006G013500 Sapur.006G018000 Sapur.007G125000 Sapur.007G125300 Sapur.007G125900 Sapur.009G029000 Sapur.016G020000 Sapur.016G151100 Sapur.017G063800
GO:0046906	13	454	tetrapyrrole binding	Sapur.001G097300 Sapur.002G099200 Sapur.002G099300 Sapur.002G180700 Sapur.004G088700 Sapur.006G140500 Sapur.009G079400 Sapur.010G021800 Sapur.010G088700 Sapur.010G088800 Sapur.010G088900 Sapur.016G007400 Sapur.15ZG114600
GO:0020037	13	454	heme binding	Sapur.001G097300 Sapur.002G099200 Sapur.002G099300 Sapur.002G180700 Sapur.004G088700 Sapur.006G140500 Sapur.009G079400 Sapur.010G021800 Sapur.010G088700 Sapur.010G088800 Sapur.010G088900 Sapur.016G007400 Sapur.15ZG114600
GO:0016655	2	12	oxidoreductase activity, acting on NAD(P)H, quinone or similar compound as acceptor	Sapur.002G061400 Sapur.013G142900
GO:0016705	10	354	oxidoreductase activity, acting on paired donors, with incorporation or reduction of molecular oxygen	Sapur.001G097300 Sapur.002G099200 Sapur.002G099300 Sapur.002G180700 Sapur.006G140500 Sapur.010G021800 Sapur.010G088700 Sapur.010G088800 Sapur.010G088900 Sapur.016G007400
GO:0016830	3	42	carbon-carbon lyase activity	Sapur.004G122100 Sapur.009G098500 Sapur.016G305700
GO:0005506	10	373	iron ion binding	Sapur.001G097300 Sapur.002G099200 Sapur.002G099300 Sapur.002G180700 Sapur.006G140500 Sapur.010G021800 Sapur.010G088700 Sapur.010G088800 Sapur.010G088900 Sapur.016G007400
GO:0000287	5	125	magnesium ion binding	Sapur.002G040700 Sapur.003G056600 Sapur.019G015000 Sapur.019G024100 Sapur.019G047800
GO:0016651	2	19	oxidoreductase activity, acting on NAD(P)H	Sapur.002G061400 Sapur.013G142900
GO:000465	1	2	proline dehydrogenase activity	Sapur.004G079100

	7				
	GO:0016620	2	22	oxidoreductase activity, acting on the aldehyde or oxo group of donors, NAD or NADP as acceptor	Sapur.002G133000 Sapur.005G203600
	GO:0016649	1	3	oxidoreductase activity, acting on the CH-NH group of donors, quinone or similar compound as acceptor	Sapur.004G079100
	GO:0016903	2	26	oxidoreductase activity, acting on the aldehyde or oxo group of donors	Sapur.002G133000 Sapur.005G203600
	GO:0008124	1	4	4-alpha-hydroxytetrahydrobiopterin dehydratase activity	Sapur.15ZG110400
	GO:0015078	3	67	proton transmembrane transporter activity	Sapur.004G008500 Sapur.012G055400 Sapur.018G022500
	GO:0016872	2	2	intramolecular lyase activity	Sapur.005G061300 Sapur.007G081700
	GO:0004512	2	2	inositol-3-phosphate synthase activity	Sapur.005G061300 Sapur.007G081700
	GO:0005506	14	373	iron ion binding	Sapur.001G097000 Sapur.002G032500 Sapur.003G024300 Sapur.004G051700 Sapur.005G034700 Sapur.006G045200 Sapur.007G103200 Sapur.009G053600 Sapur.011G066900 Sapur.011G067400 Sapur.013G143000 Sapur.014G027100 Sapur.014G027400 Sapur.016G214600
r_up_MF	GO:0016705	13	354	oxidoreductase activity, acting on paired donors, with incorporation or reduction of molecular oxygen	Sapur.001G097000 Sapur.002G032500 Sapur.003G024300 Sapur.005G034700 Sapur.006G045200 Sapur.007G103200 Sapur.009G053600 Sapur.011G066900 Sapur.011G067400 Sapur.013G143000 Sapur.014G027100 Sapur.014G027400 Sapur.016G214600
	GO:0140096	57	2472	catalytic activity, acting on a protein	Sapur.002G060000 Sapur.002G130500 Sapur.002G153400 Sapur.003G139700 Sapur.004G016100 Sapur.004G016200 Sapur.005G043600 Sapur.005G043700 Sapur.005G182300 Sapur.005G188000 Sapur.006G055700 Sapur.006G163900 Sapur.006G195000 Sapur.008G123300 Sapur.009G083300 Sapur.010G042300 Sapur.010G070700 Sapur.011G024200 Sapur.012G009700 Sapur.012G100000

				Sapur.013G020900 Sapur.013G028400 Sapur.013G035400 Sapur.013G133400 Sapur.014G107200 Sapur.014G107500 Sapur.016G008900 Sapur.016G010000 Sapur.016G185800 Sapur.016G270800 Sapur.016G284500 Sapur.017G001700 Sapur.017G002000 Sapur.017G010000 Sapur.017G036500 Sapur.017G036800 Sapur.017G039000 Sapur.017G098300 Sapur.018G010400 Sapur.018G015000 Sapur.018G114900 Sapur.018G115800 Sapur.018G115900 Sapur.019G016700 Sapur.019G065500 Sapur.019G082400 Sapur.019G107300 Sapur.15WG048900 Sapur.15WG052300 Sapur.007G048900 Sapur.007G064100 Sapur.009G022500 Sapur.010G050700 Sapur.012G084700 Sapur.018G119100 Sapur.15WG014200 Sapur.15ZG015100
GO:0004672	38	1565	protein kinase activity	Sapur.002G060000 Sapur.002G130500 Sapur.003G139700 Sapur.004G016100 Sapur.004G016200 Sapur.005G043600 Sapur.005G043700 Sapur.005G188000 Sapur.006G163900 Sapur.006G195000 Sapur.008G123300 Sapur.010G042300 Sapur.010G070700 Sapur.011G024200 Sapur.012G100000 Sapur.013G020900 Sapur.013G028400 Sapur.013G035400 Sapur.013G133400 Sapur.016G008900 Sapur.016G010000 Sapur.016G185800 Sapur.016G270800 Sapur.016G284500 Sapur.017G001700 Sapur.017G002000 Sapur.017G036500 Sapur.017G036800 Sapur.017G039000 Sapur.017G098300 Sapur.018G015000 Sapur.018G114900 Sapur.018G115800 Sapur.018G115900 Sapur.019G082400 Sapur.019G107300 Sapur.15WG048900 Sapur.15WG052300
GO:0004722	6	124	protein serine/threonine phosphatase activity	Sapur.007G048900 Sapur.007G064100 Sapur.009G022500 Sapur.018G119100 Sapur.15WG014200 Sapur.15ZG015100
GO:0004553	12	357	hydrolase activity, hydrolyzing O-glycosyl compounds	Sapur.001G112100 Sapur.002G182500 Sapur.004G137100 Sapur.006G114600 Sapur.014G071900 Sapur.014G072000 Sapur.014G118500 Sapur.018G070800 Sapur.019G037200 Sapur.T006700 Sapur.T171300 Sapur.010G143900
GO:0016491	38	1583	oxidoreductase activity	Sapur.001G193100 Sapur.002G153400 Sapur.003G090600 Sapur.003G097100 Sapur.004G051700 Sapur.004G117800 Sapur.005G153800 Sapur.011G111200 Sapur.012G002800 Sapur.012G110300 Sapur.014G107200 Sapur.014G107500 Sapur.016G139000 Sapur.016G226800 Sapur.016G226900 Sapur.016G241900 Sapur.017G010000 Sapur.019G084900 Sapur.019G085000 Sapur.15ZG109800

				Sapur.001G129600 Sapur.010G050700 Sapur.013G058600 Sapur.017G066000 Sapur.15ZG110300 Sapur.001G097000 Sapur.002G032500 Sapur.003G024300 Sapur.005G034700 Sapur.006G045200 Sapur.007G103200 Sapur.009G053600 Sapur.011G066900 Sapur.011G067400 Sapur.013G143000 Sapur.014G027100 Sapur.014G027400 Sapur.016G214600
GO:0046906	14	454	tetrapyrrole binding	Sapur.001G097000 Sapur.002G032500 Sapur.003G024300 Sapur.005G034700 Sapur.005G153800 Sapur.006G045200 Sapur.007G103200 Sapur.009G053600 Sapur.011G066900 Sapur.011G067400 Sapur.013G143000 Sapur.014G027100 Sapur.014G027400 Sapur.016G214600
GO:0020037	14	454	heme binding	Sapur.001G097000 Sapur.002G032500 Sapur.003G024300 Sapur.005G034700 Sapur.005G153800 Sapur.006G045200 Sapur.007G103200 Sapur.009G053600 Sapur.011G066900 Sapur.011G067400 Sapur.013G143000 Sapur.014G027100 Sapur.014G027400 Sapur.016G214600
GO:0015035	5	98	protein-disulfide reductase activity	Sapur.002G153400 Sapur.014G107200 Sapur.014G107500 Sapur.017G010000 Sapur.010G050700
GO:0015036	5	101	disulfide oxidoreductase activity	Sapur.002G153400 Sapur.014G107200 Sapur.014G107500 Sapur.017G010000 Sapur.010G050700
GO:0016798	12	379	hydrolase activity, acting on glycosyl bonds	Sapur.001G112100 Sapur.002G182500 Sapur.004G137100 Sapur.006G114600 Sapur.014G071900 Sapur.014G072000 Sapur.014G118500 Sapur.018G070800 Sapur.019G037200 Sapur.T006700 Sapur.T171300 Sapur.010G143900
GO:0003824	141	7297	catalytic activity	Sapur.001G193100 Sapur.002G060000 Sapur.002G130500 Sapur.002G132500 Sapur.002G144200 Sapur.002G153400 Sapur.003G061600 Sapur.003G090600 Sapur.003G097100 Sapur.003G139700 Sapur.004G016100 Sapur.004G016200 Sapur.004G051700 Sapur.004G117800 Sapur.005G043600 Sapur.005G043700 Sapur.005G153800 Sapur.005G182300 Sapur.005G188000 Sapur.006G055700 Sapur.006G163900 Sapur.006G195000 Sapur.007G093800 Sapur.008G123300 Sapur.009G083300 Sapur.009G111300 Sapur.010G042300 Sapur.010G070700 Sapur.011G024200 Sapur.011G111200 Sapur.012G002800 Sapur.012G009700 Sapur.012G100000 Sapur.012G110300 Sapur.013G020900 Sapur.013G028400 Sapur.013G035400 Sapur.013G104300 Sapur.013G133400 Sapur.014G107200

				Sapur.014G107500 Sapur.016G008900 Sapur.016G010000 Sapur.016G139000 Sapur.016G185800 Sapur.016G226800 Sapur.016G226900 Sapur.016G241900 Sapur.016G270800 Sapur.016G284500 Sapur.017G001700 Sapur.017G002000 Sapur.017G010000 Sapur.017G036500 Sapur.017G036800 Sapur.017G039000 Sapur.017G098300 Sapur.018G010400 Sapur.018G015000 Sapur.018G114900 Sapur.018G115800 Sapur.018G115900 Sapur.019G016700 Sapur.019G065500 Sapur.019G082400 Sapur.019G084900 Sapur.019G085000 Sapur.019G107300 Sapur.15WG048900 Sapur.15WG052300 Sapur.15ZG109800 Sapur.001G112100 Sapur.001G129600 Sapur.002G182500 Sapur.003G116600 Sapur.004G137100 Sapur.004G173300 Sapur.005G061300 Sapur.006G092400 Sapur.006G114600 Sapur.007G048900 Sapur.007G064100 Sapur.007G081700 Sapur.009G022500 Sapur.010G050700 Sapur.011G098300 Sapur.012G084700 Sapur.013G058600 Sapur.014G071900 Sapur.014G072000 Sapur.014G118500 Sapur.016G069000 Sapur.017G066000 Sapur.017G066600 Sapur.017G112500 Sapur.017G124700 Sapur.018G021000 Sapur.018G070800 Sapur.018G119100 Sapur.019G037200 Sapur.15WG014200 Sapur.15ZG015100 Sapur.15ZG047100 Sapur.15ZG080600 Sapur.15ZG081500 Sapur.15ZG110300 Sapur.15ZG127900 Sapur.T006700 Sapur.T171300 Sapur.002G076000 Sapur.003G101600 Sapur.003G133600 Sapur.004G018300 Sapur.004G042500 Sapur.005G021300 Sapur.006G083700 Sapur.006G141500 Sapur.006G143600 Sapur.008G056400 Sapur.008G110500 Sapur.008G112400 Sapur.010G143900 Sapur.012G058000 Sapur.012G111000 Sapur.014G031700 Sapur.016G166200 Sapur.016G182100 Sapur.017G083100 Sapur.001G097000 Sapur.002G032500 Sapur.003G024300 Sapur.005G034700 Sapur.006G045200 Sapur.007G103200 Sapur.009G053600 Sapur.011G066900 Sapur.011G067400 Sapur.013G143000 Sapur.014G027100 Sapur.014G027400 Sapur.016G214600
GO:0033925	1	2	mannosyl-glycoprotein endo-beta-N-acetylglucosaminidase activity	Sapur.010G143900
GO:004356	10	300	sequence-specific DNA binding	Sapur.001G067700 Sapur.003G102000 Sapur.005G067000 Sapur.006G089400

5				Sapur.006G122700 Sapur.006G217600 Sapur.009G016800 Sapur.014G036800 Sapur.014G074800 Sapur.014G081100
GO:0043167	92	4562	ion binding	Sapur.001G097000 Sapur.002G032500 Sapur.002G036100 Sapur.002G060000 Sapur.003G024300 Sapur.004G051700 Sapur.004G175300 Sapur.005G034700 Sapur.006G015100 Sapur.006G045200 Sapur.007G103200 Sapur.008G129000 Sapur.009G053600 Sapur.011G066900 Sapur.011G067400 Sapur.012G003800 Sapur.013G143000 Sapur.014G027100 Sapur.014G027400 Sapur.016G214600 Sapur.017G121300 Sapur.017G121400 Sapur.017G121500 Sapur.017G121600 Sapur.017G121700 Sapur.017G122100 Sapur.15ZG002800 Sapur.15ZG110300 Sapur.T080100 Sapur.002G012000 Sapur.002G130500 Sapur.002G132500 Sapur.002G144200 Sapur.003G061600 Sapur.003G139700 Sapur.004G016100 Sapur.004G018300 Sapur.004G051400 Sapur.004G112100 Sapur.005G031700 Sapur.005G043600 Sapur.005G043700 Sapur.005G087300 Sapur.006G083700 Sapur.006G138300 Sapur.006G195000 Sapur.007G008900 Sapur.007G016800 Sapur.007G088300 Sapur.007G093800 Sapur.008G123300 Sapur.008G128300 Sapur.009G002700 Sapur.009G008500 Sapur.009G094100 Sapur.009G094200 Sapur.010G042900 Sapur.010G070700 Sapur.010G143600 Sapur.011G102200 Sapur.012G100000 Sapur.012G104300 Sapur.013G104300 Sapur.013G133400 Sapur.013G134200 Sapur.016G053300 Sapur.016G139000 Sapur.016G200900 Sapur.016G311100 Sapur.017G002000 Sapur.017G036500 Sapur.017G036800 Sapur.017G039000 Sapur.017G085100 Sapur.017G124700 Sapur.018G015000 Sapur.018G069600 Sapur.018G105800 Sapur.018G114900 Sapur.018G115800 Sapur.018G115900 Sapur.019G000800 Sapur.019G028700 Sapur.019G028900 Sapur.019G029100 Sapur.019G082400 Sapur.019G084900 Sapur.019G085000 Sapur.019G107300 Sapur.15WG048900 Sapur.15WG052300 Sapur.15ZG112200
GO:0016301	38	1669	kinase activity	Sapur.002G060000 Sapur.002G130500 Sapur.003G139700 Sapur.004G016100 Sapur.004G016200 Sapur.005G043600 Sapur.005G043700 Sapur.005G188000 Sapur.006G163900 Sapur.006G195000 Sapur.008G123300 Sapur.010G042300 Sapur.010G070700 Sapur.011G024200 Sapur.012G100000 Sapur.013G020900 Sapur.013G028400 Sapur.013G035400 Sapur.013G133400 Sapur.016G008900

			Sapur.016G010000 Sapur.016G185800 Sapur.016G270800 Sapur.016G284500 Sapur.017G001700 Sapur.017G002000 Sapur.017G036500 Sapur.017G036800 Sapur.017G039000 Sapur.017G098300 Sapur.018G015000 Sapur.018G114900 Sapur.018G115800 Sapur.018G115900 Sapur.019G082400 Sapur.019G107300 Sapur.15WG048900 Sapur.15WG052300
GO:0046872	43	1929	metal ion binding	Sapur.001G097000 Sapur.002G032500 Sapur.002G036100 Sapur.002G060000 Sapur.003G024300 Sapur.004G051700 Sapur.004G175300 Sapur.005G034700 Sapur.006G015100 Sapur.006G045200 Sapur.007G103200 Sapur.008G129000 Sapur.009G053600 Sapur.011G066900 Sapur.011G067400 Sapur.012G003800 Sapur.013G143000 Sapur.014G027100 Sapur.014G027400 Sapur.016G214600 Sapur.017G121300 Sapur.017G121400 Sapur.017G121500 Sapur.017G121600 Sapur.017G121700 Sapur.017G122100 Sapur.15ZG002800 Sapur.15ZG110300 Sapur.T080100 Sapur.002G012000 Sapur.004G018300 Sapur.004G112100 Sapur.005G087300 Sapur.006G083700 Sapur.007G088300 Sapur.009G002700 Sapur.009G008500 Sapur.011G102200 Sapur.013G134200 Sapur.016G139000 Sapur.016G311100 Sapur.019G084900 Sapur.019G085000
GO:0003700	16	584	DNA-binding transcription factor activity	Sapur.001G067700 Sapur.003G102000 Sapur.005G067000 Sapur.006G037000 Sapur.006G089400 Sapur.006G122700 Sapur.006G217600 Sapur.009G016800 Sapur.010G120100 Sapur.013G041800 Sapur.013G096900 Sapur.014G036800 Sapur.014G074800 Sapur.014G081100 Sapur.014G101000 Sapur.15ZG126100
GO:0016773	38	1675	phosphotransferase activity, alcohol group as acceptor	Sapur.002G060000 Sapur.002G130500 Sapur.003G139700 Sapur.004G016100 Sapur.004G016200 Sapur.005G043600 Sapur.005G043700 Sapur.005G188000 Sapur.006G163900 Sapur.006G195000 Sapur.008G123300 Sapur.010G042300 Sapur.010G070700 Sapur.011G024200 Sapur.012G100000 Sapur.013G020900 Sapur.013G028400 Sapur.013G035400 Sapur.013G133400 Sapur.016G008900 Sapur.016G010000 Sapur.016G185800 Sapur.016G270800 Sapur.016G284500 Sapur.017G001700 Sapur.017G002000 Sapur.017G036500 Sapur.017G036800 Sapur.017G039000 Sapur.017G098300 Sapur.018G015000 Sapur.018G114900 Sapur.018G115800 Sapur.018G115900 Sapur.019G082400 Sapur.019G107300 Sapur.15WG048900 Sapur.15WG052300

GO:0016791	7	192	phosphatase activity	Sapur.004G173300 Sapur.007G048900 Sapur.007G064100 Sapur.009G022500 Sapur.018G119100 Sapur.15WG014200 Sapur.15ZG015100
GO:0043169	43	1941	cation binding	Sapur.001G097000 Sapur.002G032500 Sapur.002G036100 Sapur.002G060000 Sapur.003G024300 Sapur.004G051700 Sapur.004G175300 Sapur.005G034700 Sapur.006G015100 Sapur.006G045200 Sapur.007G103200 Sapur.008G129000 Sapur.009G053600 Sapur.011G066900 Sapur.011G067400 Sapur.012G003800 Sapur.013G143000 Sapur.014G027100 Sapur.014G027400 Sapur.016G214600 Sapur.017G121300 Sapur.017G121400 Sapur.017G121500 Sapur.017G121600 Sapur.017G121700 Sapur.017G122100 Sapur.15ZG002800 Sapur.15ZG110300 Sapur.T080100 Sapur.002G012000 Sapur.004G018300 Sapur.004G112100 Sapur.005G087300 Sapur.006G083700 Sapur.007G088300 Sapur.009G002700 Sapur.009G008500 Sapur.011G102200 Sapur.013G134200 Sapur.016G139000 Sapur.016G311100 Sapur.019G084900 Sapur.019G085000
