# Supplementary material for: Insights into Molecular Mechanism of Secondary Xylem Rapid Growth in Salix psammophila
Source: Plants (Basel). 2025 Feb 5;14(3):459. doi: 10.3390/plants14030459 (PMC11819810; doi:10.3390/plants14030459)
Supplement: Supplementary file 1 [file plants-14-00459-s001.zip › Supplementary Table/Table S7.pdf]

Table S7 Genes regulating turquoise module-related transcription factors.

| X | Description | PFAMs |
|---|---|---|
| **Sapur.001G004000** | transcription repressor | Myb_DNA-binding |
| **Sapur.001G016300** | Myb/SANT-like DNA-binding domain | Myb_DNA-bind_4 |
| **Sapur.001G016300** | Myb/SANT-like DNA-binding domain | Myb_DNA-bind_4 |
| **Sapur.001G021900** | Myb/SANT-like DNA-binding domain | Myb_DNA-bind_4 |
| **Sapur.001G023600** | Myb/SANT-like DNA-binding domain | Myb_DNA-bind_4 |
| **Sapur.001G027800** | Nascent polypeptide-associated complex subunit alpha-like | NAC |
| **Sapur.001G028700** | dnaJ homolog subfamily C member | DnaJ,Myb_DNA-binding |
| **Sapur.001G028800** | transcription factor | Myb_DNA-binding |
| **Sapur.001G028800** | transcription factor | Myb_DNA-binding |
| **Sapur.001G028800** | transcription factor | Myb_DNA-binding |
| **Sapur.001G028800** | transcription factor | Myb_DNA-binding |
| **Sapur.001G052200** | Myb/SANT-like DNA-binding domain | Myb_DNA-bind_4 |
| **Sapur.001G052200** | Myb/SANT-like DNA-binding domain | Myb_DNA-bind_4 |
| **Sapur.001G060000** | Transcription factor | Myb_DNA-binding |
| **Sapur.001G070400** | SANT SWI3, ADA2, N-CoR and TFIIIB'' DNA-binding domains | Myb_DNA-binding |
| **Sapur.001G070500** | SANT SWI3, ADA2, N-CoR and TFIIIB'' DNA-binding domains | Myb_DNA-binding |
| **Sapur.001G070500** | SANT SWI3, ADA2, N-CoR and TFIIIB'' DNA-binding domains | Myb_DNA-binding |
| **Sapur.001G070600** | SANT SWI3, ADA2, N-CoR and TFIIIB'' DNA-binding domains | Myb_DNA-binding |
| **Sapur.001G093000** | Myb/SANT-like DNA-binding domain | Myb_DNA-bind_4 |
| **Sapur.001G113200** | transcription factor | Myb_DNA-binding |
| **Sapur.001G113200** | transcription factor | Myb_DNA-binding |
| **Sapur.001G113200** | transcription factor | Myb_DNA-binding |
| **Sapur.001G113200** | transcription factor | Myb_DNA-binding |
| **Sapur.001G113200** | transcription factor | Myb_DNA-binding |

| | | |
|---|---|---|
| **Sapur.001G113200** | transcription factor | Myb_DNA-binding |
| **Sapur.001G117100** | PLATZ transcription factor family protein | PLATZ |
| **Sapur.001G121200** | Two-component response regulator-like | Myb_DNA-binding,Response_reg |
| **Sapur.001G121200** | Two-component response regulator-like | Myb_DNA-binding,Response_reg |
| **Sapur.001G121200** | Two-component response regulator-like | Myb_DNA-binding,Response_reg |
| **Sapur.001G121200** | Two-component response regulator-like | Myb_DNA-binding,Response_reg |
| **Sapur.001G121200** | Two-component response regulator-like | Myb_DNA-binding,Response_reg |
| **Sapur.001G121200** | Two-component response regulator-like | Myb_DNA-binding,Response_reg |
| **Sapur.001G121200** | Two-component response regulator-like | Myb_DNA-binding,Response_reg |
| **Sapur.001G121200** | Two-component response regulator-like | Myb_DNA-binding,Response_reg |
| **Sapur.001G121200** | Two-component response regulator-like | Myb_DNA-binding,Response_reg |
| **Sapur.001G121200** | Two-component response regulator-like | Myb_DNA-binding,Response_reg |
| **Sapur.001G121200** | Two-component response regulator-like | Myb_DNA-binding,Response_reg |
| **Sapur.001G121200** | Two-component response regulator-like | Myb_DNA-binding,Response_reg |
| **Sapur.001G121900** | dnaJ homolog subfamily C member | Myb_DNA-bind_6,Myb_DNA-binding |
| **Sapur.001G129000** | Homeobox-leucine zipper protein | HALZ,HD-ZIP_N,Homeobox |
| **Sapur.001G129300** | transcription factor | Myb_DNA-binding |
| **Sapur.001G129700** | transcription factor | Myb_DNA-binding |
| **Sapur.001G130300** | Plant lipoxygenase may be involved in a number of diverse aspects of plant physiology including growth and development, pest resistance, and senescence or responses to wounding | Lipoxygenase,PLAT |
| **Sapur.001G130300** | Plant lipoxygenase may be involved in a number of diverse aspects of plant physiology including growth | Lipoxygenase,PLAT |

| | | |
|---|---|---|
| | and development, pest resistance, and senescence or responses to wounding | |
| **Sapur.001G147500** | isoform X1 | Myb_DNA-bind_6,Myb_DNA-binding |
| **Sapur.001G147500** | isoform X1 | Myb_DNA-bind_6,Myb_DNA-binding |
| **Sapur.001G147500** | isoform X1 | Myb_DNA-bind_6,Myb_DNA-binding |
| **Sapur.001G147900** | transcription factor | Myb_DNA-binding |
| **Sapur.001G150000** | Myb-related protein | Myb_DNA-binding |
| **Sapur.001G150000** | Myb-related protein | Myb_DNA-binding |
| **Sapur.001G154100** | Myb/SANT-like DNA-binding domain | Myb_DNA-bind_4 |
| **Sapur.001G161900** | transcription factor | Myb_DNA-binding |
| **Sapur.001G171900** | Protein PHOTOPERIOD-INDEPENDENT EARLY FLOWERING | HSA,Helicase_C,Myb_DNA-bind_6,SNF2_N |
| **Sapur.001G171900** | Protein PHOTOPERIOD-INDEPENDENT EARLY FLOWERING | HSA,Helicase_C,Myb_DNA-bind_6,SNF2_N |
| **Sapur.001G171900** | Protein PHOTOPERIOD-INDEPENDENT EARLY FLOWERING | HSA,Helicase_C,Myb_DNA-bind_6,SNF2_N |
| **Sapur.002G001300** | PLATZ transcription factor | PLATZ |
| **Sapur.002G001300** | PLATZ transcription factor | PLATZ |
| **Sapur.002G001300** | PLATZ transcription factor | PLATZ |
| **Sapur.002G002800** | SWI SNF complex subunit | Myb_DNA-binding,SWIRM,SWIRM-assoc_1 |
| **Sapur.002G002800** | SWI SNF complex subunit | Myb_DNA-binding,SWIRM,SWIRM-assoc_1 |
| **Sapur.002G003600** | SANT SWI3, ADA2, N-CoR and TFIIIB'' DNA-binding domains | Myb_DNA-bind_6,Myb_DNA-binding |
| **Sapur.002G003600** | SANT SWI3, ADA2, N-CoR and TFIIIB'' DNA-binding domains | Myb_DNA-bind_6,Myb_DNA-binding |
| **Sapur.002G026800** | RADIALIS-like | Myb_DNA-binding |
| **Sapur.002G029500** | Myb-related protein | Myb_DNA-binding |
| **Sapur.002G029500** | Myb-related protein | Myb_DNA-binding |
| **Sapur.002G029500** | Myb-related protein | Myb_DNA-binding |
| **Sapur.002G053600** | Myb/SANT-like DNA-binding domain | Myb_DNA-bind_4 |
| **Sapur.002G064100** | SWI SNF complex subunit | Myb_DNA-binding,SWIRM,SW |

| | | |
|---|---|---|
| | | IRM-assoc_1 |
| **Sapur.002G064100** | SWI SNF complex subunit | Myb_DNA-binding,SWIRM,SWIRM-assoc_1 |
| **Sapur.002G085700** | SANT SWI3, ADA2, N-CoR and TFIIIB'' DNA-binding domains | Myb_DNA-binding |
| **Sapur.002G085700** | SANT SWI3, ADA2, N-CoR and TFIIIB'' DNA-binding domains | Myb_DNA-binding |
| **Sapur.002G085700** | SANT SWI3, ADA2, N-CoR and TFIIIB'' DNA-binding domains | Myb_DNA-binding |
| **Sapur.002G085700** | SANT SWI3, ADA2, N-CoR and TFIIIB'' DNA-binding domains | Myb_DNA-binding |
| **Sapur.002G099700** | transcription factor | Myb_DNA-binding |
| **Sapur.002G110600** | Homeobox-leucine zipper protein | HALZ,HD-ZIP_N,Homeobox |
| **Sapur.002G113600** | Alcohol dehydrogenase transcription factor Myb/SANT-like | Myb_DNA-bind_4 |
| **Sapur.002G123000** | Synaptotagmin-like mitochondrial-lipid-binding domain | C2,SMP_LBD |
| **Sapur.002G123000** | Synaptotagmin-like mitochondrial-lipid-binding domain | C2,SMP_LBD |
| **Sapur.002G124900** | response regulator | Myb_DNA-binding,Response_reg |
| **Sapur.002G131300** | AT-rich interactive domain-containing protein | ARID,Myb_DNA-binding |
| **Sapur.002G132800** | SANT SWI3, ADA2, N-CoR and TFIIIB'' DNA-binding domains | Myb_DNA-binding |
| **Sapur.002G137500** | Protein LHY-like isoform X1 | Myb_DNA-binding |
| **Sapur.002G137500** | Protein LHY-like isoform X1 | Myb_DNA-binding |
| **Sapur.002G142500** | SANT SWI3, ADA2, N-CoR and TFIIIB'' DNA-binding domains | Myb_DNA-binding |
| **Sapur.002G150500** | reveille | Myb_DNA-binding |
| **Sapur.002G160000** | Myb/SANT-like DNA-binding domain | Myb_DNA-bind_3 |
| **Sapur.002G160000** | Myb/SANT-like DNA-binding domain | Myb_DNA-bind_3 |

| | | |
|---|---|---|
| **Sapur.002G166100** | transcription factor | Myb_DNA-binding |
| **Sapur.002G188400** | Myb/SANT-like DNA-binding domain | Myb_DNA-bind_4 |
| **Sapur.002G188500** | HSA | HSA,Myb_DNA-bind_6 |
| **Sapur.002G188500** | HSA | HSA,Myb_DNA-bind_6 |
| **Sapur.002G188500** | HSA | HSA,Myb_DNA-bind_6 |
| **Sapur.002G188500** | HSA | HSA,Myb_DNA-bind_6 |
| **Sapur.002G188500** | HSA | HSA,Myb_DNA-bind_6 |
| **Sapur.002G201000** | Protein PHR1-LIKE 1-like isoform | Myb_CC_LHEQLE,Myb_DNA-binding |
| **Sapur.002G201000** | Protein PHR1-LIKE 1-like isoform | Myb_CC_LHEQLE,Myb_DNA-binding |
| **Sapur.002G201000** | Protein PHR1-LIKE 1-like isoform | Myb_CC_LHEQLE,Myb_DNA-binding |
| **Sapur.002G201000** | Protein PHR1-LIKE 1-like isoform | Myb_CC_LHEQLE,Myb_DNA-binding |
| **Sapur.002G201000** | Protein PHR1-LIKE 1-like isoform | Myb_CC_LHEQLE,Myb_DNA-binding |
| **Sapur.002G201000** | Protein PHR1-LIKE 1-like isoform | Myb_CC_LHEQLE,Myb_DNA-binding |
| **Sapur.002G201000** | Protein PHR1-LIKE 1-like isoform | Myb_CC_LHEQLE,Myb_DNA-binding |
| **Sapur.002G201000** | Protein PHR1-LIKE 1-like isoform | Myb_CC_LHEQLE,Myb_DNA-binding |
| **Sapur.002G201000** | Protein PHR1-LIKE 1-like isoform | Myb_CC_LHEQLE,Myb_DNA-binding |
| **Sapur.002G201000** | Protein PHR1-LIKE 1-like isoform | Myb_CC_LHEQLE,Myb_DNA-binding |
| **Sapur.002G201000** | Protein PHR1-LIKE 1-like isoform | Myb_CC_LHEQLE,Myb_DNA-binding |
| **Sapur.002G201000** | Protein PHR1-LIKE 1-like isoform | Myb_CC_LHEQLE,Myb_DNA-binding |
| **Sapur.002G201000** | Protein PHR1-LIKE 1-like isoform | Myb_CC_LHEQLE,Myb_DNA-binding |
| **Sapur.002G202700** | radialis-like | Myb_DNA-binding |
| **Sapur.003G013100** | Protein PHOTOPERIOD-INDEPENDENT EARLY FLOWERING | HSA,Helicase_C,Myb_DNA-bind_6,SNF2_N |
| **Sapur.003G013100** | Protein PHOTOPERIOD-INDEPENDENT EARLY FLOWERING | HSA,Helicase_C,Myb_DNA-bind_6,SNF2_N |
| **Sapur.003G013100** | Protein PHOTOPERIOD-INDEPENDENT EARLY FLOWERING | HSA,Helicase_C,Myb_DNA-bind_6,SNF2_N |

| | | |
|---|---|---|
| **Sapur.003G013100** | Protein PHOTOPERIOD-INDEPENDENT EARLY FLOWERING | HSA,Helicase_C,Myb_DNA-bind_6,SNF2_N |
| **Sapur.003G027300** | transcription factor | Myb_DNA-binding |
| **Sapur.003G029400** | Myb/SANT-like DNA-binding domain | Myb_DNA-bind_4 |
| **Sapur.003G035500** | isoform X1 | Myb_DNA-bind_6,Myb_DNA-binding |
| **Sapur.003G035500** | isoform X1 | Myb_DNA-bind_6,Myb_DNA-binding |
| **Sapur.003G035500** | isoform X1 | Myb_DNA-bind_6,Myb_DNA-binding |
| **Sapur.003G035500** | isoform X1 | Myb_DNA-bind_6,Myb_DNA-binding |
| **Sapur.003G035500** | isoform X1 | Myb_DNA-bind_6,Myb_DNA-binding |
| **Sapur.003G035500** | isoform X1 | Myb_DNA-bind_6,Myb_DNA-binding |
| **Sapur.003G035500** | isoform X1 | Myb_DNA-bind_6,Myb_DNA-binding |
| **Sapur.003G035500** | isoform X1 | Myb_DNA-bind_6,Myb_DNA-binding |
| **Sapur.003G035500** | isoform X1 | Myb_DNA-bind_6,Myb_DNA-binding |
| **Sapur.003G035500** | isoform X1 | Myb_DNA-bind_6,Myb_DNA-binding |
| **Sapur.003G035500** | isoform X1 | Myb_DNA-bind_6,Myb_DNA-binding |
| **Sapur.003G035500** | isoform X1 | Myb_DNA-bind_6,Myb_DNA-binding |
| **Sapur.003G035500** | isoform X1 | Myb_DNA-bind_6,Myb_DNA-binding |
| **Sapur.003G036400** | transcription factor | Myb_DNA-binding |
| **Sapur.003G044200** | PLATZ transcription factor | PLATZ |
| **Sapur.003G047600** | transcription factor | Myb_DNA-binding |
| **Sapur.003G047900** | Homeobox-leucine zipper protein | HALZ,HD-ZIP_N,Homeobox |
| **Sapur.003G048100** | Homeobox-leucine zipper protein | HALZ,HD-ZIP_N,Homeobox |
| **Sapur.003G060100** | PLATZ transcription factor family protein | PLATZ |
| **Sapur.003G063100** | transcription factor | Myb_DNA-binding |
| **Sapur.003G063100** | transcription factor | Myb_DNA-binding |
| **Sapur.003G063100** | transcription factor | Myb_DNA-binding |

| | | |
|---|---|---|
| **Sapur.003G072800** | Lipoxygenase homology domain-containing protein 1-like | ATS3,PLAT |
| **Sapur.003G083100** | Myb/SANT-like DNA-binding domain | Myb_DNA-bind_4 |
| **Sapur.003G106900** | SANT SWI3, ADA2, N-CoR and TFIIIB'' DNA-binding domains | Myb_DNA-binding |
| **Sapur.003G115100** | Transcription factor | Myb_DNA-binding |
| **Sapur.003G120700** | Myb/SANT-like DNA-binding domain | Myb_DNA-bind_4 |
| **Sapur.003G125700** | SANT SWI3, ADA2, N-CoR and TFIIIB'' DNA-binding domains | Myb_DNA-bind_6,Myb_DNA-binding |
| **Sapur.003G125700** | SANT SWI3, ADA2, N-CoR and TFIIIB'' DNA-binding domains | Myb_DNA-bind_6,Myb_DNA-binding |
| **Sapur.003G145100** | transcription factor | Myb_DNA-binding |
| **Sapur.003G145100** | transcription factor | Myb_DNA-binding |
| **Sapur.003G145100** | transcription factor | Myb_DNA-binding |
| **Sapur.003G145100** | transcription factor | Myb_DNA-binding |
| **Sapur.003G145200** | dnaJ homolog subfamily C member | DnaJ,Myb_DNA-binding |
| **Sapur.003G145200** | dnaJ homolog subfamily C member | DnaJ,Myb_DNA-binding |
| **Sapur.003G145900** | Nascent polypeptide-associated complex subunit alpha-like protein | NAC |
| **Sapur.003G145900** | Nascent polypeptide-associated complex subunit alpha-like protein | NAC |
| **Sapur.003G146000** | Myb/SANT-like DNA-binding domain | Myb_DNA-bind_3 |
| **Sapur.003G151200** | Myb/SANT-like DNA-binding domain | Myb_DNA-bind_4 |
| **Sapur.003G154400** | Myb/SANT-like DNA-binding domain | Myb_DNA-bind_4 |
| **Sapur.003G157300** | Myb/SANT-like DNA-binding domain | Myb_DNA-bind_4 |
| **Sapur.003G166600** | transcription repressor | Myb_DNA-binding |
| **Sapur.004G008600** | SANT SWI3, ADA2, N-CoR and TFIIIB'' DNA-binding | Myb_DNA-binding |

| | | |
|---|---|---|
| | domains | |
| **Sapur.004G008600** | SANT SWI3, ADA2, N-CoR and TFIIIB'' DNA-binding domains | Myb_DNA-binding |
| **Sapur.004G013400** | MYB-like transcription factor ETC1 | Myb_DNA-binding |
| **Sapur.004G013400** | MYB-like transcription factor ETC1 | Myb_DNA-binding |
| **Sapur.004G023800** | bromo domain | Bromodomain,Myb_DNA-binding |
| **Sapur.004G040000** | Myb-like DNA-binding domain | Myb_DNA-binding |
| **Sapur.004G040000** | Myb-like DNA-binding domain | Myb_DNA-binding |
| **Sapur.004G051100** | Transcription factor ASG4-like | Myb_DNA-binding |
| **Sapur.004G051100** | Transcription factor ASG4-like | Myb_DNA-binding |
| **Sapur.004G051900** | SANT SWI3, ADA2, N-CoR and TFIIIB'' DNA-binding domains | Myb_DNA-binding |
| **Sapur.004G051900** | SANT SWI3, ADA2, N-CoR and TFIIIB'' DNA-binding domains | Myb_DNA-binding |
| **Sapur.004G054900** | Nascent polypeptide-associated complex subunit beta | NAC |
| **Sapur.004G054900** | Nascent polypeptide-associated complex subunit beta | NAC |
| **Sapur.004G057900** | transcription | Myb_DNA-binding |
| **Sapur.004G062500** | SANT SWI3, ADA2, N-CoR and TFIIIB'' DNA-binding domains | Myb_DNA-binding |
| **Sapur.004G070900** | SANT SWI3, ADA2, N-CoR and TFIIIB'' DNA-binding domains | Myb_DNA-binding |
| **Sapur.004G070900** | SANT SWI3, ADA2, N-CoR and TFIIIB'' DNA-binding domains | Myb_DNA-binding |
| **Sapur.004G070900** | SANT SWI3, ADA2, N-CoR and TFIIIB'' DNA-binding domains | Myb_DNA-binding |
| **Sapur.004G070900** | SANT SWI3, ADA2, N-CoR | Myb_DNA-binding |

| | | |
|---|---|---|
| | and TFIIIB'' DNA-binding domains | |
| **Sapur.004G070900** | SANT SWI3, ADA2, N-CoR and TFIIIB'' DNA-binding domains | Myb_DNA-binding |
| **Sapur.004G098900** | Myb/SANT-like DNA-binding domain | Myb_DNA-bind_3 |
| **Sapur.004G104600** | Myb-related protein | Myb_DNA-binding |
| **Sapur.004G104600** | Myb-related protein | Myb_DNA-binding |
| **Sapur.004G108100** | Myb-like DNA-binding domain | Myb_DNA-binding |
| **Sapur.004G115100** | SWI SNF complex subunit | Myb_DNA-binding,SWIRM,SWIRM-assoc_1,ZZ |
| **Sapur.004G115100** | SWI SNF complex subunit | Myb_DNA-binding,SWIRM,SWIRM-assoc_1,ZZ |
| **Sapur.004G129400** | Myb/SANT-like DNA-binding domain | Myb_DNA-bind_3 |
| **Sapur.004G130600** | Myb-related protein 308-like | Myb_DNA-binding |
| **Sapur.004G130800** | lipid transport | C2,SMP_LBD |
| **Sapur.004G168000** | Myb-like DNA-binding domain | Myb_DNA-binding |
| **Sapur.005G000800** | Transcription factor | Myb_DNA-binding |
| **Sapur.005G000800** | Transcription factor | Myb_DNA-binding |
| **Sapur.005G022000** | Protein ALWAYS EARLY | DIRP,Myb_DNA-binding |
| **Sapur.005G022000** | Protein ALWAYS EARLY | DIRP,Myb_DNA-binding |
| **Sapur.005G022100** | Protein ALWAYS EARLY | DIRP,Myb_DNA-binding |
| **Sapur.005G022400** | Protein ALWAYS EARLY | DIRP,Myb_DNA-binding |
| **Sapur.005G026700** | adenylyltransferase | CTP_transf_like,Myb_DNA-bind_4 |
| **Sapur.005G026700** | adenylyltransferase | CTP_transf_like,Myb_DNA-bind_4 |
| **Sapur.005G026800** | Plant lipoxygenase may be involved in a number of diverse aspects of plant physiology including growth and development, pest resistance, and senescence or responses to wounding | Lipoxygenase,PLAT |
| **Sapur.005G026900** | Plant lipoxygenase may be involved in a number of diverse aspects of plant physiology including growth and development, pest resistance, and senescence or | Lipoxygenase,PLAT |

| | | |
|---|---|---|
| | responses to wounding | |
| **Sapur.005G040000** | Trihelix transcription factor | Myb_DNA-bind_4 |
| **Sapur.005G069400** | Transcription factor | Myb_DNA-binding |
| **Sapur.005G069400** | Transcription factor | Myb_DNA-binding |
| **Sapur.005G069400** | Transcription factor | Myb_DNA-binding |
| **Sapur.005G069400** | Transcription factor | Myb_DNA-binding |
| **Sapur.005G088700** | Cell cycle checkpoint protein | Myb_DNA-bind_3,Rad17 |
| **Sapur.005G089500** | Myb-related protein | Myb_DNA-binding |
| **Sapur.005G105800** | Myb-like DNA-binding domain | Myb_DNA-binding |
| **Sapur.005G111000** | SANT SWI3, ADA2, N-CoR and TFIIIB'' DNA-binding domains | Myb_DNA-binding |
| **Sapur.005G111000** | SANT SWI3, ADA2, N-CoR and TFIIIB'' DNA-binding domains | Myb_DNA-binding |
| **Sapur.005G111000** | SANT SWI3, ADA2, N-CoR and TFIIIB'' DNA-binding domains | Myb_DNA-binding |
| **Sapur.005G111000** | SANT SWI3, ADA2, N-CoR and TFIIIB'' DNA-binding domains | Myb_DNA-binding |
| **Sapur.005G111000** | SANT SWI3, ADA2, N-CoR and TFIIIB'' DNA-binding domains | Myb_DNA-binding |
| **Sapur.005G124400** | transcription factor | Myb_DNA-binding |
| **Sapur.005G142000** | SWI SNF complex subunit | Myb_DNA-binding,SWIRM,SWIRM-assoc_1 |
| **Sapur.005G142000** | SWI SNF complex subunit | Myb_DNA-binding,SWIRM,SWIRM-assoc_1 |
| **Sapur.005G151000** | Myb/SANT-like DNA-binding domain | Myb_DNA-bind_4 |
| **Sapur.005G151100** | Trihelix transcription factor | Myb_DNA-bind_4 |
| **Sapur.005G182200** | RADIALIS-like | Myb_DNA-binding |
| **Sapur.005G182200** | RADIALIS-like | Myb_DNA-binding |
| **Sapur.005G193800** | Synaptotagmin-like mitochondrial-lipid-binding domain | C2,SMP_LBD |
| **Sapur.005G202400** | Encoded by | Myb_DNA-bind_3 |
| **Sapur.006G000300** | transcription factor | Myb_CC_LHEQLE,Myb_DNA-binding |
| **Sapur.006G000300** | transcription factor | Myb_CC_LHEQLE,Myb_DNA-binding |
| **Sapur.006G000300** | transcription factor | Myb_CC_LHEQLE,Myb_DNA- |

| | | |
|---|---|---|
| | | binding |
| **Sapur.006G000300** | transcription factor | Myb_CC_LHEQLE,Myb_DNA-binding |
| **Sapur.006G000300** | transcription factor | Myb_CC_LHEQLE,Myb_DNA-binding |
| **Sapur.006G000300** | transcription factor | Myb_CC_LHEQLE,Myb_DNA-binding |
| **Sapur.006G012500** | Transcriptional adapter | Myb_DNA-binding,ZZ |
| **Sapur.006G022500** | Nascent polypeptide-associated complex subunit alpha-like | NAC |
| **Sapur.006G025000** | Myb-like DNA-binding domain | Myb_DNA-binding |
| **Sapur.006G041900** | PHD zinc finger | Myb_DNA-bind_6,Myb_DNA-binding |
| **Sapur.006G053800** | SANT SWI3, ADA2, N-CoR and TFIIIB'' DNA-binding domains | Myb_DNA-binding |
| **Sapur.006G069700** | Myb-related protein | Myb_DNA-binding |
| **Sapur.006G070000** | transcription factor | Myb_DNA-binding |
| **Sapur.006G070000** | transcription factor | Myb_DNA-binding |
| **Sapur.006G070000** | transcription factor | Myb_DNA-binding |
| **Sapur.006G070000** | transcription factor | Myb_DNA-binding |
| **Sapur.006G096400** | Alcohol dehydrogenase transcription factor Myb/SANT-like | AA_kinase,Myb_DNA-bind_4 |
| **Sapur.006G096400** | Alcohol dehydrogenase transcription factor Myb/SANT-like | AA_kinase,Myb_DNA-bind_4 |
| **Sapur.006G096400** | Alcohol dehydrogenase transcription factor Myb/SANT-like | AA_kinase,Myb_DNA-bind_4 |
| **Sapur.006G096600** | Myb/SANT-like DNA-binding domain | Myb_DNA-bind_4 |
| **Sapur.006G103100** | TSL-kinase interacting protein | Myb_DNA-binding |
| **Sapur.006G103100** | TSL-kinase interacting protein | Myb_DNA-binding |
| **Sapur.006G103100** | TSL-kinase interacting protein | Myb_DNA-binding |
| **Sapur.006G103100** | TSL-kinase interacting protein | Myb_DNA-binding |
| **Sapur.006G103100** | TSL-kinase interacting protein | Myb_DNA-binding |
| **Sapur.006G103100** | TSL-kinase interacting protein | Myb_DNA-binding |
| **Sapur.006G103100** | TSL-kinase interacting protein | Myb_DNA-binding |
| **Sapur.006G103900** | SANT SWI3, ADA2, N-CoR and TFIIIB'' DNA-binding domains | Myb_DNA-bind_6,Myb_DNA-binding |

| | | |
|---|---|---|
| **Sapur.006G103900** | SANT SWI3, ADA2, N-CoR and TFIIIB'' DNA-binding domains | Myb_DNA-bind_6,Myb_DNA-binding |
| **Sapur.006G103900** | SANT SWI3, ADA2, N-CoR and TFIIIB'' DNA-binding domains | Myb_DNA-bind_6,Myb_DNA-binding |
| **Sapur.006G103900** | SANT SWI3, ADA2, N-CoR and TFIIIB'' DNA-binding domains | Myb_DNA-bind_6,Myb_DNA-binding |
| **Sapur.006G103900** | SANT SWI3, ADA2, N-CoR and TFIIIB'' DNA-binding domains | Myb_DNA-bind_6,Myb_DNA-binding |
| **Sapur.006G109700** | SANT SWI3, ADA2, N-CoR and TFIIIB'' DNA-binding domains | Myb_DNA-binding |
| **Sapur.006G109700** | SANT SWI3, ADA2, N-CoR and TFIIIB'' DNA-binding domains | Myb_DNA-binding |
| **Sapur.006G121900** | Single myb histone | Linker_histone,Myb_DNA-bind_6,Myb_DNA-binding |
| **Sapur.006G127800** | Myb/SANT-like DNA-binding domain | Myb_DNA-bind_4 |
| **Sapur.006G127900** | Trihelix transcription factor | Myb_DNA-bind_4 |
| **Sapur.006G131300** | Myb-like DNA-binding domain | Myb_DNA-binding |
| **Sapur.006G153400** | two-component response regulator | Myb_DNA-binding,Response_reg |
| **Sapur.006G153400** | two-component response regulator | Myb_DNA-binding,Response_reg |
| **Sapur.006G155400** | Transcription factor | Myb_CC_LHEQLE,Myb_DNA-binding |
| **Sapur.006G155400** | Transcription factor | Myb_CC_LHEQLE,Myb_DNA-binding |
| **Sapur.006G157800** | Homeobox-leucine zipper protein | HALZ,HD-ZIP_N,Homeobox |
| **Sapur.006G182300** | transcription factor | Myb_DNA-binding |
| **Sapur.006G182400** | Trihelix transcription factor | Myb_DNA-bind_4 |
| **Sapur.006G182400** | Trihelix transcription factor | Myb_DNA-bind_4 |
| **Sapur.006G182700** | transcription, DNA-templated | Myb_DNA-binding |
| **Sapur.006G193800** | SANT SWI3, ADA2, N-CoR and TFIIIB'' DNA-binding domains | Myb_DNA-binding |
| **Sapur.006G193800** | SANT SWI3, ADA2, N-CoR and TFIIIB'' DNA-binding | Myb_DNA-binding |

| | | |
|---|---|---|
| | domains | |
| **Sapur.006G200000** | Myb-related protein | Myb_DNA-binding |
| **Sapur.006G200000** | Myb-related protein | Myb_DNA-binding |
| **Sapur.006G200000** | Myb-related protein | Myb_DNA-binding |
| **Sapur.006G200000** | Myb-related protein | Myb_DNA-binding |
| **Sapur.006G216200** | two-component response regulator | Myb_DNA-binding,Response_reg |
| **Sapur.006G216200** | two-component response regulator | Myb_DNA-binding,Response_reg |
| **Sapur.006G219700** | Myb/SANT-like DNA-binding domain | Myb_DNA-bind_3 |
| **Sapur.006G219700** | Myb/SANT-like DNA-binding domain | Myb_DNA-bind_3 |
| **Sapur.006G219700** | Myb/SANT-like DNA-binding domain | Myb_DNA-bind_3 |
| **Sapur.006G229900** | - | Myb_DNA-bind_4 |
| **Sapur.006G231000** | - | Myb_DNA-binding |
| **Sapur.007G002400** | MYB-CC type transfactor, LHEQLE motif | Myb_CC_LHEQLE,Myb_DNA-binding |
| **Sapur.007G002400** | MYB-CC type transfactor, LHEQLE motif | Myb_CC_LHEQLE,Myb_DNA-binding |
| **Sapur.007G002400** | MYB-CC type transfactor, LHEQLE motif | Myb_CC_LHEQLE,Myb_DNA-binding |
| **Sapur.007G002400** | MYB-CC type transfactor, LHEQLE motif | Myb_CC_LHEQLE,Myb_DNA-binding |
| **Sapur.007G002400** | MYB-CC type transfactor, LHEQLE motif | Myb_CC_LHEQLE,Myb_DNA-binding |
| **Sapur.007G021100** | radialis-like | Myb_DNA-binding |
| **Sapur.007G036700** | Myb-like DNA-binding domain | Myb_DNA-binding |
| **Sapur.007G036700** | Myb-like DNA-binding domain | Myb_DNA-binding |
| **Sapur.007G046000** | transcription factor | Myb_DNA-binding |
| **Sapur.007G082800** | Lipoxygenase homology domain-containing protein | PLAT |
| **Sapur.007G082900** | Lipoxygenase homology domain-containing protein | PLAT |
| **Sapur.007G109700** | SANT SWI3, ADA2, N-CoR and TFIIIB'' DNA-binding domains | Myb_DNA-binding |
| **Sapur.008G007800** | adenylyltransferase | CTP_transf_like,Myb_DNA-bind_4 |
| **Sapur.008G008700** | Chlorophyll(Ide) b reductase NOL | Myb_DNA-binding,adh_short |

| | | |
|---|---|---|
| **Sapur.008G008700** | Chlorophyll(Ide) b reductase NOL | Myb_DNA-binding,adh_short |
| **Sapur.008G008700** | Chlorophyll(Ide) b reductase NOL | Myb_DNA-binding,adh_short |
| **Sapur.008G017300** | Trihelix transcription factor | Myb_DNA-bind_4 |
| **Sapur.008G031600** | SANT SWI3, ADA2, N-CoR and TFIIIB'' DNA-binding domains | Myb_DNA-binding |
| **Sapur.008G031600** | SANT SWI3, ADA2, N-CoR and TFIIIB'' DNA-binding domains | Myb_DNA-binding |
| **Sapur.008G050900** | transcription factor | Myb_DNA-binding |
| **Sapur.008G056500** | Myb/SANT-like DNA-binding domain | Myb_DNA-bind_4 |
| **Sapur.008G056500** | Myb/SANT-like DNA-binding domain | Myb_DNA-bind_4 |
| **Sapur.008G060700** | transcription factor | Myb_DNA-binding |
| **Sapur.008G065100** | Myb family transcription factor APL-like | Myb_CC_LHEQLE,Myb_DNA-binding |
| **Sapur.008G069000** | Myb family transcription factor | Myb_CC_LHEQLE,Myb_DNA-binding |
| **Sapur.008G069000** | Myb family transcription factor | Myb_CC_LHEQLE,Myb_DNA-binding |
| **Sapur.008G074300** | SANT SWI3, ADA2, N-CoR and TFIIIB'' DNA-binding domains | Myb_DNA-binding |
| **Sapur.008G074300** | SANT SWI3, ADA2, N-CoR and TFIIIB'' DNA-binding domains | Myb_DNA-binding |
| **Sapur.008G080000** | Transcription factor | Myb_DNA-binding |
| **Sapur.008G103400** | SANT SWI3, ADA2, N-CoR and TFIIIB'' DNA-binding domains | Myb_DNA-binding |
| **Sapur.008G109700** | Cleavage and polyadenylation specificity factor | Beta-Casp,Lactamase_B_6,RMMBL |
| **Sapur.008G109700** | Cleavage and polyadenylation specificity factor | Beta-Casp,Lactamase_B_6,RMMBL |
| **Sapur.008G109700** | Cleavage and polyadenylation specificity factor | Beta-Casp,Lactamase_B_6,RMMBL |
| **Sapur.008G110200** | two-component response regulator | Myb_DNA-binding,Response_reg |

| | | |
|---|---|---|
| **Sapur.008G111600** | PLATZ transcription factor | PLATZ,zf-B_box |
| **Sapur.008G111600** | PLATZ transcription factor | PLATZ,zf-B_box |
| **Sapur.008G111600** | PLATZ transcription factor | PLATZ,zf-B_box |
| **Sapur.008G119900** | SANT SWI3, ADA2, N-CoR and TFIIIB'' DNA-binding domains | Myb_DNA-binding |
| **Sapur.008G119900** | SANT SWI3, ADA2, N-CoR and TFIIIB'' DNA-binding domains | Myb_DNA-binding |
| **Sapur.008G119900** | SANT SWI3, ADA2, N-CoR and TFIIIB'' DNA-binding domains | Myb_DNA-binding |
| **Sapur.008G119900** | SANT SWI3, ADA2, N-CoR and TFIIIB'' DNA-binding domains | Myb_DNA-binding |
| **Sapur.008G119900** | SANT SWI3, ADA2, N-CoR and TFIIIB'' DNA-binding domains | Myb_DNA-binding |
| **Sapur.008G122500** | Plant lipoxygenase may be involved in a number of diverse aspects of plant physiology including growth and development, pest resistance, and senescence or responses to wounding | Lipoxygenase,PLAT |
| **Sapur.008G122500** | Plant lipoxygenase may be involved in a number of diverse aspects of plant physiology including growth and development, pest resistance, and senescence or responses to wounding | Lipoxygenase,PLAT |
| **Sapur.008G122500** | Plant lipoxygenase may be involved in a number of diverse aspects of plant physiology including growth and development, pest resistance, and senescence or responses to wounding | Lipoxygenase,PLAT |
| **Sapur.008G122500** | Plant lipoxygenase may be involved in a number of diverse aspects of plant physiology including growth and development, pest | Lipoxygenase,PLAT |

| | | |
|---|---|---|
| | resistance, and senescence or responses to wounding | |
| **Sapur.008G122500** | Plant lipoxygenase may be involved in a number of diverse aspects of plant physiology including growth and development, pest resistance, and senescence or responses to wounding | Lipoxygenase,PLAT |
| **Sapur.008G122500** | Plant lipoxygenase may be involved in a number of diverse aspects of plant physiology including growth and development, pest resistance, and senescence or responses to wounding | Lipoxygenase,PLAT |
| **Sapur.008G135200** | Myb-related protein Myb4-like | Myb_DNA-binding |
| **Sapur.008G135200** | Myb-related protein Myb4-like | Myb_DNA-binding |
| **Sapur.008G141000** | transcription factor | Myb_DNA-binding |
| **Sapur.008G143900** | Plant lipoxygenase may be involved in a number of diverse aspects of plant physiology including growth and development, pest resistance, and senescence or responses to wounding | Lipoxygenase,PLAT |
| **Sapur.008G143900** | Plant lipoxygenase may be involved in a number of diverse aspects of plant physiology including growth and development, pest resistance, and senescence or responses to wounding | Lipoxygenase,PLAT |
| **Sapur.008G145400** | Trihelix transcription factor | Myb_DNA-bind_4 |
| **Sapur.008G145400** | Trihelix transcription factor | Myb_DNA-bind_4 |
| **Sapur.008G145400** | Trihelix transcription factor | Myb_DNA-bind_4 |
| **Sapur.008G145400** | Trihelix transcription factor | Myb_DNA-bind_4 |
| **Sapur.008G146300** | Myb-related protein | Myb_DNA-binding |
| **Sapur.008G146500** | two-component response regulator | Myb_DNA-binding,Response_reg |
| **Sapur.008G146500** | two-component response regulator | Myb_DNA-binding,Response_reg |

| | | |
|---|---|---|
| **Sapur.008G146500** | two-component response regulator | Myb_DNA-binding,Response_reg |
| **Sapur.008G154300** | Transcription factor | Myb_DNA-binding |
| **Sapur.008G154300** | Transcription factor | Myb_DNA-binding |
| **Sapur.008G158900** | Two-component response regulator | Myb_DNA-binding,Response_reg |
| **Sapur.008G164600** | Protein ALWAYS EARLY | DIRP,Myb_DNA-binding |
| **Sapur.008G164600** | Protein ALWAYS EARLY | DIRP,Myb_DNA-binding |
| **Sapur.008G164600** | Protein ALWAYS EARLY | DIRP,Myb_DNA-binding |
| **Sapur.008G164600** | Protein ALWAYS EARLY | DIRP,Myb_DNA-binding |
| **Sapur.009G001200** | PLATZ transcription factor | PLATZ |
| **Sapur.009G001200** | PLATZ transcription factor | PLATZ |
| **Sapur.009G010800** | Transcription factor | Myb_DNA-binding |
| **Sapur.009G010800** | Transcription factor | Myb_DNA-binding |
| **Sapur.009G019900** | transcription factor | Myb_DNA-binding |
| **Sapur.009G019900** | transcription factor | Myb_DNA-binding |
| **Sapur.009G019900** | transcription factor | Myb_DNA-binding |
| **Sapur.009G026000** | transcription factor | Myb_DNA-binding |
| **Sapur.009G026000** | transcription factor | Myb_DNA-binding |
| **Sapur.009G026000** | transcription factor | Myb_DNA-binding |
| **Sapur.009G026000** | transcription factor | Myb_DNA-binding |
| **Sapur.009G026000** | transcription factor | Myb_DNA-binding |
| **Sapur.009G026000** | transcription factor | Myb_DNA-binding |
| **Sapur.009G026000** | transcription factor | Myb_DNA-binding |
| **Sapur.009G030400** | Myb SANT-like DNA-binding domain protein | Myb_DNA-bind_3 |
| **Sapur.009G031600** | transcription factor | Myb_DNA-binding |
| **Sapur.009G047500** | transcription factor | Myb_DNA-binding |
| **Sapur.009G068200** | Telomere repeat-binding factor | Linker_histone,Myb_DNA-binding |
| **Sapur.009G068200** | Telomere repeat-binding factor | Linker_histone,Myb_DNA-binding |
| **Sapur.009G068200** | Telomere repeat-binding factor | Linker_histone,Myb_DNA-binding |
| **Sapur.009G074900** | Myb DNA-binding like | Myb_DNA-bind_7 |
| **Sapur.009G083700** | Myb-like DNA-binding domain | Myb_DNA-binding |
| **Sapur.009G083700** | Myb-like DNA-binding domain | Myb_DNA-binding |
| **Sapur.009G083700** | Myb-like DNA-binding domain | Myb_DNA-binding |
| **Sapur.009G090800** | SWI SNF complex subunit | Myb_DNA-binding,SWIRM,SWIRM-assoc_1,ZZ |
| **Sapur.009G092200** | SANT SWI3, ADA2, N-CoR | Myb_DNA-binding |

| | | |
|---|---|---|
| | and TFIIIB'' DNA-binding domains | |
| **Sapur.009G106800** | lipid transport | C2,SMP_LBD |
| **Sapur.009G106800** | lipid transport | C2,SMP_LBD |
| **Sapur.010G000200** | Two-component response regulator | Myb_DNA-binding,Response_reg |
| **Sapur.010G000200** | Two-component response regulator | Myb_DNA-binding,Response_reg |
| **Sapur.010G000200** | Two-component response regulator | Myb_DNA-binding,Response_reg |
| **Sapur.010G016600** | Protein ALWAYS EARLY | DIRP,Myb_DNA-binding |
| **Sapur.010G016600** | Protein ALWAYS EARLY | DIRP,Myb_DNA-binding |
| **Sapur.010G016600** | Protein ALWAYS EARLY | DIRP,Myb_DNA-binding |
| **Sapur.010G016600** | Protein ALWAYS EARLY | DIRP,Myb_DNA-binding |
| **Sapur.010G017500** | Myb DNA-binding like | Myb_DNA-bind_7 |
| **Sapur.010G017500** | Myb DNA-binding like | Myb_DNA-bind_7 |
| **Sapur.010G017500** | Myb DNA-binding like | Myb_DNA-bind_7 |
| **Sapur.010G017500** | Myb DNA-binding like | Myb_DNA-bind_7 |
| **Sapur.010G017500** | Myb DNA-binding like | Myb_DNA-bind_7 |
| **Sapur.010G017500** | Myb DNA-binding like | Myb_DNA-bind_7 |
| **Sapur.010G017500** | Myb DNA-binding like | Myb_DNA-bind_7 |
| **Sapur.010G017500** | Myb DNA-binding like | Myb_DNA-bind_7 |
| **Sapur.010G017500** | Myb DNA-binding like | Myb_DNA-bind_7 |
| **Sapur.010G017500** | Myb DNA-binding like | Myb_DNA-bind_7 |
| **Sapur.010G017500** | Myb DNA-binding like | Myb_DNA-bind_7 |
| **Sapur.010G017500** | Myb DNA-binding like | Myb_DNA-bind_7 |
| **Sapur.010G017600** | Myb DNA-binding like | Myb_DNA-bind_7 |
| **Sapur.010G018800** | two-component response regulator | Myb_DNA-binding,Response_reg |
| **Sapur.010G030400** | Transcription factor | Myb_DNA-binding |
| **Sapur.010G036700** | Myb/SANT-like DNA-binding domain | Myb_DNA-bind_3 |
| **Sapur.010G047900** | transcription factor | Myb_DNA-binding |
| **Sapur.010G056500** | Trihelix transcription factor | Myb_DNA-bind_4 |
| **Sapur.010G056500** | Trihelix transcription factor | Myb_DNA-bind_4 |
| **Sapur.010G061900** | PHD - plant homeodomain finger protein | Myb_DNA-bind_3,PHD_Oberon |
| **Sapur.010G061900** | PHD - plant homeodomain finger protein | Myb_DNA-bind_3,PHD_Oberon |
| **Sapur.010G061900** | PHD - plant homeodomain finger protein | Myb_DNA-bind_3,PHD_Oberon |
| **Sapur.010G061900** | PHD - plant homeodomain finger protein | Myb_DNA-bind_3,PHD_Oberon |
| **Sapur.010G061900** | PHD - plant homeodomain | Myb_DNA-bind_3,PHD_Obero |

| | | |
|---|---|---|
| | finger protein | n |
| **Sapur.010G061900** | PHD - plant homeodomain finger protein | Myb_DNA-bind_3,PHD_Obero n |
| **Sapur.010G061900** | PHD - plant homeodomain finger protein | Myb_DNA-bind_3,PHD_Obero n |
| **Sapur.010G061900** | PHD - plant homeodomain finger protein | Myb_DNA-bind_3,PHD_Obero n |
| **Sapur.010G069000** | Cleavage and polyadenylation specificity factor subunit | Beta-Casp,Lactamase_B_6,RMM BL,zf-RING_2 |
| **Sapur.010G069000** | Cleavage and polyadenylation specificity factor subunit | Beta-Casp,Lactamase_B_6,RMM BL,zf-RING_2 |
| **Sapur.010G069500** | two-component response regulator | Myb_DNA-binding,Response_r eg |
| **Sapur.010G069500** | two-component response regulator | Myb_DNA-binding,Response_r eg |
| **Sapur.010G069500** | two-component response regulator | Myb_DNA-binding,Response_r eg |
| **Sapur.010G069500** | two-component response regulator | Myb_DNA-binding,Response_r eg |
| **Sapur.010G069500** | two-component response regulator | Myb_DNA-binding,Response_r eg |
| **Sapur.010G069500** | two-component response regulator | Myb_DNA-binding,Response_r eg |
| **Sapur.010G079600** | SANT SWI3, ADA2, N-CoR and TFIIIB'' DNA-binding domains | Myb_DNA-binding |
| **Sapur.010G079600** | SANT SWI3, ADA2, N-CoR and TFIIIB'' DNA-binding domains | Myb_DNA-binding |
| **Sapur.010G086400** | SANT SWI3, ADA2, N-CoR and TFIIIB'' DNA-binding domains | Myb_DNA-binding |
| **Sapur.010G094000** | Transcription factor | Myb_DNA-binding |
| **Sapur.010G099200** | transcription factor | Myb_DNA-binding |
| **Sapur.010G123000** | SANT SWI3, ADA2, N-CoR and TFIIIB'' DNA-binding domains | Myb_DNA-binding |
| **Sapur.010G135000** | Myb family transcription factor APL-like | Myb_CC_LHEQLE,Myb_DNA-binding |
| **Sapur.010G135000** | Myb family transcription factor APL-like | Myb_CC_LHEQLE,Myb_DNA-binding |
| **Sapur.010G135000** | Myb family transcription | Myb_CC_LHEQLE,Myb_DNA- |

| | | |
|---|---|---|
| | factor APL-like | binding |
| **Sapur.010G135000** | Myb family transcription factor APL-like | Myb_CC_LHEQLE,Myb_DNA-binding |
| **Sapur.010G135000** | Myb family transcription factor APL-like | Myb_CC_LHEQLE,Myb_DNA-binding |
| **Sapur.010G151300** | transcription factor | Myb_DNA-binding |
| **Sapur.010G152600** | SANT SWI3, ADA2, N-CoR and TFIIIB'' DNA-binding domains | Myb_DNA-binding |
| **Sapur.010G175400** | SANT SWI3, ADA2, N-CoR and TFIIIB'' DNA-binding domains | Myb_DNA-binding |
| **Sapur.010G186900** | Amino acid kinase family | AA_kinase,Myb_DNA-bind_4 |
| **Sapur.010G188100** | Trihelix transcription factor | Myb_DNA-bind_4 |
| **Sapur.010G194800** | transcription factor | Myb_DNA-binding |
| **Sapur.010G198600** | adenylyltransferase | CTP_transf_like,Myb_DNA-bind_4 |
| **Sapur.011G001800** | - | Myb_DNA-bind_6 |
| **Sapur.011G002000** | - | Myb_DNA-bind_6 |
| **Sapur.011G002000** | - | Myb_DNA-bind_6 |
| **Sapur.011G013100** | Myb family transcription factor APL-like | Myb_CC_LHEQLE,Myb_DNA-binding |
| **Sapur.011G015300** | MYB-like transcription factor ETC1 | Myb_DNA-binding |
| **Sapur.011G027000** | transcription factor | Myb_DNA-binding |
| **Sapur.011G059000** | SANT SWI3, ADA2, N-CoR and TFIIIB'' DNA-binding domains | Myb_DNA-binding |
| **Sapur.011G059000** | SANT SWI3, ADA2, N-CoR and TFIIIB'' DNA-binding domains | Myb_DNA-binding |
| **Sapur.011G059000** | SANT SWI3, ADA2, N-CoR and TFIIIB'' DNA-binding domains | Myb_DNA-binding |
| **Sapur.011G059000** | SANT SWI3, ADA2, N-CoR and TFIIIB'' DNA-binding domains | Myb_DNA-binding |
| **Sapur.011G059000** | SANT SWI3, ADA2, N-CoR and TFIIIB'' DNA-binding domains | Myb_DNA-binding |
| **Sapur.011G059000** | SANT SWI3, ADA2, N-CoR and TFIIIB'' DNA-binding domains | Myb_DNA-binding |
| **Sapur.011G059000** | SANT SWI3, ADA2, N-CoR | Myb_DNA-binding |

| | | |
|---|---|---|
| | and TFIIIB'' DNA-binding domains | |
| **Sapur.011G109300** | Trihelix transcription factor | Myb_DNA-bind_4 |
| **Sapur.011G109300** | Trihelix transcription factor | Myb_DNA-bind_4 |
| **Sapur.011G109300** | Trihelix transcription factor | Myb_DNA-bind_4 |
| **Sapur.011G109300** | Trihelix transcription factor | Myb_DNA-bind_4 |
| **Sapur.012G003100** | Nascent polypeptide-associated complex subunit alpha-like protein | NAC |
| **Sapur.012G003100** | Nascent polypeptide-associated complex subunit alpha-like protein | NAC |
| **Sapur.012G018700** | transcription factor | Myb_DNA-binding |
| **Sapur.012G018700** | transcription regulator recruiting activity | Myb_DNA-binding |
| **Sapur.012G024300** | Nascent polypeptide-associated complex subunit beta | NAC |
| **Sapur.012G024700** | transcription, DNA-templated | Myb_DNA-binding |
| **Sapur.012G024700** | transcription, DNA-templated | Myb_DNA-binding |
| **Sapur.012G024700** | transcription, DNA-templated | Myb_DNA-binding |
| **Sapur.012G027600** | Transcription repressor KAN1-like | Myb_DNA-binding |
| **Sapur.012G042600** | transcription factor | Myb_DNA-binding |
| **Sapur.012G043900** | AtCASP,CASP | CASP_C |
| **Sapur.012G053600** | transcription factor | Myb_DNA-binding |
| **Sapur.012G053600** | transcription factor | Myb_DNA-binding |
| **Sapur.012G062600** | transcription factor | Myb_DNA-binding |
| **Sapur.012G071900** | Zn-dependent metallo-hydrolase RNA specificity domain | Lactamase_B,Lactamase_B_2,Myb_DNA-bind_4,RMMBL |
| **Sapur.012G071900** | Zn-dependent metallo-hydrolase RNA specificity domain | Lactamase_B,Lactamase_B_2,Myb_DNA-bind_4,RMMBL |
| **Sapur.012G071900** | Zn-dependent metallo-hydrolase RNA specificity domain | Lactamase_B,Lactamase_B_2,Myb_DNA-bind_4,RMMBL |
| **Sapur.013G000900** | Transcription factor | Myb_DNA-binding |
| **Sapur.013G021600** | Protein ALWAYS EARLY | DIRP,Myb_DNA-binding |
| **Sapur.013G022800** | Plant lipoxygenase may be involved in a number of diverse aspects of plant | Lipoxygenase,PLAT |

| | | |
|---|---|---|
| | physiology including growth and development, pest resistance, and senescence or responses to wounding | |
| **Sapur.013G025100** | Myb/SANT-like DNA-binding domain | Myb_DNA-bind_3 |
| **Sapur.013G025100** | Myb/SANT-like DNA-binding domain | Myb_DNA-bind_3 |
| **Sapur.013G043000** | cell division cycle 5-like | Myb_Cef,Myb_DNA-binding |
| **Sapur.013G044700** | PHR1-LIKE 1-like | Myb_CC_LHEQLE,Myb_DNA-binding |
| **Sapur.013G044700** | PHR1-LIKE 1-like | Myb_CC_LHEQLE,Myb_DNA-binding |
| **Sapur.013G044700** | PHR1-LIKE 1-like | Myb_CC_LHEQLE,Myb_DNA-binding |
| **Sapur.013G044700** | PHR1-LIKE 1-like | Myb_CC_LHEQLE,Myb_DNA-binding |
| **Sapur.013G052300** | transcription repressor | Myb_DNA-binding |
| **Sapur.013G052400** | transcription repressor | Myb_DNA-binding |
| **Sapur.013G056000** | MYB-CC type transfactor, LHEQLE motif | Myb_CC_LHEQLE,Myb_DNA-binding |
| **Sapur.013G056000** | MYB-CC type transfactor, LHEQLE motif | Myb_CC_LHEQLE,Myb_DNA-binding |
| **Sapur.013G056000** | MYB-CC type transfactor, LHEQLE motif | Myb_CC_LHEQLE,Myb_DNA-binding |
| **Sapur.013G056000** | MYB-CC type transfactor, LHEQLE motif | Myb_CC_LHEQLE,Myb_DNA-binding |
| **Sapur.013G064700** | PLATZ transcription factor family protein | PLATZ |
| **Sapur.013G069100** | transcription factor | Myb_DNA-binding |
| **Sapur.013G069100** | transcription factor | Myb_DNA-binding |
| **Sapur.013G076300** | HSA | HSA,Myb_DNA-bind_6 |
| **Sapur.013G076400** | HSA | HSA,Myb_DNA-bind_6 |
| **Sapur.013G133100** | Encoded by | Myb_DNA-binding |
| **Sapur.013G138600** | SANT SWI3, ADA2, N-CoR and TFIIIB'' DNA-binding domains | DAO,Myb_DNA-bind_6,Myb_DNA-binding |
| **Sapur.013G138600** | SANT SWI3, ADA2, N-CoR and TFIIIB'' DNA-binding domains | DAO,Myb_DNA-bind_6,Myb_DNA-binding |
| **Sapur.014G002400** | Telomere repeat-binding factor | Linker_histone,Myb_DNA-binding |
| **Sapur.014G002400** | Telomere repeat-binding factor | Linker_histone,Myb_DNA-binding |

| | | |
|---|---|---|
| **Sapur.014G002400** | Telomere repeat-binding factor | Linker_histone,Myb_DNA-binding |
| **Sapur.014G002400** | Telomere repeat-binding factor | Linker_histone,Myb_DNA-binding |
| **Sapur.014G002400** | Telomere repeat-binding factor | Linker_histone,Myb_DNA-binding |
| **Sapur.014G002400** | Telomere repeat-binding factor | Linker_histone,Myb_DNA-binding |
| **Sapur.014G002400** | Telomere repeat-binding factor | Linker_histone,Myb_DNA-binding |
| **Sapur.014G002400** | Telomere repeat-binding factor | Linker_histone,Myb_DNA-binding |
| **Sapur.014G002400** | Telomere repeat-binding factor | Linker_histone,Myb_DNA-binding |
| **Sapur.014G002400** | Telomere repeat-binding factor | Linker_histone,Myb_DNA-binding |
| **Sapur.014G002400** | Telomere repeat-binding factor | Linker_histone,Myb_DNA-binding |
| **Sapur.014G002400** | Telomere repeat-binding factor | Linker_histone,Myb_DNA-binding |
| **Sapur.014G002400** | Telomere repeat-binding factor | Linker_histone,Myb_DNA-binding |
| **Sapur.014G002400** | Telomere repeat-binding factor | Linker_histone,Myb_DNA-binding |
| **Sapur.014G002400** | Telomere repeat-binding factor | Linker_histone,Myb_DNA-binding |
| **Sapur.014G002400** | Telomere repeat-binding factor | Linker_histone,Myb_DNA-binding |
| **Sapur.014G002400** | Telomere repeat-binding factor | Linker_histone,Myb_DNA-binding |
| **Sapur.014G012700** | transcription factor | Myb_DNA-binding |
| **Sapur.014G021400** | HSA | HSA,Myb_DNA-bind_6 |
| **Sapur.014G034500** | Homeobox-leucine zipper protein | HALZ,HD-ZIP_N,Homeobox |
| **Sapur.014G038700** | Alcohol dehydrogenase transcription factor Myb/SANT-like | Myb_DNA-bind_4 |
| **Sapur.014G045100** | PLATZ transcription factor | PLATZ |
| **Sapur.014G067000** | AT-rich interactive domain-containing protein | ARID,Myb_DNA-binding |
| **Sapur.014G067000** | AT-rich interactive domain-containing protein | ARID,Myb_DNA-binding |
| **Sapur.014G069400** | reveille | Myb_DNA-binding |
| **Sapur.014G069400** | reveille | Myb_DNA-binding |

| | | |
|---|---|---|
| **Sapur.014G069400** | reveille | Myb_DNA-binding |
| **Sapur.014G069400** | reveille | Myb_DNA-binding |
| **Sapur.014G069400** | reveille | Myb_DNA-binding |
| **Sapur.014G079200** | SANT SWI3, ADA2, N-CoR and TFIIIB'' DNA-binding domains | Myb_DNA-binding |
| **Sapur.014G084400** | Protein LHY-like isoform X1 | Myb_DNA-binding |
| **Sapur.014G084400** | Protein LHY-like isoform X1 | Myb_DNA-binding |
| **Sapur.014G084400** | Protein LHY-like isoform X1 | Myb_DNA-binding |
| **Sapur.014G084400** | Protein LHY-like isoform X1 | Myb_DNA-binding |
| **Sapur.014G084400** | Protein LHY-like isoform X1 | Myb_DNA-binding |
| **Sapur.014G084400** | Protein LHY-like isoform X1 | Myb_DNA-binding |
| **Sapur.014G084400** | Protein LHY-like isoform X1 | Myb_DNA-binding |
| **Sapur.014G084400** | Protein LHY-like isoform X1 | Myb_DNA-binding |
| **Sapur.014G084400** | Protein LHY-like isoform X1 | Myb_DNA-binding |
| **Sapur.014G093400** | transcription factor | Myb_DNA-binding |
| **Sapur.014G135700** | Transcriptional adapter | Myb_DNA-binding,ZZ |
| **Sapur.014G135700** | Transcriptional adapter | Myb_DNA-binding,ZZ |
| **Sapur.014G135700** | Transcriptional adapter | Myb_DNA-binding,ZZ |
| **Sapur.014G135700** | Transcriptional adapter | Myb_DNA-binding,ZZ |
| **Sapur.014G135700** | Transcriptional adapter | Myb_DNA-binding,ZZ |
| **Sapur.016G001700** | Myb family transcription factor | Myb_CC_LHEQLE,Myb_DNA-binding |
| **Sapur.016G001800** | transcription factor | Myb_CC_LHEQLE,Myb_DNA-binding |
| **Sapur.016G001800** | transcription factor | Myb_CC_LHEQLE,Myb_DNA-binding |
| **Sapur.016G001800** | transcription factor | Myb_CC_LHEQLE,Myb_DNA-binding |
| **Sapur.016G001800** | transcription factor | Myb_CC_LHEQLE,Myb_DNA-binding |
| **Sapur.016G005300** | Trihelix transcription factor | Myb_DNA-bind_4 |
| **Sapur.016G007100** | Transcriptional adapter | Myb_DNA-binding,ZZ |
| **Sapur.016G029600** | Myb-like DNA-binding domain | Myb_DNA-binding |
| **Sapur.016G042800** | Transcription factor | Myb_CC_LHEQLE,Myb_DNA-binding |
| **Sapur.016G042800** | Transcription factor | Myb_CC_LHEQLE,Myb_DNA-binding |
| **Sapur.016G042800** | Transcription factor | Myb_CC_LHEQLE,Myb_DNA-binding |
| **Sapur.016G042900** | Transcription factor | Myb_CC_LHEQLE,Myb_DNA-binding |
| **Sapur.016G043000** | RNA recognition motif. (a.k.a. | Myb_DNA-binding,RRM_1 |

| | | |
|---|---|---|
| | RRM, RBD, or RNP domain) | |
| **Sapur.016G047600** | PHD zinc finger | Myb_DNA-bind_6,Myb_DNA-binding |
| **Sapur.016G047600** | PHD zinc finger | Myb_DNA-bind_6,Myb_DNA-binding |
| **Sapur.016G050900** | Plant lipoxygenase may be involved in a number of diverse aspects of plant physiology including growth and development, pest resistance, and senescence or responses to wounding | Lipoxygenase,PLAT |
| **Sapur.016G051000** | Plant lipoxygenase may be involved in a number of diverse aspects of plant physiology including growth and development, pest resistance, and senescence or responses to wounding | Lipoxygenase,PLAT |
| **Sapur.016G051100** | adenylyltransferase | CTP_transf_like,Myb_DNA-bind_4 |
| **Sapur.016G051100** | adenylyltransferase | CTP_transf_like,Myb_DNA-bind_4 |
| **Sapur.016G051100** | adenylyltransferase | CTP_transf_like,Myb_DNA-bind_4 |
| **Sapur.016G051100** | adenylyltransferase | CTP_transf_like,Myb_DNA-bind_4 |
| **Sapur.016G080300** | SANT SWI3, ADA2, N-CoR and TFIIIB'' DNA-binding domains | Myb_DNA-binding |
| **Sapur.016G080300** | SANT SWI3, ADA2, N-CoR and TFIIIB'' DNA-binding domains | Myb_DNA-binding |
| **Sapur.016G080300** | SANT SWI3, ADA2, N-CoR and TFIIIB'' DNA-binding domains | Myb_DNA-binding |
| **Sapur.016G080300** | SANT SWI3, ADA2, N-CoR and TFIIIB'' DNA-binding domains | Myb_DNA-binding |
| **Sapur.016G080300** | SANT SWI3, ADA2, N-CoR and TFIIIB'' DNA-binding domains | Myb_DNA-binding |
| **Sapur.016G080300** | SANT SWI3, ADA2, N-CoR and TFIIIB'' DNA-binding | Myb_DNA-binding |

| | | |
|---|---|---|
| | domains | |
| **Sapur.016G086600** | TSL-kinase interacting protein | Myb_DNA-binding |
| **Sapur.016G089900** | Myb/SANT-like DNA-binding domain | Myb_DNA-bind_4 |
| **Sapur.016G097800** | transcription factor | Myb_DNA-binding |
| **Sapur.016G099900** | Myb/SANT-like DNA-binding domain | Myb_DNA-bind_3 |
| **Sapur.016G116600** | transcription factor | Myb_DNA-binding |
| **Sapur.016G120400** | Transcription factor | Myb_DNA-binding |
| **Sapur.016G123700** | Myb-like DNA-binding domain | Bromodomain,Myb_DNA-binding |
| **Sapur.016G123700** | Myb-like DNA-binding domain | Bromodomain,Myb_DNA-binding |
| **Sapur.016G123700** | Myb-like DNA-binding domain | Bromodomain,Myb_DNA-binding |
| **Sapur.016G123700** | Myb-like DNA-binding domain | Bromodomain,Myb_DNA-binding |
| **Sapur.016G125900** | Cleavage and polyadenylation specificity factor | Beta-Casp,CPSF100_C,Lactamase_B_6,RMMBL |
| **Sapur.016G129100** | transcription factor | Myb_DNA-binding |
| **Sapur.016G129100** | transcription factor | Myb_DNA-binding |
| **Sapur.016G129100** | transcription factor | Myb_DNA-binding |
| **Sapur.016G139100** | transcription factor | Myb_DNA-binding |
| **Sapur.016G174300** | Telomere repeat-binding factor | Linker_histone,Myb_DNA-binding |
| **Sapur.016G174300** | Telomere repeat-binding factor | Linker_histone,Myb_DNA-binding |
| **Sapur.016G174300** | Telomere repeat-binding factor | Linker_histone,Myb_DNA-binding |
| **Sapur.016G179900** | Transcription factor | Myb_DNA-binding |
| **Sapur.016G191500** | Myb family transcription factor APL | Myb_CC_LHEQLE,Myb_DNA-binding |
| **Sapur.016G191500** | Myb family transcription factor APL | Myb_CC_LHEQLE,Myb_DNA-binding |
| **Sapur.016G191500** | Myb family transcription factor APL | Myb_CC_LHEQLE,Myb_DNA-binding |
| **Sapur.016G191500** | Myb family transcription factor APL | Myb_CC_LHEQLE,Myb_DNA-binding |
| **Sapur.016G243500** | C2 domain-containing protein | C2,SMP_LBD |
| **Sapur.016G243500** | C2 domain-containing protein | C2,SMP_LBD |
| **Sapur.016G243500** | C2 domain-containing protein | C2,SMP_LBD |
| **Sapur.016G308100** | transcription factor | Myb_DNA-binding |
| **Sapur.017G011300** | Protein of unknown function | DUF3755,Myb_DNA-binding |

| | | |
|---|---|---|
| | (DUF3755) | |
| **Sapur.017G027900** | SANT SWI3, ADA2, N-CoR and TFIIIB'' DNA-binding domains | Myb_DNA-binding |
| **Sapur.017G063100** | Cleavage and polyadenylation specificity factor subunit | Beta-Casp,CPSF73-100_C,Lactamase_B,Lactamase_B_6,RMMBL |
| **Sapur.017G063100** | Cleavage and polyadenylation specificity factor subunit | Beta-Casp,CPSF73-100_C,Lactamase_B,Lactamase_B_6,RMMBL |
| **Sapur.017G063200** | Cleavage and polyadenylation specificity factor subunit | Beta-Casp,CPSF73-100_C,Lactamase_B,Lactamase_B_6,RMMBL |
| **Sapur.017G075500** | SANT SWI3, ADA2, N-CoR and TFIIIB'' DNA-binding domains | Myb_DNA-binding |
| **Sapur.017G075500** | SANT SWI3, ADA2, N-CoR and TFIIIB'' DNA-binding domains | Myb_DNA-binding |
| **Sapur.017G092900** | transcription factor | Myb_DNA-binding |
| **Sapur.017G100200** | Myb/SANT-like DNA-binding domain | Myb_DNA-bind_3 |
| **Sapur.017G100200** | Myb/SANT-like DNA-binding domain | Myb_DNA-bind_3 |
| **Sapur.017G100200** | Myb/SANT-like DNA-binding domain | Myb_DNA-bind_3 |
| **Sapur.017G100200** | Myb/SANT-like DNA-binding domain | Myb_DNA-bind_3 |
| **Sapur.017G100200** | Myb/SANT-like DNA-binding domain | Myb_DNA-bind_3 |
| **Sapur.017G106200** | SANT SWI3, ADA2, N-CoR and TFIIIB'' DNA-binding domains | Myb_DNA-binding |
| **Sapur.017G113000** | transcription | Myb_DNA-binding |
| **Sapur.017G115800** | Nascent polypeptide-associated complex subunit beta | NAC |
| **Sapur.017G122200** | Transcription factor ASG4-like | Myb_DNA-binding |
| **Sapur.017G122200** | Transcription factor ASG4-like | Myb_DNA-binding |
| **Sapur.017G124200** | SANT SWI3, ADA2, N-CoR and TFIIIB'' DNA-binding domains | Myb_DNA-binding |

| | | |
|---|---|---|
| **Sapur.018G000300** | - | Myb_DNA-binding |
| **Sapur.018G004800** | transcription factor | Myb_DNA-binding |
| **Sapur.018G016000** | two-component response regulator | Myb_DNA-binding,Response_reg |
| **Sapur.018G016400** | two-component response regulator | Myb_DNA-binding,Response_reg |
| **Sapur.018G016700** | two-component response regulator | Myb_DNA-binding,Response_reg |
| **Sapur.018G017100** | two-component response regulator | Myb_DNA-binding,Response_reg |
| **Sapur.018G017300** | two-component response regulator | Myb_DNA-binding,Response_reg |
| **Sapur.018G017400** | two-component response regulator | Myb_DNA-binding,Response_reg |
| **Sapur.018G017800** | two-component response regulator | Myb_DNA-binding,Response_reg |
| **Sapur.018G017800** | two-component response regulator | Myb_DNA-binding,Response_reg |
| **Sapur.018G017800** | two-component response regulator | Myb_DNA-binding,Response_reg |
| **Sapur.018G021400** | Synaptotagmin-like mitochondrial-lipid-binding domain | C2,SMP_LBD |
| **Sapur.018G031100** | Myb-related protein | Myb_DNA-binding |
| **Sapur.018G031100** | Myb-related protein | Myb_DNA-binding |
| **Sapur.018G037700** | transcription factor | Myb_DNA-binding |
| **Sapur.018G037700** | transcription factor | Myb_DNA-binding |
| **Sapur.018G037900** | Trihelix transcription factor | Myb_DNA-bind_4 |
| **Sapur.018G037900** | Trihelix transcription factor | Myb_DNA-bind_4 |
| **Sapur.018G047800** | Single myb histone | Linker_histone,Myb_DNA-bind_6,Myb_DNA-binding |
| **Sapur.018G047800** | Single myb histone | Linker_histone,Myb_DNA-bind_6,Myb_DNA-binding |
| **Sapur.018G047800** | Single myb histone | Linker_histone,Myb_DNA-bind_6,Myb_DNA-binding |
| **Sapur.018G047800** | Single myb histone | Linker_histone,Myb_DNA-bind_6,Myb_DNA-binding |
| **Sapur.018G047800** | Single myb histone | Linker_histone,Myb_DNA-bind_6,Myb_DNA-binding |
| **Sapur.018G053100** | Myb-like DNA-binding domain | Myb_DNA-binding |
| **Sapur.018G053100** | Myb-like DNA-binding domain | Myb_DNA-binding |
| **Sapur.018G056100** | Myb/SANT-like | Myb_DNA-bind_4 |

| | | |
|---|---|---|
| | DNA-binding domain | |
| **Sapur.018G099900** | SANT SWI3, ADA2, N-CoR and TFIIIB'' DNA-binding domains | Myb_DNA-binding |
| **Sapur.019G026800** | Myb-related protein Myb4-like | Myb_DNA-binding |
| **Sapur.019G027000** | cell division cycle 5-like | Myb_Cef,Myb_DNA-binding |
| **Sapur.019G027500** | cell division cycle 5-like | Myb_Cef,Myb_DNA-binding |
| **Sapur.019G029200** | PHR1-LIKE 1-like | Myb_CC_LHEQLE,Myb_DNA-binding |
| **Sapur.019G029200** | PHR1-LIKE 1-like | Myb_CC_LHEQLE,Myb_DNA-binding |
| **Sapur.019G034000** | MYB-CC type transfactor, LHEQLE motif | Myb_CC_LHEQLE,Myb_DNA-binding |
| **Sapur.019G034000** | MYB-CC type transfactor, LHEQLE motif | Myb_CC_LHEQLE,Myb_DNA-binding |
| **Sapur.019G034000** | MYB-CC type transfactor, LHEQLE motif | Myb_CC_LHEQLE,Myb_DNA-binding |
| **Sapur.019G034000** | MYB-CC type transfactor, LHEQLE motif | Myb_CC_LHEQLE,Myb_DNA-binding |
| **Sapur.019G034000** | MYB-CC type transfactor, LHEQLE motif | Myb_CC_LHEQLE,Myb_DNA-binding |
| **Sapur.019G035400** | transcription repressor | Myb_DNA-binding |
| **Sapur.019G035500** | transcription repressor | Myb_DNA-binding |
| **Sapur.019G035600** | transcription repressor | Myb_DNA-binding |
| **Sapur.019G040700** | transcription factor | Myb_DNA-binding |
| **Sapur.019G052200** | Myb SANT-like DNA-binding domain protein | Myb_DNA-bind_3 |
| **Sapur.15WG031800** | Myb-related protein | Myb_DNA-binding |
| **Sapur.15WG032500** | SANT SWI3, ADA2, N-CoR and TFIIIB'' DNA-binding domains | Myb_DNA-binding |
| **Sapur.15ZG002300** | Nascent polypeptide-associated complex subunit alpha-like protein | NAC |
| **Sapur.15ZG016800** | transcription factor | Myb_DNA-binding |
| **Sapur.15ZG023200** | Nascent polypeptide-associated complex subunit beta | NAC |
| **Sapur.15ZG023800** | transcription, DNA-templated | Myb_DNA-binding |
| **Sapur.15ZG024800** | Transcription repressor KAN1-like | Myb_DNA-binding |
| **Sapur.15ZG035700** | Myb-related protein | Myb_DNA-binding |

| | | |
|---|---|---|
| **Sapur.15ZG036300** | SANT SWI3, ADA2, N-CoR and TFIIIB'' DNA-binding domains | Myb_DNA-binding |
| **Sapur.15ZG070400** | Transcription factor | Myb_DNA-binding |
| **Sapur.15ZG070700** | transcription factor | Myb_DNA-binding |
| **Sapur.15ZG070700** | transcription factor | Myb_DNA-binding |
| **Sapur.15ZG083300** | transcription factor | Myb_DNA-binding |
| **Sapur.15ZG099300** | SWI SNF complex subunit | Myb_DNA-binding,SURF2,SWIRM,SWIRM-assoc_1 |
| **Sapur.15ZG099300** | SWI SNF complex subunit | Myb_DNA-binding,SURF2,SWIRM,SWIRM-assoc_1 |
| **Sapur.T005200** | Myb SANT-like DNA-binding domain protein | Myb_DNA-bind_3 |
| **Sapur.T045600** | Plant lipoxygenase may be involved in a number of diverse aspects of plant physiology including growth and development, pest resistance, and senescence or responses to wounding | Lipoxygenase,PLAT |
| **Sapur.T045600** | Plant lipoxygenase may be involved in a number of diverse aspects of plant physiology including growth and development, pest resistance, and senescence or responses to wounding | Lipoxygenase,PLAT |
| **Sapur.T045600** | Plant lipoxygenase may be involved in a number of diverse aspects of plant physiology including growth and development, pest resistance, and senescence or responses to wounding | Lipoxygenase,PLAT |
| **Sapur.T050100** | HSA | HSA,Myb_DNA-bind_6 |
| **Sapur.T050100** | HSA | HSA,Myb_DNA-bind_6 |
| **Sapur.T050100** | HSA | HSA,Myb_DNA-bind_6 |
| **Sapur.T050100** | HSA | HSA,Myb_DNA-bind_6 |
| **Sapur.T050100** | HSA | HSA,Myb_DNA-bind_6 |
| **Sapur.T050100** | HSA | HSA,Myb_DNA-bind_6 |
| **Sapur.T050100** | HSA | HSA,Myb_DNA-bind_6 |
| **Sapur.T050100** | HSA | HSA,Myb_DNA-bind_6 |
| **Sapur.T050100** | HSA | HSA,Myb_DNA-bind_6 |
| **Sapur.T050100** | HSA | HSA,Myb_DNA-bind_6 |

| | | |
|---|---|---|
| **Sapur.T050100** | HSA | HSA,Myb_DNA-bind_6 |
| **Sapur.T050100** | HSA | HSA,Myb_DNA-bind_6 |
| **Sapur.T050100** | HSA | HSA,Myb_DNA-bind_6 |
| **Sapur.T050100** | HSA | HSA,Myb_DNA-bind_6 |
| **Sapur.T050100** | HSA | HSA,Myb_DNA-bind_6 |
| **Sapur.T050100** | HSA | HSA,Myb_DNA-bind_6 |
| **Sapur.T051600** | Fanconi anemia group F protein (FANCF) | FANCF,Myb_CC_LHEQLE,Myb_DNA-binding |
| **Sapur.T051600** | Fanconi anemia group F protein (FANCF) | FANCF,Myb_CC_LHEQLE,Myb_DNA-binding |
| **Sapur.T080900** | Integrator complex subunit | Beta-Casp,Lactamase_B_6 |
| **Sapur.T080900** | Integrator complex subunit | Beta-Casp,Lactamase_B_6 |
| **Sapur.T080900** | Integrator complex subunit | Beta-Casp,Lactamase_B_6 |
| **Sapur.T080900** | Integrator complex subunit | Beta-Casp,Lactamase_B_6 |
| **Sapur.T080900** | Integrator complex subunit | Beta-Casp,Lactamase_B_6 |
| **Sapur.T080900** | Integrator complex subunit | Beta-Casp,Lactamase_B_6 |
| **Sapur.T080900** | Integrator complex subunit | Beta-Casp,Lactamase_B_6 |
| **Sapur.T080900** | Integrator complex subunit | Beta-Casp,Lactamase_B_6 |
| **Sapur.T083400** | HSA | HSA,Myb_DNA-bind_6 |
| **Sapur.T083400** | HSA | HSA,Myb_DNA-bind_6 |
| **Sapur.T083900** | HSA | HSA,Myb_DNA-bind_6 |
| **Sapur.T099600** | HSA | HSA,Myb_DNA-bind_6 |
| **Sapur.T100500** | HSA | HSA,Myb_DNA-bind_6 |
| **Sapur.T116600** | Myb-like DNA-binding domain | Myb_DNA-binding |
| **Sapur.T141400** | HSA | HSA,Myb_DNA-bind_6 |
| **Sapur.T162700** | HSA | HSA,Myb_DNA-bind_6 |
