# Supplementary material for: Insights into Molecular Mechanism of Secondary Xylem Rapid Growth in Salix psammophila
Source: Plants (Basel). 2025 Feb 5;14(3):459. doi: 10.3390/plants14030459 (PMC11819810; doi:10.3390/plants14030459)
Supplement: Supplementary file 1 [file plants-14-00459-s001.zip › Supplementary Table/Table S8.pdf]

Table S8 Genes regulating yellow module-related transcription factors.

X	Description	PFAMs
**Sapur.002G128700**	Transcription factor	Myb_DNA-binding
**Sapur.002G176600**	Transcription factor	Myb_DNA-binding
**Sapur.005G207700**	PLATZ transcription factor	PLATZ
**Sapur.005G207700**	PLATZ transcription factor	PLATZ
**Sapur.005G207700**	PLATZ transcription factor	PLATZ
**Sapur.005G207700**	PLATZ transcription factor	PLATZ
**Sapur.006G047700**	PLATZ transcription factor	PLATZ
**Sapur.006G079900**	transcription factor	Myb_DNA-binding
**Sapur.006G182500**	transcription factor	Myb_DNA-binding
**Sapur.007G022500**	SANT SWI3, ADA2, N-CoR and TFIIIB'' DNA-binding domains	Myb_DNA-binding
**Sapur.008G028100**	Synaptotagmin-3-like	C2,SMP_LBD
**Sapur.010G070500**	PLATZ transcription factor	PLATZ,zf-B_box
**Sapur.013G121100**	Transcription factor	Myb_DNA-binding
**Sapur.014G025100**	transcription factor	Myb_DNA-binding
**Sapur.014G062600**	Transcription factor	Myb_DNA-binding
**Sapur.017G060800**	transcription factor	Myb_DNA-binding
**Sapur.018G090300**	PLATZ transcription factor	PLATZ
**Sapur.15ZG079600**	Transcription factor MYB98-like	Myb_DNA-binding
**Sapur.T175900**	HSA	HSA,Myb_DNA-bind_6
