# Supplementary material for: Insights into Molecular Mechanism of Secondary Xylem Rapid Growth in Salix psammophila
Source: Plants (Basel). 2025 Feb 5;14(3):459. doi: 10.3390/plants14030459 (PMC11819810; doi:10.3390/plants14030459)
Supplement: Supplementary file 1 [file plants-14-00459-s001.zip › Supplementary Table/Table S9.pdf]

Table S9 Genes regulating blue module-related transcription factors.

X	Description	PFAMs
**Sapur.001G081600**	Transcription factor	Myb_DNA-binding
**Sapur.001G097600**	Myb-like DNA-binding domain	Myb_DNA-binding
**Sapur.001G115800**	transcription factor	Myb_DNA-binding
**Sapur.001G115800**	transcription factor	Myb_DNA-binding
**Sapur.001G125100**	Myb/SANT-like DNA-binding domain	Myb_DNA-bind_4
**Sapur.001G172200**	Myb SANT-like DNA-binding domain protein	Myb_DNA-bind_3
**Sapur.002G057700**	transcription factor	Myb_DNA-binding
**Sapur.002G151600**	SANT SWI3, ADA2, N-CoR and TFIIIB'' DNA-binding domains	Myb_DNA-binding
**Sapur.003G052300**	Myb/SANT-like DNA-binding domain	Myb_DNA-bind_4
**Sapur.003G061400**	transcription factor	Myb_DNA-binding
**Sapur.003G070200**	Trihelix transcription factor	Myb_DNA-bind_4
**Sapur.003G070200**	Trihelix transcription factor	Myb_DNA-bind_4
**Sapur.003G079100**	Myb-like DNA-binding domain	Myb_DNA-binding
**Sapur.003G095800**	Transcription factor	Myb_DNA-binding
**Sapur.004G164600**	PLATZ transcription factor	PLATZ
**Sapur.005G047800**	myb domain protein 69	Myb_DNA-binding
**Sapur.005G076300**	Myb-related protein	Myb_DNA-binding
**Sapur.005G146300**	transcription factor	Myb_DNA-binding
**Sapur.005G162100**	Myb-like DNA-binding domain	Myb_DNA-binding
**Sapur.006G098200**	PLATZ transcription factor	PLATZ
**Sapur.006G098200**	PLATZ transcription factor	PLATZ

**Sapur.006G098200**	PLATZ transcription factor family protein	PLATZ
**Sapur.007G070800**	Transcription factor	Myb_DNA-binding
**Sapur.007G070800**	Transcription factor	Myb_DNA-binding
**Sapur.007G070800**	Transcription factor	Myb_DNA-binding
**Sapur.007G070800**	Transcription factor	Myb_DNA-binding
**Sapur.007G070800**	Transcription factor	Myb_DNA-binding
**Sapur.007G070800**	Transcription factor	Myb_DNA-binding
**Sapur.007G070800**	Transcription factor	Myb_DNA-binding
**Sapur.007G070800**	Transcription factor	Myb_DNA-binding
**Sapur.007G095000**	myb domain protein 69	Myb_DNA-binding
**Sapur.007G121100**	SANT SWI3, ADA2, N-CoR and TFIIIB" DNA-binding domains	Myb_DNA-binding
**Sapur.008G049800**	SANT SWI3, ADA2, N-CoR and TFIIIB" DNA-binding domains	Myb_DNA-binding
**Sapur.008G094600**	transcription factor	Myb_DNA-binding
**Sapur.008G094600**	transcription factor	Myb_DNA-binding
**Sapur.009G016500**	Myb-like DNA-binding domain	Bromodomain,Myb_DNA-binding
**Sapur.009G016500**	Myb-like DNA-binding domain	Bromodomain,Myb_DNA-binding
**Sapur.009G040700**	transcription factor	Myb_DNA-binding
**Sapur.009G106600**	Myb-related protein 308-like	Myb_DNA-binding
**Sapur.012G025900**	SANT SWI3, ADA2, N-CoR and TFIIIB" DNA-binding domains	Myb_DNA-binding
**Sapur.012G097600**	Myb-like DNA-binding domain	Myb_DNA-binding
**Sapur.012G108200**	SANT SWI3, ADA2, N-CoR and TFIIIB" DNA-binding domains	Myb_DNA-binding
**Sapur.012G108200**	SANT SWI3, ADA2, N-CoR and TFIIIB" DNA-binding domains	Myb_DNA-binding

**Sapur.014G088200**	SANT SWI3, ADA2, N-CoR and TFIIIB'' DNA-binding domains	Myb_DNA-binding
**Sapur.014G090700**	SANT SWI3, ADA2, N-CoR and TFIIIB'' DNA-binding domains	Myb_DNA-binding
**Sapur.014G098200**	SANT SWI3, ADA2, N-CoR and TFIIIB'' DNA-binding domains	Myb_DNA-binding,P_C
**Sapur.016G055600**	Homeobox-leucine zipper protein	HALZ,HD-ZIP_N,Homeobox
**Sapur.016G124000**	Homeobox-leucine zipper protein	HALZ,HD-ZIP_N,Homeobox
**Sapur.016G135600**	transcription factor	Myb_DNA-binding
**Sapur.016G147700**	transcription factor	Myb_DNA-binding
**Sapur.016G293500**	Trihelix transcription factor	Myb_DNA-bind_4
**Sapur.016G293500**	Trihelix transcription factor	Myb_DNA-bind_4
**Sapur.016G293500**	Trihelix transcription factor	Myb_DNA-bind_4
**Sapur.016G293500**	Trihelix transcription factor	Myb_DNA-bind_4
**Sapur.016G293500**	Trihelix transcription factor	Myb_DNA-bind_4
**Sapur.017G010300**	transcription factor	Myb_DNA-binding
**Sapur.017G107600**	ODORANT1-like	Myb_DNA-binding
**Sapur.018G004700**	transcription factor	Myb_DNA-binding
**Sapur.018G004700**	transcription factor	Myb_DNA-binding
**Sapur.15ZG026100**	SANT SWI3, ADA2, N-CoR and TFIIIB'' DNA-binding domains	Myb_DNA-binding
**Sapur.15ZG120300**	Myb-like DNA-binding domain	Myb_DNA-binding
