# Supplementary figures and images for: Insights into Molecular Mechanism of Secondary Xylem Rapid Growth in Salix psammophila
Source: Plants (Basel). 2025 Feb 5;14(3):459. doi: 10.3390/plants14030459 (PMC11819810; doi:10.3390/plants14030459)

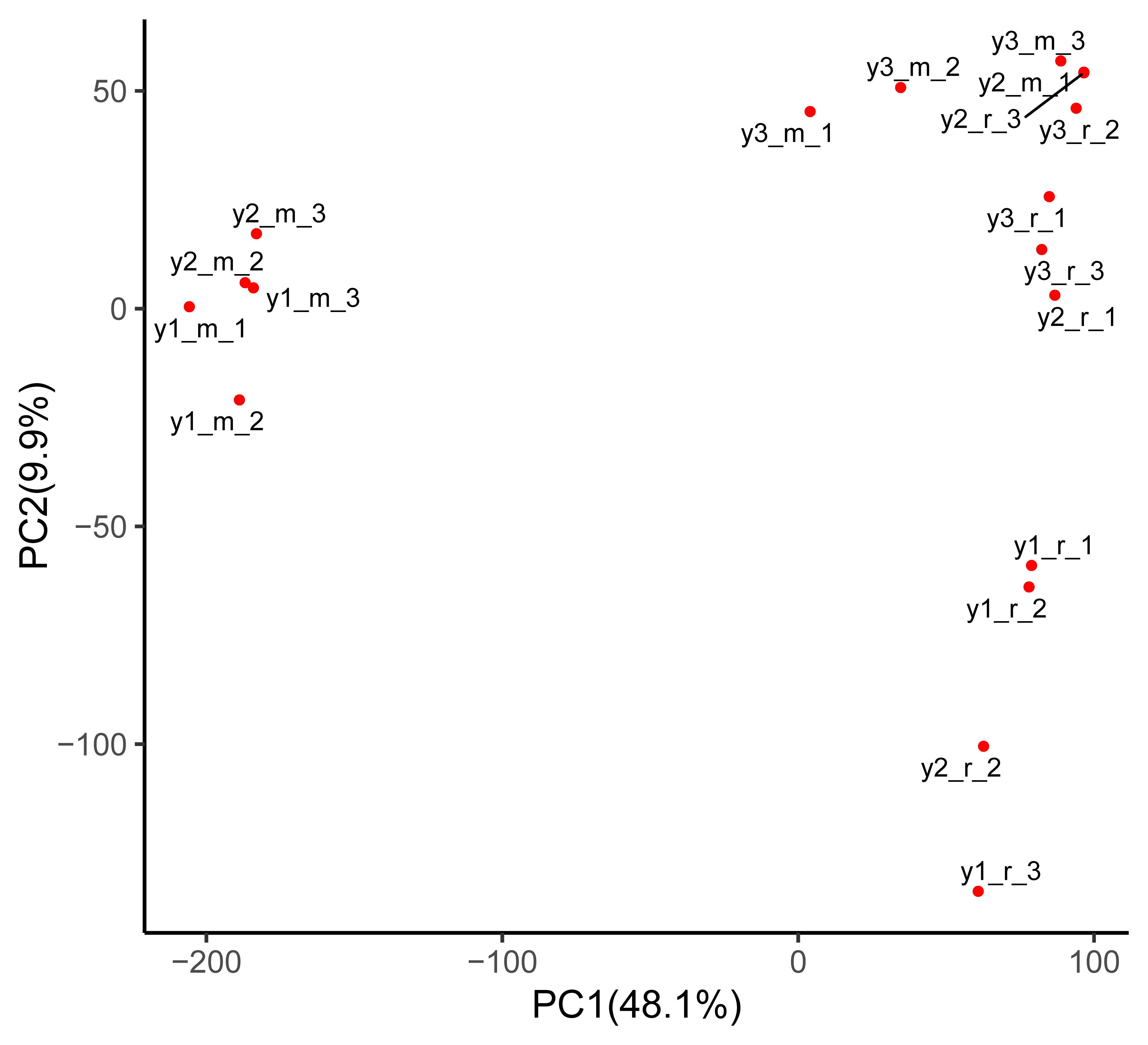

Supplement: Supplementary file 1 [file plants-14-00459-s001.zip › Supplementary Figure/Figure S2.tif]

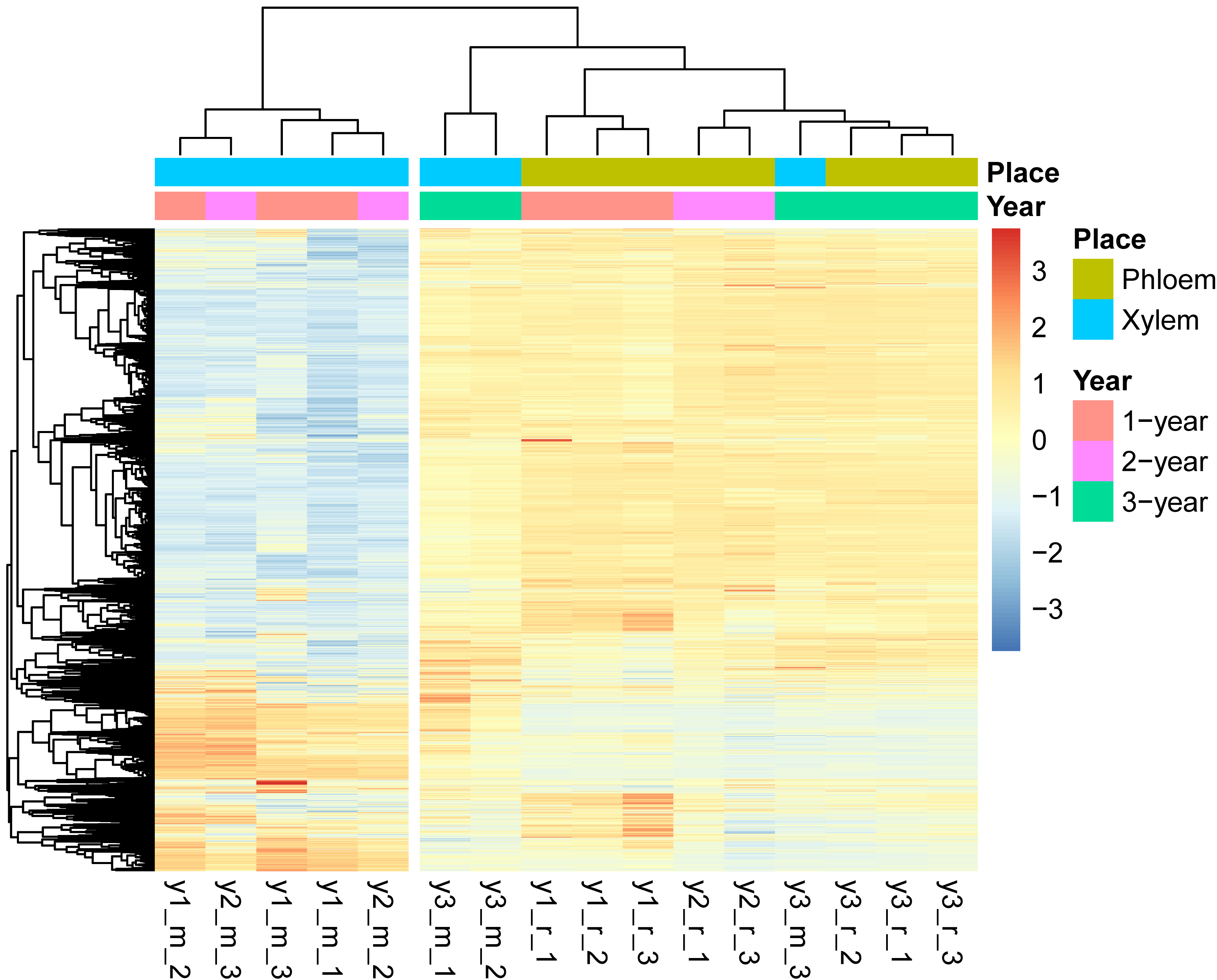

Supplement: Supplementary file 1 [file plants-14-00459-s001.zip › Supplementary Figure/Figure S3.tif]
